# SLX4IP limits replication stress globally and at ALT telomeres

**DOI:** 10.1038/s44318-026-00790-4

**Published:** 2026-05-07

**Authors:** Jessica Spindler, Francesca Pandolfo, Anna Eva Koch, Priscilla Piccirillo, Drew Jordahl, Nikhil Venkatesh, Dhruthi Suresh, K R Ylvisaker, Anita Jopkiewicz, Johanna Bihler, Sandra Buschbaum, Marcel Morgenstern, Katherine A Overmyer, Estelle Vincendeau, Joshua J Coon, Pei-Chi Wei, Robert Hänsel-Hertsch, Kavi P M Mehta, Stephanie Panier

**Affiliations:** 1 Max Planck Institute for Biology of Ageing, Cologne, Germany; 2 Center for Molecular Medicine Cologne (CMMC), University of Cologne, Cologne, Germany; 3 Department of Biomolecular Chemistry, School of Medicine and Public Health, University of Wisconsin-Madison, Madison, WI USA; 4 National Center for Quantitative Biology of Complex Systems, University of Wisconsin-Madison, Madison, WI USA; 5 Department of Comparative Biosciences, School of Veterinary Medicine, University of Wisconsin-Madison, Madison, WI USA; 6 Morgridge Institute for Research, Madison, WI USA; 7 Department of Chemistry, University of Wisconsin-Madison, Madison, WI USA; 8 Brain Mosaicism and Tumorigenesis, German Cancer Research Center, Heidelberg, Germany; 9 Interdisciplinary Center for Neuroscience, Faculty of Biosciences, Heidelberg University, Heidelberg, Germany; 10 Department of Translational Genomics, Faculty of Medicine, University of Cologne, Cologne, Germany; 11 Institute of Human Genetics, University Hospital Cologne, Cologne, Germany; 12 University of Cologne, Faculty of Medicine and University Hospital Cologne, Cologne Excellence Cluster for Aging and Aging-Associated Diseases (CECAD), Cologne, Germany; 13 Institute for Genome Stability in Aging and Disease, Medical Faculty, University of Cologne, Cologne, Germany

**Keywords:** DNA Replication, Recombination & Repair

## Abstract

Faithful DNA replication is essential for genome stability, yet replication forks face constant stress. The Bloom syndrome helicase (BLM) safeguards fork integrity, but excessive BLM activity can itself induce replication stress. We identify SLX4IP as a genome-wide regulator that restrains BLM to maintain replication fork stability. SLX4IP localizes broadly across chromatin with recruitment enhanced under replication stress. Loss of SLX4IP slows replication forks, remodels the replisome, and generates post-replicative single-stranded DNA gaps that are accompanied by elevated nuclear ADP ribose, reflecting compromised replication integrity. These defects are driven by dysregulated BLM activity, establishing SLX4IP as a negative regulator of BLM-dependent replication stress. At ALT telomeres, SLX4IP deficiency triggers ATR signaling, telomere fragility, and accumulation of ALT-associated PML bodies. Here, SLX4IP functions in parallel with FANCM to restrain BLM at ALT telomeres, with co-depletion of SLX4IP and FANCM causing synthetic lethality in ALT-positive cells, a phenotype fully rescued by BLM loss. Together, our results define SLX4IP as a critical genome-wide regulator of replication fork integrity and reveal SLX4IP as a potential vulnerability in ALT-positive cancers.

## Synopsis

BLM helicase safeguards replication forks but can itself also cause replication stress. This work identifies SLX4IP as a genome-wide brake on BLM helicase activity whose loss unleashes BLM-driven replicative stress, and reveals a potential Achilles’ heel of ALT-positive cancers.

- SLX4IP associates with chromatin genome-wide.
- SLX4IP depletion causes global replication stress.
- ALT telomeres are particularly susceptible to SLX4IP-dependent replication stress.
- SLX4IP acts in parallel to FANCM to limit replication stress at ALT telomeres.
- The synthetic lethal interaction between SLX4IP and FANCM is dependent on BLM.

## Abstract (web summary)

SLX4IP acts as a genome-wide brake on BLM helicase activity to preserve replication fork stability under stress.

## Graphical abstract

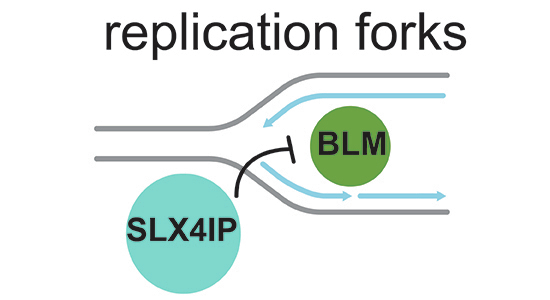

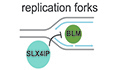

## Introduction

Faithful DNA replication is essential for genome stability, yet replication forks are continuously challenged by DNA damage and other obstacles that threaten their progression (Saxena and Zou, 2022). Failure to properly manage replication stress and prevent replication fork collapse is a major source of genome instability and a common feature of cancer cells (Macheret and Halazonetis, 2015). To stabilize stalled forks and promote their recovery, cells mount an ATR-mediated replication checkpoint, fire dormant origins, dynamically remodel fork architecture, and activate DNA damage tolerance pathways (Zeman and Cimprich, 2014). These pathways include translesion synthesis (TLS), which enables DNA synthesis across damaged templates; the specialized polymerase PRIMPOL that de novo reprimes downstream from lesions, creating single-stranded DNA gaps; and replication fork reversal, which reconfigures stalled forks to place lesions back into double-stranded DNA for subsequent repair (García-Gómez et al, 2013; Mehta et al, 2022; Quinet et al, 2018). Besides repriming, gaps are also thought to arise when the helicase uncouples from replicative polymerases as well as during defects in Okazaki fragment processing (Fielden et al, 2025; Kahli et al, 2019; Byun et al, 2005; Machacova et al, 2024).

The Bloom syndrome helicase (BLM) is a RecQ family DNA helicase that plays key roles in maintaining genome stability, particularly under conditions of replication stress (Sidorova et al, 2013). In the absence of BLM, cells exhibit multiple defects in DNA replication, including accumulation of abnormal DNA replication intermediates, reduced replication fork speed, and increased firing of dormant origins (Rao et al, 2007; Lönn et al, 1990; Davies et al, 2007). BLM, as a component of the BLM-Topoisomerase IIIα-RMI1/2 (BTR) complex, localizes to stalled replication forks and recognizes RPA-coated single-stranded (ss) DNA at stalled fork structures to initiate appropriate repair or restart responses (Davies et al, 2004, 2007; Shorrocks et al, 2021; Brosh et al, 2000; Sengupta et al, 2003). It cooperates with the replisome and genome maintenance factors to remodel stalled forks, promote fork reversal and limited resection, and channel lesions into error‑free, recombination-mediated restart rather than fork collapse (Sidorova et al, 2013; Manthei and Keck, 2013).

While BLM is essential for replication fork recovery, its activity and turnover at replication forks must be tightly controlled as BLM exhibits both pro- and antirecombinogenic activities (Kaur et al, 2021; Bugreev et al, 2007). Aberrant BLM function can drive excessive fork speed and firing of dormant origins, increase fork collapse, and lead to the toxic accumulation of recombination intermediates (Chen et al, 2024; Ellis et al, 2021; Ouyang et al, 2009, 2013; Sobinoff et al, 2017; Panier et al, 2019). Thus, the maintenance of genome integrity depends not only on BLM activity itself, but also on mechanisms that restrain and coordinate its function.

Telomeres represent a genomic region in which the challenges of DNA replication are particularly acute. Their repetitive DNA, propensity to form DNA secondary structures, and unique chromatin environment render telomeres intrinsic hotspots of replication stress that rely heavily on efficient fork stabilization and recovery pathways (Sfeir et al, 2009). These challenges are further amplified in cancers that maintain telomeres through the Alternative Lengthening of Telomeres (ALT) pathway.

A defining feature of ALT-positive cancer cells is the presence of sustained replication stress at telomeres (Arora et al, 2014; O’Sullivan et al, 2014; Flynn et al, 2015; Cesare et al, 2009). This stress arises from the structured and dynamic nature of telomeric DNA, which promotes the formation of secondary structures, RNA-DNA hybrids, and DNA lesions that impede replication fork progression (Thosar et al, 2024; Lu and Pickett, 2022). As a consequence, ALT telomeres exhibit persistent activation of ATR-dependent replication stress signaling (Zhao and Piwnica-Worms, 2001; Liu et al, 2012; Silva et al, 2019; Pan et al, 2019).

Importantly, replication stress is not merely tolerated in ALT cells but functionally integrated into the telomere maintenance mechanism. Replication-associated DNA intermediates provide substrates for recombination-coupled DNA synthesis that drives telomere elongation in a process called break-induced replication (BIR) (Lu and Pickett, 2022). However, replication and recombination activities must be tightly balanced to prevent the accumulation of excessive DNA damage and cytotoxic intermediates, including aberrant telomeric DNA structures (Pan et al, 2017; Panier et al, 2019; Lu et al, 2019; Silva et al, 2019; Pan et al, 2019; Sobinoff et al, 2017). Thus, ALT-positive cells operate within a narrow window in which replication stress supports productive telomere extension while avoiding toxic genome instability (O’Sullivan and Greenberg, 2025).

BLM plays a central role in maintaining this balance. While BLM localizes to both ALT and non-ALT telomeres, it is particularly enriched at ALT telomeres, where it promotes recombination-associated processes required for ALT activity (Barefield and Karlseder, 2012; Stavropoulos, 2002; Bhargava et al, 2022). Consistent with this role, BLM deficiency impairs ALT phenotypes and leads to telomere shortening (O’Sullivan et al, 2014; Loe et al, 2020). Conversely, unrestrained BLM activity results in hyper-ALT phenotypes that are characterized by elevated replication stress, excessive DNA synthesis, and accumulation of pathological recombination intermediates that compromise cellular viability (Panier et al, 2019; Lu et al, 2019; Sobinoff et al, 2017; Root et al, 2016; Jiang et al, 2024). Therefore, precise regulation of BLM activity is essential for ALT-positive cells.

ALT cells deploy multiple mechanisms to restrain BLM-dependent replication and recombination. A prominent example is FANCM, an ATPase/translocase, that limits BLM-driven replication stress by resolving persistent DNA structures and facilitating replication fork remodeling (Pan et al, 2017, 2019; Lu et al, 2019; Silva et al, 2019). Disruption of the FANCM-BLM interaction is synthetic lethal in ALT-positive cells, highlighting the critical importance of BLM regulation in this context (Lu et al, 2019).

SLX4IP, a SLX4-interacting scaffolding protein, has previously been characterized primarily in the context of telomere maintenance (Panier et al, 2019; Robinson et al, 2020, 2021; Mangosh et al, 2021). We showed that SLX4IP limits BLM-dependent recombination intermediate dissolution at ALT telomeres (Panier et al, 2019). Loss of SLX4IP causes a BLM-dependent hyper-ALT phenotype characterized by increased APB formation, elevated levels of extrachromosomal telomeric circles, and accumulation of catenated telomere aggregates. While these findings established SLX4IP as a modulator of telomeric DNA transactions, they did not resolve the broader physiological function of SLX4IP. In particular, it has remained unclear whether SLX4IP functions exclusively within telomere-specific pathways or instead participates more generally in replication- or recombination-associated genome maintenance processes. This distinction is especially important given that many factors initially identified at telomeres have subsequently been shown to play wider roles in DNA replication and repair. Whether SLX4IP participates in the cellular response to replication-associated stress, and how its functional relationship with BLM extends beyond telomeres, has not been resolved.

Here, we identify SLX4IP as a genome-wide regulator of replication stress that preserves replication fork integrity by restraining BLM activity. While SLX4IP functions broadly across chromatin, ALT telomeres exhibit exceptional sensitivity to its loss. Mechanistically, SLX4IP operates in parallel with FANCM at ALT telomeres, and co-depletion of both factors induces BLM-dependent replication stress and synthetic lethality, revealing a critical dependency in ALT-positive cells.

## Results

### SLX4IP occupies the chromatin genome-wide and dynamically responds to replication stress

**Figure 1:**
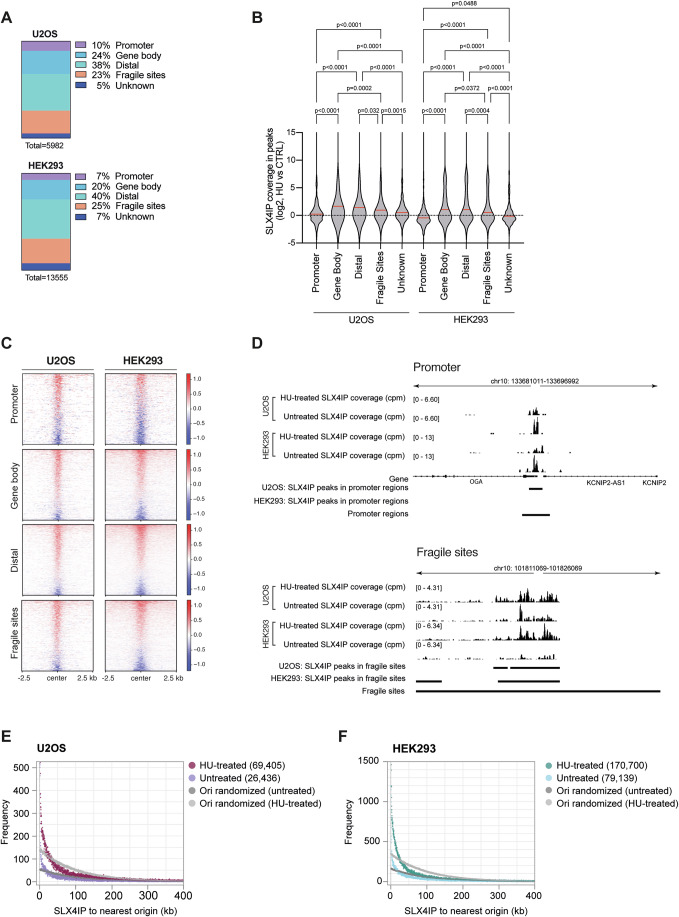
SLX4IP occupies the chromatin genome-wide and dynamically responds to replication stress. (**A**) U2OS and HEK293 cells were processed for CUT&Tag analysis. Shown is the genomic annotation of SLX4IP ChIP-seq peaks in U2OS (upper panel) and HEK293 (lower panel) cells, displayed as the percentage of peaks falling within promoters, gene bodies, distal intergenic regions, and fragile sites. (**B**) U2OS and HEK293 cells were treated with 4 mM hydroxyurea (HU) for 24 h and processed for CUT&Tag analysis. Shown is the distribution of the log_2_ ratio of normalized SLX4IP coverage (RPKM, reads per kilobase per million) after HU treatment versus untreated control. Each point represents one peak; a value > 0 denotes increased occupancy, <0 denotes decreased occupancy. Red line in violin plot shows median ratio (log_2_); one-way ANOVA with Tukey’s multiple comparisons post-test. Exact *P* values are shown in the figure; *P* < 0.05 was considered statistically significant. (**C**) Tornado heat maps showing the HU-to-CTRL coverage ratio for individual SLX4IP peaks, sorted within each genomic category in (**A**). Red indicates higher, blue lower SLX4IP signal after HU treatment. (**D**) Genome-browser snapshots of two representative promoter (upper panel) and fragile site (lower panel) loci. Shown is SLX4IP binding following HU exposure. Signal tracks are shown as log_2_-normalized read coverage. Coverage is shown as counts per million (cpm). (**E**) U2OS cells were treated with 4 mM hydroxyurea (HU) for 24 h and processed for CUT&Tag analysis. Shown is the distance between SLX4IP peaks and conserved human replication origins. Significance of SLX4IP enrichment above random distribution was assessed by a permutation test; *P* < 0.0001. (**F**) HEK293 cells were treated with 4 mM hydroxyurea (HU) for 24 h and processed for CUT&Tag analysis. Shown is the distance between SLX4IP peaks and conserved human replication origins. Significance of SLX4IP enrichment above random distribution was assessed by a permutation test. The *P* value is *P* < 0.0001 and was considered statistically significant.

SLX4IP has primarily been characterized as a telomere-associated factor, particularly in the context of ALT. However, whether SLX4IP exerts functions outside telomeric regions has remained unresolved. To uncover where SLX4IP binds to chromatin, we generated CUT&Tag maps of ALT-positive U2OS cells and ALT-negative HEK293 cells. This approach revealed that SLX4IP is broadly distributed across chromatin. In both cell types, SLX4IP peaks were detected at promoters (10% in U2OS, 7% in HEK293), gene bodies (24% in U2OS, 20% in HEK293), distal intergenic regions (38% in U2OS, 40% in HEK293), and fragile sites (23% in U2OS, 25% in HEK293), indicating that SLX4IP is not only restricted to telomeric regions (Fig. 1A).

**Figure EV1:**
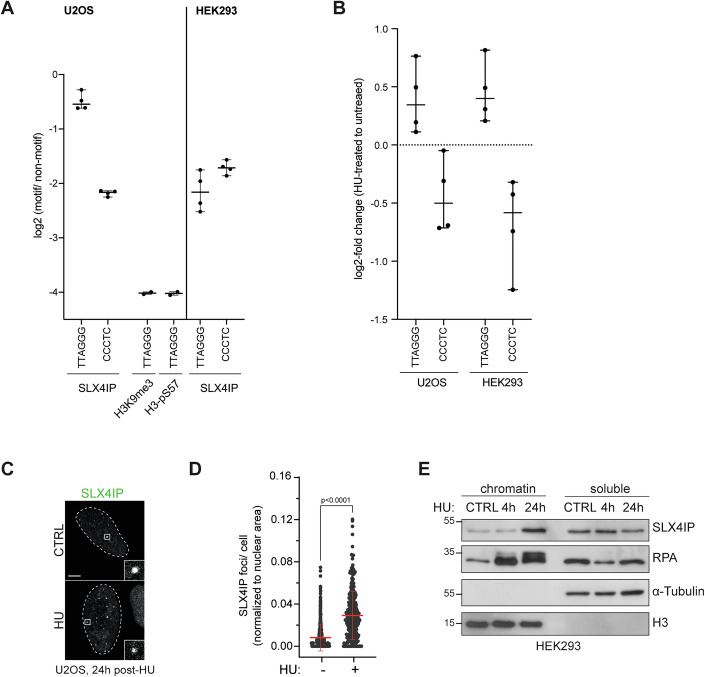
Replication stress-induced increase in SLX4IP chromatin association. (**A**) Regex analysis of TTAGGG and CCCTC motifs. TTAGGG and CCCTC repeats were defined as regex search on the sequences for each SLX4IP-enriched peak with fastaRegexFinder. The motif/non-motif ratio was calculated for each replicate. Replicate values were then statistically compared using a *t* test to determine whether the motif is significantly enriched. “non-motif” refers to all reads that do not contain the TTAGGG and CCCTC motif, respectively. Log2 enrichment was calculated from the SLX4IP CUT&Tag sequencing data (untreated) presented in Fig. 1. H3K9me3 and H3-pS57 sequencing data are from (Zhang et al, 2023; Parisis et al, 2023). Data are represented as median ± 95% confidence interval; 4 technical replicates (SLX4IP CUT&Tag). (**B**) Regex analysis of TTAGGG and CCCTC motifs to compare SLX4IP enrichment in untreated and hydroxyurea (HU)-treated cells. Log2 enrichment was calculated from the SLX4IP CUT&Tag sequencing data (untreated or treated with 4 mM hydroxyurea (HU) for 24 h) presented in Fig. 1. Data are represented as median ± 95% confidence interval; four technical replicates. (**C**) U2OS cells were either treated with 4 mM hydroxyurea (HU) or water as a control for 24 h. Cells were then immediately pre-extracted, fixed, and processed for SLX4IP immunofluorescence. Insets are 4X magnifications of the indicated fields. The dotted line represents DAPI (not shown). Scale bar represents 10 μm. (**D**) Quantification of (**C**). The number of SLX4IP foci in each cell was quantified and normalized to nuclear area to account for variations in nuclear size across cells. At least 100 cells per condition and experiment were counted. Data are represented as mean ± SD; *n* = 3, biological replicates; Student’s *t* test. The exact *P* value is shown in the figure; *P* < 0.05 was considered statistically significant. (**E**) HEK293 cells were either treated with 4 mM hydroxyurea (HU) or water as a control for 4 h or 24 h. A chromatin fractionation was performed, and the soluble and chromatin fractions were separated by SDS-PAGE and analyzed for SLX4IP and RPA32 levels by immunoblotting. α-Tubulin and H3 were used as loading controls. The numbers on the left denote the molecular weight in kDa.

To quantify telomeric association independent of the alignment constraints that are inherent to short-read sequencing, we performed repeat-based analysis by regex-driven counting of TTAGGG-containing reads directly from raw sequencing data (Thorn et al, 2022). This approach enables unbiased detection of DNA motifs independent of the mapping limitations inherent in short-read sequencing data. Using this strategy, we detected a relative enrichment of TTAGGG-containing reads in U2OS cells (Fig. EV1A). Although TTAGGG motif reads were less abundant than reads without the TTAGGG sequence, overall, their proportional representation was increased relative to that of CCCTC motifs, which correspond to CTCF-binding sites, indicating preferential recovery of telomeric sequences. To further validate the specificity of this repeat-based quantification, we also analyzed published CUT&Tag and ChIP-seq datasets from U2OS cells (H3K9me3 and pS57-H3) as negative controls (Zhang et al, 2023; Parisis et al, 2023). As expected, TTAGGG-derived signals were substantially less abundant in these datasets, confirming the specificity of the regex-based motif analysis that we applied to our SLX4IP CUT&Tag data (Fig. EV1A). Of note, in HEK293 cells, TTAGGG-containing reads were detectable but, unlike in U2OS cells, were not enriched relative to CCCTC motifs (Fig. EV1A). These findings are consistent with prior microscopy-based observations showing SLX4IP enrichment at telomeres in ALT-positive cells but not in ALT-negative cells (Panier et al, 2019).

Since SLX4IP exhibits widespread chromatin occupancy in ALT-positive and ALT-negative cells under basal conditions, we next asked whether its overall occupancy and genomic distribution are dynamically regulated following replication stress. Notably, upon 24 h treatment with 4 mM hydroxyurea (HU), a replication stress-inducing agent, SLX4IP occupancy showed an overall median increase across all genomic sites except promoters (Fig. 1B–D). Similarly, TTAGGG-containing reads were further enriched following HU exposure, consistent with increased telomeric association following exogenous replication stress, whereas CCCTC motifs were less enriched relative to non-treated conditions in both U2OS and HEK293 cells (Fig. EV1B). We confirmed the HU-dependent increase in SLX4IP chromatin association by performing SLX4IP immunofluorescence in pre-extracted U2OS cells, where HU treatment resulted in increased SLX4IP focus formation (Fig. EV1C,D). Consistently, chromatin fractionation coupled to immunoblotting showed elevated SLX4IP chromatin association also in HEK293 cells, most prominently 24 h post-HU exposure (Fig. EV1E).

Given this replication stress-induced increase of SLX4IP on chromatin, we next sought to determine whether SLX4IP accumulation exhibited positional bias relative to replication-associated genomic features. Specifically, we examined the distance between SLX4IP peaks and conserved human replication origins. We determined that enrichment was highest in the immediate vicinity of replication origins and progressively decreased with increasing distance, reaching low levels approximately 20 kb from the origin (Fig. 1E,F, blue and purple lines). To determine whether this pattern exceeded random expectation, we generated randomized origin coordinates by genome-wide shuffling while preserving origin number (Fig. 1E,F, gray lines). SLX4IP enrichment near replication origins significantly exceeded this randomized distribution and was further enhanced following HU treatment (Fig. 1E,F).

Collectively, these data indicate that SLX4IP is a chromatin-associated factor whose localization is dynamically modulated in response to replication stress. While telomeric enrichment appears to be an ALT-specific feature, the HU-induced accumulation of SLX4IP across genomic regions, including near replication origins in ALT-positive and ALT-negative cells, suggests a potential general role in replication stress-associated genome maintenance.

### SLX4IP deficiency causes global replication stress and alters replication fork composition

**Figure EV2:**
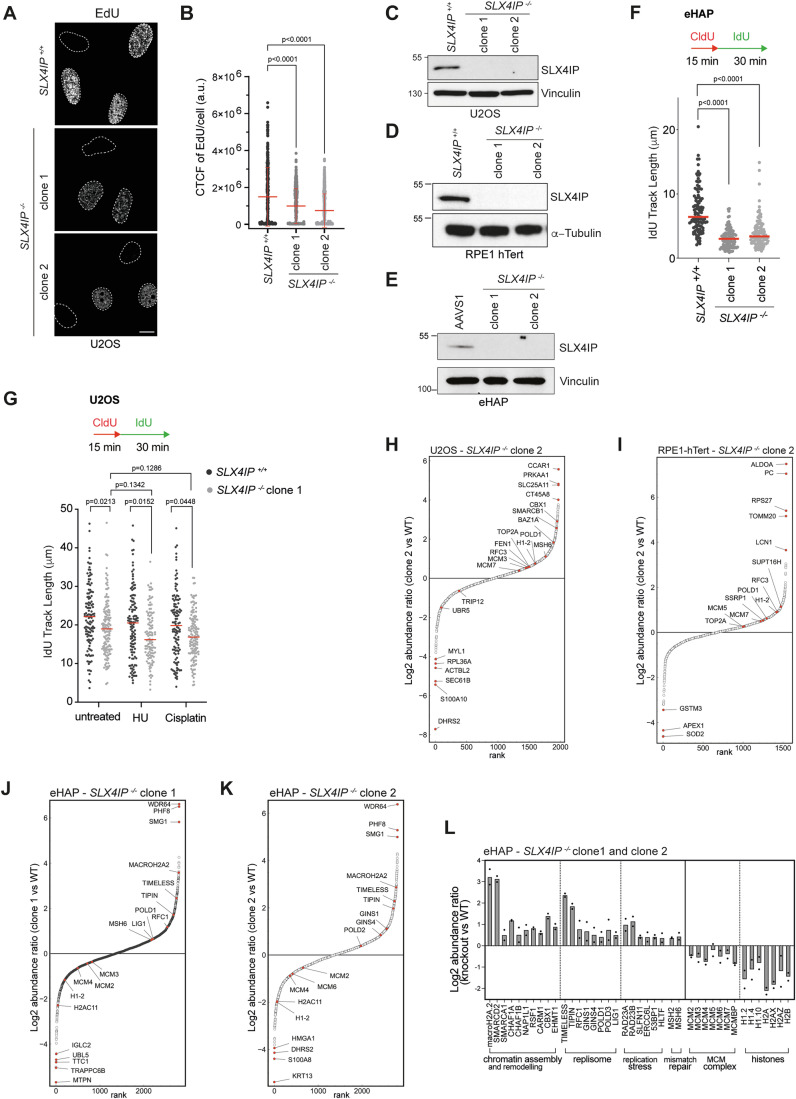
Replication stress analysis of SLX4IP-defi cient cells. (**A**) U2OS cells were subjected to a 30 min EdU pulse before fixation, followed by staining of EdU via a Click-IT reaction. The dotted line represents DAPI (not shown). Scale bar represents 10 μm. (**B**) Quantification of (**A**). The EdU signal was quantified as corrected total nuclear intensity per cell. At least 100 cells per condition and experiment were counted. Data are represented as mean ± SD; *n* = 3, biological replicates; one-way ANOVA with Dunnett’s multiple comparisons post-test. Exact *P* values are shown in the figure; *P* < 0.05 was considered statistically significant. (**C**) Whole-cell lysates of U2OS cells were separated by SDS-PAGE and analyzed for SLX4IP levels by immunoblotting. Vinculin was used as a loading control. The numbers on the left denote the molecular weight in kDa. (**D**) Whole-cell lysates of RPE1-hTert cells were separated by SDS-PAGE and analyzed for SLX4IP levels by immunoblotting. α -Tubulin was used as a loading control. The numbers on the left denote the molecular weight in kDa. (**E**) Whole-cell lysates of eHAP cells were separated by SDS-PAGE and analyzed for SLX4IP levels by immunoblotting. Vinculin was used as a loading control. The numbers on the left denote the molecular weight in kDa. (**F**) eHAP cells were labeled with CldU and IdU before DNA combing. Individual IdU fiber lengths are plotted. Median is indicated; *n* = 3, biological replicates; ANOVA with Dunn’s multiple comparisons post-test. Exact *P* values are shown in the figure; *P* < 0.05 was considered statistically significant. (**G**) U2OS cells were labeled with CldU and IdU for the indicated times before DNA combing. Samples were either with 50 μM hydroxyurea (HU; 30 min, during IdU pulse) or 50 μM Cisplatin (30 min, during IdU pulse). Individual IdU fiber lengths are plotted. Median is indicated; *n* = 3, biological replicates; one-way ANOVA with Dunn’s multiple comparisons post-test. Exact *P* values are shown in the figure; *P* < 0.05 was considered statistically significant. (**H**) U2OS were subjected to a 20 min EdU pulse to label newly synthesized DNA, followed by iPOND coupled to mass spectrometry. The graph shows the log_2_ ratio intensity of proteins accumulated at replication forks in U2OS *SLX4IP*^*−/−*^ clone 2 compared to *SLX4IP*^*+/+*^ cells. Related to Fig. 2C,E. (**I**) RPE1-hTert were subjected to a 20 min EdU pulse to label newly synthesized DNA, followed by iPOND coupled to mass spectrometry. The graph shows the log_2_ ratio intensity of proteins accumulated at replication forks in RPE1-hTert *SLX4IP*^*−/−*^ clone 2 compared to *SLX4IP*^*+/+*^ cells. Related to Fig. 2D,F. (**J**) eHAP were subjected to a 20 min EdU pulse to label newly synthesized DNA, followed by iPOND coupled to mass spectrometry. The graph shows the log_2_ ratio intensity of proteins accumulated at replication forks in *SLX4IP*^*−/−*^ clone 1 compared to *SLX4IP*^*+/+*^ cells. (**K**) eHAP were subjected to a 20 min EdU pulse to label newly synthesized DNA, followed by iPOND coupled to mass spectrometry. The graph shows the log_2_ ratio intensity of proteins accumulated at replication forks in *SLX4IP*^*−/−*^ clone 2 compared to *SLX4IP*^*+/+*^ cells. (**L**) Abundance ratios of proteins at nascent DNA for selected proteins across U2OS *SLX4IP*^−/−^ clone 1 and clone 2. Each data point corresponds to the abundance ratio in one clone; the mean is indicated.

**Figure 2:**
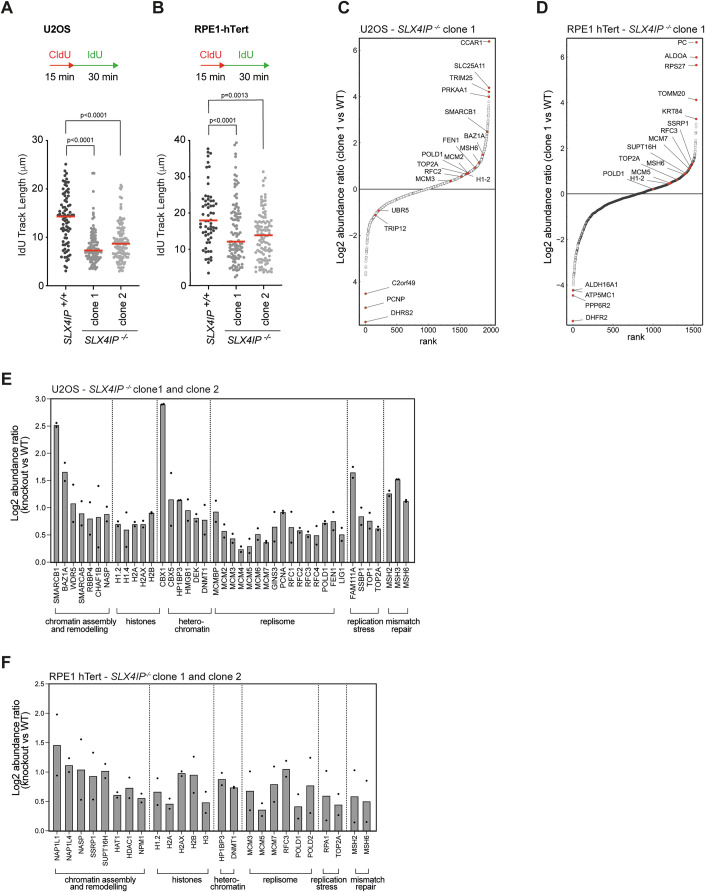
SLX4IP deficiency causes global replication stress and alters replication fork composition. (**A**) U2OS cells were labeled with CldU and IdU before DNA combing. Individual IdU fiber lengths are plotted. Median is indicated; *n* = 3, biological replicates; one-way ANOVA with Dunn’s multiple comparisons post-test. Exact *P* values are shown in the figure; *P *< 0.05 was considered statistically significant. (**B**) RPE1-hTert cells were labeled with CldU and IdU before DNA combing. Individual IdU fiber lengths are plotted. Median is indicated; *n* = 3, biological replicates; ANOVA with Dunn’s multiple comparisons post-test. Exact *P* values are shown in the figure; *P* < 0.05 was considered statistically significant. (**C**) U2OS cells were subjected to a 20 min EdU pulse to label newly synthesized DNA, followed by iPOND coupled to mass spectrometry. The rank plot shows the log_2_ ratio intensity of proteins accumulated at replication forks in U2OS *SLX4IP* ^*−/−*^ clone 1 compared to *SLX4IP* ^*+/+*^ cells. (**D**) RPE1-hTert cells were subjected to a 20 min EdU pulse to label newly synthesized DNA, followed by iPOND coupled to mass spectrometry. The rank plot shows the log_2_ ratio intensity of proteins at nascent DNA in RPE1-hTert *SLX4IP* ^*−/−*^ clone 1 compared to *SLX4IP* ^*+/+*^ cells. (**E**) Abundance ratios of proteins at nascent DNA for selected proteins across U2OS *SLX4IP *^−/−^ clone 1 and clone 2. Each data point corresponds to the abundance ratio in one clone; the mean is indicated. (**F**) Abundance ratios of proteins at nascent DNA for selected proteins across RPE1-hTert *SLX4IP*^−/−^ clone 1 and clone 2. Each data point corresponds to the abundance ratio in one clone; the mean is indicated. Source data are available online for this figure.

To determine whether SLX4IP contributes to DNA replication, we first assessed global DNA synthesis by measuring EdU incorporation. SLX4IP-deficient cells exhibited a marked reduction in EdU incorporation compared to wild-type cells, indicating impaired DNA synthesis (Fig. EV2A,B). We next examined the replication dynamics underlying this effect in SLX4IP-deficient cells by performing DNA molecular combing (Herrick and Bensimon, 2009). We first investigated whether SLX4IP is required for unperturbed DNA synthesis in U2OS (Alt-positive), RPE1-hTERT (ALT-negative), and eHAP (ALT-negative) cells (Fig. EV2C–E) by sequentially labeling replicating DNA with CldU for 15 min followed by IdU for 30 min, and subsequently measuring IdU track lengths. Loss of SLX4IP resulted in a significant reduction in IdU track length, indicating decreased replication fork speed compared to wild-type cells across all three cell types (Figs. 2A,B and  EV2F). This effect was only mildly exacerbated by the addition of a low dose of the replication stress-inducing agent hydroxyurea (HU; 50 μM for 30 min) or by an acute 30-min treatment with 50 μM cisplatin, which has also been shown to induce mild replication stress (Fig. EV2G) (Mehta et al, 2022; Quinet et al, 2020). These data suggest that SLX4IP loss induces endogenous replication stress, such that replication forks are already operating under compromised conditions, thereby limiting the impact of additional stress.

Given the pronounced effects of SLX4IP loss on replication fork progression, we performed iPOND-MS (isolation of proteins on nascent DNA-mass spectrometry) analyses in multiple clones of SLX4IP-deficient U2OS, RPE1-hTert, and eHAP cells to determine whether its absence alters replication fork composition (Figs. 2C–F and  EV2H–L) (Sirbu et al, 2013). Consistent with the observed decrease in replication fork speed, iPOND-MS revealed increased association of replisome components (such as MCM proteins, RFC proteins, and polymerases), mismatch repair factors as well as chromatin assembly factors (such as RBBP4, CHAF1B, SSRP1, and NAP1L1/4) with elongating DNA in U2OS and RPE1-hTert cells (Figs. 2C–F and Fig. EV2H,I), suggesting prolonged residence of active forks and delayed chromatin maturation around newly replicated chromatin. In addition, we observed an increase in heterochromatin assembly factors (histone H1 variants, DNMT1, HDAC1) alongside H2AX, which is consistent with fork-protective heterochromatin assembly at slowed forks (Feng et al, 2019; Gaggioli et al, 2023; Ozgencil et al, 2023).

SLX4IP-deficient eHAP cells likewise displayed strong replication stress signatures (Fig. EV2J–L). However, while chromatin remodelers were also enriched in this cell type, we observed a broader representation of replication stress response markers such as macroH2A, Timeless, Tipin, SLNF11, 53BP1, HLTF being recruited to SLX4IP-deficient replicating DNA. Notably, MCMs and particularly histones were depleted from forks in these cells, indicating MCM helicase uncoupling in these cell line clones.

Collectively, these data indicate that SLX4IP is a regulator of DNA replication whose loss triggers replication stress and alters replication fork composition in both ALT-positive and ALT-negative cells, suggesting a general role in maintaining replication fork integrity and genome stability.

### SLX4IP deficiency leads to the accumulation of single-stranded DNA gaps

**Figure 3:**
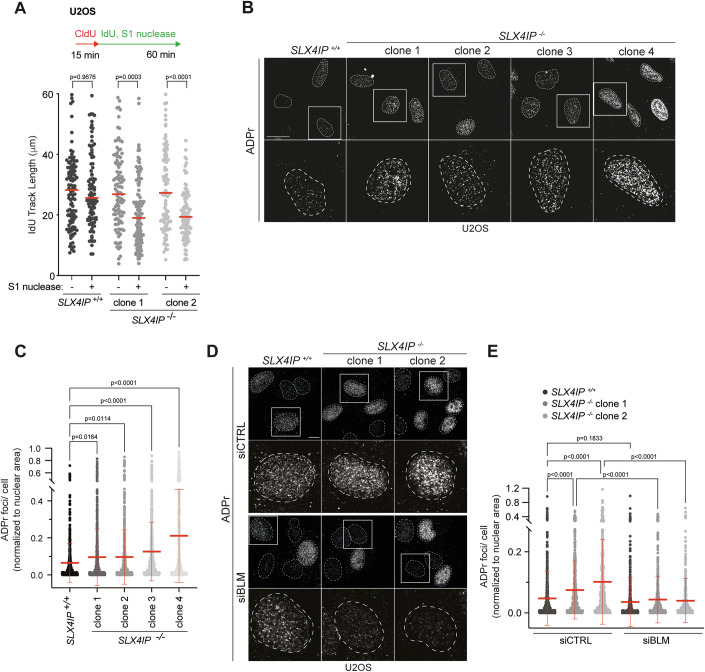
SLX4IP deficiency leads to the accumulation of single-stranded DNA gaps. (**A**) U2OS cells were labeled with CldU and IdU before DNA combing and in-gel S1 nuclease treatment. Individual IdU fiber lengths are plotted. Median is indicated; *n* = 3, biological replicates; ANOVA with Dunn’s multiple comparisons post-test. Exact *P* values are shown in the figure; *P* < 0.05 was considered statistically significant. (**B**) U2OS cells were treated with 10 µM PARG inhibitor for 30 min, fixed, and processed for ADPr immunofluorescence. The dotted line represents DAPI (not shown). Lower panels are 2.5X magnifications of the indicated fields. Scale bar represents 20 μm. (**C**) Quantification of (**B**). The number of ADPr foci in each cell was quantified and normalized to nuclear area to account for variations in nuclear size across cells. At least 100 cells per condition and experiment were counted. Data are represented as mean ± SD; *n* = 3, biological replicates; one-way ANOVA with Dunnett’s multiple comparisons post-test. Exact *P* values are shown in the figure; *P* < 0.05 was considered statistically significant. (**D**) U2OS cells were transfected either with non-targeting siRNA (siCTRL) or BLM targeting siRNA (siBLM). Cells were treated with 10 µM PARG inhibitor for 30 min, fixed, and processed for ADPr immunofluorescence. The dotted line represents DAPI (not shown). Lower panels in each condition are 2.5X magnifications of the indicated fields. Scale bar represents 10 μm. (**E**) Quantification of (**D**). The number of ADPr foci in each cell was quantified and normalized to nuclear area to account for variations in nuclear size across cells. At least 100 cells per condition and experiment were counted. Data are represented as mean ± SD; *n* = 3, biological replicates; one-way ANOVA Sidaks’s multiple comparisons post-test. Exact *P* values are shown in the figure; *P* < 0.05 was considered statistically significant. Knockdown validation is shown in Fig. EV3H. Source data are available online for this figure.

Given its role in maintaining replication fork integrity, we asked whether SLX4IP loss leads to structural defects at replicating DNA. Therefore, to assess the presence of single-stranded (ss) DNA gaps, which can arise from defects in DNA replication such as incomplete lagging-strand maturation, we performed DNA fiber combing combined with S1 nuclease treatment. S1 nuclease selectively cleaves single-stranded regions, shortening DNA fibers that contain replication-associated gaps. Compared to untreated controls, S1-treated samples exhibited a significant reduction in replication track length, which was further decreased in SLX4IP-deficient U2OS cells (Fig. 3A). This increased susceptibility to S1 digestion suggests a higher burden of single-stranded gaps in the absence of SLX4IP, consistent with replication defects that give rise to ssDNA gaps, such as impaired lagging-strand processing or incomplete gap filling.

**Figure EV3:**
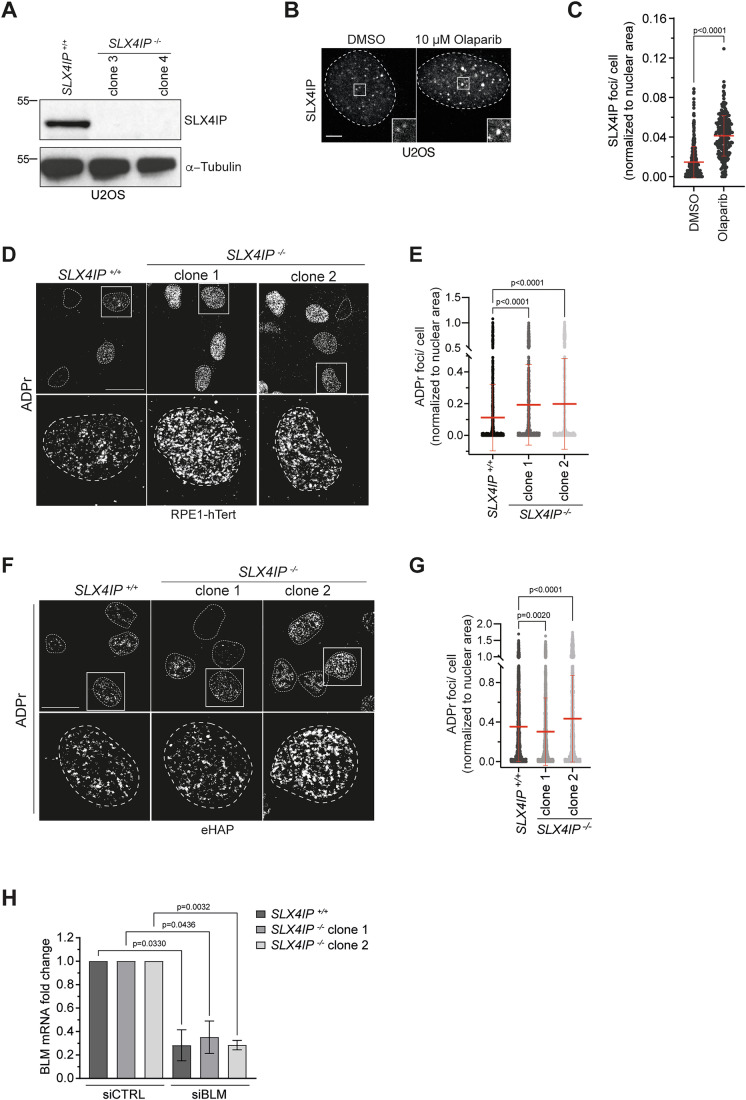
SLX4IP defi ciency increases ADPr levels on chromatin. (**A**) Whole-cell lysates of U2OS cells were separated by SDS-PAGE and analyzed for SLX4IP levels by immunoblotting. α-Tubulin was used as a loading control. The numbers on the left denote the molecular weight in kDa. (**B**) U2OS cells were either treated with 10 µM Olaparib or DMSO as a control for 48 h. Cells were then immediately pre-extracted, fixed, and processed for SLX4IP immunofluorescence. Insets are 2X magnifications of the indicated fields. The dotted line represents DAPI (not shown). Scale bar represents 5 μm. (**C**) Quantification of (**B**). The number of SLX4IP foci in each cell was quantified and normalized to nuclear area to account for variations in nuclear size across cells. At least 80 cells per condition and experiment were counted. Data are represented as mean ± SD; *n* = 3, biological replicates; Student’s *t* test. The exact *P* value is shown in the figure; *P* < 0.05 was considered statistically significant. (**D**) RPE1-hTert cells were treated with 10 µM PARG inhibitor for 30 min, immediately fixed, and processed for ADPr immunofluorescence. The dotted line represents DAPI (not shown). Lower panels are 3.5X magnifications of the indicated fields. Scale bar represents 25 μm. (**E**) Quantification of (**D**). The number of ADPr foci in each cell was quantified and normalized to nuclear area to account for variations in nuclear size across cells. At least 100 cells per condition and experiment were counted. Data are represented as mean ± SD; *n* = 3, biological replicates; one-way ANOVA with Sidak’s multiple comparisons post-test. Exact *P* values are shown in the figure; *P* < 0.05 was considered statistically significant. (**F**) eHAP cells were treated with 10 µM PARG inhibitor for 30 min, immediately fixed, and processed for ADP ribose immunofluorescence. The dotted line represents DAPI (not shown). Lower panels 3X magnifications of the indicated fields. Scale bar represents 15 μm. (**G**) Quantification of (**F**). The number of ADPr foci in each cell was quantified and normalized to nuclear area to account for variations in nuclear size across cells. At least 450 cells per condition and experiment were counted. Data are represented as mean ± SD; *n* = 3, biological replicates; one-way ANOVA with Sidak’s multiple comparisons post-test. Exact *P* values are shown in the figure; *P* < 0.05 was considered statistically significant. (**H**) RNA was isolated from cell pellets of each condition, reverse transcribed into cDNA, followed by RT-qPCR. Data were normalized to the siCTRL-treated samples. Data are represented as mean ± SD; *n* = 3, biological replicates; one-way ANOVA with Tukey’s multiple comparisons post-test. Exact *P* values are shown in the figure; *P* < 0.05 was considered statistically significant. Relates to Fig. 3D,E.

Next, we measured nuclear ADP-ribose (ADPr) levels, which predominantly mark ssDNA gaps in unstressed, replicating cells (Hanzlikova et al, 2018; Vaitsiankova et al, 2022). SLX4IP-deficient U2OS cells exhibited a significant increase in chromatin-associated ADPr compared to wild-type controls (Figs. 3B,C,  EV2C, and  EV3A), consistent with elevated levels of replication stress. In line with the ADPr increase, SLX4IP was more strongly associated with chromatin after treatment with 10 μM Olaparib (Fig. EV3B,C), indicating that SLX4IP responds to stressed or gap-containing forks that cannot activate ADPr signaling. Analysis in additional cell lines revealed that RPE1-hTert cells similarly show increased ADPr upon SLX4IP loss (Fig. EV3D,E), whereas eHAP cells did not display elevated ADPr under the same conditions (Fig. EV3F,G), highlighting a cell-type-specific response that mirrors the fork composition differences observed by iPOND-MS.

Finally, given the established role of BLM in maintaining fork integrity and our previous finding implicating SLX4IP as a negative regulator of BLM at ALT telomeres, we asked whether the replication stress phenotype observed in SLX4IP-deficient cells is dependent on BLM activity (Panier et al, 2019). Co-depletion of BLM in SLX4IP-deficient U2OS cells rescued the elevated ADPr signal back to wild-type levels (Figs. 3D,E and  EV3H), indicating that the increased replication stress in these cells arises from BLM-dependent processing of replication fork intermediates.

Overall, our results establish that SLX4IP is critical for maintaining replication fork integrity by suppressing ssDNA gaps and BLM-dependent replication stress.

### ALT telomeres are particularly vulnerable to SLX4IP-induced replication stress

Our findings indicate that SLX4IP maintains replication fork integrity across the genome. Given the pronounced enrichment of SLX4IP at ALT telomeres (Fig. EV1A and Panier et al, 2019) and the intrinsic replication challenges associated with these regions, we asked whether replicating ALT telomeres exhibit heightened sensitivity to SLX4IP loss.

**Figure 4:**
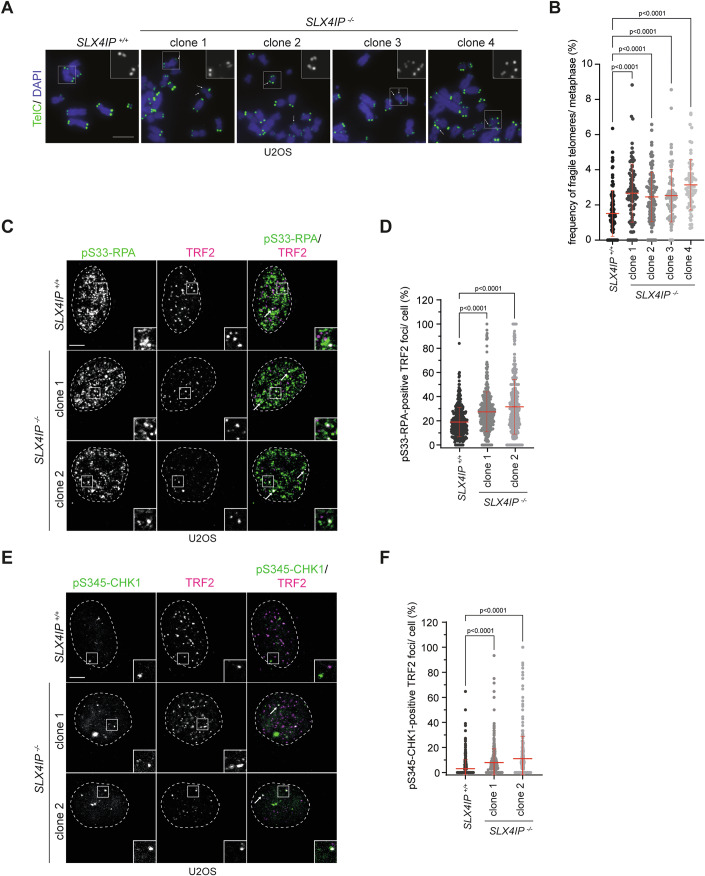
ALT telomeres are particularly vulnerable to SLX4IP-induced replication stress. (**A**) U2OS cells were fixed, and metaphases were processed for telomere PNA (TelC) FISH and DAPI. Insets are 1.5X magnifications of the indicated fields. Scale bar represents 100 μm. Arrows indicate fragile telomeres. (**B**) Quantification of (**A**). The number of fragile telomeres counted in each metaphase was normalized to the metaphase size. Data are represented as mean ± SD; *n* = 3 with at least 25 metaphases per experiment, biological replicates; one-way ANOVA with Dunnett’s multiple comparisons post-test. Exact *P* values are shown in the figure; *P* < 0.05 was considered statistically significant. (**C**) U2OS cells were pre-extracted, fixed, and processed for pSer33-RPA and TRF2 immunofluorescence. Insets are 2X magnifications of the indicated fields. The dotted line represents DAPI (not shown). Scale bar represents 5 μm. (**D**) Quantification of (**C**). The percentage of TRF2 foci that overlap with pSer33-RPA foci in each cell was quantified. At least 100 cells per condition and experiment were counted. Data are represented as mean ± SD; *n* = 3, biological replicates; one-way ANOVA with Dunnett’s multiple comparisons post-test. Exact *P* values are shown in the figure; *P* < 0.05 was considered statistically significant. (**E**) U2OS cells were pre-extracted, fixed, and processed for pSer345-CHK1 and TRF2 immunofluorescence. Insets are 2X magnifications of the indicated fields. The dotted line represents DAPI (not shown). Scale bar represents 5 μm. (**F**) Quantification of (**E**). The percentage of TRF2 foci that overlap with pSer345-CHK1 foci in each cell was quantified. At least 100 cells per condition and experiment were counted. Data are represented as mean ± SD; *n *= 3, biological replicates; one-way ANOVA with Dunnett’s multiple comparisons post-test. Exact *P* values are shown in the figure; *P *< 0.05 was considered statistically significant. Source data are available online for this figure.

**Figure EV4:**
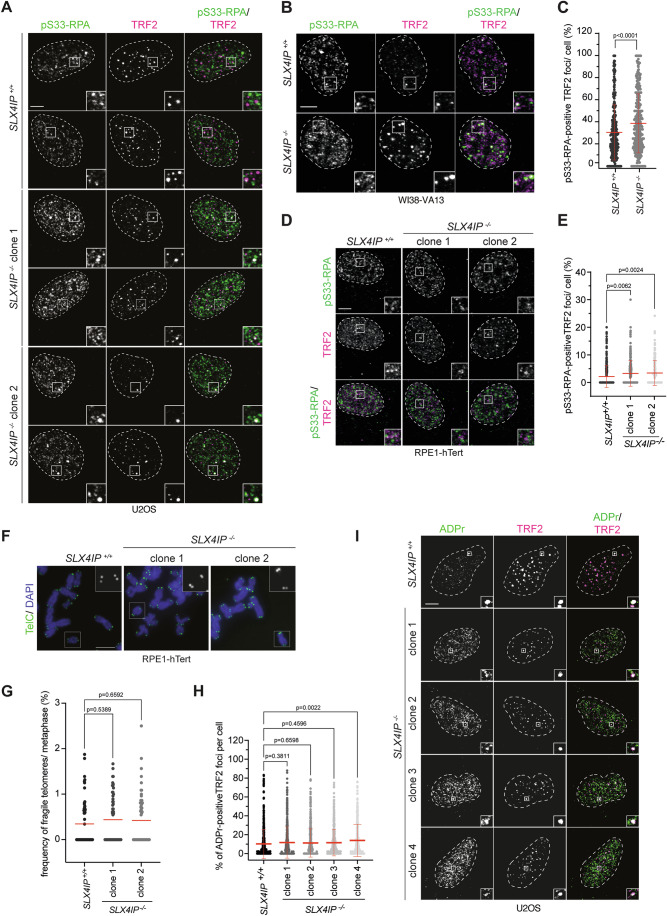
SLX4IP defi ciency causes replication stress at ALT telomeres. (**A**) U2OS cells were pre-extracted, fixed, and processed for pSer33-RPA and TRF2 immunofluorescence. Insets are 2X magnifications of the indicated fields. The dotted line represents DAPI (not shown). Scale bar represents 5 μm. Additional representative images related to Fig. 4C,D. (**B**) WI38-VA13 cells were pre-extracted, fixed, and processed for pSer33-RPA and TRF2 immunofluorescence. Insets are 2X magnifications of the indicated fields. The dotted line represents DAPI (not shown). Scale bar represents 5 μm. (**C**) Quantification of (**B**). The percentage of TRF2 foci that overlap with pSer33-RPA foci in each cell was quantified. At least 85 cells per condition and experiment were counted. Data are represented as mean ± SD; *n* = 3, biological replicates; Student’s *t* test. The exact *P* value is shown in the figure; *P* < 0.05 was considered statistically significant. (**D**) RPE1-hTert cells were pre-extracted, fixed, and processed for pSer33-RPA and TRF2 immunofluorescence. Insets are 2X magnifications of the indicated fields. The dotted line represents DAPI (not shown). Scale bar represents 5 μm. (**E**) Quantification of (**F**). The percentage of TRF2 foci that overlap with pSer33-RPA foci in each cell was quantified. At least 85 cells per condition and experiment were counted. Data are represented as mean ± SD; *n* = 3, biological replicates; one-way ANOVA with Sidak’s multiple comparisons post-test. Exact *P* values are shown in the figure; *P* < 0.05 was considered statistically significant. (**F**) RPE1-hTert cells were fixed, and metaphases were processed for telomere PNA (TelC) FISH and DAPI. Insets are ×3 magnifications of the indicated fields. Scale bar represents 100  μm. Arrows indicate fragile telomeres. (**G**) Quantification of (**F**). The number of fragile telomeres counted in each metaphase was normalized to the metaphase size. Data are represented as mean; *n* = 2 with at least 30 metaphases per experiment, biological replicates; one-way ANOVA with Dunnett’s multiple comparisons post-test. Exact *P* values are shown in the figure; *P* < 0.05 was considered statistically significant. (**H**) U2OS cells were treated with 10 µM PARG inhibitor for 30 min, fixed, and processed for ADPr and TRF2 immunofluorescence. The number of ADPr foci in each cell was quantified and normalized to nuclear area to account for variations in nuclear size across cells. At least 100 cells per condition and experiment were counted. Data are represented as mean ± SD; *n* = 3, biological replicates; one-way ANOVA with Dunnett’s multiple comparisons post-test. Exact *P* values are shown in the figure; *P* < 0.05 was considered statistically significant. (**I**) Representative images of (**H**). The dotted line represents DAPI (not shown). Insets are 3X magnifications of the indicated fields. Scale bar represents 5 μm.

To test this, we examined telomere fragility and ATR-dependent replication stress signaling in ALT-positive and ALT-negative cells. SLX4IP deficiency resulted in a marked increase in telomere fragility in U2OS cells (Fig. 4A,B). Consistently, we observed increased accumulation of ATR-dependent replication stress markers at telomeres, including pSer33-RPA in both U2OS and WI38-VA13 cells (Figs. 4C,D and  EV4A–C), as well as pS345-CHK1 in U2OS cells (Fig. 4E,F). In contrast, ALT-negative RPE1-hTert cells displayed only a minor increase in telomeric Ser33-RPA phosphorylation (Fig. EV4D,E) and no significant change in telomere fragility (Fig. EV4F,G), suggesting that non-ALT cells more effectively tolerate or compensate for SLX4IP loss at telomeres.

**Figure EV5:**
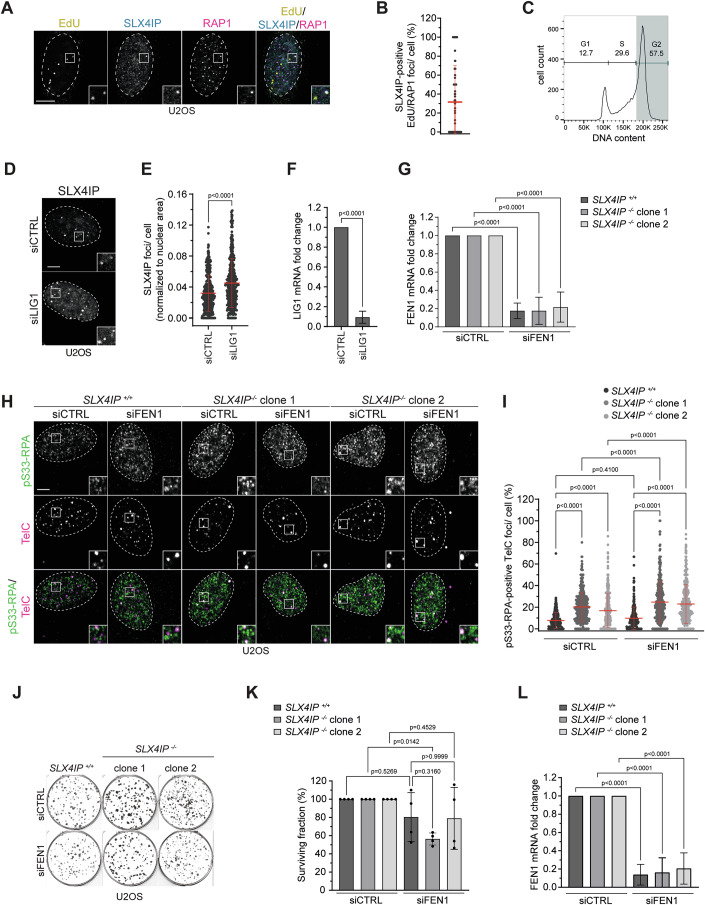
SLX4IP potentially regulates lagging-strand replication stress at ALT telomeres. (**A**) U2OS cells were subjected to a double thymidine block to enrich for G2 cells. An EdU pulse was performed 2 h before fixation, followed by staining of EdU via a Click-IT reaction. SLX4IP and RAP1 were stained by indirect immunofluorescence. Scale bar represents 10 μm. Insets are 2X magnifications of the indicated fields. The dotted line represents DAPI (not shown). Scale bar represents 5 μm. (**B**) Quantification of (**A**). The percentage of SLX4IP foci that overlap with EdU-positive RAP1 foci in G2 cells was quantified and normalized to nuclear area to account for variations in nuclear size across cells. At least 60 cells per experiment were counted. Data are represented as mean ± SD; *n* = 3, biological replicates. (**C**) FACS analysis. U2OS cells subjected to a double thymidine block and released to G2-phase were fixed, stained with DAPI, and analyzed by flow cytometry. The gating was performed based on the cell cycle distribution of asynchronous cells. Related to (**A**). (**D**) U2OS cells were transfected either with non-targeting siRNA (siCTRL) or knocked down for Ligase1 (siLIG1), pre-extracted, fixed, and processed for SLX4IP immunofluorescence. Insets are 2X magnifications of the indicated fields. The dotted line represents DAPI (not shown). Scale bar represents 5 μm. (**E**) Quantification of (**D**). The number of SLX4IP foci in each cell was quantified and normalized to nuclear area to account for variations in nuclear size across cells. At least 100 cells per condition and experiment were counted. Data are represented as mean ± SD; *n* = 3, biological replicates; Student’s *t* test. The exact *P* value is shown in the figure; *P *< 0.05 was considered statistically significant. (**F**) RNA was isolated from cell pellets of each condition, reverse transcribed into cDNA, followed by RT-qPCR. Data were normalized to the siCTRL-treated samples; *n* = 3, biological replicates; Student’s *t* test. The exact *P* value is shown in the figure; *P* < 0.05 was considered statistically significant. Related to (**D**, **E**). (**G**) RNA was isolated from cell pellets of each condition, reverse transcribed into cDNA, followed by RT-qPCR. Data were normalized to the siCTRL-treated samples. Data are represented as mean ± SD; *n* = 3, biological replicates; one-way ANOVA with Tukey’s multiple comparisons post-test. Exact *P* values are shown in the figure; *P* < 0.05 was considered statistically significant. Related to (**H**, **I**). (**H**) U2OS cells were transfected either with non-targeting siRNA (siCTRL) or FEN1 targeting siRNA (siFEN1), pre-extracted, fixed, and processed for pSer33-RPA immunofluorescence followed by telomeric PNA (TelC) FISH. Insets are 2X magnifications of the indicated fields. The dotted line represents DAPI (not shown). Scale bar represents 5 μm. (**I**) Quantification of (**H**). The percentage of TelC foci that overlap with pSer33-RPA foci in each cell was quantified. At least 100 cells per condition and experiment were counted. Data are represented as mean ± SD; *n* = 3, biological replicates; one-way ANOVA with Sidak’s multiple comparisons post-test. Exact *P* values are shown in the figure; *P* < 0.05 was considered statistically significant. (**J**) U2OS cells were transfected either with non-targeting siRNA (siCTRL) or FEN1 targeting siRNA (siFEN1) and seeded in a clonogenic survival assay. (**K**) Quantification of (**J**). Data are represented as mean ± SD; *n* = 3, biological replicates; one-way ANOVA with Sidak’s multiple comparisons post-test. Exact *P* values are shown in the figure; *P* < 0.05 was considered statistically significant. (**L**) RNA was isolated from cell pellets of each condition, reverse transcribed into cDNA, followed by RT-qPCR. Data were normalized to the siCTRL-treated samples. Data are represented as mean ± SD; *n* = 3, biological replicates; one-way ANOVA with Tukey’s multiple comparisons post-test. Exact *P* values are shown in the figure; *P* < 0.05 was considered statistically significant. Related to (**J**, **K**).

Consistent with a replication-associated role, SLX4IP localizes to 30% of EdU-positive telomere foci in G2 phase (Fig. EV5A–C), indicating association with replicating telomeres. Although we did not detect a telomere-specific increase in ADPr (Fig. EV4H,I), this may be due to the limited fraction of total DNA represented by ALT telomeres, and does not preclude the existence of ssDNA gaps at these sites. Indeed, we noticed that the lagging-strand replication factors FEN1 and Ligase 1 were enriched at replication forks specifically in SLX4IP-deficient U2OS cells (Fig. 2C,D), suggesting that lagging-strand replication may be especially sensitive to SLX4IP loss in ALT-positive cells. Based on this observation, we asked whether SLX4IP functionally and genetically interacts with these factors at ALT telomeres. Notably, SLX4IP accumulation at telomeres increased upon Ligase 1 depletion (Fig. EV5D–F), which is consistent with lagging-strand stress triggering its recruitment to telomeric chromatin. Moreover, co-depletion of FEN1 in SLX4IP-deficient cells did not further increase pSer33-RPA at telomeres (Fig. EV5G–I) nor did it result in a synthetic growth defect (Fig. EV5J–L), suggesting that these factors act in overlapping pathways rather than independently at telomeres.

**Figure EV6:**
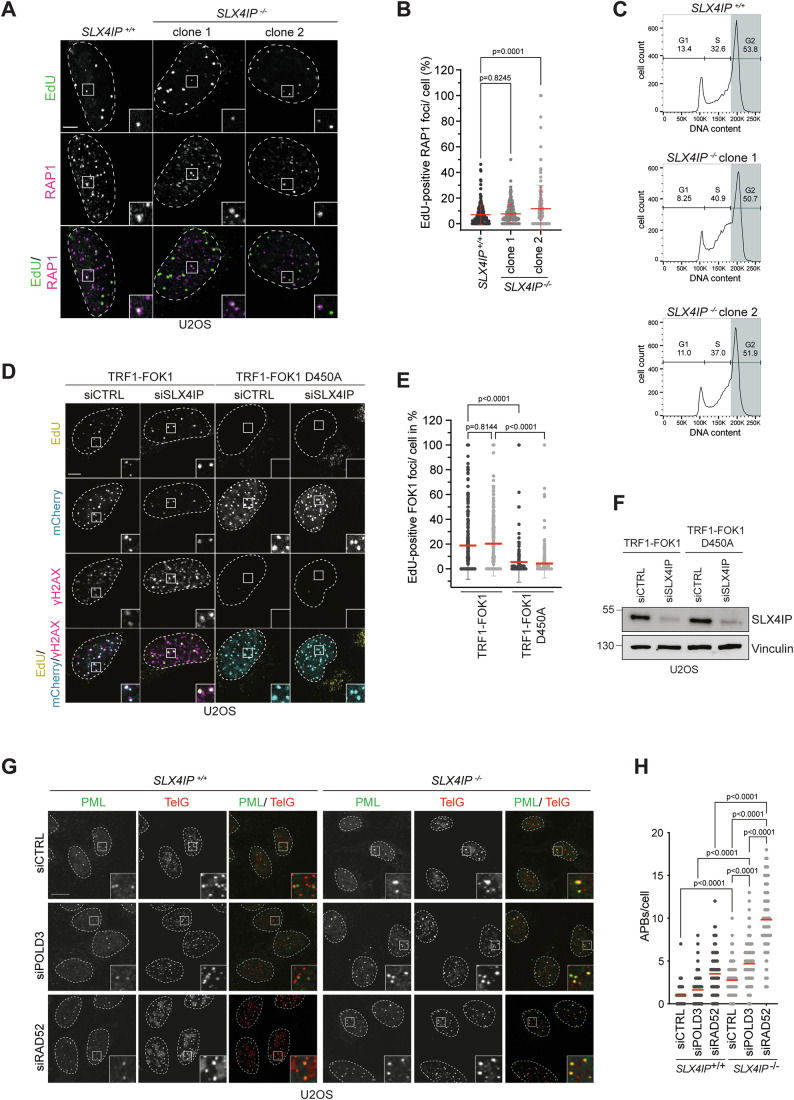
SLX4IP is not involved in break-induced replication. (**A**) U2OS cells were subjected to a double thymidine block to enrich for G2 cells. An EdU pulse was performed 2 h before fixation, followed by staining of EdU via a Click-IT reaction and staining of RAP1 by indirect immunofluorescence. The dotted line represents DAPI (not shown). Insets are 2X magnifications of the indicated fields. Scale bar represents 5 μm. (**B**) Quantification of (**A**). The percentage of RAP1 foci that overlap with EdU foci in each G2 cell was quantified. At least 50 cells per condition and experiment were counted. Data are represented as mean ± SD; *n* = 3, biological replicates; one-way ANOVA with Sidak’s multiple comparisons post-test. Exact *P* values are shown in the figure; *P* < 0.05 was considered statistically significant. (**C**) FACS analysis. U2OS cells subjected to a double thymidine block and released to G2-phase were fixed, stained with DAPI, and analyzed by flow cytometry. The gating was performed based on the cell cycle distribution of asynchronous cells. Related to (**A**, **B**). (**D**) U2OS cells expressing mCherry-labeled TRF1-FOK1 or TRF1-FOK1 D450A were transfected either with non-targeting siRNA (siCTRL) or SLX4IP targeting siRNA (siSLX4IP). They were subjected to a 4 h EdU pulse before fixation, followed by staining of EdU via a Click-IT reaction and staining of γH2AX by indirect immunofluorescence. The dotted line represents DAPI (not shown). Insets are 2X magnifications of the indicated fields. Scale bar represents 5 μm. (**E**) Quantification of (**D**). The percentage of mCherry foci that overlap with EdU foci in each cell was quantified. At least 40 cells per condition and experiment were counted. Data are represented as mean ± SD; *n* = 3, biological replicates; one-way ANOVA with Sidak’s multiple comparisons post-test. Exact *P* values are shown in the figure; *P* < 0.05 was considered statistically significant. (**F**) Whole-cell lysates of U2OS cells were separated by SDS-PAGE and analyzed for SLX4IP levels by immunoblotting. Vinculin was used as a loading control. The numbers on the left denote the molecular weight in kDa. (**G**) U2OS cells were transfected either with non-targeting siRNA (siCTRL), POLD3 targeting siRNA (siPOLD3), or RAD52 targeting siRNA (siRAD52), fixed, and processed for PML immunofluorescence followed by telomeric PNA (TelG) FISH. Insets are 3X magnifications of the indicated fields. The dotted line represents DAPI (not shown). Scale bar represents 10 μm. (**H**) Quantification of (**G**). At least 100 cells per condition and experiment were counted. Mean is indicated; *n* = 2, biological replicates; one-way ANOVA with Tukey’s multiple comparisons post-test. Exact *P* values are shown in the figure; *P* < 0.05 was considered statistically significant.

Since replication stress ultimately triggers telomere extension via break-induced replication at ALT telomeres, we also asked whether SLX4IP is directly involved in telomeric DNA synthesis. However, SLX4IP loss did not elevate EdU incorporation, neither at telomeres in G2 phase of the cell cycle, when BIR takes place (Fig. EV6A–C), nor at TRF1-FOK1-induced telomeric breaks (Fig. EV6D–F). Furthermore, co-depletion of the BIR factors POLD3 and RAD52 in SLX4IP-deficient cells further increased APB formation (Fig. EV6G,H), indicating that SLX4IP acts independently of the canonical BIR machinery and arguing against a direct role in break-induced replication.

Collectively, these data indicate that ALT telomeres are particularly sensitive to SLX4IP loss, with SLX4IP acting to mitigate replication stress rather than directly promoting break-induced replication.

### SLX4IP acts in parallel to FANCM to limit replication stress at ALT telomeres

Our findings indicate that SLX4IP mitigates replication stress at ALT telomeres independently of BIR. Given that replication stress is a key driver of ALT activity, we asked whether additional DNA replication stress factors at ALT telomeres work with SLX4IP to maintain fork integrity. FANCM, an ATPase/translocase that limits replication stress at ALT telomeres (Pan et al, 2017; Lu et al, 2019; Silva et al, 2019; Pan et al, 2019), emerged as a strong candidate because its loss shares several striking similarities with SLX4IP depletion. Specifically, both SLX4IP and FANCM loss lead to elevated replication stress at ALT telomeres and a BLM-dependent hyper-ALT phenotype (Panier et al, 2019; Silva et al, 2019). In addition, both proteins interact with BLM, with SLX4IP binding directly and FANCM interacting indirectly through RMI1/2 in the context of the BTR complex, although the molecular consequences of these interactions for BLM remain unclear (Panier et al, 2019; Ling et al, 2016). These common observations raised the question of whether SLX4IP and FANCM act in the same pathway or in parallel to counteract replication stress at ALT telomeres.

**Figure EV7:**
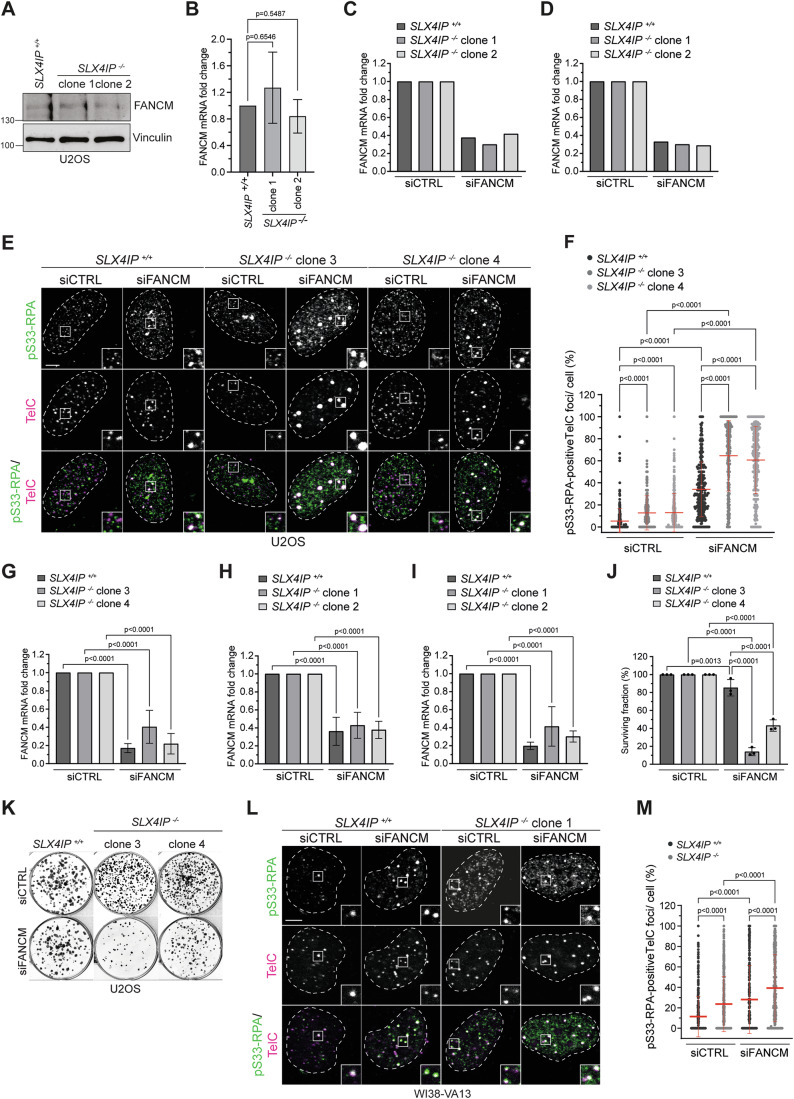
SLX4IP is synthetic lethal with FANCM in ALT-positive cells. (**A**) Whole-cell lysates of U2OS cells were separated by SDS-PAGE and analyzed for FANCM levels by immunoblotting. Vinculin was used as a loading control. The numbers on the left denote the molecular weight in kDa. (**B**) RNA was isolated from cell pellets of each condition, reverse transcribed into cDNA, followed by RT-qPCR. Data were normalized to the wild-type samples. Data are represented as mean ± SD; *n* = 3, biological replicates; one-way ANOVA with Dunnett’s multiple comparisons post-test. Exact *P* values are shown in the figure; *P *< 0.05 was considered statistically significant. (**C**) RNA was isolated from cell pellets of each condition, reverse transcribed into cDNA followed by RT-qPCR. Data were normalized to the siCTRL-treated samples; *n* = 1. Related to Fig. 5A,B. Panel represents a representative knockdown control experiment. (**D**) RNA was isolated from cell pellets of each condition, reverse transcribed into cDNA, followed by RT-qPCR. Data were normalized to the siCTRL-treated samples; *n* = 1. Related to Fig. 5C,D. Panel represents a representative knockdown control experiment. (**E**) U2OS cells were transfected either with non-targeting siRNA (siCTRL) or FANCM targeting siRNA (siFANCM), pre-extracted, fixed, and processed for pSer33-RPA immunofluorescence followed by telomeric PNA (TelC) FISH. Insets are 2X magnifications of the indicated fields. The dotted line represents DAPI (not shown). Scale bar represents 5 μm. (**F**) Quantification of (**E**). The percentage of TelC foci that overlap with pSer33-RPA foci in each cell was quantified. At least 85 cells per condition and experiment were counted. Data are represented as mean ± SD; *n* = 3, biological replicates; one-way ANOVA with Sidak’s multiple comparisons post-test. Exact *P* values are shown in the figure; *P* < 0.05 was considered statistically significant. (**G**) RNA was isolated from cell pellets of each condition, reverse transcribed into cDNA, followed by RT-qPCR. Data were normalized to the siCTRL-treated samples. Data are represented as mean ± SD; *n* = 5, biological replicates; one-way ANOVA with Tukey’s multiple comparisons post-test. Exact *P* values are shown in the figure; *P* < 0.05 was considered statistically significant. Related to (**E**, **F**, **J**, **K**). (**H**) RNA was isolated from cell pellets of each condition, reverse transcribed into cDNA followed by RT-qPCR. Data were normalized to the siCTRL-treated samples. Data are represented as mean ± SD; *n* = 4, biological replicates; one-way ANOVA with Tukey’s multiple comparisons post-test. Exact *P* values are shown in the figure; *P* < 0.05 was considered statistically significant. Related to Fig. 5E,F. (**I**) RNA was isolated from cell pellets of each condition, reverse transcribed into cDNA, followed by RT-qPCR. Data were normalized to the siCTRL-treated samples. Data are represented as mean ± SD; *n* = 4, biological replicates; one-way ANOVA with Tukey’s multiple comparisons post-test. Exact *P* values are shown in the figure; *P* < 0.05 was considered statistically significant. Related to Fig. 5G,H. (**J**) U2OS cells were transfected either with non-targeting siRNA (siCTRL) or FANCM targeting siRNA (siFANCM) and seeded in a clonogenic survival assay. Data are represented as mean ± SD; *n* = 3, biological replicates; one-way ANOVA with Tukey’s multiple comparisons post-test. Exact *P* values are shown in the figure; *P *< 0.05 was considered statistically significant. Knockdown validation is shown in (**G**). (**K**) Representative images of (**J**). (**L**) WI38-VA13 cells were transfected either with non-targeting siRNA (siCTRL) or FANCM targeting siRNA (siFANCM), pre-extracted, fixed, and processed for pSer33-RPA immunofluorescence followed by telomeric PNA (TelC) FISH. Insets are 2X magnifications of the indicated fields. The dotted line represents DAPI (not shown). Scale bar represents 5 μm. (**M**) Quantification of (**L**). The percentage of TelC foci that overlap with pSer33-RPA foci in each cell was quantified. At least 100 cells per condition and experiment were counted. Data are represented as mean ± SD; *n* = 3, biological replicates; one-way ANOVA with Sidak’s multiple comparisons post-test. Exact *P* values are shown in the figure; *P* < 0.05 was considered statistically significant. Knockdown validation is shown in Fig. EV8C.

To rule out the possibility that SLX4IP deficiency indirectly affects FANCM function by altering its expression, we measured FANCM protein and mRNA levels in SLX4IP-deficient cells. Neither FANCM protein abundance (Fig. EV7A) nor transcript levels (Fig. EV7B) were detectably changed by loss of SLX4IP, as assessed by immunoblotting and qPCR, indicating that SLX4IP does not regulate FANCM expression or stability.

**Figure 5:**
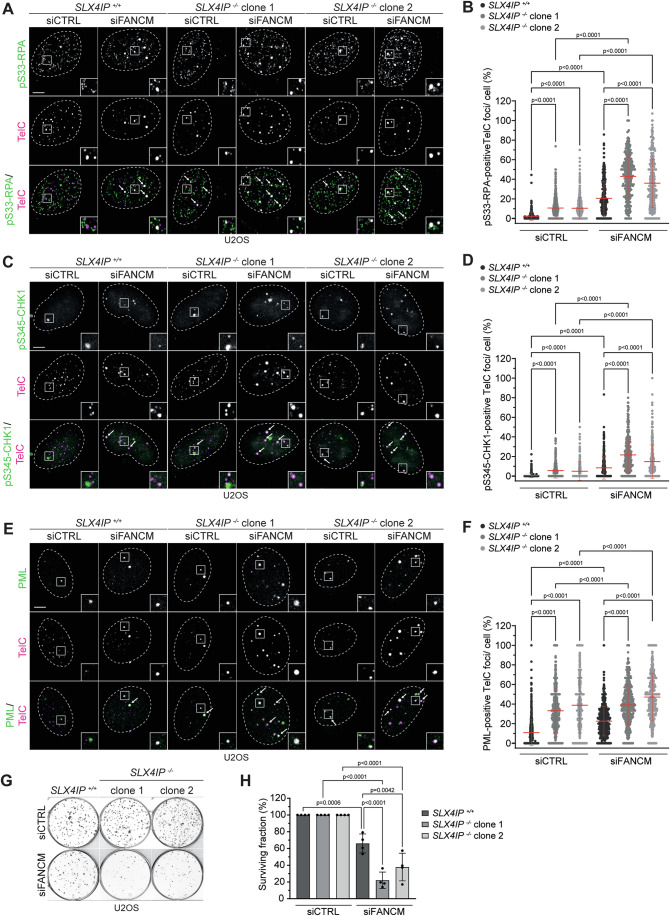
SLX4IP acts in parallel to FANCM to limit replication stress at ALT telomeres. (**A**) U2OS cells were transfected either with non-targeting siRNA (siCTRL) or FANCM targeting siRNA (siFANCM), pre-extracted, fixed, and processed for pSer33-RPA immunofluorescence followed by telomeric PNA (TelC) FISH. Insets are 2X magnifications of the indicated fields. The dotted line represents DAPI (not shown). Scale bar represents 5 μm. Arrows indicate pSer33-RPA/TelC-positive foci. (**B**) Quantification of (**A**). The percentage of TelC foci that overlap with pSer33-RPA foci in each cell was quantified. At least 100 cells per condition and experiment were counted. Data are represented as mean ± SD; *n* = 3, biological replicates; one-way ANOVA with Sidak’s multiple comparisons post-test. Knockdown validation is shown in Fig. EV7C. (**C**) U2OS cells were transfected either with non-targeting siRNA (siCTRL) or FANCM targeting siRNA (siFANCM), pre-extracted, fixed, and processed for pSer345-CHK1 immunofluorescence followed by telomeric PNA (TelC) FISH. Insets are 2X magnifications of the indicated fields. The dotted line represents DAPI (not shown). Scale bar represents 5 μm. Arrows indicate pSer345-CHK1/ TelC-positive foci. (**D**) Quantification of (**C**). The percentage of TelC foci that overlap with pSer345-CHK1 foci in each cell was quantified. At least 80 cells per condition and experiment were counted. Data are represented as mean ± SD; *n* = 3, biological replicates; one-way ANOVA with Sidak’s multiple comparisons post-test. Exact *P* values are shown in the figure; *P *< 0.05 was considered statistically significant. Knockdown validation is shown in Fig. EV7D. (**E**) U2OS cells were transfected either with non-targeting siRNA (siCTRL) or FANCM targeting siRNA (siFANCM), pre-extracted, fixed, and processed for PML immunofluorescence followed by telomeric PNA (TelC) FISH. Insets are 2X magnifications of the indicated fields. The dotted line represents DAPI (not shown). Scale bar represents 5 μm. Arrows indicate PML/TelC-positive foci. (**F**) Quantification of (**E**). APBs were quantified as the percentage of TelC foci that overlap with PML foci in each cell. At least 95 cells per condition and experiment were counted. Data are represented as mean ± SD; *n* = 5, biological replicates; one-way ANOVA with Sidak’s multiple comparisons post-test. Exact *P* values are shown in the figure; *P* < 0.05 was considered statistically significant. Knockdown validation is shown in Fig. EV7H. (**G**) U2OS cells were transfected either with non-targeting siRNA (siCTRL) or FANCM targeting siRNA (siFANCM) and seeded in a clonogenic survival assay. (**H**) Quantification of (**G**). Data are represented as mean ± SD; *n* = 4, biological replicates; one-way ANOVA with Tukey’s multiple comparisons post-test. Exact *P* values are shown in the figure; *P* < 0.05 was considered statistically significant. Knockdown validation is shown in Fig. EV7I. Source data are available online for this figure.

**Figure EV8:**
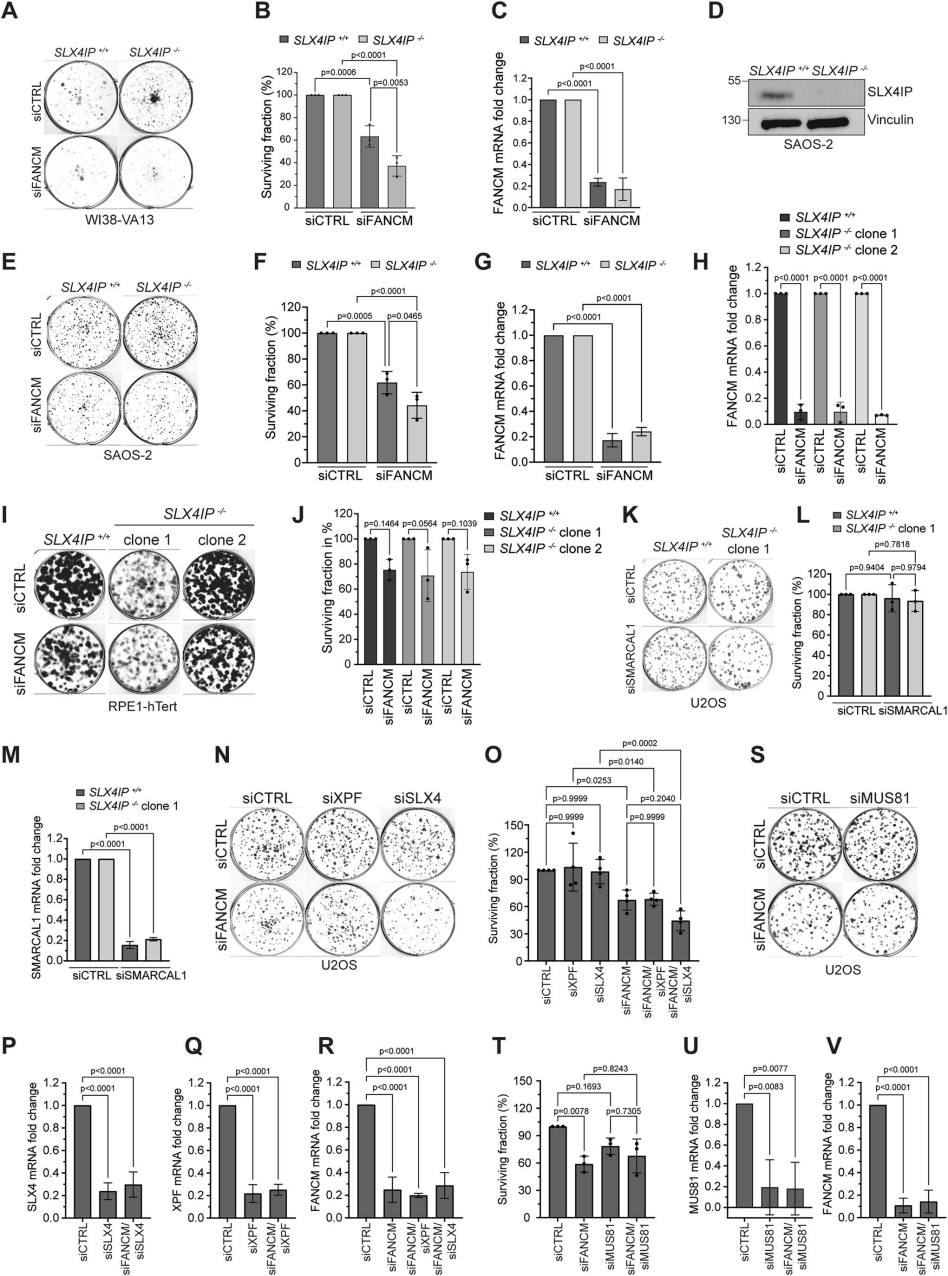
The synthetic lethal interaction between SLX4IP and FANCM is specifi c to ALT-positive cells. (**A**) WI38-VA13 cells were transfected either with non-targeting siRNA (siCTRL) or FANCM targeting siRNA (siFANCM) and seeded in a clonogenic survival assay. (**B**) Quantification of (**A**). Data are represented as mean ± SD; *n* = 3, biological replicates; one-way ANOVA; with Tukey’s multiple comparisons post-test. Exact *P* values are shown in the figure; *P* < 0.05 was considered statistically significant. (**C**) RNA was isolated from cell pellets of each condition, reverse transcribed into cDNA followed by RT-qPCR. Data were normalized to the siCTRL-treated samples. Data are represented as mean ± SD; *n* = 3, biological replicates; one-way ANOVA with Tukey’s multiple comparisons post-test. Exact *P* values are shown in the figure; *P* < 0.05 was considered statistically significant. Related to Fig. EV7L,M and **A**, **B**). (**D**) Whole-cell lysates of SAOS-2 cells were separated by SDS-PAGE and analyzed for SLX4IP levels by immunoblotting. Vinculin was used as a loading control. The numbers on the left denote the molecular weight in kDa. (**E**) SAOS-2 cells were transfected either with non-targeting siRNA (siCTRL) or FANCM targeting siRNA (siFANCM) and seeded in a clonogenic survival assay. (**F**) Quantification of €. Data are represented as mean ± SD; *n* = 3, biological replicates; one-way ANOVA with Tukey’s multiple comparisons post-test. Exact *P* values are shown in the figure; *P* < 0.05 was considered statistically significant. (**G**) RNA was isolated from cell pellets of each condition, reverse transcribed into cDNA, followed by RT-qPCR. Data were normalized to the siCTRL-treated samples. Data are represented as mean ± SD; *n* = 3, biological replicates; one-way ANOVA with Tukey’s multiple comparisons post-test. Exact *P* values are shown in the figure; *P* < 0.05 was considered statistically significant. Related to (**E**, **F**). (**H**) RNA was isolated from cell pellets of each condition, reverse transcribed into cDNA, followed by RT-qPCR. Data were normalized to the siCTRL-treated samples. Data are represented as mean ± SD; *n* = 3, biological replicates; one-way ANOVA with Tukey’s multiple comparisons post-test. Exact *P* values are shown in the figure; *P* < 0.05 was considered statistically significant. Related to (**I**, **J**). (**I**) RPE1-hTert cells were transfected either with non-targeting siRNA (siCTRL) or FANCM targeting siRNA (siFANCM) and seeded in a clonogenic survival assay. (**J**) Quantification of (**I**). Data are represented as mean ± SD; *n* = 3, biological replicates; one-way ANOVA with Tukey’s multiple comparisons post-test. Exact *P* values are shown in the figure; *P *< 0.05 was considered statistically significant. Knockdown validation is shown in (**H**). (**K**) U2OS cells were transfected either with non-targeting siRNA (siCTRL) or SMARCAL1 targeting siRNA (siSMARCAL1) and seeded in a clonogenic survival assay. (**L**) Quantification of (**K**). Data are represented as mean ± SD; *n* = 3, biological replicates; one-way ANOVA with Tukey’s multiple comparisons post-test. Exact *P* values are shown in the figure; *P* < 0.05 was considered statistically significant. (**M**) RNA was isolated from cell pellets of each condition, reverse transcribed into cDNA, followed by RT-qPCR. Data were normalized to the siCTRL-treated samples. Data are represented as mean ± SD; *n* = 3, biological replicates, one-way ANOVA with Tukey’s multiple comparisons post-test. Related to (**K**, **L**). (**N**) U2OS cells were transfected with the denoted combinations of non-targeting siRNA (siCTRL), siFANCM, siSLX4, and siXPF. Cells were then seeded in a clonogenic survival assay. (**O**) Quantification of (**N**). Data are represented as mean ± SD; *n* = 4, biological replicates; one-way ANOVA with Sidak’s multiple comparisons post-test. Exact *P* values are shown in the figure; *P* < 0.05 was considered statistically significant. (**P**) RNA was isolated from cell pellets of each condition, reverse transcribed into cDNA, followed by RT-qPCR. Data were normalized to the siCTRL-treated samples. Data are represented as mean ± SD; *n* = 4, biological replicates; one-way ANOVA with Tukey’s multiple comparisons post-test. Exact *P* values are shown in the figure; *P* < 0.05 was considered statistically significant. Related to (**N**, **O**). (**Q**) RNA was isolated from cell pellets of each condition, reverse transcribed into cDNA followed by RT-qPCR. Data were normalized to the siCTRL-treated samples. Data are represented as mean ± SD; *n* = 4, biological replicates; one-way ANOVA with Tukey’s multiple comparisons post-test. Exact *P* values are shown in the figure; *P* < 0.05 was considered statistically significant. Related to (**N**, **O**). (**R**) RNA was isolated from cell pellets of each condition, reverse transcribed into cDNA, followed by RT-qPCR. Data were normalized to the siCTRL-treated samples. Data are represented as mean ± SD; *n* = 4, biological replicates; one-way ANOVA with Tukey’s multiple comparisons post-test. Exact *P* values are shown in the figure; *P* < 0.05 was considered statistically significant. Related to (**N**, **O**). (**S**) U2OS cells were transfected with the denoted combinations of non-targeting siRNA (siCTRL), siFANCM, and siMUS81. Cells were then seeded in a clonogenic survival assay. (**T**) Quantification of (**S**). Data are represented as mean ± SD; *n *= 3, biological replicates; one-way ANOVA with Sidak’s multiple comparisons post-test. Exact *P* values are shown in the figure; *P* < 0.05 was considered statistically significant. (**U**) RNA was isolated from cell pellets of each condition, reverse transcribed into cDNA, followed by RT-qPCR. Data were normalized to the siCTRL-treated samples. Data are represented as mean ± SD; *n* = 3, biological replicates; one-way ANOVA with Tukey’s multiple comparisons post-test. Exact *P* values are shown in the figure; *P* < 0.05 was considered statistically significant. Related to (**S**, **T**). (**V**) RNA was isolated from cell pellets of each condition, reverse transcribed into cDNA, followed by RT-qPCR. Data were normalized to the siCTRL-treated samples. Data are represented as mean ± SD; *n* = 3, biological replicates; one-way ANOVA with Tukey’s multiple comparisons post-test. Exact *P* values are shown in the figure; *P* < 0.05 was considered statistically significant. Related to (**S**, **T**).

Having established that FANCM levels remain intact in SLX4IP-deficient cells, we next asked whether the two proteins functionally interact. Strikingly, simultaneous loss of SLX4IP and FANCM led to a significant augmentation of telomeric pSer33-RPA (Figs. 5A,B,  EV7C, and  EV7E–G) and pCHK1 (Figs. 5C,D and  EV7D) in U2OS cells. This was accompanied by a further increase in APB formation (Figs. 5E,F and  EV7H) and a pronounced synthetic growth defect (Figs. 5G,H,  EV7I, and  EV7G,J,K). Consistently, FANCM depletion in the context of SLX4IP deficiency led to elevated pSer33-RPA levels (Fig. EV7L,M) and a pronounced synthetic growth defect (Fig. EV8A–C) also in ALT-positive WI38-VA13 cells (Panier et al, 2019). To exclude the possibility that the observed synthetic lethal phenotype arose from clonal artifacts in the U2OS and WI38-VA13 knockout models, we further validated the genetic interaction between SLX4IP and FANCM in an independent, pooled ALT-positive SLX4IP knockout SAOS-2 cell line (Fig. EV8D). In this setting, FANCM and SLX4IP co-depletion resulted in a ~30% reduction in cell growth relative to FANCM depletion alone (Fig. EV8E–G). Although the magnitude of the growth defect varied across ALT cell lines, the synthetic lethal phenotypes were reproducible and directionally consistent. This variability aligns with the well-established heterogeneity of ALT phenotypes across distinct cellular backgrounds (Cesare et al, 2009; Henson et al, 2009; Loe et al, 2020). Of note, simultaneous loss of SLX4IP and FANCM did not lead to a synthetic growth defect in ALT-negative RPE1-hTert cells (Fig. EV8H–J), suggesting that the synthetic lethal relationship of SLX4IP and FANCM reflects an ALT-specific vulnerability.

To determine whether the synthetic growth defect observed with FANCM was specific to this factor, we also examined SMARCAL1, a related DNA translocase that acts in a pathway parallel to FANCM and that has also been implicated in ALT telomere maintenance (Feng et al, 2024; Cox et al, 2016). However, co-depletion of SLX4IP and SMARCAL1 did not result in a synthetic growth defect (Fig. EV8K–M), suggesting that SMARCAL1 and SLX4IP function epistatically at replicating ALT telomeres. In addition, we tested whether SLX4, a key interactor of SLX4IP, and its associated endonucleases, XPF and MUS81, exhibit synthetic lethality with FANCM in ALT-positive U2OS cells. Unlike SLX4IP, co-depletion of FANCM with either XPF (Fig. EV8N,O,Q,R) or MUS81 (Figs. EV8T,S and  EV8U,V) did not reduce clonogenic survival. Co-depletion with SLX4 produced a consistent but very modest decrease in clonal cell growth. However, this effect was not statistically significant (Figs. EV8N,O and  EV8P,R). These data suggest that the synthetic interaction with FANCM is specific to SLX4IP and is not shared by SLX4 or its associated endonucleases.

Together, our findings indicate that SLX4IP and FANCM function in distinct but complementary pathways to counteract replication stress at ALT telomeres.

### The synthetic lethal interaction between SLX4IP and FANCM is dependent on BLM

**Figure 6:**
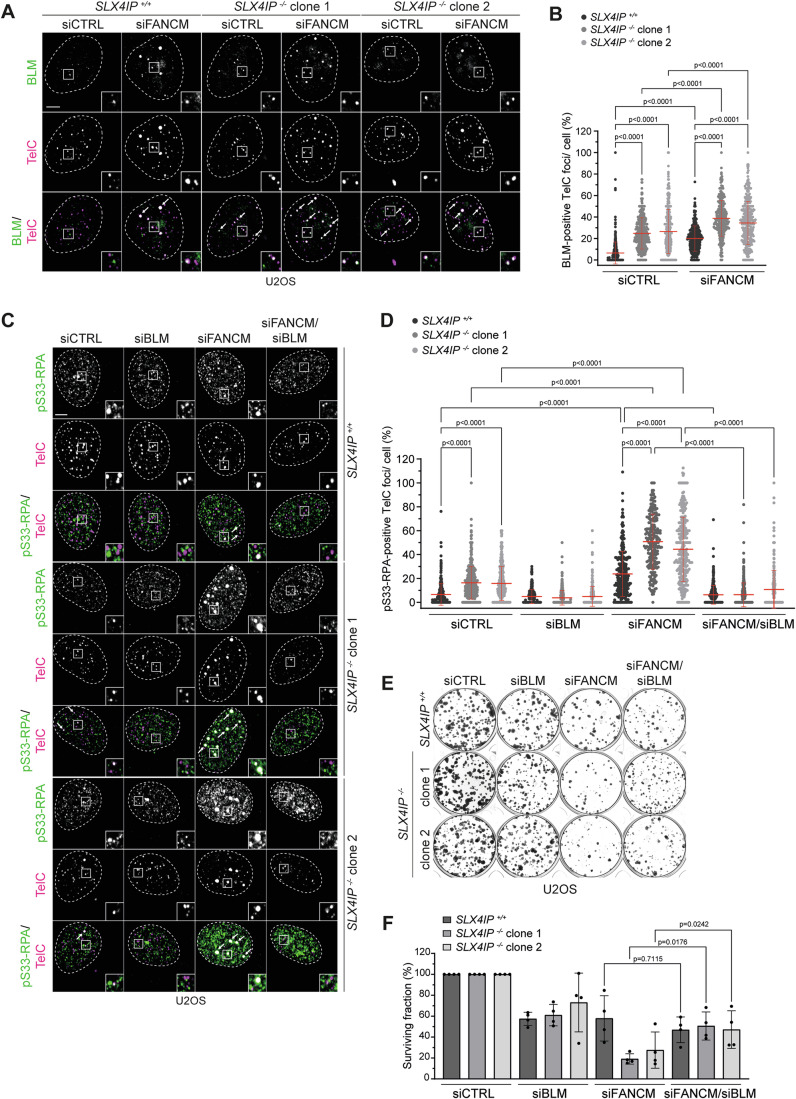
The synthetic lethal interaction between SLX4IP and FANCM is dependent on BLM. (**A**) U2OS cells were transfected either with non-targeting siRNA (siCTRL) or FANCM targeting siRNA (siFANCM), pre-extracted, fixed, and processed for BLM immunofluorescence followed by telomeric PNA (TelC) FISH. Insets are 2X magnifications of the indicated fields. The dotted line represents DAPI (not shown). Scale bar represents 5 μm. Arrows indicate BLM/ TelC-positive foci. (**B**) Quantification of (**A**). The percentage of TelC foci that overlap with BLM foci in each cell was quantified. At least 95 cells per condition and experiment were counted. Data are represented as mean ± SD; *n* = 3, biological replicates; one-way ANOVA with Tukey’s multiple comparisons post-test. Exact *P* values are shown in the figure; *P* < 0.05 was considered statistically significant. Knockdown validation is shown in Fig. EV9A. (**C**) U2OS cells were transfected either with non-targeting siRNA (siCTRL) or knocked down for FANCM (siFANCM), BLM (siBLM), or both (siFANCM/siBLM). Cells were pre-extracted, fixed, and processed for pSer33-RPA immunofluorescence followed by telomeric PNA (TelC) FISH. Insets are 2X magnifications of the indicated fields. The dotted line represents DAPI (not shown). Scale bar represents 5 μm. Arrows indicate pSer33-RPA/ TelC-positive foci. (**D**) Quantification of (**C**). The percentage of TelC foci that overlap with pSer33-RPA foci in each cell was quantified. At least 68 cells per condition and experiment were counted. Data are represented as mean ± SD; *n* = 3, biological replicates; one-way ANOVA with Sidak’s multiple comparisons post-test. Exact *P* values are shown in the figure; *P* < 0.05 was considered statistically significant. Knockdown validation is shown in Fig. EV9B,C. (**E**) U2OS cells were transfected either with non-targeting siRNA (siCTRL) or knocked down for FANCM (siFANCM), BLM (siBLM), or both (siFANCM/siBLM). Cells were then seeded in a clonogenic survival assay. (**F**) Quantification of (**E**). Data are represented as mean ± SD; *n* = 4, biological replicates; one-way ANOVA with Sidak’s multiple comparisons post-test. Exact *P* values are shown in the figure; *P* < 0.05 was considered statistically significant. Knockdown validation is shown in Fig. EV9D,E. Source data are available online for this figure.

**Figure EV9:**
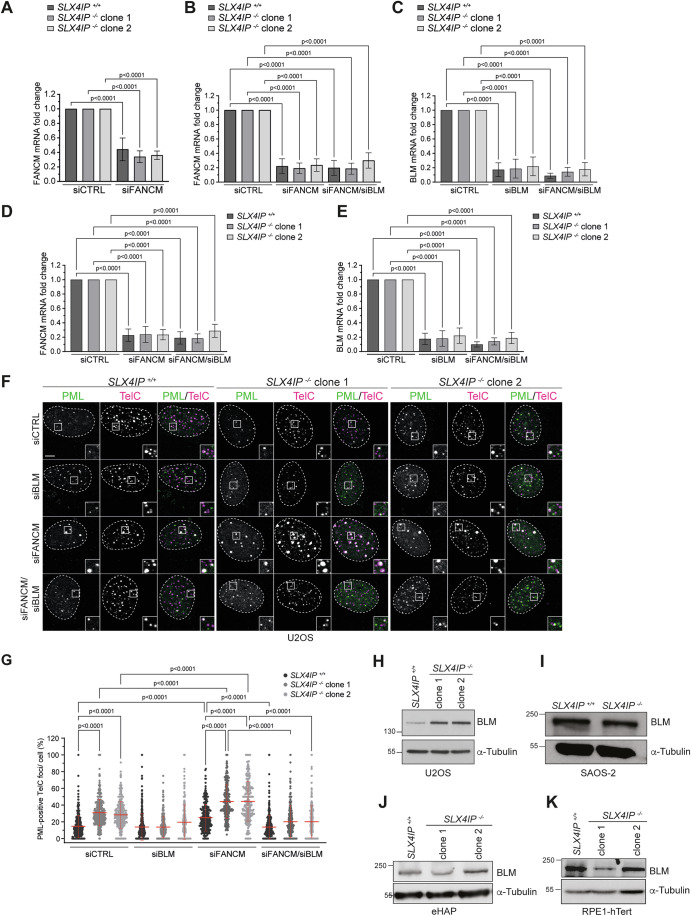
Co-depletion of BLM rescues SLX4IP/FANCM-dependent telomere phenotypes. (**A**) RNA was isolated from cell pellets of each condition, reverse transcribed into cDNA, followed by RT-qPCR. Data were normalized to the siCTRL-treated samples. Data are represented as mean ± SD; *n* = 3, biological replicates; one-way ANOVA with Tukey’s multiple comparisons post-test. Exact *P* values are shown in the figure; *P *< 0.05 was considered statistically significant. Related to Fig. 6A,B. (**B**) RNA was isolated from cell pellets of each condition, reverse transcribed into cDNA, followed by RT-qPCR. Data were normalized to the siCTRL-treated samples. Data are represented as mean ± SD; *n* = 3, biological replicates; one-way ANOVA with Tukey’s multiple comparisons post-test. Exact *P* values are shown in the figure; *P* < 0.05 was considered statistically significant. Related to Fig. 6C,D. (**C**) RNA was isolated from cell pellets of each condition, reverse transcribed into cDNA, followed by RT-qPCR. Data were normalized to the siCTRL-treated samples. Data are represented as mean ± SD; *n* = 3, biological replicates; one-way ANOVA with Tukey’s multiple comparisons post-test. Exact *P* values are shown in the figure; *P* < 0.05 was considered statistically significant. Related to Fig. 6C,D. (**D**) RNA was isolated from cell pellets of each condition, reverse transcribed into cDNA followed by RT-qPCR. Data were normalized to the siCTRL-treated samples. Data are represented as mean ± SD; *n* = 4, biological replicates; one-way ANOVA with Tukey’s multiple comparisons post-test. Exact *P* values are shown in the figure; *P* < 0.05 was considered statistically significant. Related to Fig. 6E,F. (**E**) RNA was isolated from cell pellets of each condition, reverse transcribed into cDNA, followed by RT-qPCR. Data were normalized to the siCTRL-treated samples. Data are represented as mean ± SD; *n* = 4, biological replicates; one-way ANOVA with Tukey’s multiple comparisons post-test. Exact *P* values are shown in the figure; *P* < 0.05 was considered statistically significant. Related to Fig. 6E,F. (**F**) U2OS cells were transfected either with non-targeting siRNA (siCTRL) or knocked down for FANCM (siFANCM), BLM (siBLM), or both (siFANCM/siBLM). Cells were pre-extracted, fixed, and processed for PML immunofluorescence followed by telomeric PNA (TelC) FISH. Insets are 2X magnifications of the indicated fields. The dotted line represents DAPI (not shown). Scale bar represents 5 μm. (**G**) Quantification of (**F**). APBs were quantified as the percentage of TelC foci that overlap with PML foci in each cell. At least 68 cells per condition and experiment were counted. Data are represented as mean ± SD; *n* = 3, biological replicates; one-way ANOVA with Sidak’s multiple comparisons post-test. Exact *P* values are shown in the figure; *P* < 0.05 was considered statistically significant. Knockdown validation is shown in (**B**, **C**). (**H**) Whole-cell lysates of U2OS cells were separated by SDS-PAGE and analyzed for BLM levels by immunoblotting. α-Tubulin was used as a loading control. The numbers on the left denote the molecular weight in kDa. (**I**) Whole-cell lysates of SAOS-2 cells were separated by SDS-PAGE and analyzed for BLM levels by immunoblotting. α-Tubulin was used as a loading control. The numbers on the left denote the molecular weight in kDa. (**J**) Whole-cell lysates of eHap cells were separated by SDS-PAGE and analyzed for BLM levels by immunoblotting. α-Tubulin was used as a loading control. The numbers on the left denote the molecular weight in kDa. (**K**) Whole-cell lysates of eHap cells were separated by SDS-PAGE and analyzed for BLM levels by immunoblotting. α-Tubulin was used as a loading control. The numbers on the left denote the molecular weight in kDa.

ALT-telomeric replication stress induced by FANCM is dependent on BLM activity (Silva et al, 2019). Given the non-epistatic genetic relationship of SLX4IP and FANCM, we hypothesized that the increased levels of replication stress observed in SLX4IP-deficient cells may also be mediated by BLM. To explore this possibility, we assessed BLM accumulation at ALT telomeres lacking SLX4IP. We observed an enhanced association of BLM with telomeres, a phenotype that was further amplified upon co-depletion of FANCM (Figs. 6A,B and  EV9A). Given this extensive telomeric accumulation of BLM, we next investigated whether the increased replication stress seen in SLX4IP-depleted cells, as well as the synthetic growth defect observed upon simultaneous depletion of SLX4IP and FANCM, might be dependent on BLM activity. To test this, we co-depleted BLM in SLX4IP-deficient cells as well as in SLX4IP/FANCM-co-depleted cells and assessed the impact on pSer33-RPA, cell viability, and APB formation. Strikingly, loss of BLM rescued the elevated pSer33-RPA signals observed at SLX4IP-deficient cells, indicating a restoration of normal replication dynamics at ALT telomeres (Figs. 6C,D and  EV9B,C). Furthermore, co-depletion of BLM rescued the synthetic growth defect observed upon simultaneous loss of SLX4IP and FANCM, demonstrating that the cytotoxicity that results from combined SLX4IP and FANCM deficiency is dependent on excessive BLM activity (Figs. 6E,F and  EV9D,E). In addition, the increase in APB formation observed in SLX4IP/FANCM co-depleted cells was significantly reduced upon BLM loss (Figs. EV9B,C and  EV9F,G), further supporting a model in which hyperactive BLM causes a hyper-ALT phenotype through excessive replicative stress in the absence of SLX4IP and FANCM.

Finally, although BLM levels are elevated in two independent SLX4IP knockout U2OS clones (Panier et al, 2019) (Fig. EV9H), BLM levels did not consistently change upon SLX4IP loss in pooled, ALT-positive SAOS-2 cells, or ALT-negative RPE1-hTert and eHAP cells (Fig. EV9I–K), suggesting that SLX4IP-dependent regulation of BLM is not simply a matter of gene expression or protein stability, but instead may reflect a more context-dependent functional mechanism.

Together, these findings indicate that SLX4IP depletion leads to a BLM-dependent hyper-replication stress phenotype at ALT telomeres. This suggests that SLX4IP functions as a critical regulator of BLM activity to maintain telomere stability and preserve cellular viability in ALT-positive cells, not only at the level of the telomere recombination intermediate (Panier et al, 2019), but also to prevent excessive replication stress.

## Discussion

In this study, we identify SLX4IP as a regulator of replication stress responses that preserves replication fork stability. Our findings show that SLX4IP localizes broadly across the genome, including but not limited to telomeres, and that this recruitment is enhanced under conditions of replication stress. Loss of SLX4IP leads to reduced fork speed, altered replisome composition, accumulation of post-replicative ssDNA gaps, and elevated nuclear ADPr, consistent with a role for SLX4IP in maintaining replication fork integrity and limiting replication-associated stress. Notably, SLX4IP is preferentially enriched proximal to replication origins, supporting a role in the stabilization of stalled forks near origins, the processing or repair of replication intermediates, or the regulation of origin-proximal replication dynamics.

Mechanistically, our findings describe a functional link between SLX4IP and BLM-dependent processing of replication forks. BLM performs multiple functions at replication forks, including fork reversal, unwinding of structured DNA, remodeling of stalled forks, and processing of fork recombination intermediates (Manthei and Keck, 2013; Sidorova et al, 2013). While these activities are essential for fork recovery and restart, excessive or untimely BLM action can destabilize replication intermediates, generate ssDNA, and promote pathological replication structures (Chen et al, 2024; Ellis et al, 2021; Ouyang et al, 2009, 2013; Sobinoff et al, 2017; Panier et al, 2019). Our observations that SLX4IP depletion causes accumulation of post-replicative ssDNA gaps and elevated nuclear ADPr are consistent with a model in which SLX4IP restrains aberrant BLM-dependent fork processing that would otherwise exacerbate replication stress. In long-pulse DNA combing experiments, measured fork progression decreases over time as termination events accumulate. Strikingly, under these extended labeling conditions in the S1 nuclease assay, replication tracts in SLX4IP-deficient cells eventually reach lengths comparable to wild-type, despite being S1-sensitive. This “catch-up” phenotype suggests that SLX4IP may influence replication termination dynamics as well as steady-state fork elongation. Future studies will directly test this possibility.

How SLX4IP negatively regulates BLM remains an important mechanistic question. Notably, our data indicate that SLX4IP does not regulate global BLM protein levels, suggesting that its effects arise from functional modulation rather than transcriptional or proteostatic control. Although SLX4IP directly interacts with BLM, it does not inhibit helicase activity in vitro, indicating that its regulatory effects are unlikely to reflect direct enzymatic suppression (Panier et al, 2019). Instead, SLX4IP most likely functions as a regulatory or scaffold factor for BLM function. One possibility is that SLX4IP influences context-dependent BLM recruitment, retention, or interaction and coordination with fork protection factors. Given the prominent role of post-translational modifications in controlling BLM function, an additional possibility is that SLX4IP influences modification-dependent regulatory pathways governing BLM activity (Böhm and Bernstein, 2014). Future studies will be required to more clearly define the molecular basis by which SLX4IP restrains BLM to prevent pathological fork remodeling and recombination.

Consistent with a primary role in replication stress regulation, iPOND-MS experiments in both U2OS and RPE1-hTERT cells reveal enrichment of chromatin remodelers, histones, replisome components, mismatch repair factors, and replication stress-associated proteins at elongating DNA following SLX4IP depletion. These signatures are consistent with longer dwell times of replisome and nucleosome assembly factors because of reduced fork speed (Nakamura et al, 2021; Sirbu et al, 2013). Notably, the enrichment of heterochromatin-associated factors and Histone H1 variants resembles chromatin remodeling pathways previously described at stressed forks and suggests that SLX4IP loss triggers fork-protective mechanisms (Feng et al, 2019; Kim et al, 2018; Gaggioli et al, 2023; Ozgencil et al, 2023).

Loss of BLM promotes the persistence of aberrant DNA structures that arise during replication of repetitive and structure-forming loci, and therefore requires substantial, but unknown regulation. BLM suppresses crossover formation through dissolution of double Holliday junctions, limiting aberrant recombination outcomes (Wu and Hickson, 2003). Beyond junction dissolution, BLM is required to maintain stability at structure-forming repetitive DNA, including AT-rich common fragile sites, where its loss increases replication-associated instability (Wang et al, 2018). In this setting, fork stalling at repetitive DNA can generate misaligned intermediates containing heteroduplex DNA or short insertion–deletions loops (IDLs). These mismatch-containing structures fall within the substrate specificity of the MSH6–MSH2 complex, which recognizes single-base mismatches and small insertion-deletion-loops (IDLs) arising during replication slippage and is enriched in the U2OS and RPE-hTert iPOND datasets (Kunkel and Erie, 2015; Gradia et al, 1997). Thus, the BLM/SLX4IP axis may primarily suppress the formation and persistence of structure-driven recombination intermediates at repetitive loci, whereas MSH6 may act downstream.

SLX4IP-deficient eHAP cells also exhibit strong replication stress signatures, with a proteomic composition suggestive of more frequent fork collapse events. This distinction likely reflects the presence of SLFN11 in eHAP cells, a factor known to promote RPA-dependent MCM unloading and potent checkpoint activation (Coleman et al, 2025; Stanage et al, 2026; Murai et al, 2018; Jo et al, 2021). In contrast, both U2OS and RPE1-hTERT cells lack SLFN11, a status generally associated with increased tolerance to replication stress through preservation of fork restart capacity and avoidance of irreversible fork blockade or degradation (Coleman et al, 2025; Stanage et al, 2026; Murai et al, 2018; Jo et al, 2021). As such, replication stress induced by SLX4IP loss appears to be less severe in these cell types as fork destabilization is less readily coupled to collapse.

Our CUT&Tag data show that SLX4IP is not restricted to telomeres but instead associates with diverse genomic loci, including fragile sites. This observation is consistent with prior studies linking SLX4IP to the stability of fragile genomic regions and supports a broader role for SLX4IP in safeguarding replication-challenged chromatin (Ingham et al, 2025; Pladevall-Morera et al, 2019). ALT telomeres represent a particularly extreme example of such vulnerable genomic regions and are subject to very high and persistent levels of replication stress (O’Sullivan and Greenberg, 2025). Indeed, despite its genome-wide functions, SLX4IP loss manifests particularly at ALT telomeres as SLX4IP deficiency induces pronounced BLM-dependent telomeric replication stress characterized by elevated ATR signaling, telomere fragility, and APB accumulation. Notably, our data suggest that these phenotypes occur without increased telomeric DNA synthesis, indicating that replication-associated stress accumulates at telomeres without promoting productive DNA extension in SLX4IP-deficient cells. We propose that SLX4IP keeps replication stress at levels that are conducive to recombination-coupled DNA synthesis rather than directly promoting break-induced replication. In its absence, elevated ssDNA gaps and replication stress may stimulate excessive recombination-associated processes that are inefficient or abortive. Viewed together with our prior work (Panier et al, 2019), these findings suggest that SLX4IP acts both at the level of BLM-dependent replication regulation and recombination intermediate processing, highlighting the importance of SLX4IP-dependent control of BLM activity in maintaining genome stability.

In addition to its genome-wide functions in controlling BLM, SLX4IP may additionally exert context-specific control over BLM at ALT telomeres by restraining BLM-driven unwinding of unligated Okazaki fragments, which is a source of lagging-strand stress that is especially relevant at ALT telomeres (Jiang et al, 2024). Here, hyperactive BLM aberrantly unwinds unligated Okazaki fragments, generating ssDNA and toxic 5’flap intermediates. Loss of SLX4IP could exacerbate this process by failing to constrain BLM-dependent Okazaki fragment unwinding at ALT telomeres. Indeed, iPOND data suggest increases in lagging-strand factor (including FEN1) recruitment to DNA in the absence of SLX4IP.

Negative regulation of BLM is essential in ALT cells to balance replication stress and recombination, thereby maintaining telomere stability (O’Sullivan and Greenberg, 2025). While FANCM has previously been implicated in counteracting BLM-dependent replication stress (Silva et al, 2019), our findings indicate that SLX4IP functions in a parallel pathway to limit replication stress at ALT telomeres. Co-depletion of FANCM and SLX4IP exacerbates replication stress and induces synthetic lethality that is rescued by BLM loss, highlighting excessive BLM activity as a central driver of cytotoxicity. Together, SLX4IP and FANCM act to counteract the hyperactivity of BLM, ensuring that replication stress is managed to allow for recombination without compromising cell viability.

Importantly, we observe synthetic lethality between SLX4IP and FANCM across multiple ALT cell lines, albeit with varying magnitude. Although the severity of the growth defect differs among ALT models, the phenotypes are reproducible and directionally consistent. This variability is consistent with the established heterogeneity of ALT mechanisms across distinct cellular contexts (Cesare et al, 2009; Henson et al, 2009; Loe et al, 2020). Notably, despite quantitative differences between systems, the underlying mechanistic relationship linking SLX4IP loss, replication stress, and FANCM dependency remains preserved.

These findings have potential clinical implications. ALT cells are particularly vulnerable to FANCM loss (Pan et al, 2017; Lu et al, 2019; Silva et al, 2019; Pan et al, 2019), which supports the idea of FANCM inhibition as a therapeutic strategy for ALT cancers (O’Rourke et al, 2019). Notably, SLX4IP is inactivated in a subset of ALT cancers (Panier et al, 2019). The ALT-specific synthetic lethal interaction between SLX4IP and FANCM suggests that the efficacy of FANCM inhibitors may be enhanced in SLX4IP-deficient ALT tumors. Our observations across multiple ALT cell lines suggest that SLX4IP status could serve as a marker for replication stress vulnerabilities of ALT-positive cancers, warranting further investigation in clinical settings.

**Figure 7:**
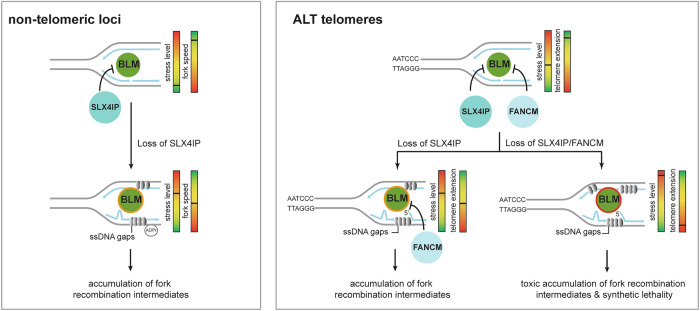
Model of SLX4IP as a replication stress response factor genome-wide and at ALT telomeres. Model of SLX4IP function at replication forks genome-wide and at ALT telomeres. Left: SLX4IP localizes broadly across the genome, where it maintains replication fork speed and limits the BLM-dependent accumulation of post-replicative ssDNA gaps that drive local ADP-ribosylation (ADPr) and checkpoint activation. SLX4IP restrains aberrant BLM-mediated processing of replication intermediates that would otherwise generate pathological fork structures and slow down replication progression. The molecular basis by which SLX4IP restrains BLM to prevent pathological fork remodeling and recombination remains unknown. Right: ALT telomeres, which are subject to chronic replication stress, are particularly vulnerable to hyperactive BLM activity. Here, acting in a pathway parallel to FANCM, SLX4IP limits BLM-driven replication stress to maintain levels compatible with productive recombination-coupled DNA synthesis. One potential mode of regulation at ALT telomeres is to constrain the BLM-dependent unwinding of unligated Okazaki fragments, thereby preventing the formation of toxic ssDNA gaps and 5′ flap intermediates. Loss of SLX4IP alone elevates telomeric replication stress but can still be tolerated in the presence of FANCM. However, combined loss of SLX4IP and FANCM shifts this balance toward excessive, non-productive replication stress, resulting in the BLM-dependent generation of ssDNA gaps, accumulation of toxic replication and recombination intermediates, and synthetic lethality.

In summary, our findings define a previously unrecognized importance for SLX4IP as a regulator of replication stress, not only at telomeres but also genome-wide (Fig. 7). SLX4IP preserves replication fork stability by maintaining replication fork speed, by limiting ssDNA gap formation, and restraining pathological BLM-dependent processing of replication intermediates. The effects of SLX4IP loss are most evident at ALT telomeres, where SLX4IP acts in a pathway parallel to FANCM to restrain BLM activity, ensuring that replication stress and recombination are managed without generating cytotoxic intermediates. Together, these results establish SLX4IP as a key modulator of BLM activity at replication forks genome-wide and at ALT telomeres and highlight its potential as a biomarker and therapeutic target in ALT-positive cancers.

## Methods

**Table:** Reagents and tools table

Reagent/resource	Reference or source	Identifier or catalog number
**Experimental models**		
Human: U2OS	The Francis Crick Institute Cell Services	
Human: U2OS SLX4IP-/- clones 1-4	Panier et al, 2019	
Human: U2OS TRF1-FOK1 (WT and D450A)	Tang et al, 2013, Roger Greenberg	
Human HEK293	The Francis Crick Institute Cell Services	
Human SAOS-2	EditCo	
Human SAOS-2, SLX4IP-/-pool	EditCo, this study	
Human RPE1-hTert	The Francis Crick Institute Cell Services	
Human: U2OS SLX4IP−/− clones 1–2	Panier et al, 2019	
Human eHAP iCAS9 clone #3	Hewitt et al, 2021, Graeme Hewitt	
Human eHAP iCas9 sgAAVS1 (CTRL)	This study	
Human eHAP iCas9 sgSLX4IP clones 1-2	This study	
Human WI38-VA13	The Francis Crick Institute Cell Services	
Human WE38-VA13 SLX4IP−/− clone 1	Panier et al, 2019	
**Recombinant DNA**		
n/a		
**Antibodies**		
Mouse monoclonal anti-SLX4IP (clone G4)	Santa Cruz Biotechnology	Cat#sc-377066; RRID: AB_2752253
Sheep polyclonal anti-SLX4IP	MRC PPU	Cat#S587D
Rabbit polyclonal anti-phosphoSer33-RPA	Abcam	Cat#ab211877; RRID: AB_2818947
Rabbit monoclonal anti-phosphoSer345-CHK1 (113D3)	Cell Signaling Technology	Cat#2348; RRID: AB_331212
Rabbit polyclonal anti-BLM	Abcam	Cat#ab2179; RRID: AB_2290411
Mouse monoclonal anti-PML (clone PG-M3)	Santa Cruz	Cat#sc-966; RRID: AB_628162
Mouse monoclonal anti-TRF2 (clone 4A794)	Abcam	Cat#ab13579; RRID: AB_300474
Rabbit monoclonal anti-Poly/Mono-ADP Ribose (clone D9P7Z)	Cell Signaling	Cat#89190S
Mouse monoclonal anti-FANCM	Novus biologicals	NBP2-50418
Rabbit polyclonal anti-RAP1	Bethyl Laboratories Inc.	Cat#A300-306A; RRID: AB_162721
Mouse monoclonal anti-alpha-Tubulin (clone B-5-1-2)	Sigma-Aldrich	Cat#T6074; RRID: AB_477582
Mouse monoclonal anti-Vinculin (clone VIN-11-5)	Sigma-Aldrich	Cat#V4505; RRID: AB_477617
Mouse polyclonal anti-Histone3	Abcam	Cat#ab1791; RRID: AB_302613
Rabbit monoclonal anti-RPA32 (clone 9H8)	Abcam	Cat#ab2175; RRID: AB_302873
Mouse monoclonal anti-pS139-H2AX (clone JBW301)	Millipore	Cat#05-636; RRID: AB_2755003
Goat anti-Mouse IgG (H + L) Highly Cross-Adsorbed Secondary Antibody, Alexa Fluor 488-conjugated	Thermo Fisher Scientific	Cat#A11029; RRID: AB_2534088
Goat anti-Rabbit IgG (H + L) Highly Cross-Adsorbed Secondary Antibody, Alexa Fluor 488-conjugated	Thermo Fisher Scientific	Cat#A11034; RRID: AB_2576217
Goat anti-Mouse IgG (H + L) Highly Cross-Adsorbed Secondary Antibody, Alexa Fluor 546-conjugated	Thermo Fisher Scientific	Cat#A11030; RRID: AB_2534089
Goat anti-Rabbit IgG (H + L) Highly Cross-Adsorbed Secondary Antibody, Alexa Fluor 546-conjugated	Thermo Fisher Scientific	Cat#A11035; RRID: AB_2534093
Goat polyclonal anti-rabbit, horseradish peroxidase-conjugated	Merck Millipore	Cat#12-348; RRID: AB_11214240
Goat polyclonal anti-mouse, horseradish peroxidase-conjugated	Merck Millipore	Cat#12-349; RRID: AB_390192
Rabbit polyclonal anti-sheep, horseradish peroxidase-conjugated	Merck Millipore	Cat#12-342; RRID: AB_11213530
Rat monoclonal anti-BrdU (clone BU1/75 (ICR1))	Abcam	Cat#ab6326; RRID: AB_305426
Mouse monoclonal anti-BrdU (clone B44)	BD	Cat#347580 RRID: AB_400326
**Oligonucleotides and other sequence-based reagents**		
Non-targeting Control Pool siRNA	Horizon Discovery	D-001810-10
FANCM siRNA	Horizon Discovery	L-021955-00
BLM siRNA	Horizon Discovery	L-007287-00
LIG1 siRNA	Horizon Discovery	L-011076-00
SLX4IP siRNA	Horizon Discovery	L-048874-01
SLX4 siRNA	Horizon Discovery	L-014895-00
XPF siRNA	Horizon Discovery	L-019946-00
MUS81 siRNA	Horizon Discovery	L-016143-01
FEN1 siRNA	Horizon Discovery	L-010344-00
SMARCAL1 siRNA	Horizon Discovery	L-013058-00
FANCM forward: AATCTTGGCTCTAAGTGCCAC	Sigma-Aldrich	
FANCM reverse: TCTGCCCAATTAGCAGGTTAGTA	Sigma-Aldrich	
BLM forward: GGACCTTGACACCTCTGACAG	Eurofins	
BLM reverse: GGATTCAGCTCCTGCATACTCA	Eurofins	
LIG1 forward: GAAGGAGGCATCCAATAGCAG	Sigma-Aldrich	
LIG1 reverse: ACTCTCGGACACCACTCCATT	Sigma-Aldrich	
SLX4 forward: TTGGTCCTACAGCGAATGCAG	Eurofins	
SLX4 reverse: CATGTGCCGATGCTCCTACC	Eurofins	
XPF forward: CCTCTTTCGCCAGAAAAACAAAC	Eurofins	
XPF reverse: TTTACTGCTACATGGAACCTTGG	Eurofins	
MUS81 forward: ATCCTAGAGACCCAGCAAAC	Sigma-Aldrich	
MUS81 reverse: GAGGTTGTGGACGGAACCAT	Sigma-Aldrich	
FEN1 forward: ATGACATCAAGAGCTACTTTGGC	Sigma-Aldrich	
FEN1 reverse: GGCGAACAGCAATCAGGAACT	Sigma-Aldrich	
SMARCAL1 forward: ACAGCATCAGAGGACTAGCTC	Sigma-Aldrich	
SMARCAL1 reverse: CACTGGCTTACAAGACTCCCT	Sigma-Aldrich	
**Chemicals, enzymes, and other reagents**		
Hydroxyurea	Sigma-Aldrich	4000465
PDD00017273, 10 mM	Selleckchem	S8862
Olaparib	Biomol	Cay10621
Cisplatin	Sigma-Aldrich	232120
Thymidine	Sigma-Aldrich	T1895
Doxycycline	Sigma-Aldrich	D9891
Shield1 ligand	Takara Bio	632189
4-Hydroxytamoxifen	Sigma-Aldrich	H6278
5-Chloro-2’-deoxyuridine	Sigma-Aldrich	C6891
5-Iodo-2’-deoxyuridine	Sigma-Aldrich	l7125
S1 Nuclease	Thermo Fisher Scientific	EN0321
Colcemid	Thermo Fisher Scientific	15212012
Exonuclease III	Promega	M1811
Benzonase	Sigma-Aldrich	E1014
Micrococcal Nuclease	Sigma-Aldrich	N5386
16% formaldehyde	Life Technologies	28908
formamide	Carl Roth	P040.2
Blocking Reagent, Roche	Merck Millipore	11096176001
TelC-Cy5 PNA probe	PNA Bio	F1003
Bovine Serum Albumin (BSA)	Fisher Scientific	BP9703
DAPI	Sigma-Aldrich	D9542
Prolong Diamond Antifade mountant	Life Technologies	P36970
Click-iT Plus EdU Cell Proliferation Kit for Imaging, Alexa Fluor 488	Thermo Fisher Scientific	C10637
DharmaFECT 1	Horizon Discovery	T-2001
5X siRNA buffer	Horizon Discovery	B-002000-UB-100
Luna Universal qPCR Master Mix-SYBR green	NEB	M3003
Luna Script RT SuperMix Kit	BioLabs	E3010
DMEM High w/ glutamax	Life Technologies	61965059
McCoy’s 5 A Medium (modified)	Life Technologies	26600023
IMDM	Life Technologies	12440061
DMEM Nutrient Mixture F-12 1:1	Life Technologies	11330057
Fetal Bovine Serum	PAN biotech	p30-3602
Tetracycline-free FBS	PAN biotech	P30-3602
**Software**		
Fiji	NIH	https://imagej.net/software/fiji/downloads
Graphpad Prism 10	GraphPad Software	https://www.graphpad.com/
Adobe Illustrator v 25.1	Adobe	https://www.adobe.com/de/products/illustrator.html
FlowJo v10.9.0.	FlowJo	https://www.flowjo.com/
fastaRegexFinder	Dario Beraldi	https://github.com/dariober/bioinformatics-cafe/tree/master/fastaRegexFinder
Instant Clue	Hendrik Nolte	Nolte et al, 2018
**Other**		
n/a		

### Cell culture

U2OS, HEK293, and WI38-VA13 cells were cultured in Dulbecco’s modified Eagle medium with Glutamax supplemented with 10% (v/v) fetal bovine serum (FBS). SAOS-2 cells were cultured in McCoy’s 5A Medium (modified) with 15% (v/v) FBS, RPE1-hTert cells in Dulbecco’s modified Eagle medium nutrient mixture F-12 1:1 with 10% (v/v) FBS, and eHap cells in Iscove’s Modified Dulbecco’s Medium with Tetracycline-free 10% (v/v) FBS. Cells were maintained using standard tissue culture procedures and kept in a humidified incubator set to 37 °C and 5% CO_2_. Cells were regularly tested for Mycoplasma. Cells were frozen in 90% (v/v) FBS and 10% (v/v) DMSO, transferred to Mr. Frosty freezing containers (Nalgene), and kept for at least 1 day at −80 °C. For long-term storage, cells were transferred into a liquid N2 tank.

iCas9 eHAP AAVS1 and SLX4IP KO cells were generated by lentiviral transduction of the sgRNA guides GGGGCCACTAGGGACAGGAT and TGGAAGTTCGCAAACAGCAC, respectively, obtained from Human CRISPR Knockout Pooled Library (Brunello) (Pooled Library #73179). iCas9 eHAP cells were plated in 2 mL/well of IMDM + 10% TET-FREE FBS in a six-well plate to obtain 60–70% confluency the next day. The day after seeding, the medium was exchanged with 2 mL/well of IMDM + 10% TET-FREE FBS + 8 µg/mL of Polybrene (sc-134220), and 75 µl of the lentivirus was added to the cells. Cells were incubated with the virus for 24 h followed by 48 h of selection with Puromycin (2 µg/mL). Cas9 expression was induced with 2 µg/ml Doxycycline for 72 h. Single cells were then seeded in a 96-well plate in conditioned medium, and single clones were validated by PCR and SLX4IP immunoblotting.

SAOS-2 knockout cells were purchased from EditCo. The following guides were used to target the SLX4IP locus in these cells: UGAUUGCAGAGAUAGCAAGG; AGAAUCCAUGGGACAAGCAA; GGGGCUUCCUGUUCAAAAGC.

### Double thymidine block

U2OS cells were plated in 10-cm cell culture dishes to obtain 30–40% confluency the next day. The plates were equipped with #1.5 glass coverslips for later processing for EdU Click-iT reactions and indirect immunofluorescence. The day after seeding, cells were treated with 2 mM thymidine for 18 h. Cells were released from the thymidine block by washing with 10 mL DPBS three times and adding fresh growth medium for 8 h. After the release, cells were again treated with 2 mM thymidine for 18 h to synchronize them in early S-phase. After that, cells were released by washing with 10 mL DPBS three times and adding fresh growth medium for 7.5 h to allow cells to accumulate in G2-phase. At the 6 h timepoint post-release, cells were treated with 10 µM EdU for 1.5 h to later visualize telomere synthesis in G2 cells. Coverslips were transferred to 24-well plates and processed for EdU Click-iT reactions and indirect immunofluorescence of SLX4IP and RAP1 as described below.

### Cell cycle analysis by flow cytometry

Asynchronous and U2OS cells synchronized to G2 phase were rinsed with 1× PBS, trypsinized for 15 min, and resuspended in DMEM. Cells were centrifuged at 500× *g* for 5 min and medium was discarded. Cell pellets were resuspended with 10 mL cold PBS on ice and centrifuged at 500× *g* for 5 min at 4 °C. Cells were resuspended in 1 mL cold PBS and centrifuged for 1 min in a microcentrifuge. PBS was discarded, and cells were resuspended in 300 µL PBS. While gently vortexing the cell suspension, 300 µL of 8% (w/v) formaldehyde in PBS was added drop-wise for fixation. After incubating cells for 15 min at RT, 900 µL 1× PBS was added on top and cells were centrifuged for 1 min in a microcentrifuge. The fixative was discarded, cells were resuspended in 1 mL 1× PBS, and centrifuged for 1 min in a microcentrifuge. After discarding PBS, cells were resuspended in 200 µL storage buffer (3% (v/v) FBS, 0.09% (w/v) sodium azide in 1× PBS) and kept at 4 °C up to 1 week.

On the day of flow cytometry analysis, cells were stained with DAPI. For that, the storage buffer was removed by centrifuging cells for 1 min in a microcentrifuge. Cells were resuspended in 1 mL filtered 1 mg/mL BSA/1× PBS solution and centrifuged for 1 min in a microcentrifuge. After that, the BSA/PBS solution was discarded, and cells were permeabilized in 1 mL 0.1% (v/v) Triton X-100 in PBS and rotated at RT for 20 min. Cells were centrifuged for 1 min in a microcentrifuge, and the permeabilization buffer was discarded. Following one wash in 1 mL filtered 1 mg/mL BSA/1× PBS, cells were resuspended in 200 µL DAPI staining solution (1 µg/mL DAPI, 1 mg/mL BSA in 1× PBS), passed through a 50 µM cell strainer, and analyzed by flow cytometry. Gating and analysis were performed manually using FlowJo v10.9.0.

### TRF1-FOKI induction

U2OS TRF1-FokI and U2OS TRF1-FokI D450 cells were seeded to obtain a confluency of 30–40% on the day of transfection. The plates were equipped with #1.5 glass coverslips for later processing for EdU Click-iT reactions and indirect immunofluorescence. SLX4IP was depleted through an siRNA-mediated knockdown. Forty-eight hours post transfection, cells were incubated with 40 ng/mL of doxycycline overnight to induce expression of the mCherry-tagged TRF1-FokI mRNA. Post doxycycline incubation, cells were treated for 4 h with 1 µM of Shield-1 to stabilize the TRF1-FokI protein, 1 µM of 4-Hydroxytamoxifen to promote its nuclear internalization, and 10 µM of EdU to visualize break-induced replication at the telomeres. Coverslips were transferred to 24-well plates and processed for EdU Click-iT reactions and indirect immunofluorescence of pS139-H2AX as described in the respective sections.

### RNA interference

RNAi transfections were performed using DharmaFECT 1 (Horizon Discovery) in a forward transfection mode. Cells were treated with a final amount of 40 nM in all knockdown experiments except for experiments including knockdown of SLX4IP, where cells were treated with a final amount of 80 nM siRNA. The transfection reagent volumes used were according to the manufacturer’s manual. Cells were plated to obtain 30–40% confluency on the day of transfection. The next day, Dharmafect 1 and Opti-MEM were mixed by vortexing, shortly spun down, and incubated for 5 min at RT. siRNA was added to the transfection mix, vortexed, spun down, and incubated for 20 min at RT. Meanwhile, the cell culture medium was replaced with fresh medium. After incubation, medium was added to the transfection reagent/siRNA complex, briefly vortexed, shortly spun down, and dispensed dropwise onto the cells. 5-7 h after transfection, the medium was substituted for fresh medium. Cells were further processed 48–72 h post-transfection.

### Indirect immunofluorescence

Cells were grown on #1.5 glass coverslips in a 24-well plate. Where indicated, cells were treated with hydroxyurea (HU; 4 mM, 24 h) and water as a control or with Olaparib (1 µM or 10 µM, 48 h) and DMSO as control before fixation. In siRNA knockdown experiments, cells were fixed ~72 h post transfection. Cells were washed with 1 mL/well 1× PBS once at RT and pre-extracted with 0.5 mL/well ice-cold pre-extraction buffer (20 mM HEPES pH 7.5, 20 mM NaCl, 5 mM MgCl_2_, 300 mM sucrose, 0.5% NP-40, 1 mM DTT, phosphatase and protease inhibitor tablets) for 20 min on ice. The pre-extraction buffer was removed, and cells were immediately fixed with 2% (w/v) formaldehyde (Life Technologies) in PBS for 20 min at RT. After fixation, cells were washed with 1X PBS three times for 5 min respectively and then blocked with 0.5 mL/well Antibody Dilution Buffer (ADB; 10% (v/v) normal goat serum, 0.1% (v/v) Triton X-100, 0.1% (v/v) saponin in PBS) for at least 1 h. Cells were incubated with 30 µL primary antibody diluted in ADB in a humid chamber for 1 h at RT. The primary antibodies were diluted as follows: mouse anti-SLX4IP 1:50, rabbit anti-BLM 1:1000, mouse anti-PML 1:1000, rabbit anti-pS33-RPA 1:1000, rabbit anti-pS345-CHK1 1:50, rabbit anti-RAP1 1:2000, mouse anti-TRF2 1:500 and mouse anti-pS139-H2AX 1:10,000. After incubation in primary antibodies, cells were washed once with 1× PBS + 0.1% (v/v) Triton-X-100 for 5 min, followed by two washes with 1× PBS for 5 min. Each coverslip was then counterstained with 300 µL Alexa Fluor secondary antibodies (Thermo Fisher Scientific) diluted 1:1000 in ADB, for 1 h at RT. After washing once with 1× PBS + 0.1% (v/v) Triton-X-100 for 5 min, cells were washed with 1× PBS two times for 5 min. To visualize nuclei, cells were stained with 1 mL/well 0.1 µg/mL DAPI in 1× PBS for 5 min, followed by two more washes with 1 mL/well 1× PBS. Coverslips were mounted onto glass slides with 10 µL drops of Prolong Diamond Antifade mounting agent (Life Technologies) and left to dry overnight at RT in the dark. Slides were stored at 4 °C. Pre-extraction, fixation, blocking, all wash steps, the secondary antibody incubation, and DAPI staining were carried out on a horizontal shaker. Starting from the addition of the fluorescent secondary antibodies, coverslips were always incubated in the dark.

For immunofluorescence of ADP ribose, cells were grown on #1.5 glass coverslips in a 24-well plate. Before fixation, live cells were treated with 10 µM PARG inhibitor PDD00017273 (Selleckchem) for 30 min. For the consecutive steps, two different procedures were followed: (A) Cells were washed with 1 mL/well 1× PBS once at RT and fixed with 0.5 mL/well ice-cold 100% methanol for 15 min at −20 °C. Methanol was kept at −20 °C overnight before using it for fixation. After fixation, cells were washed with 1 mL/well 1× PBS three times for 5 min each. Cells were always processed the same day for ADP ribose immunofluorescence. For that, cells were blocked with 0.5 mL/well blocking buffer (1× PBS/5% (v/v) normal goat serum/0.3% (v/v) Triton X-100) for 1 h at RT. Cells were then incubated in 300 µL rabbit anti-Poly/Mono-ADP Ribose (Cell Signaling) diluted 1:3200 in antibody dilution buffer (1× PBS/1% (w/v) BSA/0.3% (v/v) Triton X-100) overnight at 4 °C. After washing once with 1× PBS + 0.1% (v/v) Triton-X-100 for 5 min, cells were washed with 1× PBS two times for 5 min. Each coverslip was then counterstained with 300 µL goat anti-rabbit Alexa Fluor 488 secondary antibody (Thermo Fisher Scientific) diluted 1:1000 in ADB, for 1 h at RT. After washing once with 1× PBS + 0.1% (v/v) Triton-X-100 for 5 min, cells were washed with 1× PBS two times for 5 min. To visualize nuclei, cells were stained with 1 mL/well 0.1 µg/mL DAPI in 1× PBS for 5 min, followed by two more washes with 1 mL/well 1× PBS. Coverslips were mounted onto glass slides with 10 µL drops of Prolong Diamond Antifade mounting agent (Life Technologies) and left to dry overnight at RT in the dark. (B) Alternatively, cells were washed with 1 mL/well 1× PBS once at RT and pre-extracted in CSK + 0.5% (v/v) Triton X-100 (20 mM HEPES pH 8.0/100 mM NaCl/3 mM MgCl_2_/300 mM Sucrose/0.5% (v/v) Triton X-100) supplemented with 10 μM PARP inhibitor (Biomol) and PARG inhibitor (Selleckchem) on ice for 5 min. Cells were fixed with cold 3% (w/v) formaldehyde for 15 min and then permeabilized using ice-cold methanol/acetone (1:1) mix for 5 min, followed by another 5 min in pre-chilled 0.5% (v/v) Triton X-100 in 1× PBS. Cells were blocked in 3% (w/v) BSA in 1× PBS for 1 h at room temperature. Then, cells were incubated in 300 µL rabbit anti-Poly/Mono-ADP Ribose (Cell Signaling) diluted 1:3200 in antibody dilution buffer (1× PBS/0.1% (v/v) Tween-20/5% (v/v) goat serum) at 4 °C. After washing once with 1X PBS + 0.1% (v/v) Triton-X-100 for 5 min, cells were washed with 1× PBS two times for 5 min. Each coverslip was then counterstained with 300 µL goat anti-rabbit Alexa Fluor 488 secondary antibody (Thermo Fisher Scientific) diluted 1:1000 in ADB, for 1 h at RT. After washing once with 1× PBS + 0.1% (v/v) Triton-X-100 for 5 min, cells were washed with 1× PBS two times for 5 min. To visualize nuclei, cells were stained with 1 mL/well 0.1 µg/mL DAPI in 1× PBS for 5 min, followed by two more washes with 1 mL/well 1× PBS. Coverslips were mounted onto glass slides with 10 µL drops of Prolong Diamond Antifade mounting agent (Life Technologies) and left to dry overnight at RT in the dark. Slides were stored at 4 °C. All antibody incubations, all wash steps, and DAPI stainings were carried out on a horizontal shaker. Starting from the addition of the fluorescent secondary antibody, coverslips were always incubated in the dark.

### Telomeric peptide nucleic acid fluorescence in situ hybridization (PNA-FISH)

Cells were treated with 0.2 µg/ml of colcemid for 90 min to arrest cells in metaphase. Trypsinized cells were then incubated in 75 mM KCl for 30 min and pelleted at 1000 rpm for 5 min, fixed with methanol:acetic acid (3:1), spread on glass slides, and left overnight at room temperature to dry. The slides were rehydrated in PBS for 5 min, fixed in 4% formaldehyde for 5 min, treated with pre-warmed 1 mg/ml of pepsin (in 10 mM glycine pH 2) for 10 min at 37 °C, washed twice in 1× PBS for 5 min each, fixed in 4% formaldehyde for 5 min, and washed three times with 1× PBS for 5 min each. Next, slides were dehydrated in 70%, 85%, and 100% (v/v) ethanol for 15 min each and then air-dried. Metaphase chromosome spreads were hybridized with a Cy5-TelG 5’-(CCCTAA)_3_-3’ PNA probe (Bio-synthesis) in hybridizing solution (70% formamide, 0.5% blocking reagent (Roche), 10 mM Tris-HCl pH 7.2) for 120 s at 80 °C followed by 2 h at room temperature, washed twice with hybridization wash A (70% formamide, 10 mM Tris-HCl pH 7.2, 0.1% BSA) for 15 min each at room temperature, followed by 3 washes with hybridization wash B (0.1 M Tris-HCl pH 7.2, 0.15 M NaCl, 0.08% Tween-20) for 5 min each at room temperature. DAPI was added to the second last wash. Slides were mounted using ProLong Diamond antifade (Life Technologies).

### Immunofluorescence coupled to fluorescence in situ hybridization (IF-FISH)

Samples were processed as described in the section ‘indirect immunofluorescence’. After incubating cells with Alexa Fluor secondary antibodies (Thermo Fisher Scientific), cells were washed once with 1 mL/well 1× PBS + 0.1% (v/v) Triton-X-100 for 5 min followed by two washes with 1 mL/well 1× PBS for 5 min. After that, cells were fixed again with 1 mL/well 2% (w/v) formaldehyde in PBS for 20 min at RT and then washed three times with 1 mL/well 1× PBS. Next, coverslips were dehydrated on ice in 0.5 mL/well ice-cold 70%, 95%, and 100% (v/v) ethanol for 5 min each and then air-dried. Coverslips were processed no later than the next day after dehydration. Glass slides were preheated for 1 min on a heat block set to 75 °C. Then 30 µL drops of hybridizing solution (10 nM TelC-Cy5 PNA probe (PNA Bio) in 70% (v/v) formamide, 0.5% (w/v) blocking reagent (Roche), 10 mM Tris-HCl pH 7.2) were placed on the glass slides and heated for 1 min. Dry coverslips were placed with cells facing downwards into the hybridizing solution and hybridized for 90 s at 75 °C followed by 2 h at RT in a humid chamber. After incubation, cells were washed twice with 0.5 mL/well washing buffer (70% (v/v) formamide, 10 mM Tris-HCl pH 7.2) for 15 min at RT. After that, coverslips were washed with 1 mL/well 1× PBS for 5 min. To visualize nuclei, cells were stained with 1 mL/well 0.1 µg/mL DAPI in 1× PBS for 5 min, followed by two more washes with 1 mL/well 1× PBS. Coverslips were mounted onto glass slides with 10 µL drops of Prolong Diamond Antifade mounting agent (Life Technologies). Secondary antibody incubations, all wash steps, and DAPI stainings were carried out on a horizontal shaker. Starting from the addition of the fluorescent secondary antibodies, coverslips were always incubated in the dark.

### EdU Click-IT reaction

Cells were grown on #1.5 glass coverslips in a 24-well plate. Before fixation, live cells were incubated in 0.5 mL/well medium supplied with 10 µM EdU for 30 min. They were washed with 1 mL/well 1× PBS once at RT and pre-extracted with 0.5 mL/well ice-cold pre-extraction buffer (0.2% (v/v) Triton-X-100 in 1× PBS) for 10 min on ice. The pre-extraction buffer was removed, and cells were immediately fixed with 4% (w/v) formaldehyde (Life Technologies) in PBS for 15 min at RT. The EdU incorporation was visualized with the Click-iT Plus EdU Alexa Fluor 488 Imaging Kit (Thermofisher). Permeabilization, blocking, washing steps, and the EdU Click-IT reaction were performed according to the manufacturer’s manual. To each coverslip, 250 µL of the Click-iT Plus reaction cocktail was added. After washing cells with 1 mL/well of 3% (w/v) BSA in 1× PBS, cells were washed two more times with 1× PBS for 5 min. To visualize nuclei, cells were stained with 1 mL/well 0.1 µg/mL DAPI in 1× PBS for 5 min, followed by two more washes with 1 mL/well 1× PBS. Coverslips were mounted onto glass slides with 10 µL drops of Prolong Diamond Antifade mounting agent (Life Technologies). Blocking, the EdU Click-IT reaction, all wash steps, and the DAPI staining were carried out on a horizontal shaker.

### Image acquisition and analysis of IF data

Images of IF and IF-FISH stainings were acquired with a Leica SP8-X or SP8-DLS inverted confocal microscope equipped with a 63× glycerol objective. Following the acquisition, images were imported into ImageJ (NIH) for automated quantification.

Unblinded but automated quantification of foci, colocalization analysis of foci, and nuclear size measurements were performed in Fiji. Foci and colocalizations were quantified using the speckle inspector plugin from the Biovoxxel Toolbox (Brocher, J., *biovoxxel/BioVoxxel-Toolbox: BioVoxxel Toolbox v2.6.0 (biovoxxel-toolbox_v2.6.0a)*. 2023, Zenodo). To correct the foci numbers to the nuclear size, the number of foci of each nucleus was divided by the respective nucleus area. For the corrected total nuclear fluorescence intensity (CTNF), the integrated density and nuclear sizes were quantified automatically with a custom Fiji script. Background measurements were taken manually from each image by choosing three regions of interest in each image and calculating their average mean intensity. The CTNF was then calculated by the following equation: Integrated density – (Area of nucleus * average mean intensity of background).

### Whole-cell extracts

Cells were rinsed with 1× PBS, trypsinized, and collected in DMEM. Cells were pelleted by centrifugation at 300× *g* for 5 min and washed with 1× PBS. Cell pellets were snap-frozen on dry ice and stored at −80 °C. For lysis, cell pellets were thawed on ice and resuspended in lysis buffer (50 mM Hepes-KOH, pH 7.5, 100 mM KCl, 2 mM EDTA, 0.5% (v/v) IGEPAL, 10% (v/v) glycerol, 1 mM DTT, 1× protease and phosphatase inhibitors). Lysates were incubated on ice for 30 min and sonicated in the bioruptur for 10 cycles, 30 s on and off. Cell lysates were clarified by centrifugation at 13,000× *g* for 20 min at 4 °C. Protein concentration was determined using the BCA method (DC protein assay (Biorad)) according to the manufacturer’s instructions. Lysates were denatured in 1× Laemmli Sample Buffer and 5 mM DTT for 5 min at 95–100 °C and stored at −20 °C or directly used for SDS-PAGE.

### Chromatin fractionation

HEK293 cells were plated in 10 cm cell culture dishes and treated with 4 mM hydroxyurea for 4 h, or 24 h, or treated with water as a control. Cells were washed once with 1× PBS, trypsinized, and resuspended in cell culture medium. The cell suspension was centrifuged at 300× *g* for 3.5 min. Medium was discarded, and cell pellets were washed once with 1× PBS. After the supernatant was discarded, pellets were either snap-frozen and stored at −80 °C or further processed for lysis and fractionation. Cell pellets were resuspended in cold EBC1 buffer (50 mM Tris-HCl pH 7.5, 100 mM NaCl, 0.05% (v/v) IGEPAL, 1 mM EDTA, 1 mM DTT, 1× protease and phosphatase inhibitors (Pierce Protease Inhibitor Tablets EDTA-free, Pierce Phosphatase Inhibitor Mini Tablets, Thermo Fisher Scientific)) and lysed for 5 min on ice. Lysates were centrifuged at 1000× *g* for 5 min at 4 °C. The supernatant was the soluble fraction, transferred to fresh tubes, and preserved. Cell pellets were washed once with 500 µL EBC1 buffer. After the supernatant was discarded, the pellets, which are the chromatin fraction, were resuspended in 150 µL EBC2 buffer (50 mM Tris-HCl pH 7.5, 300 mM NaCl, 5 mM CaCl_2_, 1× protease and phosphatase inhibitors) and supplemented with 10 U micrococcal nuclease (50 µL of 1U/5 µL stock). Pellets were solubilized for at least 15 min at 300 rpm and 30 °C. The chromatin-enriched lysate was cleared by centrifugation at 16,000× *g* at 4 °C for 15 min. The soluble and chromatin fractions were snap-frozen and stored at −80 °C till further processing. To prepare fractions for SDS-PAGE and immunoblotting, the protein concentrations were determined using the BCA method (DC protein assay (Biorad)) according to the manufacturer’s instructions. Lysates were denatured in 1× Laemmli Sample Buffer and 5 mM DTT for 5 min at 95–100 °C and stored at −20 °C or directly used for SDS-PAGE.

### SDS-PAGE and immunoblotting

Proteins were separated by SDS-PAGE. Proteins were transferred onto a nitrocellulose membrane (Biorad) at 100 V for 2 h. After transfer, the membrane was blocked in 5% (w/v) skim milk/ TBST (1X TBS/ 0.1% (v/v) Tween-20) for at least 30 min at RT and incubated with the indicated primary antibody diluted in 5% skim milk/ TBST for 1 h at RT or overnight at 4 °C. The primary antibodies sheep anti-SLX4IP, mouse anti-Vinculin, rabbit anti-BLM, and mouse anti-alpha-Tubulin were diluted 1:1000. Mouse anti-RPA32 was diluted 1:100, and rabbit anti-H3 was diluted 1:12,000. The membrane was then washed five times for 5 min with TBST, incubated with a horseradish peroxidase-conjugated secondary antibody diluted 1:5000 in 5% skim milk/TBST for 1 h at RT, and washed five times again for 5 min with TBST. The immunoblot was developed on Hyperfilm (Fisher Scientific) using ECL Western Blotting Reagent (Thermo Fisher Scientific). All incubations were carried out on a horizontal shaker.

For FANCM immunoblotting, proteins were separated by SDS-PAGE using self-cast 7.5% acrylamide gels at 100 V for 15 min, followed by 200 V for at least 1 h. Proteins were transferred onto a nitrocellulose membrane (Biorad) at 100 V for 2 h. After transfer, the membrane was blocked in 5% (w/v) BSA/PBST (1× PBS/0.1% (v/v) Tween-20) for at least 30 min at RT and incubated with the indicated primary antibody diluted in 5% BSA/PBST overnight at 4 °C. The primary antibody, mouse anti-FANCM (Novus Biologicals) was diluted 1:500. The membrane was then washed five times for 5 min with PBST, incubated with a horseradish peroxidase-conjugated secondary antibody diluted 1:5000 in 5% BSA/PBST for 1 h at RT, and washed again five times for 5 min with PBST. The immunoblot was developed on Hyperfilm (Fisher Scientific) using ECL Western Blotting Reagent (Thermo Fisher Scientific). All incubations were carried out on a horizontal shaker.

### Clonogenic survival assay

Forty-eight hours after siRNA transfection, cells were trypsinized, strained with 40 µM cell strainers, counted, and re-plated into six-well dishes in conditioned medium. For that, conditioned medium was collected from confluent cell culture flasks and filtered with a sterile 0.22 µM filter. Generally, conditioned medium was stored for up to 2 weeks at 4 °C. Each condition was plated into a six-well plate in duplicates or triplicates. Remaining cells were collected in cell pellets for validating knockdowns by qPCR. For the survival assay, cells were grown for 8–11 days and fixed in a 20% (v/v) methanol/0.4% (w/v) crystal violet solution for 10 min. Crystal violet was removed, and residual dye was washed off with deionized water. Plates were placed upside down and left to dry overnight. Plates were imaged with the GelCount scanner (Oxford Optronix) at a 600 dpi resolution, and images were saved as tif files. The area percentage of colonies covering each well was analyzed with a custom Fiji script.

### RT-qPCR

Cells were centrifuged at 300× *g* for 5 min. Medium was discarded, and cells were resuspended in 1 mL DPBS and transferred into RNAse-free microcentrifuge tubes. Cells were centrifuged again at 500× *g* for 5 min and the supernatant was discarded. Cell pellets were washed one more time with 0.5 mL DPBS, snap-frozen on dry ice, and stored at −80 °C. RNA isolation was performed with the RNeasy Mini Kit (Qiagen) according to the manufacturer’s instructions. RNA was reverse transcribed into cDNA with the Luna Script RT SuperMix Kit (Biolabs). RT-qPCR was performed using Luna Universal qPCR Master Mix-SYBR green (NEB).

### CUT&Tag

Protein A-Tn5 purification and assembly of transposomes. The 3XFLAG tag was removed from the 3Xflag-pA-Tn5-Fl plasmid (Addgene, #124601) through molecular cloning. For this, the 3Xflag-pA-Tn5-Fl plasmid was amplified using mutagenesis primers 5’-ACCATGGGTATGACCATGATTACGCC-3’ and 5’-CATGGTCATACCCATGGTATATCTCCTTC-3’, and subsequently cloned using the In-Fusion HDCloning Plus (Takara Bio, #638911). Protein A-Tn5 was then expressed and purified as previously described (Kaya-Okur et al, 2019). Annealing of Tn5MEDS was performed by mixing equal amounts of 200 µM pMENTS oligo with 200 µM Tn5ME-A or 200 µM Tn5ME-B followed by incubation at 95 °C for 2 min. The temperature was subsequently decreased to 25 °C in 5 °C increments, with each step held for 5 min. Annealed Tn5MEDS were stored at −20 °C. For transposome assembly, each 4 µL of 200 µM annealed Tn5MEDS-A and Tn5MEDS-B was combined with 100 µL of the protein A-Tn5 glycerol stock and incubated for 50 min at 23 °C. Assembled transposomes were stored at −20 °C.

Cleavage Under Targets and Tagmentation (CUT&Tag). For nuclei isolation, HEK293 or U2OS cells treated with or without 4 mM hydroxyurea (HU) for 24 h were incubated in NE1 buffer (20 mM HEPES-KOH pH 7.9, 10 mM KCl, 0.5 mM Spermidine, 0.1% Triton X-100, 20% glycerol, 1× cOmplete Proteinase Inhibitor) on ice for 5 min or 20 min, respectively. Nuclei were fixed with 0.1% formaldehyde (Thermo Scientific, 28906) for 2 min at RT, followed by quenching with 75 mM glycine. Nuclei were pelleted by centrifugation at 1300×*g* for 4 min at 4 °C and washed with PBS. CUT&Tag experiments were performed as described previously (Kaya-Okur et al, 2019), with minor modifications: Nuclei were not immobilized on concanavalin A-conjugated beads. Instead, nuclei were pelleted to remove supernatant through centrifugation at 600xg for 4 min at RT. All wash buffers were supplemented with 0.01% digitonin and 0.01% NP-40. Briefly, 1 × 10^5^ fixed nuclei were washed with 150-wash buffer (20 mM HEPES pH 7.5, 150 mM NaCl, 0.5 mM spermidine, Protease inhibitor, 0.01% digitonin, 0.01% NP-40) and incubated with mouse anti-SLX4IP (sc-377066, Santa Cruz Biotechnology) 1:100 in 50 µl of primary antibody binding buffer (150-wash buffer, 1% BSA, 2 mM EDTA) at 4 °C overnight. The next day, nuclei were incubated with rabbit anti-mouse antibody (1:100; ab46540, abcam) for 1 h at RT, followed by a wash with 150-wash buffer. Nuclei were resuspended in 50 µl of 300-wash buffer (20 mM HEPES pH 7.5, 300 mM NaCl, 0.5 mM spermidine, Protease inhibitor, 0.01% digitonin, 0.01% NP-40) with a 1:200 dilution of pA-Tn5 P7/P5 adapter complex and incubated for 1 h at RT, rotating. Nuclei were washed with 300-wash buffer followed by tagmentation in 50 µl of tagmentation buffer (300-wash buffer, 10 mM MgCl_2_). To avoid bias arising from cell cycle differences between HU-treated and untreated nuclei, only G1-phase nuclei were selected for further analysis using FACS. For that, nuclei were resuspended after tagmentation in 300 µl FACS sorting buffer (10% fetal bovine serum in PBS) supplemented with DAPI (1:1000, BD Pharmingen #564907). The suspension was filtered through a 35 µm cell strainer into a polypropylene tube. G1-phase nuclei were sorted using a FACSAria III cell sorter into 100 µl DNA binding buffer (Zymo Research, #D4004). Where applicable, 10,000 G1-phase nuclei were collected per sample. Tagmented DNA was isolated from sorted nuclei using DNA Clean & Concentrator kit (Zymo Research, #D4004) and eluted from the column in 23 µl Nuclease-free H_2_O. Libraries were amplified with barcoded i7 and i5 primers with NEBNext High-Fidelity 2X PCR Master Mix (NEB, M0541L). The optimal number of amplification cycles was determined via qPCR. Amplified libraries were subjected to double-sided size selection using 0.67x and 1.3x of High-Prep PCR magnetic beads (Magbio, #AC-60050). Libraries were sequenced for 100 cycles paired-ended on NextSeq1000 platforms. CUT&Tag experiments were performed with two biological per cell line with each two technical replicates.

#### CUT&Tag bioinformatics

Generation of a non-redundant union peak set for SLX4IP CUT&Tag. For each cell line, we combined the stringent SEACR peak calls obtained from all biological replicates (four ± HU and four ± SLX4IP, eight BED files in total per line) into a single, non-redundant region set that was used in all downstream analyses.

1. Input. Scaled, replicate-level peak BED files produced by SEACR were collected from the project directories
2. Quality control. A Bash wrapper halted with an error if no files were found and printed a warning if the expected eight files were not present, ensuring that every merge contained the full replicate set.
3. Sorting. All replicate peaks for a given cell line were concatenated and sorted with BEDTools sort (v 2.31.0; default parameters).
4. Merging. Overlapping or immediately adjacent intervals were collapsed with BEDTools merge (default parameters), generating the cell-line-specific union set.

Annotation of SLX4IP CUT&Tag peaks and generation of per-category summary. For each cell line, we classified the union peak set into genomic categories and exported a tab-delimited summary table that was subsequently plotted in GraphPad Prism (10.4.2).

1. Input: The cell-line-specific union peak BED file was imported into R (v 4.3.0) with GenomicRanges (Bioconductor 3.18).
2. Annotation tracks:
  Gene models: TxDb.Hsapiens.UCSC.hg38.knownGene. Promoters were defined as −1 kb/+250 bp around the transcription-start site; gene bodies comprised the remaining exonic + intronic sequence; distal regions were the 100 kb upstream flank exclusive of promoters.
  Fragile sites: experimentally defined common fragile-site coordinates supplied as a custom BED file. All tracks were trimmed to standard chromosomes and harmonized to UCSC sequence-level style.
3. Peak annotation and summary: Full-containment overlaps were calculated with countOverlaps (default settings). Peaks were assigned in the priority order FragileSite > Promoter > GeneBody > Distal; peaks matching none of the tracks were labeled Unannotated. Category counts and percentages were written to per_category_peak_summary.tsv.

The same workflow was executed for HEK293 and U2OS, differing only in the input peak file.

Coverage quantification, RPKM normalization and log₂(HU/SLX4IP) ratio calculation. For every genomic region set we quantified CUT&Tag signal in four merged libraries (U2OS HU, U2OS SLX4IP, HEK HU, HEK SLX4IP), converted the counts to RPKM, and derived log₂ ratios between HU-treated and SLX4IP-depleted samples.

1. Input. Region BED files and four duplicate-filtered, hg38-aligned BAM files.
2. Raw coverage. Region-level read counts were extracted with BEDTools multicov (v 2.31.0, default parameters).
3. Library-size normalization. Total mapped reads per BAM were obtained with samtools view -c (v 1.19). Counts were converted to
  RPKM=reads in region × 10^9^/region length (bp)×total mapped reads.
4. Ratio calculation. For each region, the log₂(HU/SLX4IP) ratio was computed separately for U2OS and HEK; a pseudocount of 1 × 10⁻⁶ was added to zero values before taking the logarithm.
5. Output. Visualization by GraphPad Prism (10.4.2).

#### Log₂(HU/SLX4IP) coverage tracks and heat-map visualization

For each cell line, we generated log₂-ratio coverage tracks and visualized HU-induced changes across category-specific peak sets.

1. Input. CPM-normalized bigWig files for HU-treated and SLX4IP-depleted libraries (HEK293 and U2OS) together with BED files defining distal, fragile-site, promoter, and gene-body peak subsets.
2. Log₂-ratio bigWigs. Coverage was compared with deepTools bigwigCompare (v 3.5.2; --operation log2 --pseudocount 1 --skipZeroOverZero).
3. Signal matrix. Using computeMatrix reference-point ( ± 2.5 kb around region centers, 50 bp bins, --missingDataAsZero), per-category matrices were created and exported as both compressed (*.gz) and tab-delimited text files.
4. Visualization. Heat maps and average profiles were rendered with plotHeatmap (per-group mode, color map bwr, zMin = -1.2, zMax = 1.2), yielding PDF figures that summarize log₂(HU/SLX4IP) signal distributions across the selected peak categories.

The workflow was executed independently for HEK293 and U2OS; only the input bigWigs and BED region files differed. Genome browser visualization of SLX4IP occupancy. IGV (v2.16.1) was used to generate genomic snapshots of a promoter and fragile site showing differential SLX4IP occupancy. Normalized coverages of SLX4IP (cpm) and annotated regional files were used as input for the visualization.

#### Regex analysis of TTAGGG and CCCTC motifs

TTAGGG and CCCTC repeats were defined as regex search on the sequences for each SLX4IP-enriched peak with fastaRegexFinder by Dario Beraldi (https://github.com/dariober/bioinformatics-cafe/tree/master/fastaRegexFinder). The motif/non-motif ratio was calculated for each replicate. Replicate values were then statistically compared using a *t* test to determine whether the motif is significantly enriched. Of note, in the analysis, “non-motif” does not refer to a “random motif,” but rather to all reads that do not contain the TTAGGG and CCCTC motif, respectively. The ratio therefore indicates the frequency of motif-containing reads relative to all remaining reads in the dataset.

#### Distance analysis between SLX4IP peaks and replication origins

Distance analysis between SLX4IP peaks and replication origins (a long version with lots details).

Genomic distances between SLX4IP peaks and replication origin Q1 sites were calculated using R (v4.2.2) and Bioconductor packages. SLX4IP peak coordinates, which varied between experimental conditions as described in the manuscript, were defined as genomic summit positions derived from condition-specific peak-calling analysis and imported as BED-formatted genomic intervals. Coordinates of Q1 replication origins were obtained from a previously published genome-wide replication origin map (10.1038/s41467-020-18527-0) and converted into GRanges objects using GenomicRanges (v1.50.2).

For each SLX4IP peak interval, the nearest Q1 origin was identified using the nearest()function implemented in GenomicRanges, which determines the closest genomic interval based on linear genomic distance. The signed genomic distance was calculated as the difference between the genomic coordinate of the SLX4IP peak summit and the coordinate of the nearest Q1 origin summit. Absolute distances were subsequently calculated to quantify proximity independent of genomic orientation.

Absolute distances between SLX4IP peak summits and the nearest Q1 origin summits were discretized into fixed-width intervals of 200 bp to generate distance-frequency distributions. Binning was performed using base R (v4.2.2) by assigning each absolute distance value to a non-overlapping interval spanning the full observed distance range. Specifically, bin boundaries were defined using the seq()function to generate a continuous series of breakpoints at 200 bp increments between the minimum and maximum observed distances. Individual distance values were assigned to bins using the cut() function with left-inclusive interval boundaries. The number of peaks within each bin was quantified using the table() function to generate frequency counts. For randomized Q1 location, bedtools shuffle()function was employed in shell and the shuffled bed files were imported back to R.

To preserve the complete distance distribution, bins without observed peaks were explicitly retained and assigned zero counts. Distance-frequency tables were subsequently converted into data frame format for downstream aggregation and visualization. This approach ensured uniform resolution of distance measurements and enabled direct comparison of distance-frequency profiles across experimental conditions. Frequency distributions were generated by counting the number of SLX4IP peak summits falling within each distance bin.

Distance distributions were visualized using the ggplot2 package (v3.4.2). Frequency profiles were generated by plotting the number of SLX4IP peak summits as a function of absolute genomic distance from the nearest Q1 origin. Absolute distance was plotted on the x-axis and peak frequency on the y-axis, with distinct color coding used to distinguish experimental conditions. Fixed axis limits were applied to enable direct comparison between samples. Raw binned frequency values were used without smoothing to preserve the native distance-dependent distribution and avoid introducing artificial trends.

To evaluate whether SLX4IP peak summits were enriched in proximity to Q1 origins, permutation-based enrichment analysis was performed. For each sample, the cumulative frequency of peaks within a defined proximity window was calculated and compared to an empirical null distribution generated by random sampling from matched randomized genomic regions. This sampling procedure was repeated 10,000 times to establish the expected distribution under the null hypothesis of random positioning. Empirical *P* values were calculated as (k + 1)/(*n* + 1), where k represents the number of permutations with equal or greater enrichment than observed and *n* represents the total number of permutations. All analyses were performed using R with GenomicRanges (v1.50.2), dplyr (v1.1.2), tidyr (v1.3.0), purrr (v1.0.1), and ggplot2 (v3.4.2).

### iPOND coupled to mass spectrometry

#### iPOND

Cells were labeled with 10 mM EdU for 20 min. Cells were cross-linked in 1% fresh formaldehyde/PBS for 10 min (RT) and quenched with 1.25 M glycine. Cells were permeabilized in 0.25% Triton-X/PBS and washed once with 0.5% BSA/PBS, followed by PBS. Click reaction was performed for 1 h with PEG4-biotin azide (Invitrogen). The cells were subsequently lysed by sonication using a Diagenode Pico sonicator on ultra-high for 30 min. DNA-protein complexes were purified using streptavidin C1 magnetic beads for 1 h. Samples were washed (5 min each) with lysis buffer (1% SDS in 50 mM Tris, pH 8.0), low salt buffer (1% Triton X-100, 20 mM Tris, pH 8.0, 2 mM EDTA, pH 8.0, 150 mM NaCl), high salt buffer(1% Triton X-100, 20 mM Tris, pH 8.0, 2 mM EDTA, pH 8.0, 500 mM NaCl), lithium chloride buffer (100 mM Tris, pH 8.0, 500 mM LiCl, 1% Igepal), followed by two washes in lysis buffer. Captured proteins were eluted, and cross-links were reversed in 2× SDS sample buffer for 30 min at 95 °C.

#### In-gel digestion for mass spectrometry

All incubations were carried out at room temperature unless otherwise specified. iPOND samples were boiled at 95 °C for 30 min, concentrated below 20 µL using a vacuum centrifuge, and boiled again for 5 min with 1:1 Laemmli buffer. Samples were separated on 4–12% Tris-glycine mini gels (Novex, Invitrogen) at 120 V to 4/5 of gel length. Following electrophoresis, gels were incubated for 30 min in fixing solution (50% methanol, 1.2% phosphoric acid) to immobilize proteins. After briefly rinsing with water, gels were stained overnight in a solution containing 50% methanol, 1.2% phosphoric acid, 1.3 M ammonium sulfate, and 0.1% (w/v) Coomassie Brilliant Blue G-250.

Each lane was subsequently cut into small rectangular pieces for digestion. Gel pieces were destained with several rounds of washing using 25 mM ammonium bicarbonate, 50% ethanol for 15 min at 60 °C until gel pieces were destained completely. The supernatant was discarded before gel pieces were dehydrated in 100% ethanol for 10 min and were then dried in a vacuum centrifuge to remove residual liquid. Cysteine residues were reduced with 20 mM DTT, 50 mM ammonium bicarbonate for 20 min, and then alkylated with 80 mM chloroacetamide, 50 mM ammonium bicarbonate for 20 min. Reduction and alkylation compounds were removed with successive 10 min washes in (i) 50 mM ammonium bicarbonate, (ii) 25 mM ammonium bicarbonate, 50% ethanol, and (iii) 100% ethanol before drying in a vacuum centrifuge. For overnight digestion at 37 °C, 1 µg trypsin (Promega) was added to each lane in 50 mM ammonium bicarbonate. On the following day, peptides were eluted twice using 150 µL 80% acetonitrile, 0.2% formic acid, and were evaporated to dryness in a vacuum centrifuge.

#### LC-MS proteomics analysis

Peptides were resuspended in 20 µL 0.2% formic acid and were quantified by UV/Vis using a Nanodrop One (ε205 = 31, Thermo Fisher). For liquid chromatography mass spectrometry (LC-MS) analysis, 500 µg of peptide was loaded onto a house-packed ~45 cm capillary column (75-360 µm inner-outer diameter bare-fused silica shells with laser-pulled electrospray emitter tip; made in-house) filled with 1.7 µm, 130 Å pore size, Bridged Ethylene Hybrid (BEH) C18 particles (Waters), maintained at 50 °C inside a custom-built column heater. The source voltage was set to 2 kV, and the ion transfer tube was held at 275 °C. The elution gradient was delivered by a Vanquish Neo nanoLC system (Thermo Fisher) at a flow rate of 0.3 µL/min over a 73-min active gradient (from 3–46% Mobile Phase B). Mobile phase A consisted of 0.2% formic acid in water, and mobile phase B consisted of 80% acetonitrile and 0.2% formic acid in water.

Mass spectra were collected using data dependent acquisition (DDA) on an Orbitrap Ascend Tribrid mass spectrometer (Thermo Fisher) in positive mode with settings as follows: Orbitrap MS^1^ scans were performed at 240k resolution every 1 s over 300–1350 *m/z* with an automatic gain control (AGC) target of 1 × 10^6^, normalized AGC target of 250%, a maximum injection time of 50 ms, and an RF lens setting of 30%. MIPS (monoisotopic peak determination) was set to “peptide”, and the isolation window center was set to “most abundant peak”. Charge states 2–5 were included, and dynamic exclusion was set to 20 s with a  ±  5 ppm mass tolerance. MS^2^ scans were performed in the ion trap using an isolation window of 0.8 *m/z*, a normalized HCD collision energy of 24%, an ion trap scan rate set to “Turbo,” a scan range of 150–1350 *m/z*, a normalized AGC target of 250%, and a maximum injection time of 12 ms.

#### LC-MS data processing

The resulting LC-MS data were processed using separate DDA searches for each cell line on Fragpipe (v23.1). Searches used the Human reference proteome containing only Swiss-Prot entries downloaded from UniProt on January 9, 2025. The Closed Search default configuration was used for MSFragger, and MS1 Quantification was performed with IonQuant using default settings. The searched data was filtered by removing protein groups with less than 2 out of 3 valid MaxLFQ values per cell line, log_2_ transformed, and imputed using a left-censored imputation algorithm in Perseus (v2.1.3.0) with default settings.

## Supplementary information

Peer Review File

Source data Fig. 2

Source data Fig. 3

Source data Fig. 4

Source data Fig. 5

Source data Fig. 6

Expanded View Figures

## Data Availability

The CUT&Tag datasets are available in the Gene Expression Omnibus (GEO) repository under accession number GSE298728. The source data of this paper are collected in the following database record: biostudies:S-SCDT-10_1038-S44318-026-00790-4.
