# Supplementary figures and images for: SLX4IP limits replication stress globally and at ALT telomeres (part 1 of 2)
Source: EMBO J. 2026 May 7;45(12):4176–219. doi: 10.1038/s44318-026-00790-4 (PMC13269807; doi:10.1038/s44318-026-00790-4)

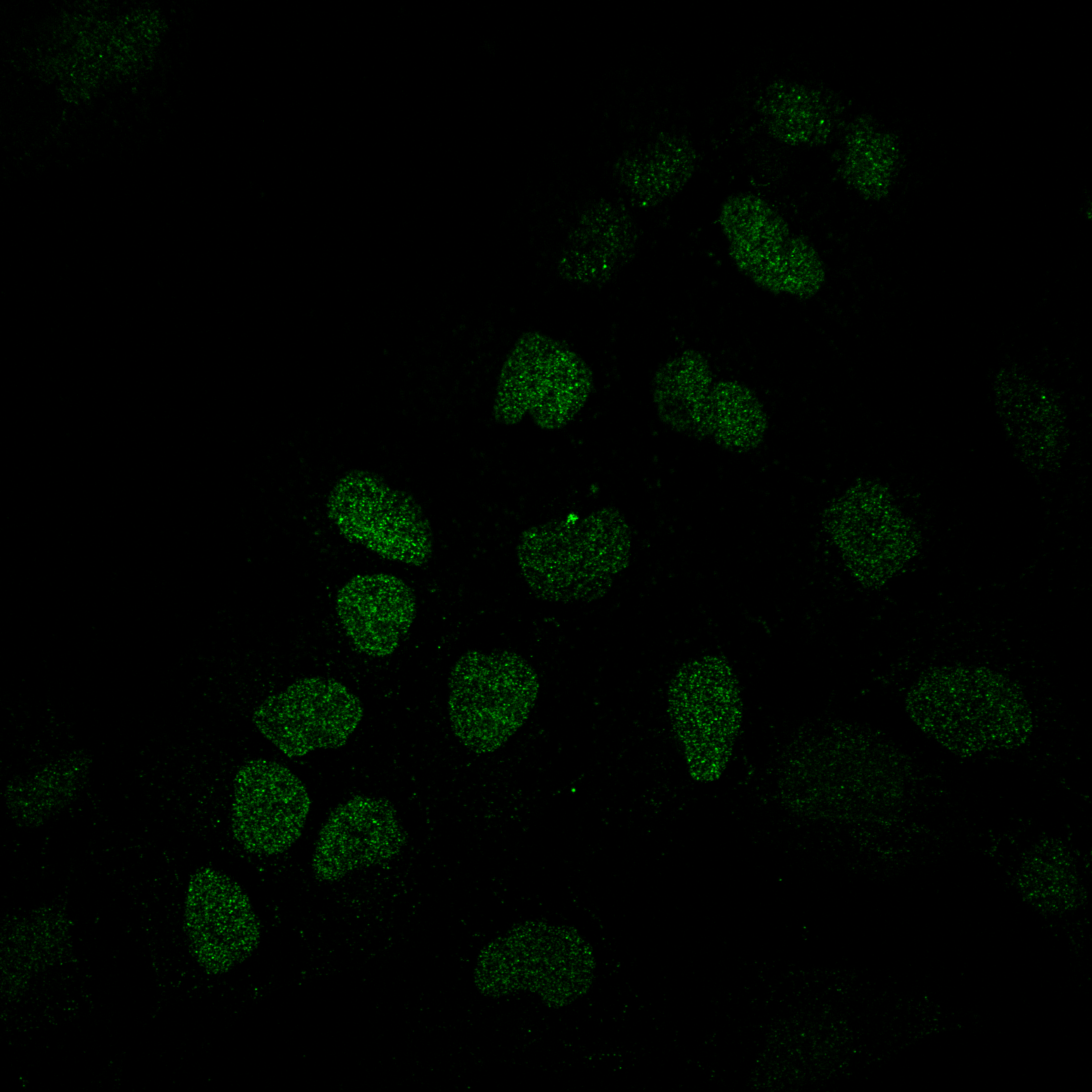

Supplement: Supplementary file 3 — Source data Fig. 3 [file 44318_2026_790_MOESM3_ESM.zip › Figure 3/Figure3B_ADPr_staining_U2OS/U2OS_clone3_ADPr.png]

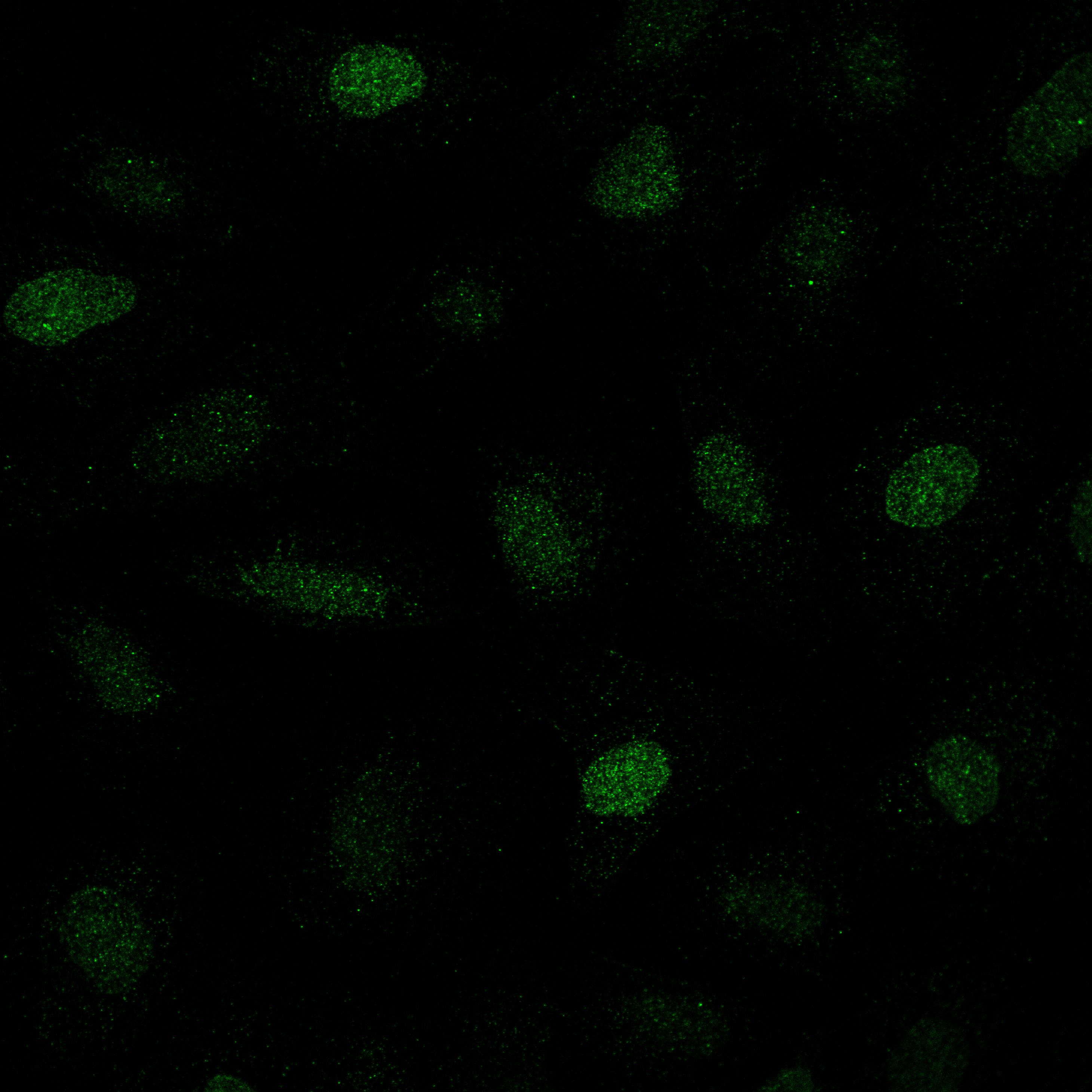

Supplement: Supplementary file 3 — Source data Fig. 3 [file 44318_2026_790_MOESM3_ESM.zip › Figure 3/Figure3B_ADPr_staining_U2OS/U2OS_clone2_ADPr.png]

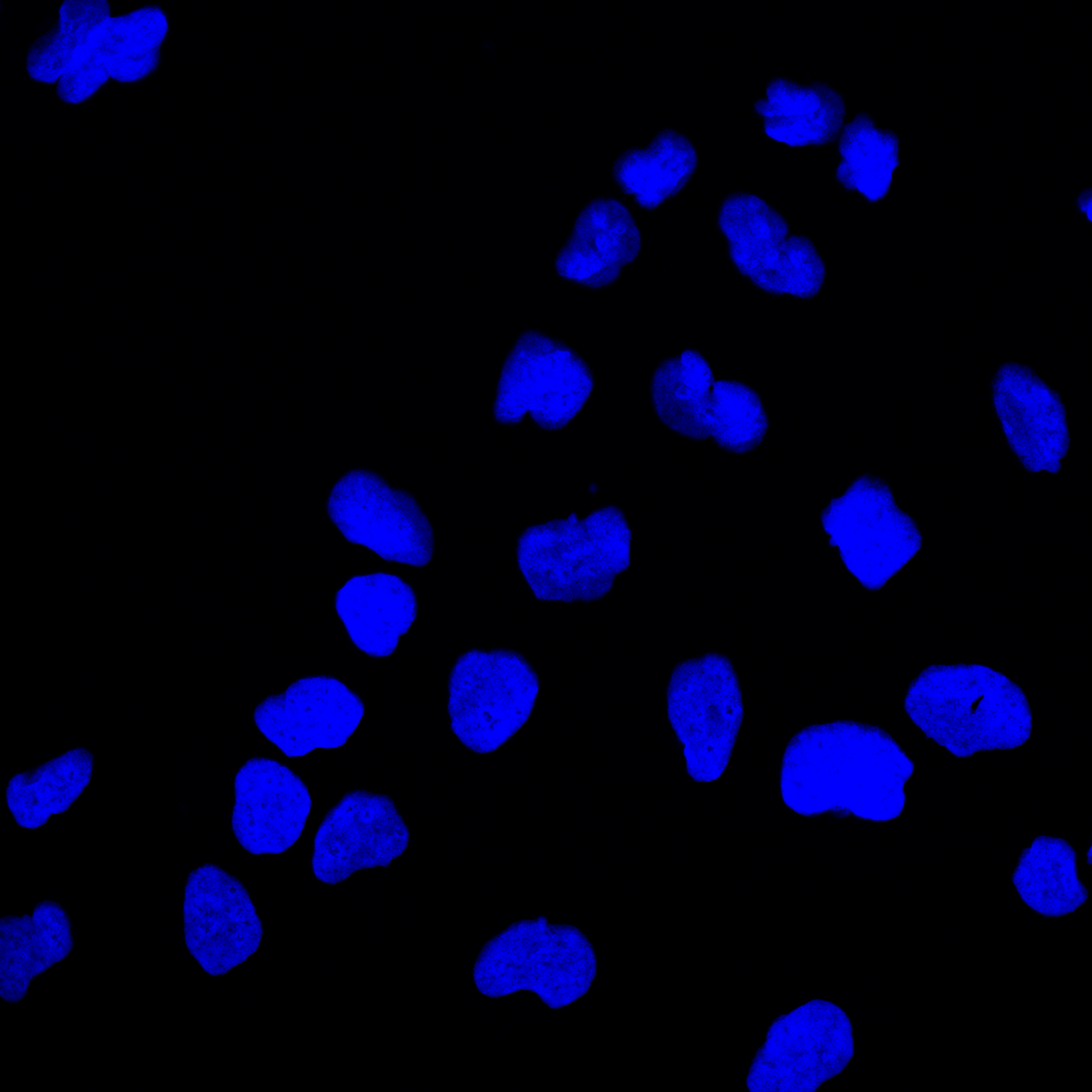

Supplement: Supplementary file 3 — Source data Fig. 3 [file 44318_2026_790_MOESM3_ESM.zip › Figure 3/Figure3B_ADPr_staining_U2OS/U2OS_clone3_DAPI.png]

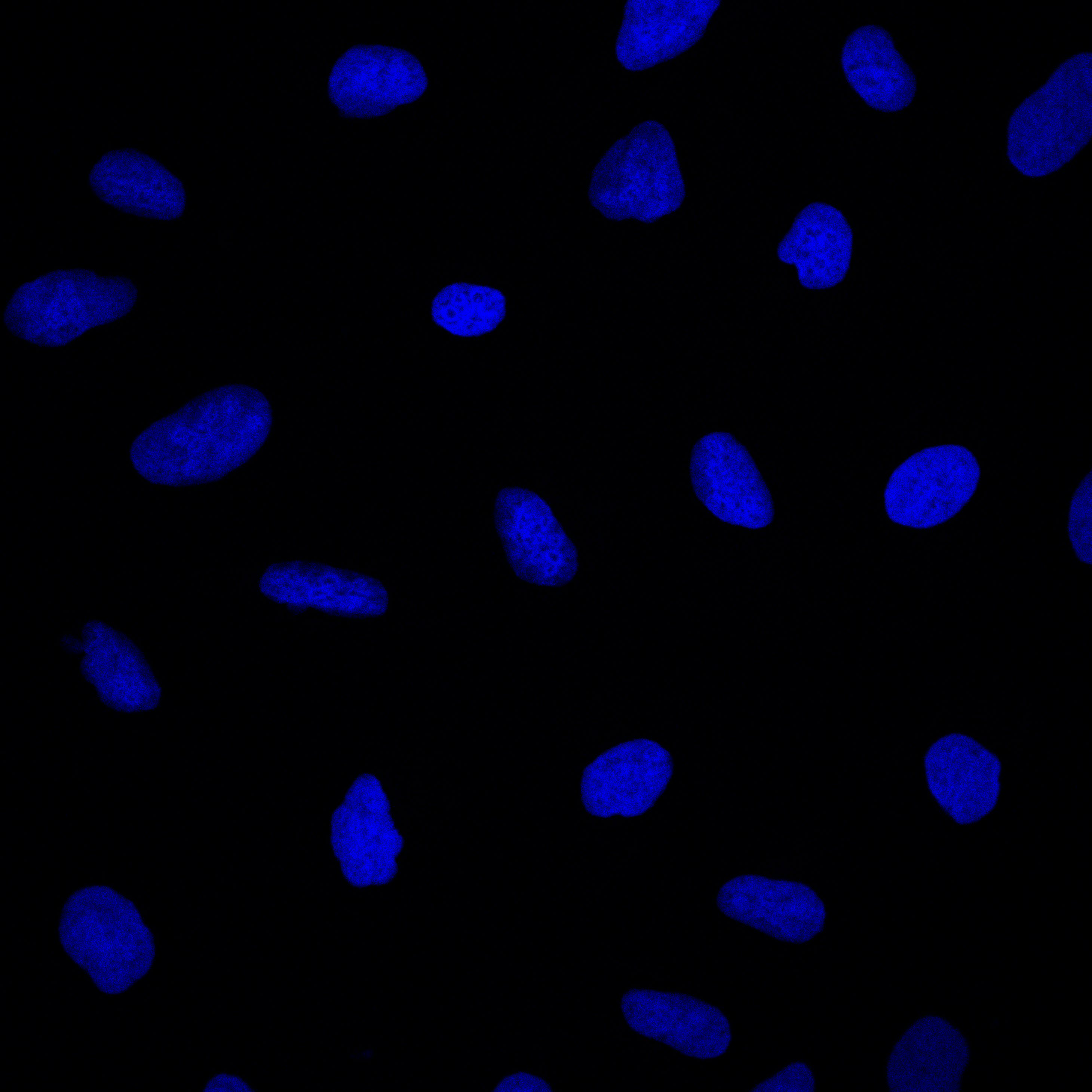

Supplement: Supplementary file 3 — Source data Fig. 3 [file 44318_2026_790_MOESM3_ESM.zip › Figure 3/Figure3B_ADPr_staining_U2OS/U2OS_clone2_DAPI.png]

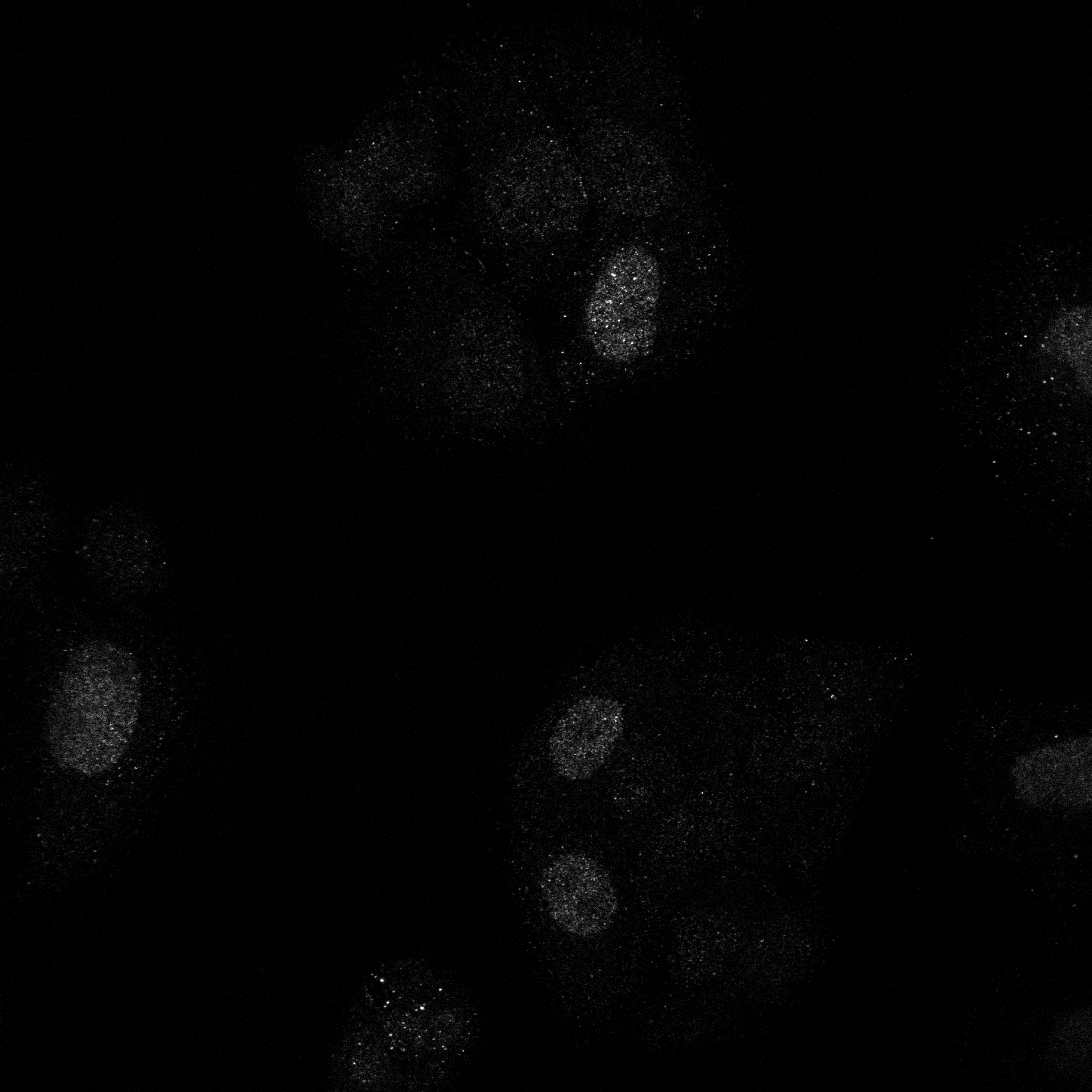

Supplement: Supplementary file 3 — Source data Fig. 3 [file 44318_2026_790_MOESM3_ESM.zip › Figure 3/Figure3D_ADPr_BLMrescue/U2OS_clone_1_siBLM_ADPr.tif]

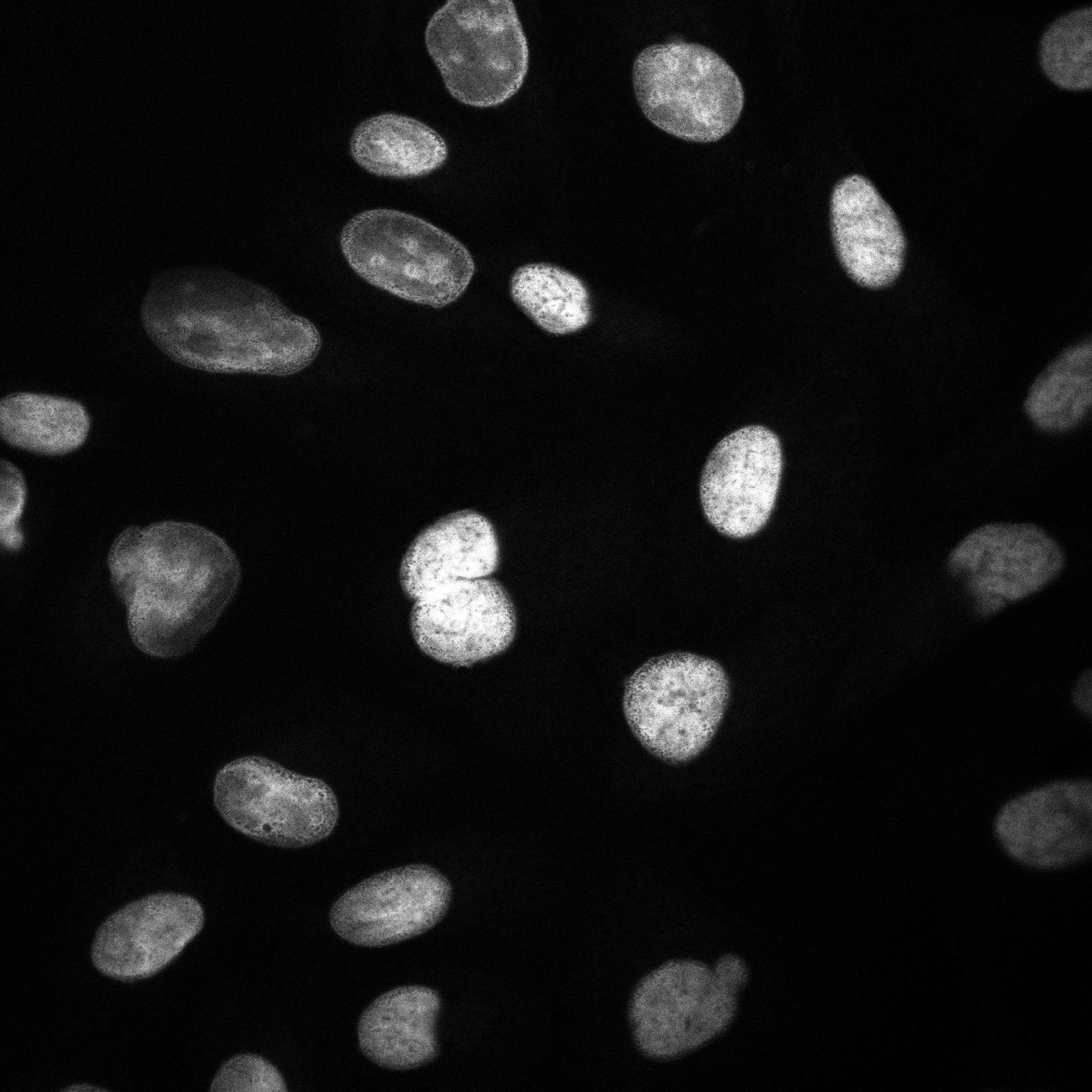

Supplement: Supplementary file 3 — Source data Fig. 3 [file 44318_2026_790_MOESM3_ESM.zip › Figure 3/Figure3D_ADPr_BLMrescue/U2OS_clone_1_siCTRL_DAPI.tif]

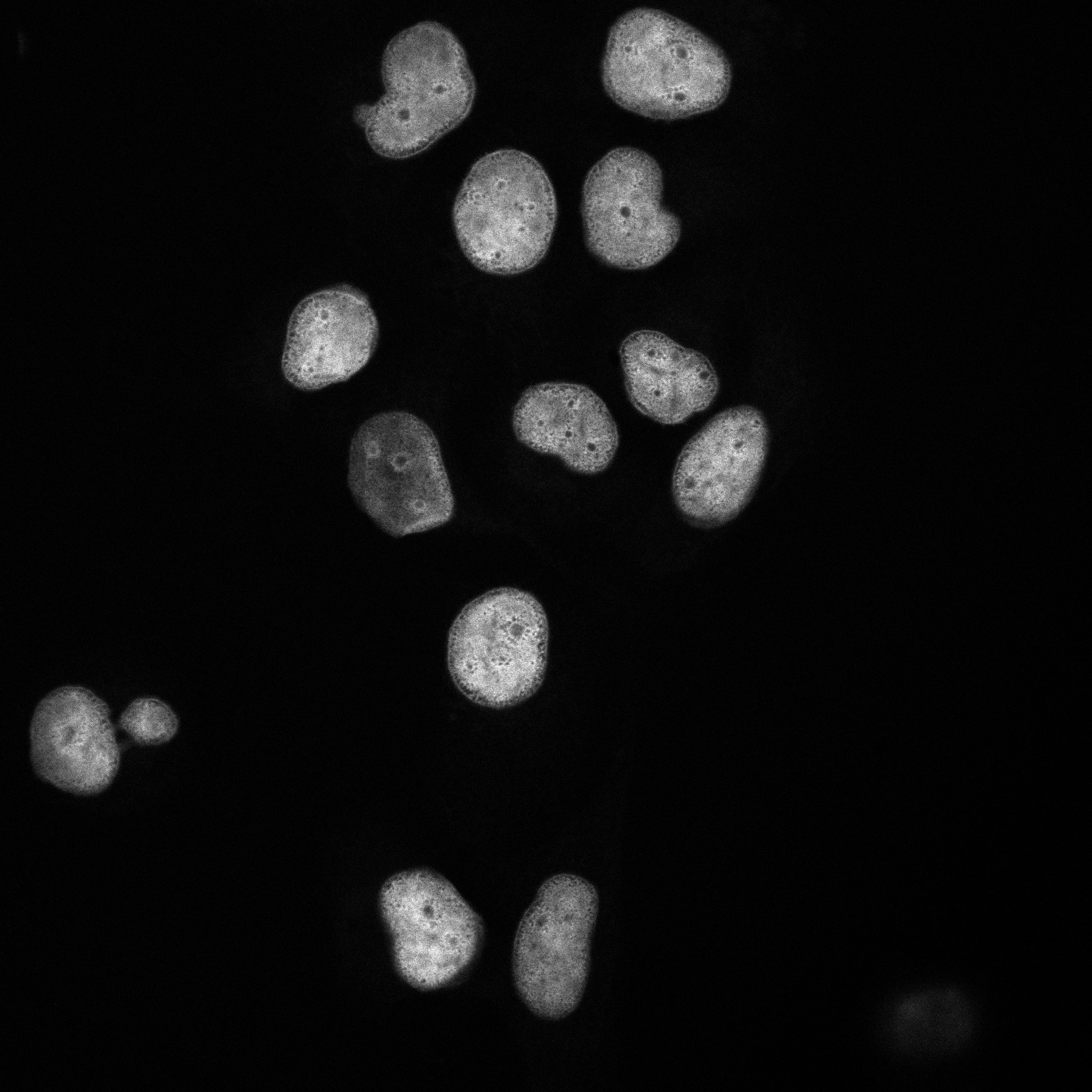

Supplement: Supplementary file 3 — Source data Fig. 3 [file 44318_2026_790_MOESM3_ESM.zip › Figure 3/Figure3D_ADPr_BLMrescue/U2OS_clone_2_siBLM_DAPI.tif]

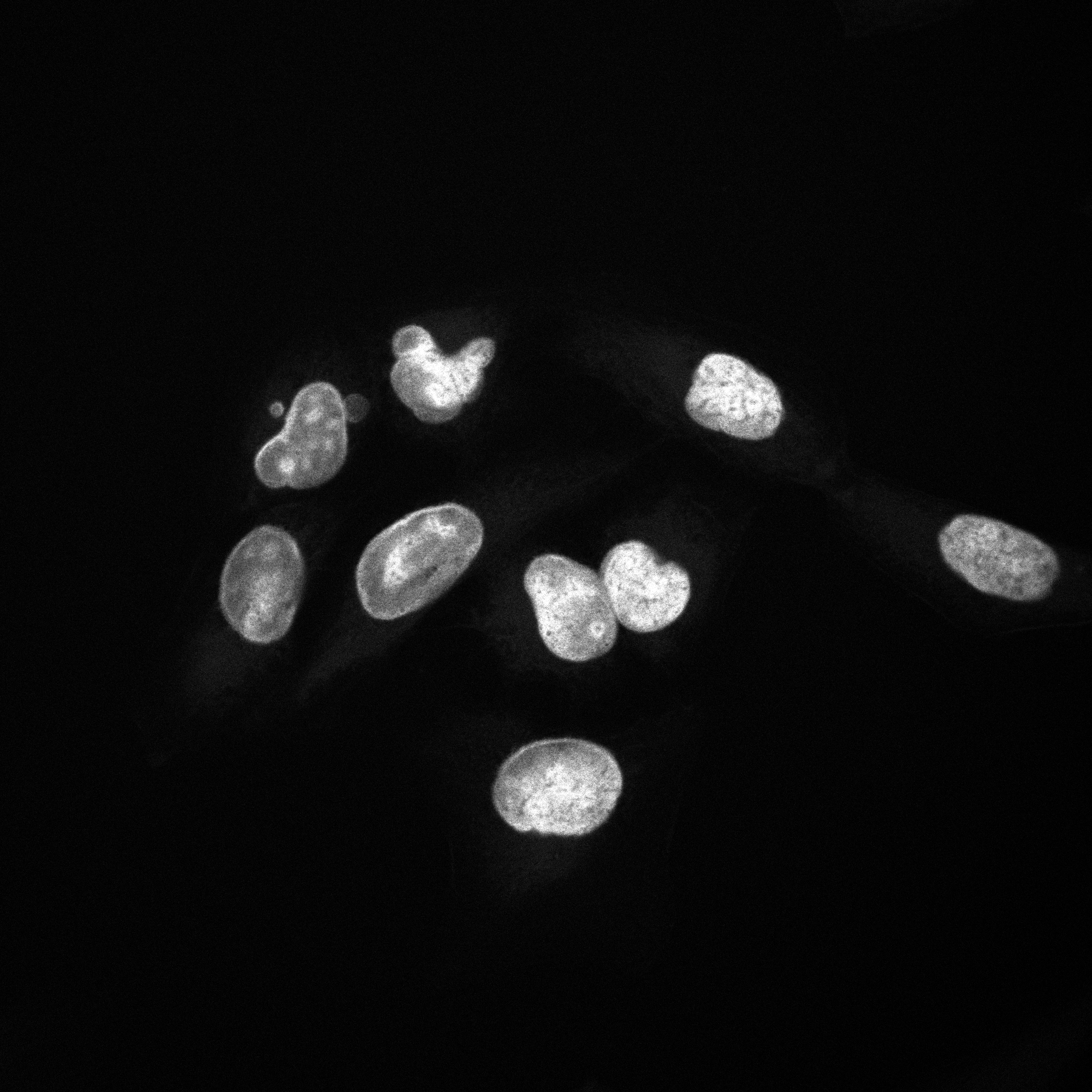

Supplement: Supplementary file 3 — Source data Fig. 3 [file 44318_2026_790_MOESM3_ESM.zip › Figure 3/Figure3D_ADPr_BLMrescue/U2OS_WT_siCTRL_DAPI.tif]

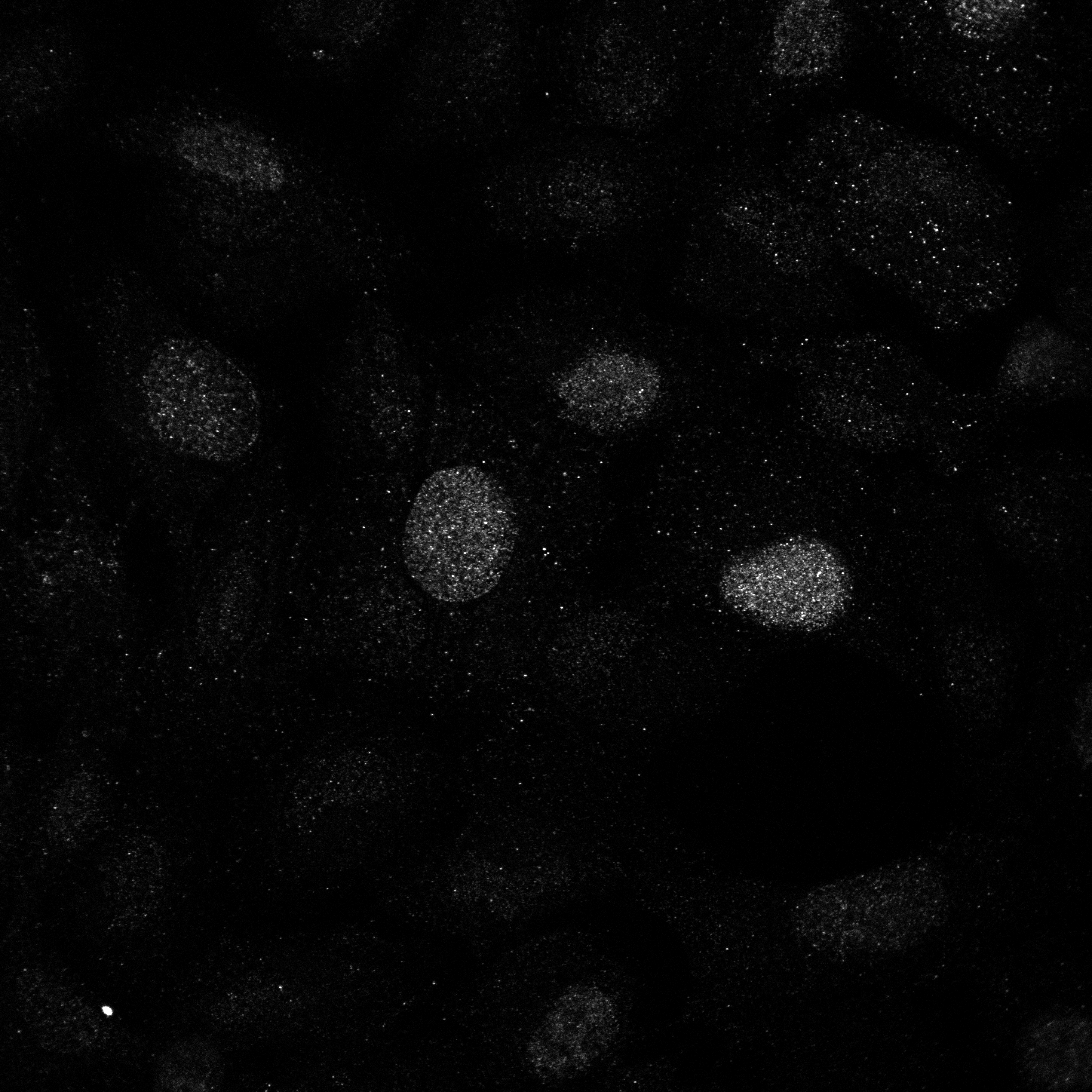

Supplement: Supplementary file 3 — Source data Fig. 3 [file 44318_2026_790_MOESM3_ESM.zip › Figure 3/Figure3D_ADPr_BLMrescue/U2OSWT_siBLM_ADPr.tif]

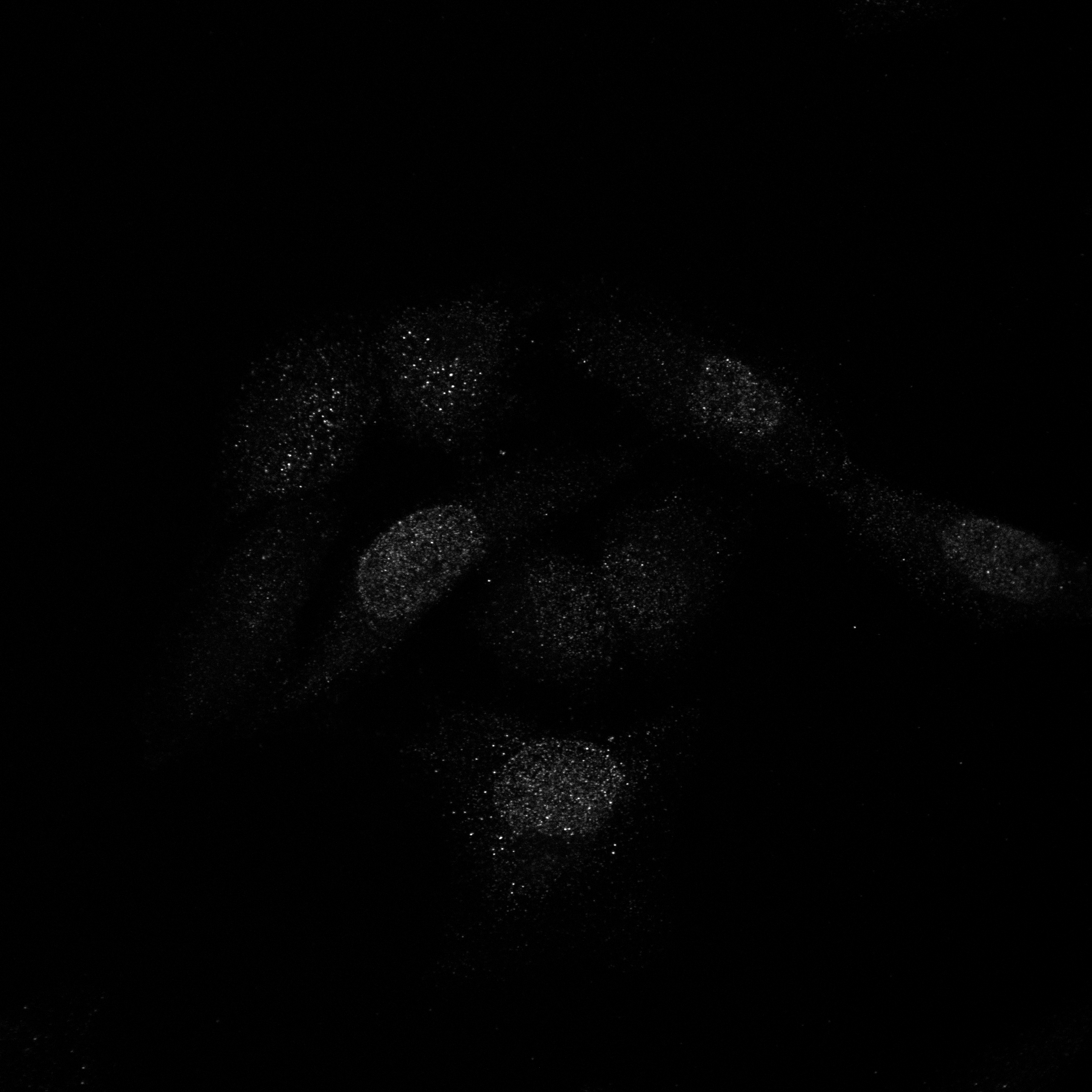

Supplement: Supplementary file 3 — Source data Fig. 3 [file 44318_2026_790_MOESM3_ESM.zip › Figure 3/Figure3D_ADPr_BLMrescue/U2OS_WT_siCTRL_ADPr.tif]

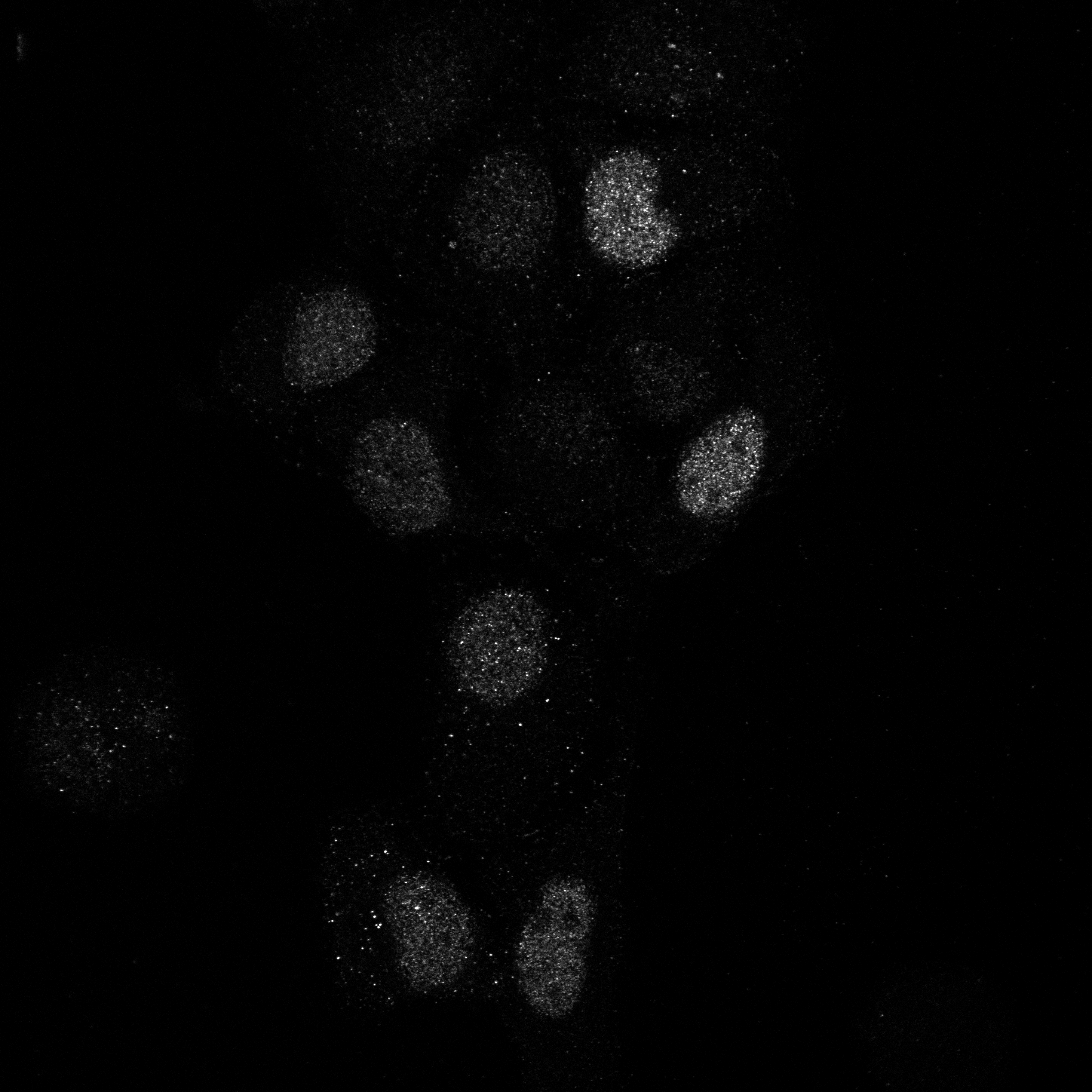

Supplement: Supplementary file 3 — Source data Fig. 3 [file 44318_2026_790_MOESM3_ESM.zip › Figure 3/Figure3D_ADPr_BLMrescue/U2OS_clone_2_siBLM_ADPr.tif]

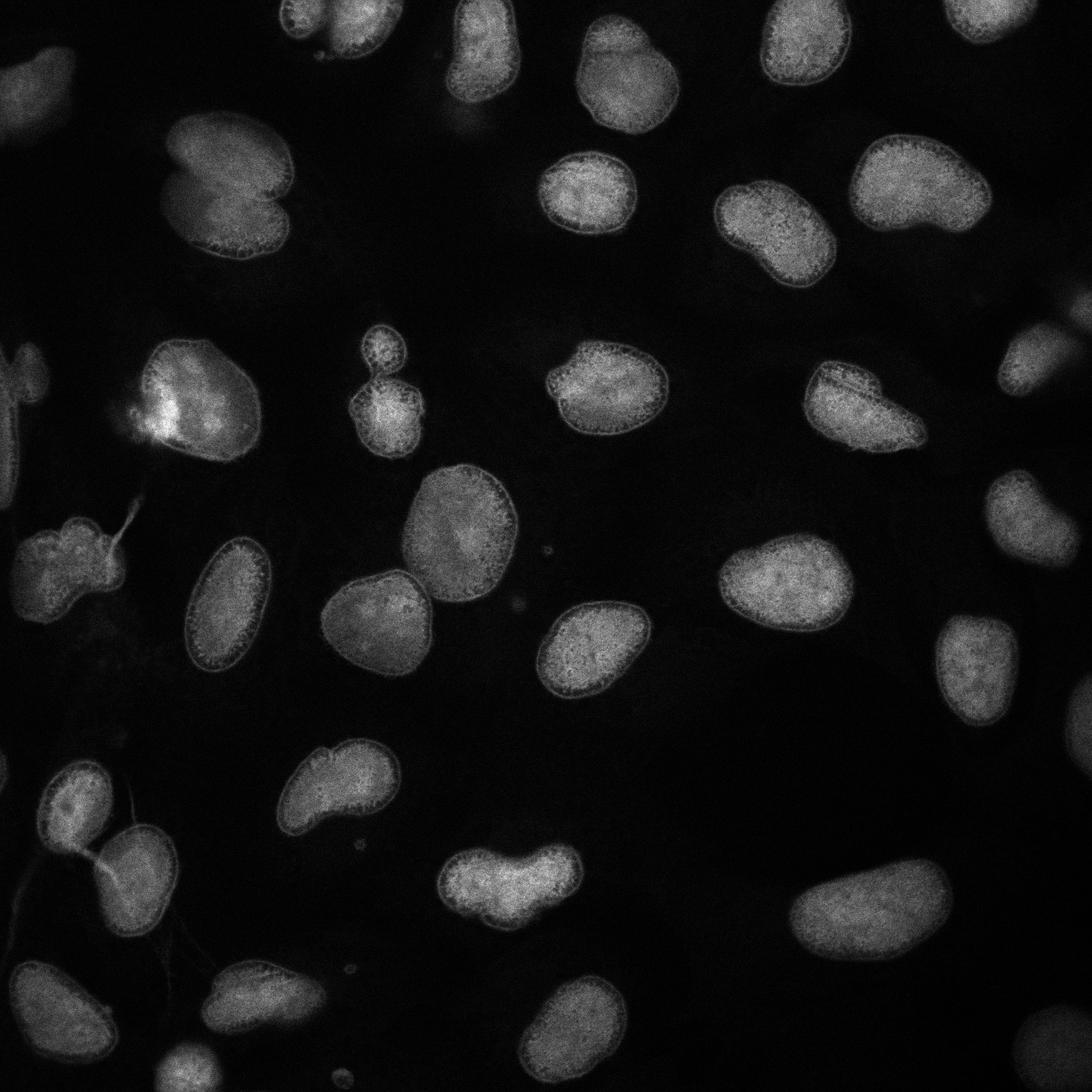

Supplement: Supplementary file 3 — Source data Fig. 3 [file 44318_2026_790_MOESM3_ESM.zip › Figure 3/Figure3D_ADPr_BLMrescue/U2OSWT_siBLM_DAPI.tif]

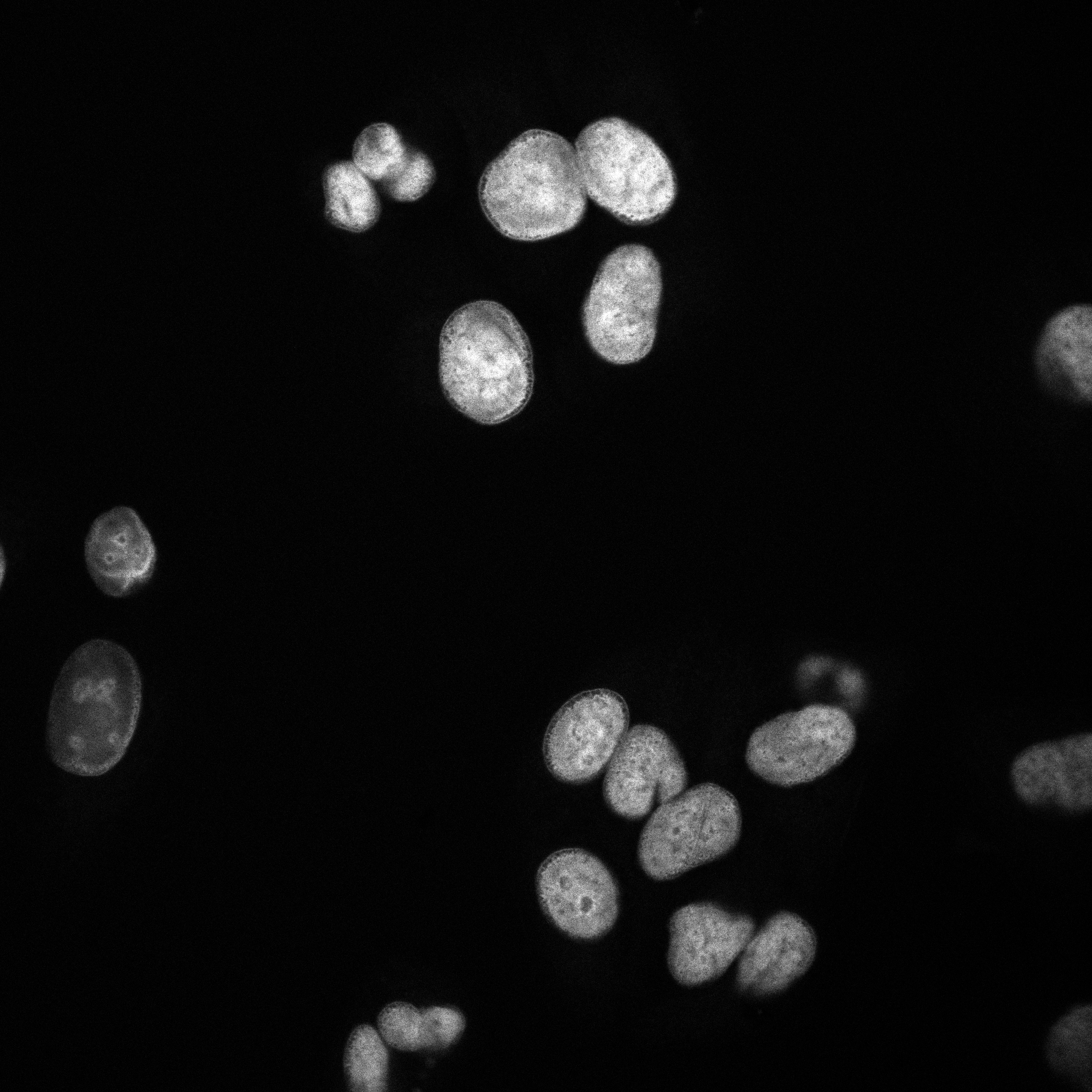

Supplement: Supplementary file 3 — Source data Fig. 3 [file 44318_2026_790_MOESM3_ESM.zip › Figure 3/Figure3D_ADPr_BLMrescue/U2OS_clone_1_siBLM_DAPI.tif]

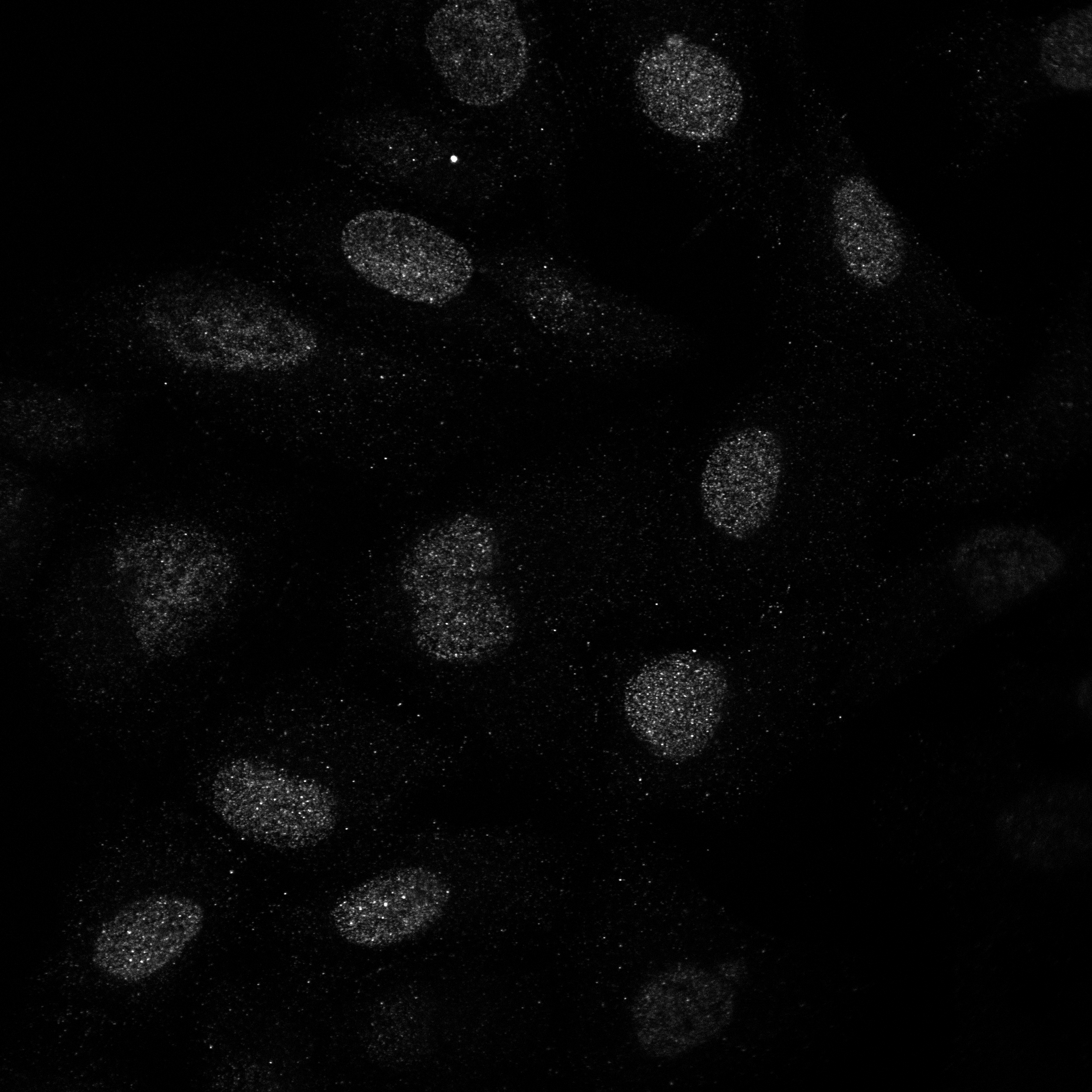

Supplement: Supplementary file 3 — Source data Fig. 3 [file 44318_2026_790_MOESM3_ESM.zip › Figure 3/Figure3D_ADPr_BLMrescue/U2OS_clone_1_siCTRL_ADPr.tif]

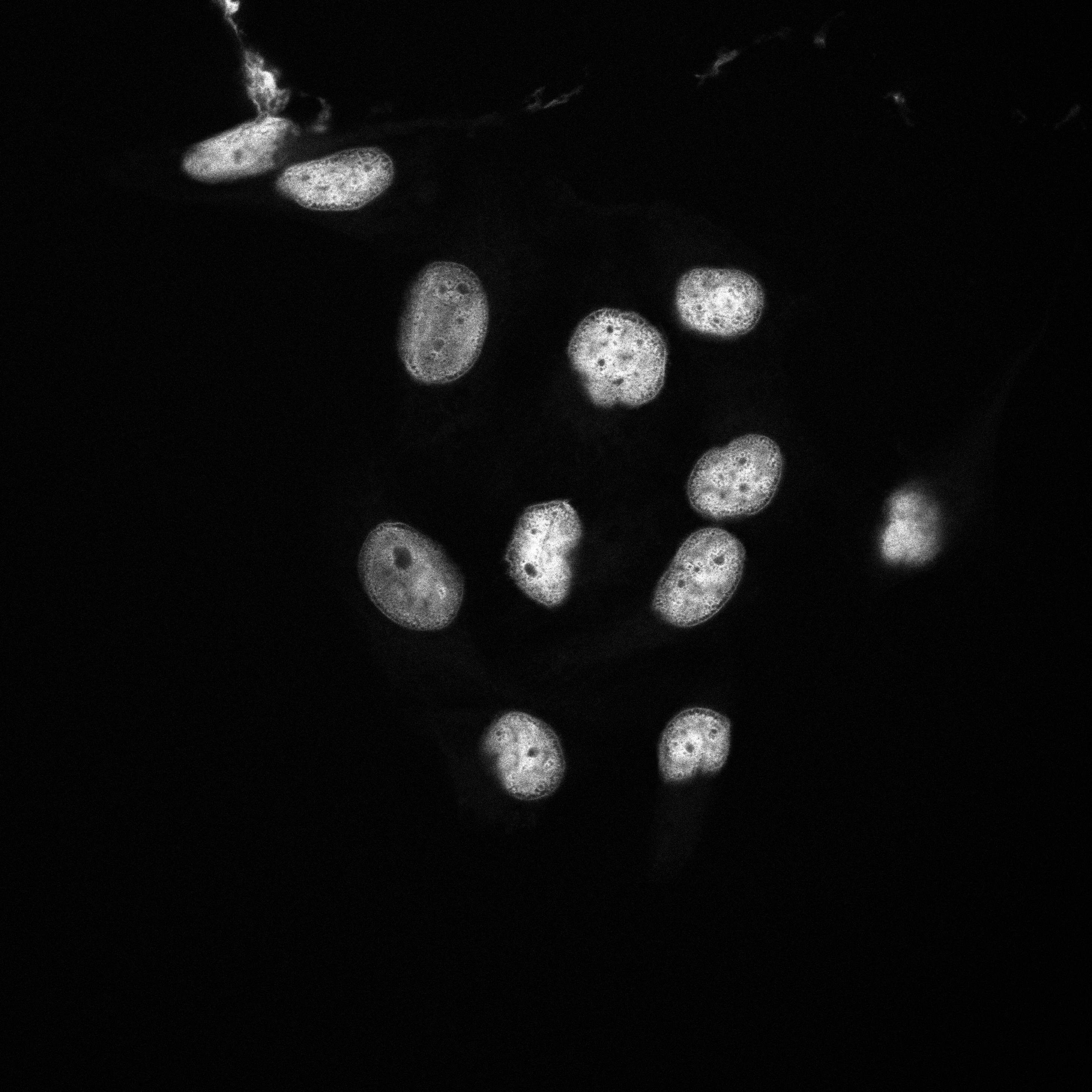

Supplement: Supplementary file 3 — Source data Fig. 3 [file 44318_2026_790_MOESM3_ESM.zip › Figure 3/Figure3D_ADPr_BLMrescue/U2OS_clone_2_siCTRL_DAPI.tif]

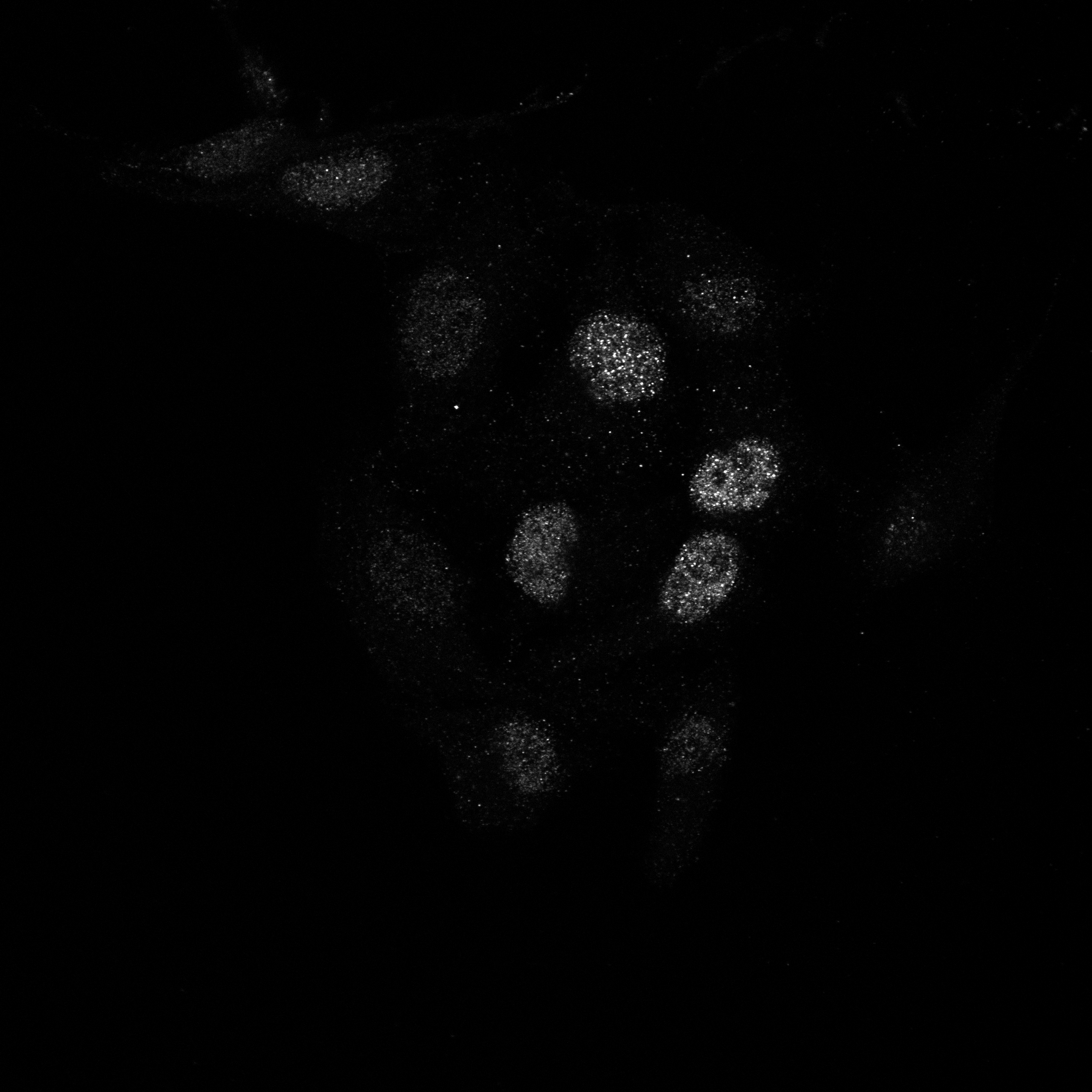

Supplement: Supplementary file 3 — Source data Fig. 3 [file 44318_2026_790_MOESM3_ESM.zip › Figure 3/Figure3D_ADPr_BLMrescue/U2OS_clone_2_siCTRL_ADPr.tif]

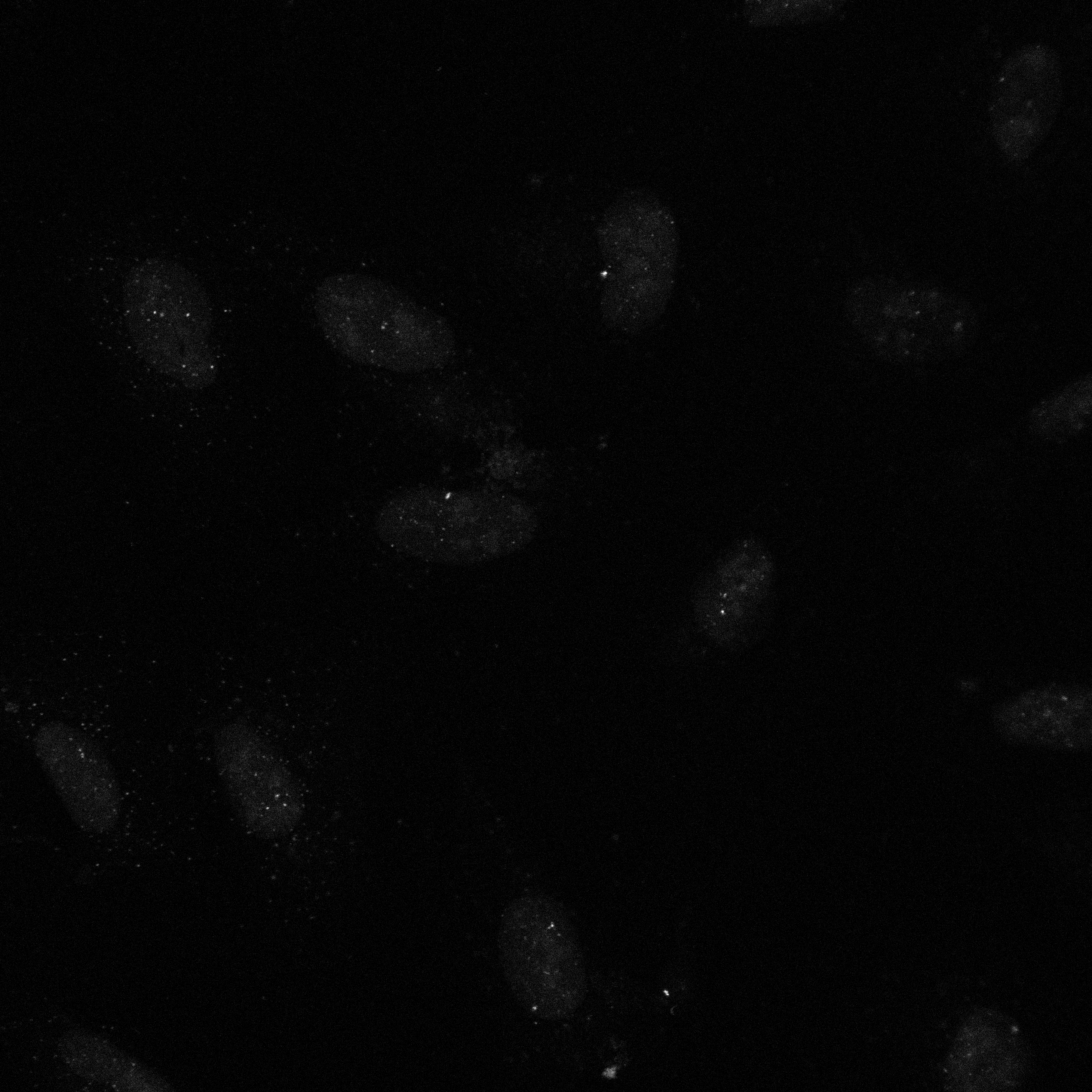

Supplement: Supplementary file 4 — Source data Fig. 4 [file 44318_2026_790_MOESM4_ESM.zip › Figure 4/Figure 4E_pCHK1_TRF2_staining_U2OS/C3-U2OS_WT_pSer345-CHK1.tif]

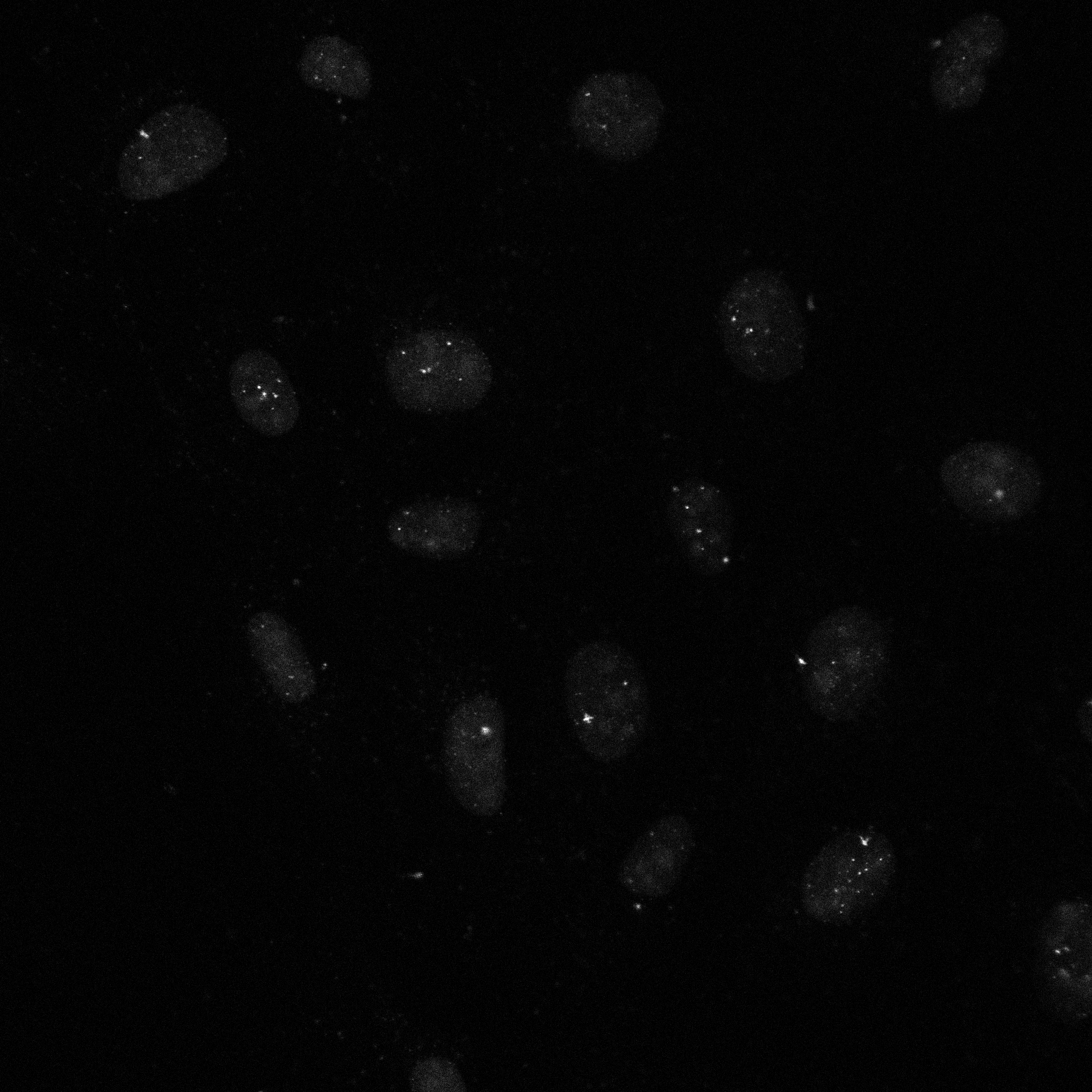

Supplement: Supplementary file 4 — Source data Fig. 4 [file 44318_2026_790_MOESM4_ESM.zip › Figure 4/Figure 4E_pCHK1_TRF2_staining_U2OS/C3-U2OS_SLX4IP_KO_clone_2_pS345-CHK1.tif]

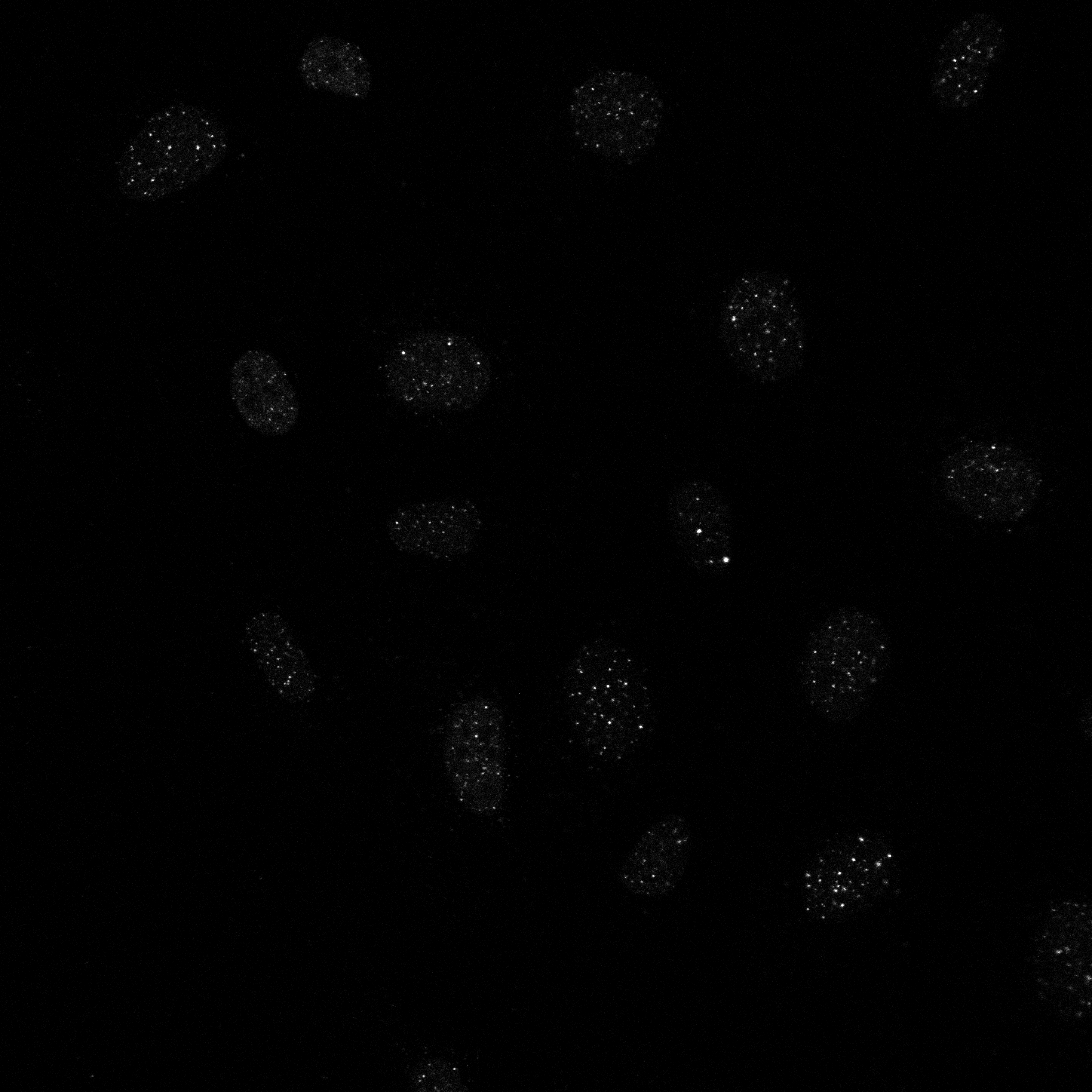

Supplement: Supplementary file 4 — Source data Fig. 4 [file 44318_2026_790_MOESM4_ESM.zip › Figure 4/Figure 4E_pCHK1_TRF2_staining_U2OS/C2-U2OS_SLX4IP_KO_clone_2_TRF2.tif]

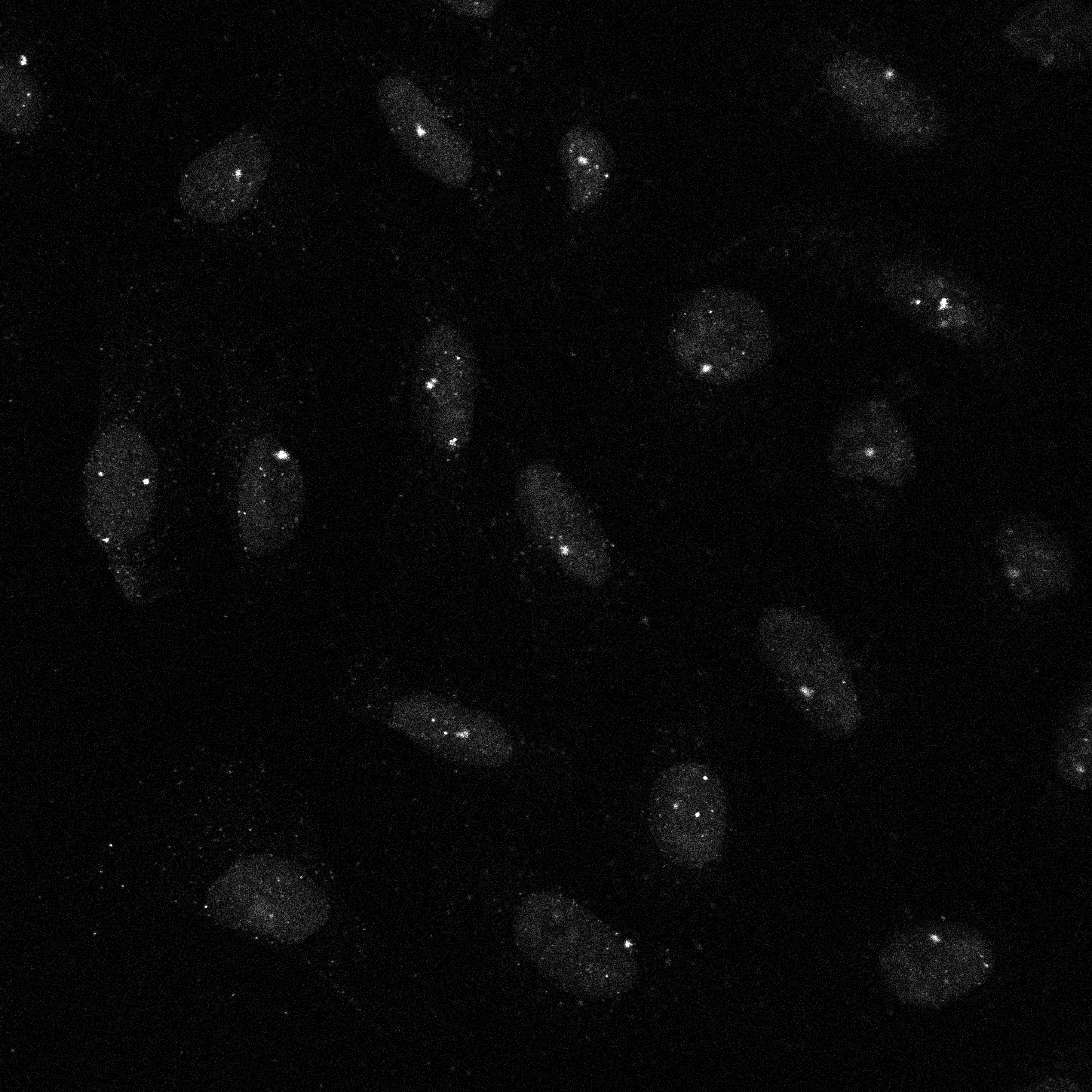

Supplement: Supplementary file 4 — Source data Fig. 4 [file 44318_2026_790_MOESM4_ESM.zip › Figure 4/Figure 4E_pCHK1_TRF2_staining_U2OS/C3-U2OS_SLX4IP_KO_clone_1_pS345-CHK1.tif]

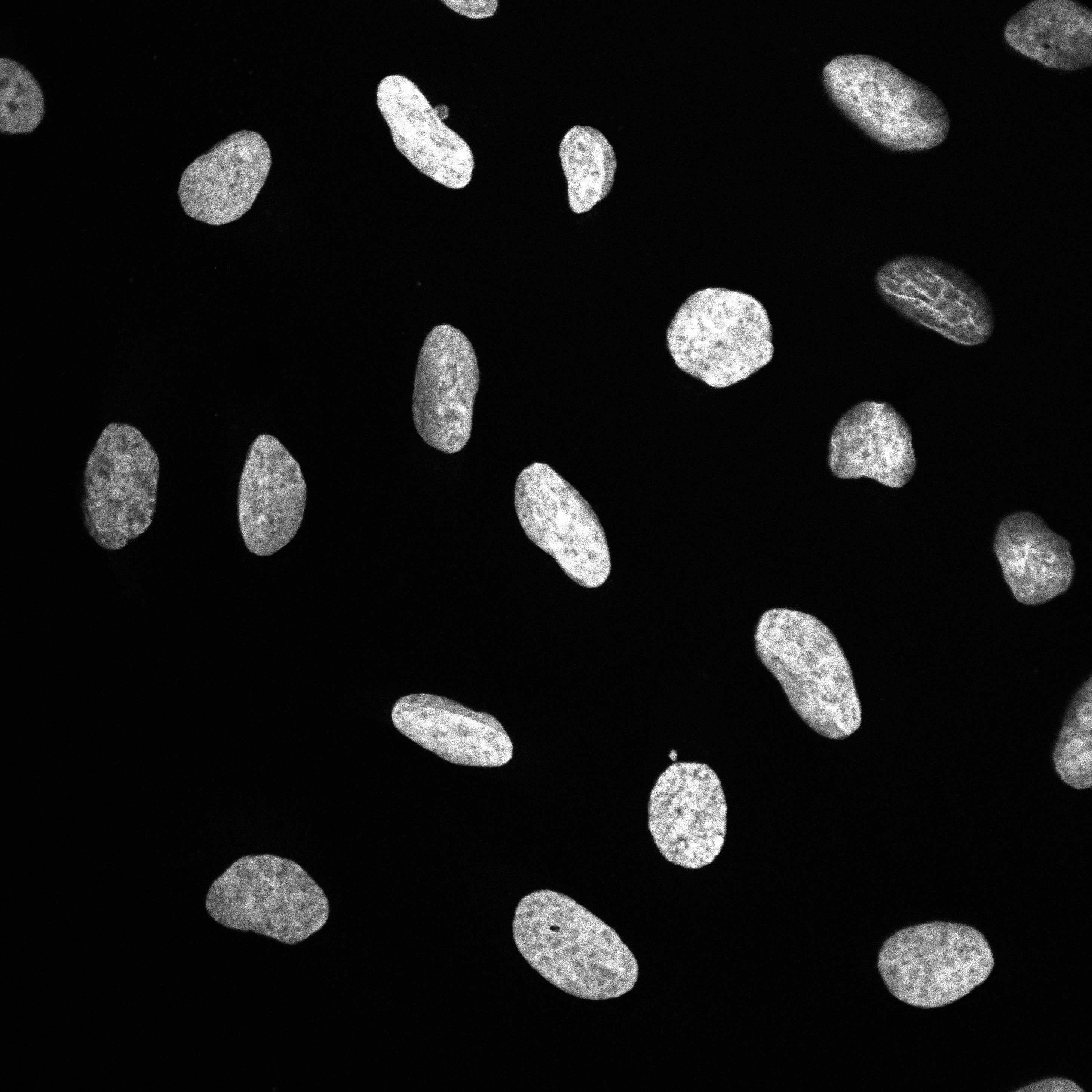

Supplement: Supplementary file 4 — Source data Fig. 4 [file 44318_2026_790_MOESM4_ESM.zip › Figure 4/Figure 4E_pCHK1_TRF2_staining_U2OS/C1-U2OS_SLX4IP_KO_clone_1_DAPI.tif]

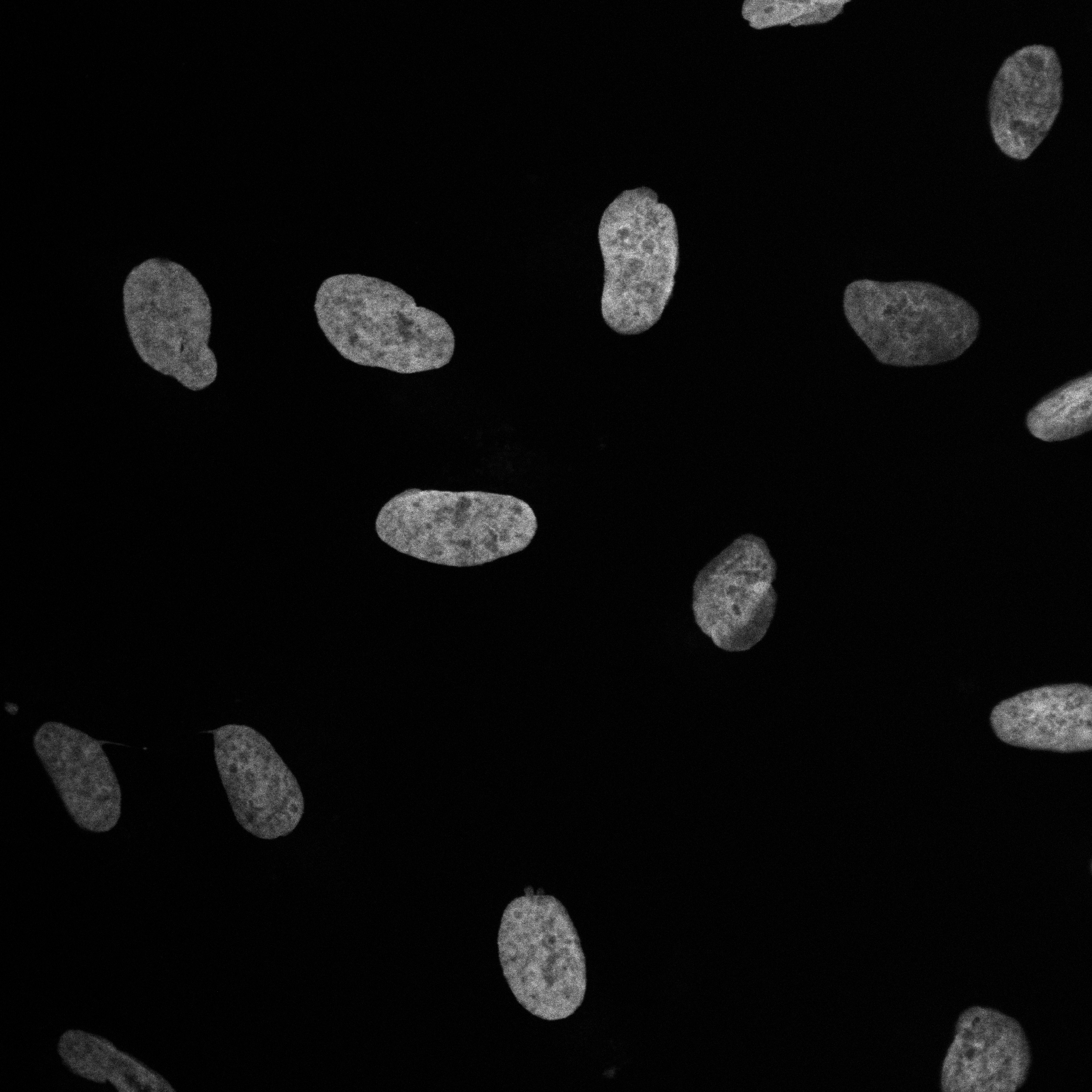

Supplement: Supplementary file 4 — Source data Fig. 4 [file 44318_2026_790_MOESM4_ESM.zip › Figure 4/Figure 4E_pCHK1_TRF2_staining_U2OS/C1-U2OS_WT_DAPI.tif]

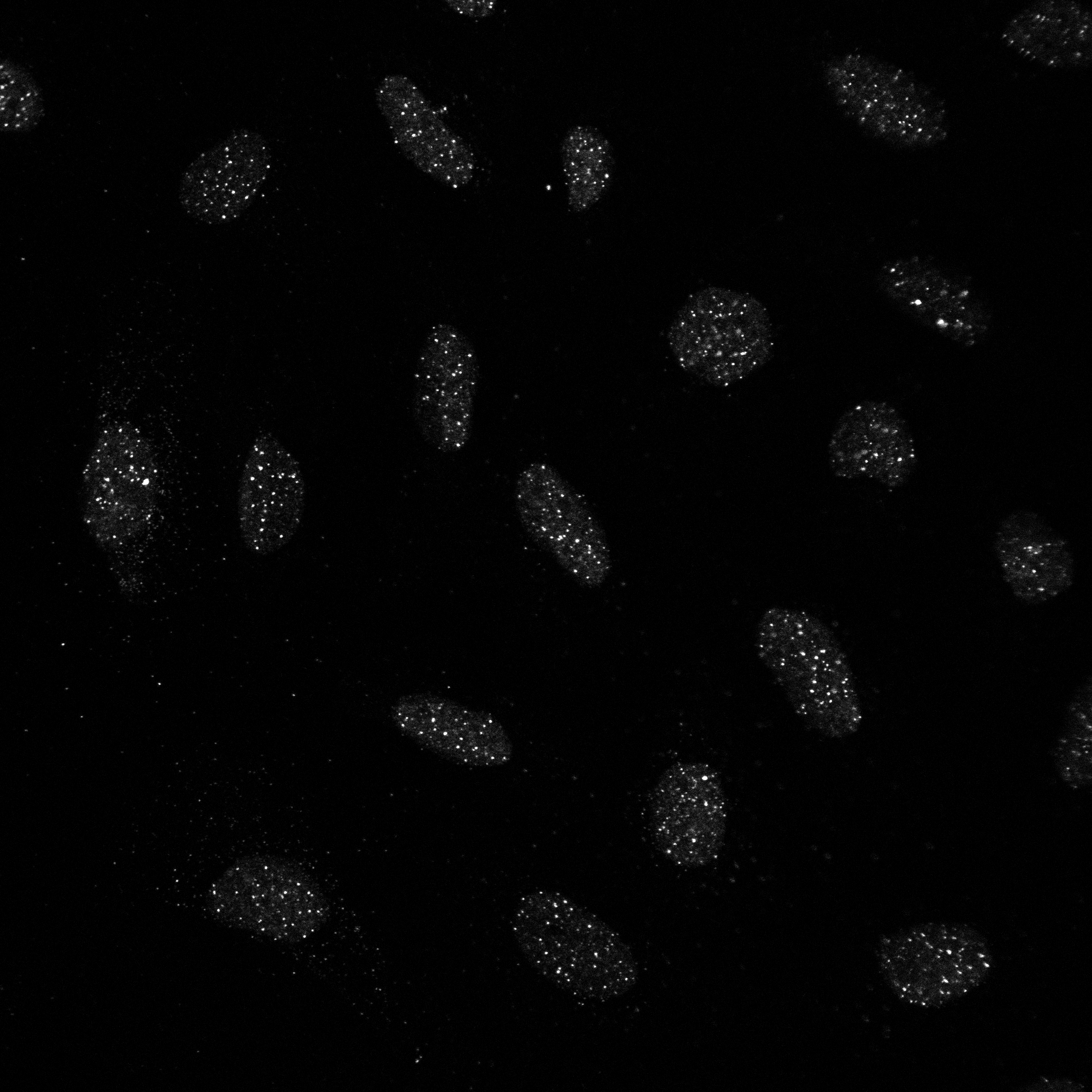

Supplement: Supplementary file 4 — Source data Fig. 4 [file 44318_2026_790_MOESM4_ESM.zip › Figure 4/Figure 4E_pCHK1_TRF2_staining_U2OS/C2-U2OS_SLX4IP_KO_clone_1_TRF2.tif]

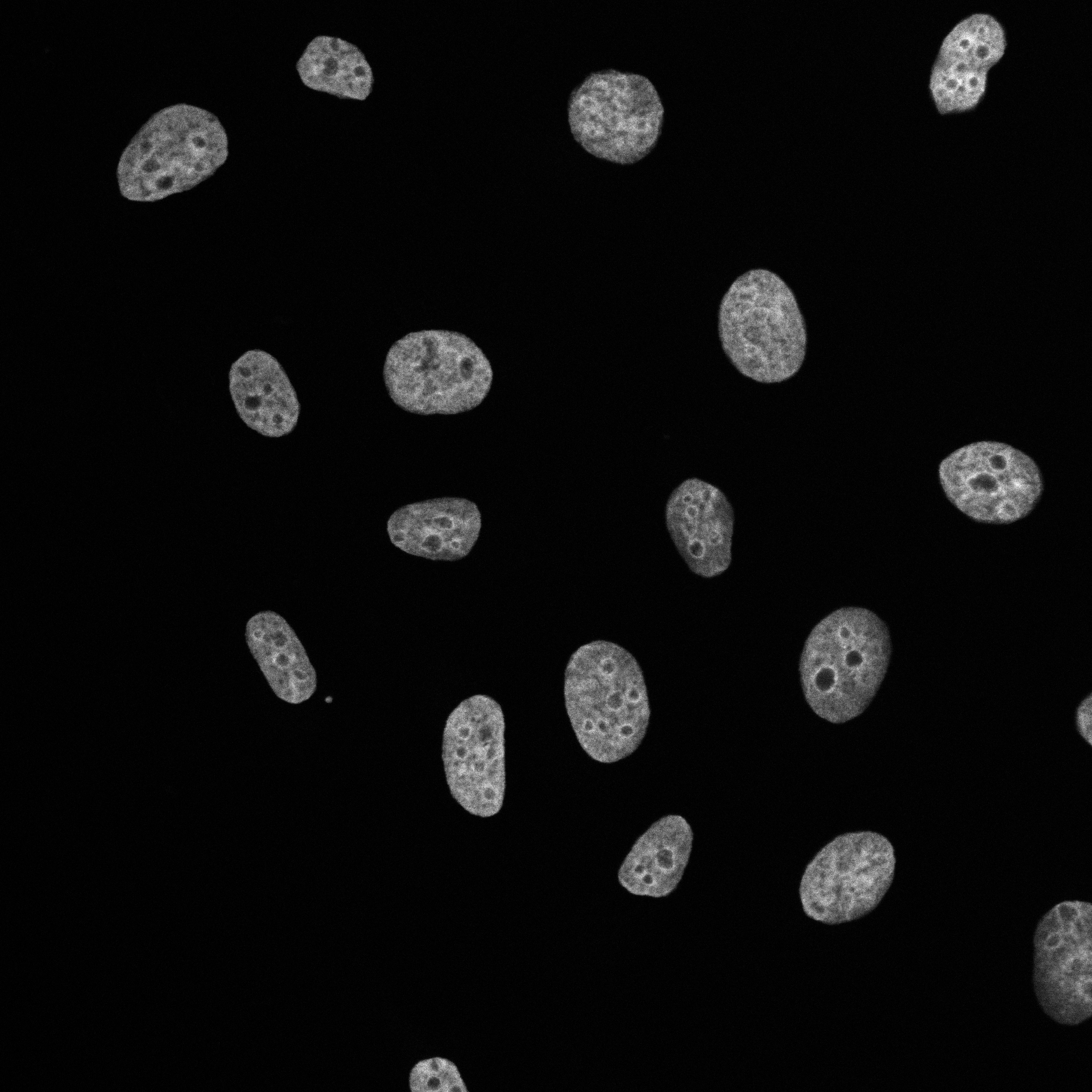

Supplement: Supplementary file 4 — Source data Fig. 4 [file 44318_2026_790_MOESM4_ESM.zip › Figure 4/Figure 4E_pCHK1_TRF2_staining_U2OS/C1-U2OS_SLX4IP_KO_clone_2_DAPI.tif]

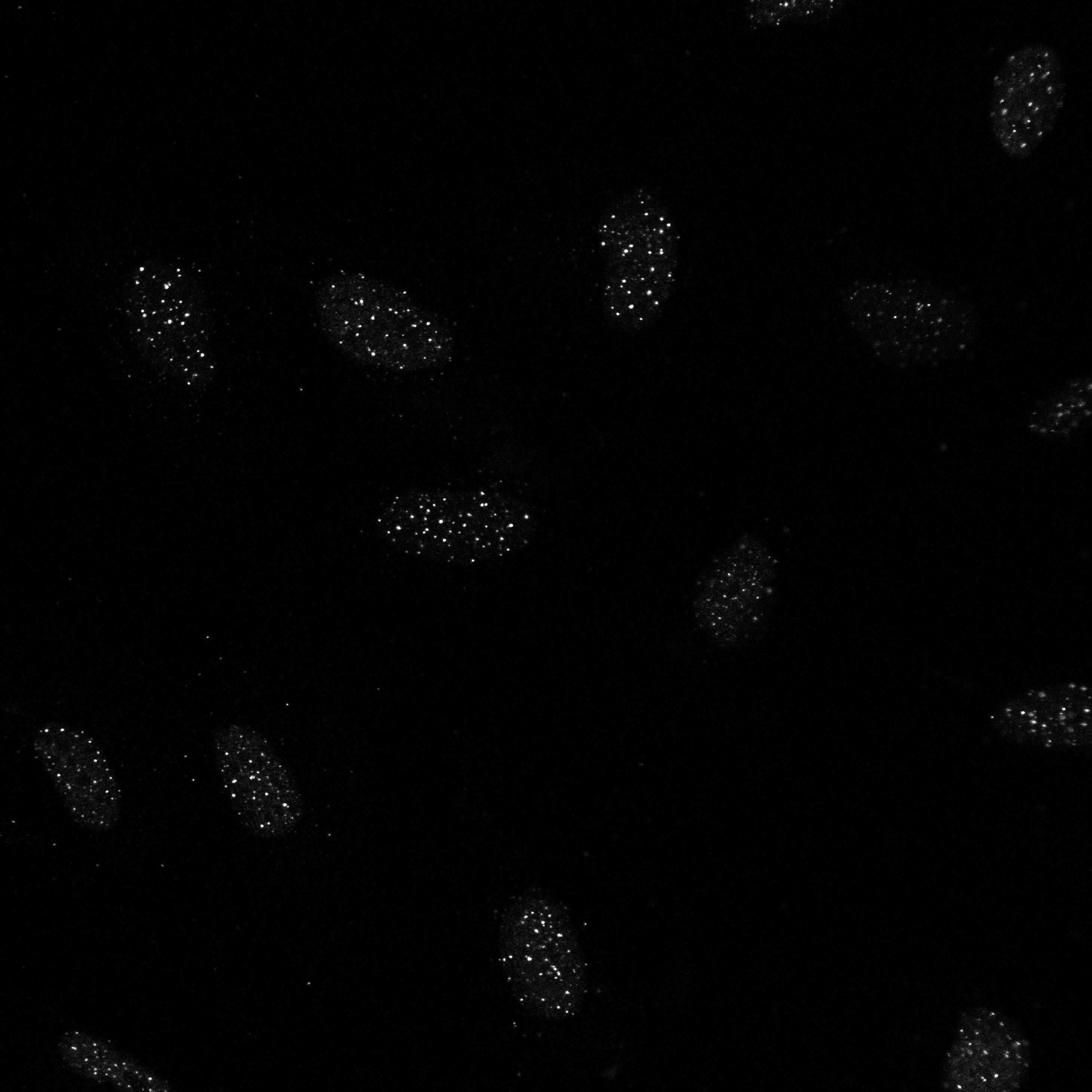

Supplement: Supplementary file 4 — Source data Fig. 4 [file 44318_2026_790_MOESM4_ESM.zip › Figure 4/Figure 4E_pCHK1_TRF2_staining_U2OS/C2-U2OS_WT_TRF2.tif]

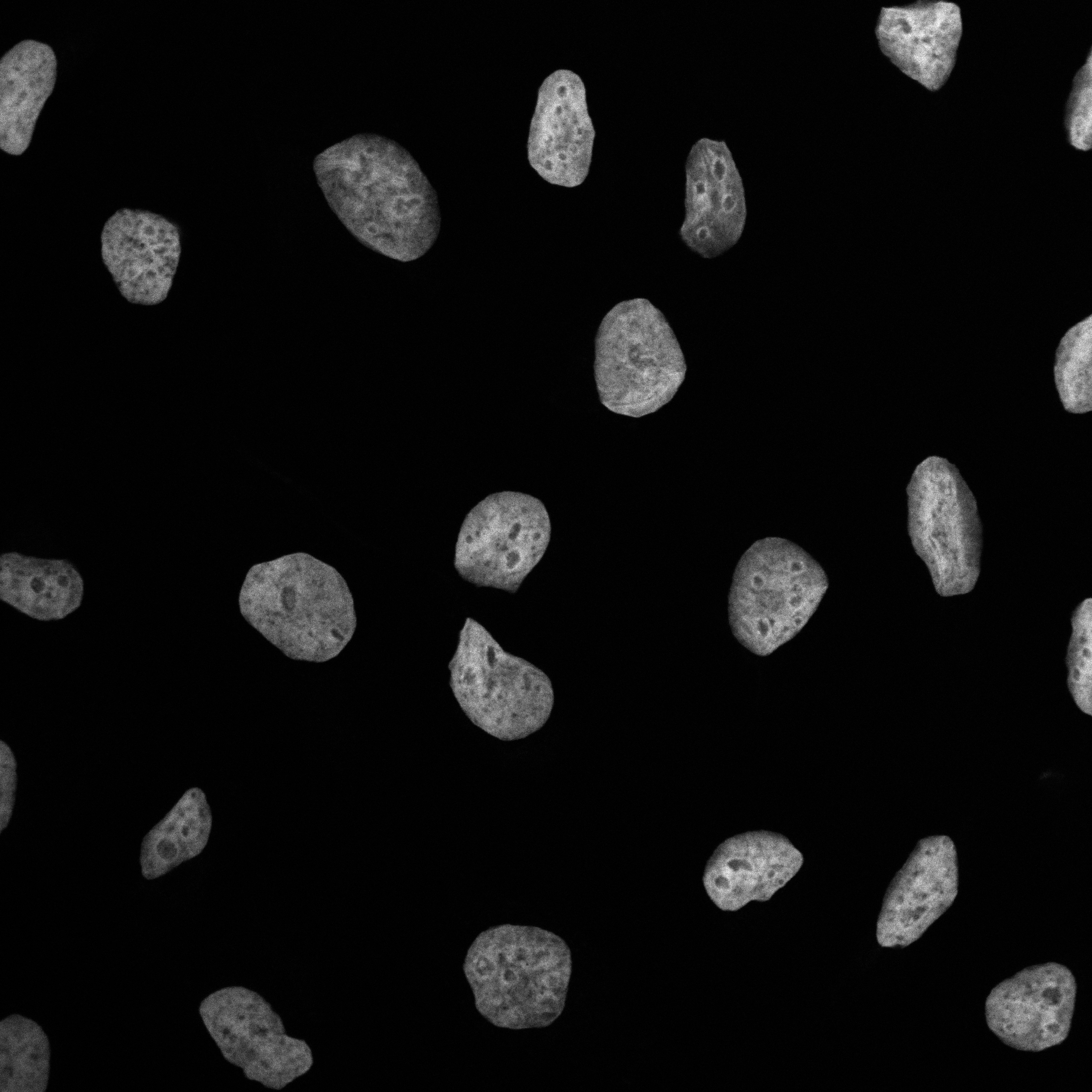

Supplement: Supplementary file 4 — Source data Fig. 4 [file 44318_2026_790_MOESM4_ESM.zip › Figure 4/Figure 4C_pRPA_TRF2_staining_U2OS/1_U2OS_SLX4IP_KO_clone_2_DAPI.tif]

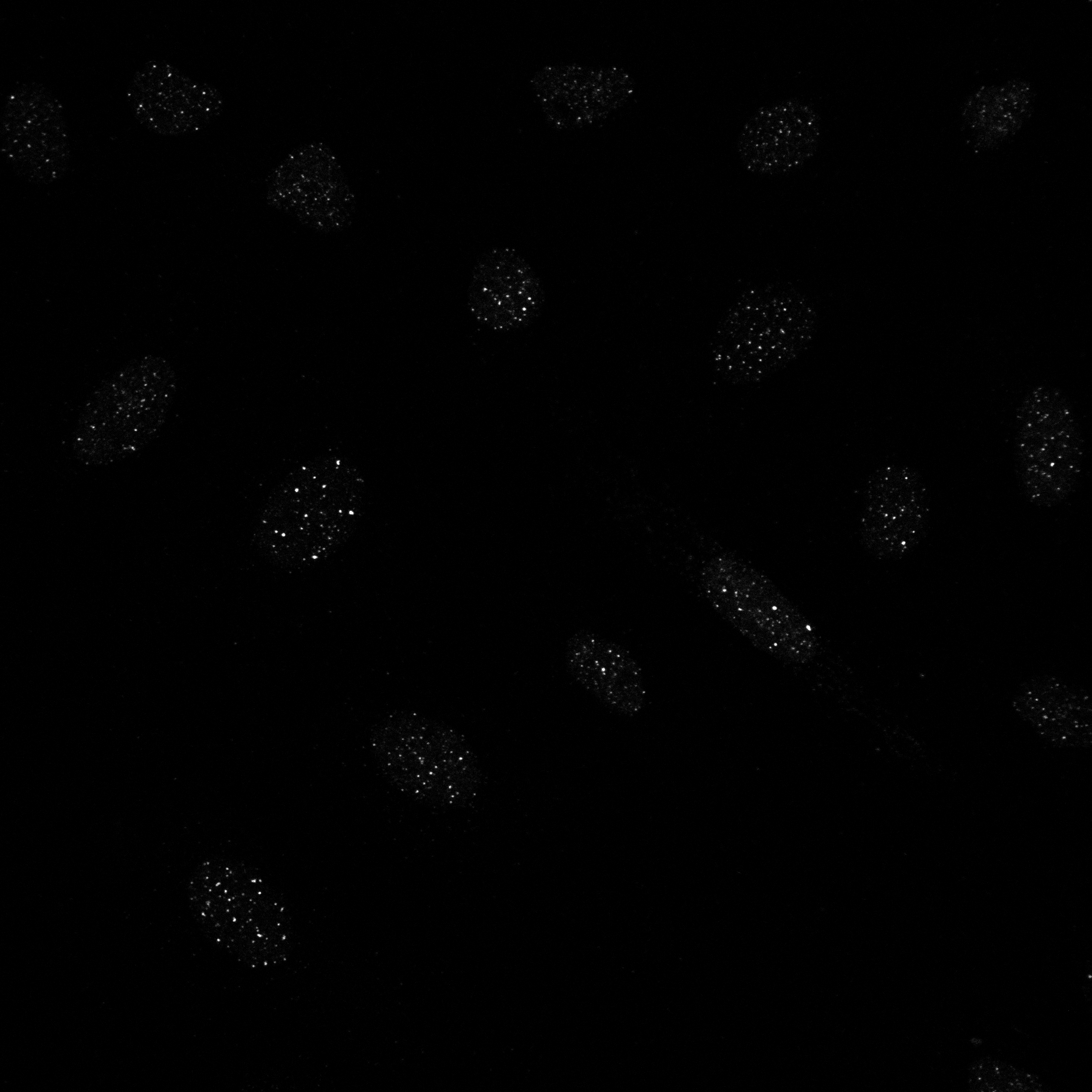

Supplement: Supplementary file 4 — Source data Fig. 4 [file 44318_2026_790_MOESM4_ESM.zip › Figure 4/Figure 4C_pRPA_TRF2_staining_U2OS/1_U2OS_SLX4IP_KO_clone_1_TRF2.tif]

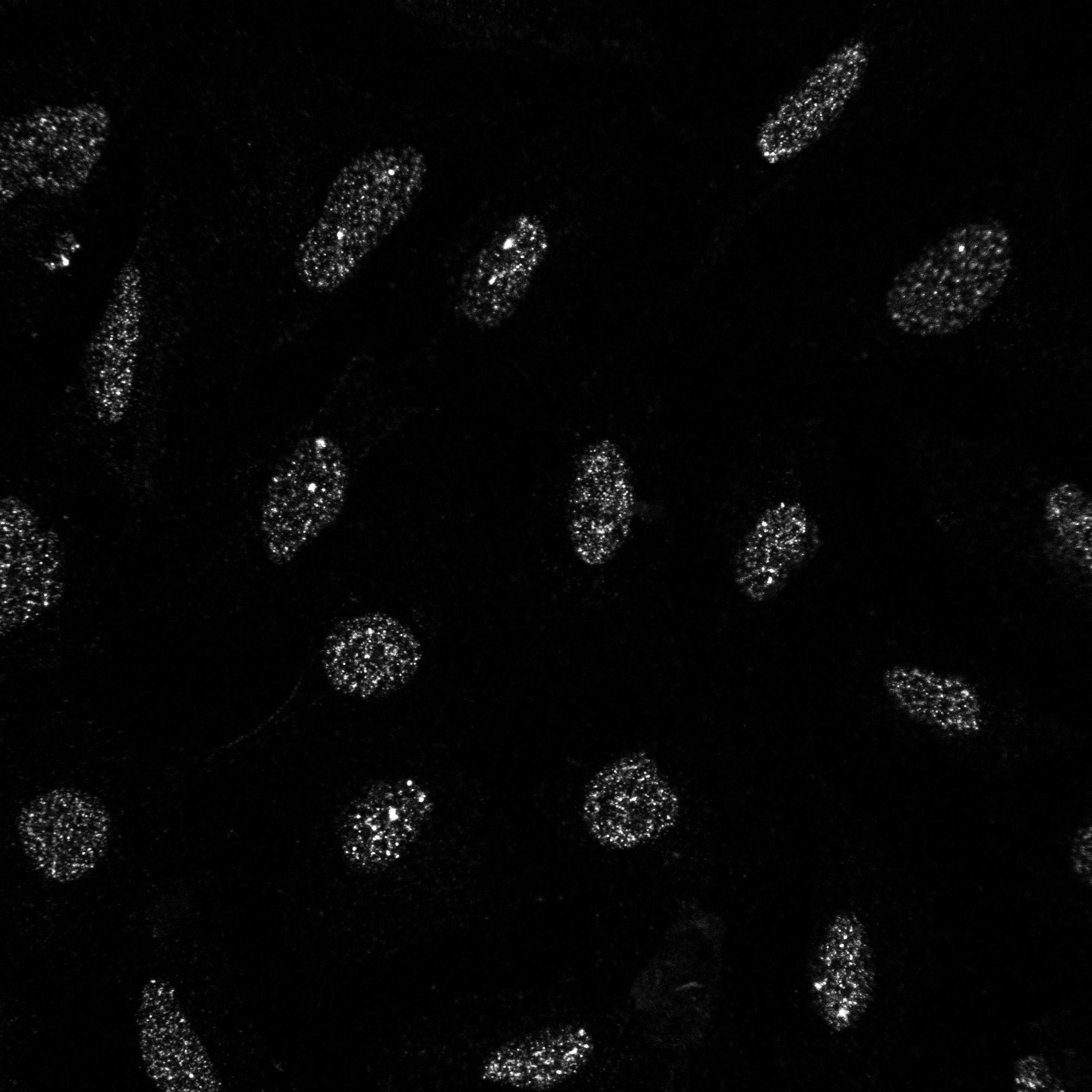

Supplement: Supplementary file 4 — Source data Fig. 4 [file 44318_2026_790_MOESM4_ESM.zip › Figure 4/Figure 4C_pRPA_TRF2_staining_U2OS/1_U2OS_WT_pS33-RPA.tif]

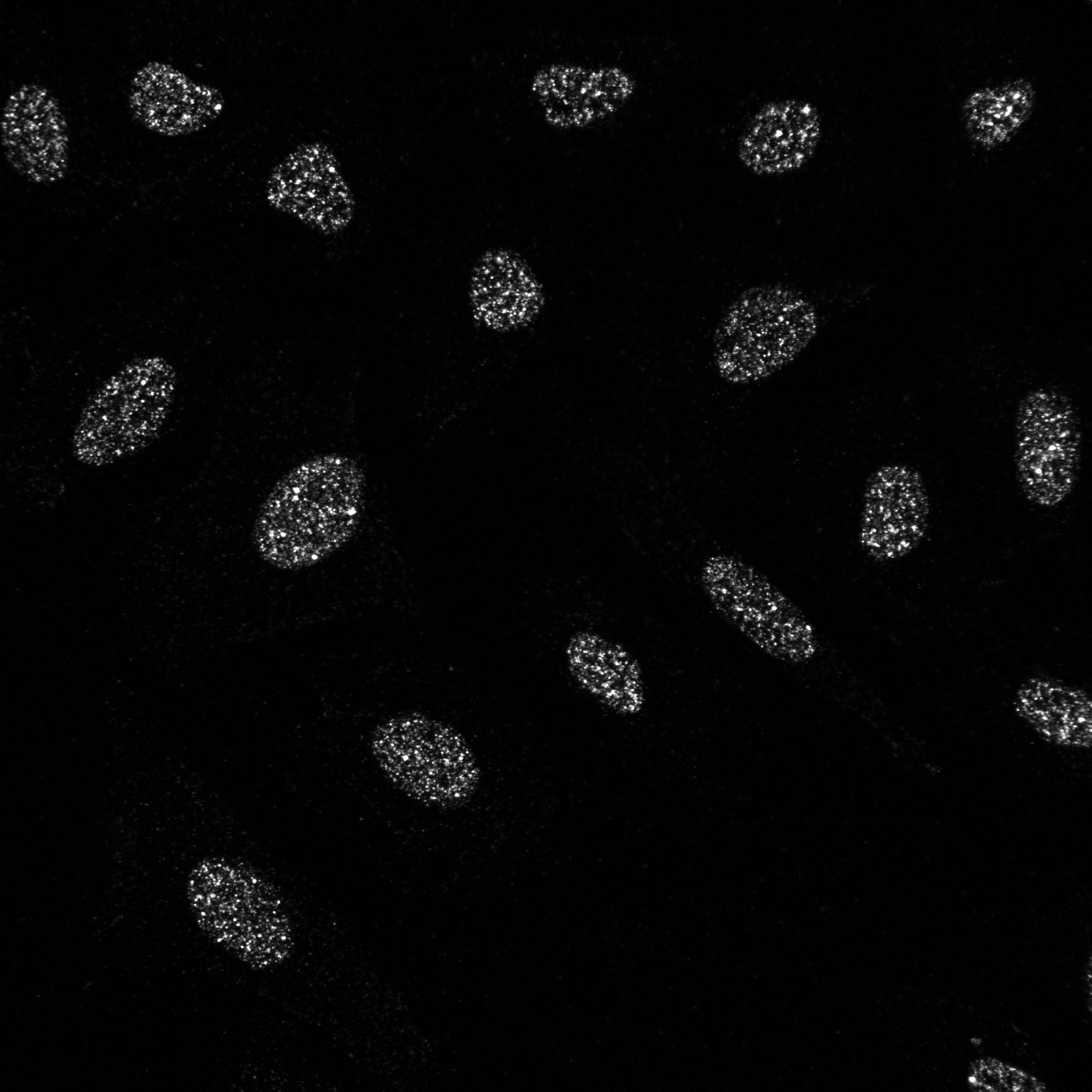

Supplement: Supplementary file 4 — Source data Fig. 4 [file 44318_2026_790_MOESM4_ESM.zip › Figure 4/Figure 4C_pRPA_TRF2_staining_U2OS/1_U2OS_SLX4IP_KO_clone_1_pS33-RPA.tif]

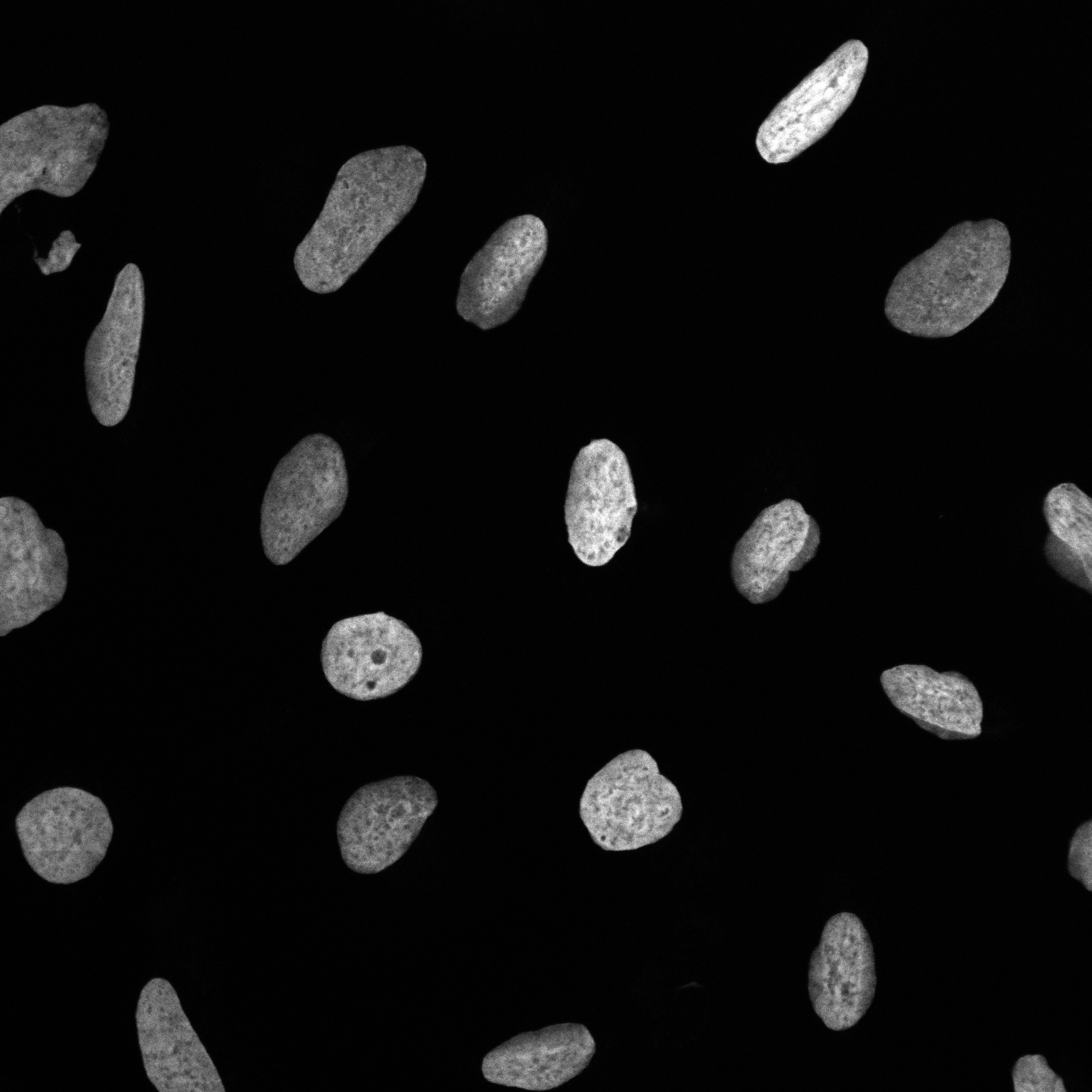

Supplement: Supplementary file 4 — Source data Fig. 4 [file 44318_2026_790_MOESM4_ESM.zip › Figure 4/Figure 4C_pRPA_TRF2_staining_U2OS/1_U2OS_WT_DAPI.tif]

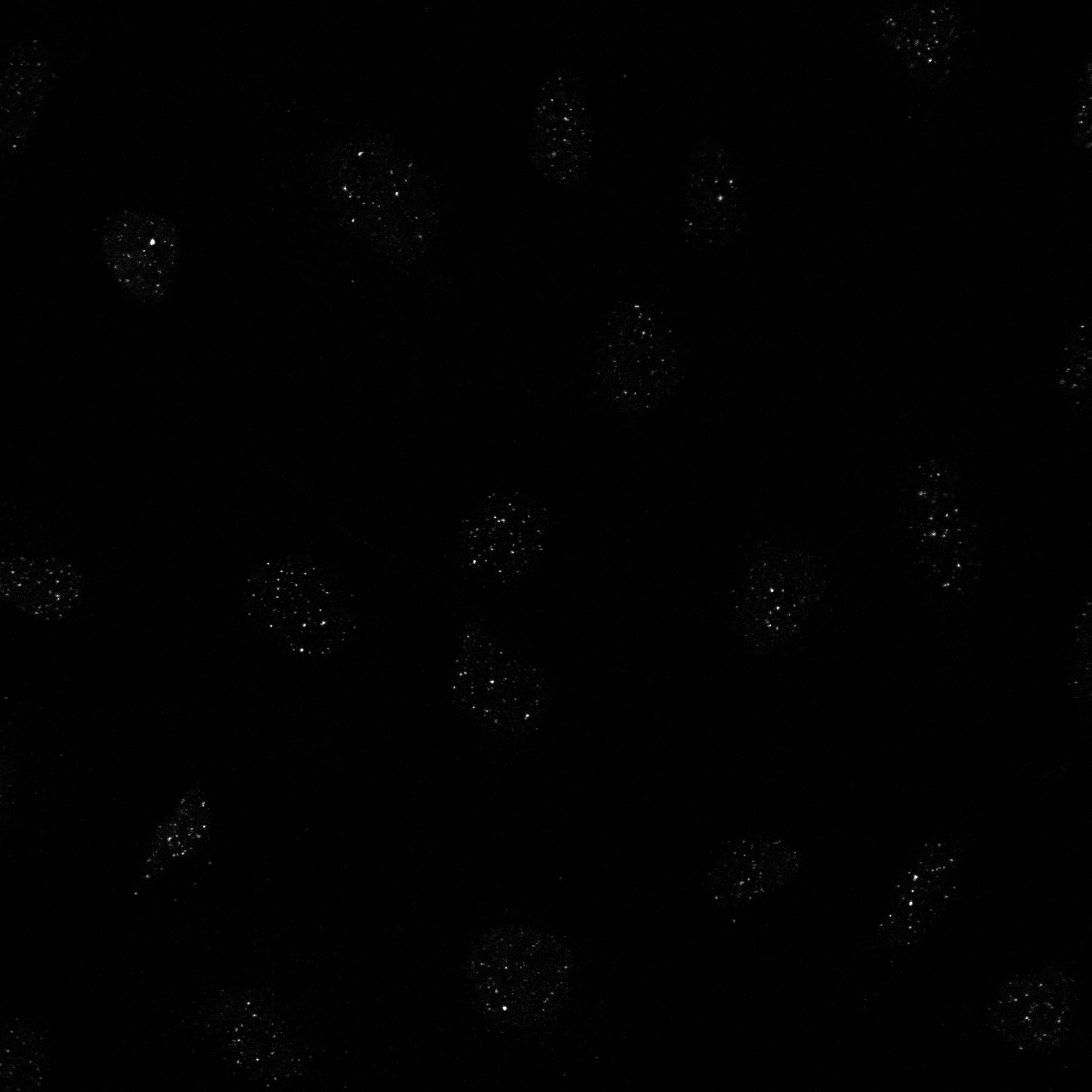

Supplement: Supplementary file 4 — Source data Fig. 4 [file 44318_2026_790_MOESM4_ESM.zip › Figure 4/Figure 4C_pRPA_TRF2_staining_U2OS/1_U2OS_SLX4IP_KO_clone_2_TRF2.tif]

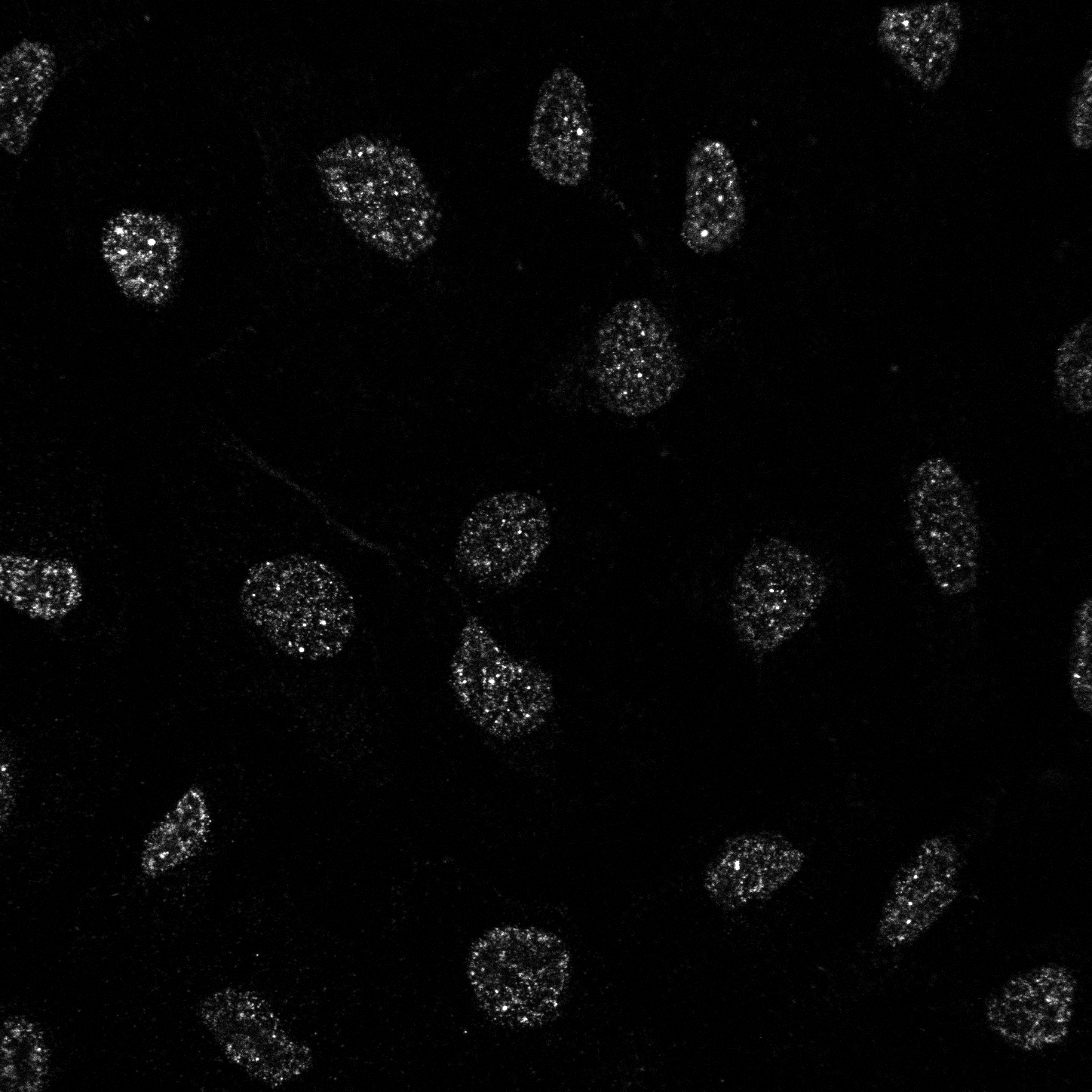

Supplement: Supplementary file 4 — Source data Fig. 4 [file 44318_2026_790_MOESM4_ESM.zip › Figure 4/Figure 4C_pRPA_TRF2_staining_U2OS/1_U2OS_SLX4IP_KO_clone_2_pS33-RPA.tif]

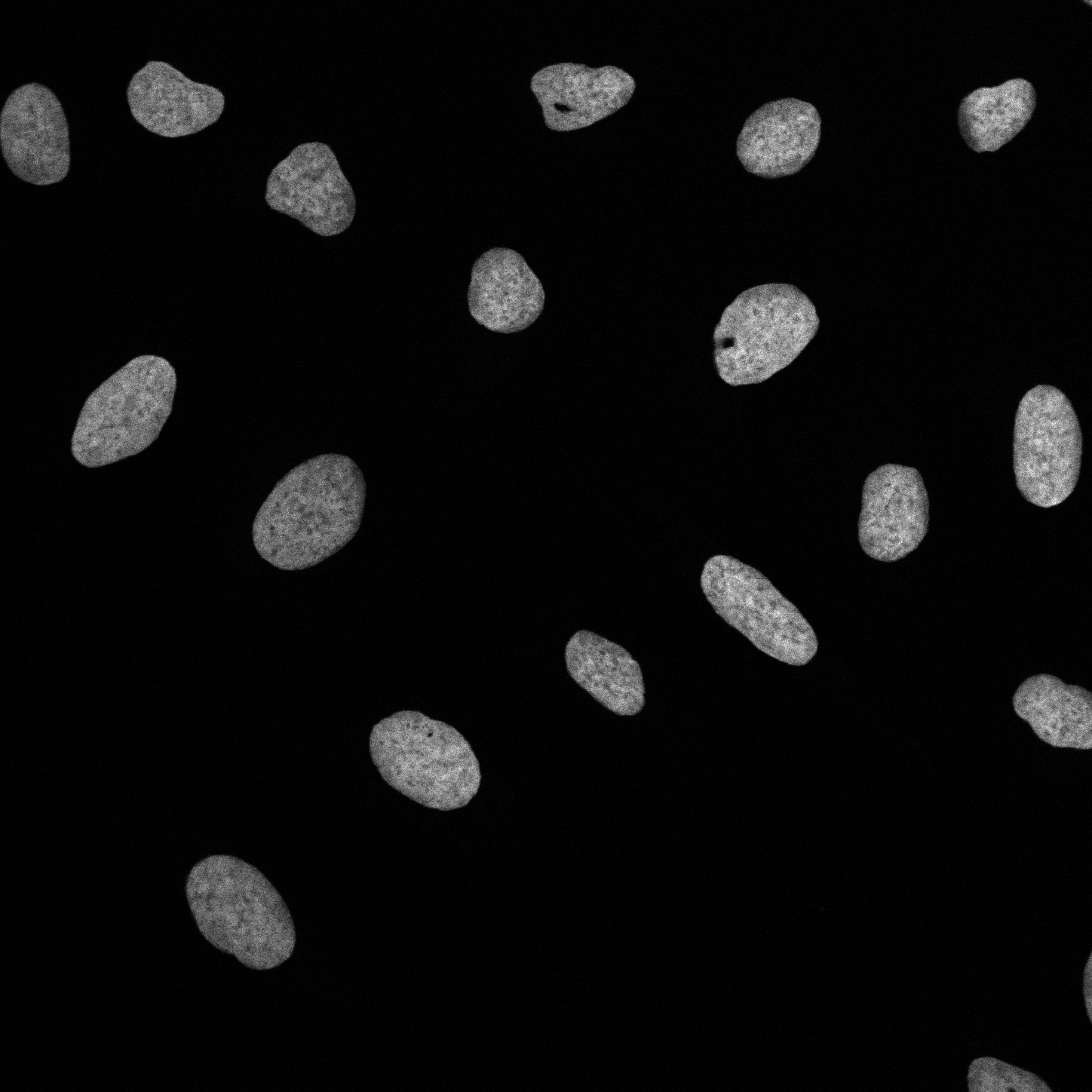

Supplement: Supplementary file 4 — Source data Fig. 4 [file 44318_2026_790_MOESM4_ESM.zip › Figure 4/Figure 4C_pRPA_TRF2_staining_U2OS/1_U2OS_SLX4IP_KO_clone_1_DAPI.tif]

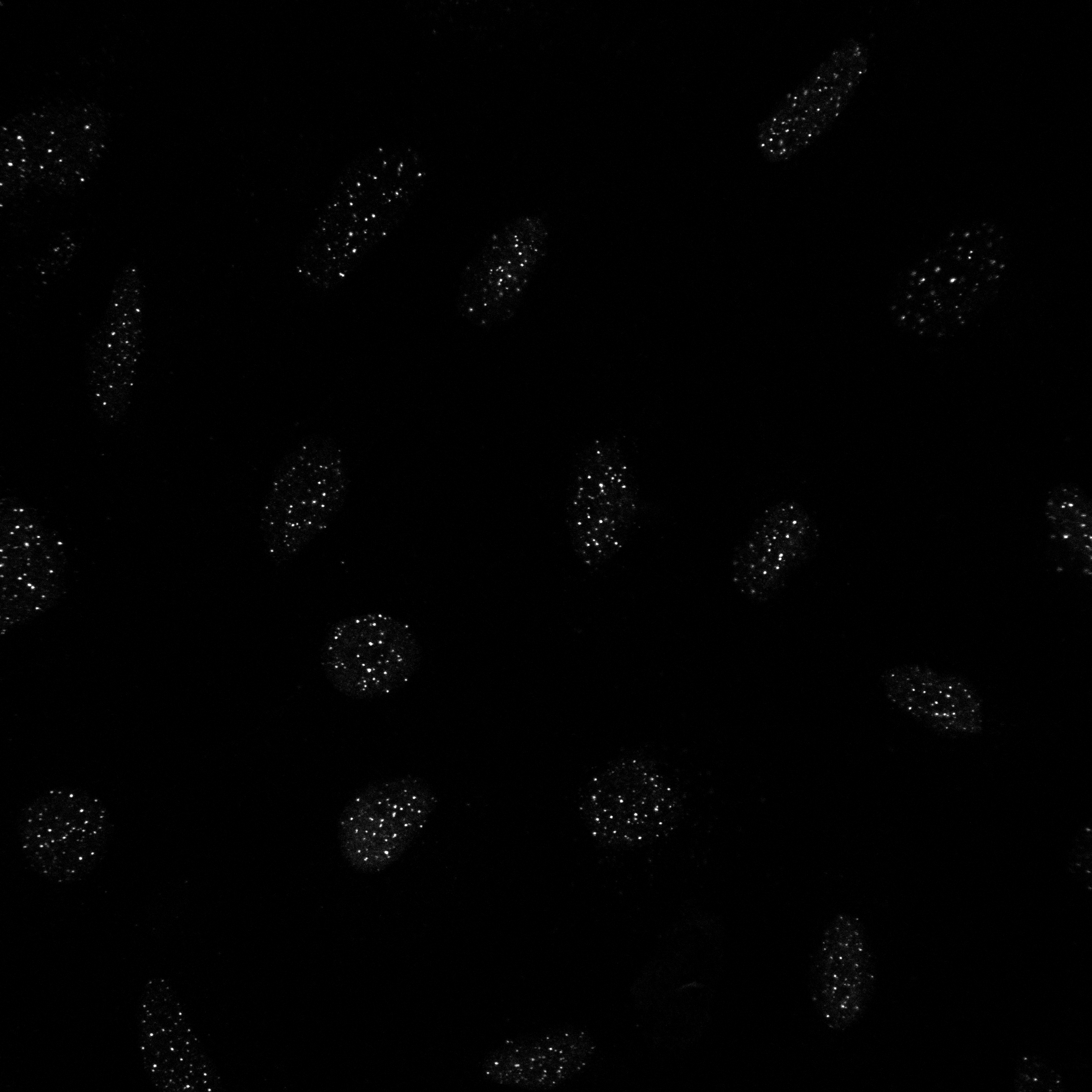

Supplement: Supplementary file 4 — Source data Fig. 4 [file 44318_2026_790_MOESM4_ESM.zip › Figure 4/Figure 4C_pRPA_TRF2_staining_U2OS/1_U2OS_WT_TRF2.tif]

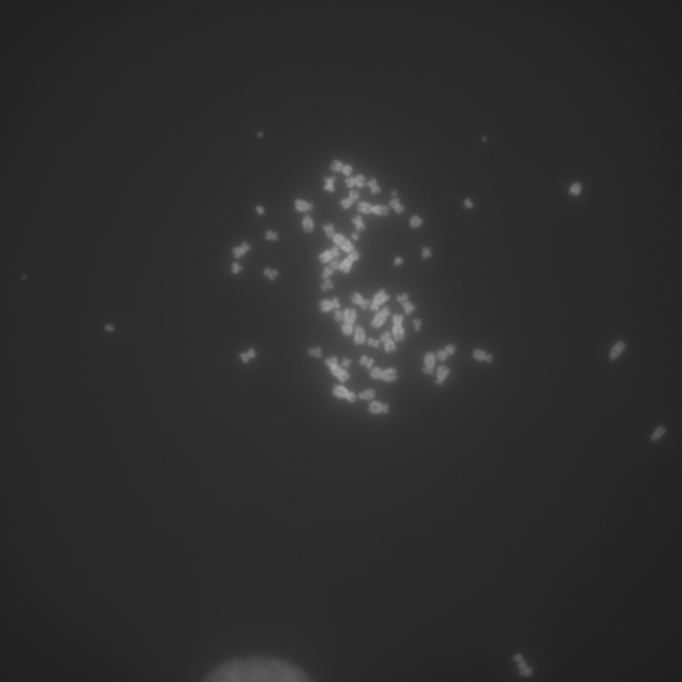

Supplement: Supplementary file 4 — Source data Fig. 4 [file 44318_2026_790_MOESM4_ESM.zip › Figure 4/Figure 4A_U2OS fragility/U2OS_repeat 2_251110_U2OS_2_6_09_RAW_ch00.tif]

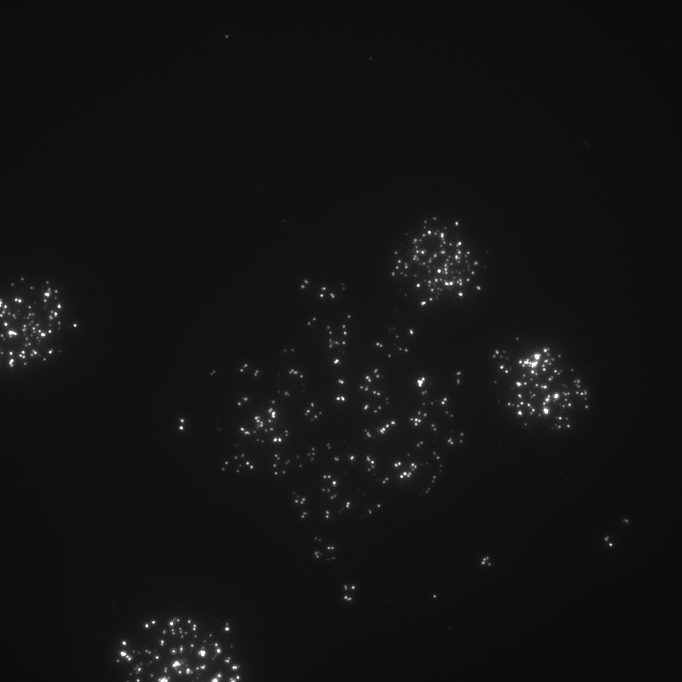

Supplement: Supplementary file 4 — Source data Fig. 4 [file 44318_2026_790_MOESM4_ESM.zip › Figure 4/Figure 4A_U2OS fragility/U2OS_repeat 2_251110_U2OS_2_28_26_RAW_ch02.tif]

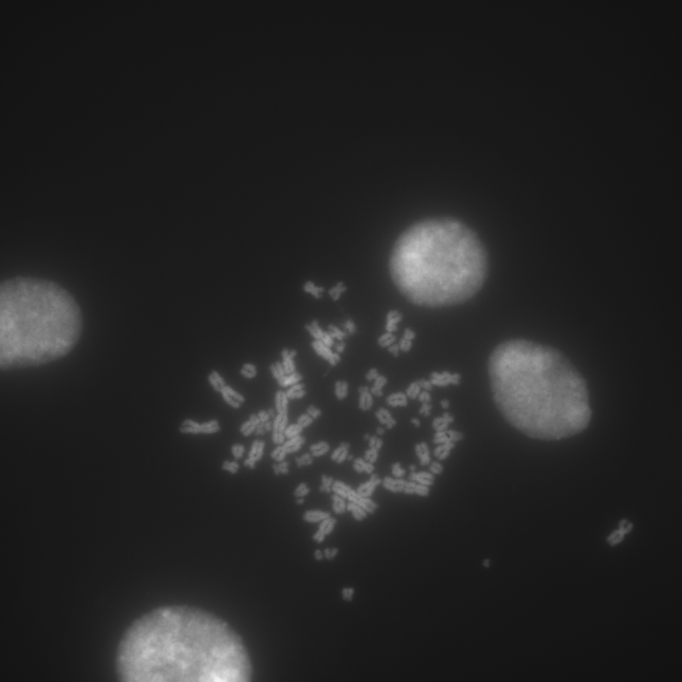

Supplement: Supplementary file 4 — Source data Fig. 4 [file 44318_2026_790_MOESM4_ESM.zip › Figure 4/Figure 4A_U2OS fragility/U2OS_repeat 2_251110_U2OS_2_28_26_RAW_ch00.tif]

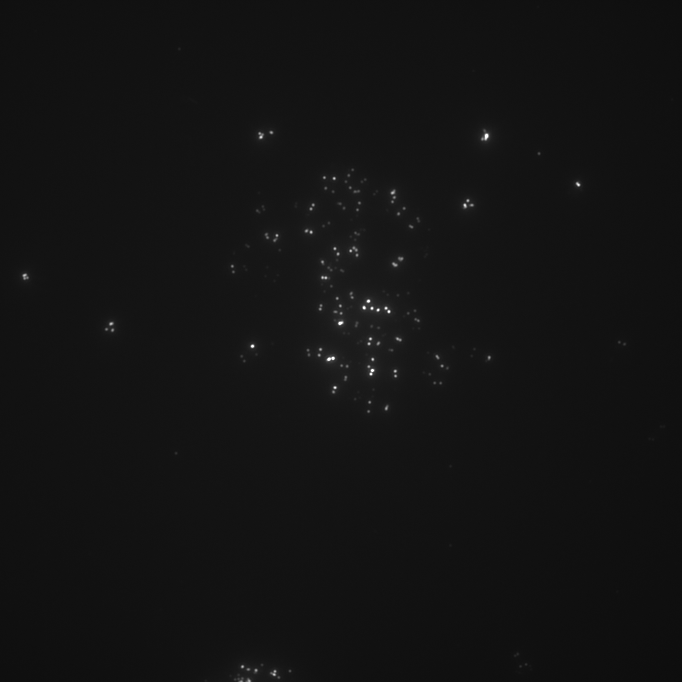

Supplement: Supplementary file 4 — Source data Fig. 4 [file 44318_2026_790_MOESM4_ESM.zip › Figure 4/Figure 4A_U2OS fragility/U2OS_repeat 2_251110_U2OS_2_6_09_RAW_ch02.tif]

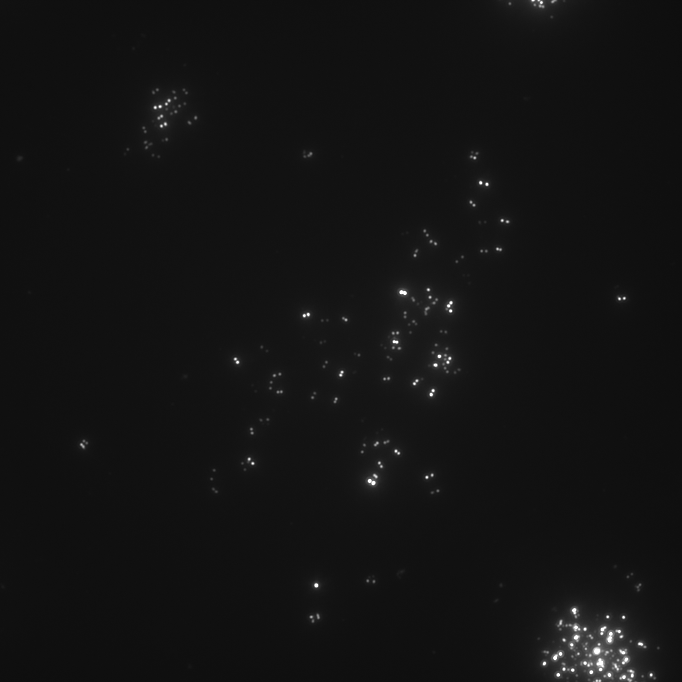

Supplement: Supplementary file 4 — Source data Fig. 4 [file 44318_2026_790_MOESM4_ESM.zip › Figure 4/Figure 4A_U2OS fragility/U2OS_repeat 2_251110_U2OS_2_WT_26_RAW_ch02.tif]

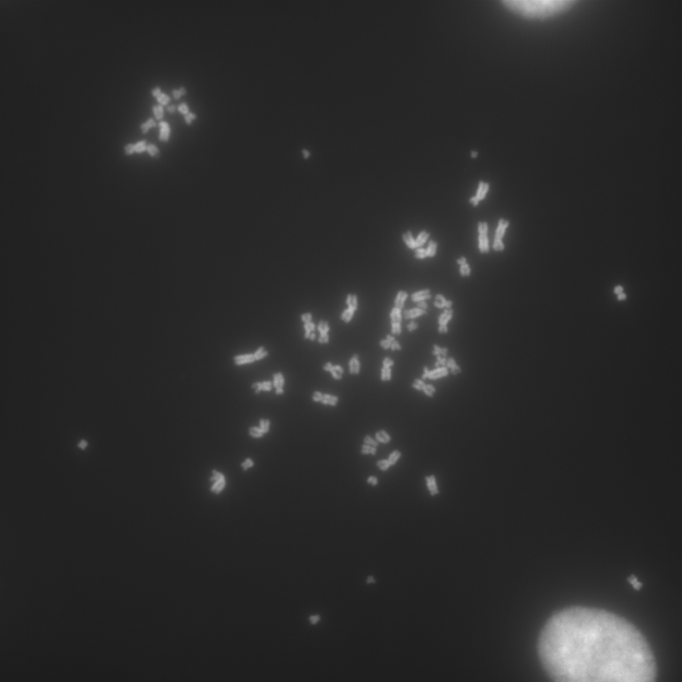

Supplement: Supplementary file 4 — Source data Fig. 4 [file 44318_2026_790_MOESM4_ESM.zip › Figure 4/Figure 4A_U2OS fragility/U2OS_repeat 2_251110_U2OS_2_WT_26_RAW_ch00.tif]

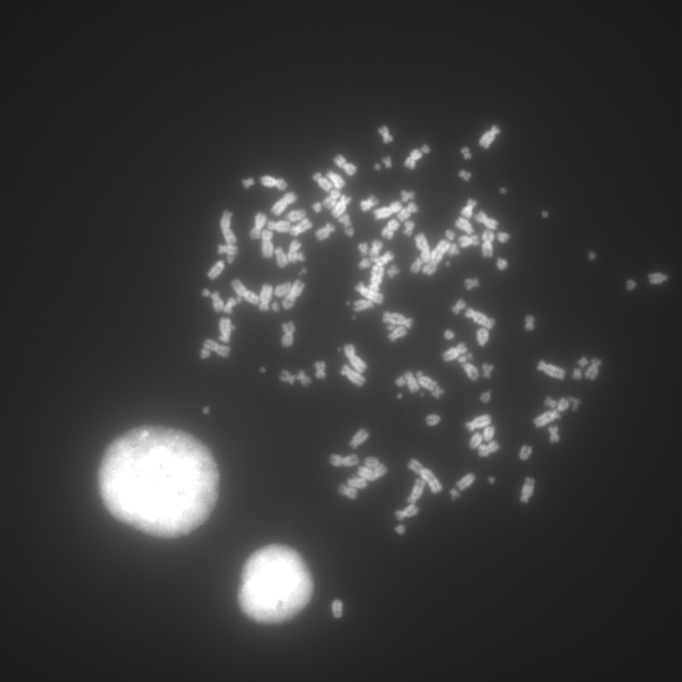

Supplement: Supplementary file 4 — Source data Fig. 4 [file 44318_2026_790_MOESM4_ESM.zip › Figure 4/Figure 4A_U2OS fragility/U2OS_repeat 2_251110_U2OS_2_26_23_RAW_ch00.tif]

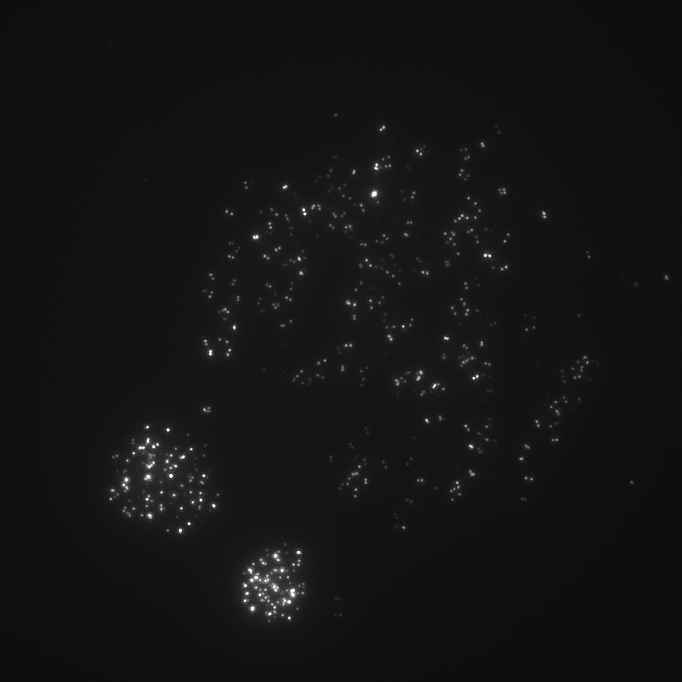

Supplement: Supplementary file 4 — Source data Fig. 4 [file 44318_2026_790_MOESM4_ESM.zip › Figure 4/Figure 4A_U2OS fragility/U2OS_repeat 2_251110_U2OS_2_26_23_RAW_ch02.tif]

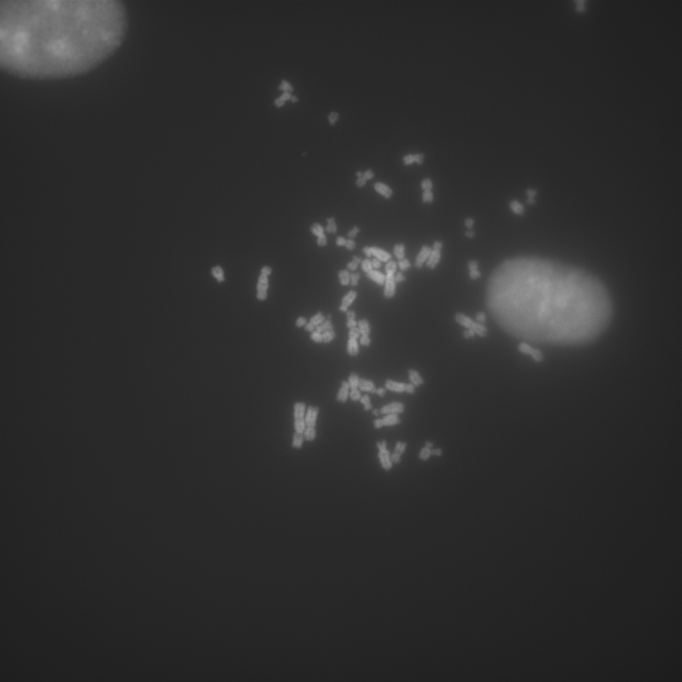

Supplement: Supplementary file 4 — Source data Fig. 4 [file 44318_2026_790_MOESM4_ESM.zip › Figure 4/Figure 4A_U2OS fragility/U2OS_repeat 2_251110_U2OS_2_15_14_RAW_ch00.tif]

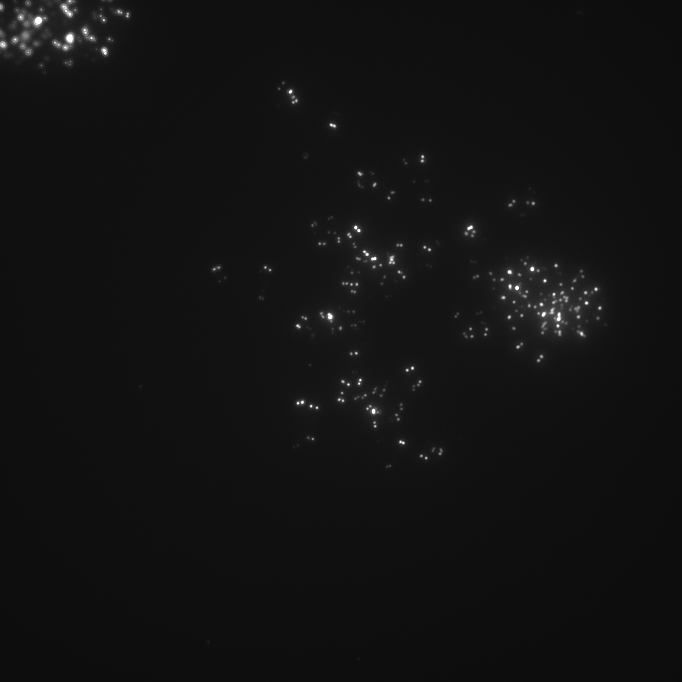

Supplement: Supplementary file 4 — Source data Fig. 4 [file 44318_2026_790_MOESM4_ESM.zip › Figure 4/Figure 4A_U2OS fragility/U2OS_repeat 2_251110_U2OS_2_15_14_RAW_ch02.tif]

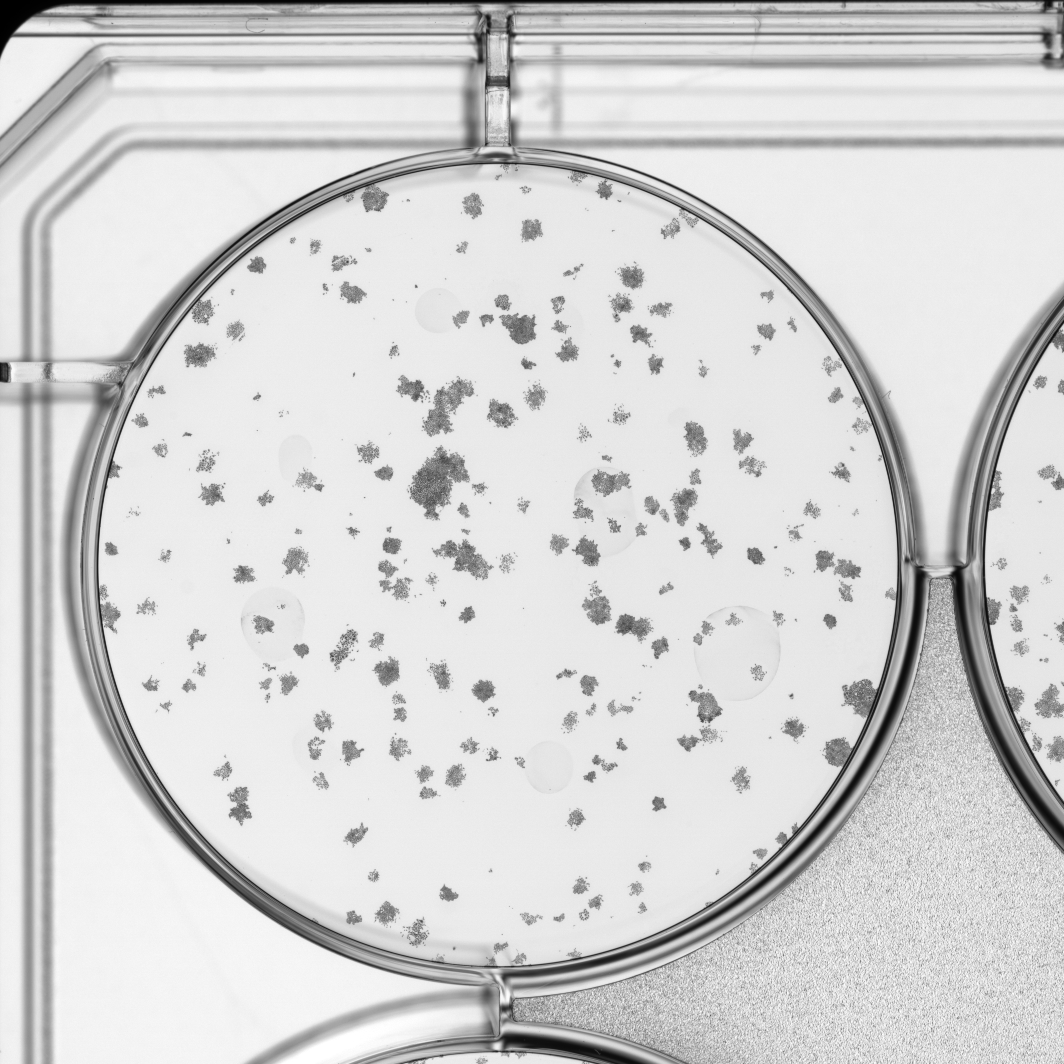

Supplement: Supplementary file 5 — Source data Fig. 5 [file 44318_2026_790_MOESM5_ESM.zip › Figure 5/Figure 5G_clonogenics_U2OS_siFANCM/U2OS_WT_siCTRL.tif]

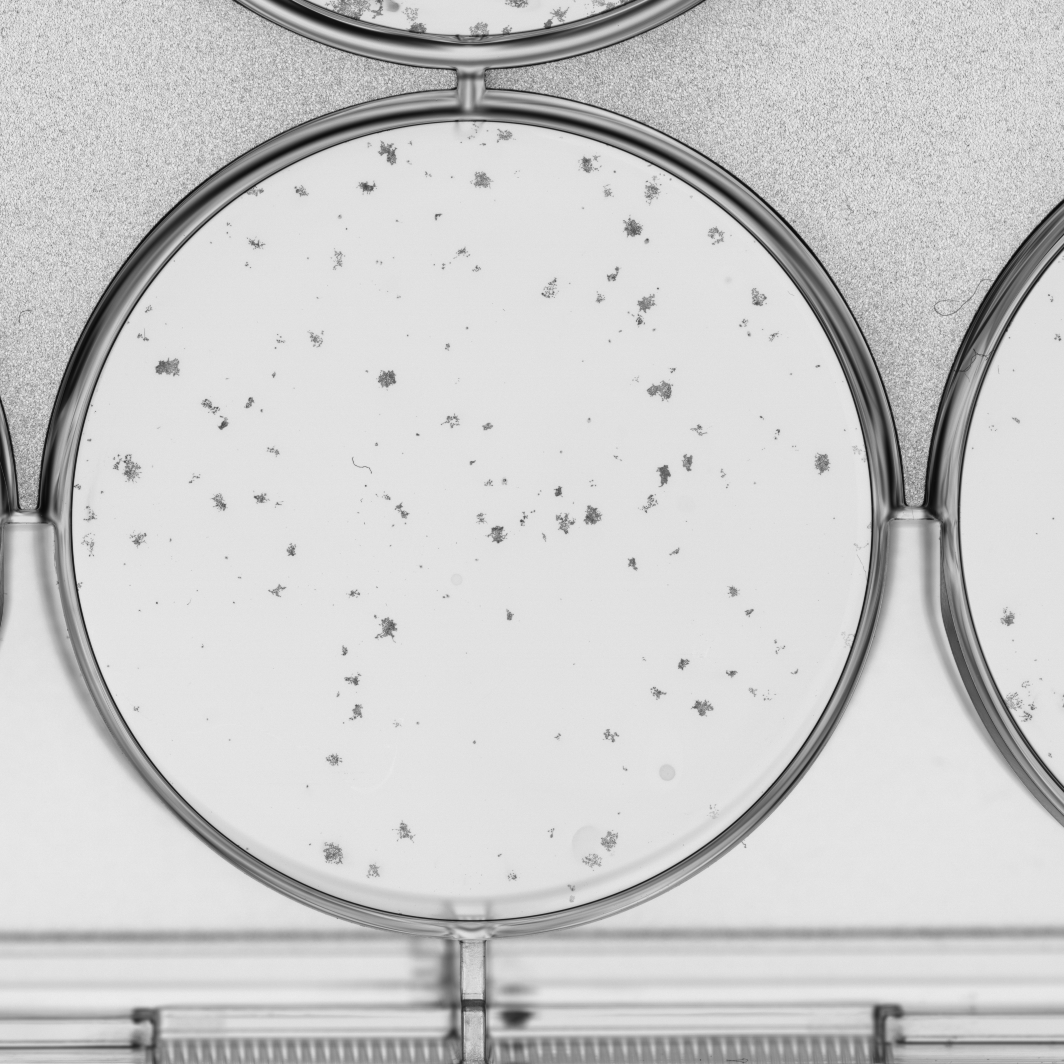

Supplement: Supplementary file 5 — Source data Fig. 5 [file 44318_2026_790_MOESM5_ESM.zip › Figure 5/Figure 5G_clonogenics_U2OS_siFANCM/U2OS_SLX4IP_KO_clone_2_siFANCM.tif]

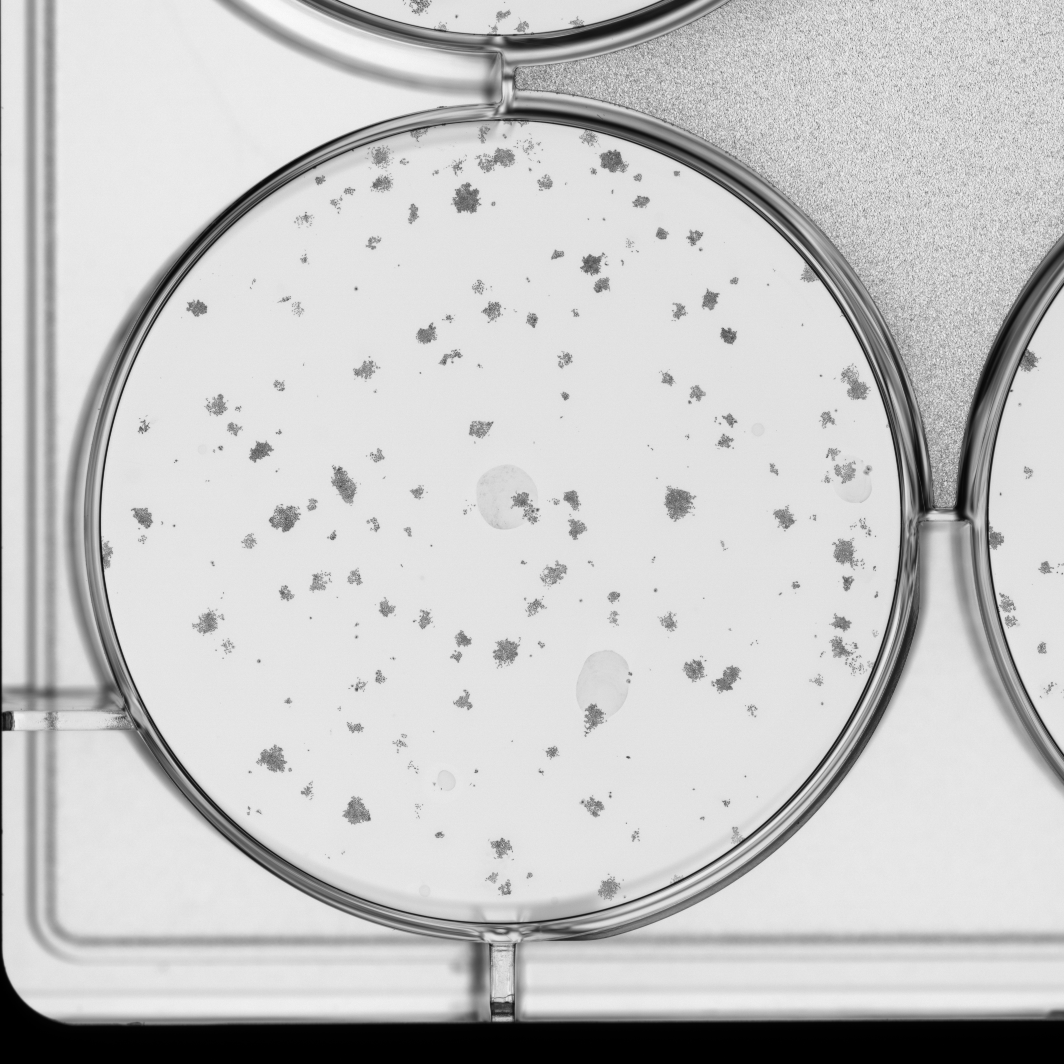

Supplement: Supplementary file 5 — Source data Fig. 5 [file 44318_2026_790_MOESM5_ESM.zip › Figure 5/Figure 5G_clonogenics_U2OS_siFANCM/U2OS_WT_siFANCM.tif]

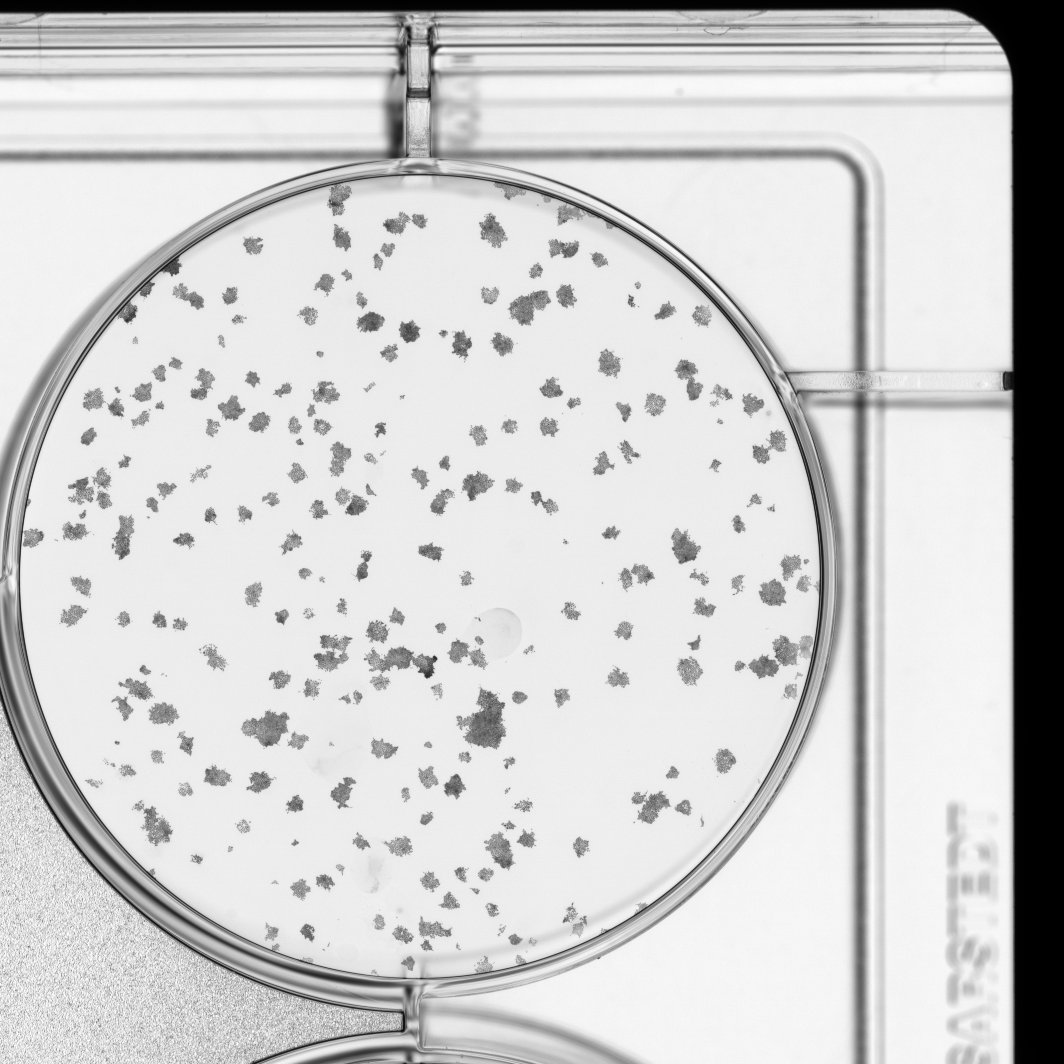

Supplement: Supplementary file 5 — Source data Fig. 5 [file 44318_2026_790_MOESM5_ESM.zip › Figure 5/Figure 5G_clonogenics_U2OS_siFANCM/U2OS_SLX4IP_KO_clone_1_siCTRL.tif]

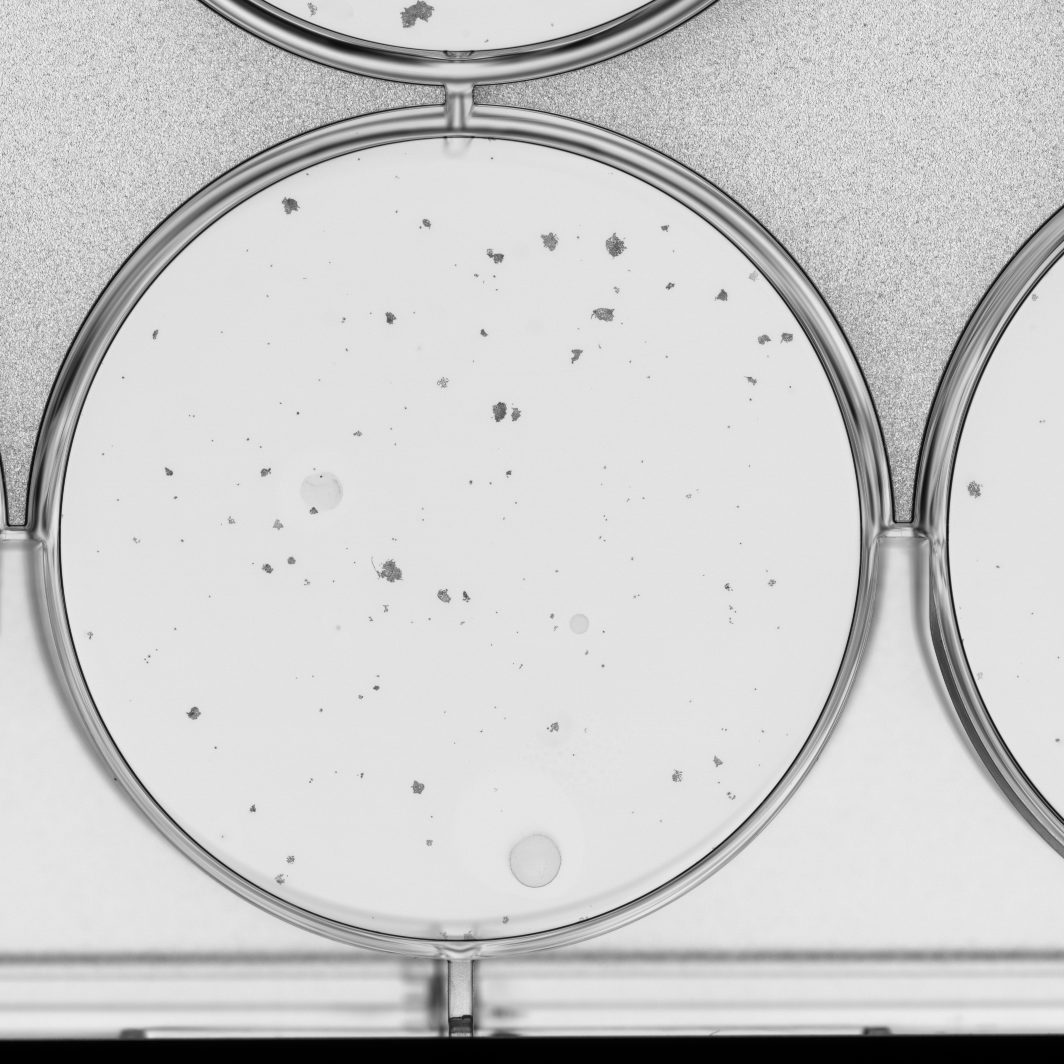

Supplement: Supplementary file 5 — Source data Fig. 5 [file 44318_2026_790_MOESM5_ESM.zip › Figure 5/Figure 5G_clonogenics_U2OS_siFANCM/U2OS_SLX4IP_KO_clone_1_siFANCM.tif]

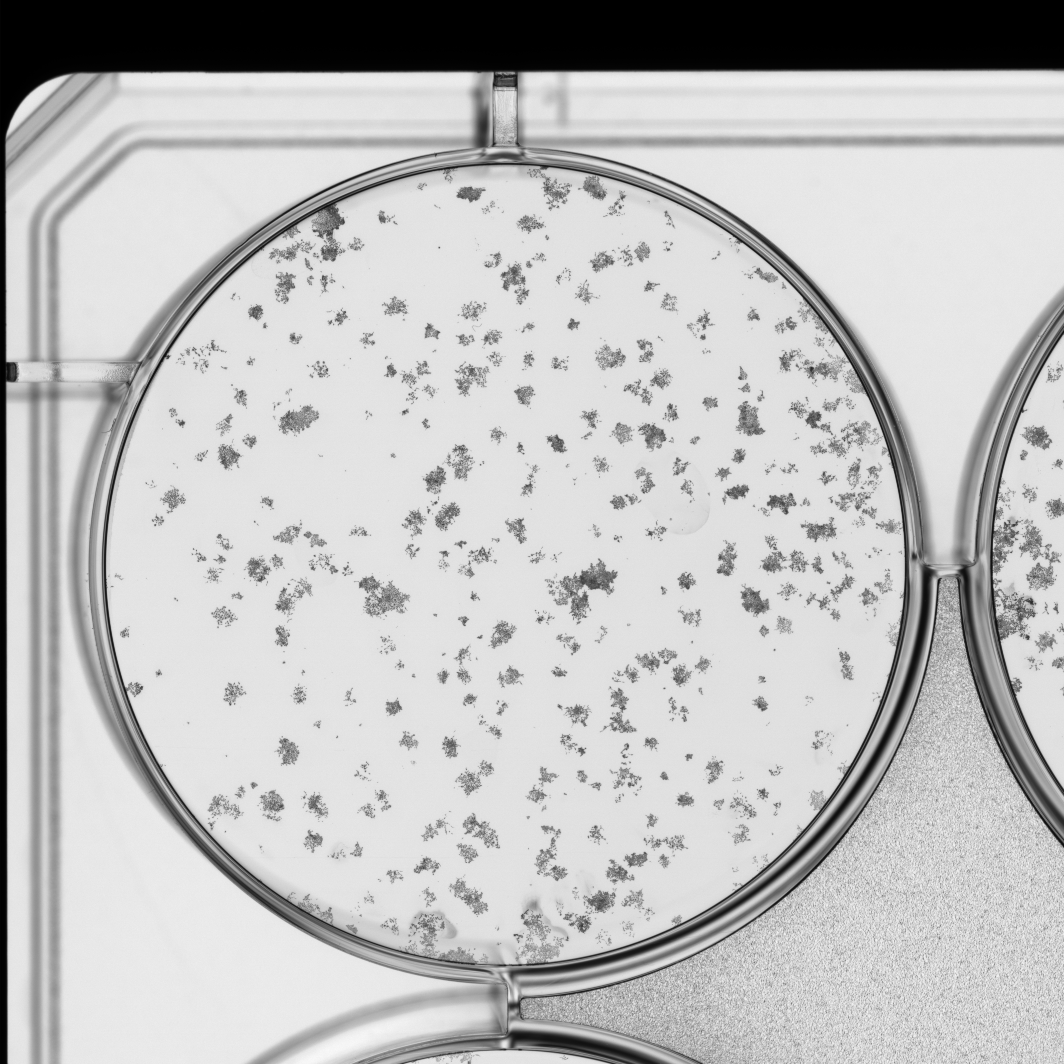

Supplement: Supplementary file 5 — Source data Fig. 5 [file 44318_2026_790_MOESM5_ESM.zip › Figure 5/Figure 5G_clonogenics_U2OS_siFANCM/U2OS_SLX4IP_KO_clone_2_siCTRL.tif]

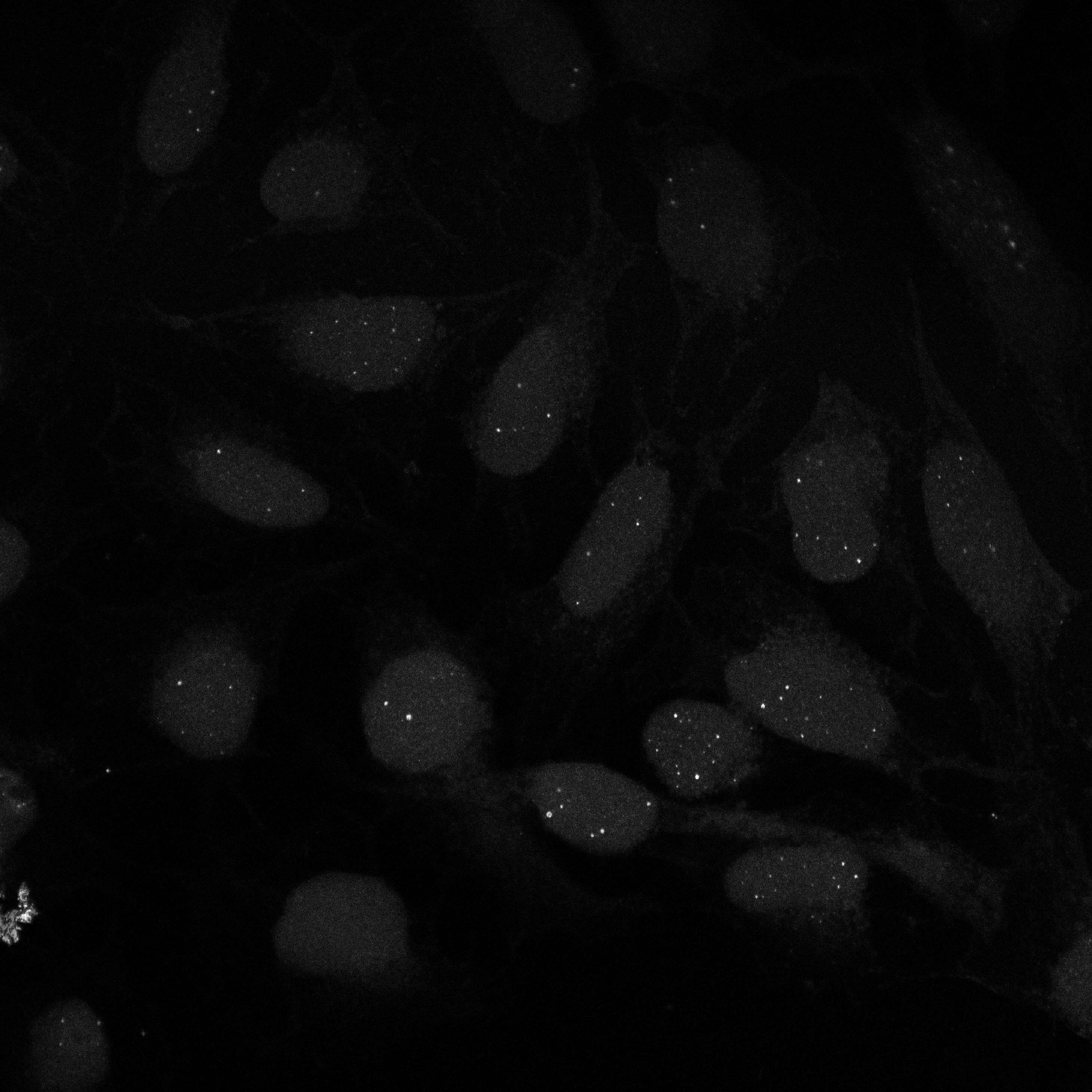

Supplement: Supplementary file 5 — Source data Fig. 5 [file 44318_2026_790_MOESM5_ESM.zip › Figure 5/Figure 5E_PML_TelC_U2OS_siFANCM/C2-U2OS_WT_siFANCM_PML.tif]

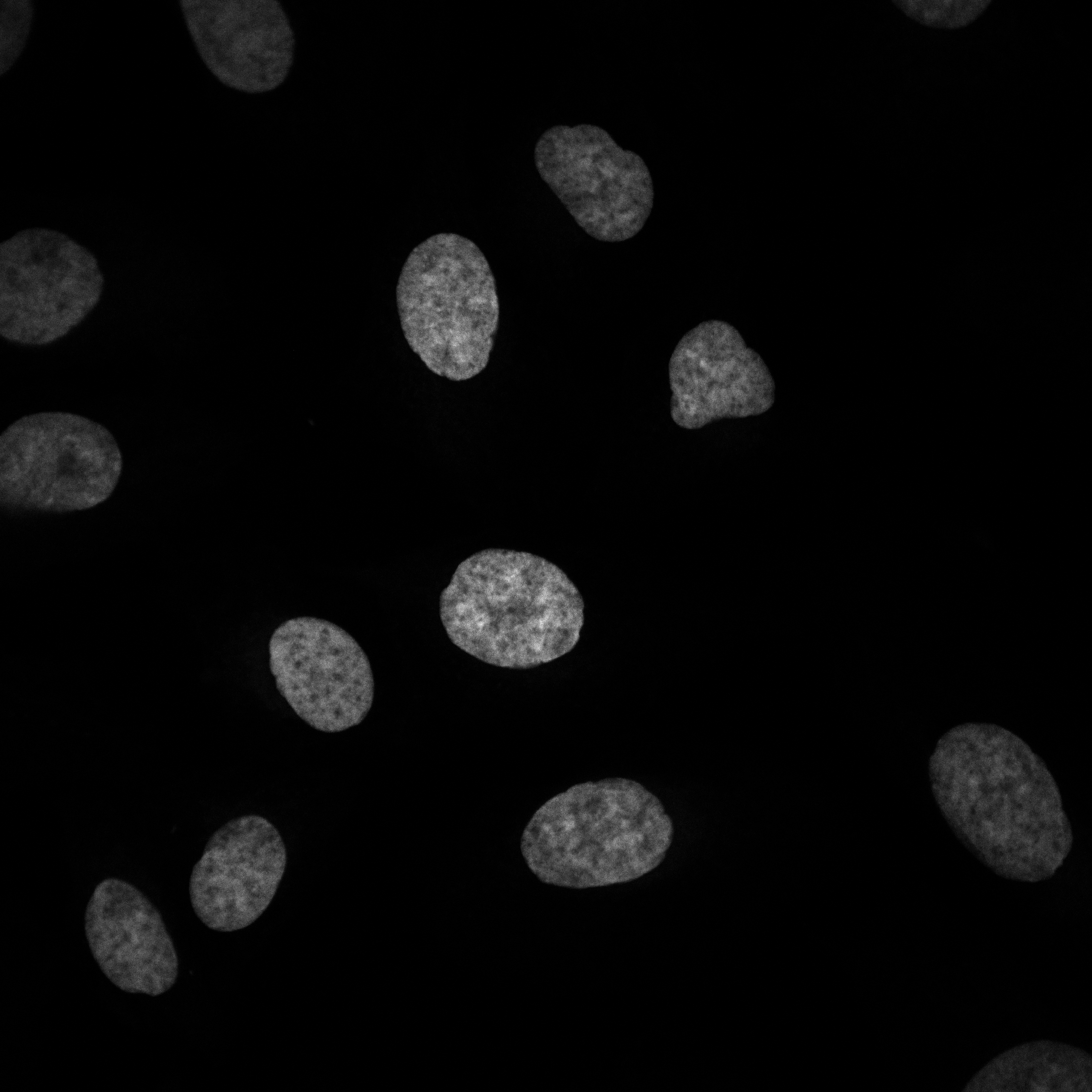

Supplement: Supplementary file 5 — Source data Fig. 5 [file 44318_2026_790_MOESM5_ESM.zip › Figure 5/Figure 5E_PML_TelC_U2OS_siFANCM/C1-U2OS_KO_clone_1_siFANCM_DAPI.tif]

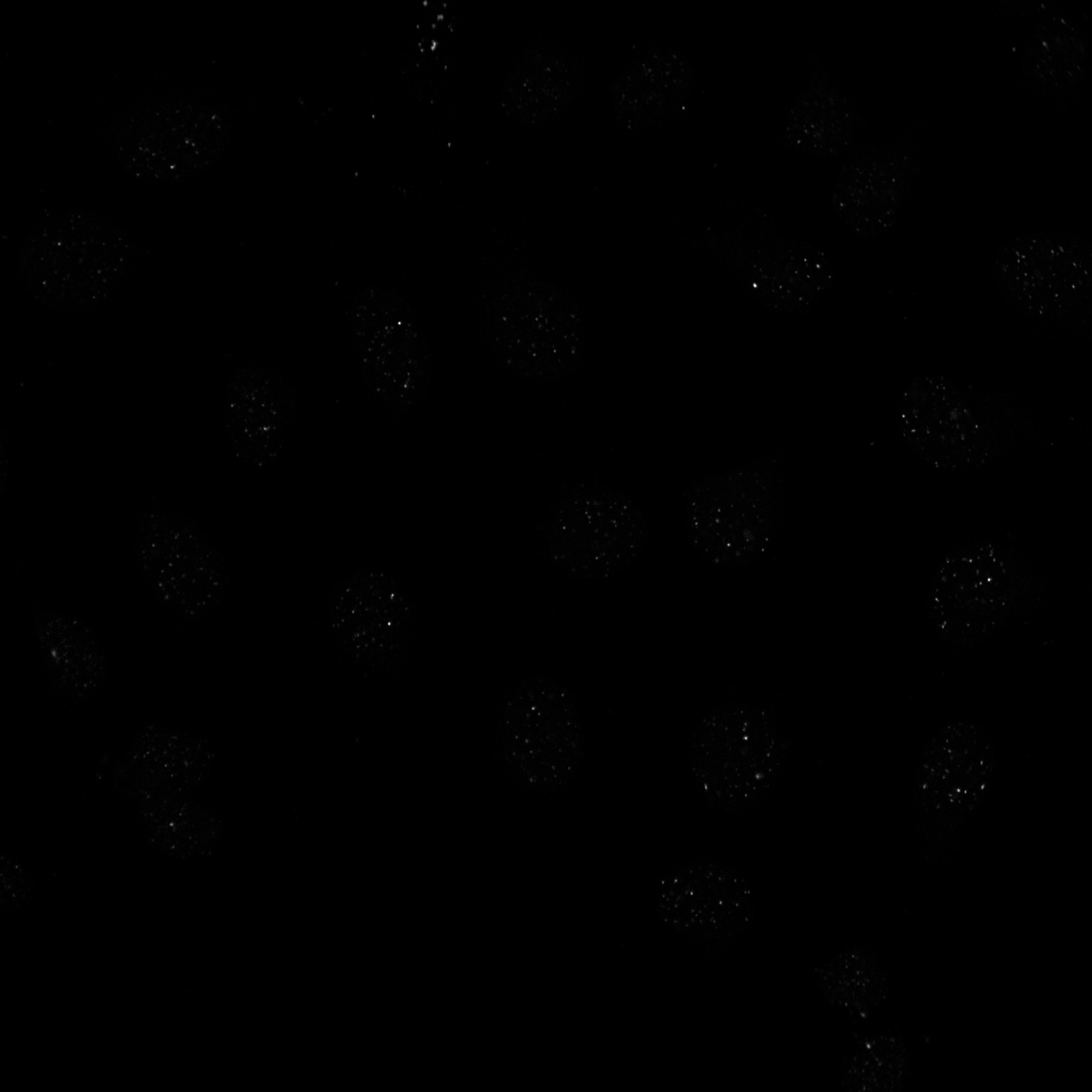

Supplement: Supplementary file 5 — Source data Fig. 5 [file 44318_2026_790_MOESM5_ESM.zip › Figure 5/Figure 5E_PML_TelC_U2OS_siFANCM/C4-U2OS_KO_clone_2_siCTRL_TelC.tif]

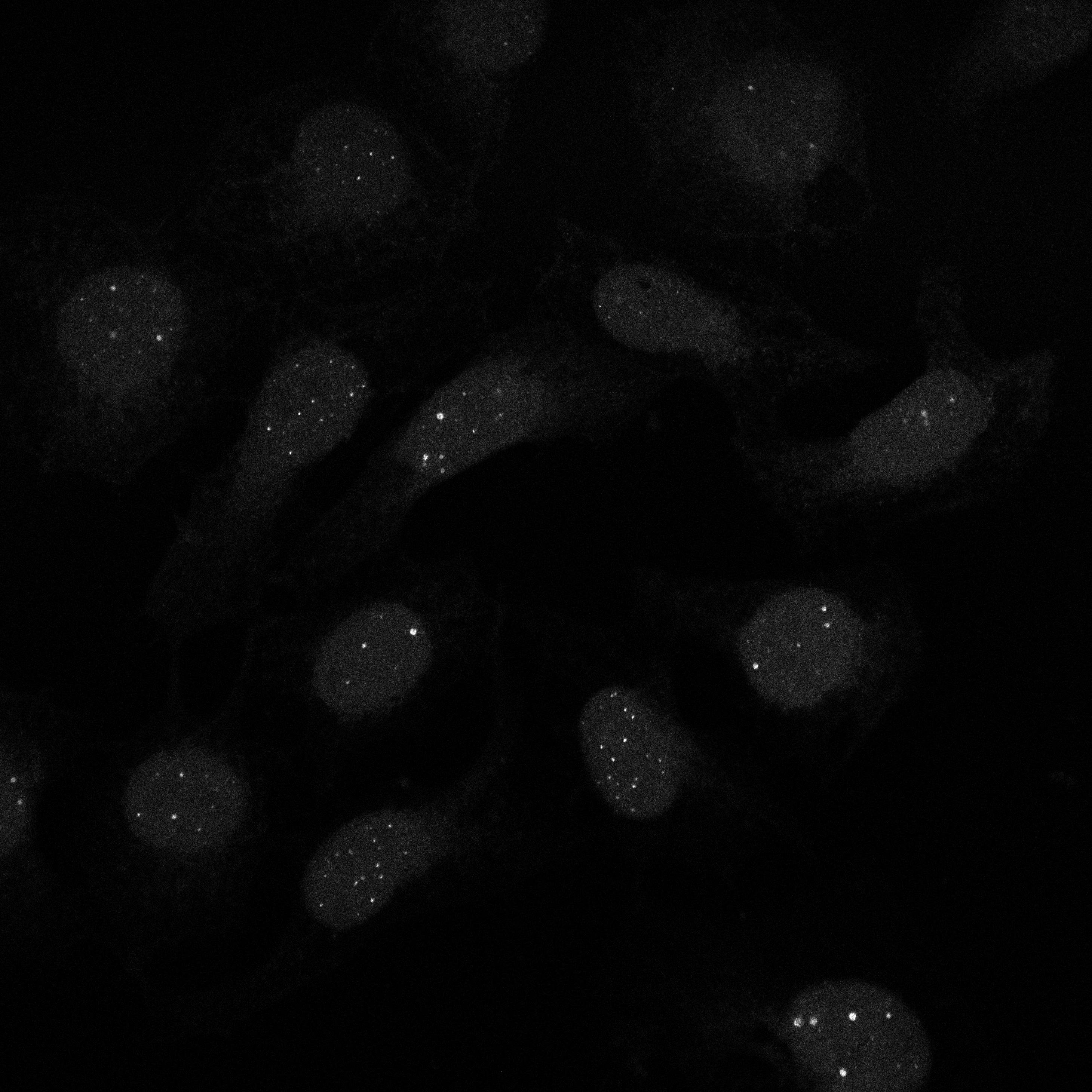

Supplement: Supplementary file 5 — Source data Fig. 5 [file 44318_2026_790_MOESM5_ESM.zip › Figure 5/Figure 5E_PML_TelC_U2OS_siFANCM/C2-U2OS_KO_clone_2_siFANCM_PML.tif]

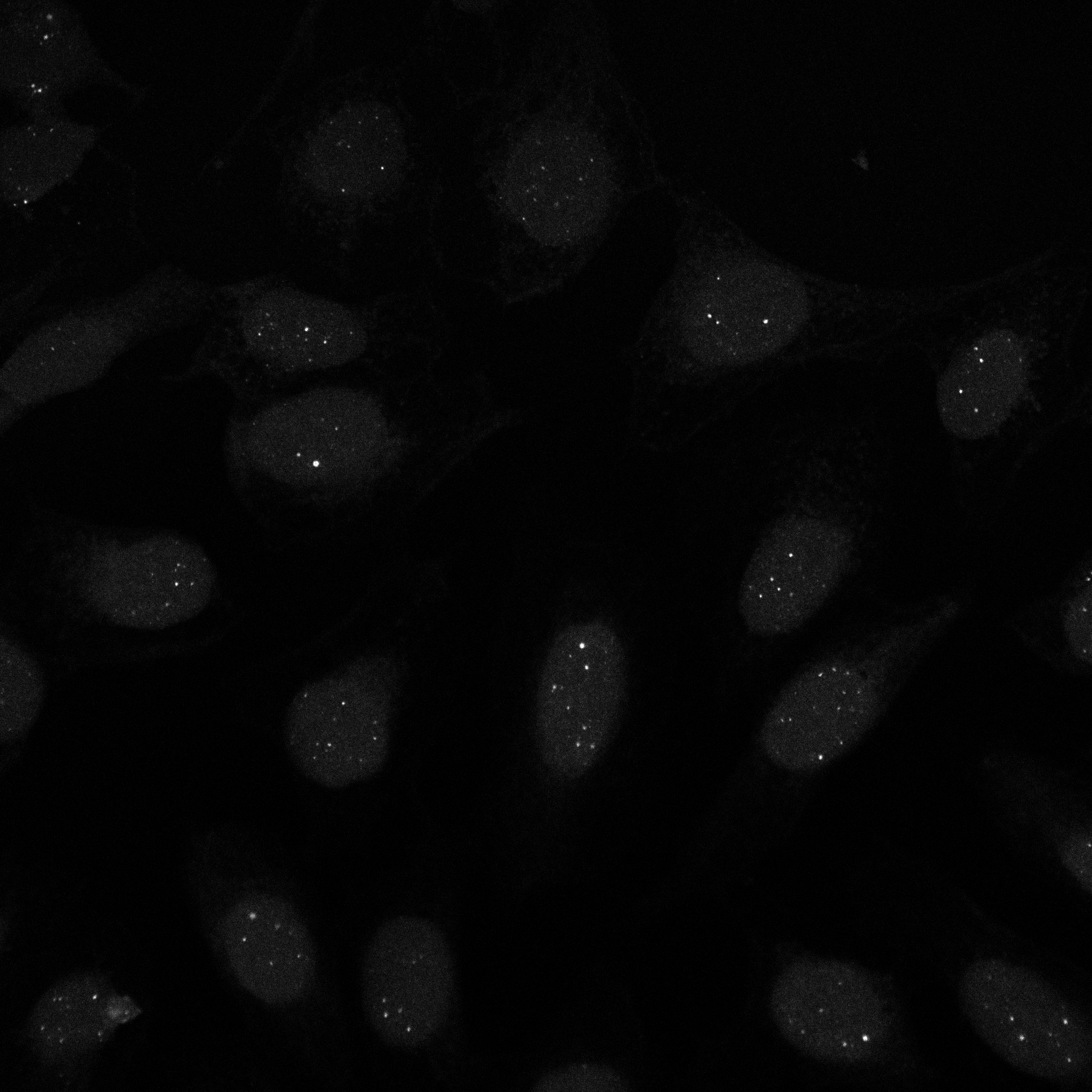

Supplement: Supplementary file 5 — Source data Fig. 5 [file 44318_2026_790_MOESM5_ESM.zip › Figure 5/Figure 5E_PML_TelC_U2OS_siFANCM/C2-U2OS_KO_clone_1_siCTRL_PML.tif]

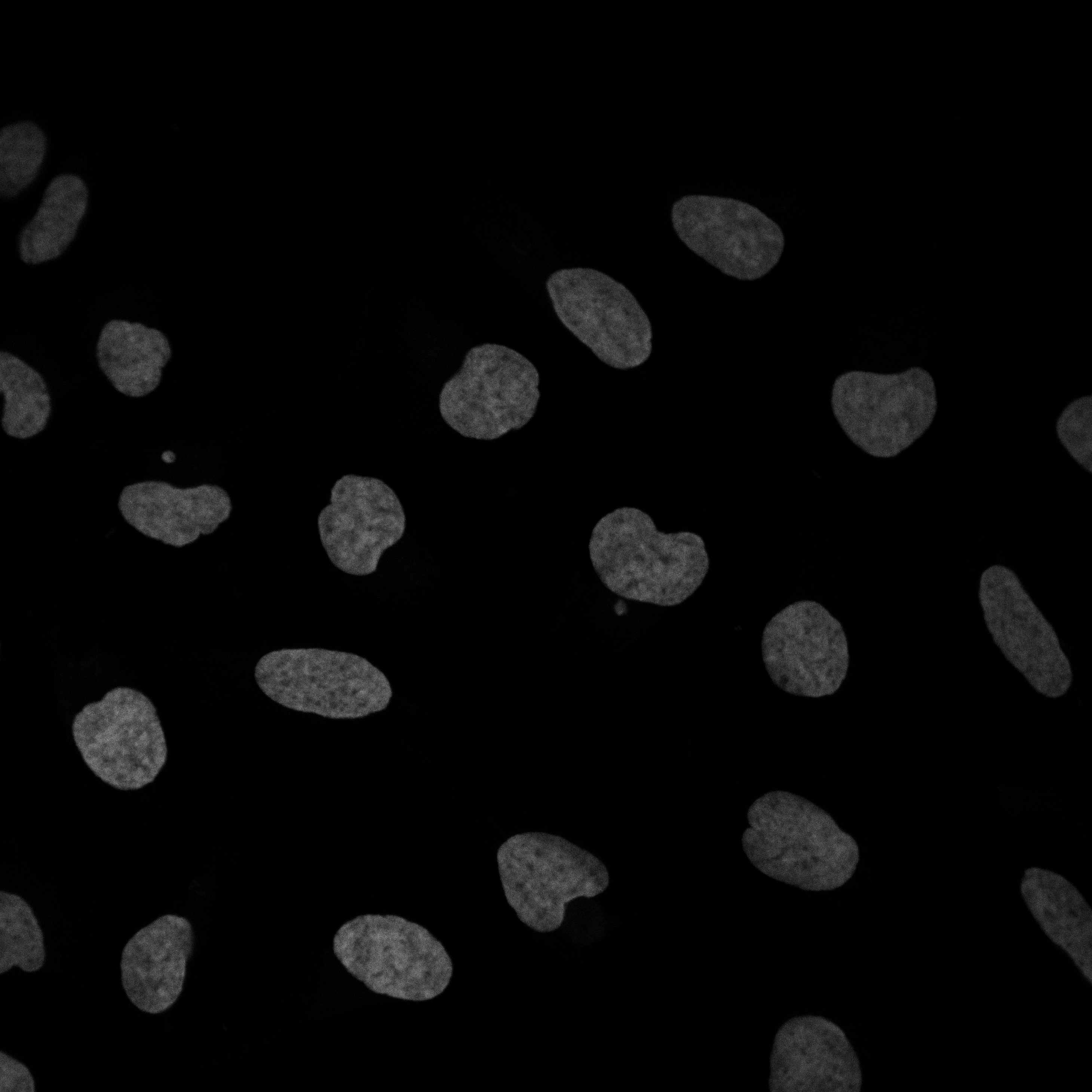

Supplement: Supplementary file 5 — Source data Fig. 5 [file 44318_2026_790_MOESM5_ESM.zip › Figure 5/Figure 5E_PML_TelC_U2OS_siFANCM/C1-U2OS_WT_siCTRL_DAPI.tif]

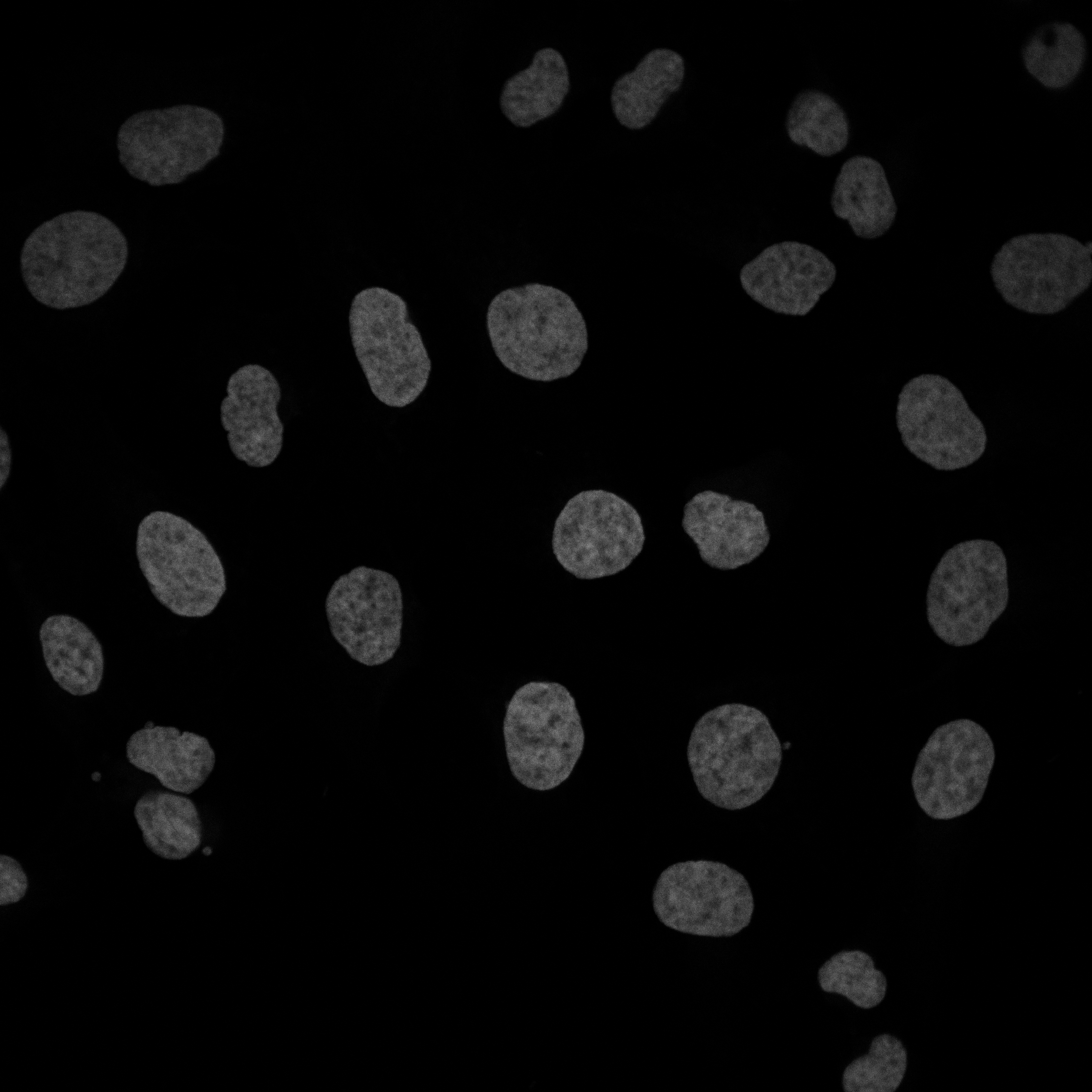

Supplement: Supplementary file 5 — Source data Fig. 5 [file 44318_2026_790_MOESM5_ESM.zip › Figure 5/Figure 5E_PML_TelC_U2OS_siFANCM/C1-U2OS_KO_clone_2_siCTRL_DAPI.tif]

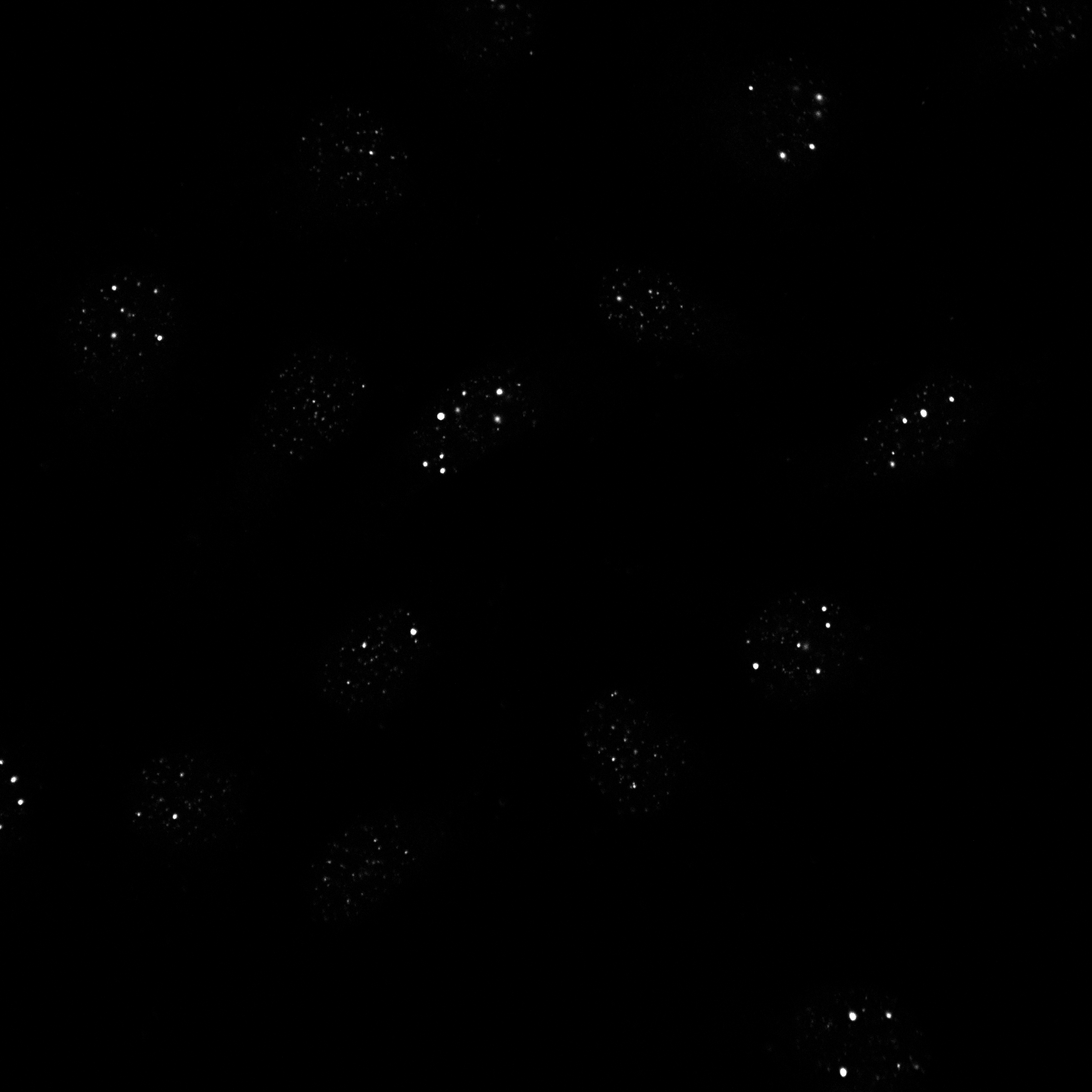

Supplement: Supplementary file 5 — Source data Fig. 5 [file 44318_2026_790_MOESM5_ESM.zip › Figure 5/Figure 5E_PML_TelC_U2OS_siFANCM/C4-U2OS_KO_clone_2_siFANCM_TelC.tif]

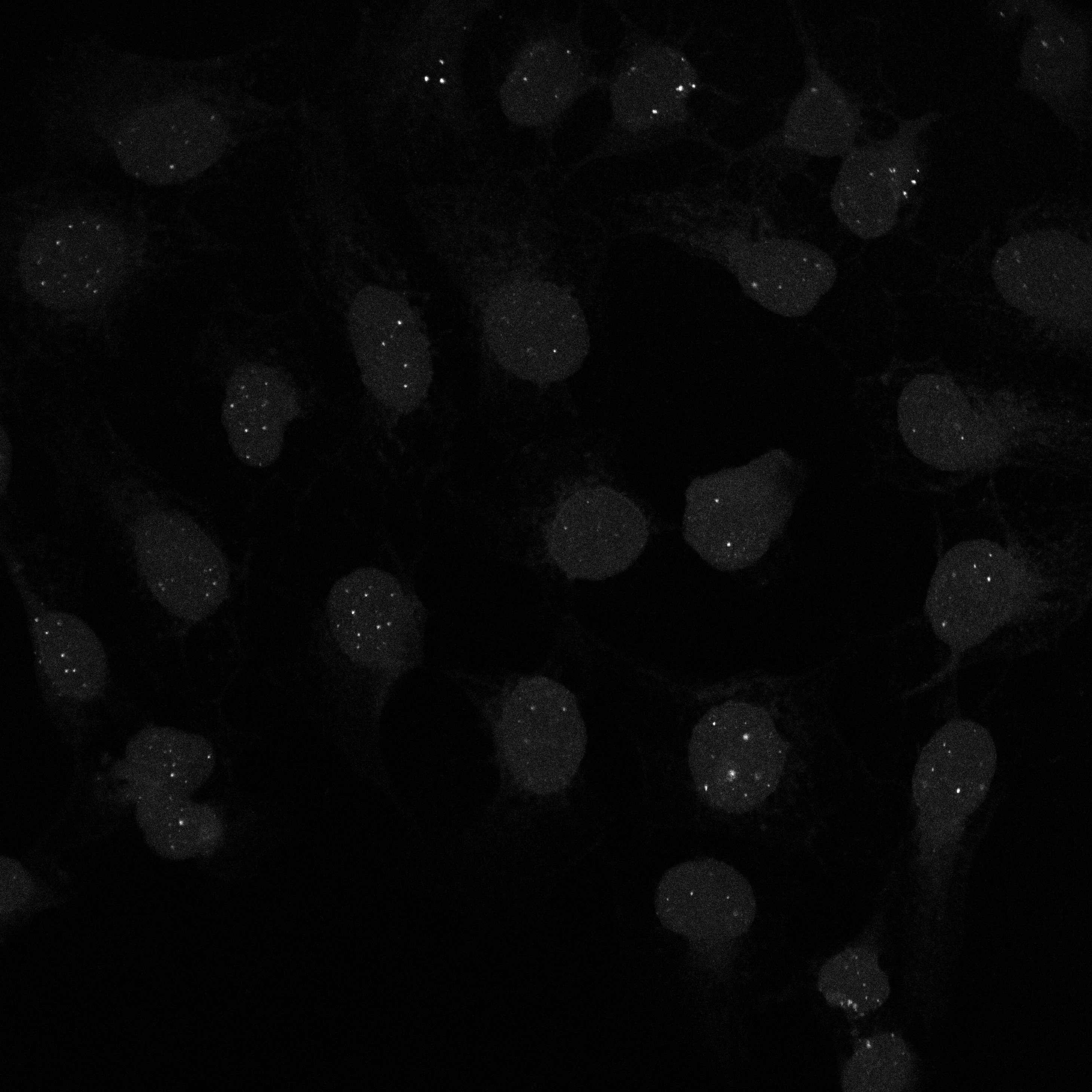

Supplement: Supplementary file 5 — Source data Fig. 5 [file 44318_2026_790_MOESM5_ESM.zip › Figure 5/Figure 5E_PML_TelC_U2OS_siFANCM/C2-U2OS_KO_clone_2_siCTRL_PML.tif]

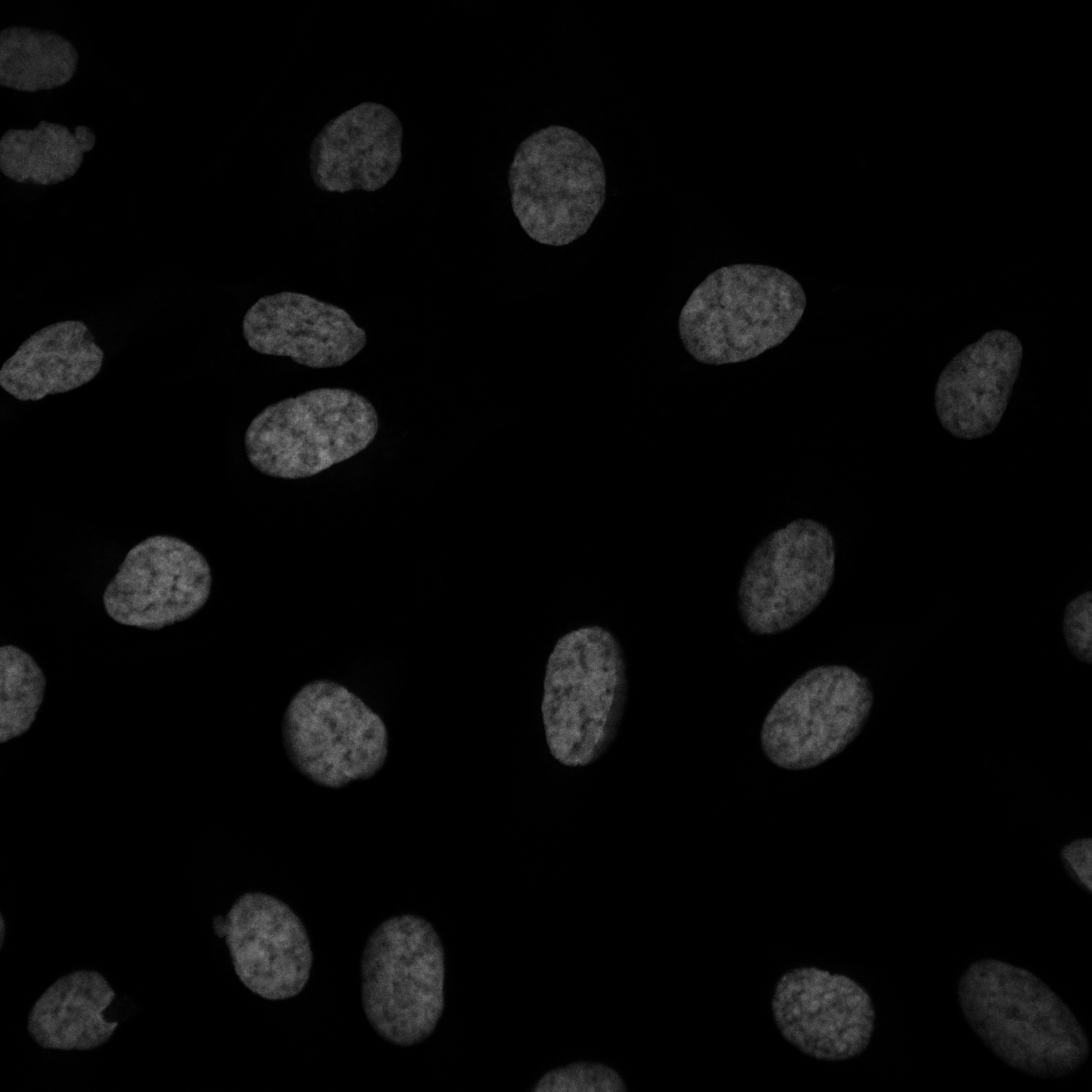

Supplement: Supplementary file 5 — Source data Fig. 5 [file 44318_2026_790_MOESM5_ESM.zip › Figure 5/Figure 5E_PML_TelC_U2OS_siFANCM/C1-U2OS_KO_clone_1_siCTRL_DAPI.tif]

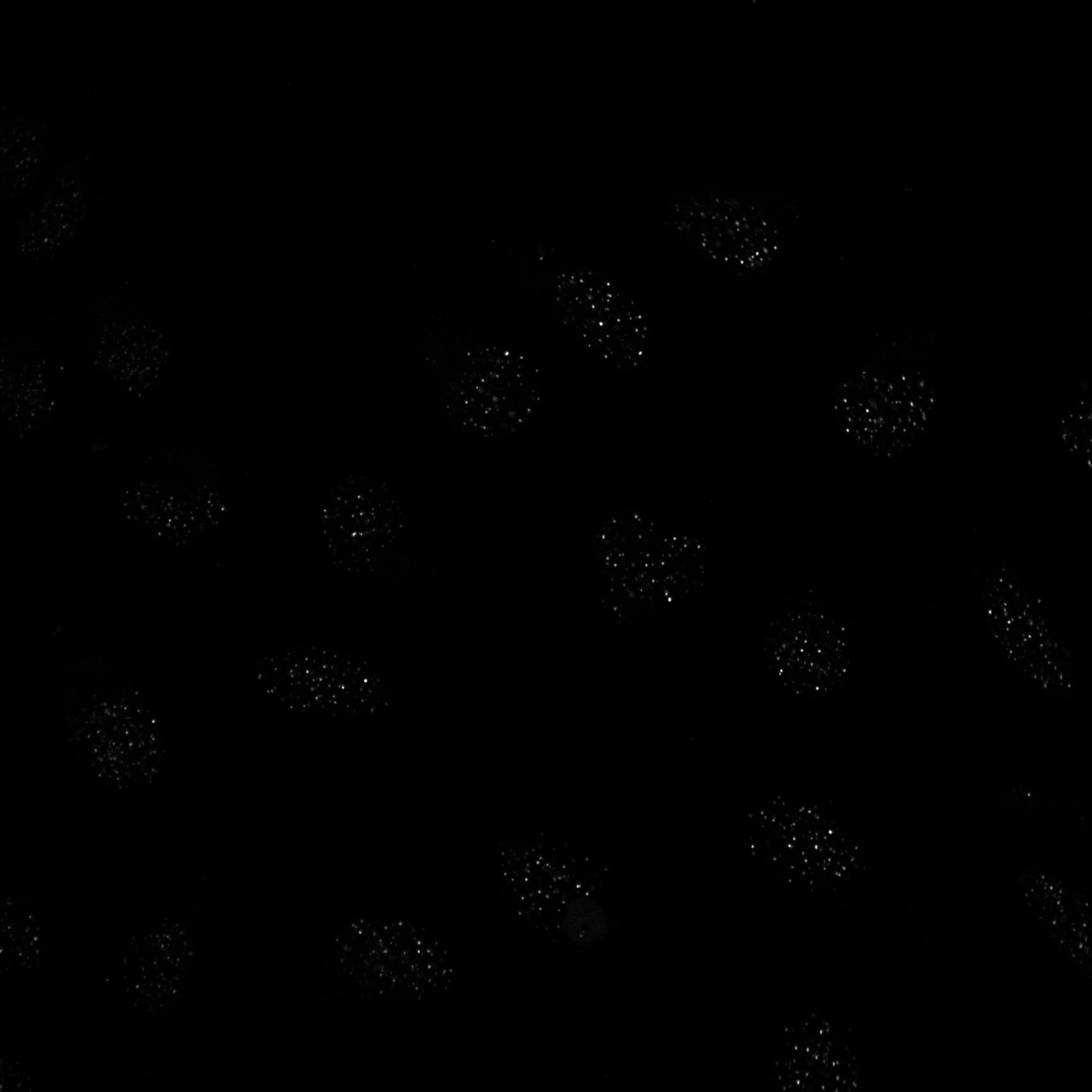

Supplement: Supplementary file 5 — Source data Fig. 5 [file 44318_2026_790_MOESM5_ESM.zip › Figure 5/Figure 5E_PML_TelC_U2OS_siFANCM/C4-U2OS_WT_siCTRL_TelC.tif]

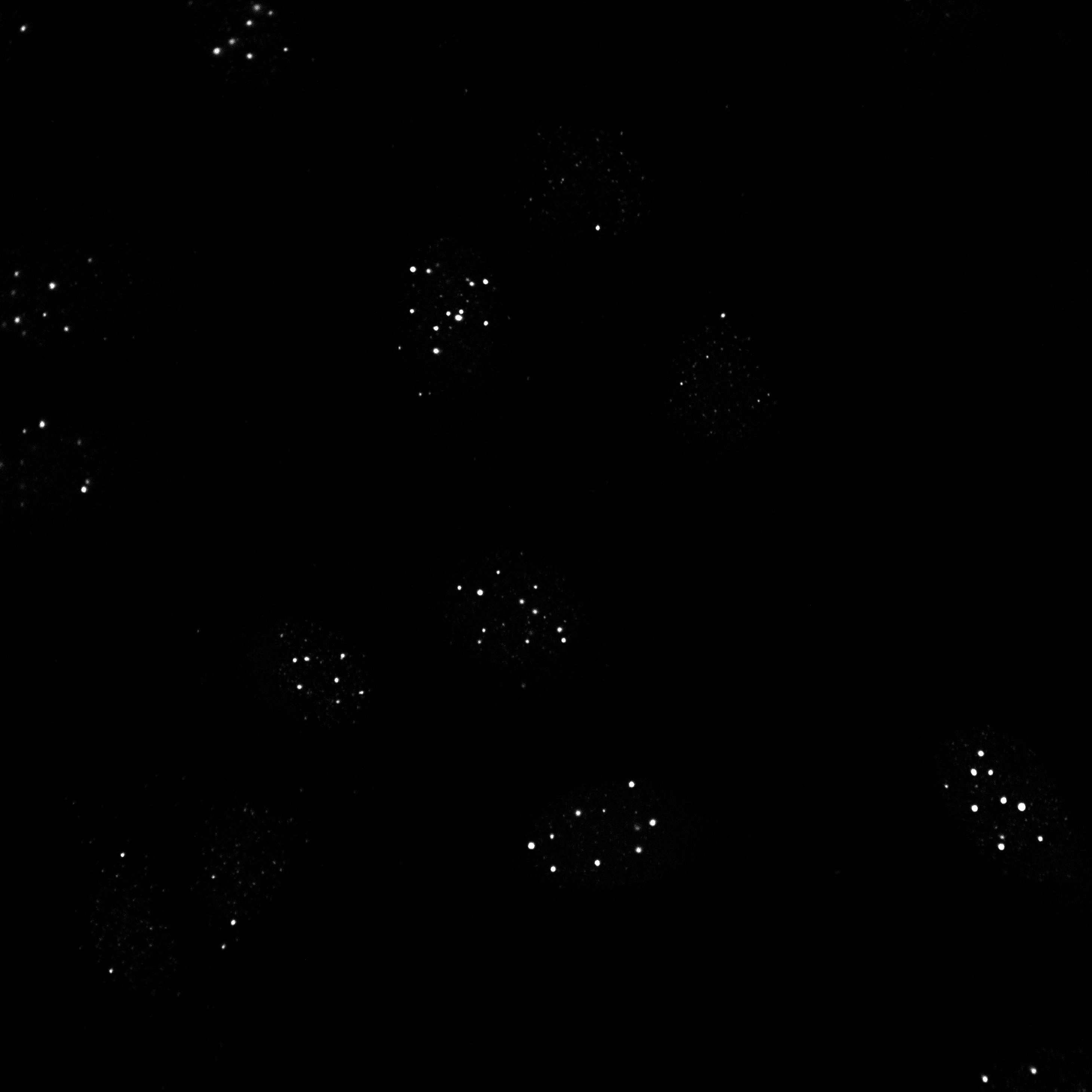

Supplement: Supplementary file 5 — Source data Fig. 5 [file 44318_2026_790_MOESM5_ESM.zip › Figure 5/Figure 5E_PML_TelC_U2OS_siFANCM/C4-U2OS_KO_clone_1_siFANCM_TelC.tif]

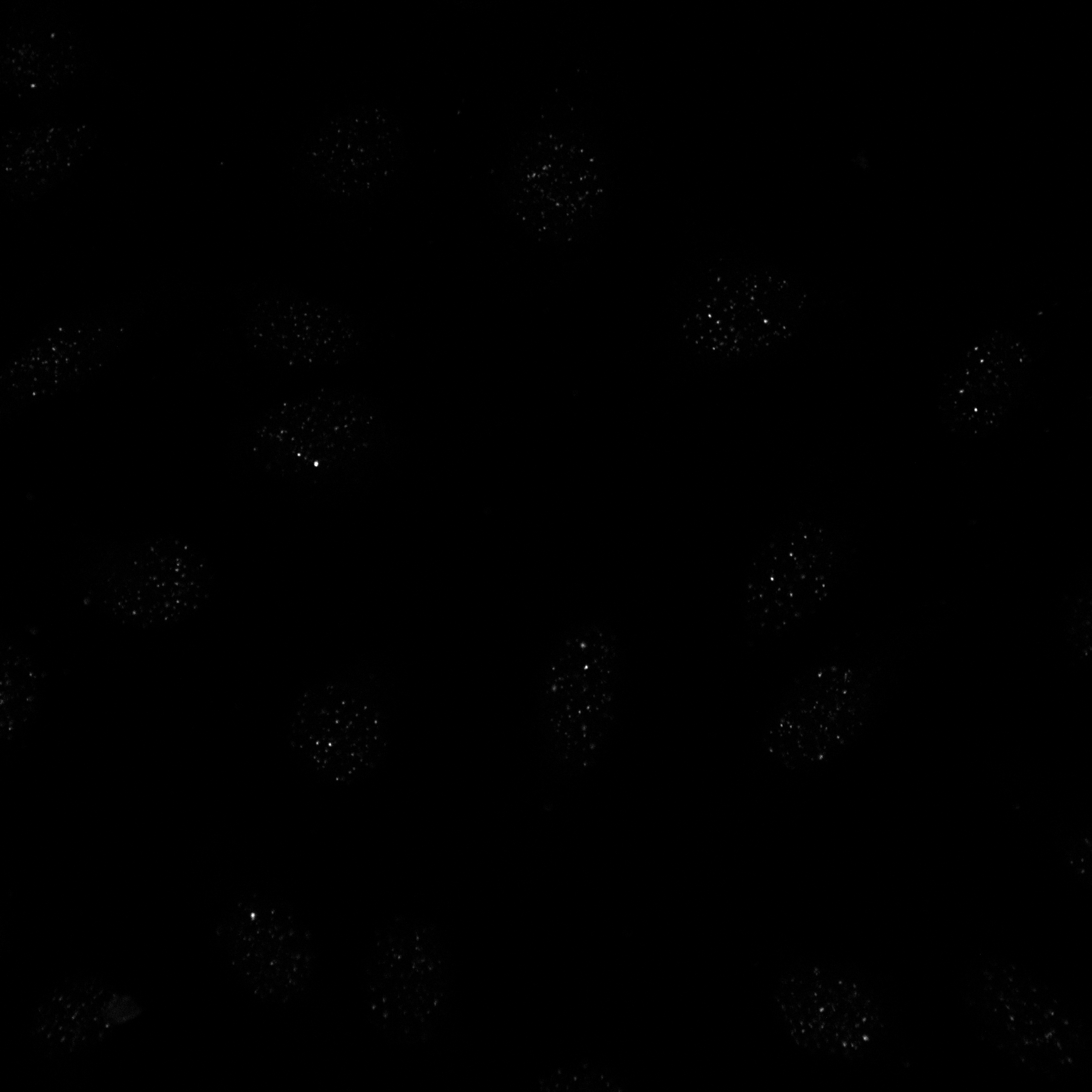

Supplement: Supplementary file 5 — Source data Fig. 5 [file 44318_2026_790_MOESM5_ESM.zip › Figure 5/Figure 5E_PML_TelC_U2OS_siFANCM/C4-U2OS_KO_clone_1_siCTRL_TelC.tif]

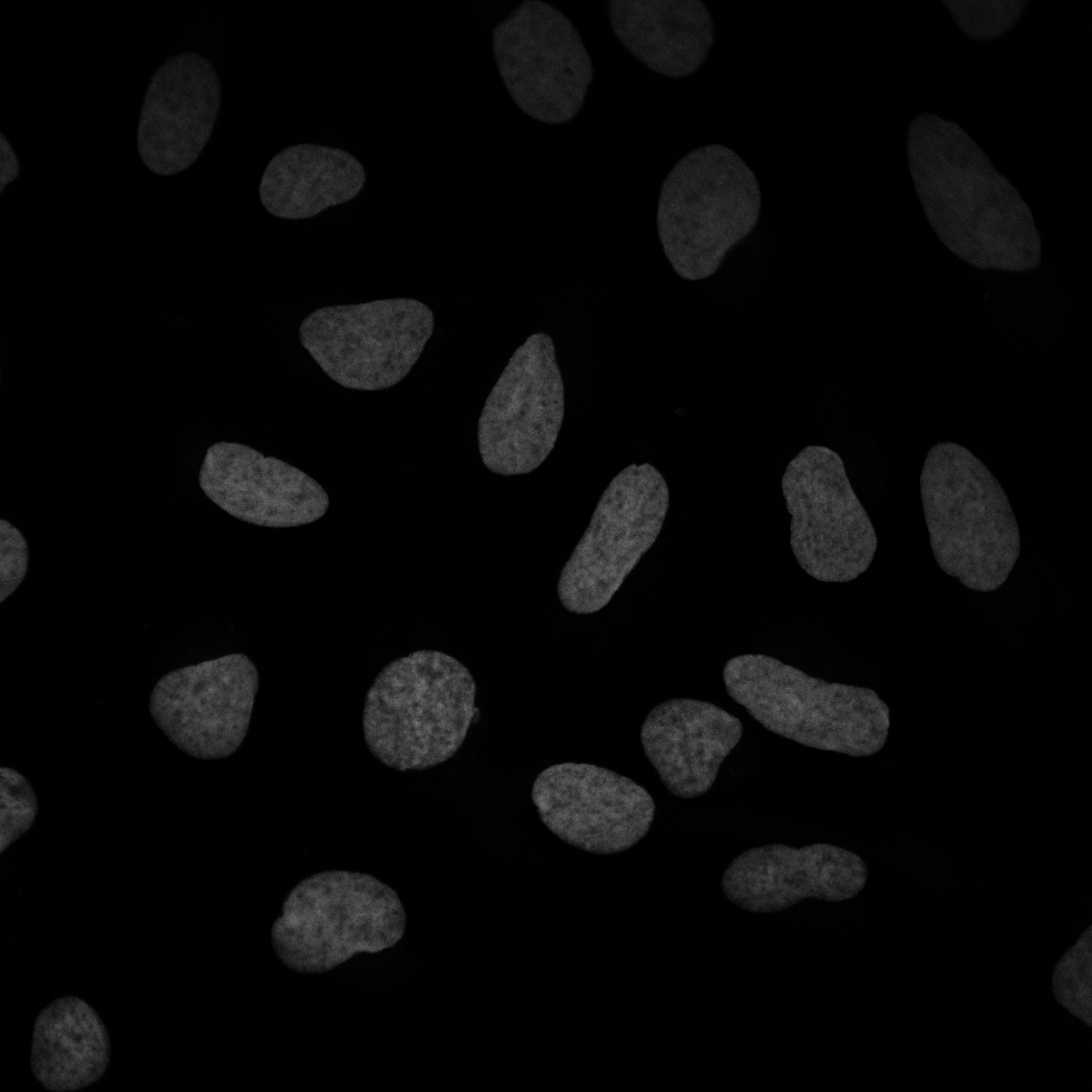

Supplement: Supplementary file 5 — Source data Fig. 5 [file 44318_2026_790_MOESM5_ESM.zip › Figure 5/Figure 5E_PML_TelC_U2OS_siFANCM/C1-U2OS_WT_siFANCM_DAPI.tif]

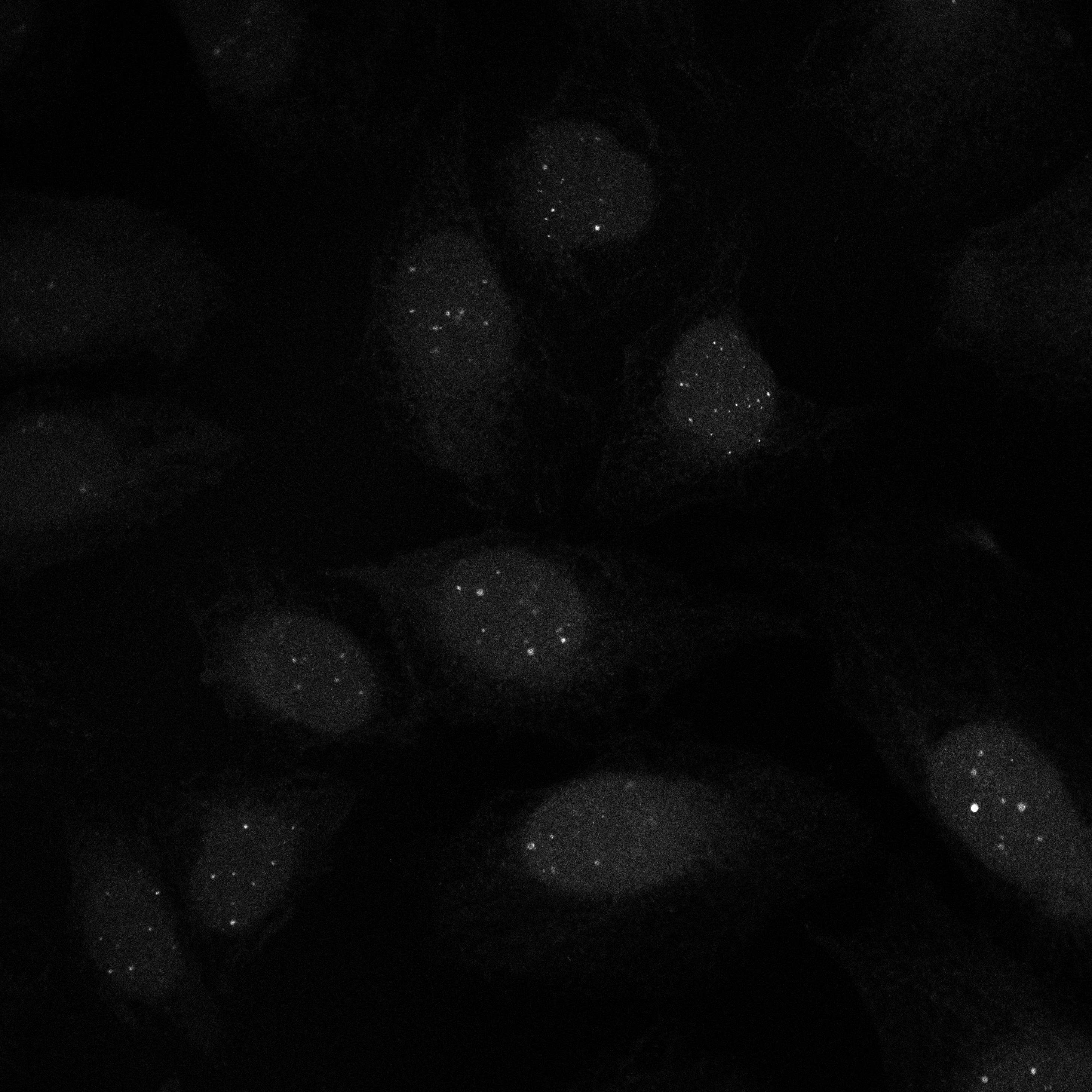

Supplement: Supplementary file 5 — Source data Fig. 5 [file 44318_2026_790_MOESM5_ESM.zip › Figure 5/Figure 5E_PML_TelC_U2OS_siFANCM/C2-U2OS_KO_clone_1_siFANCM_PML.tif]

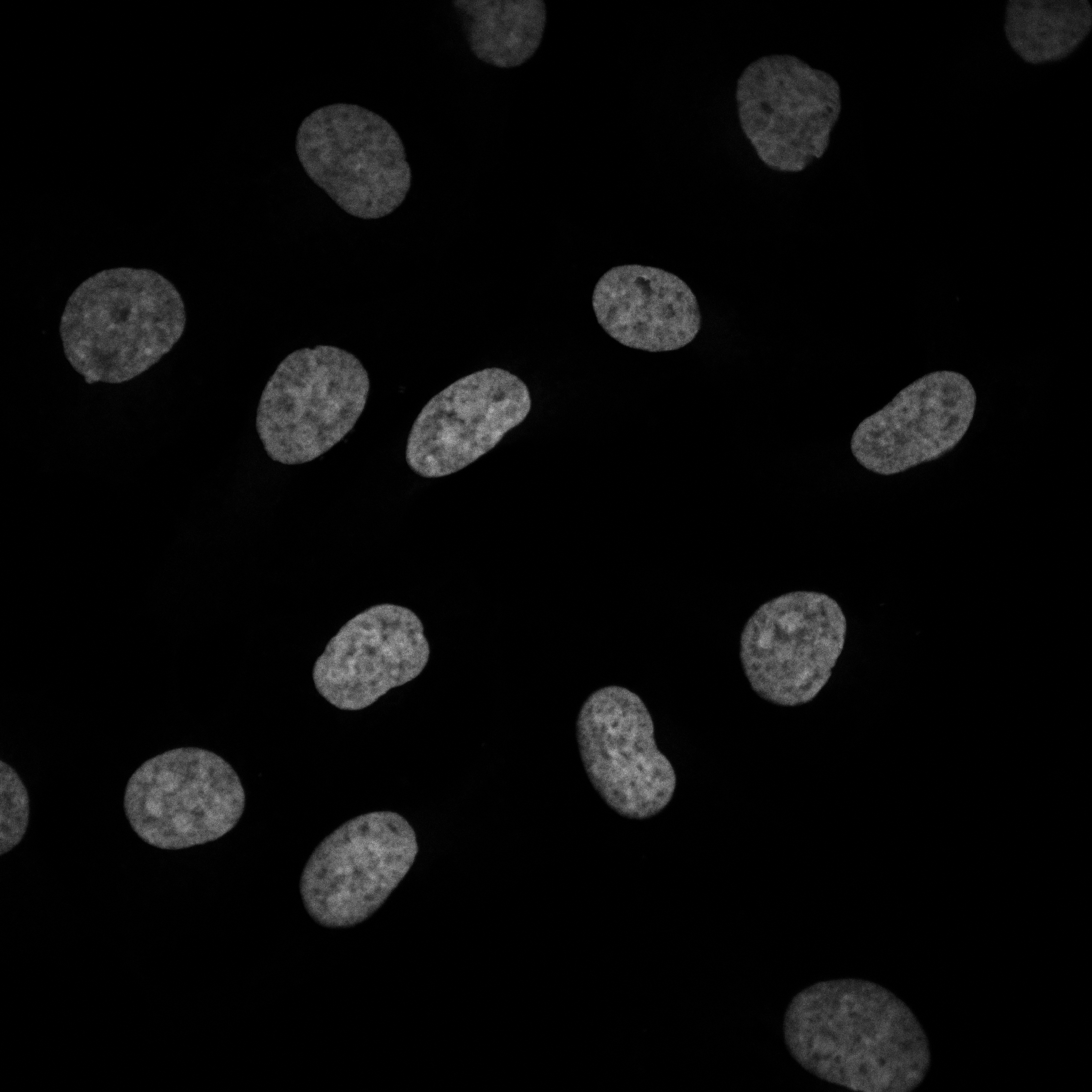

Supplement: Supplementary file 5 — Source data Fig. 5 [file 44318_2026_790_MOESM5_ESM.zip › Figure 5/Figure 5E_PML_TelC_U2OS_siFANCM/C1-U2OS_KO_clone_2_siFANCM_DAPI.tif]

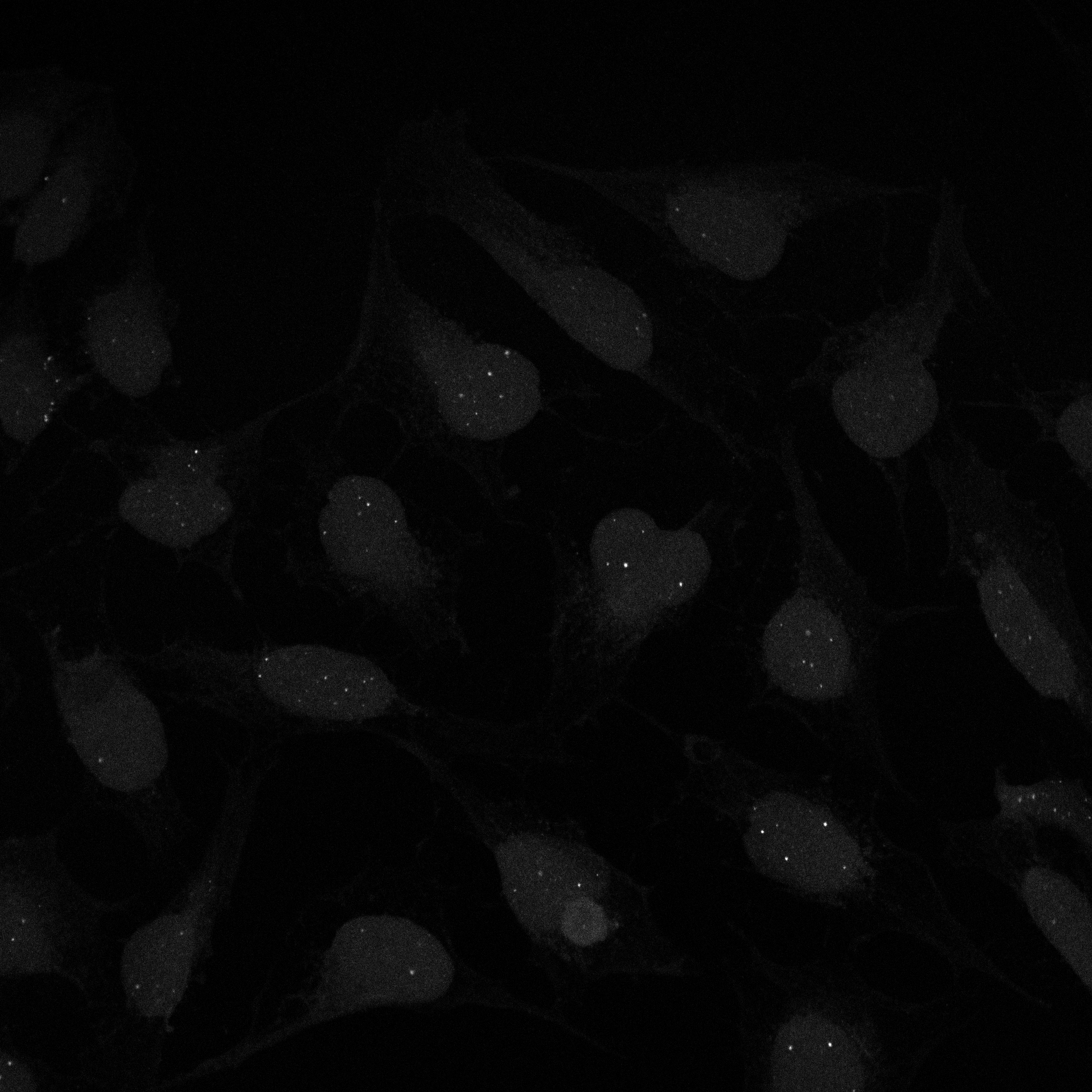

Supplement: Supplementary file 5 — Source data Fig. 5 [file 44318_2026_790_MOESM5_ESM.zip › Figure 5/Figure 5E_PML_TelC_U2OS_siFANCM/C2-U2OS_WT_siCTRL_PML.tif]

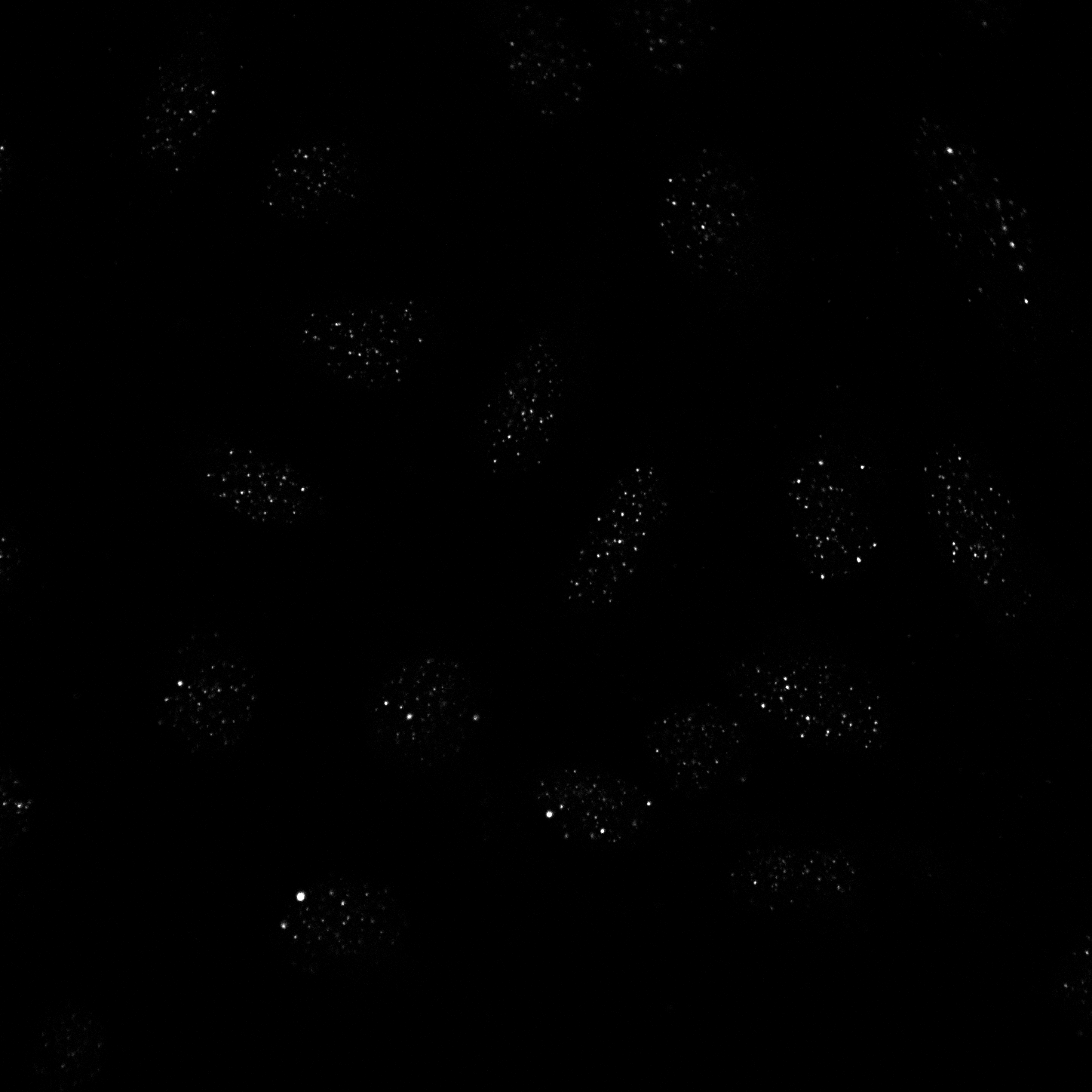

Supplement: Supplementary file 5 — Source data Fig. 5 [file 44318_2026_790_MOESM5_ESM.zip › Figure 5/Figure 5E_PML_TelC_U2OS_siFANCM/C4-U2OS_WT_siFANCM_TelC.tif]

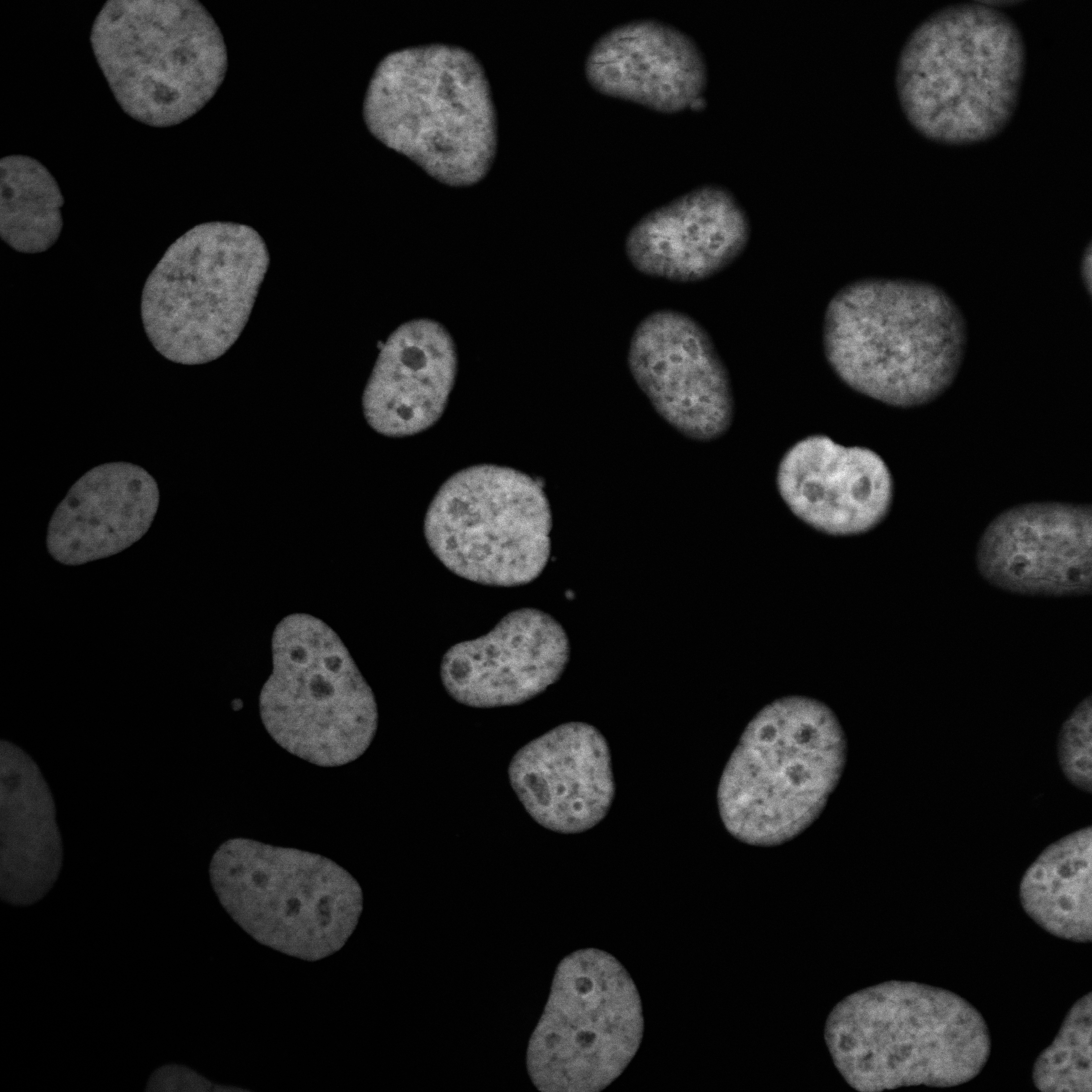

Supplement: Supplementary file 5 — Source data Fig. 5 [file 44318_2026_790_MOESM5_ESM.zip › Figure 5/Figure 5A_pRPA_TelC_U2OS_siFANCM/C1-U2OS_SLX4IP_KO_clone_2_siCTRL_DAPI.tif]

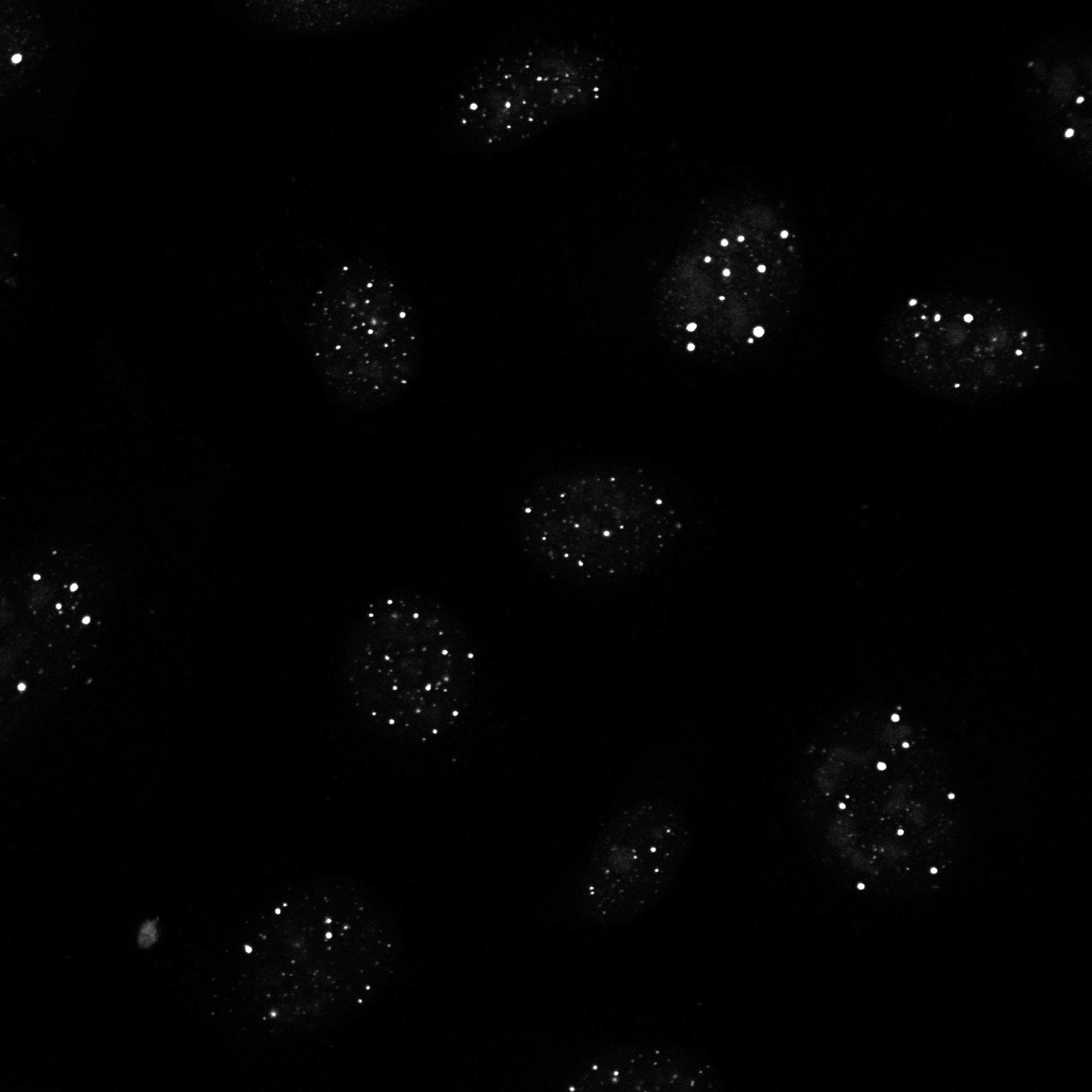

Supplement: Supplementary file 5 — Source data Fig. 5 [file 44318_2026_790_MOESM5_ESM.zip › Figure 5/Figure 5A_pRPA_TelC_U2OS_siFANCM/C3-U2OS_SLX4IP_KO_clone_1_siFANCM_TelC.tif]

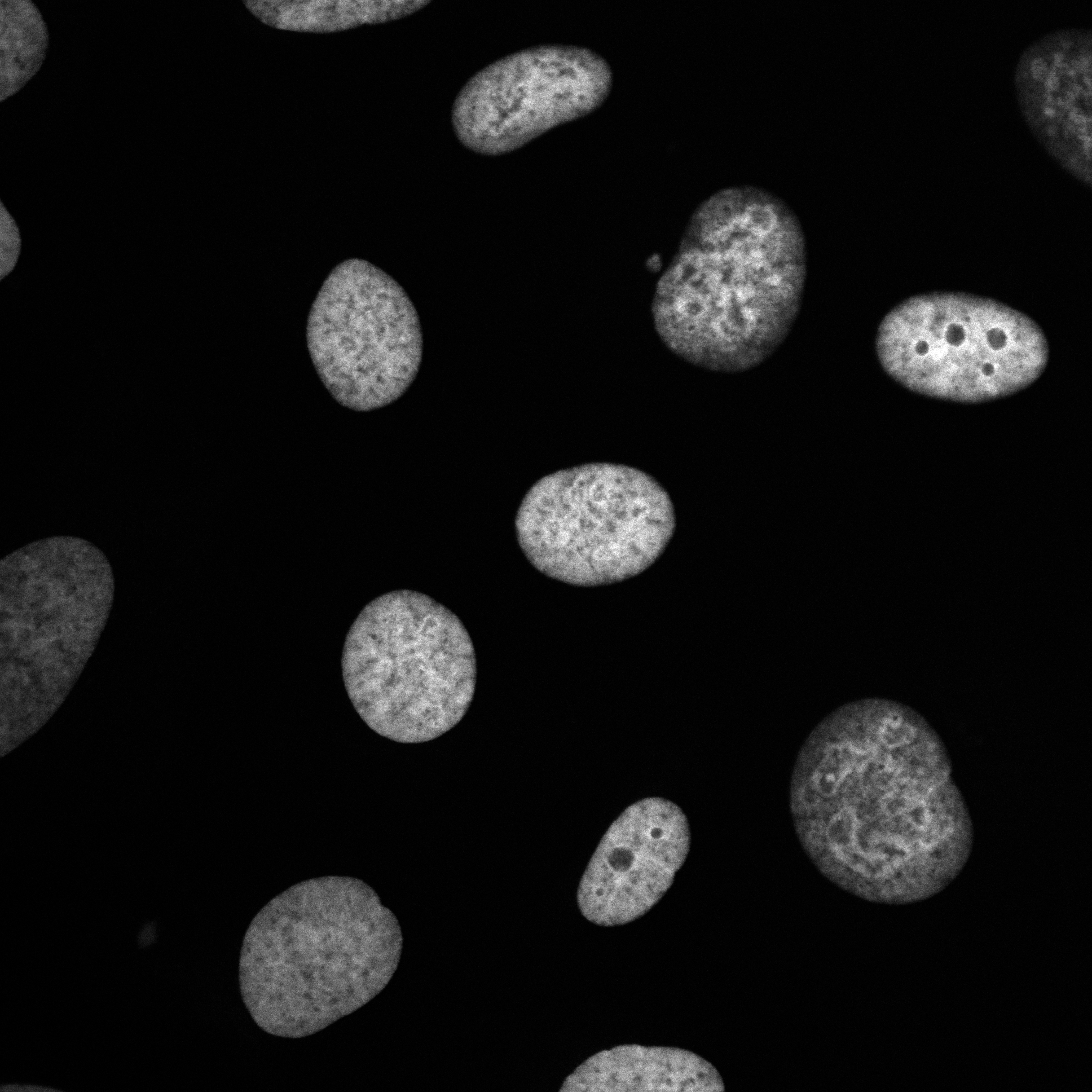

Supplement: Supplementary file 5 — Source data Fig. 5 [file 44318_2026_790_MOESM5_ESM.zip › Figure 5/Figure 5A_pRPA_TelC_U2OS_siFANCM/C1-U2OS_SLX4IP_KO_clone_1_siFANCM_DAPI.tif]

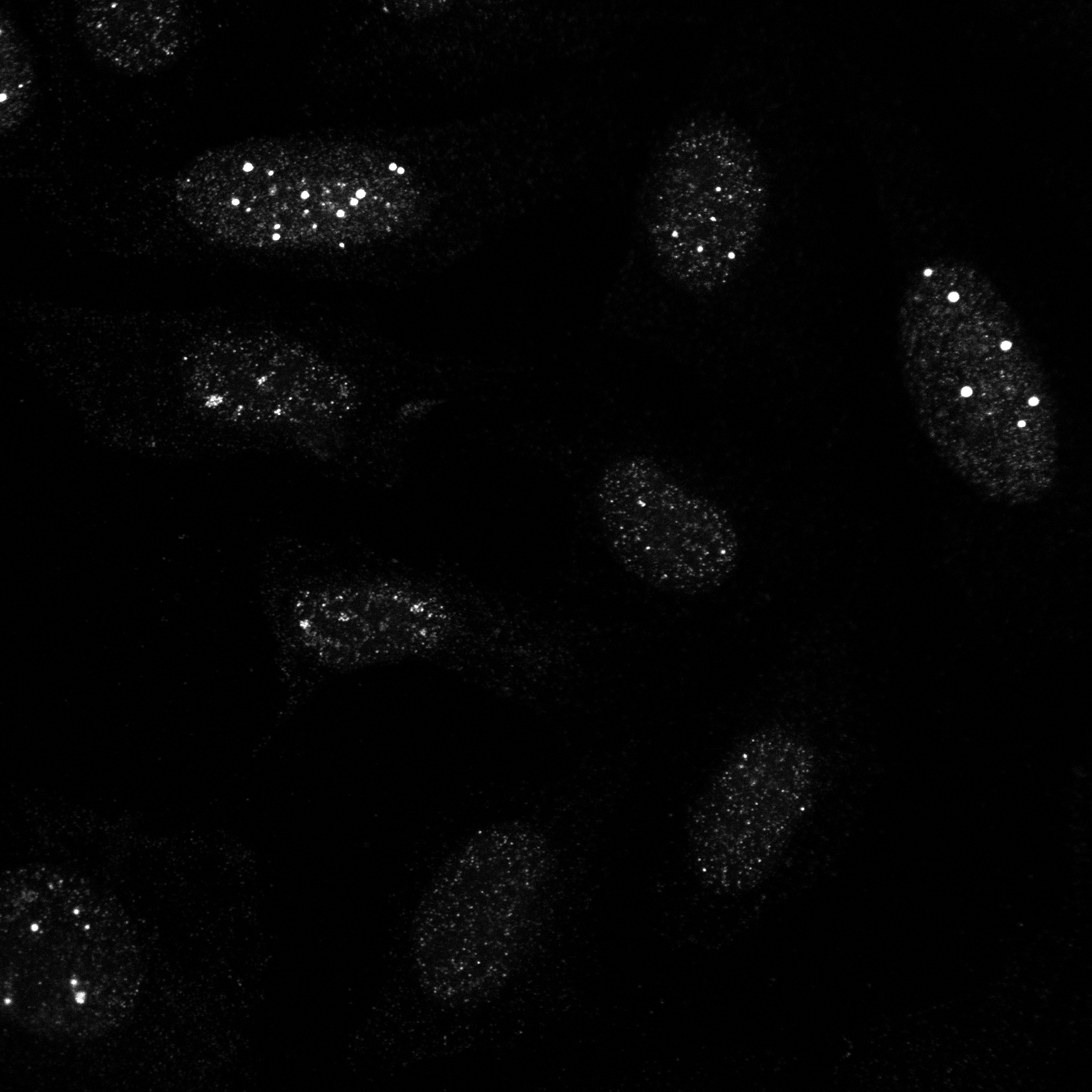

Supplement: Supplementary file 5 — Source data Fig. 5 [file 44318_2026_790_MOESM5_ESM.zip › Figure 5/Figure 5A_pRPA_TelC_U2OS_siFANCM/C2-U2OS_WT_siFANCM_pS33-RPA.tif]

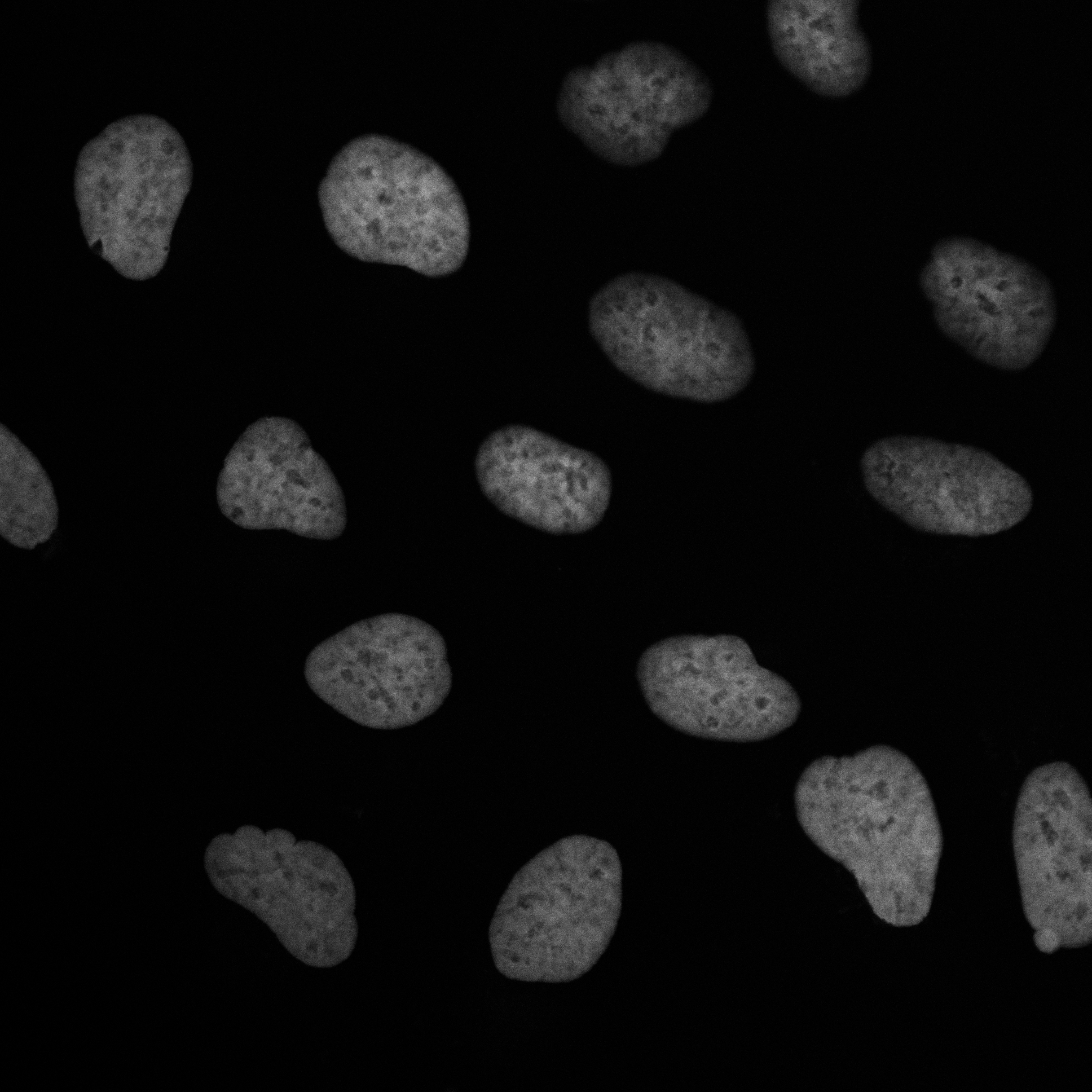

Supplement: Supplementary file 5 — Source data Fig. 5 [file 44318_2026_790_MOESM5_ESM.zip › Figure 5/Figure 5A_pRPA_TelC_U2OS_siFANCM/C1-U2OS_WT_siCTRL_DAPI.tif]

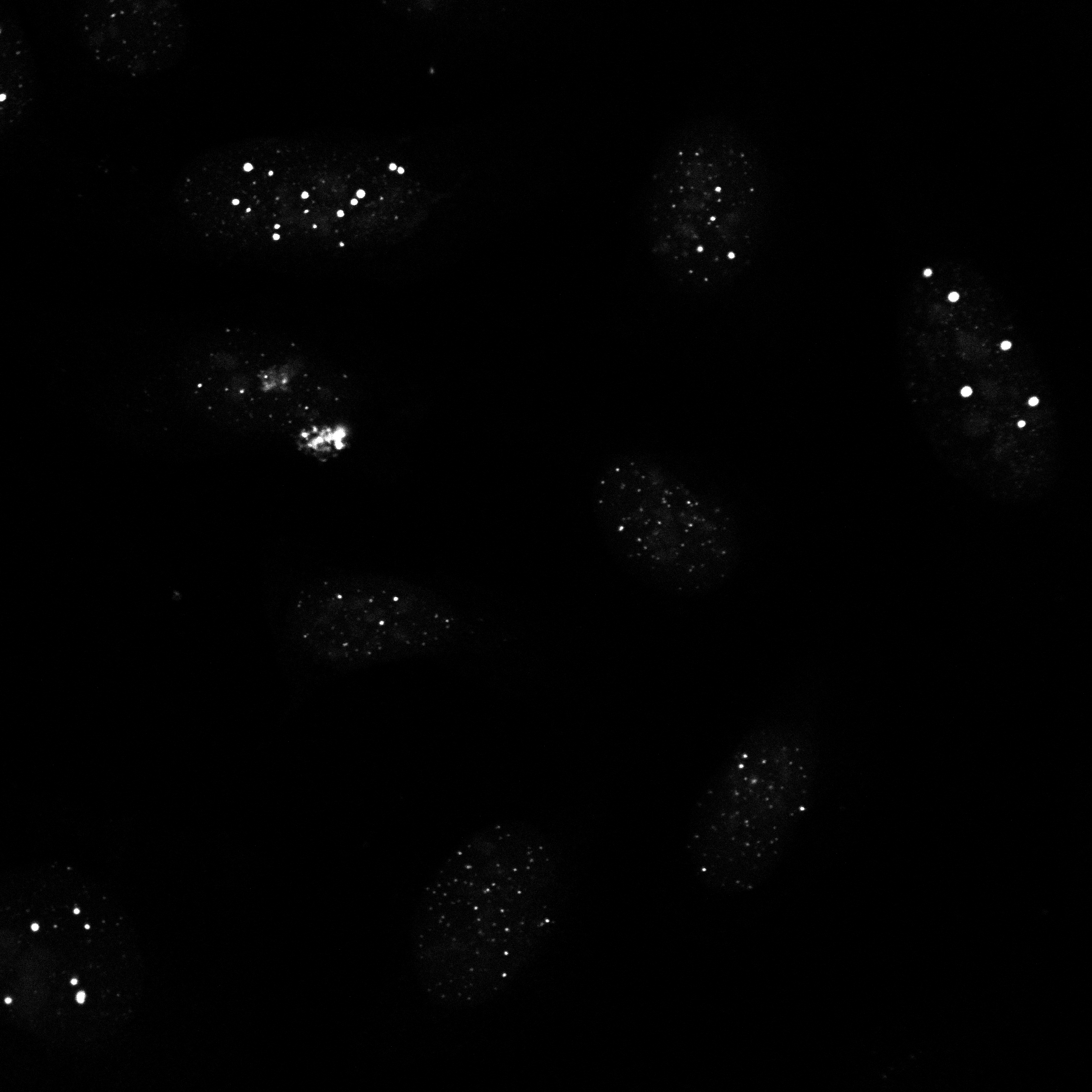

Supplement: Supplementary file 5 — Source data Fig. 5 [file 44318_2026_790_MOESM5_ESM.zip › Figure 5/Figure 5A_pRPA_TelC_U2OS_siFANCM/C3-U2OS_WT_siFANCM_TelC.tif]

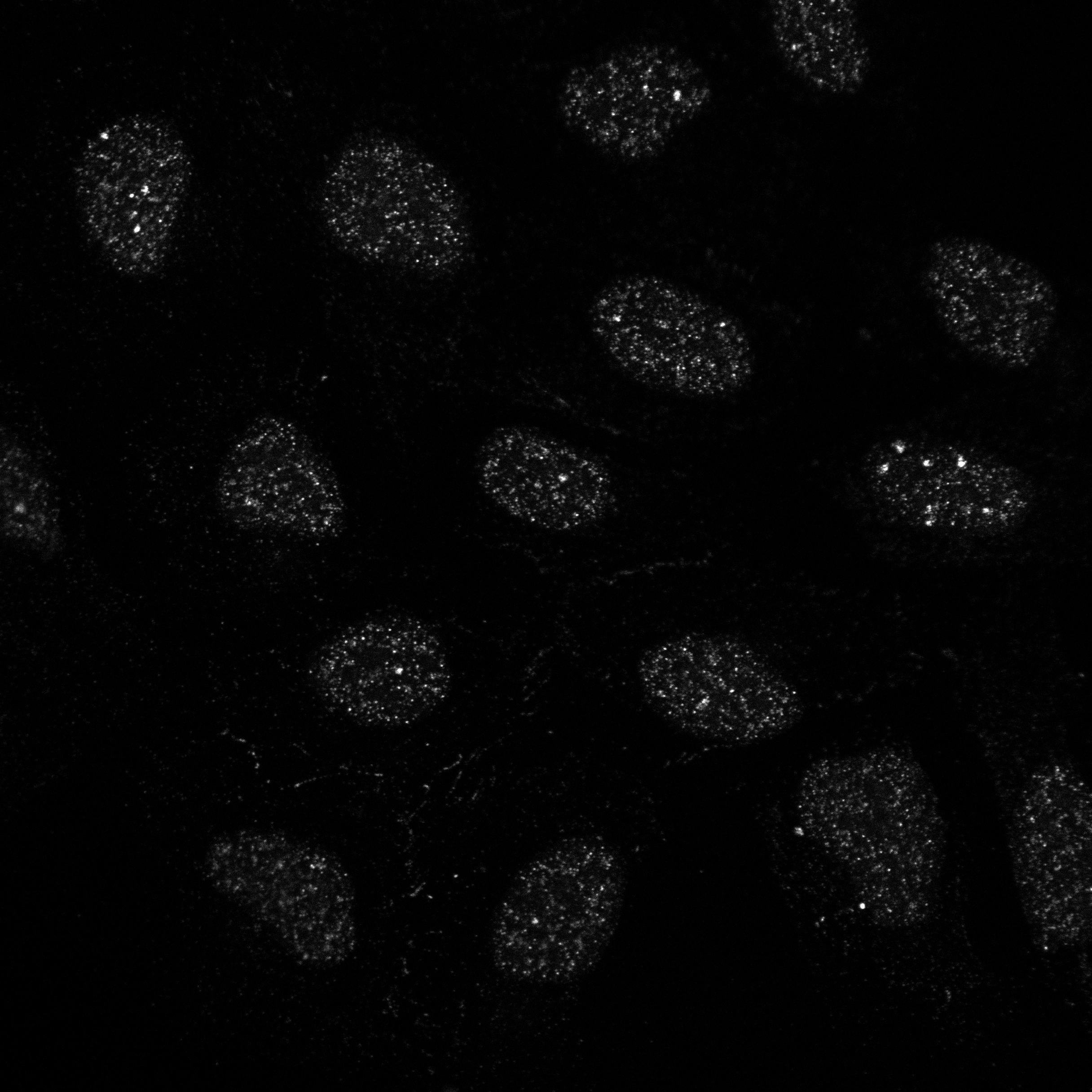

Supplement: Supplementary file 5 — Source data Fig. 5 [file 44318_2026_790_MOESM5_ESM.zip › Figure 5/Figure 5A_pRPA_TelC_U2OS_siFANCM/C2-U2OS_WT_siCTRL_pS33-RPA.tif]

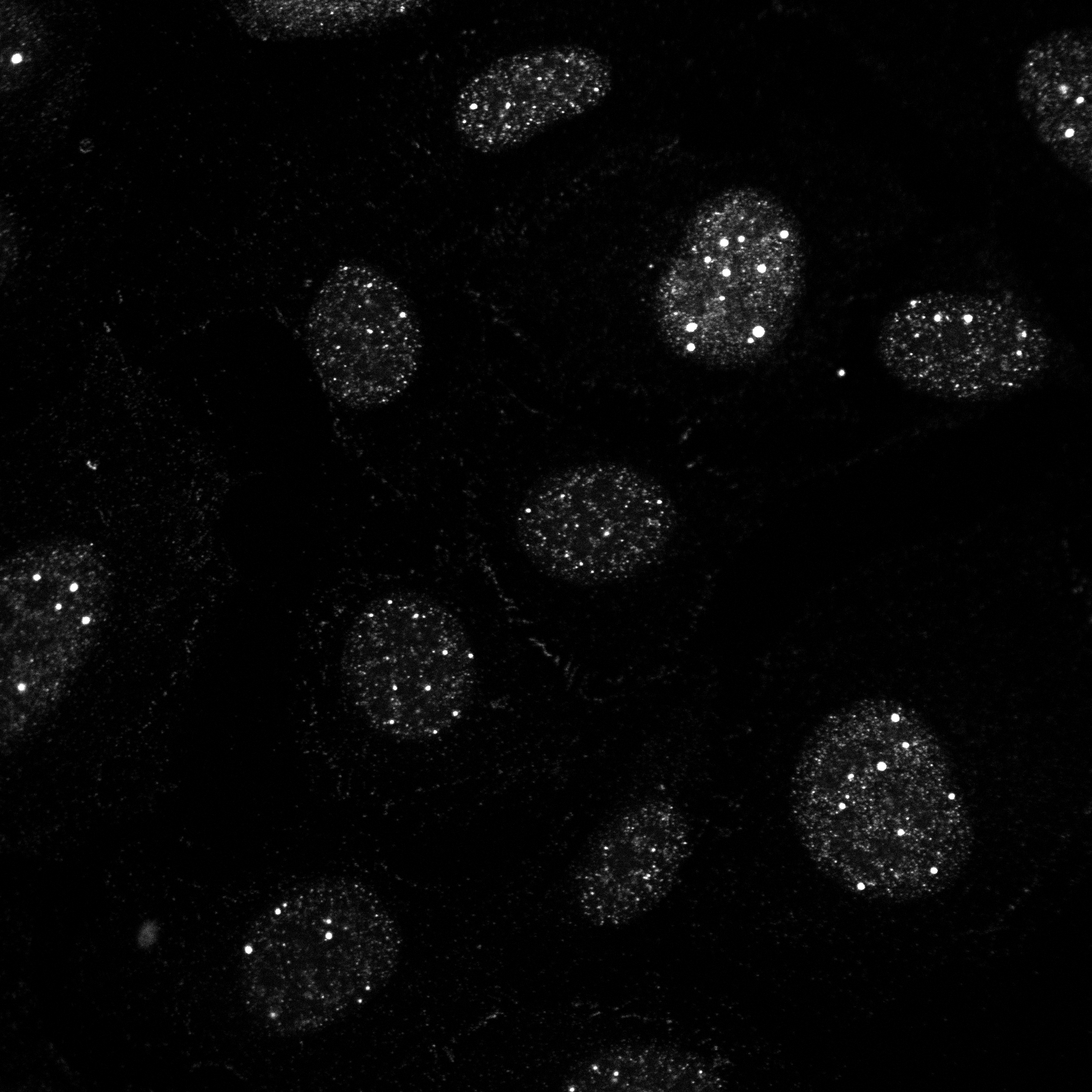

Supplement: Supplementary file 5 — Source data Fig. 5 [file 44318_2026_790_MOESM5_ESM.zip › Figure 5/Figure 5A_pRPA_TelC_U2OS_siFANCM/C2-U2OS_SLX4IP_KO_clone_1_siFANCM_pS33-RPA.tif]

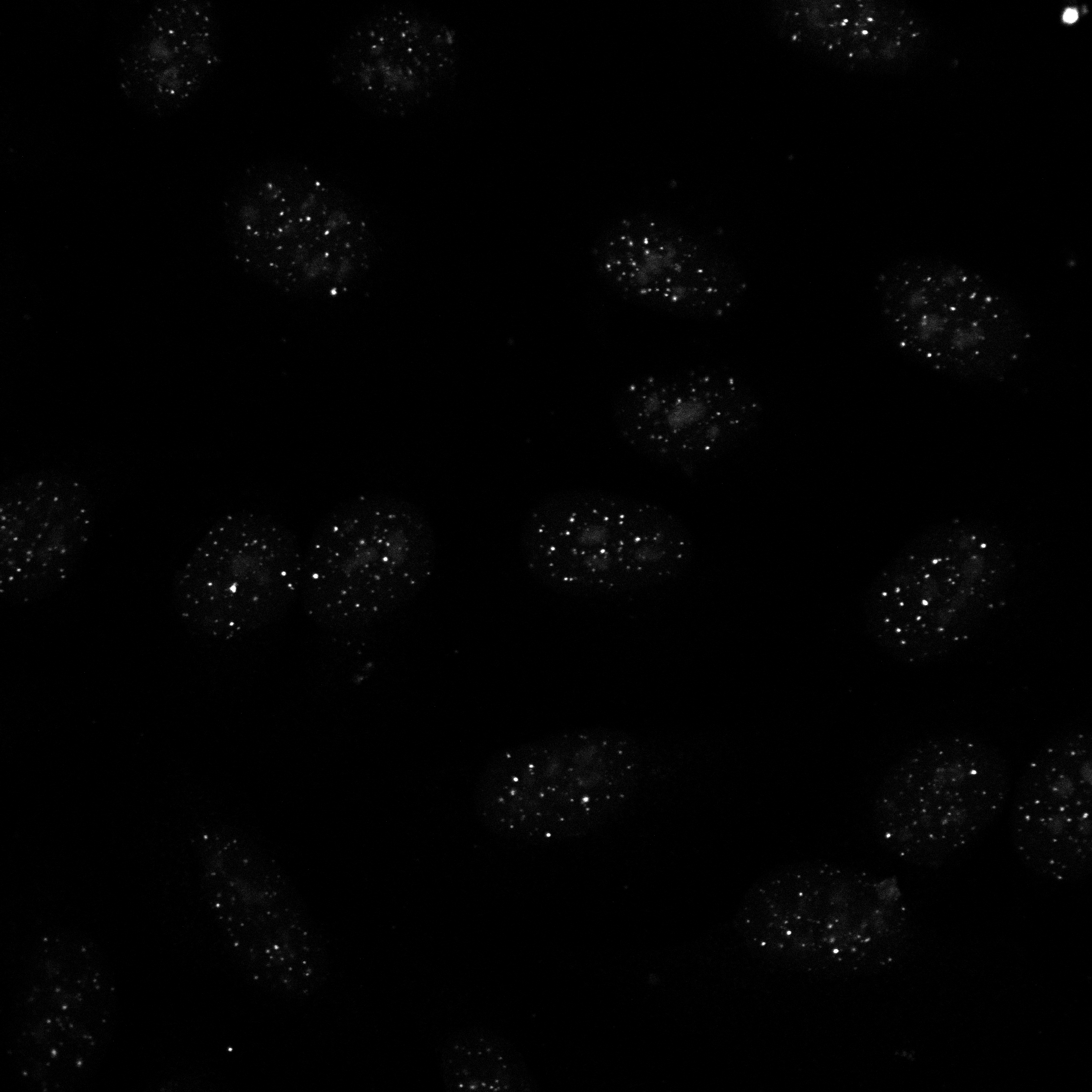

Supplement: Supplementary file 5 — Source data Fig. 5 [file 44318_2026_790_MOESM5_ESM.zip › Figure 5/Figure 5A_pRPA_TelC_U2OS_siFANCM/C3-U2OS_SLX4IP_KO_clone_1_siCTRL_TelC.tif]

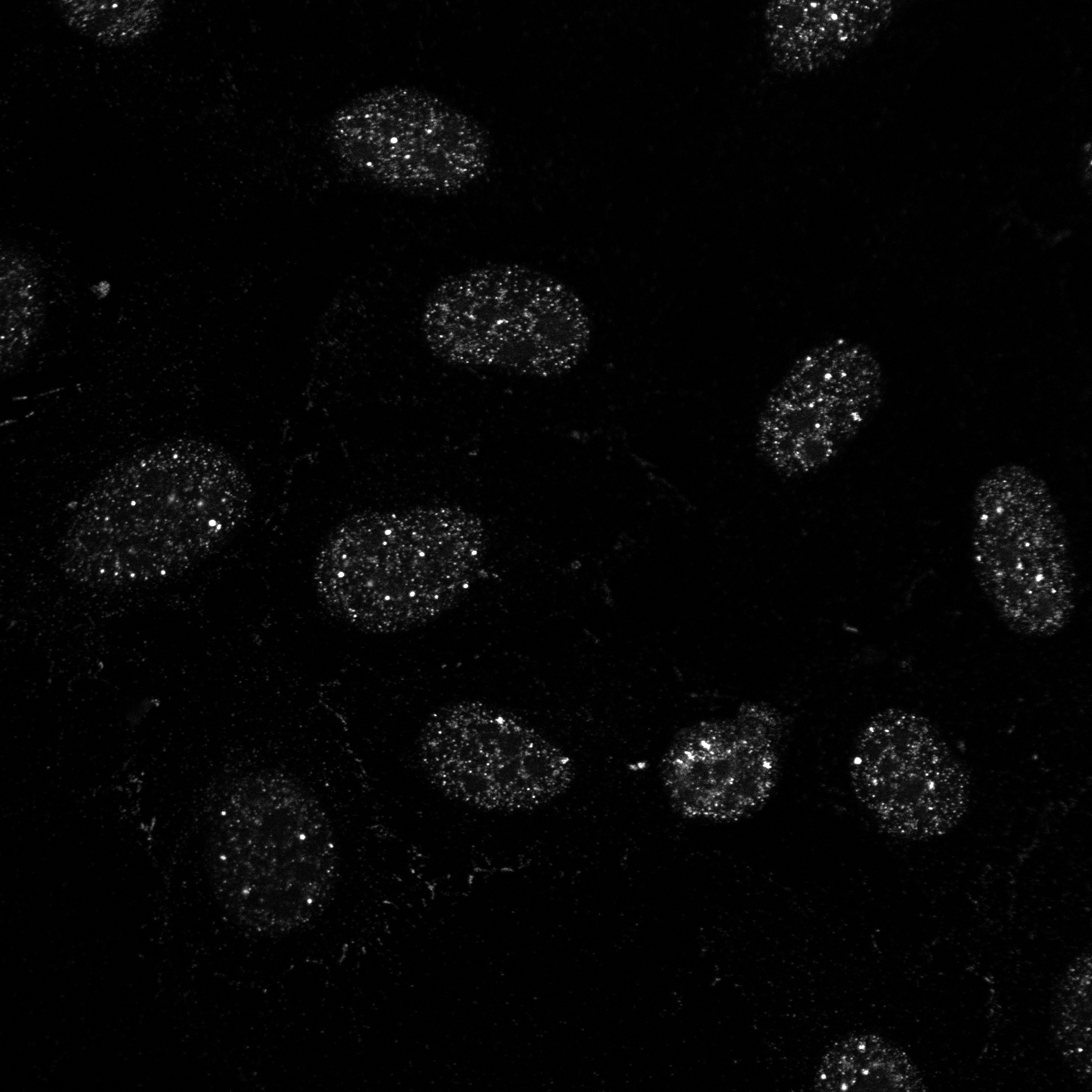

Supplement: Supplementary file 5 — Source data Fig. 5 [file 44318_2026_790_MOESM5_ESM.zip › Figure 5/Figure 5A_pRPA_TelC_U2OS_siFANCM/C2-U2OS_SLX4IP_KO_clone_2_siFANCM_pS33-RPA.tif]

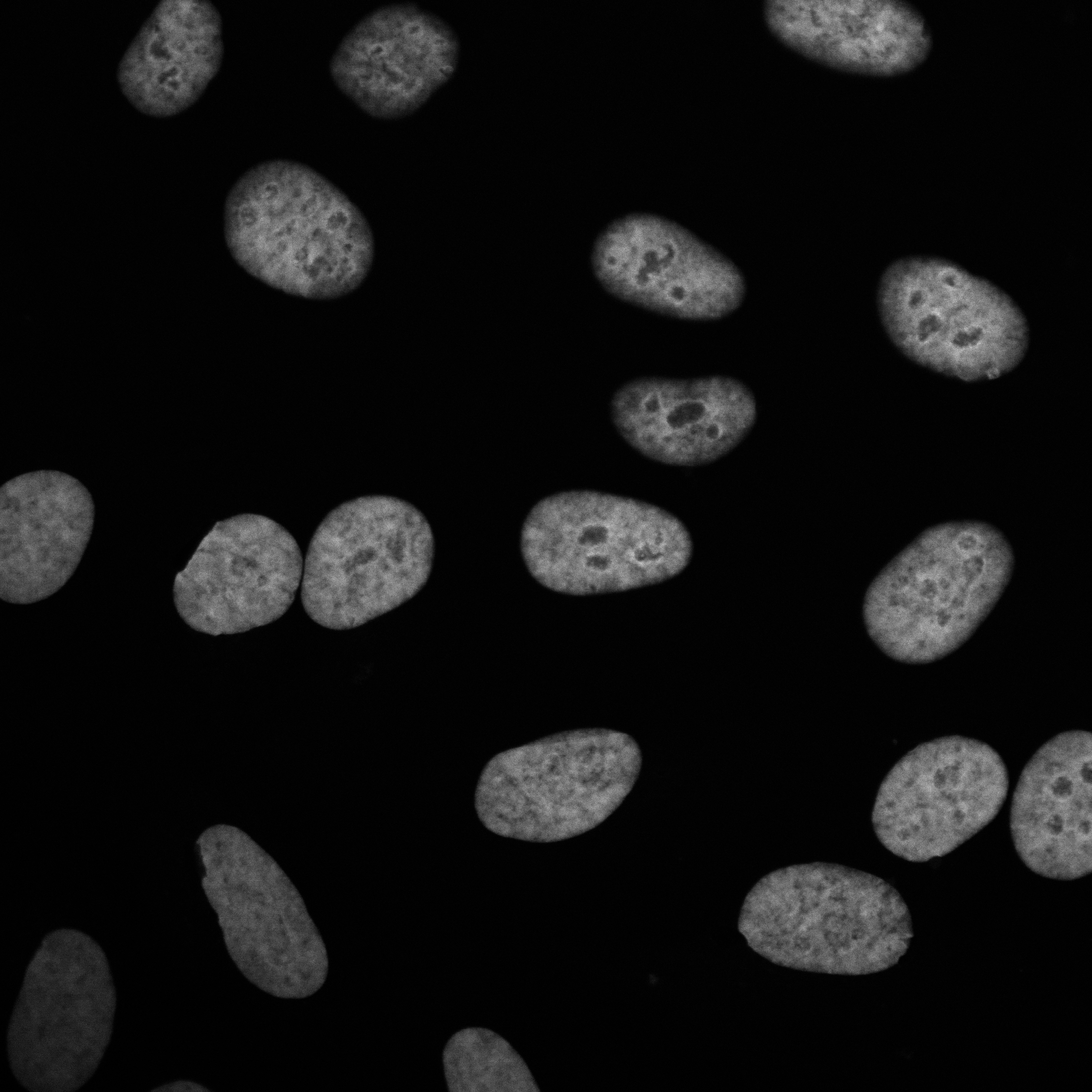

Supplement: Supplementary file 5 — Source data Fig. 5 [file 44318_2026_790_MOESM5_ESM.zip › Figure 5/Figure 5A_pRPA_TelC_U2OS_siFANCM/C1-U2OS_SLX4IP_KO_clone_1_siCTRL_DAPI.tif]

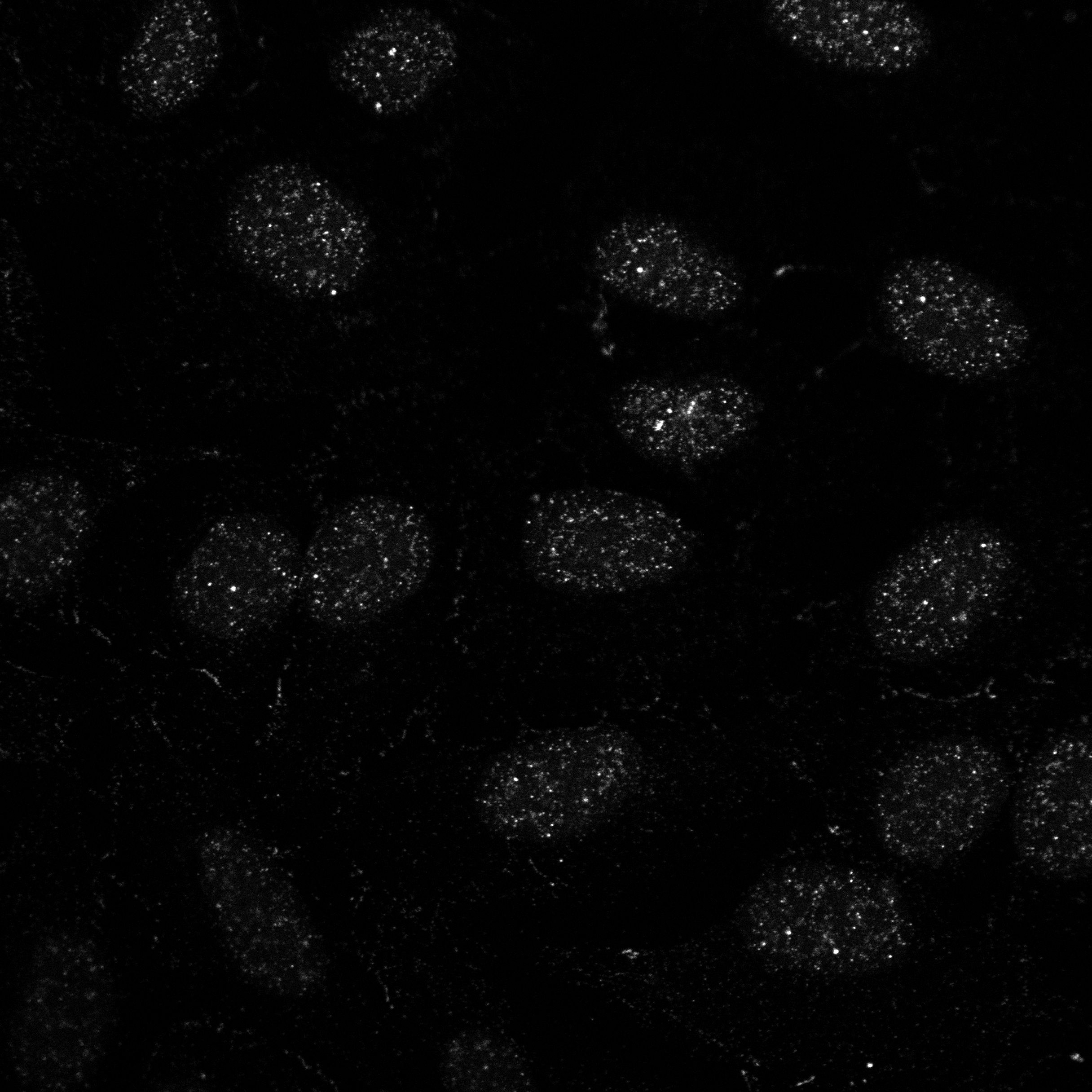

Supplement: Supplementary file 5 — Source data Fig. 5 [file 44318_2026_790_MOESM5_ESM.zip › Figure 5/Figure 5A_pRPA_TelC_U2OS_siFANCM/C2-U2OS_SLX4IP_KO_clone_1_siCTRL_pS33-RPA.tif]

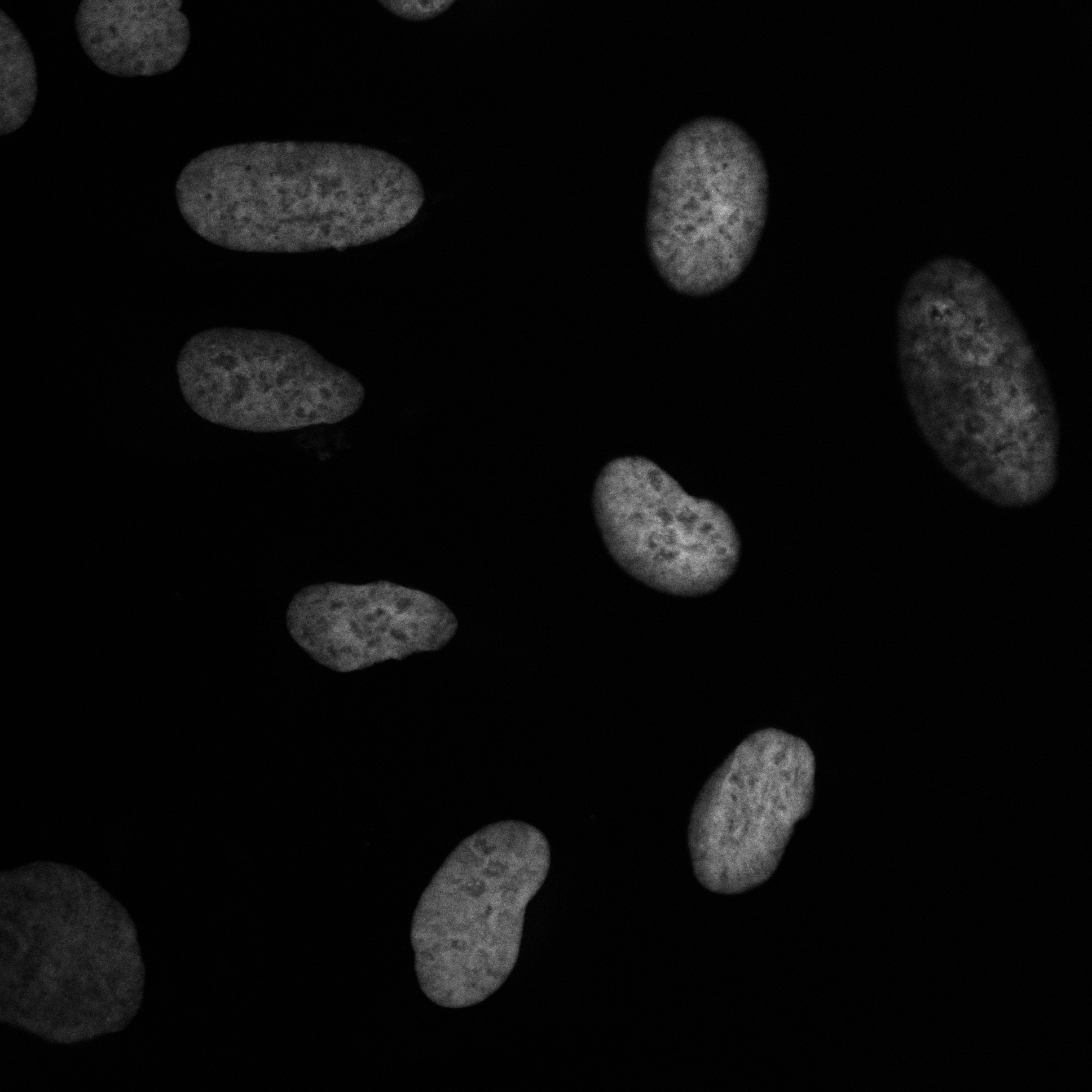

Supplement: Supplementary file 5 — Source data Fig. 5 [file 44318_2026_790_MOESM5_ESM.zip › Figure 5/Figure 5A_pRPA_TelC_U2OS_siFANCM/C1-U2OS_WT_siFANCM_DAPI.tif]

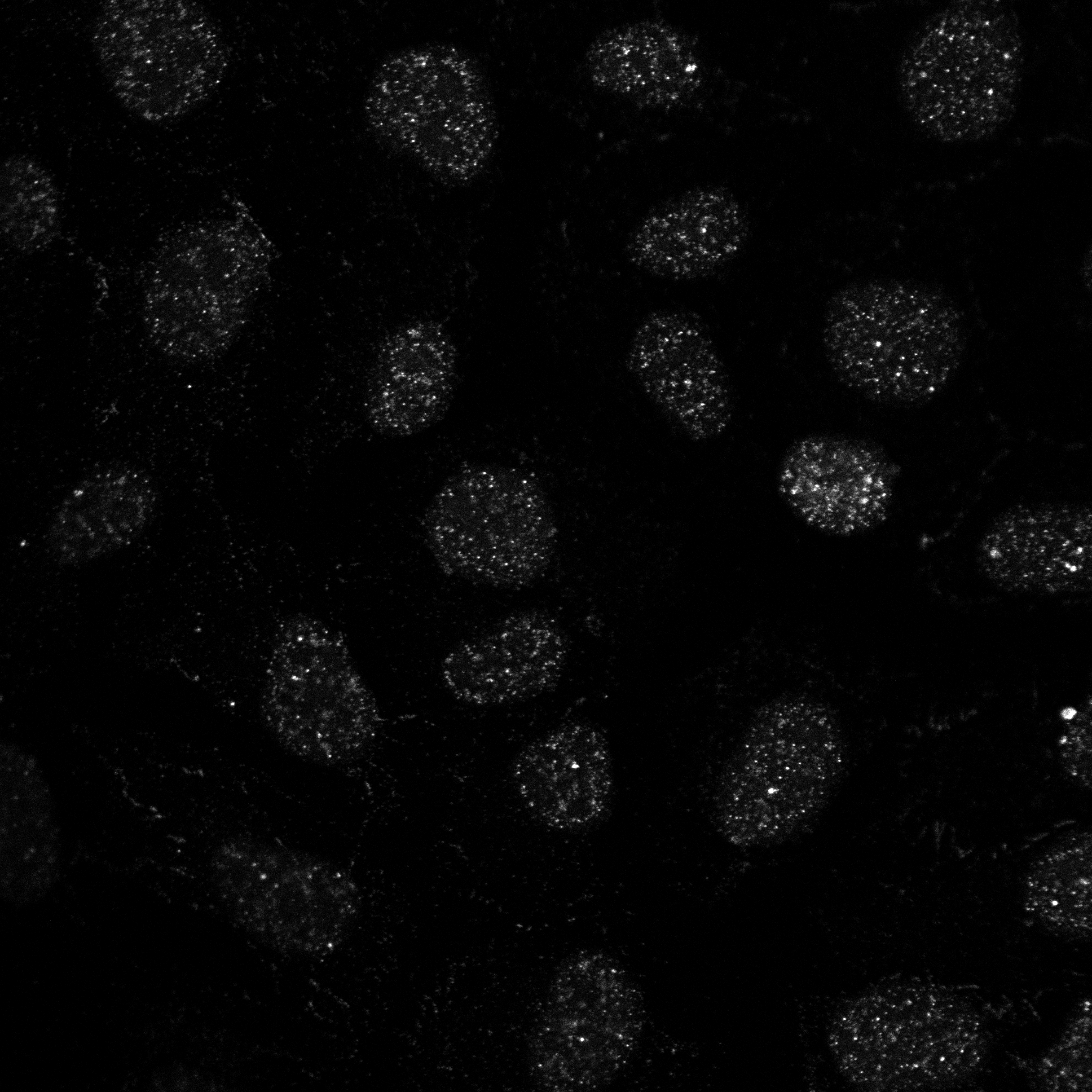

Supplement: Supplementary file 5 — Source data Fig. 5 [file 44318_2026_790_MOESM5_ESM.zip › Figure 5/Figure 5A_pRPA_TelC_U2OS_siFANCM/C2-U2OS_SLX4IP_KO_clone_2_siCTRL_pS33-RPA.tif]

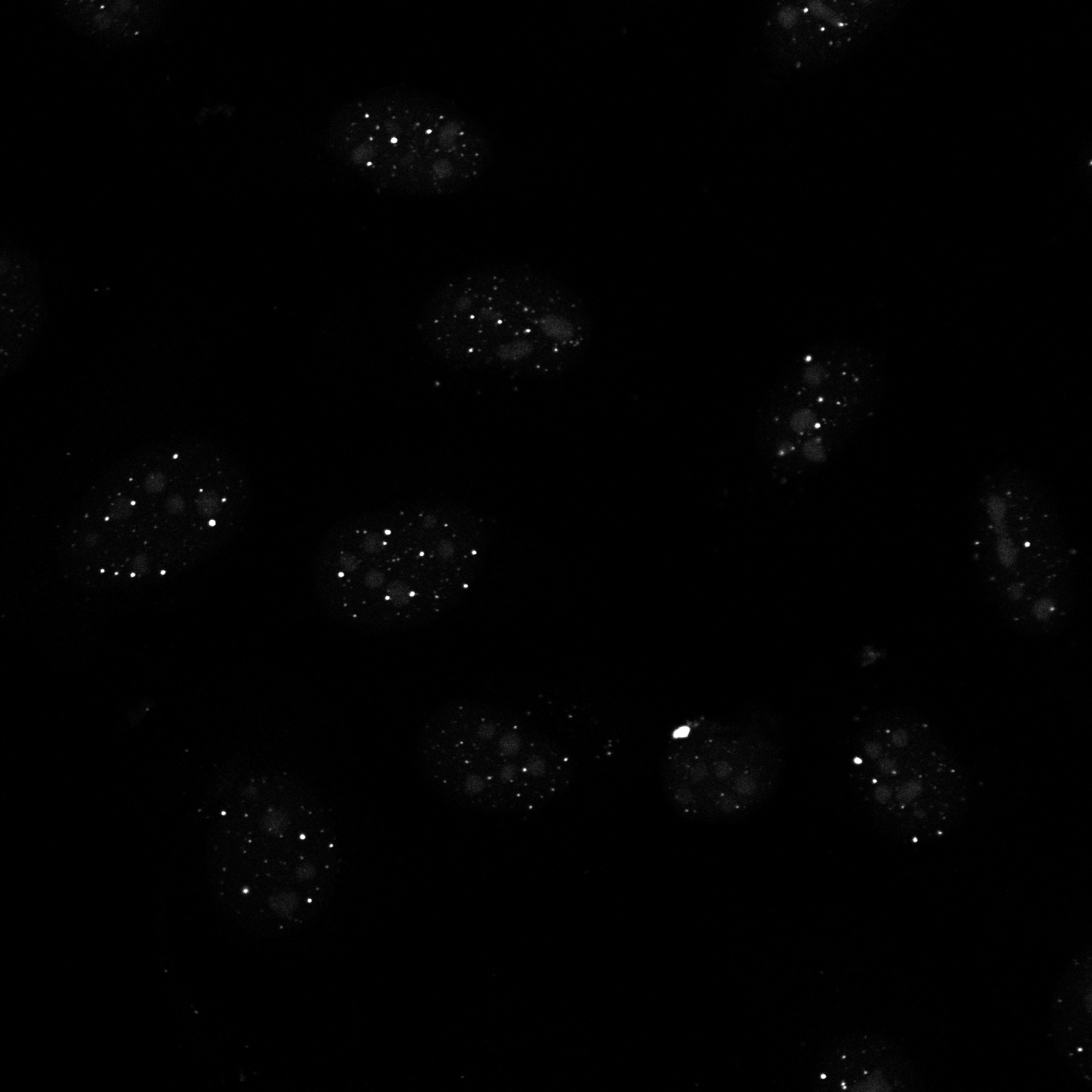

Supplement: Supplementary file 5 — Source data Fig. 5 [file 44318_2026_790_MOESM5_ESM.zip › Figure 5/Figure 5A_pRPA_TelC_U2OS_siFANCM/C3-U2OS_SLX4IP_KO_clone_2_siFANCM_TelC.tif]

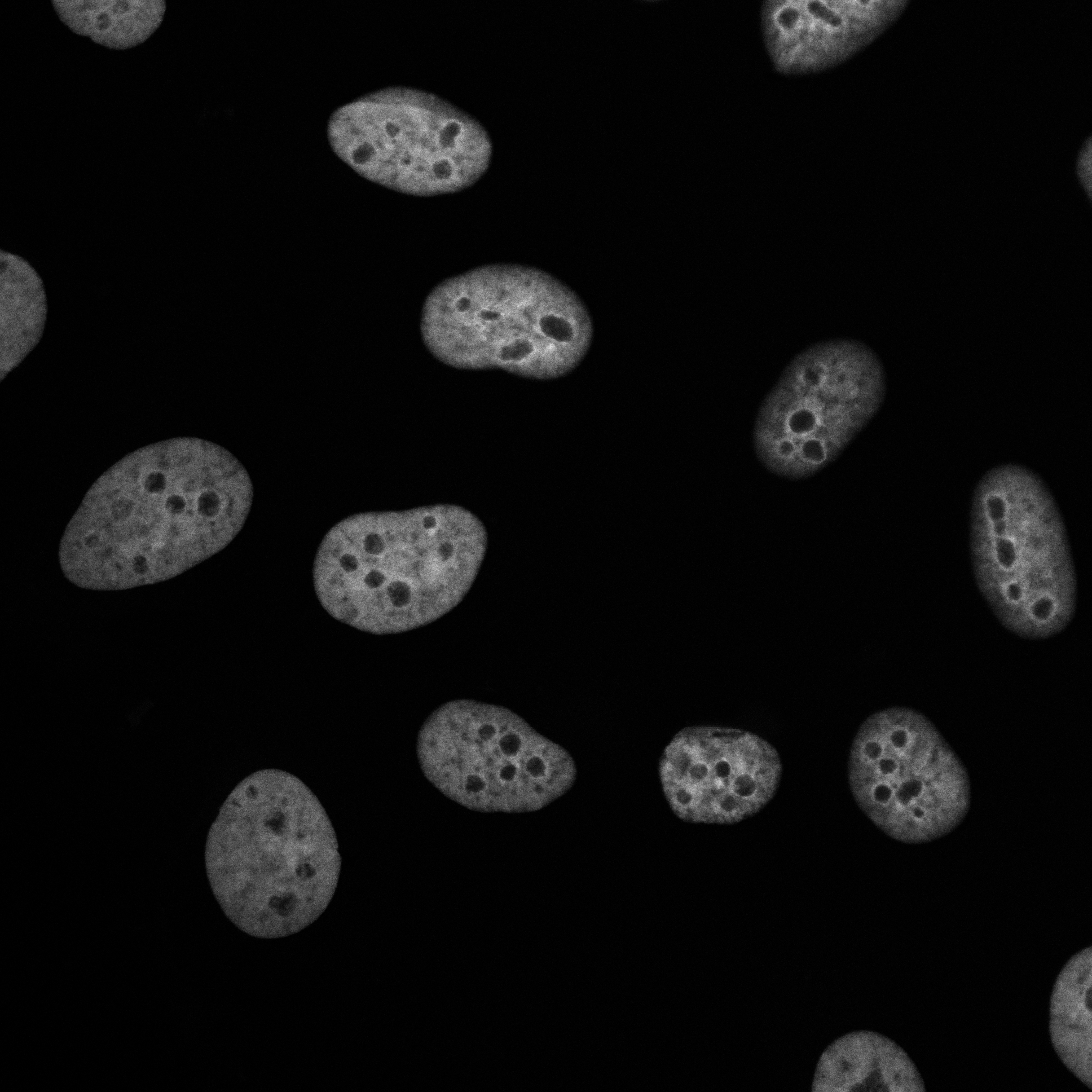

Supplement: Supplementary file 5 — Source data Fig. 5 [file 44318_2026_790_MOESM5_ESM.zip › Figure 5/Figure 5A_pRPA_TelC_U2OS_siFANCM/C1-U2OS_SLX4IP_KO_clone_2_siFANCM_DAPI.tif]

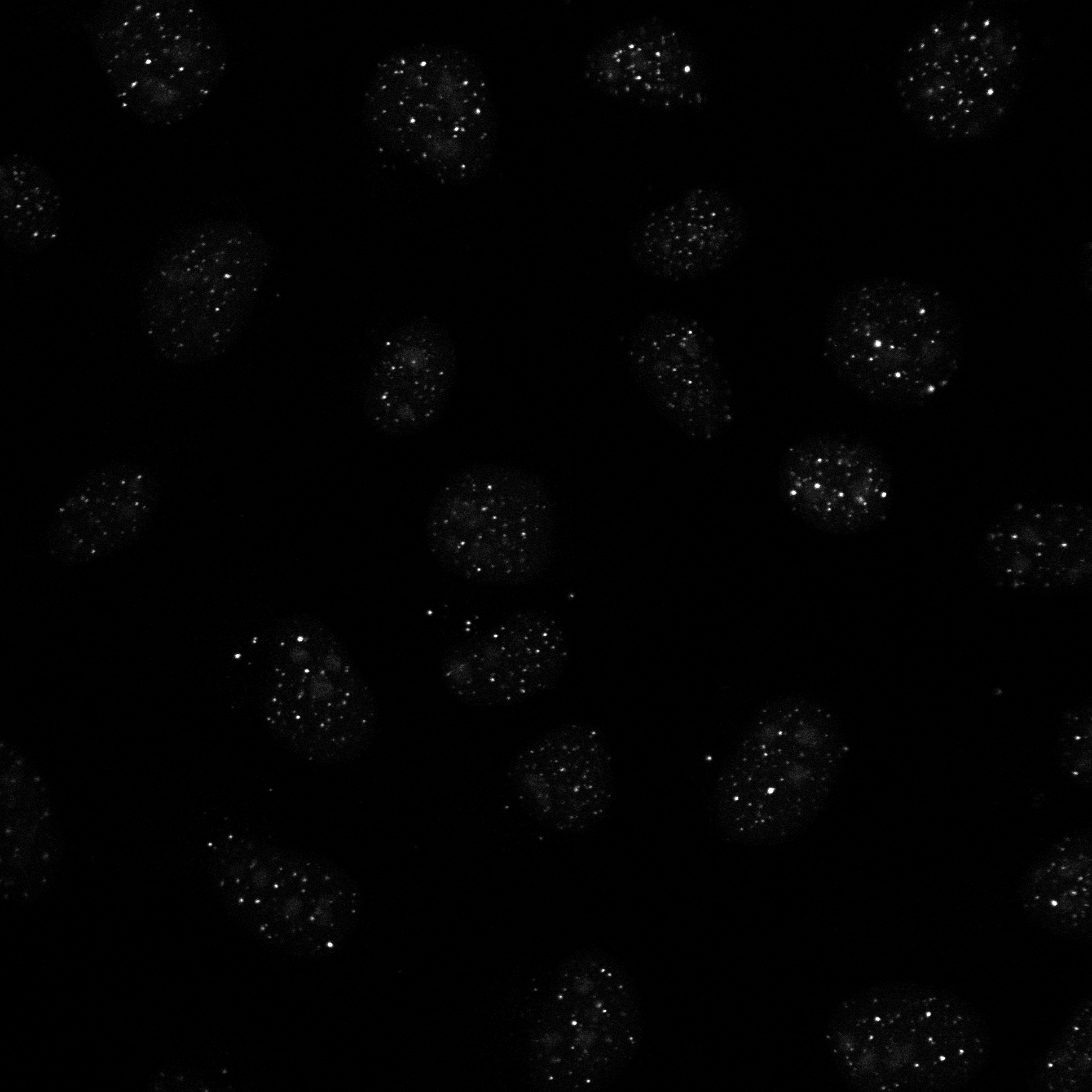

Supplement: Supplementary file 5 — Source data Fig. 5 [file 44318_2026_790_MOESM5_ESM.zip › Figure 5/Figure 5A_pRPA_TelC_U2OS_siFANCM/C3-U2OS_SLX4IP_KO_clone_2_siCTRL_TelC.tif]

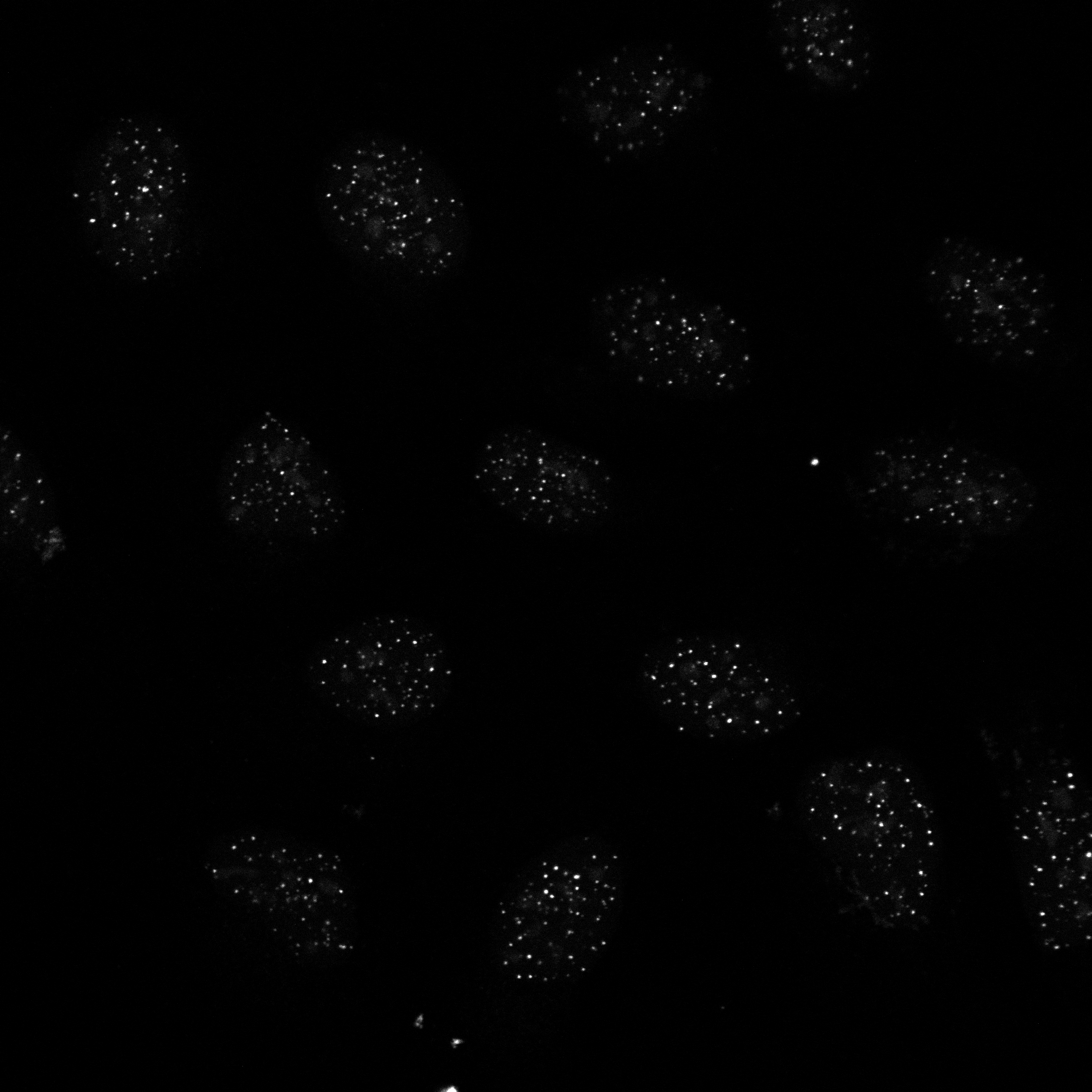

Supplement: Supplementary file 5 — Source data Fig. 5 [file 44318_2026_790_MOESM5_ESM.zip › Figure 5/Figure 5A_pRPA_TelC_U2OS_siFANCM/C3-U2OS_WT_siCTRL_TelC.tif]

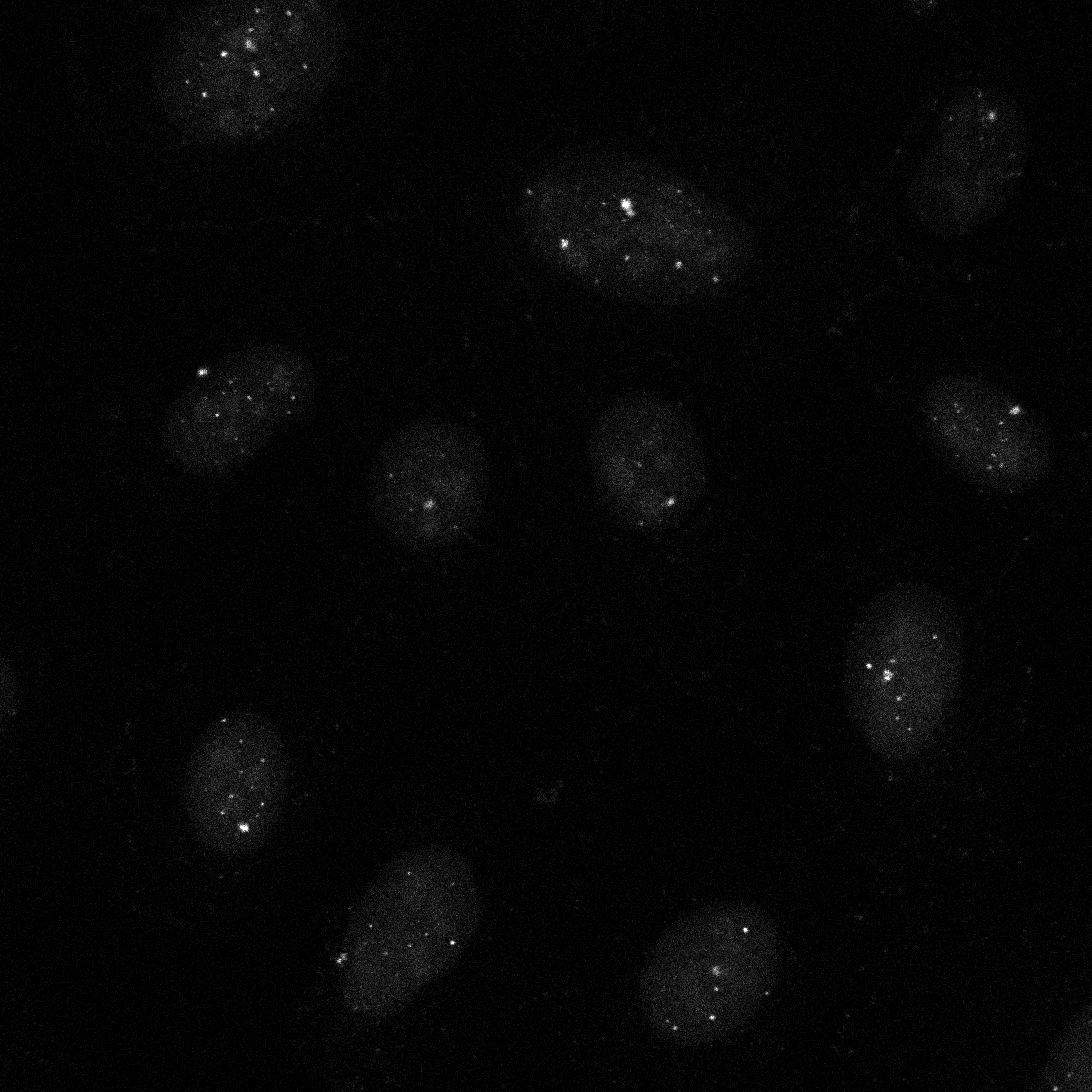

Supplement: Supplementary file 5 — Source data Fig. 5 [file 44318_2026_790_MOESM5_ESM.zip › Figure 5/Figure 5C_pCHK1_TelC_U2OS_siFANCM/C2-U2OS_KO_clone_1_siFANCM_pS345-CHK1.tif]

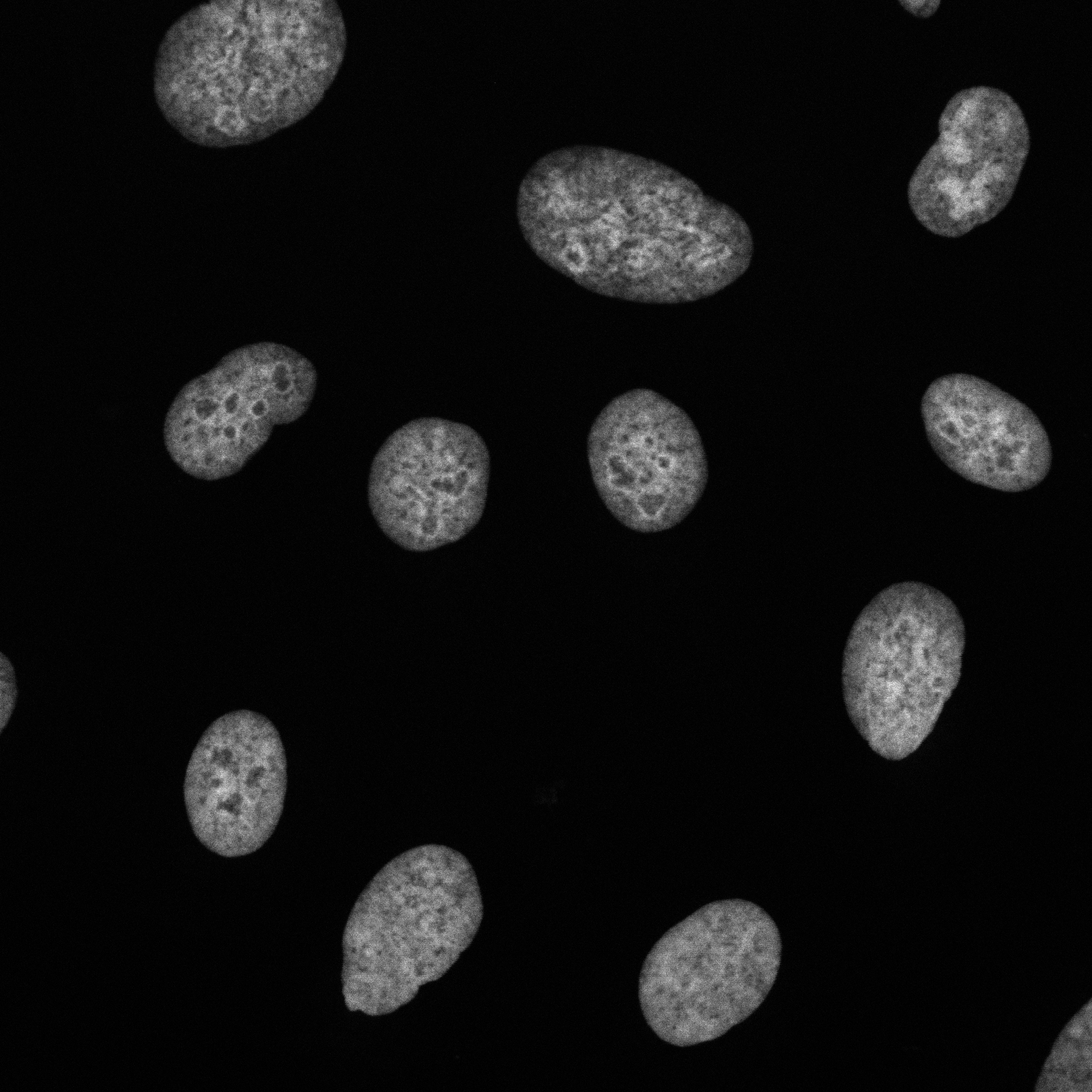

Supplement: Supplementary file 5 — Source data Fig. 5 [file 44318_2026_790_MOESM5_ESM.zip › Figure 5/Figure 5C_pCHK1_TelC_U2OS_siFANCM/C1-U2OS_KO_clone_1_siFANCM_DAPI.tif]

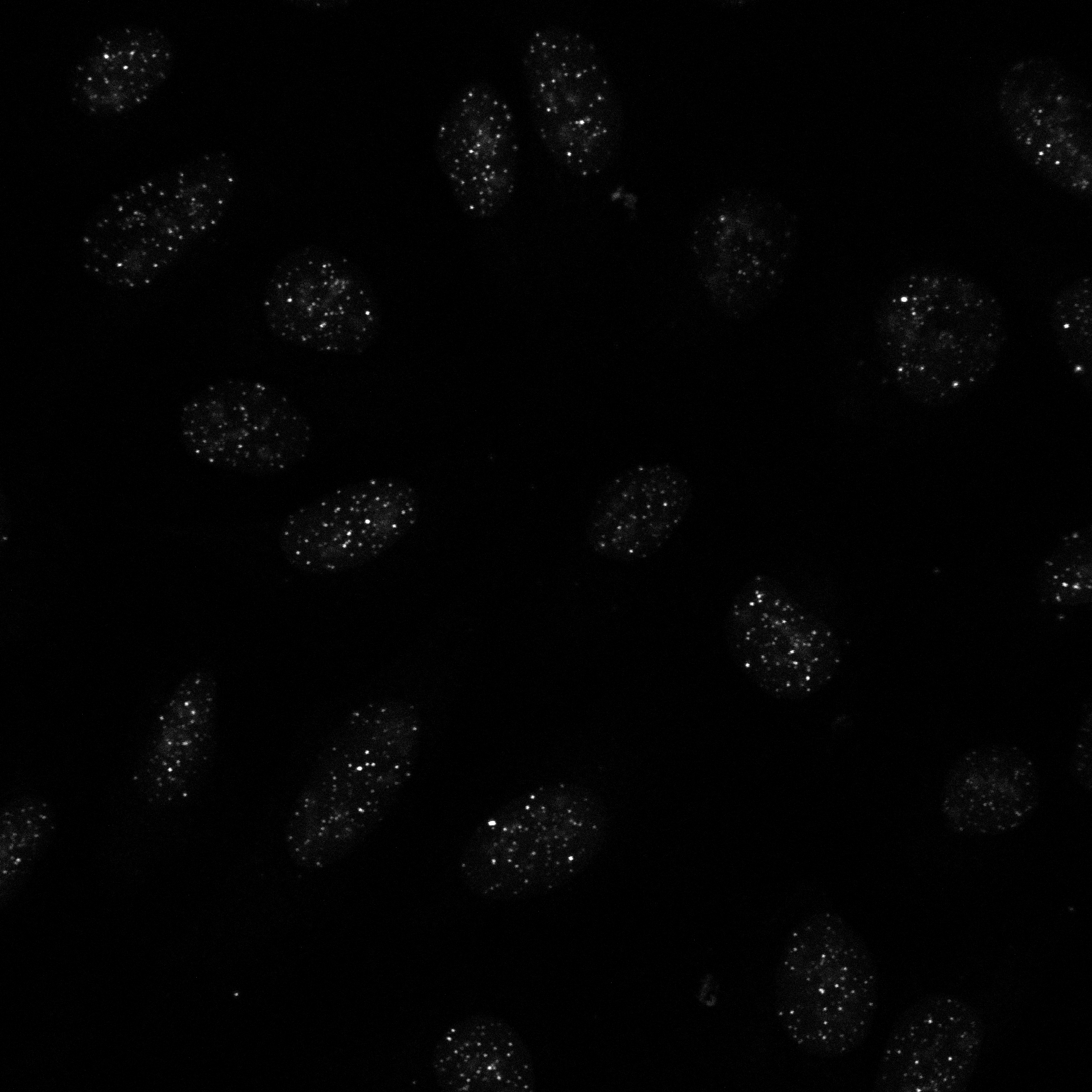

Supplement: Supplementary file 5 — Source data Fig. 5 [file 44318_2026_790_MOESM5_ESM.zip › Figure 5/Figure 5C_pCHK1_TelC_U2OS_siFANCM/C3-U2OS_KO_clone_1_siCTRL_TelC.tif]

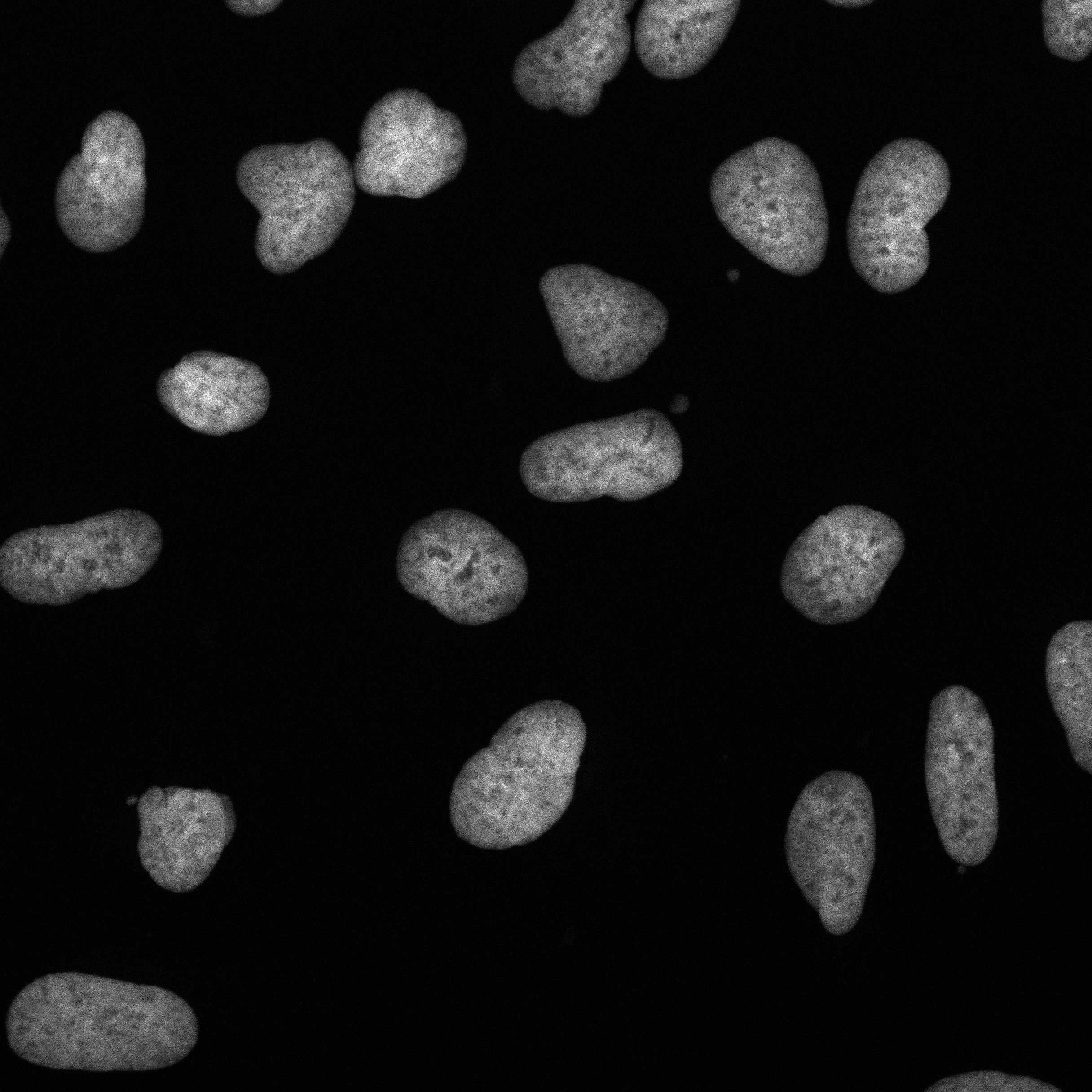

Supplement: Supplementary file 5 — Source data Fig. 5 [file 44318_2026_790_MOESM5_ESM.zip › Figure 5/Figure 5C_pCHK1_TelC_U2OS_siFANCM/C1-U2OS_WT_siCTRL_DAPI.tif]

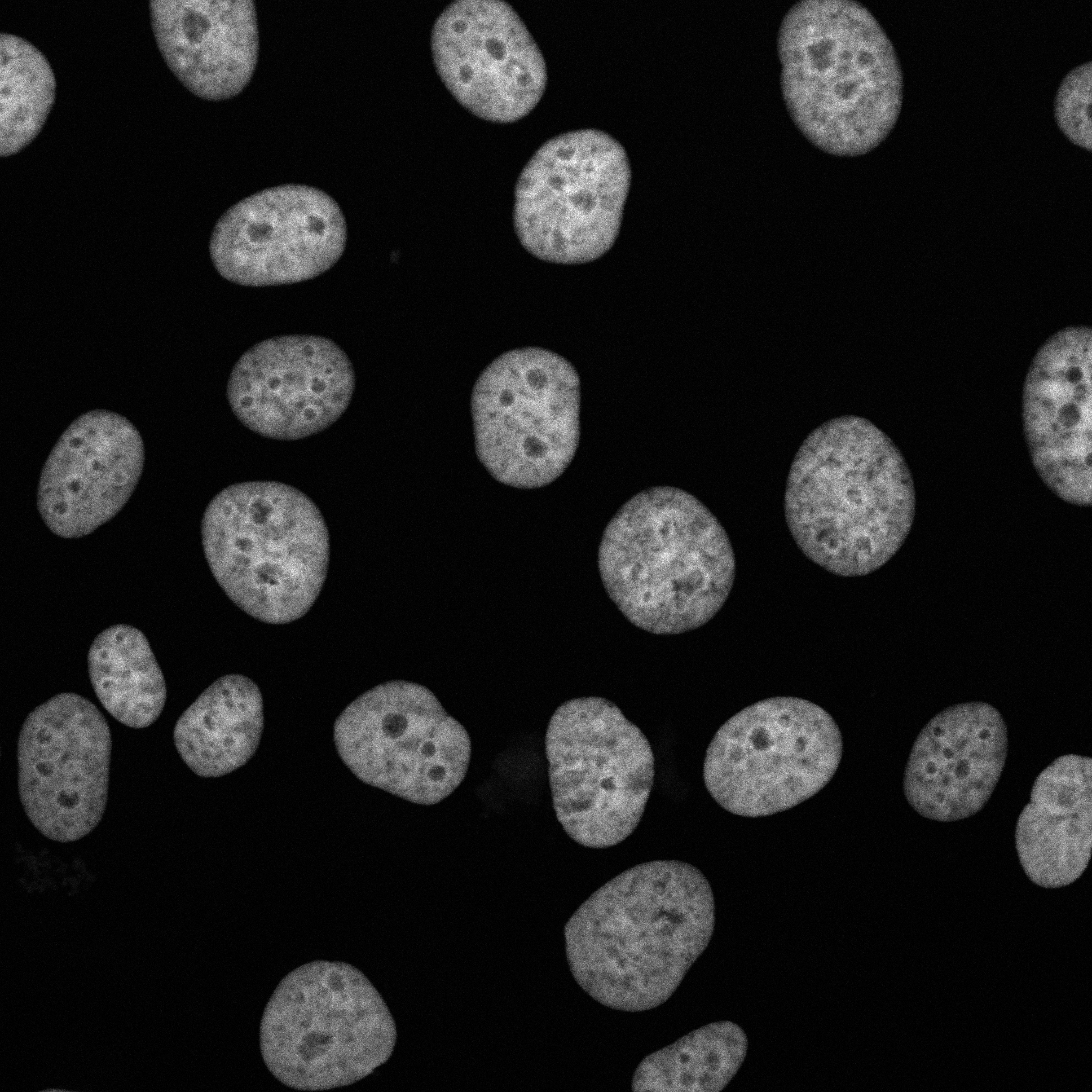

Supplement: Supplementary file 5 — Source data Fig. 5 [file 44318_2026_790_MOESM5_ESM.zip › Figure 5/Figure 5C_pCHK1_TelC_U2OS_siFANCM/C1-U2OS_KO_clone_2_siCTRL_DAPI.tif]

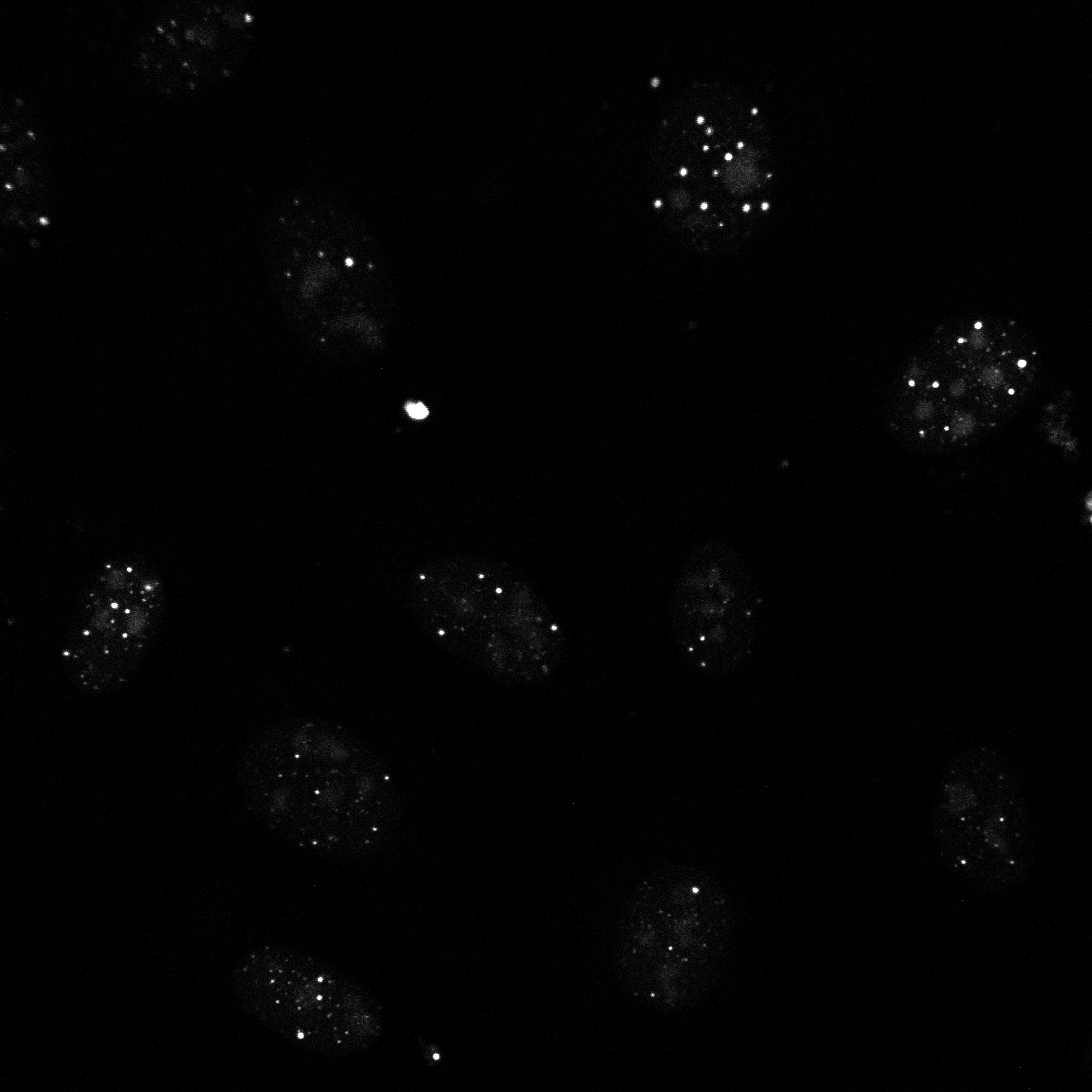

Supplement: Supplementary file 5 — Source data Fig. 5 [file 44318_2026_790_MOESM5_ESM.zip › Figure 5/Figure 5C_pCHK1_TelC_U2OS_siFANCM/C3-U2OS_KO_clone_2_siFANCM_TelC.tif]

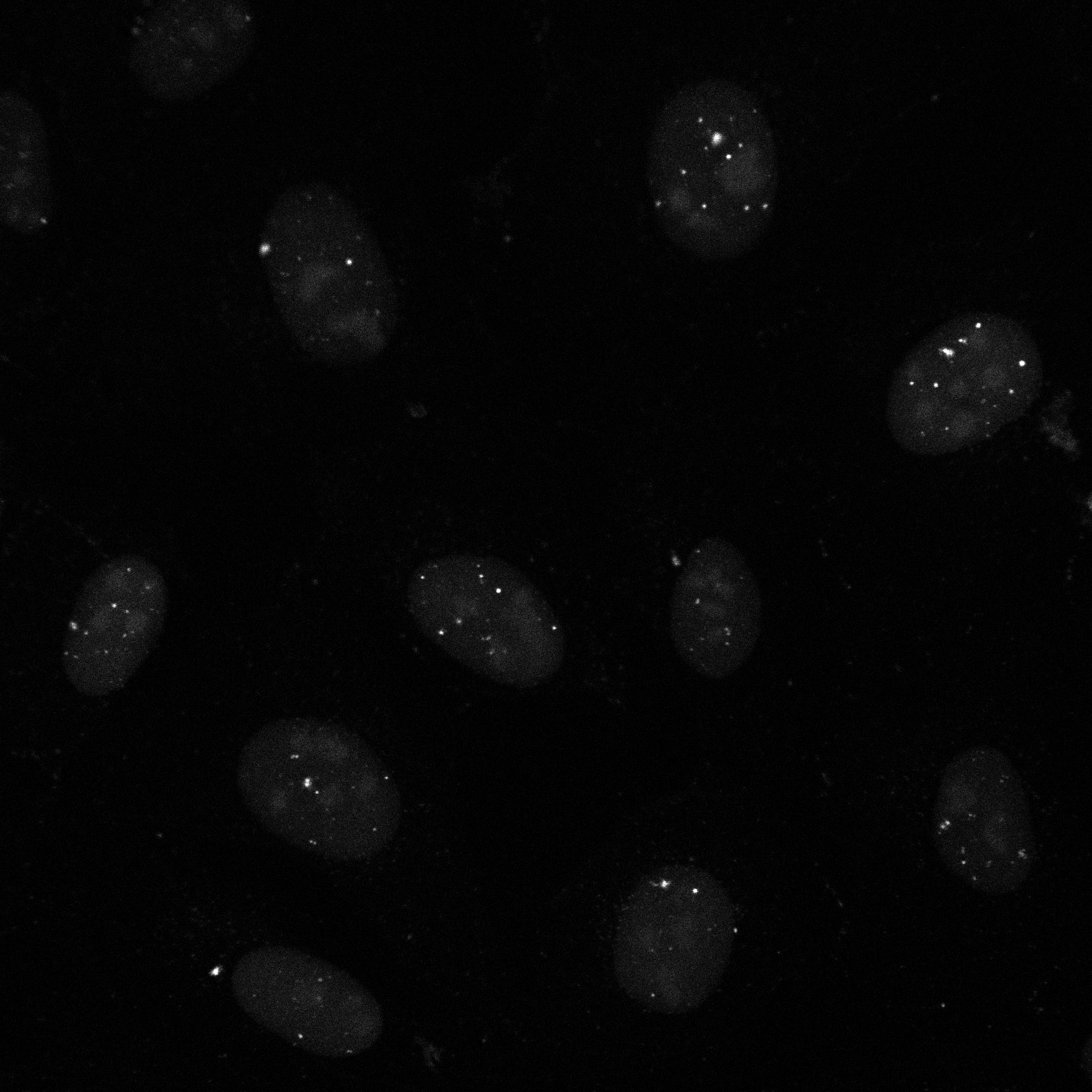

Supplement: Supplementary file 5 — Source data Fig. 5 [file 44318_2026_790_MOESM5_ESM.zip › Figure 5/Figure 5C_pCHK1_TelC_U2OS_siFANCM/C2-U2OS_KO_clone_2_siFANCM_pS345-CHK1.tif]

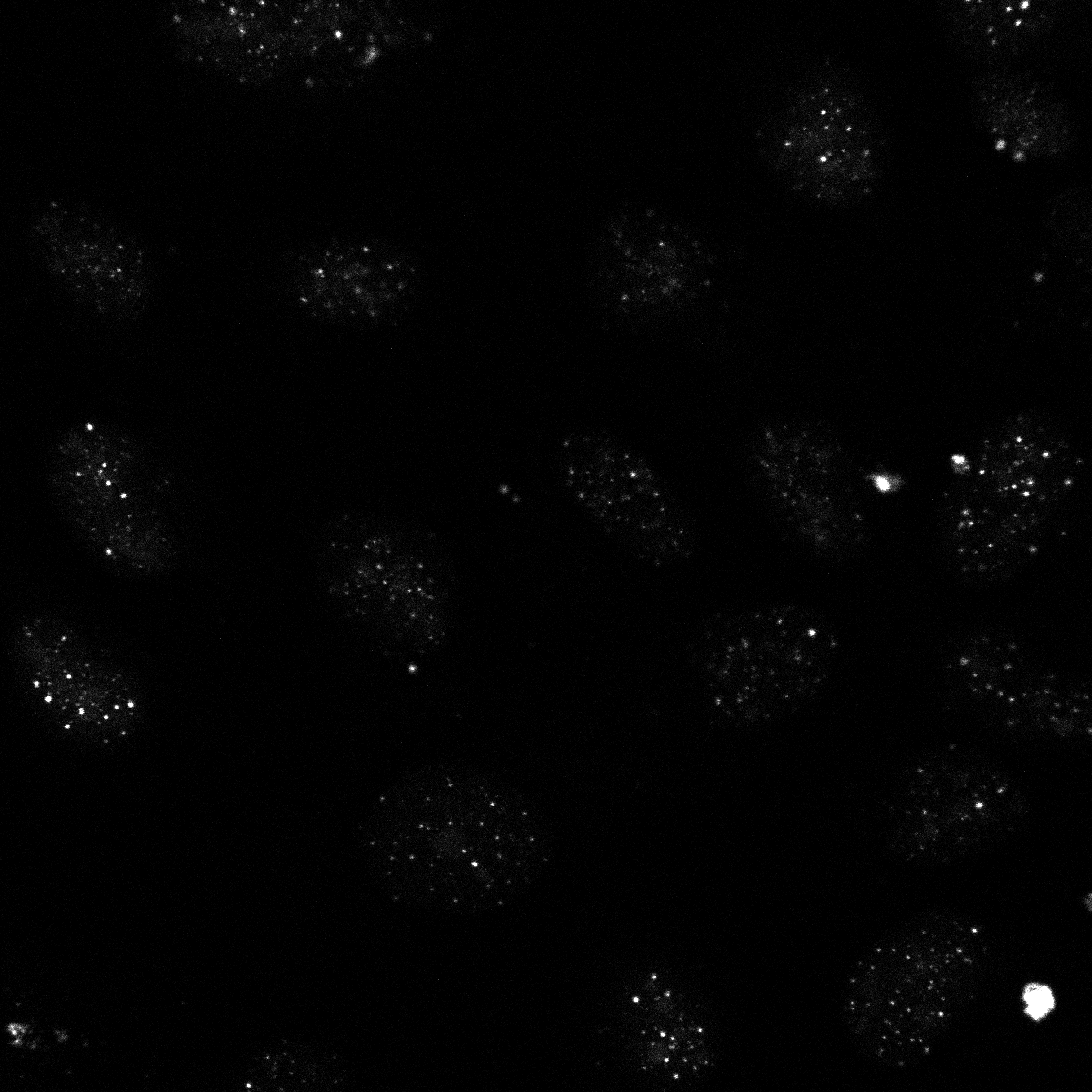

Supplement: Supplementary file 5 — Source data Fig. 5 [file 44318_2026_790_MOESM5_ESM.zip › Figure 5/Figure 5C_pCHK1_TelC_U2OS_siFANCM/C3-U2OS_WT_siFANCM_TelC.tif]

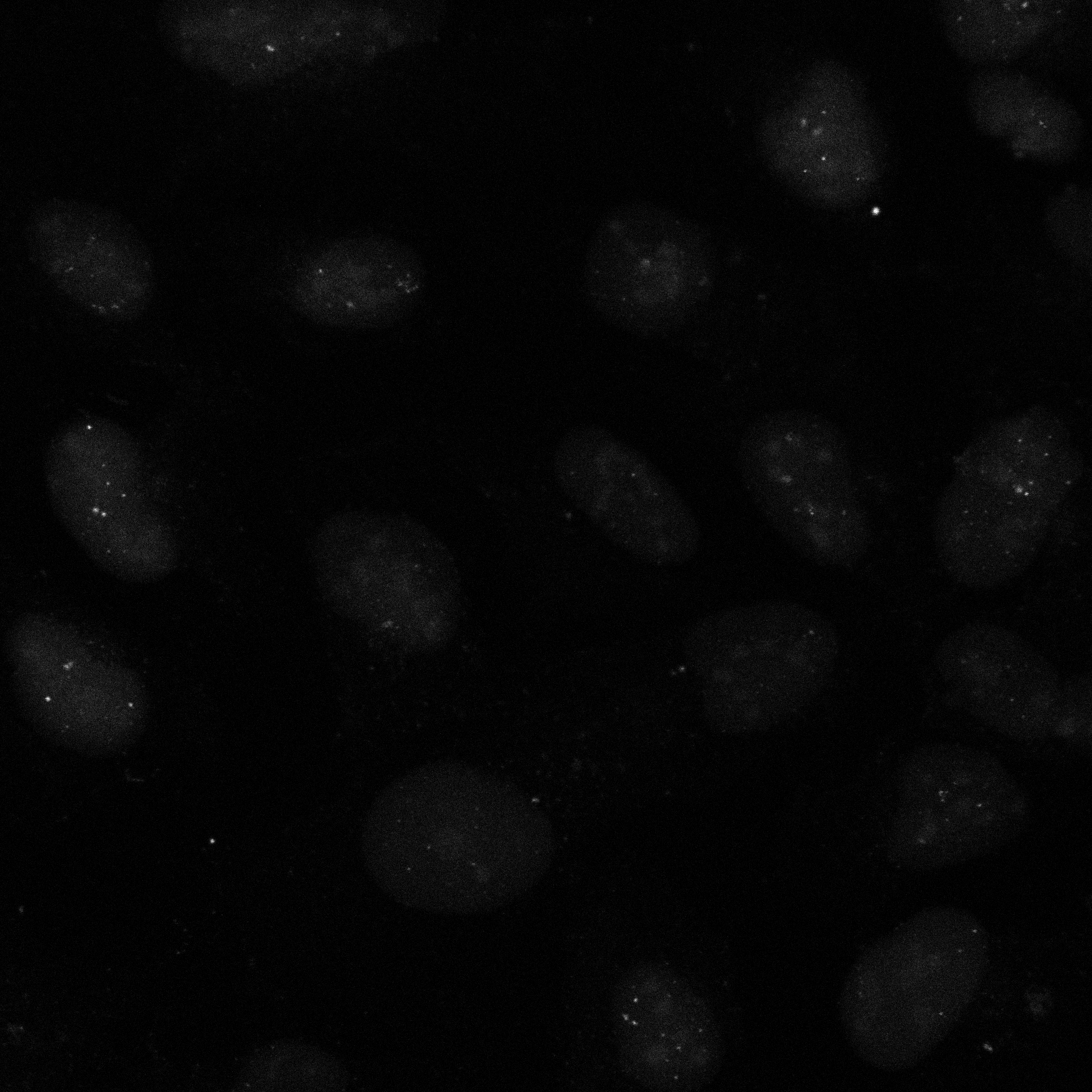

Supplement: Supplementary file 5 — Source data Fig. 5 [file 44318_2026_790_MOESM5_ESM.zip › Figure 5/Figure 5C_pCHK1_TelC_U2OS_siFANCM/C2-U2OS_WT_siFANCM_pS345-CHK1.tif]

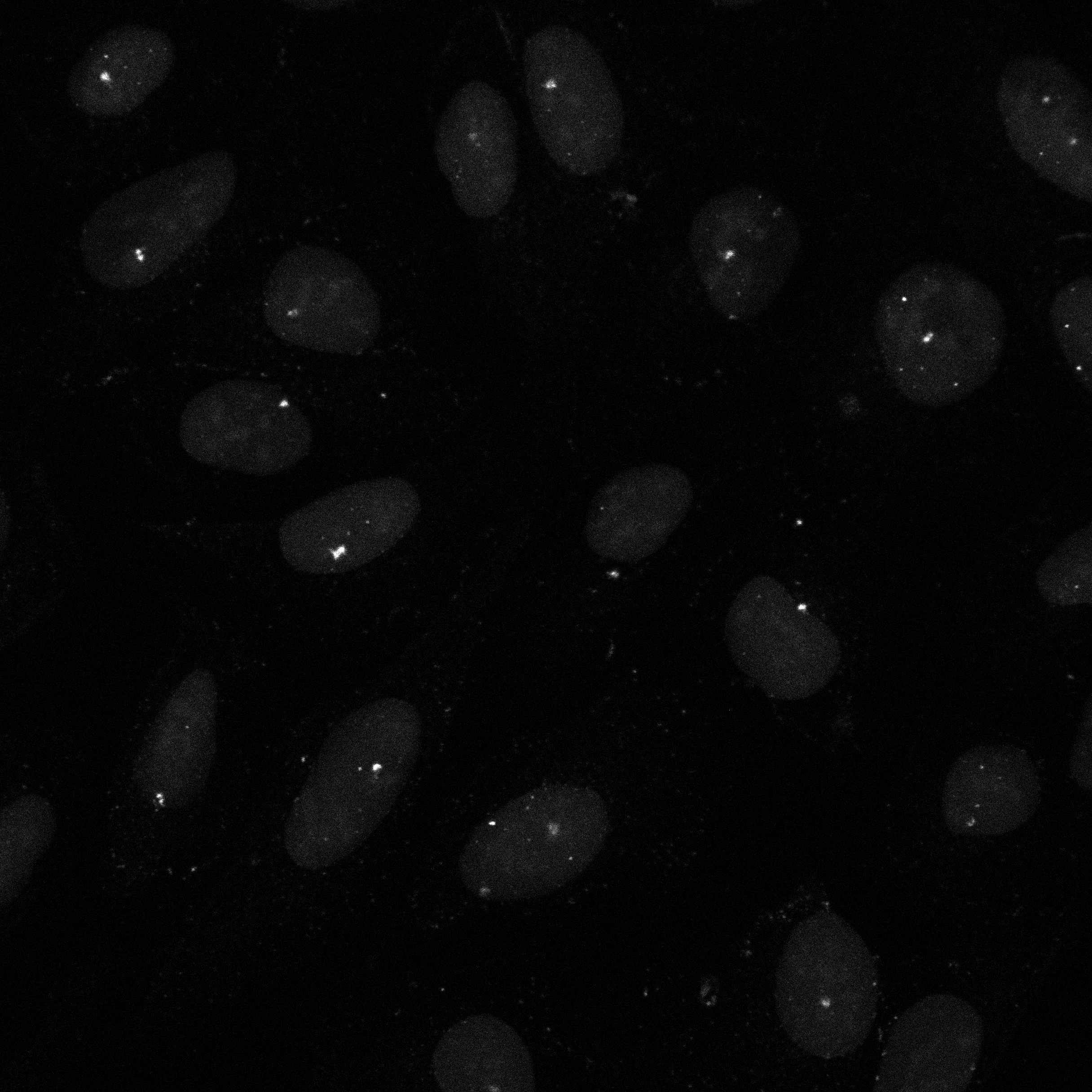

Supplement: Supplementary file 5 — Source data Fig. 5 [file 44318_2026_790_MOESM5_ESM.zip › Figure 5/Figure 5C_pCHK1_TelC_U2OS_siFANCM/C2-U2OS_KO_clone_1_siCTRL_pS345-CHK1.tif]

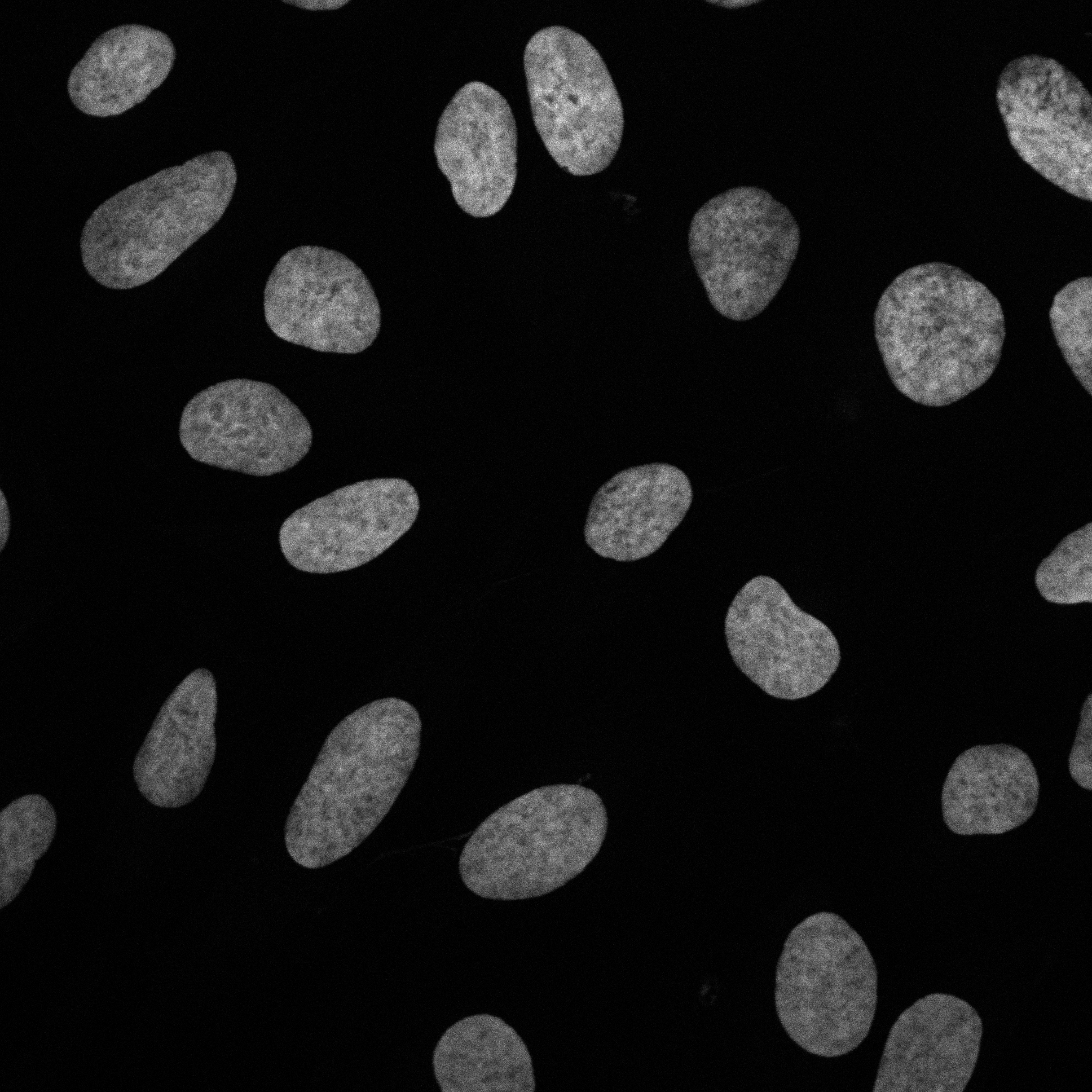

Supplement: Supplementary file 5 — Source data Fig. 5 [file 44318_2026_790_MOESM5_ESM.zip › Figure 5/Figure 5C_pCHK1_TelC_U2OS_siFANCM/C1-U2OS_KO_clone_1_siCTRL_DAPI.tif]

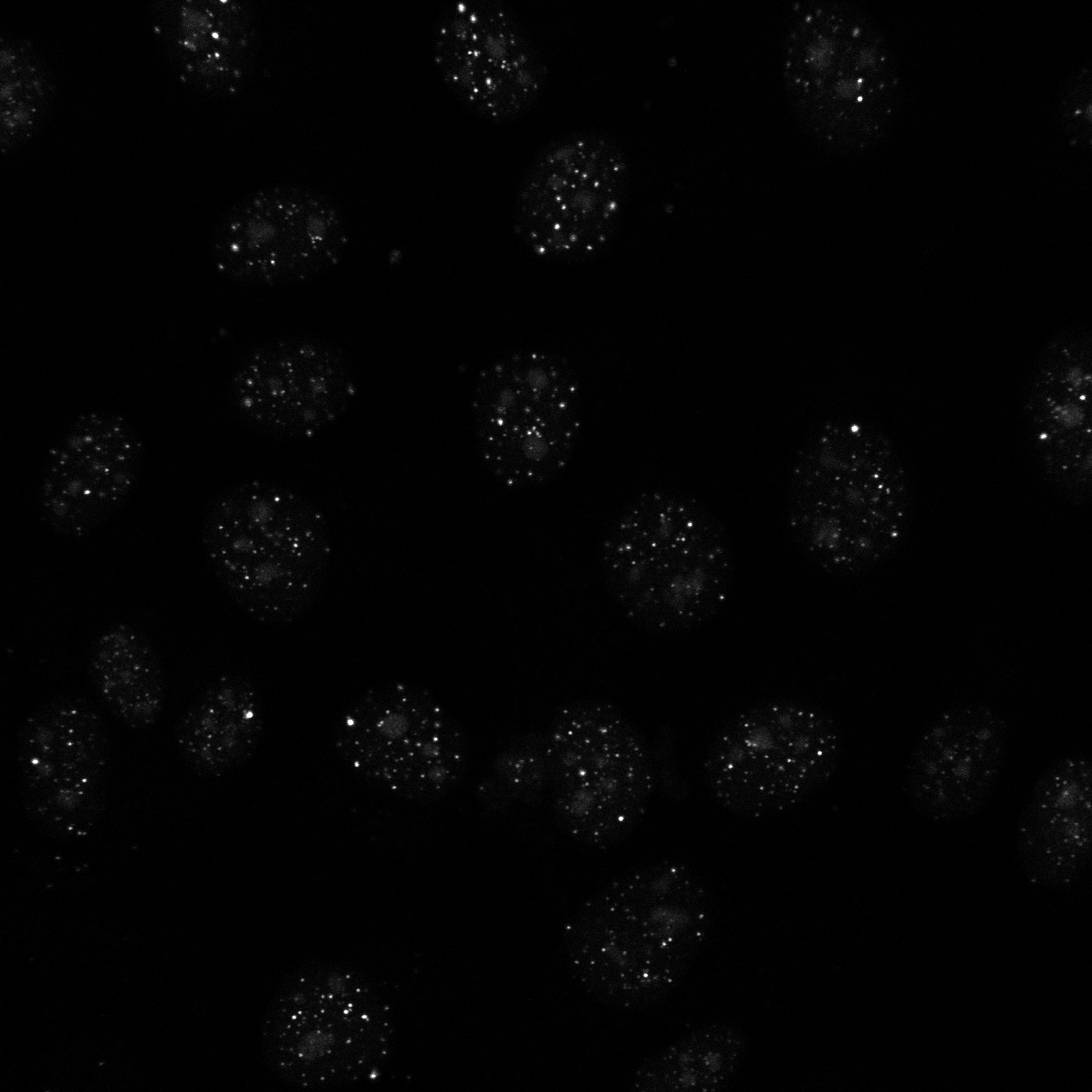

Supplement: Supplementary file 5 — Source data Fig. 5 [file 44318_2026_790_MOESM5_ESM.zip › Figure 5/Figure 5C_pCHK1_TelC_U2OS_siFANCM/C3-U2OS_KO_clone_2_siCTRL_TelC.tif]

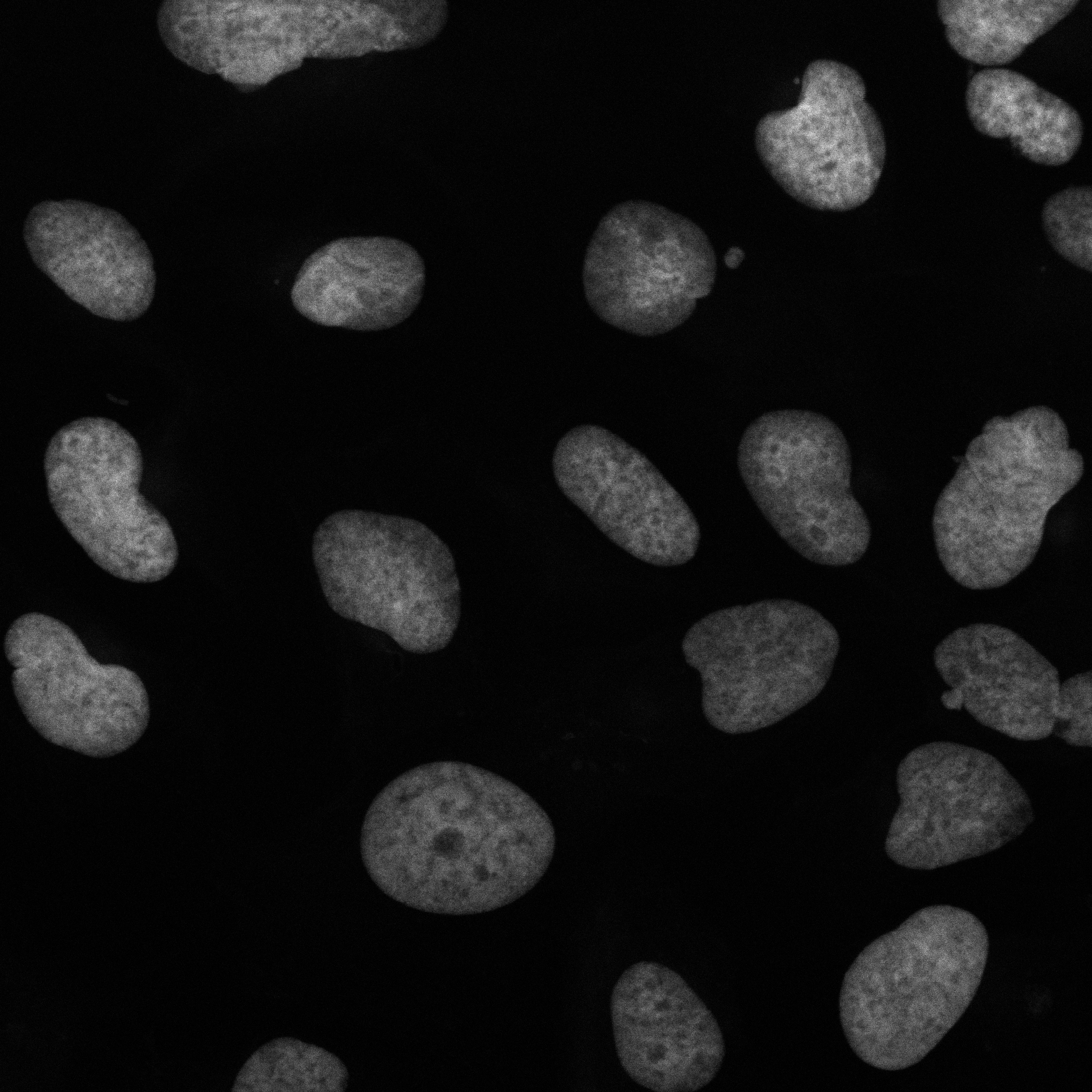

Supplement: Supplementary file 5 — Source data Fig. 5 [file 44318_2026_790_MOESM5_ESM.zip › Figure 5/Figure 5C_pCHK1_TelC_U2OS_siFANCM/C1-U2OS_WT_siFANCM_DAPI.tif]

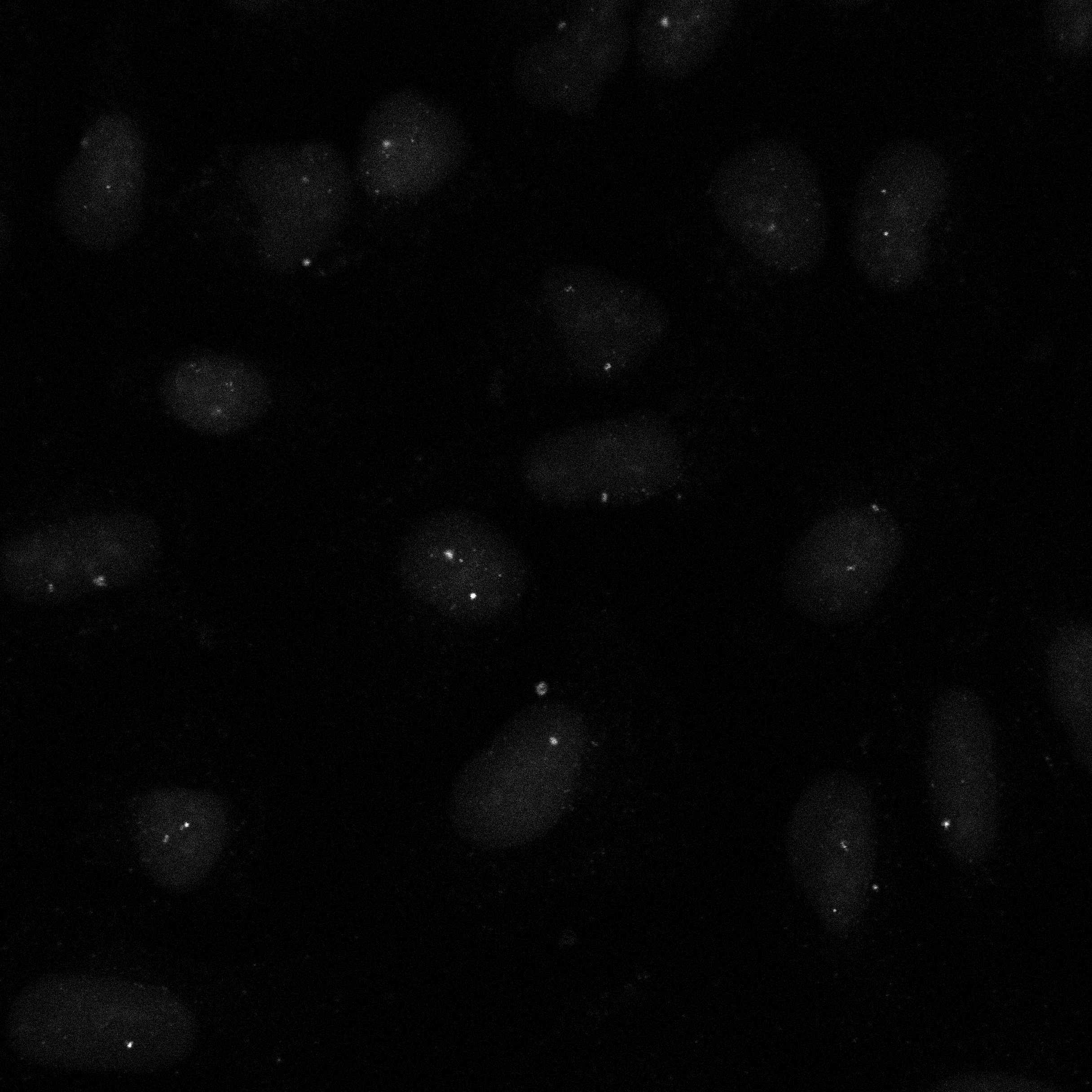

Supplement: Supplementary file 5 — Source data Fig. 5 [file 44318_2026_790_MOESM5_ESM.zip › Figure 5/Figure 5C_pCHK1_TelC_U2OS_siFANCM/C2-U2OS_WT_siCTRL_pS345-CHK1.tif]
