# Supplementary figures and images for: SLX4IP limits replication stress globally and at ALT telomeres (part 2 of 2)
Source: EMBO J. 2026 May 7;45(12):4176–219. doi: 10.1038/s44318-026-00790-4 (PMC13269807; doi:10.1038/s44318-026-00790-4)

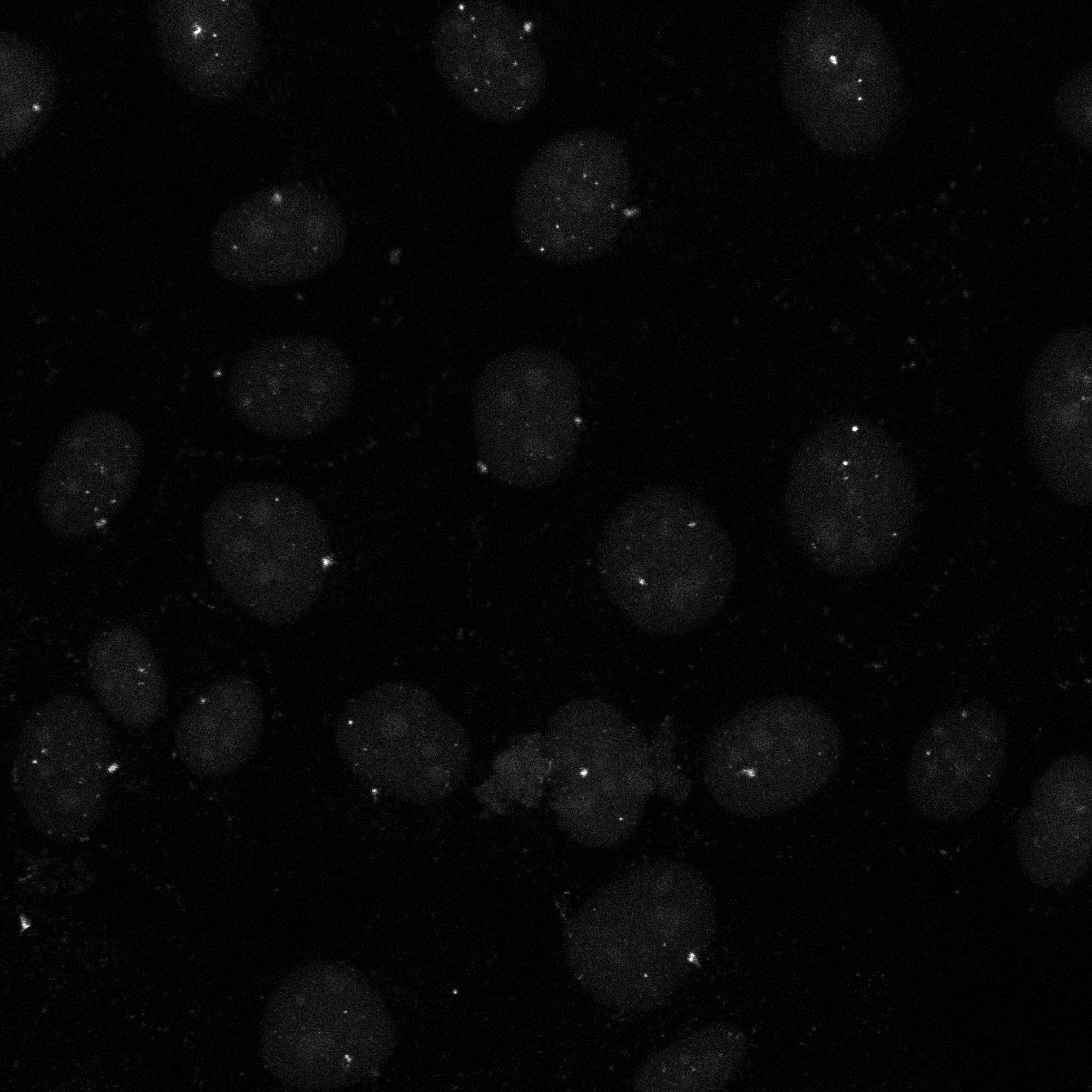

Supplement: Supplementary file 5 — Source data Fig. 5 [file 44318_2026_790_MOESM5_ESM.zip › Figure 5/Figure 5C_pCHK1_TelC_U2OS_siFANCM/C2-U2OS_KO_clone_2_siCTRL_pS345-CHK1.tif]

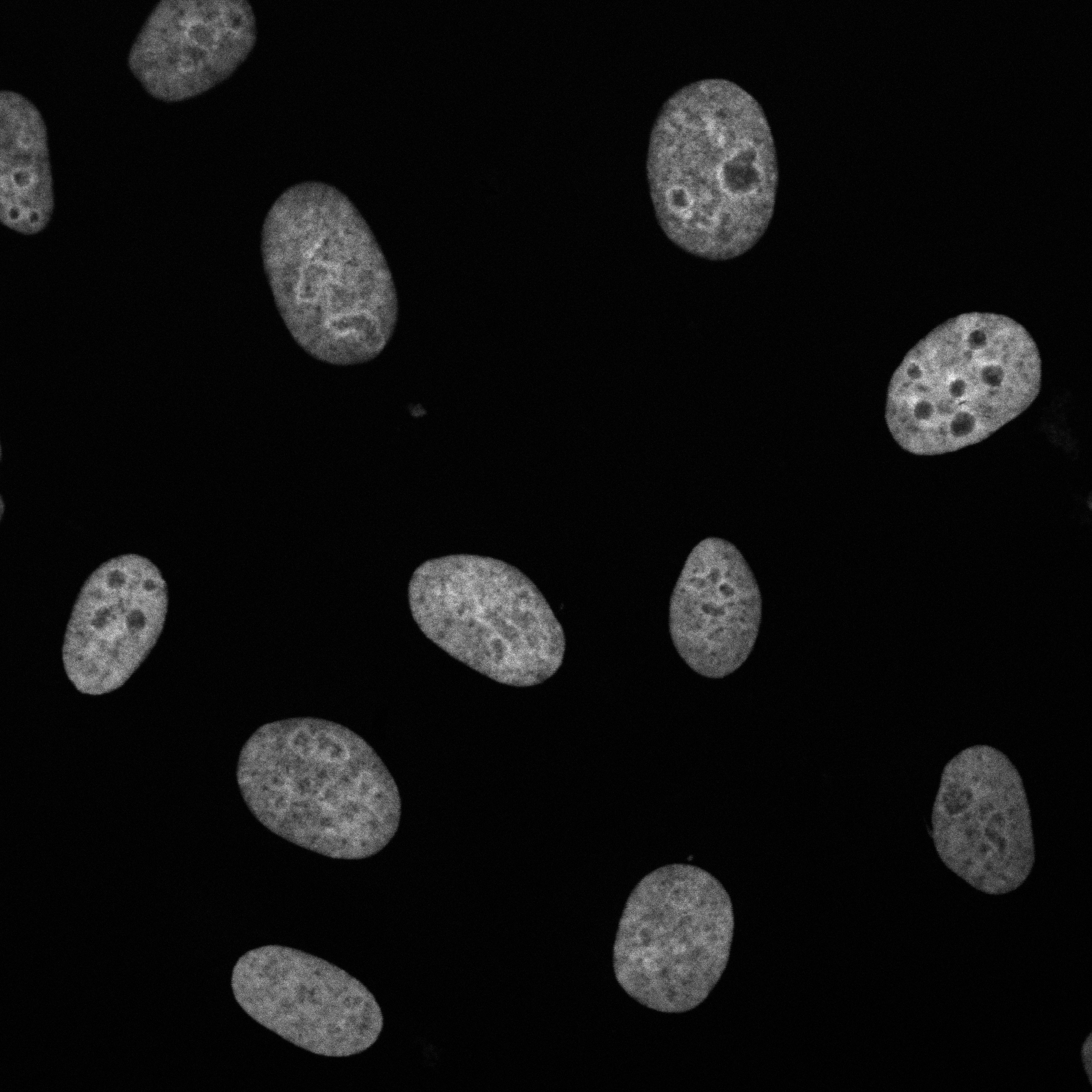

Supplement: Supplementary file 5 — Source data Fig. 5 [file 44318_2026_790_MOESM5_ESM.zip › Figure 5/Figure 5C_pCHK1_TelC_U2OS_siFANCM/C1-U2OS_KO_clone_2_siFANCM_DAPI.tif]

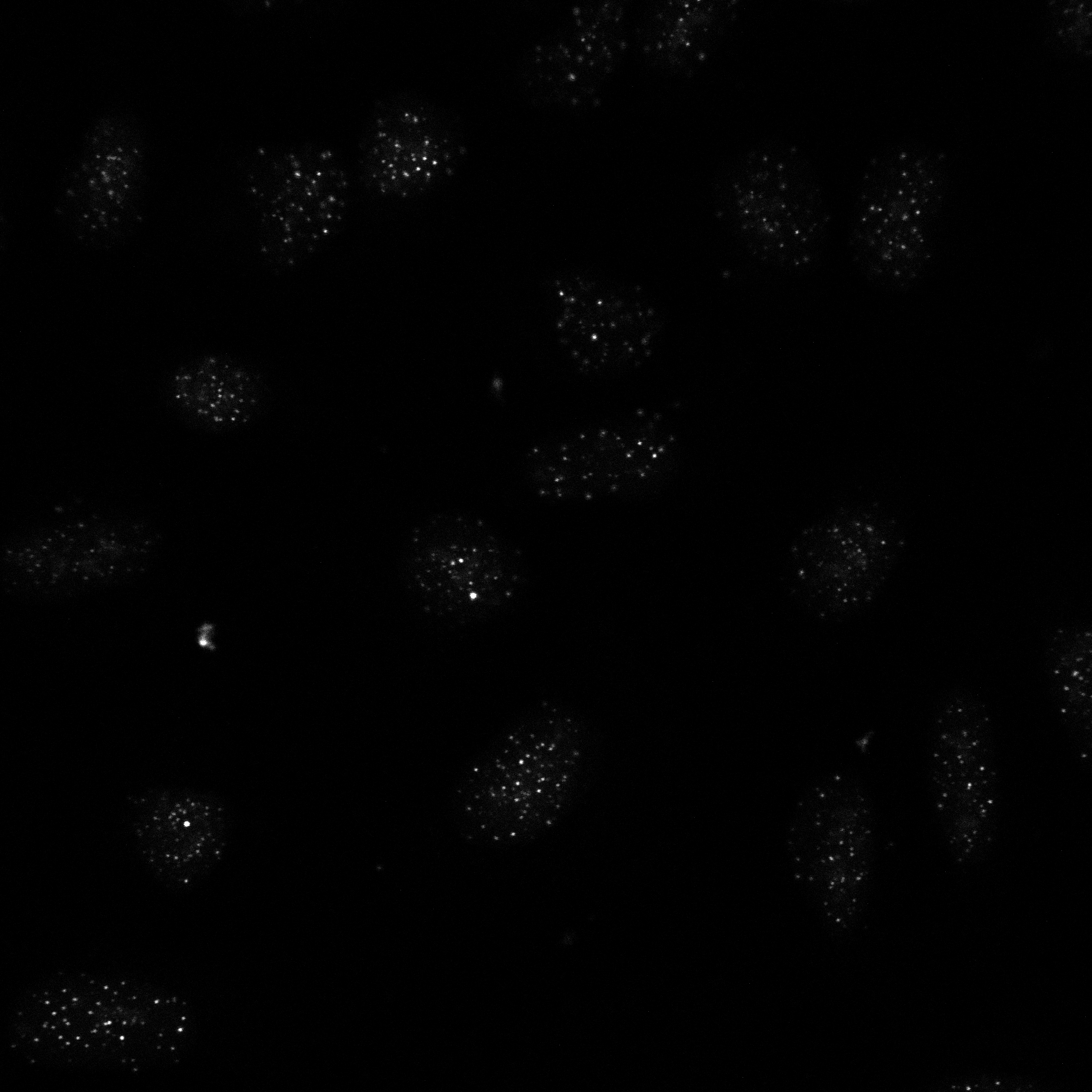

Supplement: Supplementary file 5 — Source data Fig. 5 [file 44318_2026_790_MOESM5_ESM.zip › Figure 5/Figure 5C_pCHK1_TelC_U2OS_siFANCM/C3-U2OS_WT_siCTRL_TelC.tif]

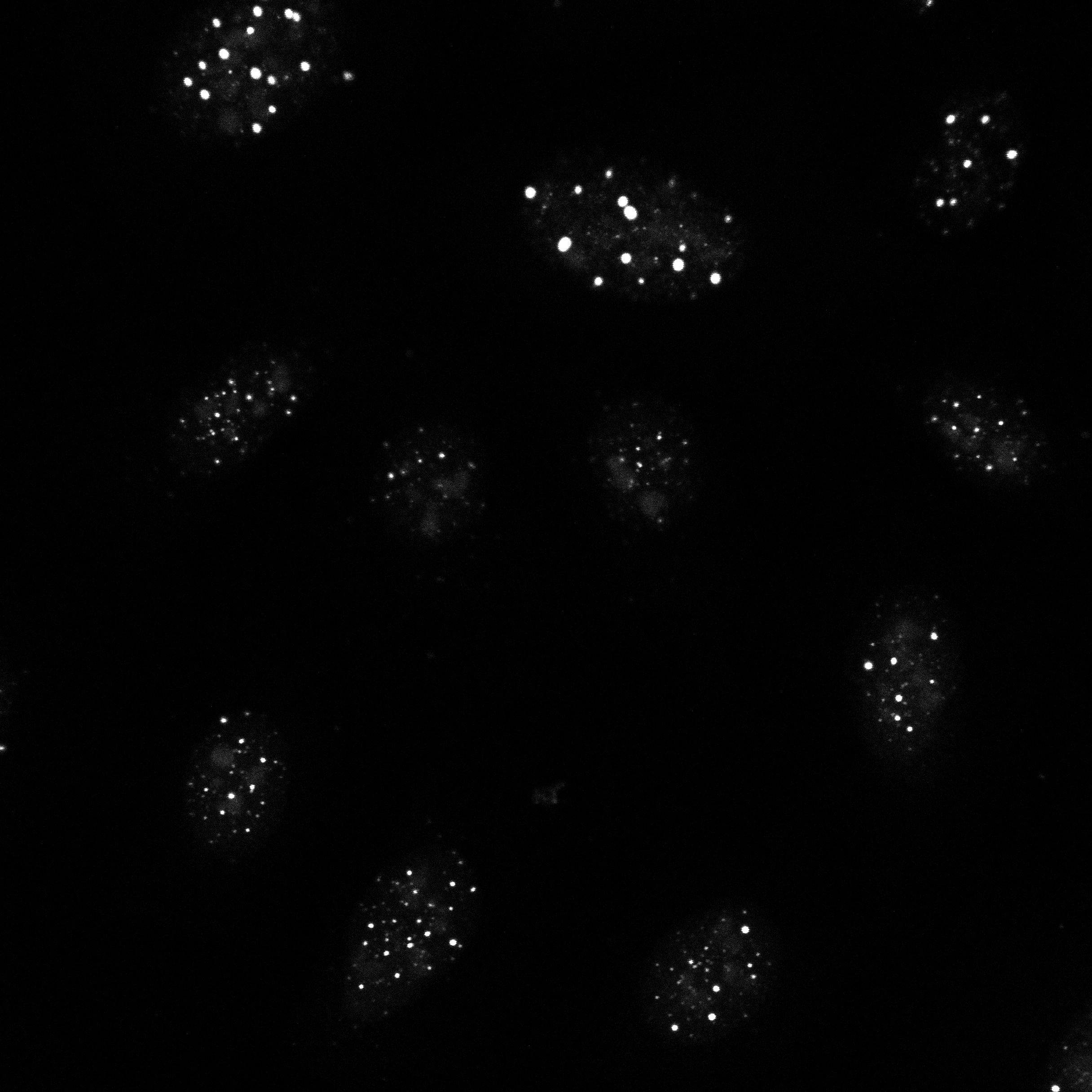

Supplement: Supplementary file 5 — Source data Fig. 5 [file 44318_2026_790_MOESM5_ESM.zip › Figure 5/Figure 5C_pCHK1_TelC_U2OS_siFANCM/C3-U2OS_KO_clone_1_siFANCM_TelC.tif]

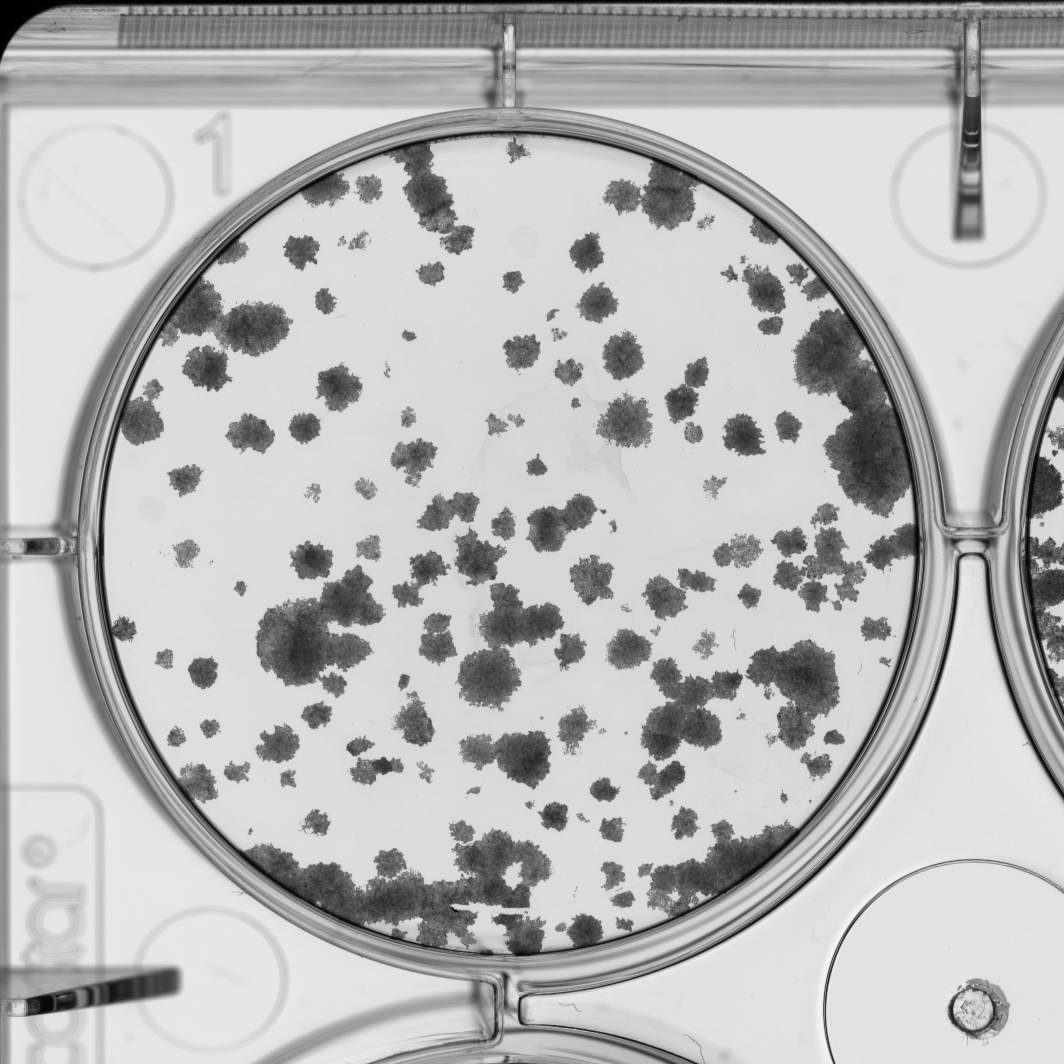

Supplement: Supplementary file 6 — Source data Fig. 6 [file 44318_2026_790_MOESM6_ESM.zip › Figure 6/Figure 6E_clonogenics_U2OS_BLM_rescue/U2OS_WT_siCTRL.tif]

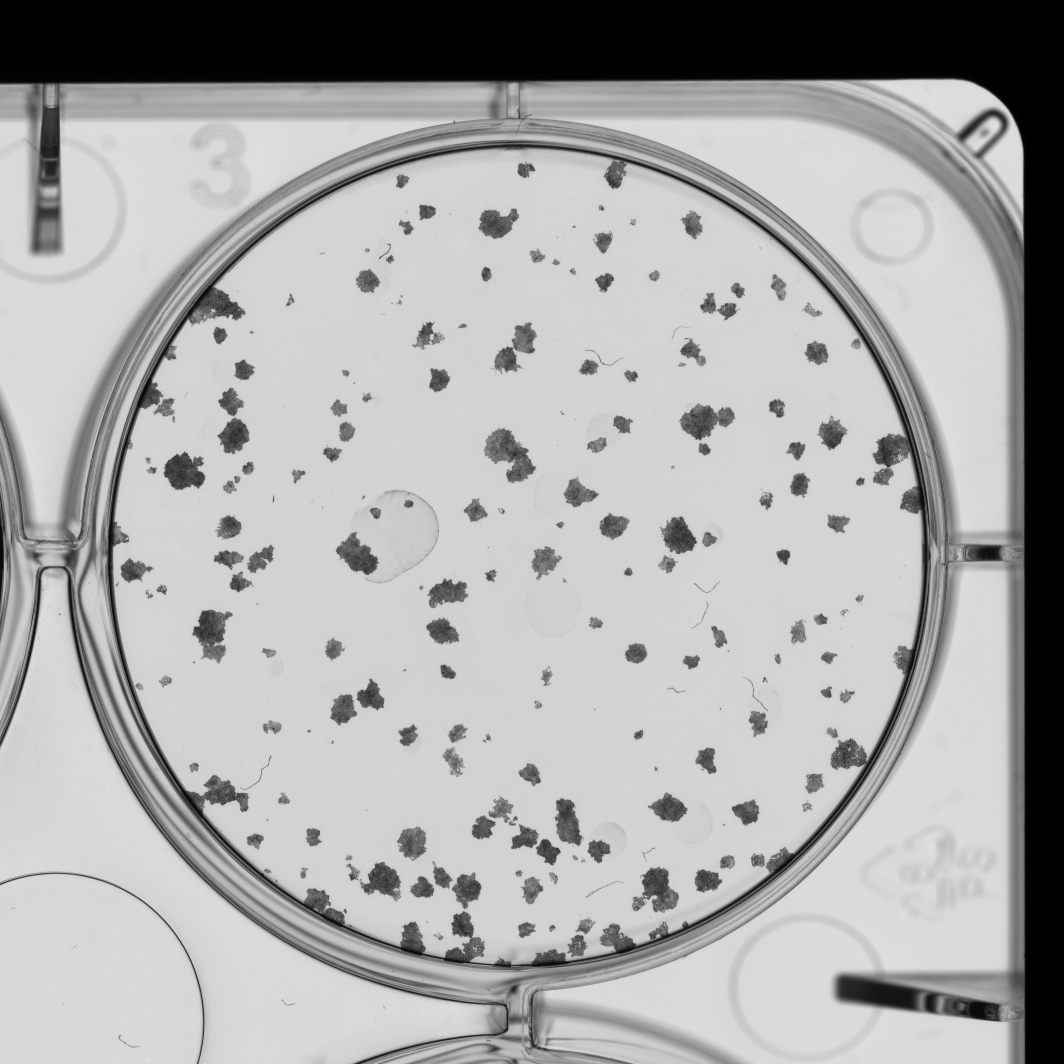

Supplement: Supplementary file 6 — Source data Fig. 6 [file 44318_2026_790_MOESM6_ESM.zip › Figure 6/Figure 6E_clonogenics_U2OS_BLM_rescue/U2OS_SLX4IP_KO_clone_1_siFANCM_siBLM.tif]

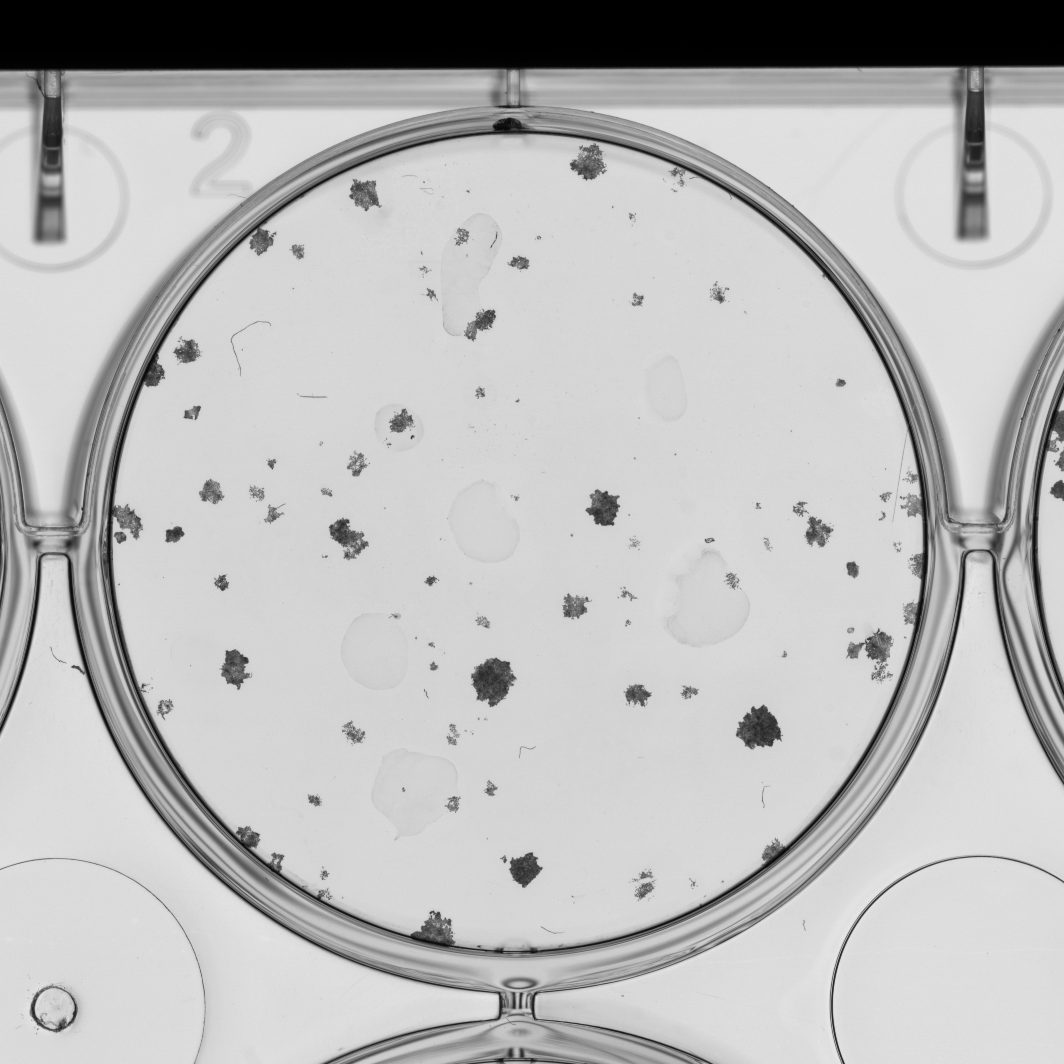

Supplement: Supplementary file 6 — Source data Fig. 6 [file 44318_2026_790_MOESM6_ESM.zip › Figure 6/Figure 6E_clonogenics_U2OS_BLM_rescue/U2OS_SLX4IP_KO_clone_2_siFANCM.tif]

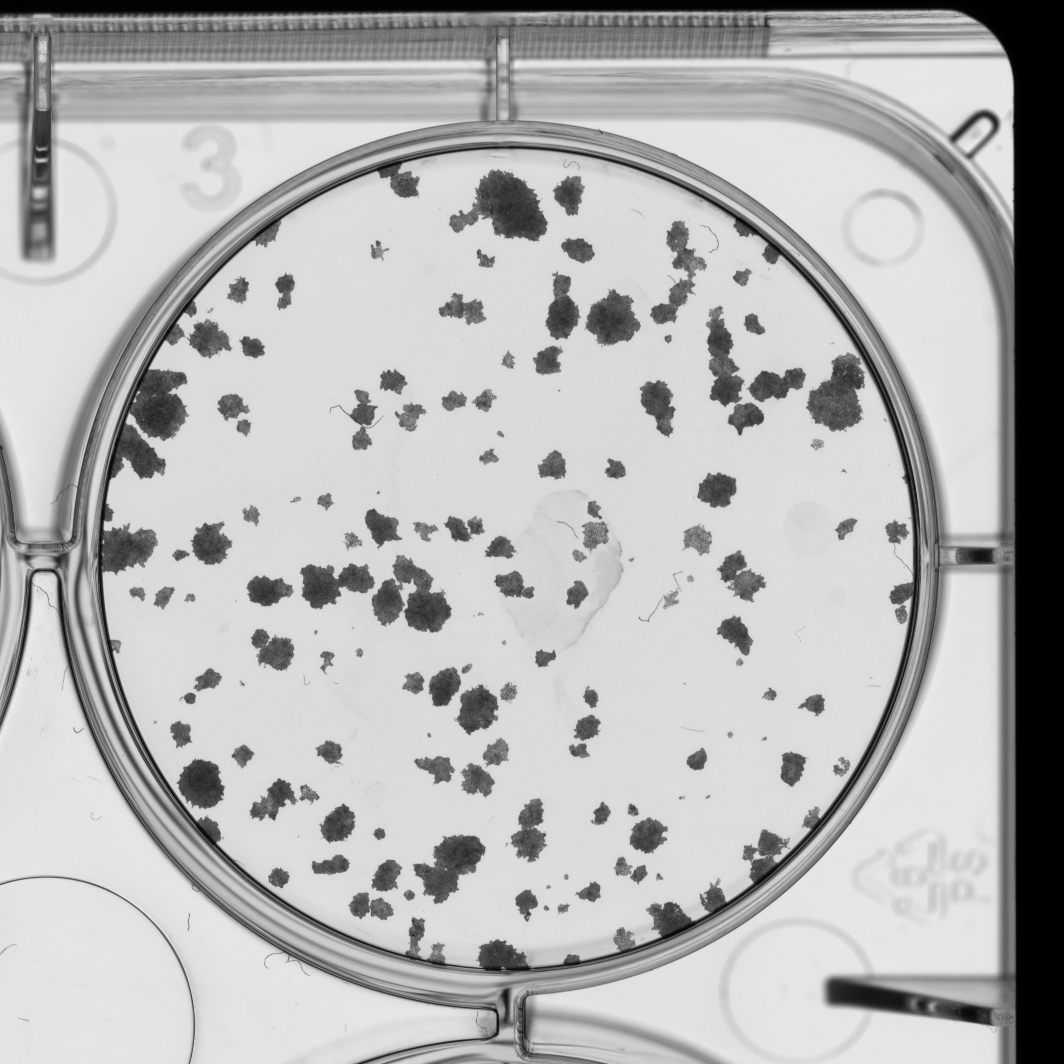

Supplement: Supplementary file 6 — Source data Fig. 6 [file 44318_2026_790_MOESM6_ESM.zip › Figure 6/Figure 6E_clonogenics_U2OS_BLM_rescue/U2OS_SLX4IP_KO_clone_1_siBLM.tif]

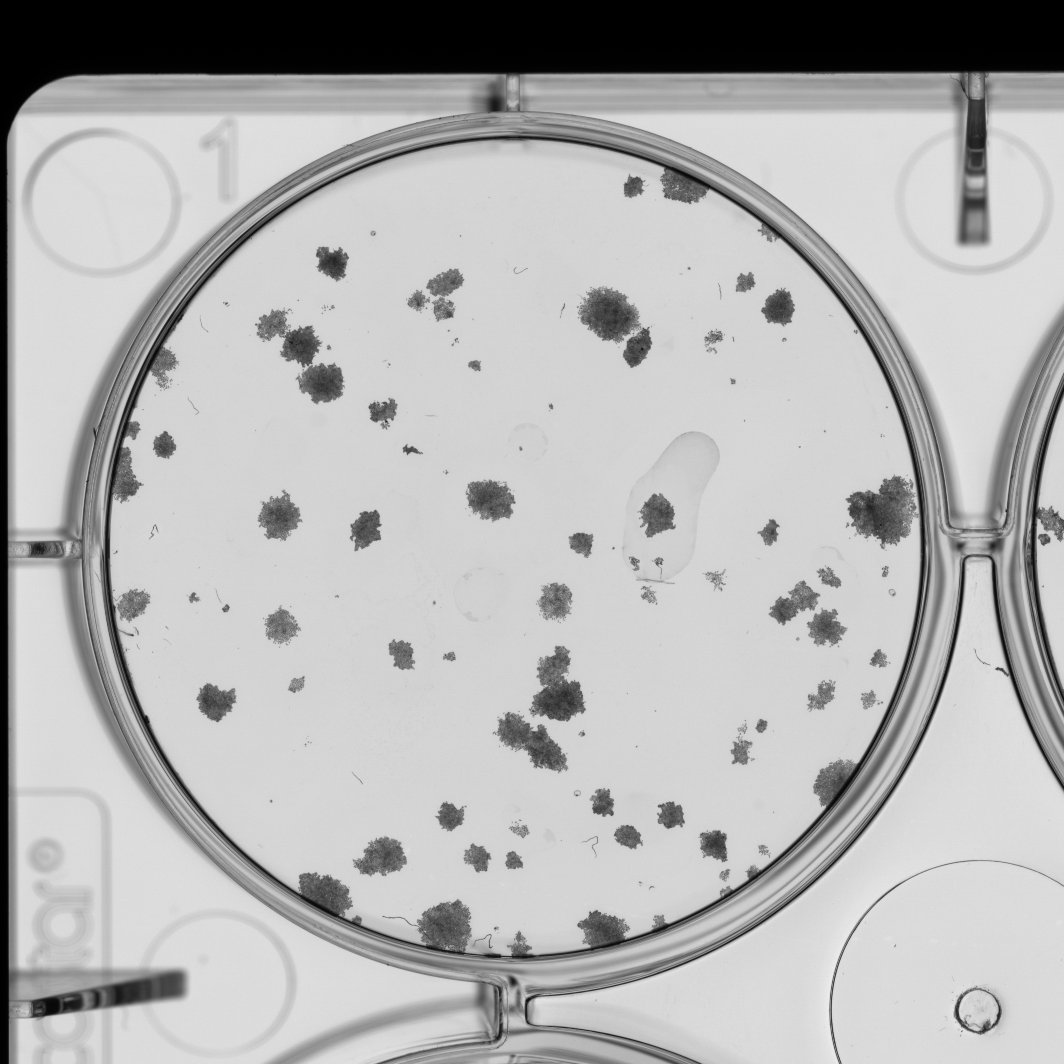

Supplement: Supplementary file 6 — Source data Fig. 6 [file 44318_2026_790_MOESM6_ESM.zip › Figure 6/Figure 6E_clonogenics_U2OS_BLM_rescue/U2OS_WT_siFANCM.tif]

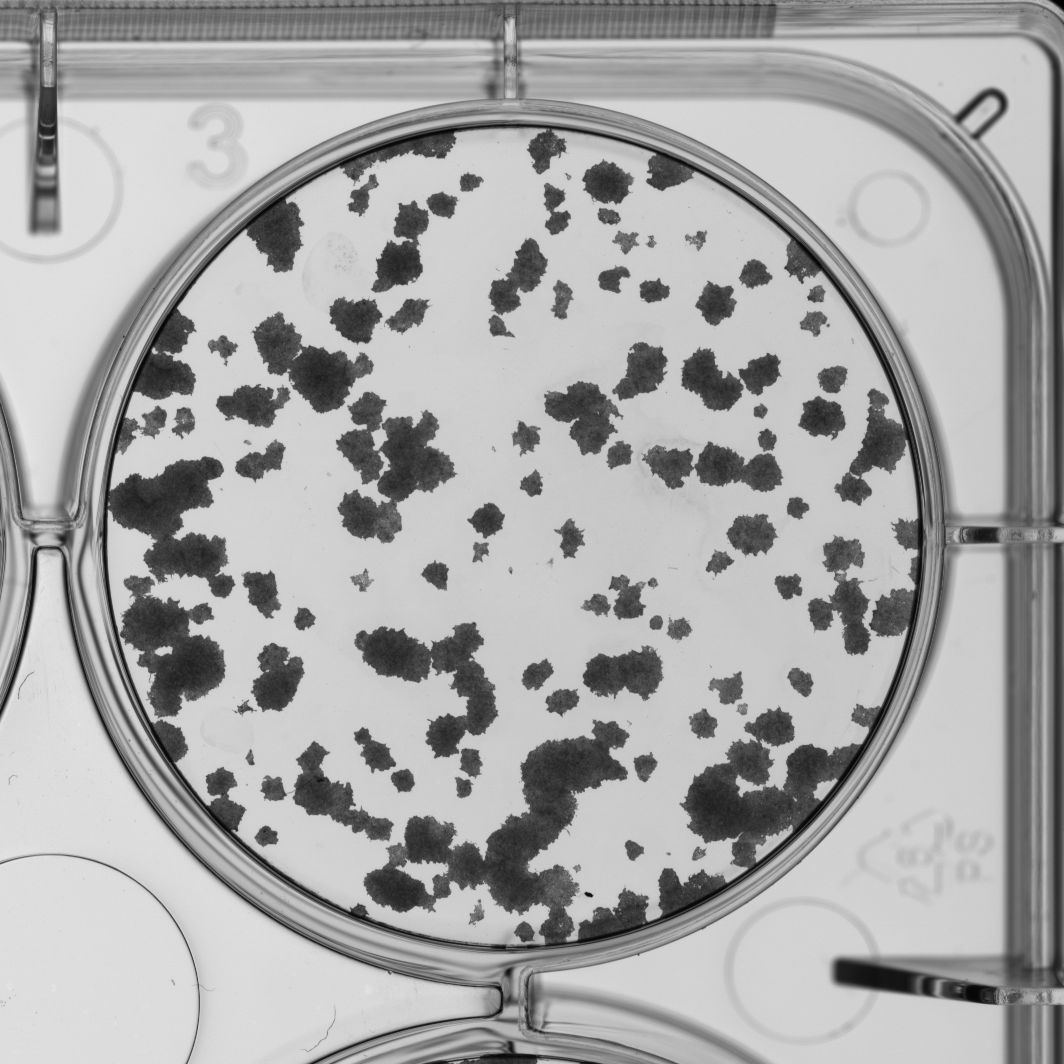

Supplement: Supplementary file 6 — Source data Fig. 6 [file 44318_2026_790_MOESM6_ESM.zip › Figure 6/Figure 6E_clonogenics_U2OS_BLM_rescue/U2OS_SLX4IP_KO_clone_1_siCTRL.tif]

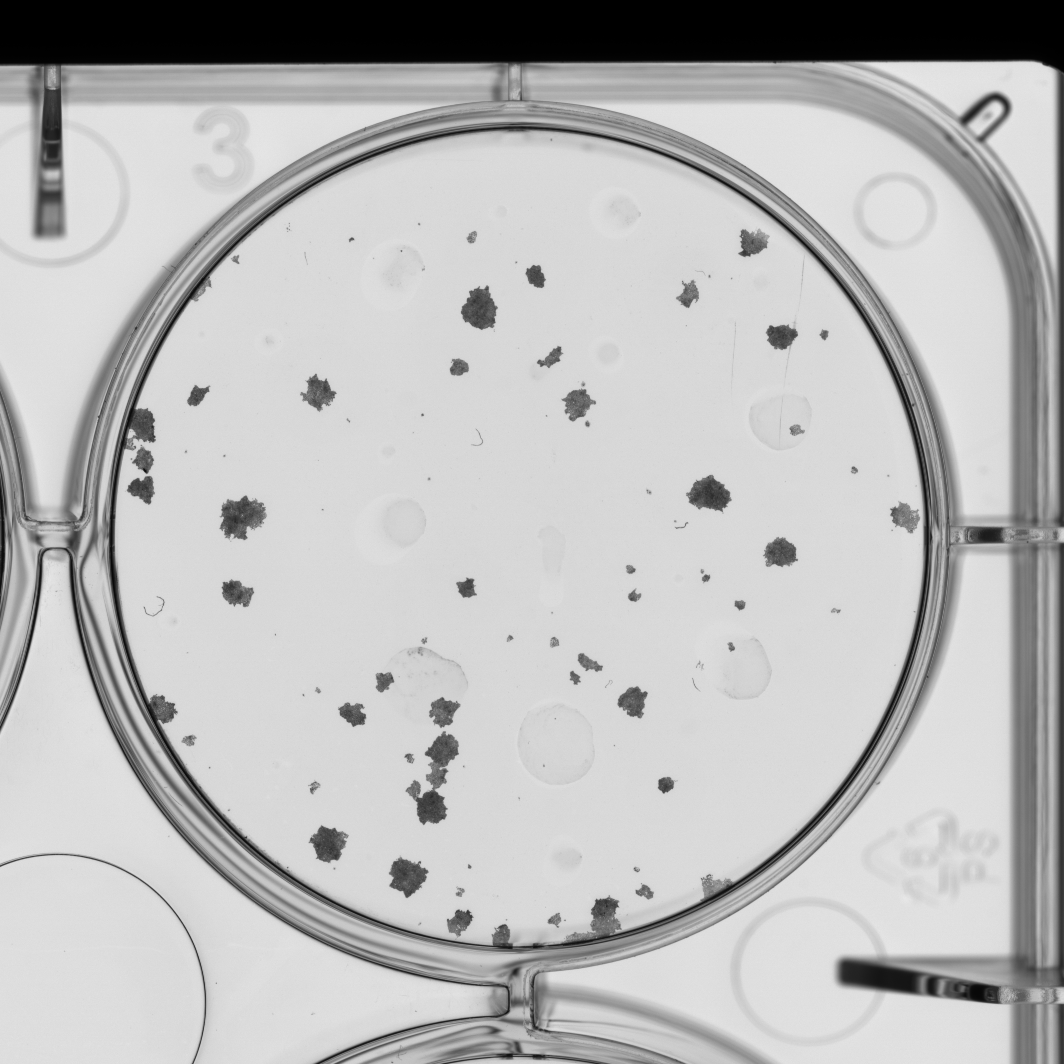

Supplement: Supplementary file 6 — Source data Fig. 6 [file 44318_2026_790_MOESM6_ESM.zip › Figure 6/Figure 6E_clonogenics_U2OS_BLM_rescue/U2OS_SLX4IP_KO_clone_1_siFANCM.tif]

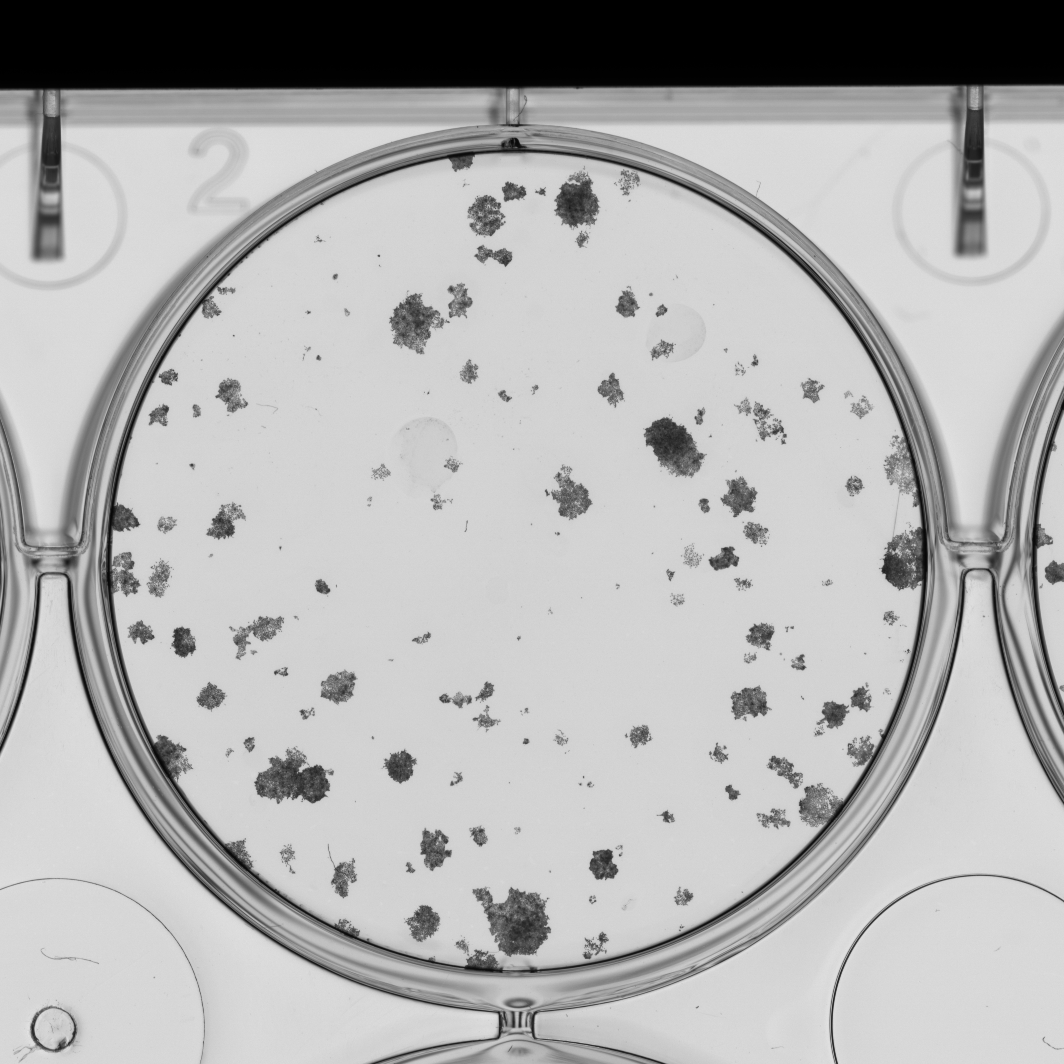

Supplement: Supplementary file 6 — Source data Fig. 6 [file 44318_2026_790_MOESM6_ESM.zip › Figure 6/Figure 6E_clonogenics_U2OS_BLM_rescue/U2OS_SLX4IP_KO_clone_2_siFANCM_siBLM.tif]

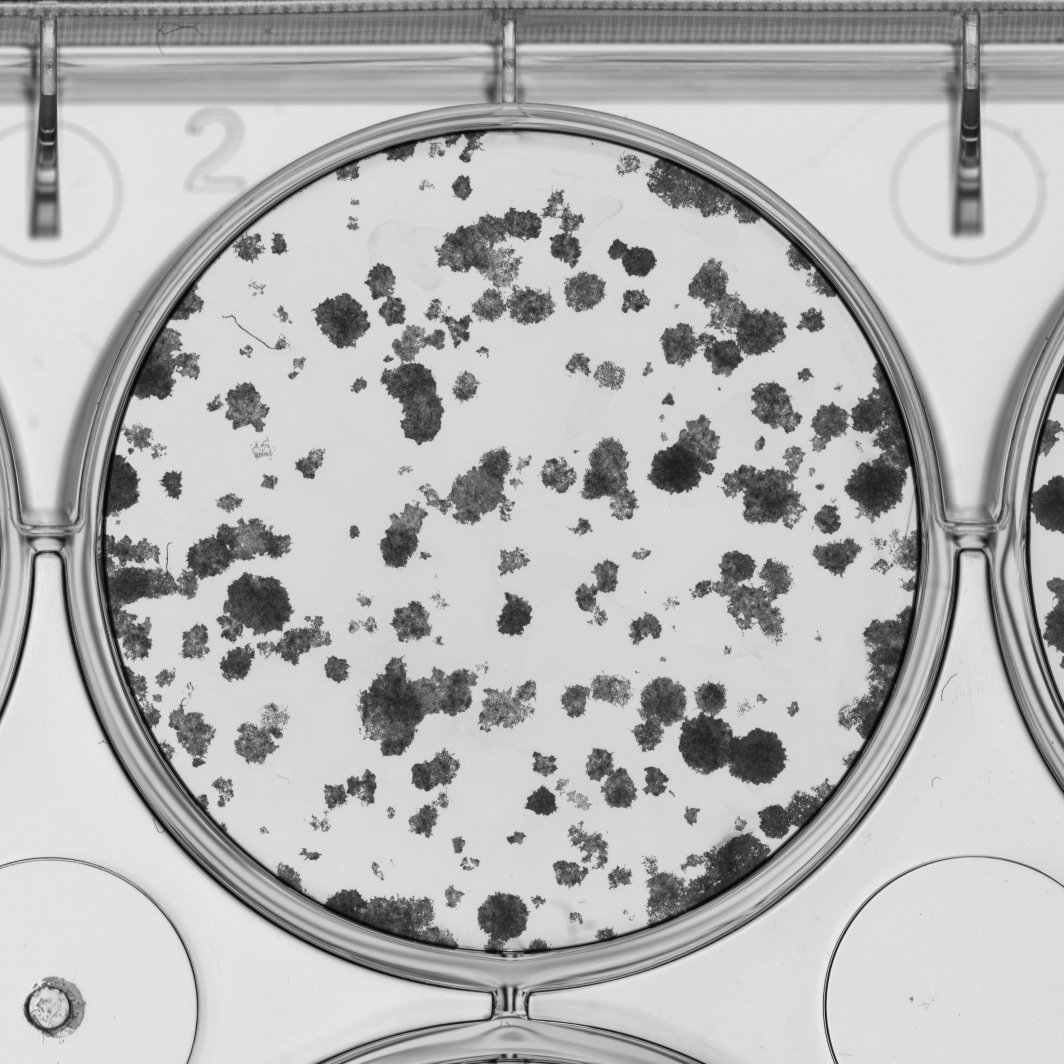

Supplement: Supplementary file 6 — Source data Fig. 6 [file 44318_2026_790_MOESM6_ESM.zip › Figure 6/Figure 6E_clonogenics_U2OS_BLM_rescue/U2OS_SLX4IP_KO_clone_2_siCTRL.tif]

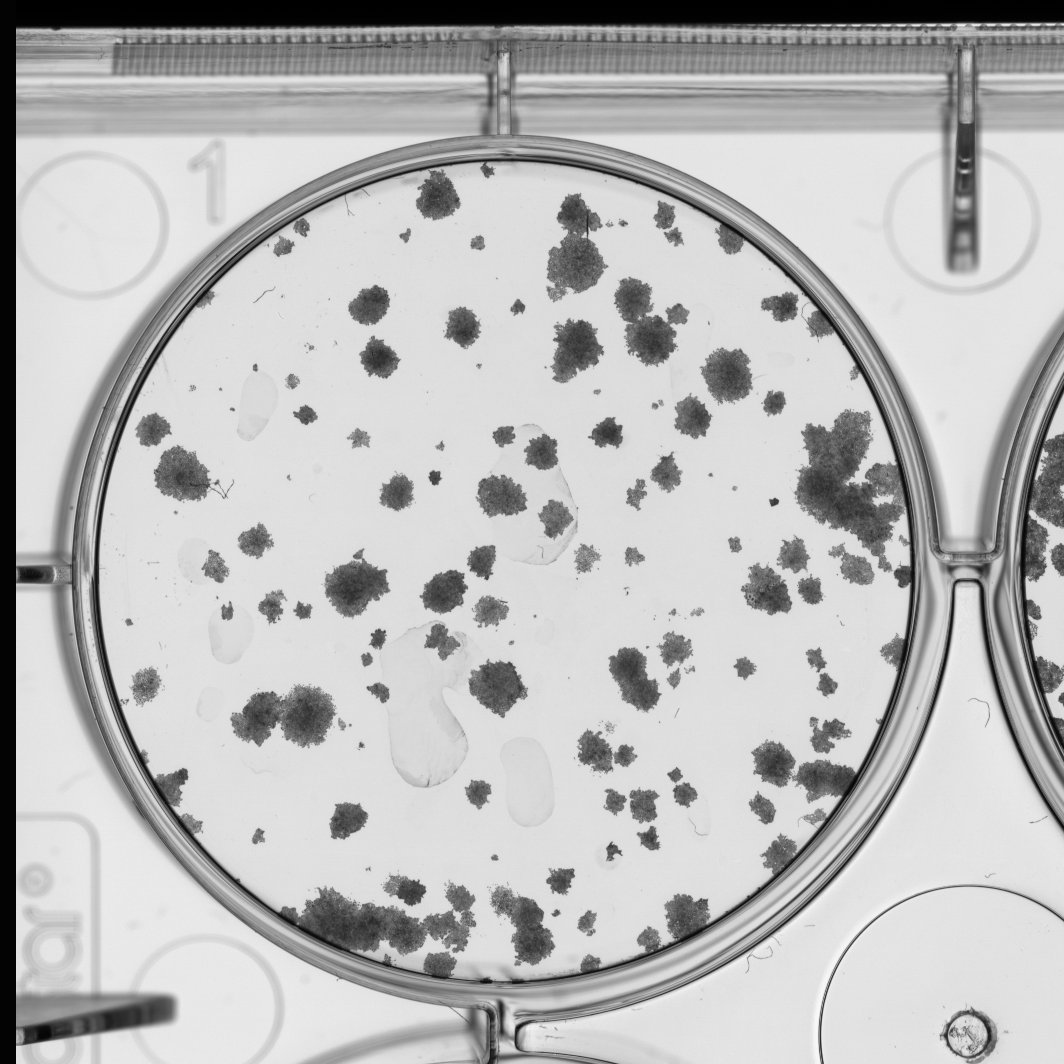

Supplement: Supplementary file 6 — Source data Fig. 6 [file 44318_2026_790_MOESM6_ESM.zip › Figure 6/Figure 6E_clonogenics_U2OS_BLM_rescue/U2OS_WT_siBLM.tif]

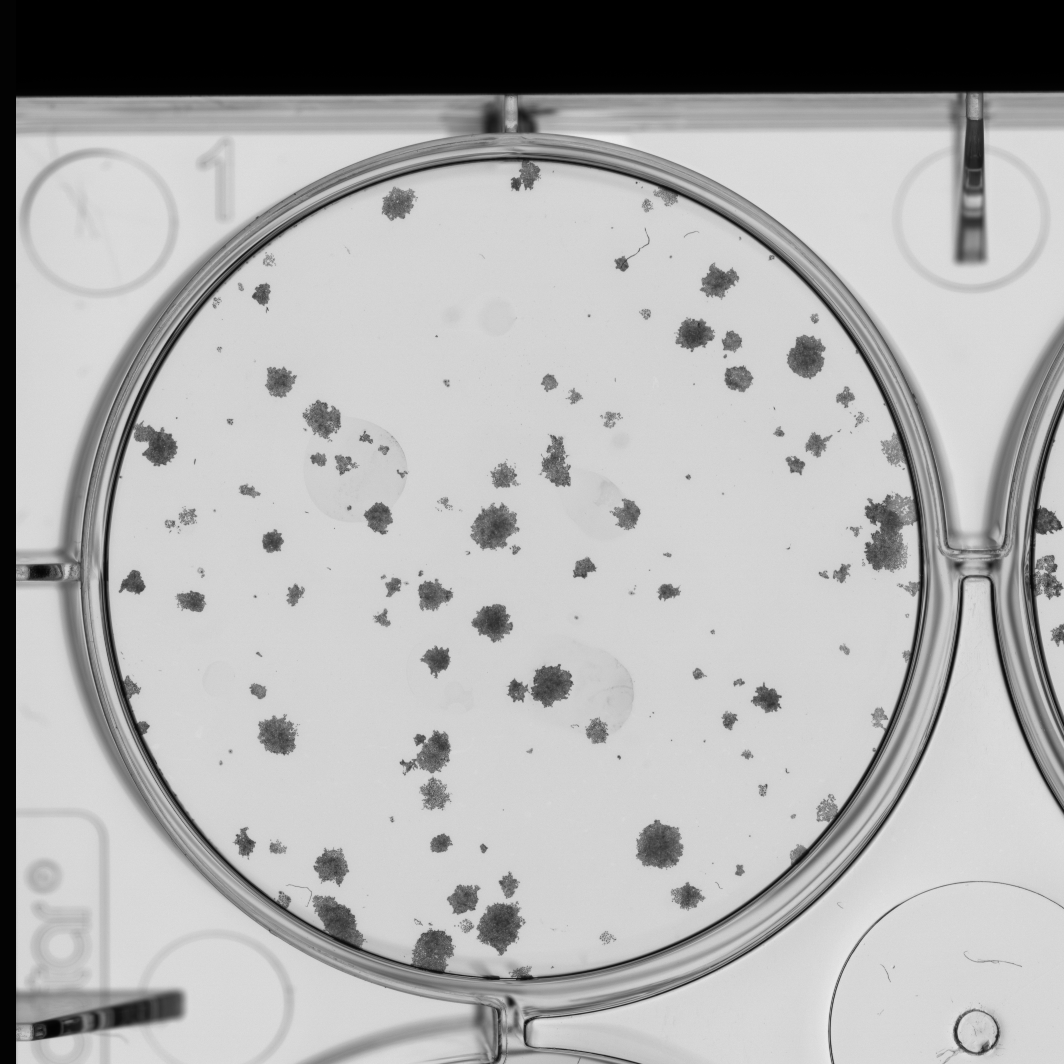

Supplement: Supplementary file 6 — Source data Fig. 6 [file 44318_2026_790_MOESM6_ESM.zip › Figure 6/Figure 6E_clonogenics_U2OS_BLM_rescue/U2OS_WT_siFANCM_siBLM.tif]

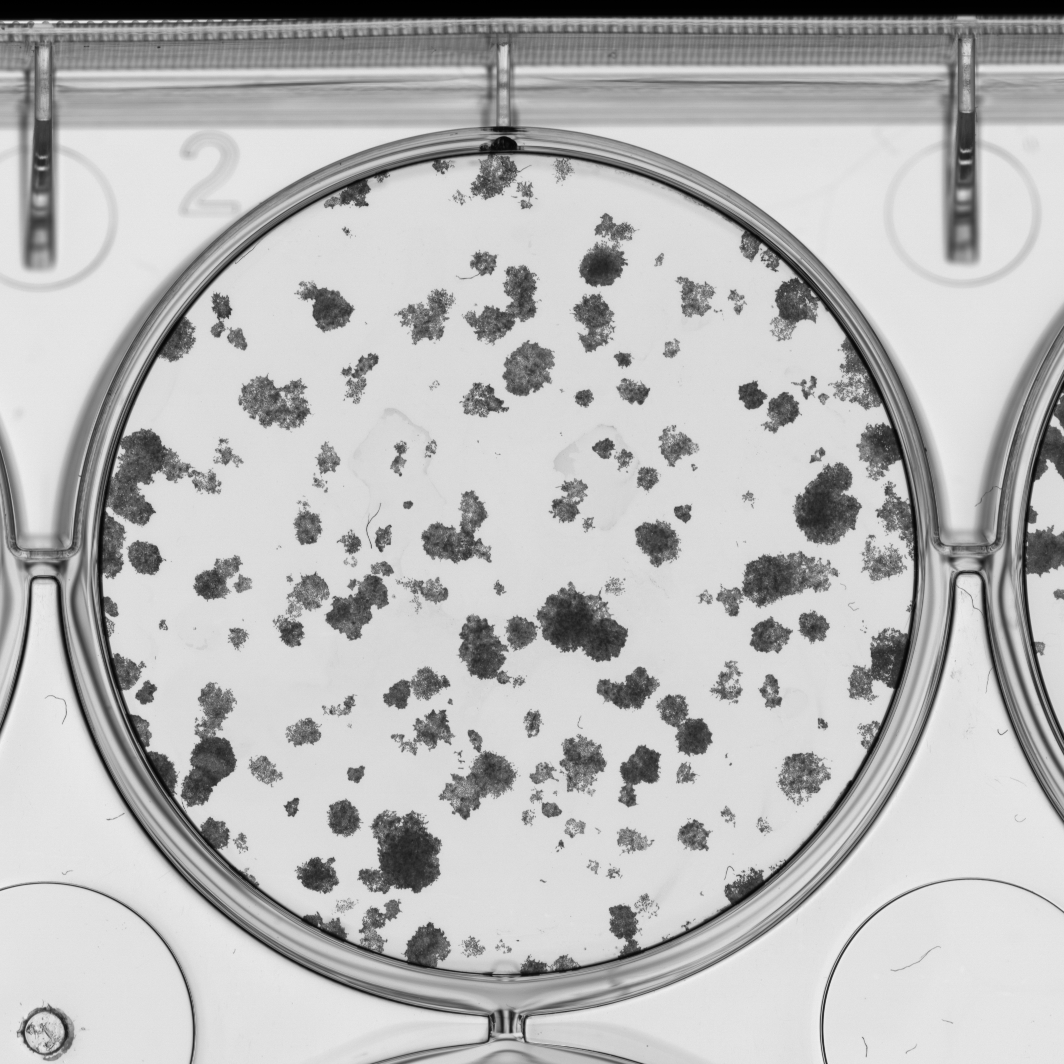

Supplement: Supplementary file 6 — Source data Fig. 6 [file 44318_2026_790_MOESM6_ESM.zip › Figure 6/Figure 6E_clonogenics_U2OS_BLM_rescue/U2OS_SLX4IP_KO_clone_2_siBLM.tif]

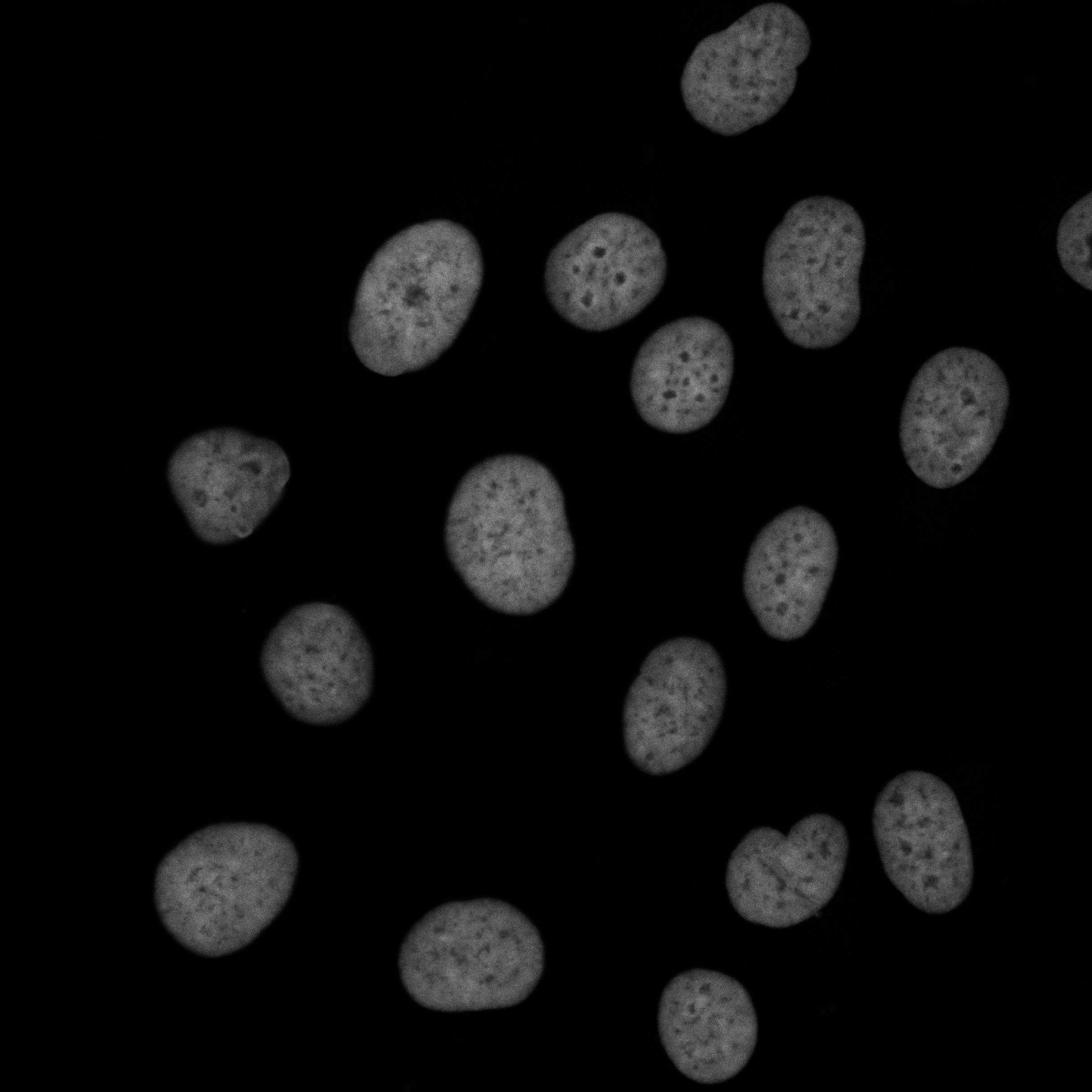

Supplement: Supplementary file 6 — Source data Fig. 6 [file 44318_2026_790_MOESM6_ESM.zip › Figure 6/Figure 6C_pRPA_TelC_U2OS_BLM_rescue/C1-U2OS_SLX4IP_KO_clone_2_siCTRL_DAPI.tif]

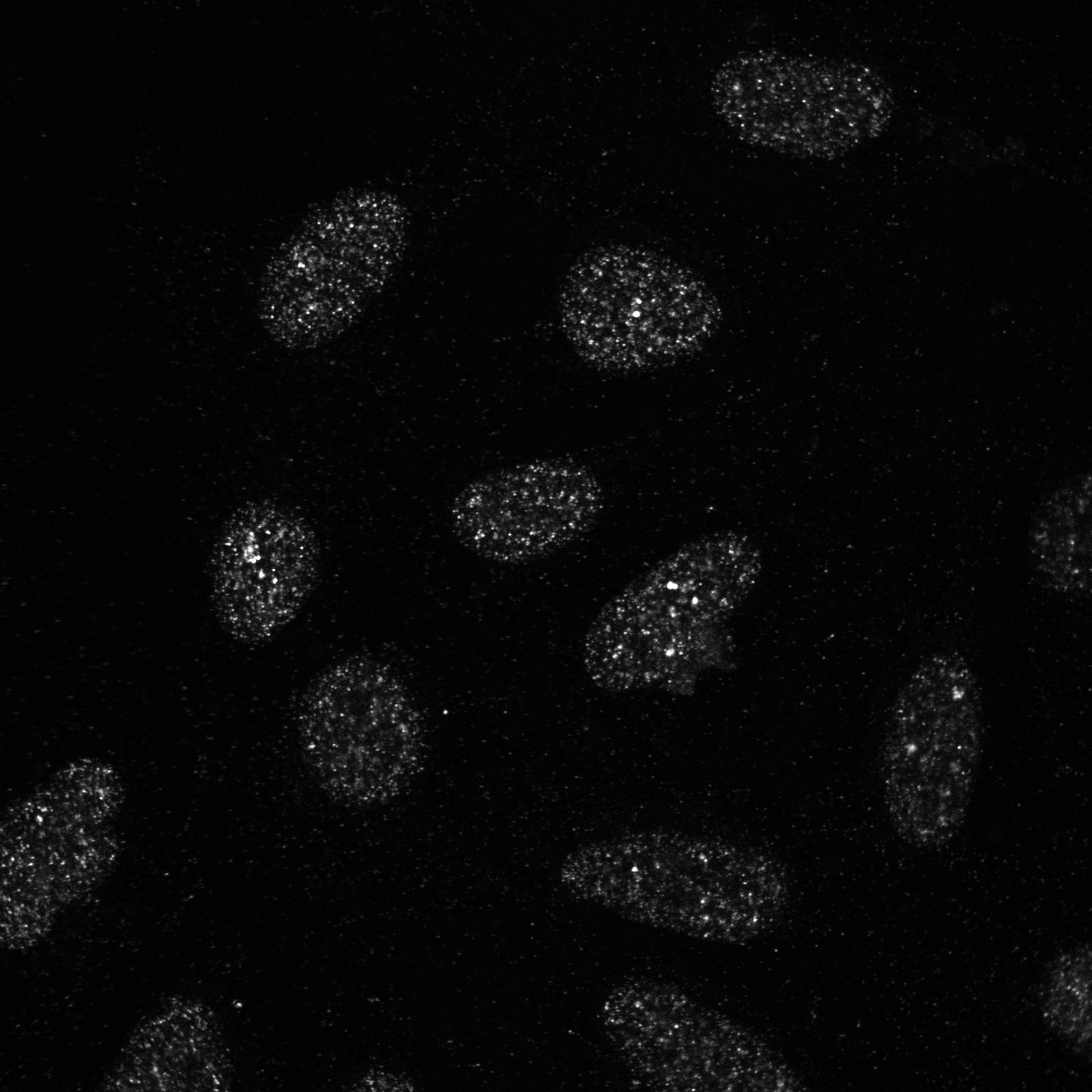

Supplement: Supplementary file 6 — Source data Fig. 6 [file 44318_2026_790_MOESM6_ESM.zip › Figure 6/Figure 6C_pRPA_TelC_U2OS_BLM_rescue/C3-U2OS_WT_siCTRL_pS33-RPA.tif]

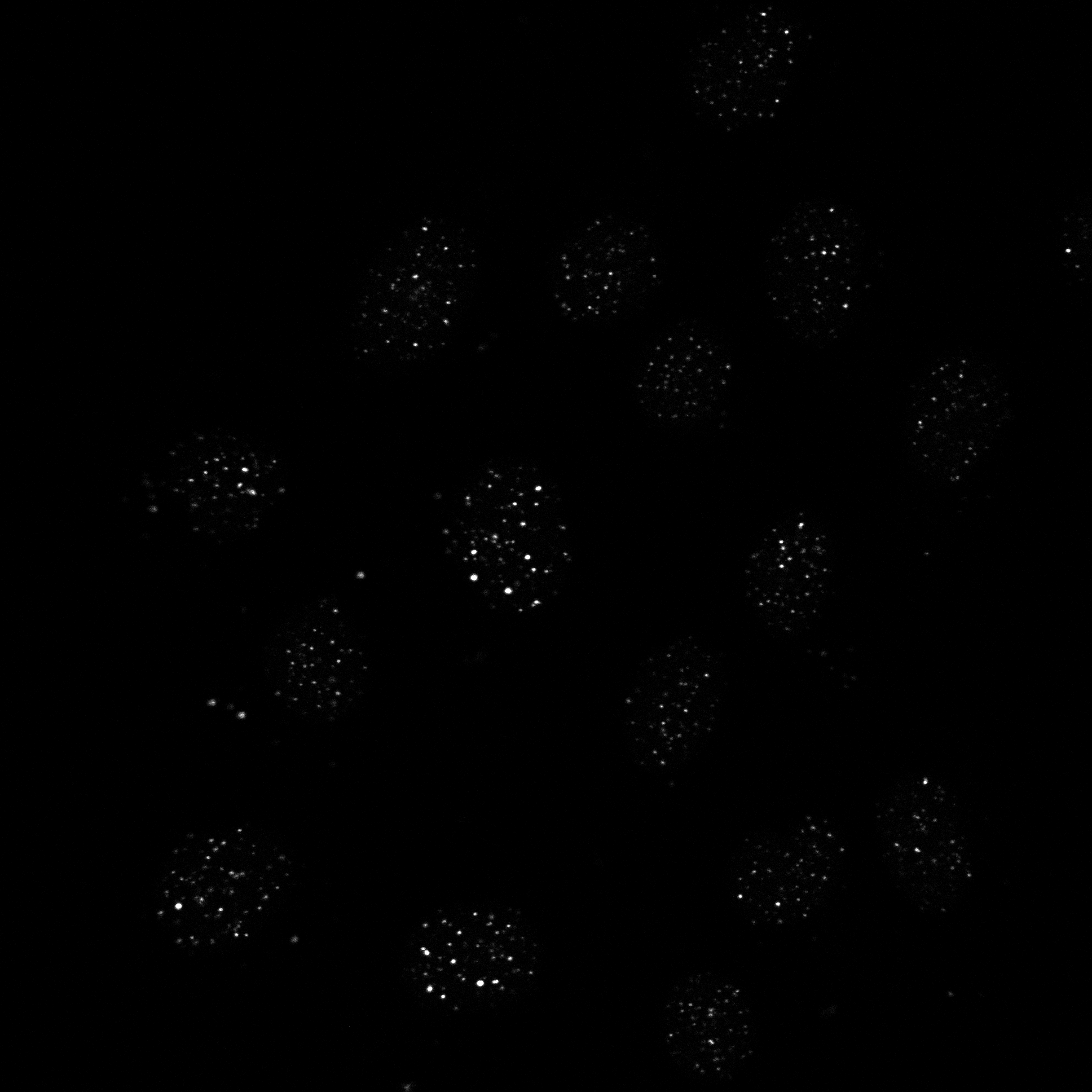

Supplement: Supplementary file 6 — Source data Fig. 6 [file 44318_2026_790_MOESM6_ESM.zip › Figure 6/Figure 6C_pRPA_TelC_U2OS_BLM_rescue/C4-U2OS_SLX4IP_KO_clone_2_siCTRL_TelC.tif]

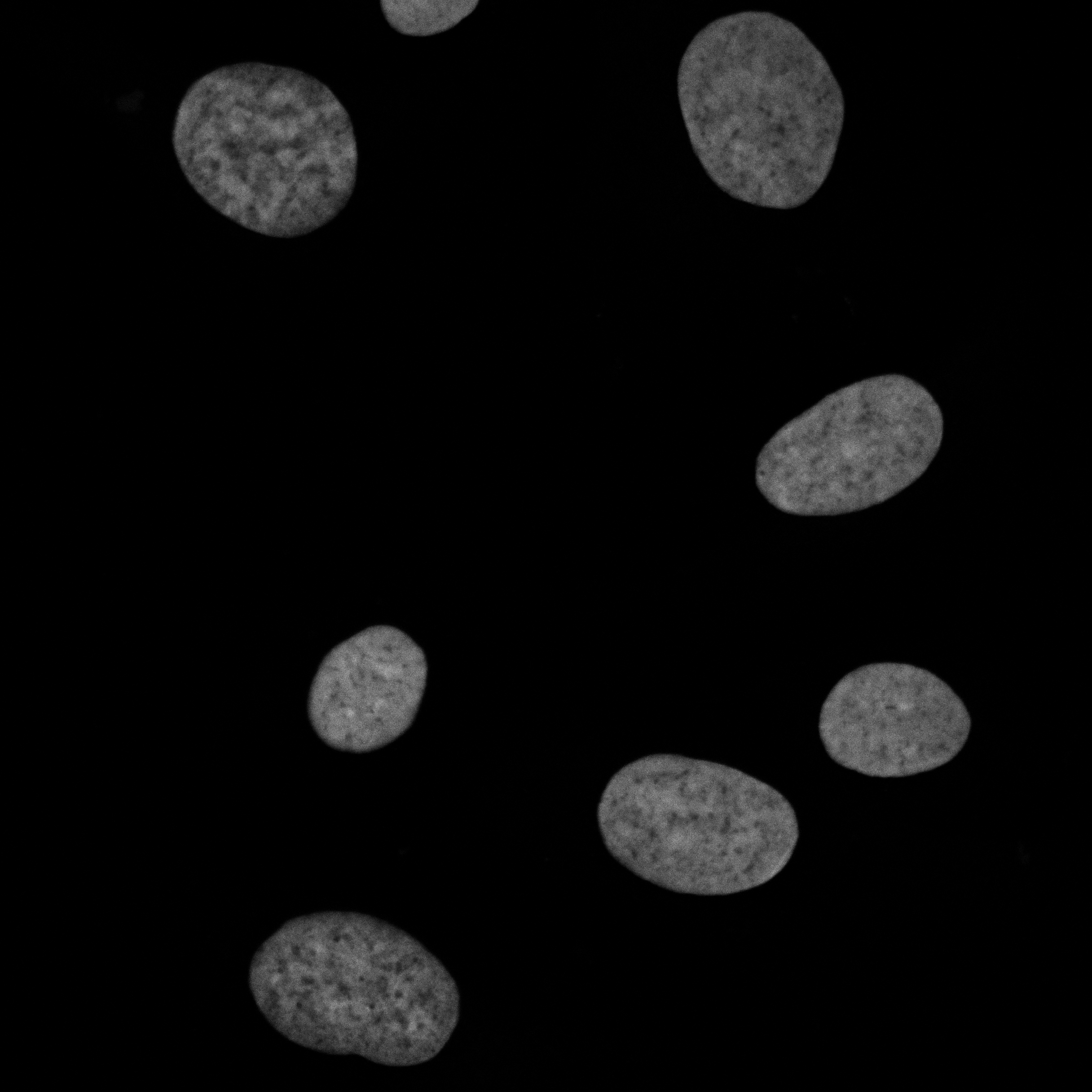

Supplement: Supplementary file 6 — Source data Fig. 6 [file 44318_2026_790_MOESM6_ESM.zip › Figure 6/Figure 6C_pRPA_TelC_U2OS_BLM_rescue/C1-U2OS_SLX4IP_KO_clone_1_siFANCM_DAPI.tif]

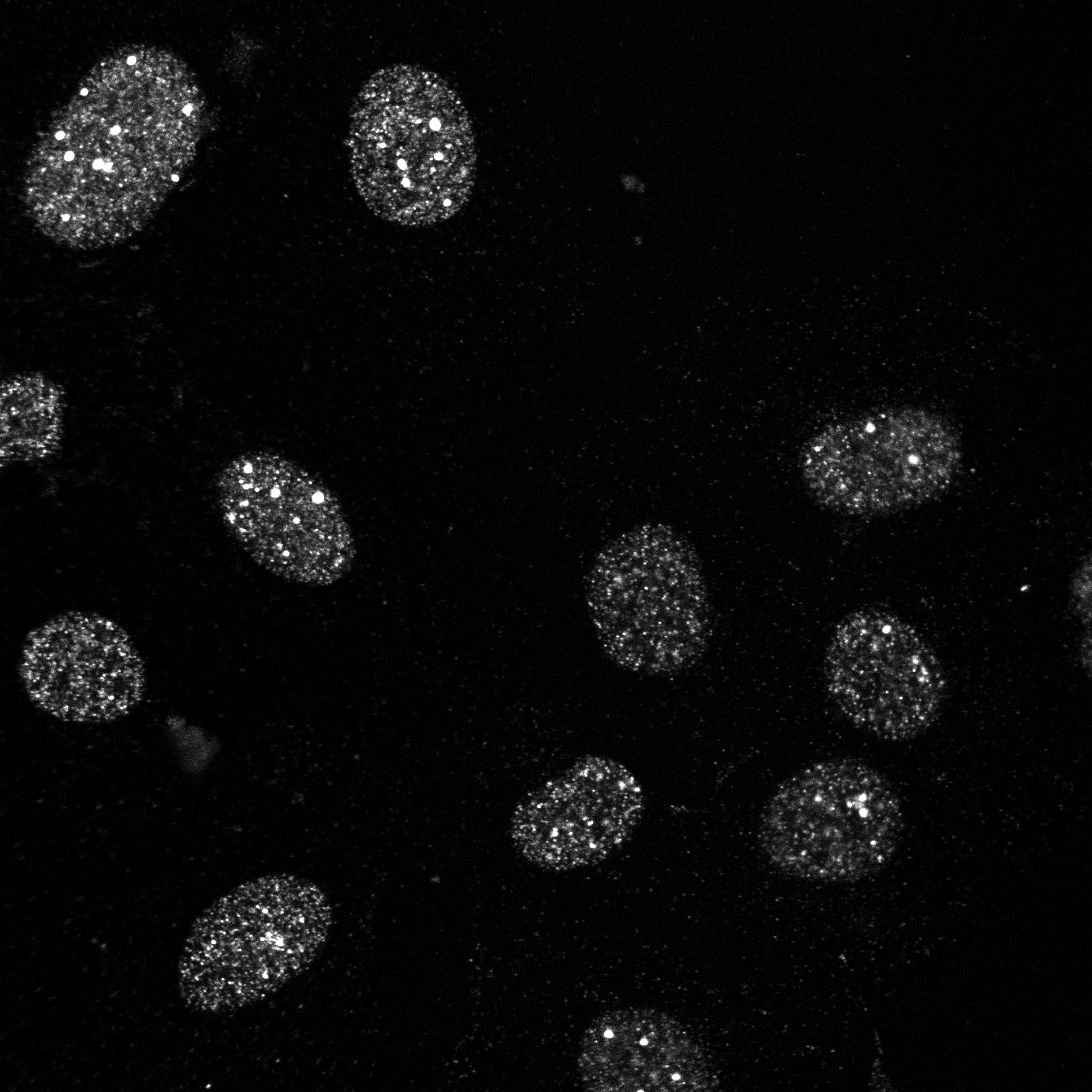

Supplement: Supplementary file 6 — Source data Fig. 6 [file 44318_2026_790_MOESM6_ESM.zip › Figure 6/Figure 6C_pRPA_TelC_U2OS_BLM_rescue/C3-U2OS_SLX4IP_KO_clone_2_siFANCM_pS33-RPA.tif]

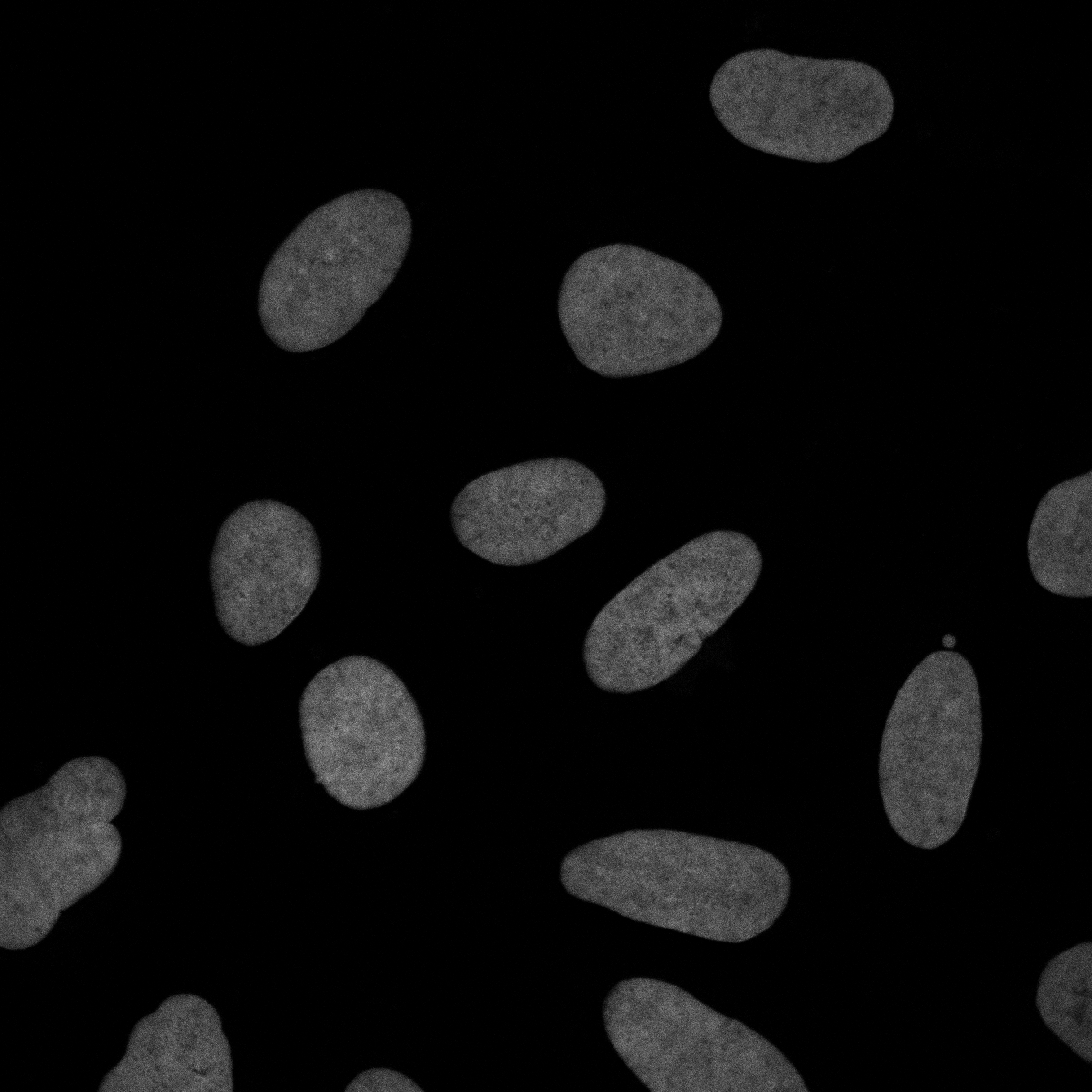

Supplement: Supplementary file 6 — Source data Fig. 6 [file 44318_2026_790_MOESM6_ESM.zip › Figure 6/Figure 6C_pRPA_TelC_U2OS_BLM_rescue/C1-U2OS_WT_siCTRL_DAPI.tif]

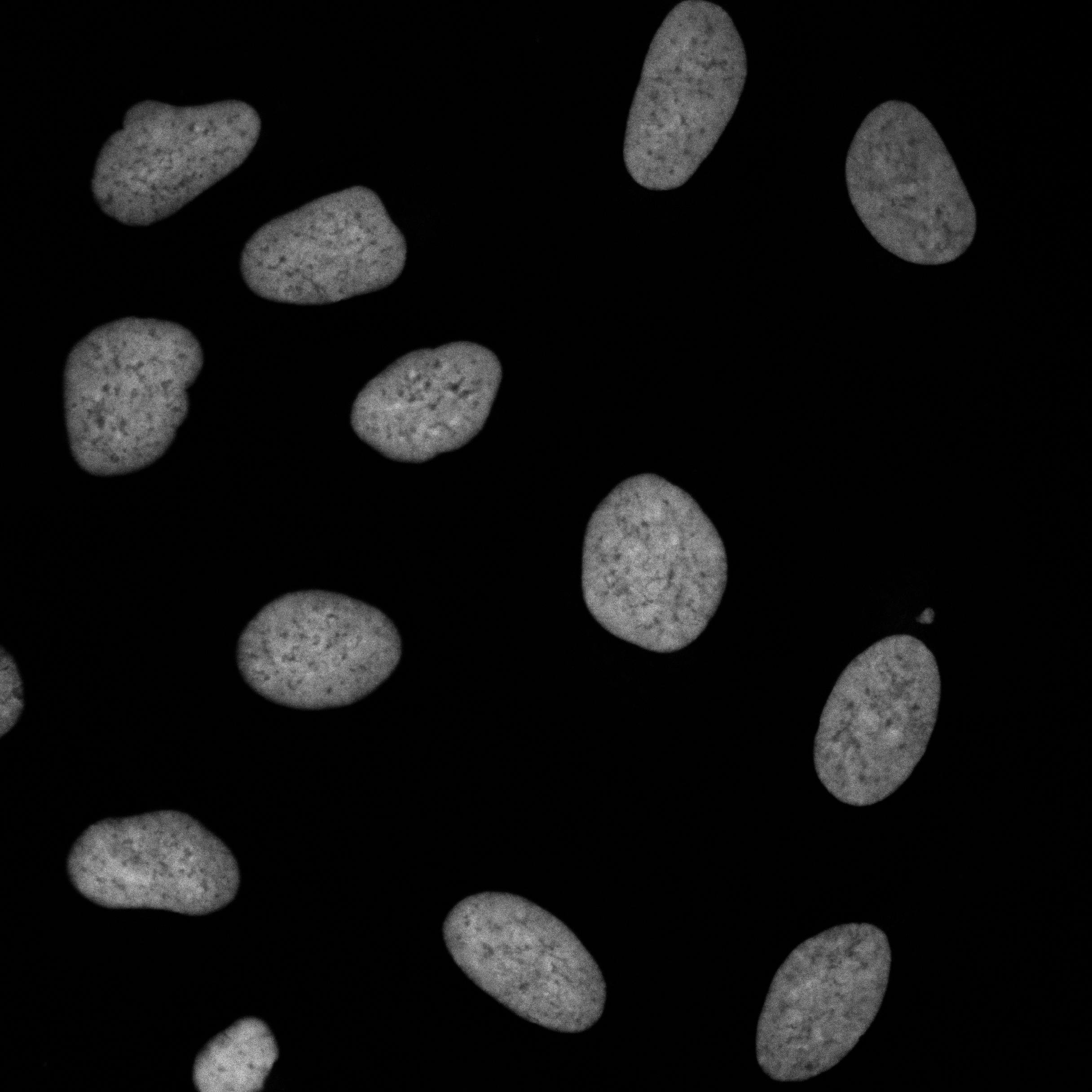

Supplement: Supplementary file 6 — Source data Fig. 6 [file 44318_2026_790_MOESM6_ESM.zip › Figure 6/Figure 6C_pRPA_TelC_U2OS_BLM_rescue/C1-U2OS_WT_siFANCM_siBLM_DAPI.tif]

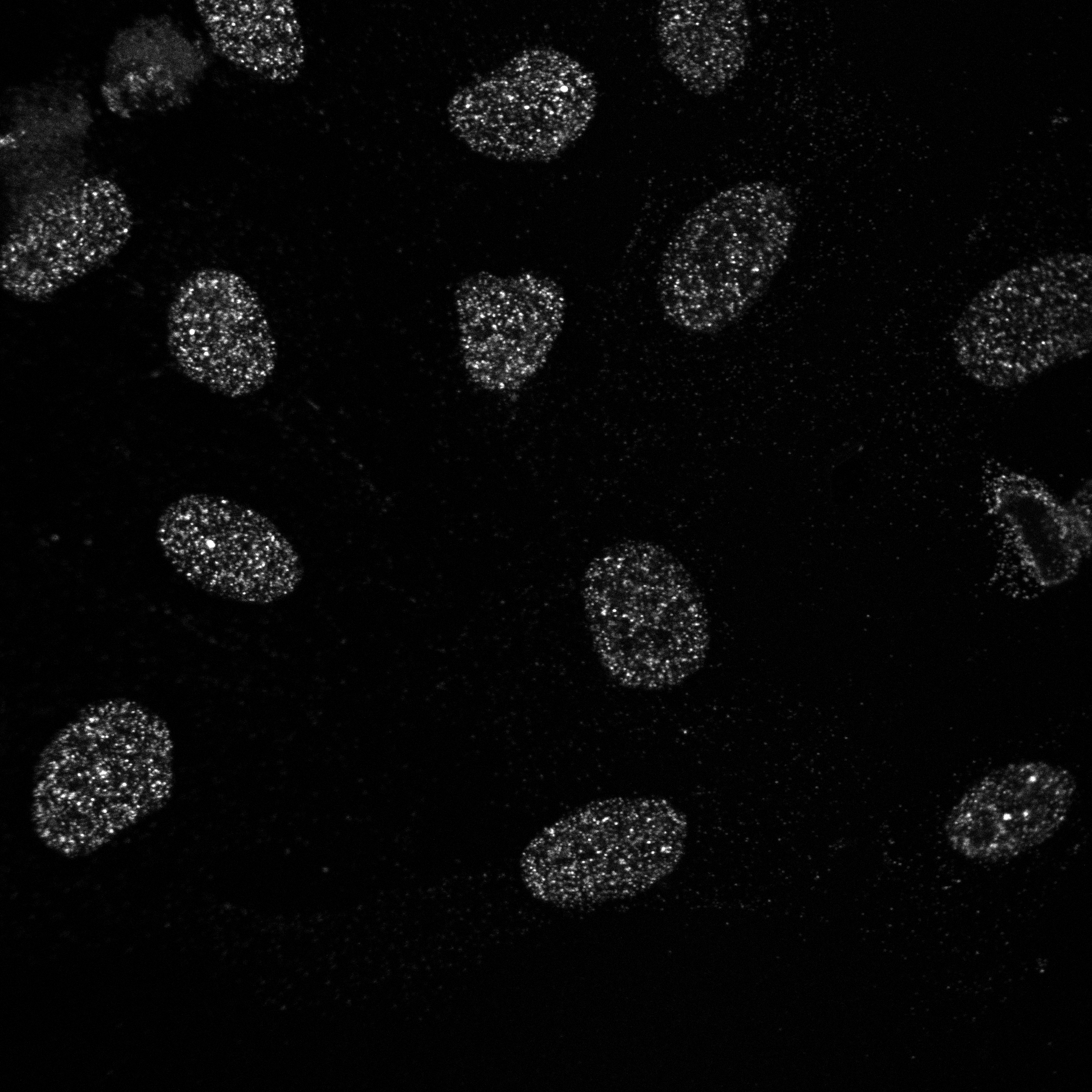

Supplement: Supplementary file 6 — Source data Fig. 6 [file 44318_2026_790_MOESM6_ESM.zip › Figure 6/Figure 6C_pRPA_TelC_U2OS_BLM_rescue/C3-U2OS_SLX4IP_KO_clone_1_siFANCM_siBLM_pS33-RPA.tif]

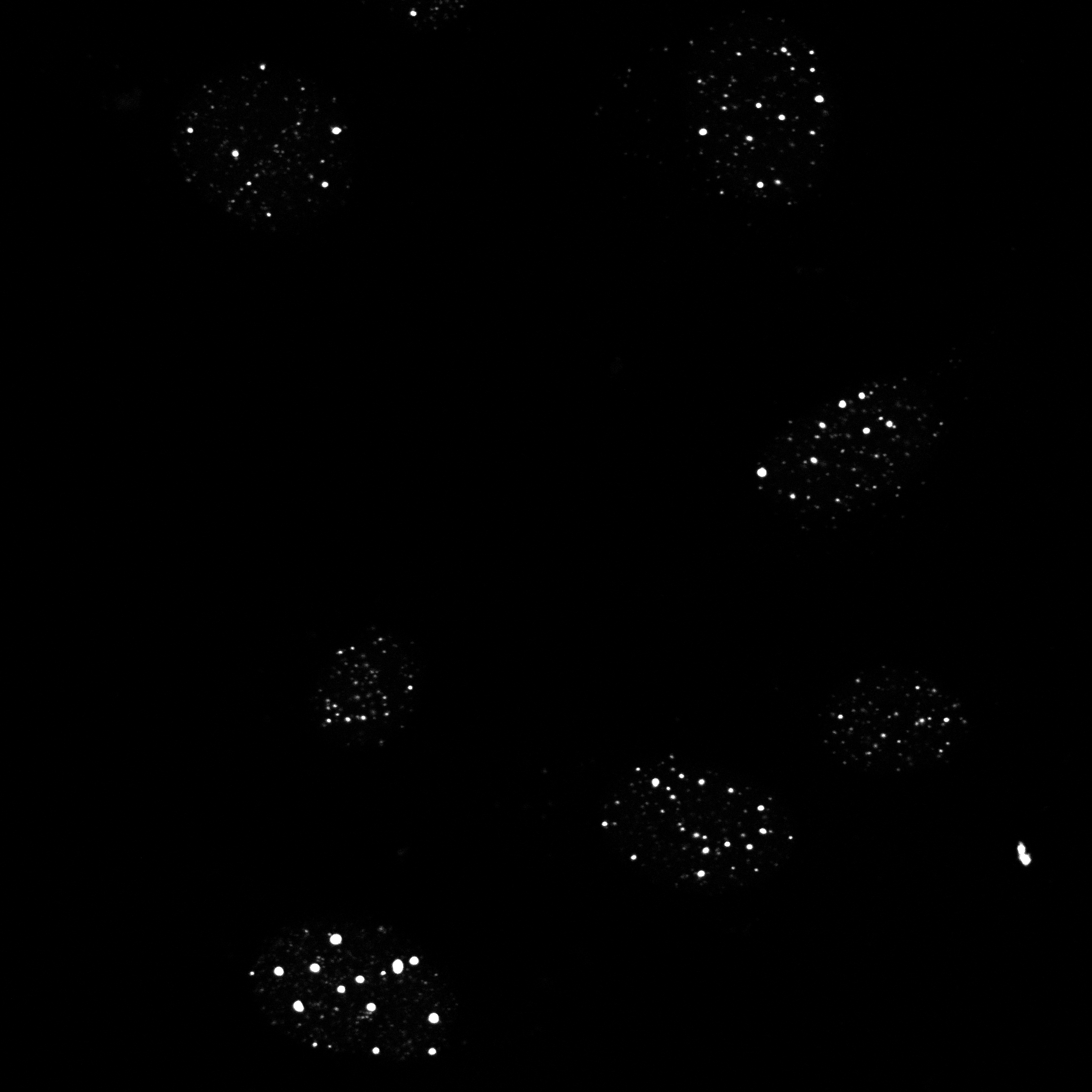

Supplement: Supplementary file 6 — Source data Fig. 6 [file 44318_2026_790_MOESM6_ESM.zip › Figure 6/Figure 6C_pRPA_TelC_U2OS_BLM_rescue/C4-U2OS_SLX4IP_KO_clone_1_siFANCM_TelC.tif]

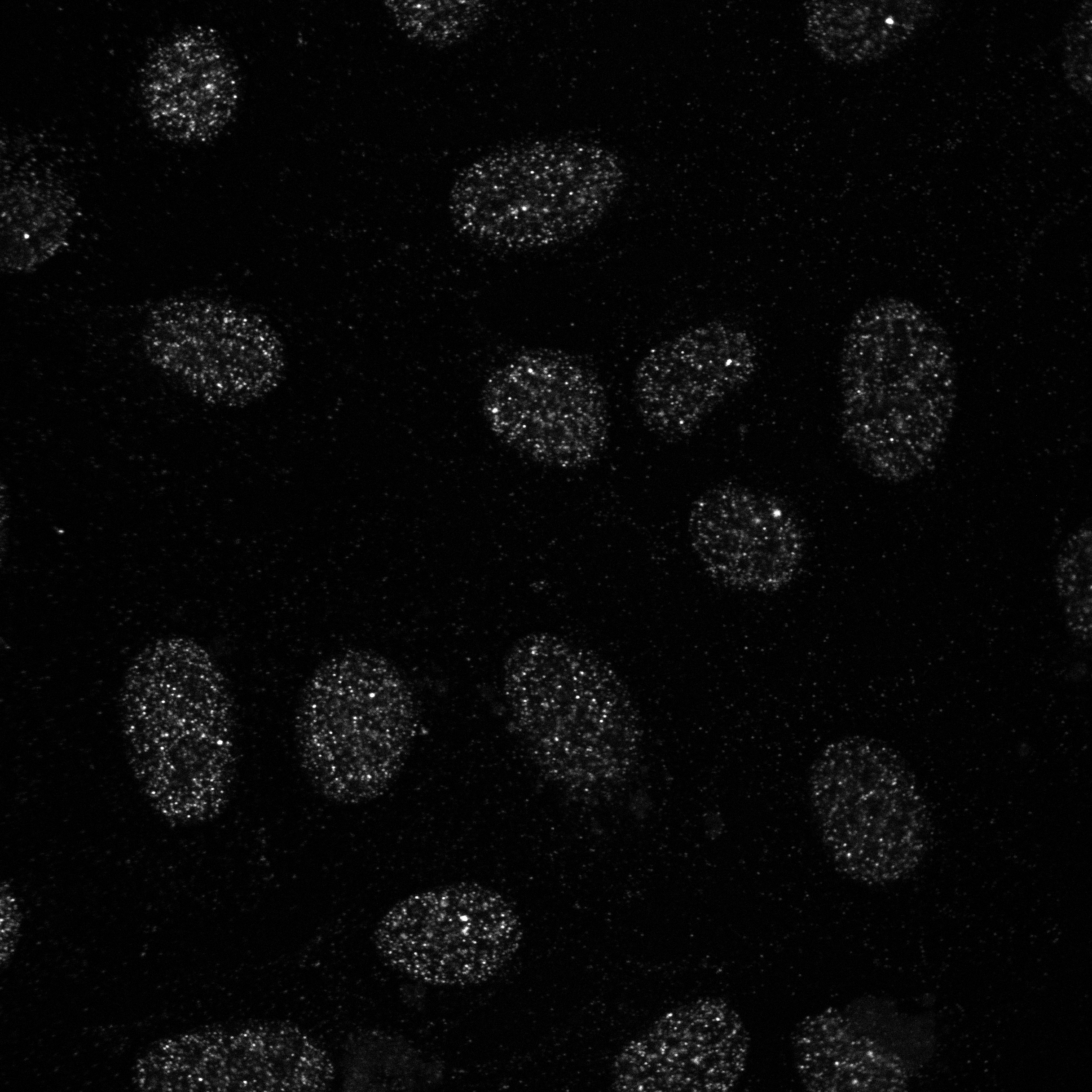

Supplement: Supplementary file 6 — Source data Fig. 6 [file 44318_2026_790_MOESM6_ESM.zip › Figure 6/Figure 6C_pRPA_TelC_U2OS_BLM_rescue/C3-U2OS_SLX4IP_KO_clone_1_siBLM_pS33-RPA.tif]

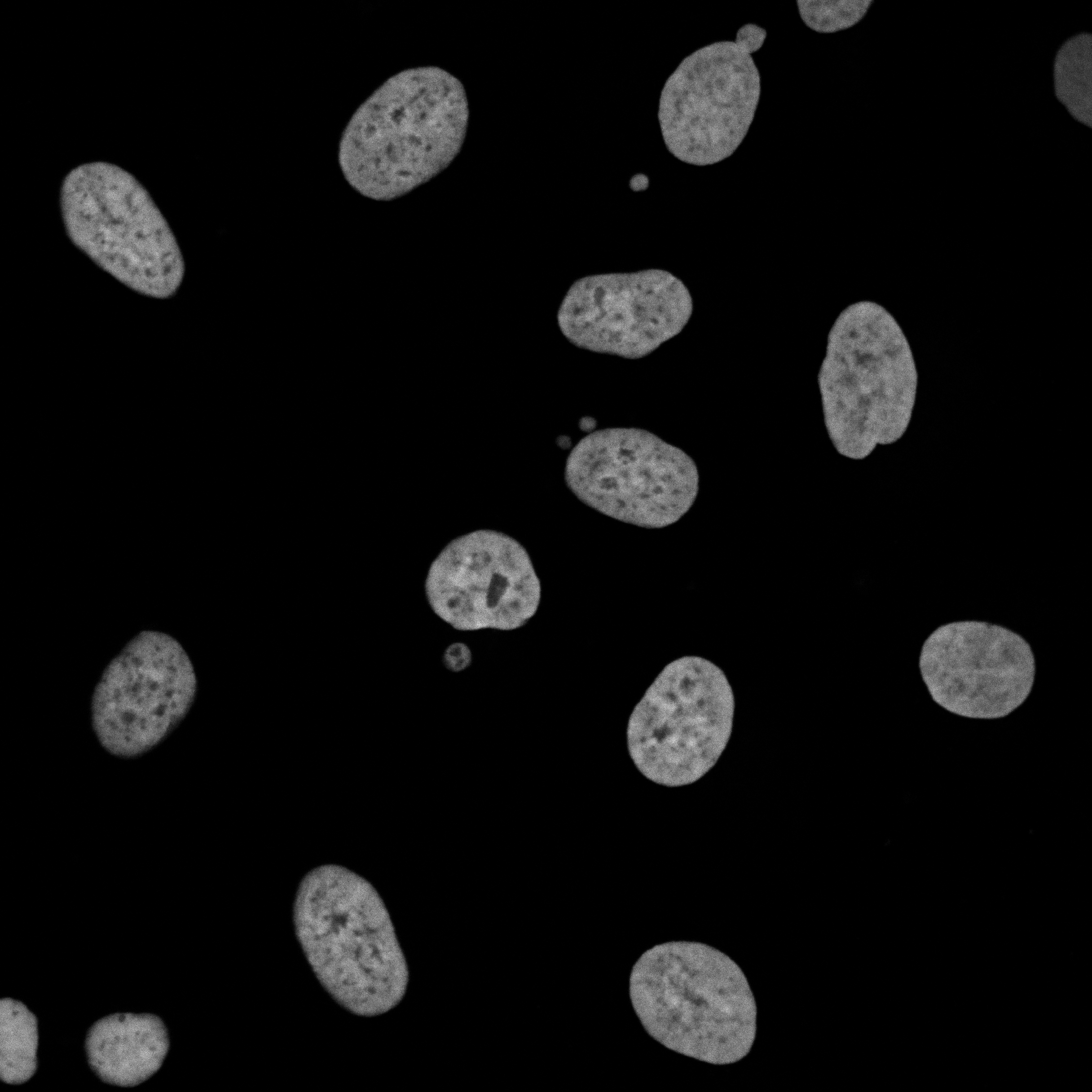

Supplement: Supplementary file 6 — Source data Fig. 6 [file 44318_2026_790_MOESM6_ESM.zip › Figure 6/Figure 6C_pRPA_TelC_U2OS_BLM_rescue/C1-U2OS_SLX4IP_KO_clone_2_siFANCM_siBLM_DAPI.tif]

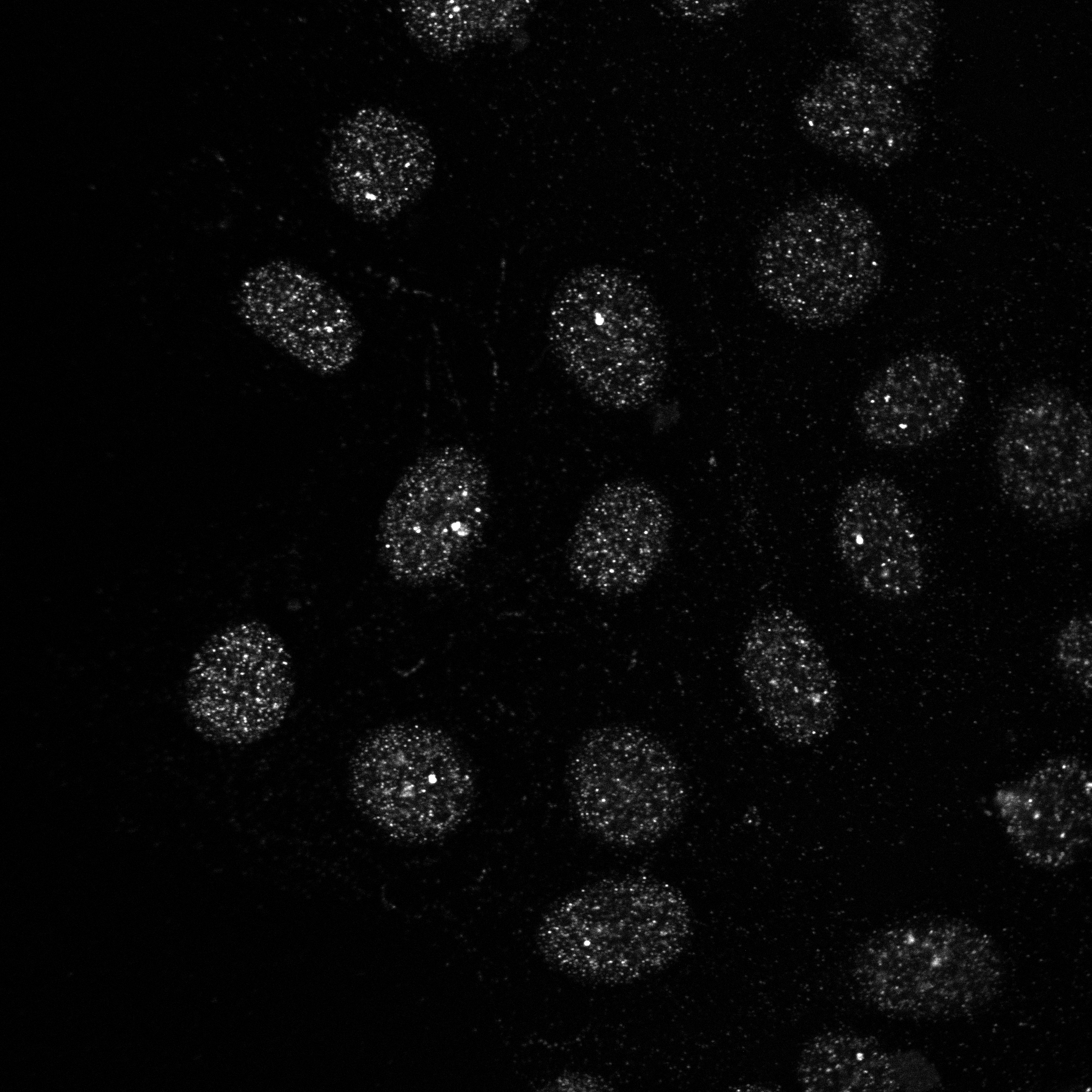

Supplement: Supplementary file 6 — Source data Fig. 6 [file 44318_2026_790_MOESM6_ESM.zip › Figure 6/Figure 6C_pRPA_TelC_U2OS_BLM_rescue/C3-U2OS_SLX4IP_KO_clone_2_siBLM_pS33-RPA.tif]

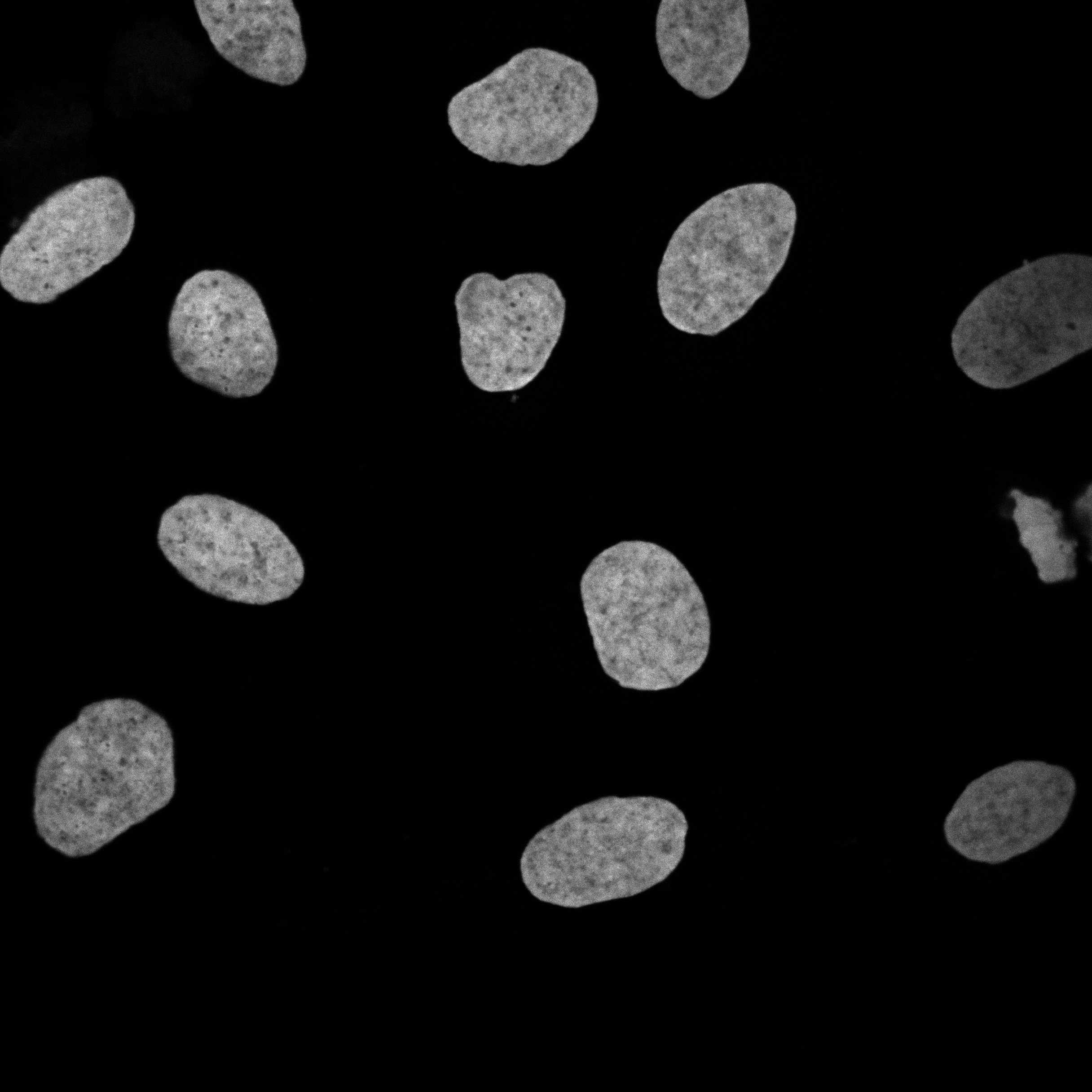

Supplement: Supplementary file 6 — Source data Fig. 6 [file 44318_2026_790_MOESM6_ESM.zip › Figure 6/Figure 6C_pRPA_TelC_U2OS_BLM_rescue/C1-U2OS_SLX4IP_KO_clone_1_siFANCM_siBLM_DAPI.tif]

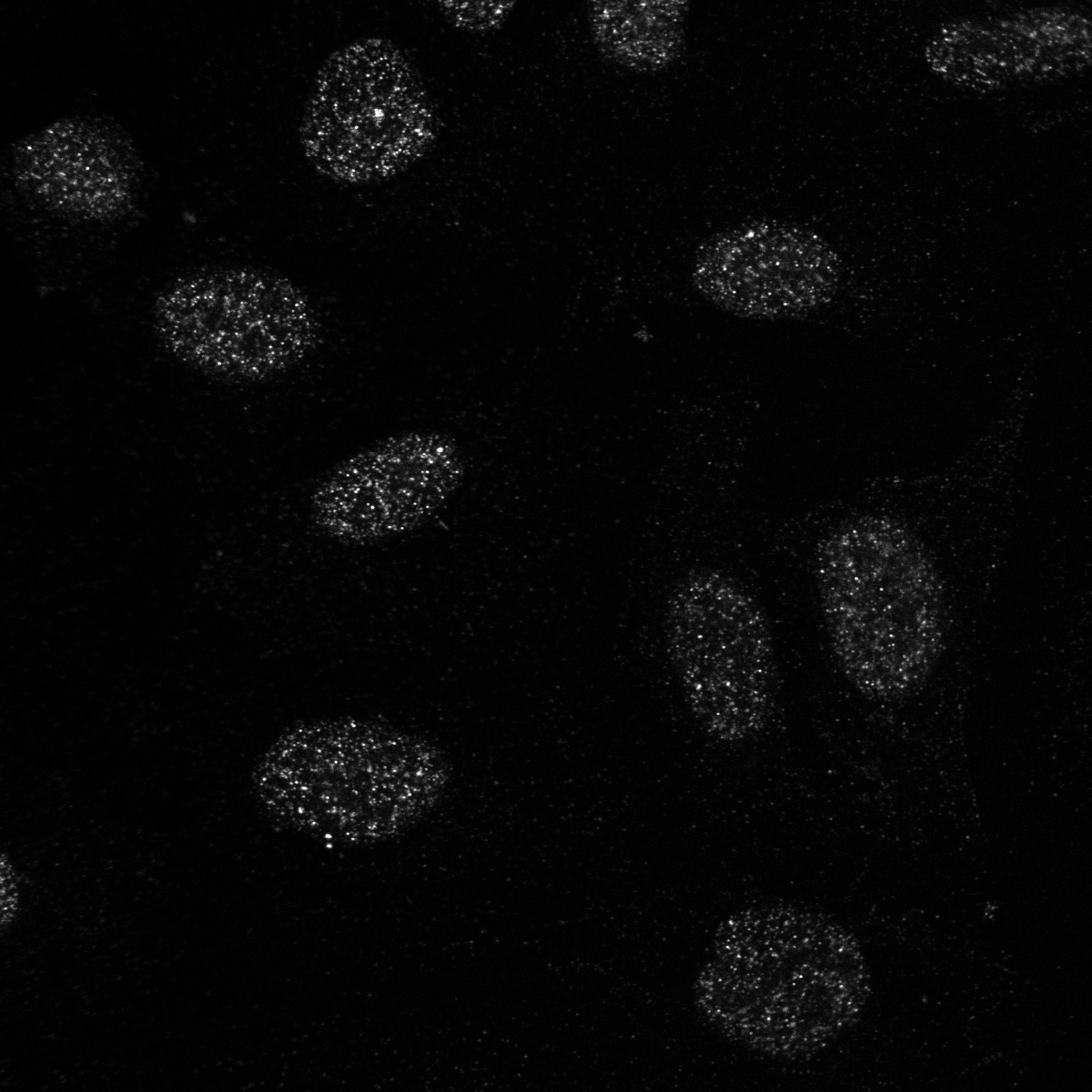

Supplement: Supplementary file 6 — Source data Fig. 6 [file 44318_2026_790_MOESM6_ESM.zip › Figure 6/Figure 6C_pRPA_TelC_U2OS_BLM_rescue/C3-U2OS_WT_siBLM_pS33-RPA.tif]

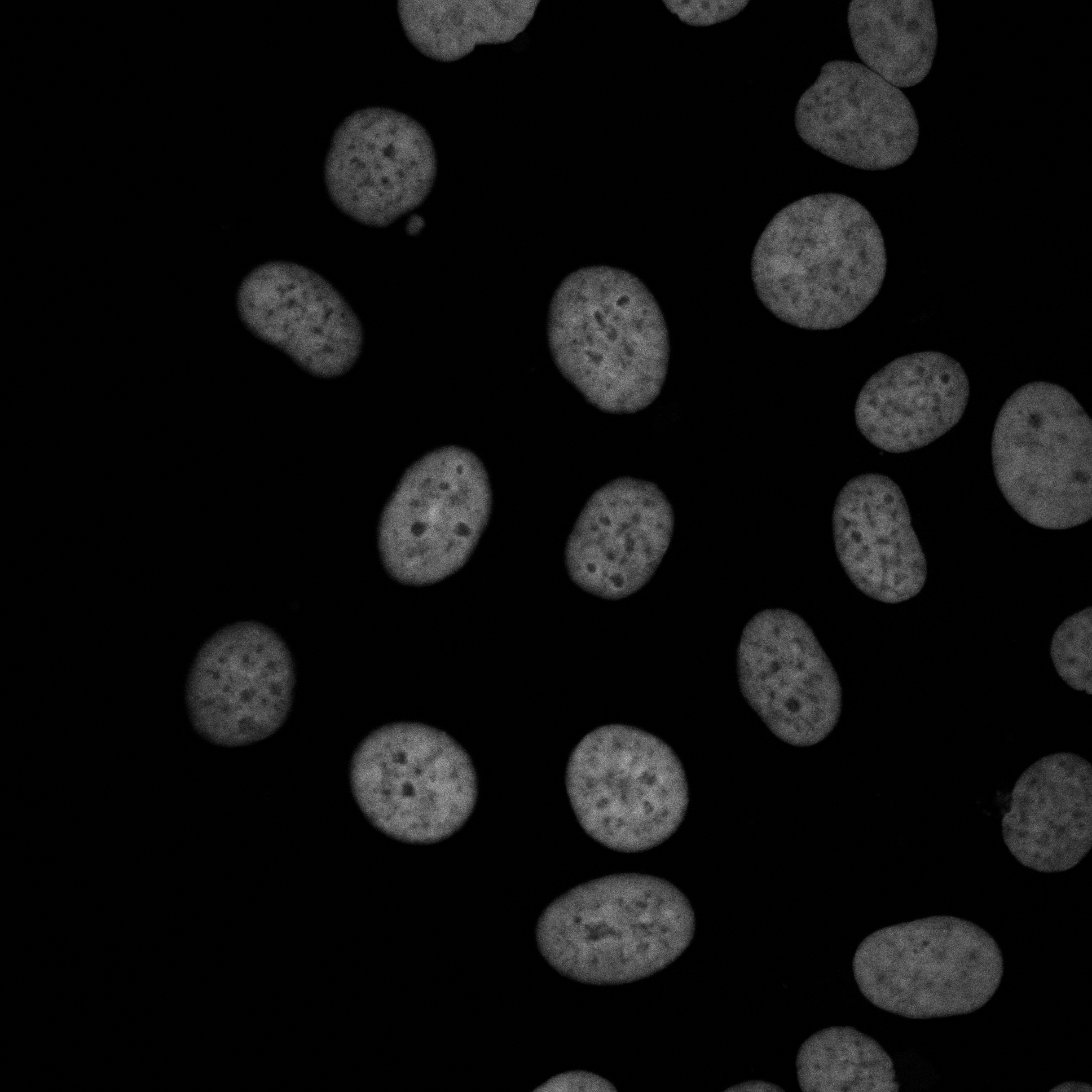

Supplement: Supplementary file 6 — Source data Fig. 6 [file 44318_2026_790_MOESM6_ESM.zip › Figure 6/Figure 6C_pRPA_TelC_U2OS_BLM_rescue/C1-U2OS_SLX4IP_KO_clone_2_siBLM_DAPI.tif]

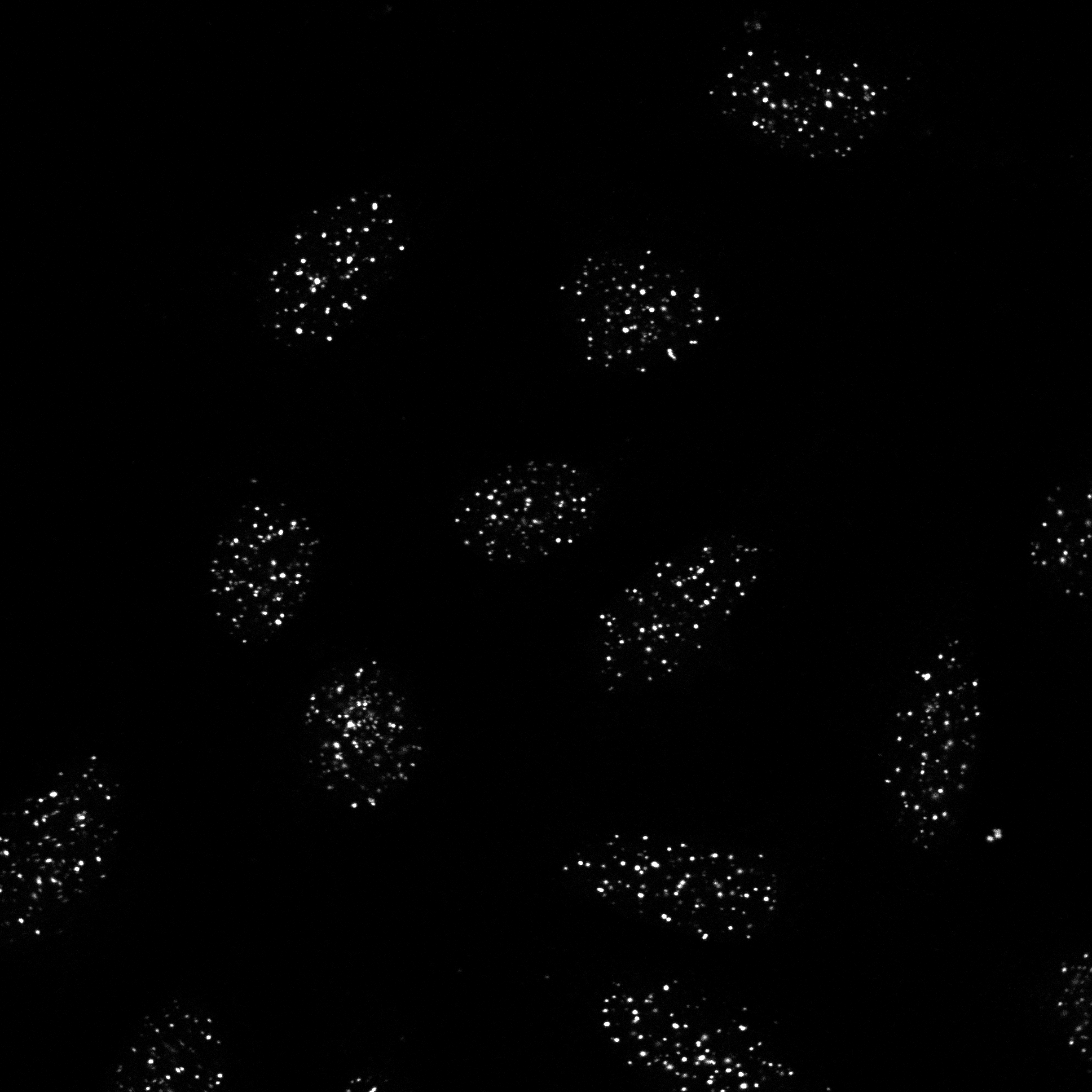

Supplement: Supplementary file 6 — Source data Fig. 6 [file 44318_2026_790_MOESM6_ESM.zip › Figure 6/Figure 6C_pRPA_TelC_U2OS_BLM_rescue/C4-U2OS_WT_siCTRL_TelC.tif]

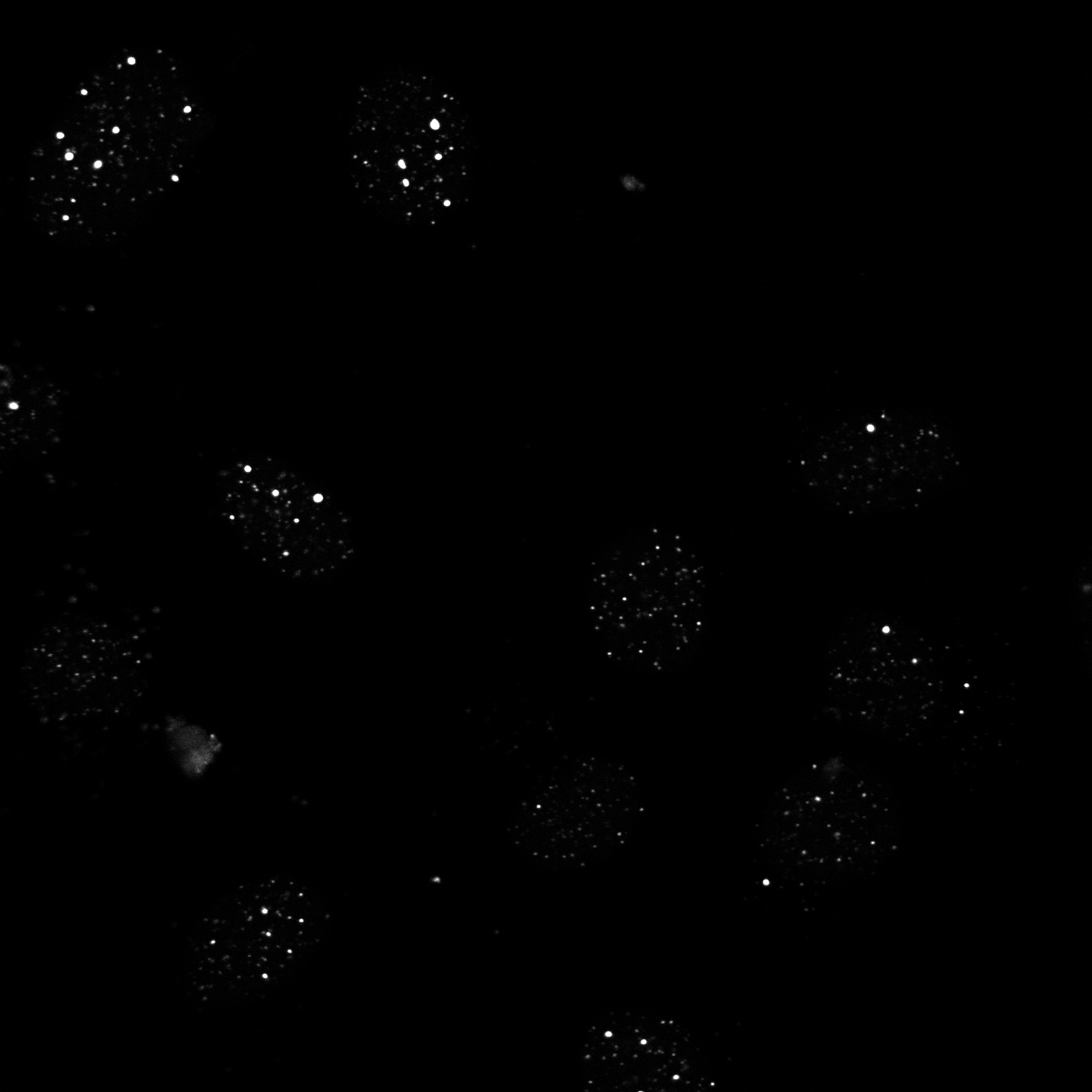

Supplement: Supplementary file 6 — Source data Fig. 6 [file 44318_2026_790_MOESM6_ESM.zip › Figure 6/Figure 6C_pRPA_TelC_U2OS_BLM_rescue/C4-U2OS_SLX4IP_KO_clone_2_siFANCM_TelC.tif]

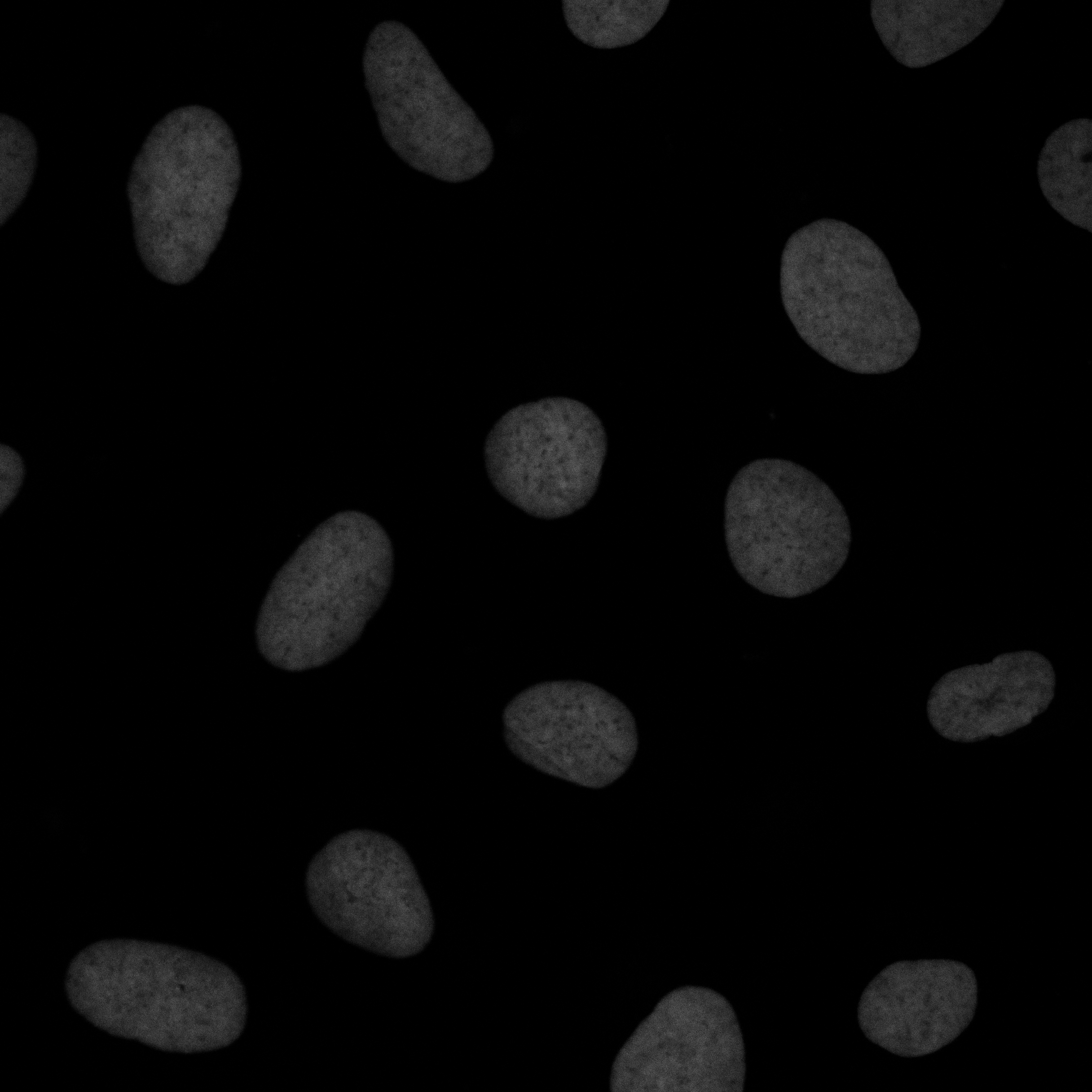

Supplement: Supplementary file 6 — Source data Fig. 6 [file 44318_2026_790_MOESM6_ESM.zip › Figure 6/Figure 6C_pRPA_TelC_U2OS_BLM_rescue/C1-U2OS_SLX4IP_KO_clone_1_siCTRL_DAPI.tif]

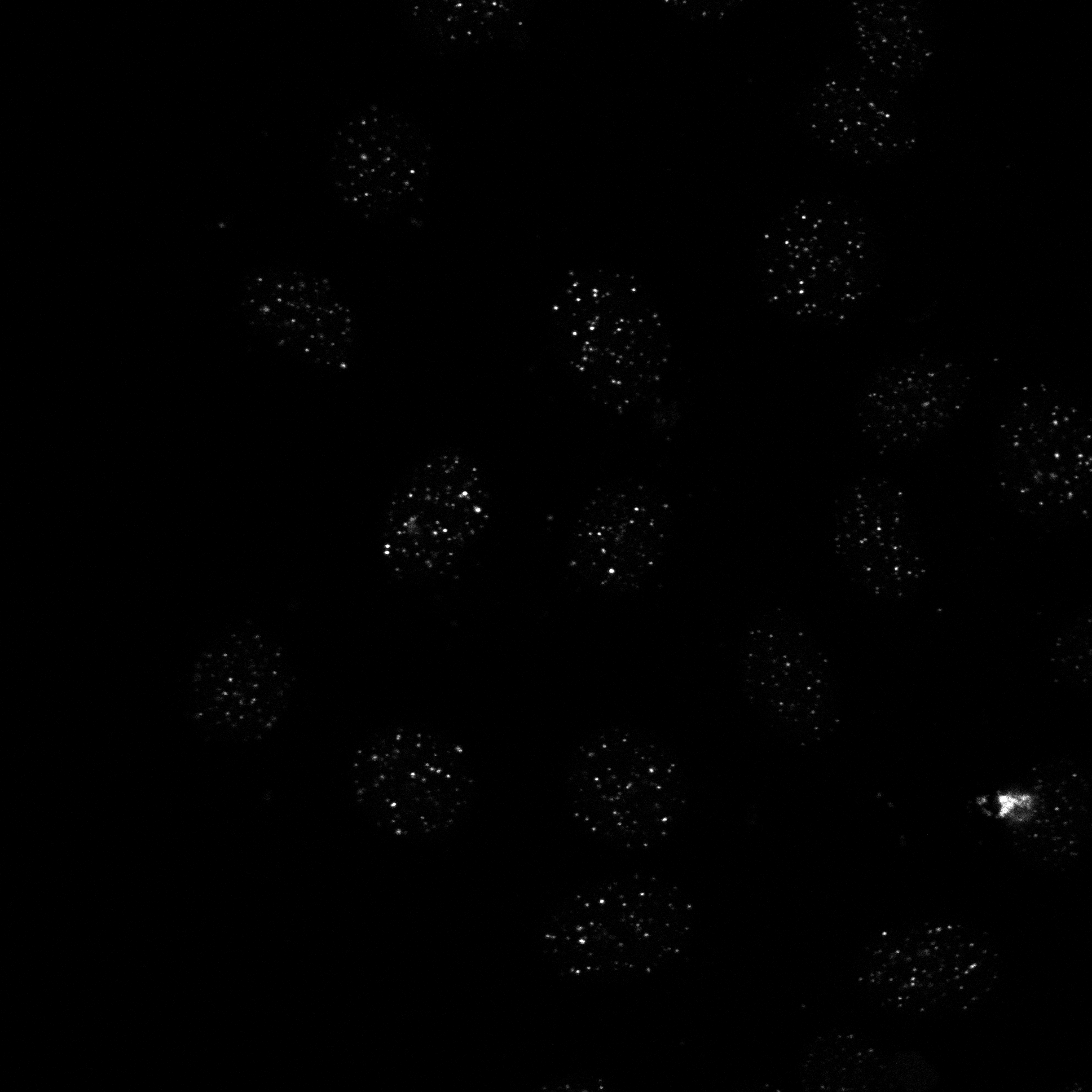

Supplement: Supplementary file 6 — Source data Fig. 6 [file 44318_2026_790_MOESM6_ESM.zip › Figure 6/Figure 6C_pRPA_TelC_U2OS_BLM_rescue/C4-U2OS_SLX4IP_KO_clone_2_siBLM_TelC.tif]

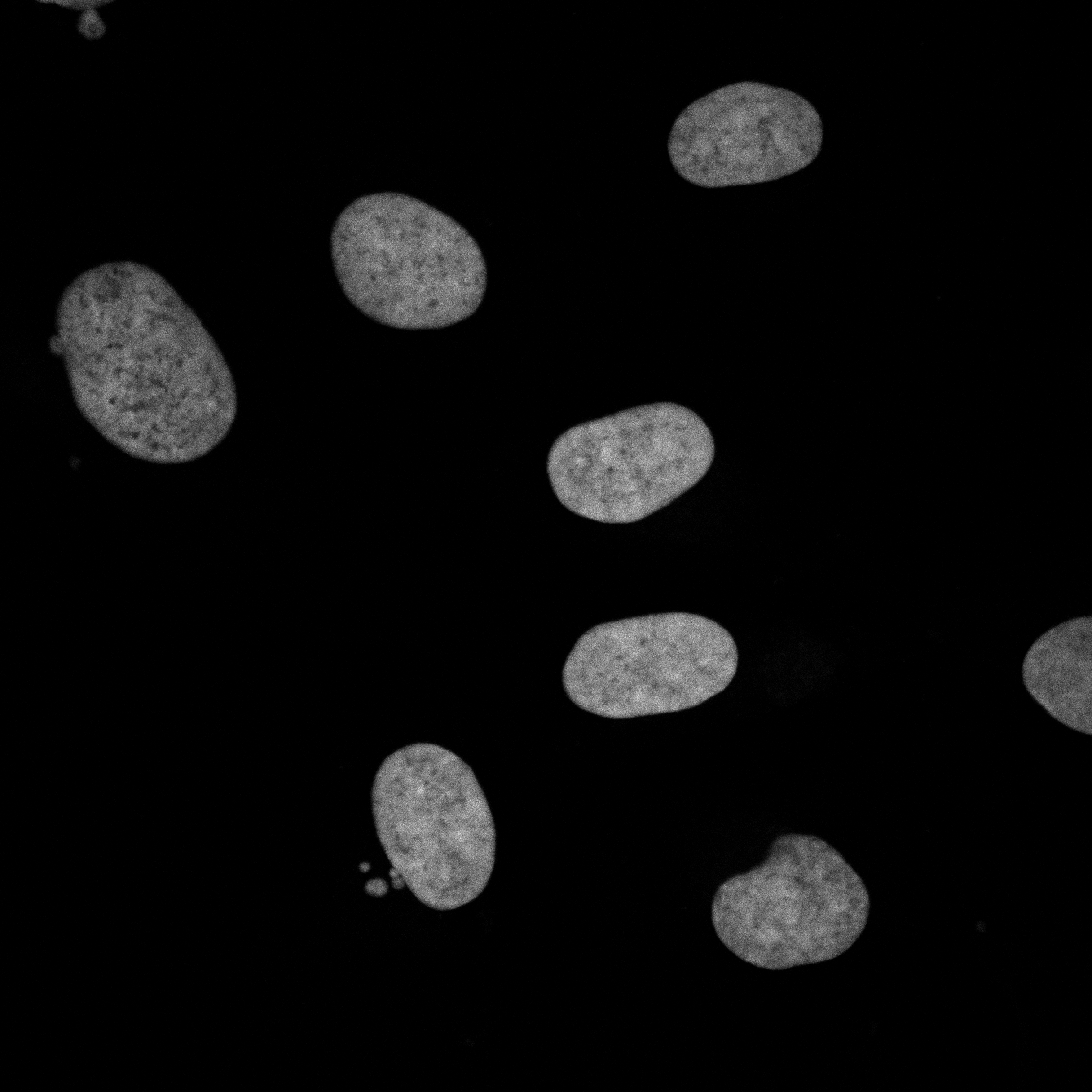

Supplement: Supplementary file 6 — Source data Fig. 6 [file 44318_2026_790_MOESM6_ESM.zip › Figure 6/Figure 6C_pRPA_TelC_U2OS_BLM_rescue/C1-U2OS_WT_siFANCM_DAPI.tif]

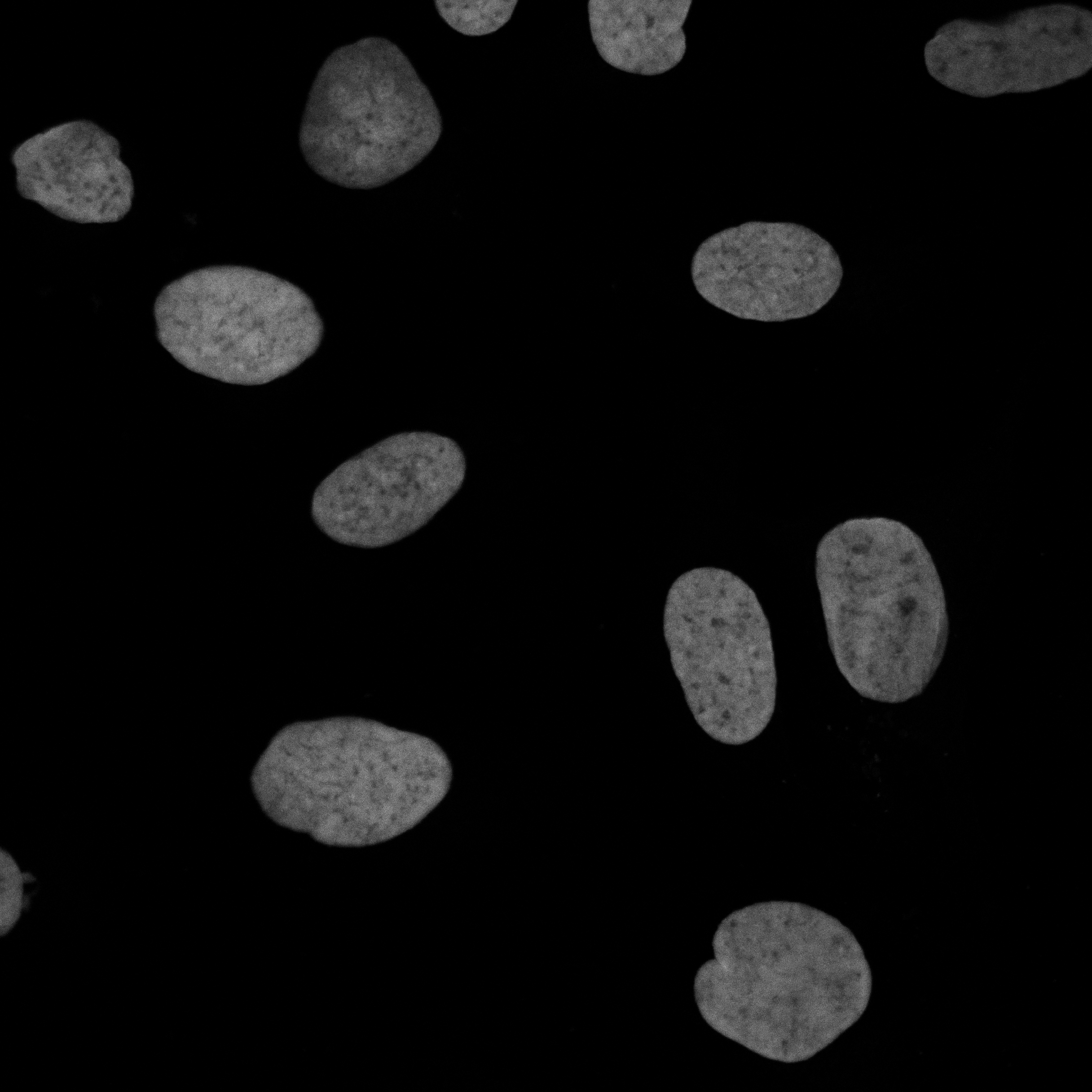

Supplement: Supplementary file 6 — Source data Fig. 6 [file 44318_2026_790_MOESM6_ESM.zip › Figure 6/Figure 6C_pRPA_TelC_U2OS_BLM_rescue/C1-U2OS_WT_siBLM_DAPI.tif]

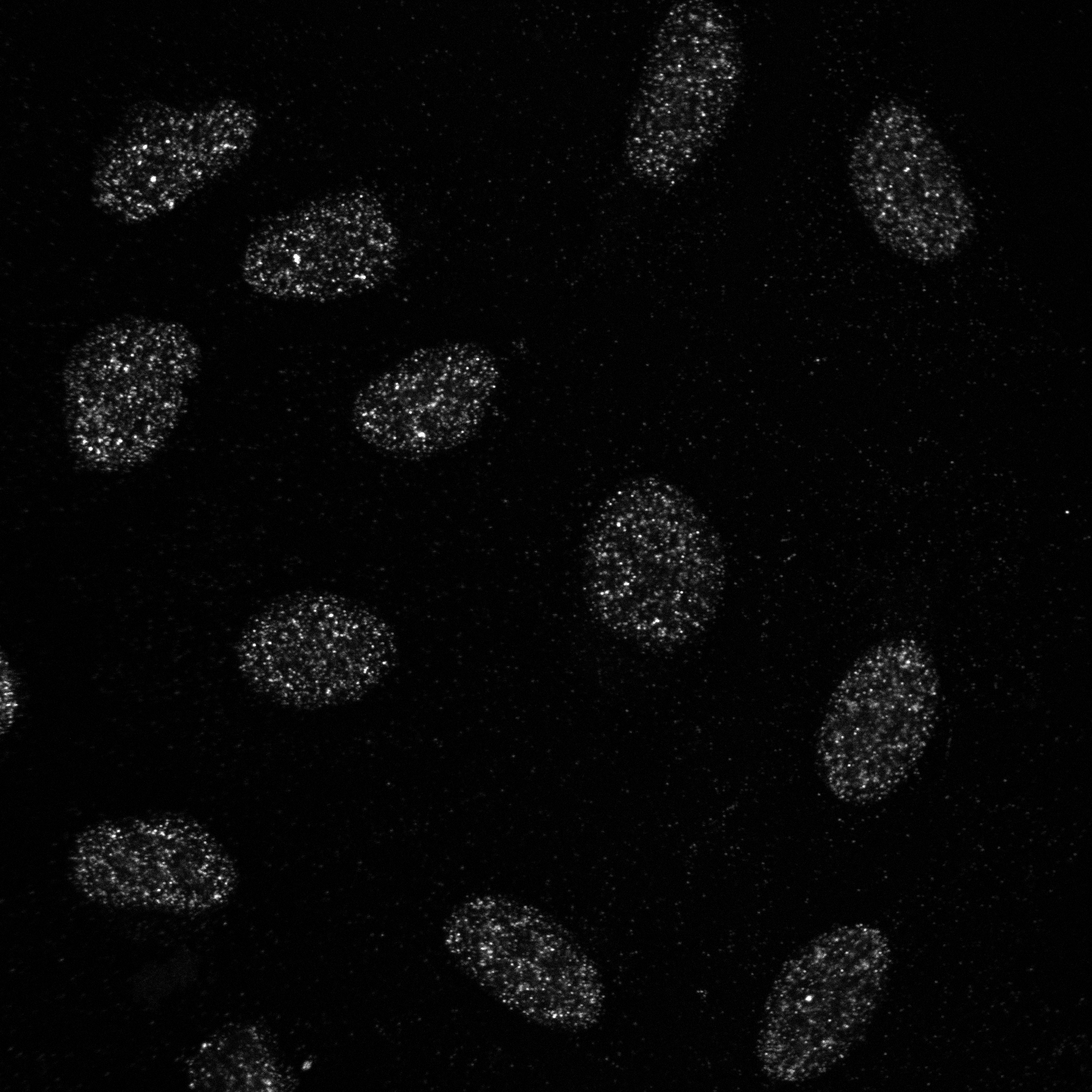

Supplement: Supplementary file 6 — Source data Fig. 6 [file 44318_2026_790_MOESM6_ESM.zip › Figure 6/Figure 6C_pRPA_TelC_U2OS_BLM_rescue/C3-U2OS_WT_siFANCM_siBLM_pS33-RPA.tif]

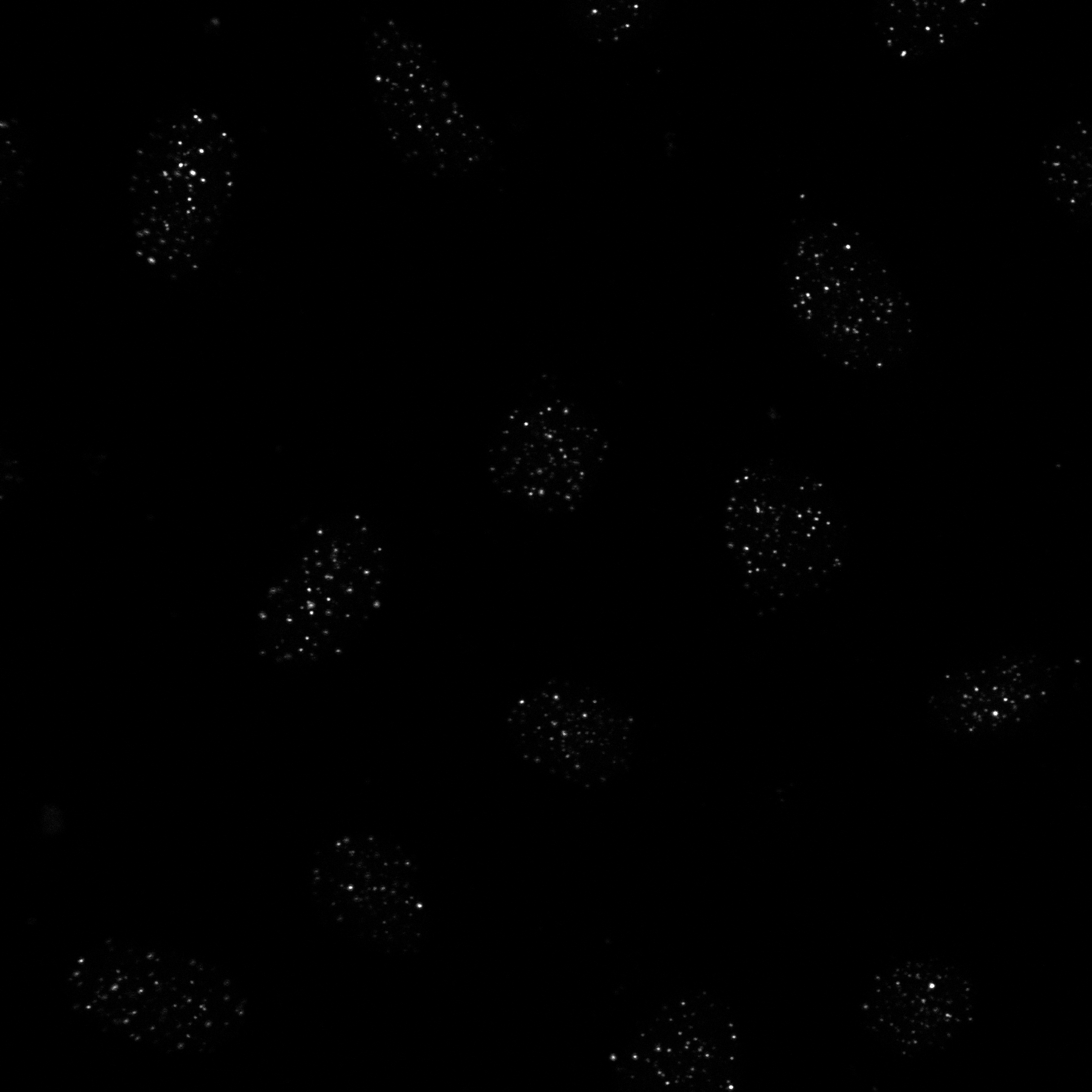

Supplement: Supplementary file 6 — Source data Fig. 6 [file 44318_2026_790_MOESM6_ESM.zip › Figure 6/Figure 6C_pRPA_TelC_U2OS_BLM_rescue/C4-U2OS_SLX4IP_KO_clone_1_siCTRL_TelC.tif]

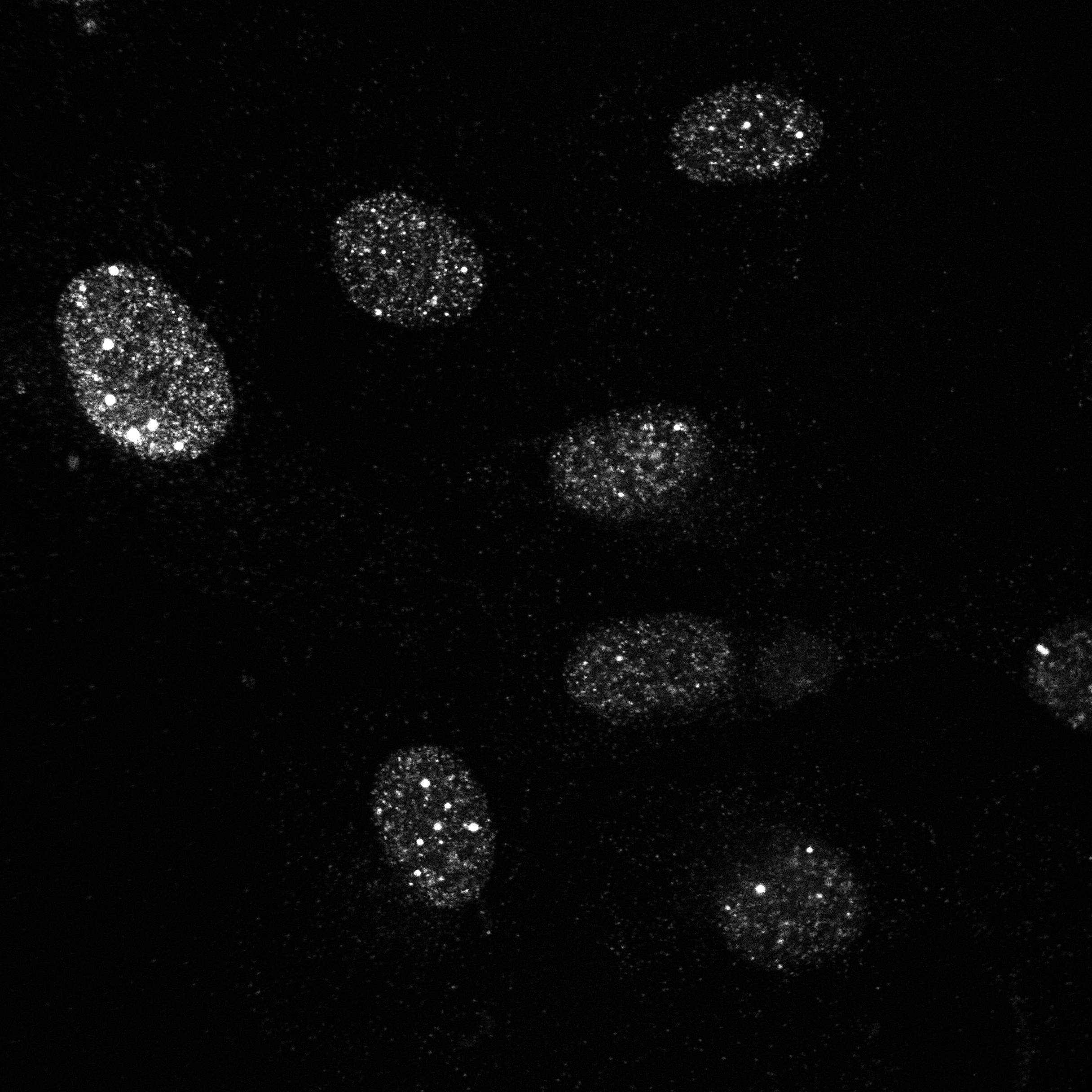

Supplement: Supplementary file 6 — Source data Fig. 6 [file 44318_2026_790_MOESM6_ESM.zip › Figure 6/Figure 6C_pRPA_TelC_U2OS_BLM_rescue/C3-U2OS_WT_siFANCM_pS33-RPA.tif]

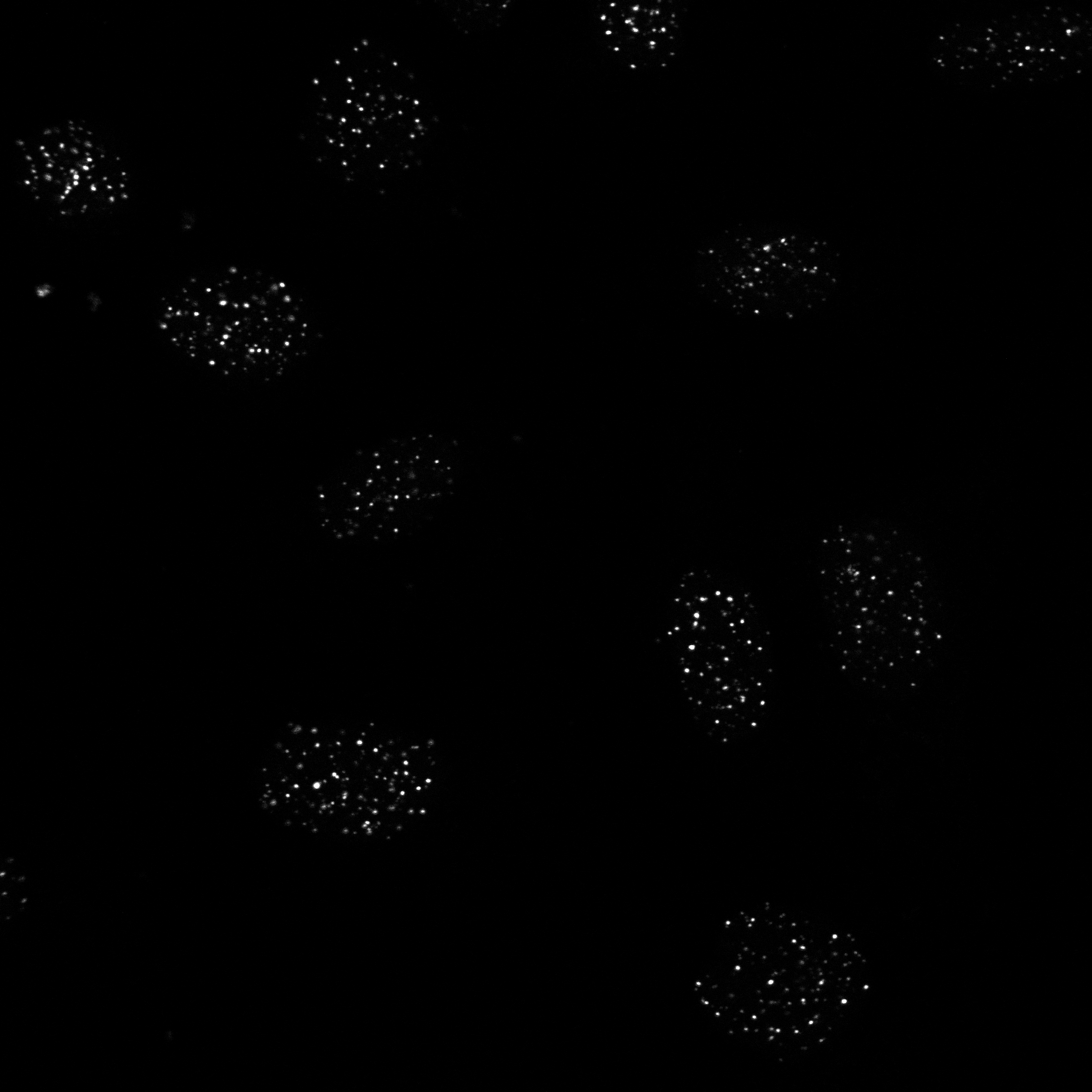

Supplement: Supplementary file 6 — Source data Fig. 6 [file 44318_2026_790_MOESM6_ESM.zip › Figure 6/Figure 6C_pRPA_TelC_U2OS_BLM_rescue/C4-U2OS_WT_siBLM_TelC.tif]

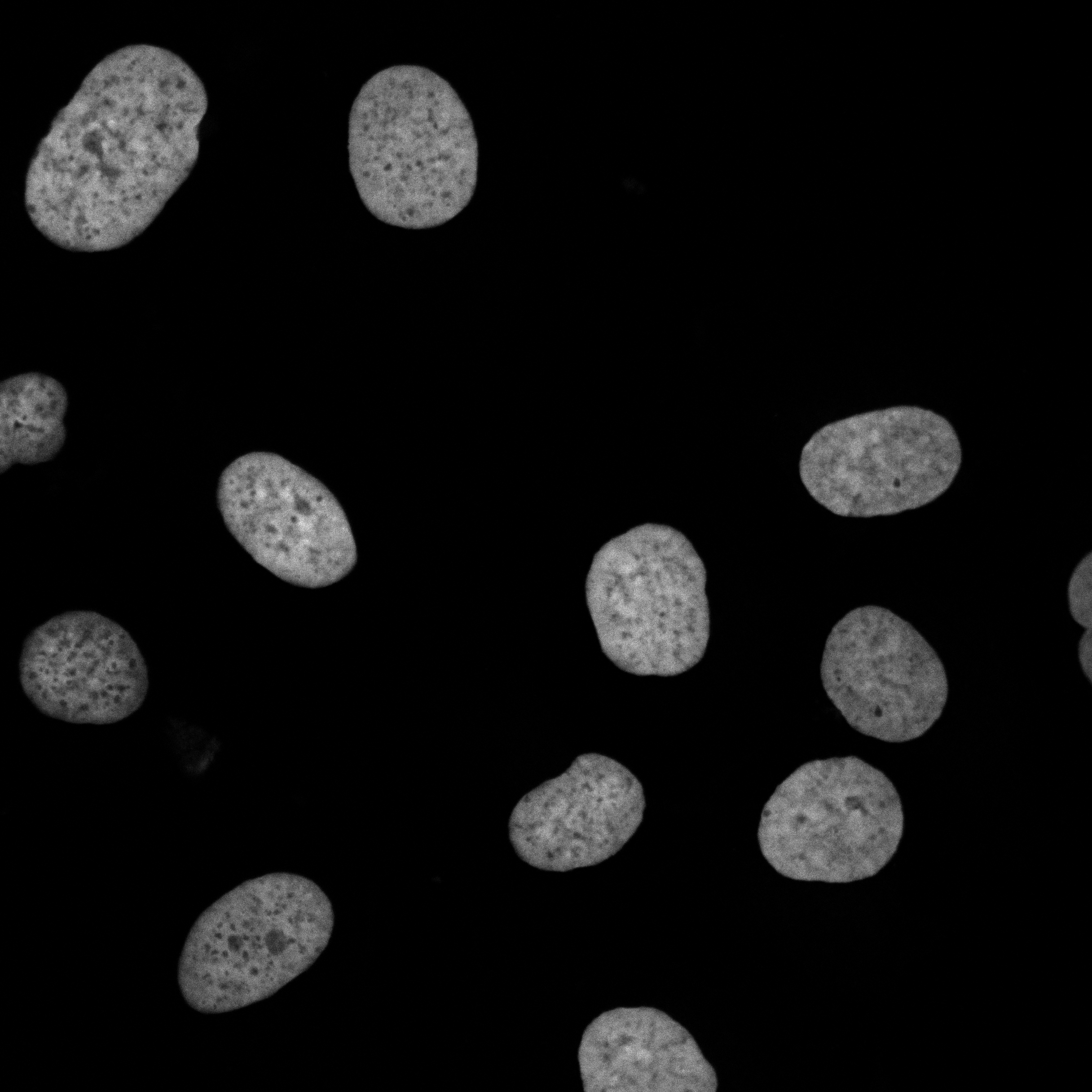

Supplement: Supplementary file 6 — Source data Fig. 6 [file 44318_2026_790_MOESM6_ESM.zip › Figure 6/Figure 6C_pRPA_TelC_U2OS_BLM_rescue/C1-U2OS_SLX4IP_KO_clone_2_siFANCM_DAPI.tif]

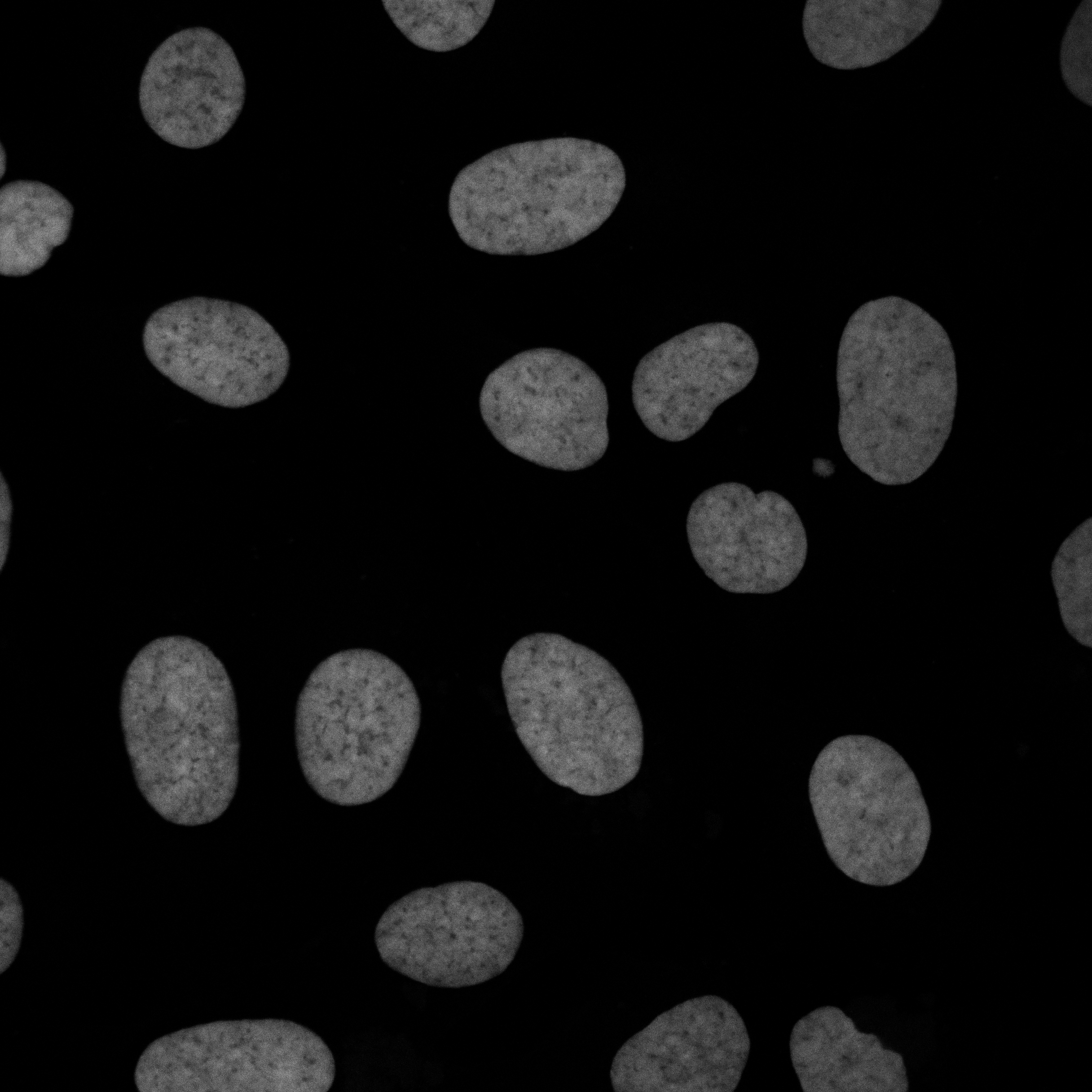

Supplement: Supplementary file 6 — Source data Fig. 6 [file 44318_2026_790_MOESM6_ESM.zip › Figure 6/Figure 6C_pRPA_TelC_U2OS_BLM_rescue/C1-U2OS_SLX4IP_KO_clone_1_siBLM_DAPI.tif]

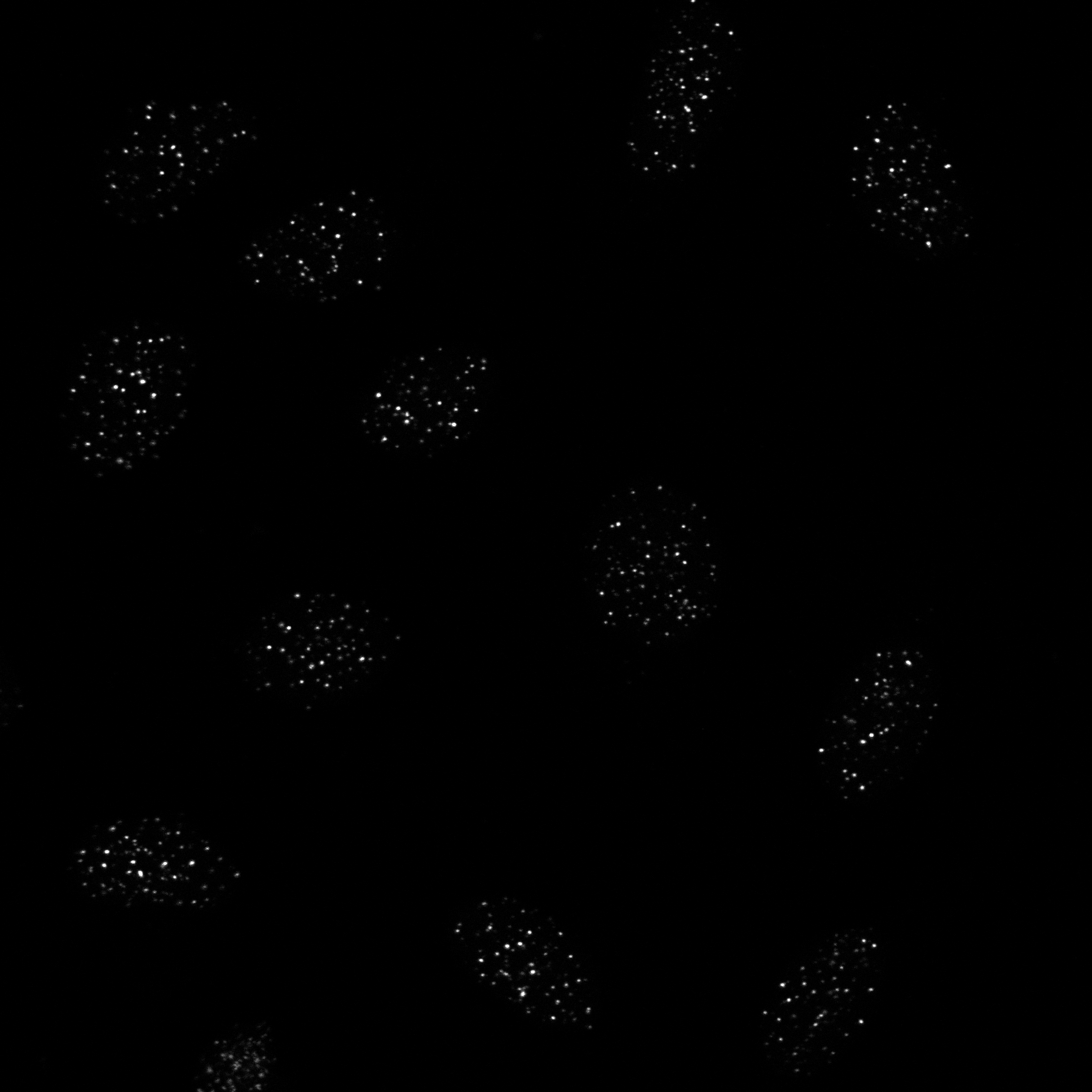

Supplement: Supplementary file 6 — Source data Fig. 6 [file 44318_2026_790_MOESM6_ESM.zip › Figure 6/Figure 6C_pRPA_TelC_U2OS_BLM_rescue/C4-U2OS_WT_siFANCM_siBLM_TelC.tif]

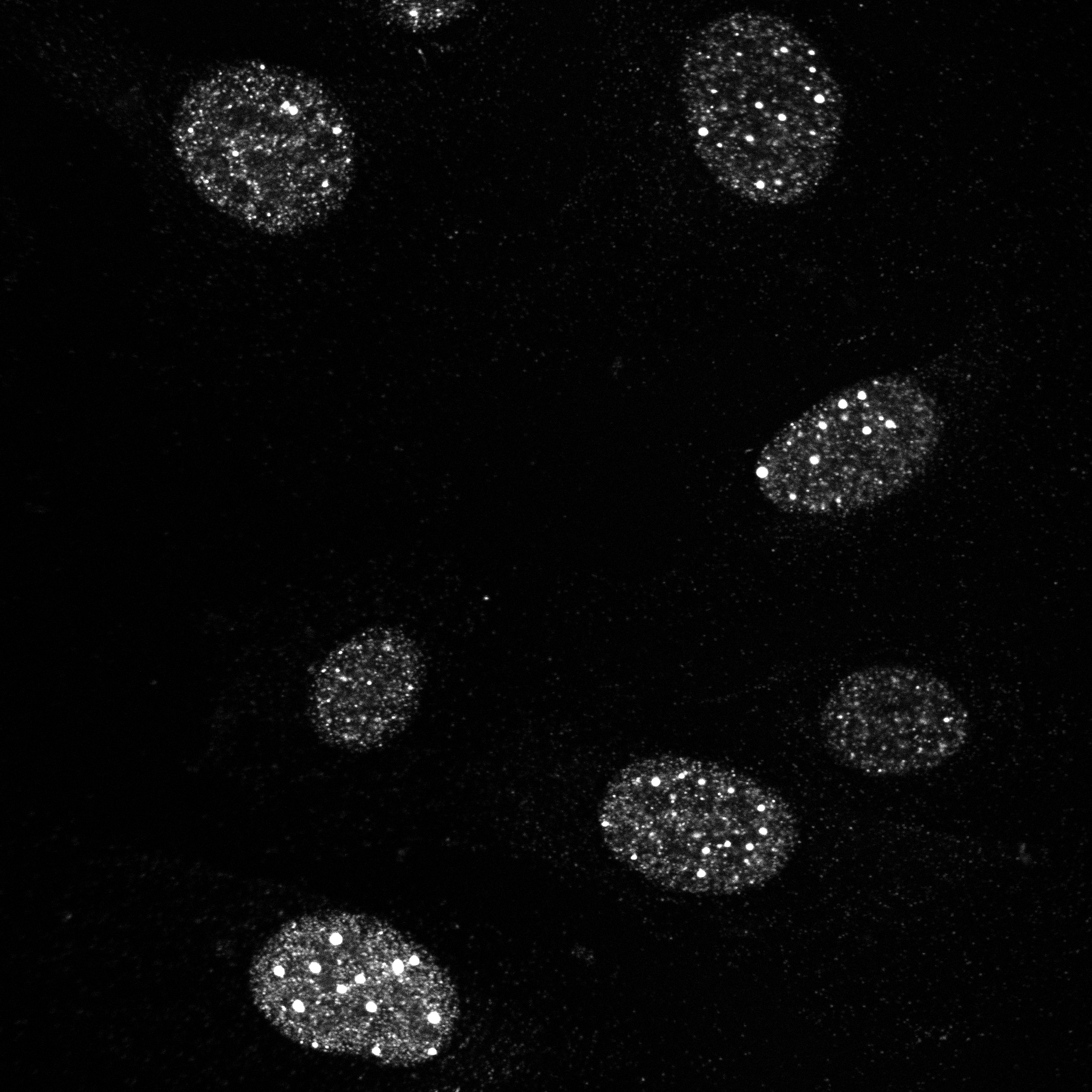

Supplement: Supplementary file 6 — Source data Fig. 6 [file 44318_2026_790_MOESM6_ESM.zip › Figure 6/Figure 6C_pRPA_TelC_U2OS_BLM_rescue/C3-U2OS_SLX4IP_KO_clone_1_siFANCM_pS33-RPA.tif]

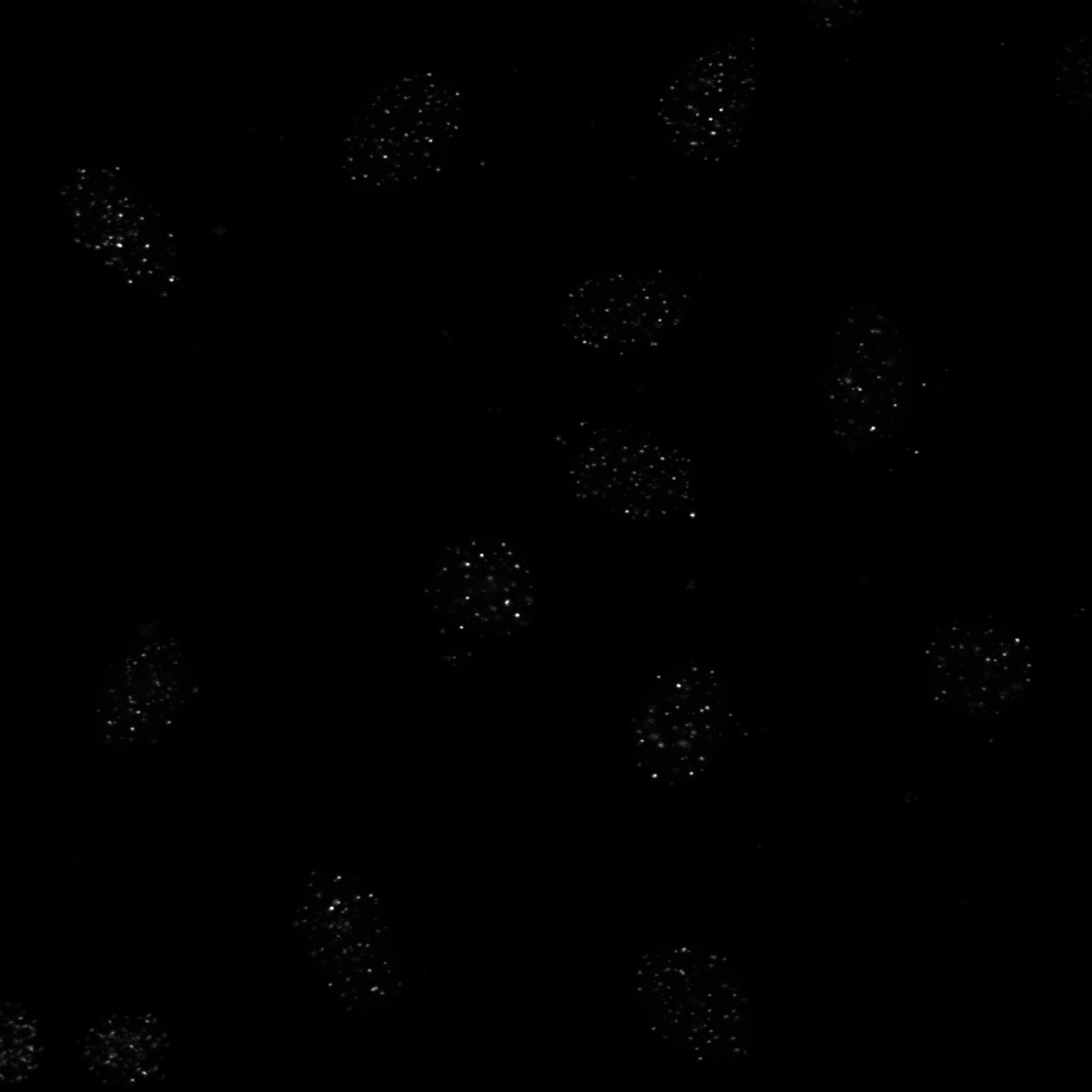

Supplement: Supplementary file 6 — Source data Fig. 6 [file 44318_2026_790_MOESM6_ESM.zip › Figure 6/Figure 6C_pRPA_TelC_U2OS_BLM_rescue/C4-U2OS_SLX4IP_KO_clone_2_siFANCM_siBLM_TelC.tif]

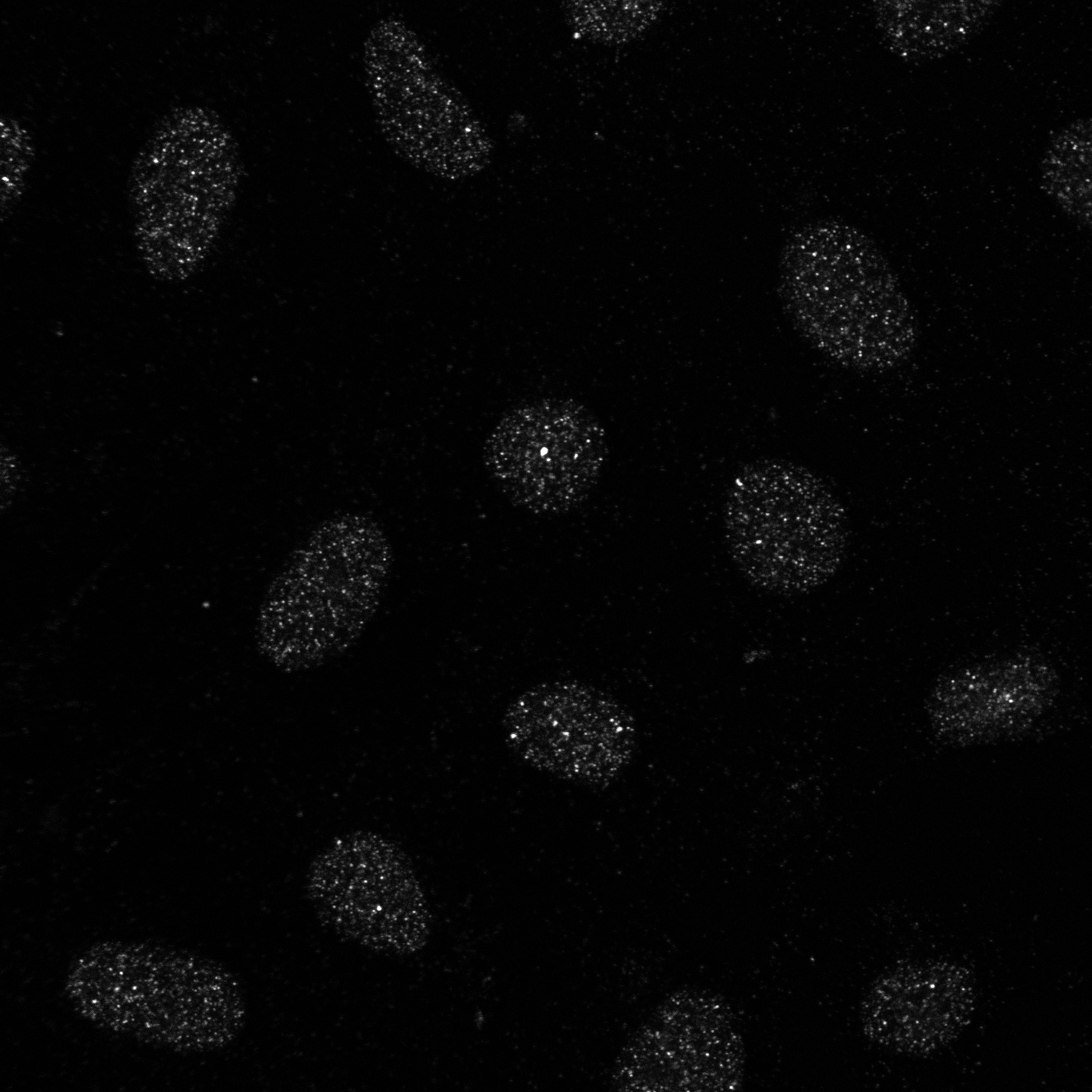

Supplement: Supplementary file 6 — Source data Fig. 6 [file 44318_2026_790_MOESM6_ESM.zip › Figure 6/Figure 6C_pRPA_TelC_U2OS_BLM_rescue/C3-U2OS_SLX4IP_KO_clone_1_siCTRL_pS33-RPA.tif]

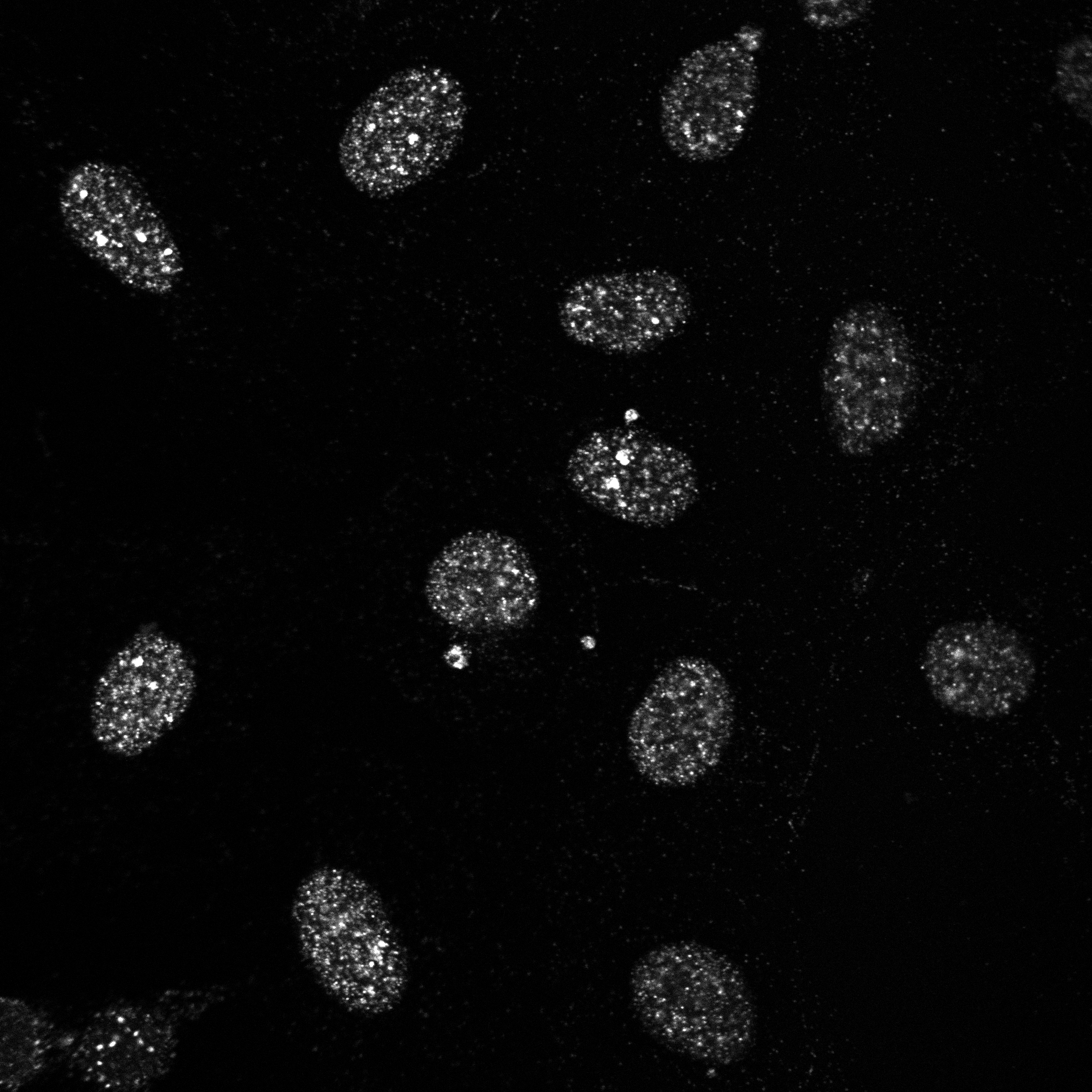

Supplement: Supplementary file 6 — Source data Fig. 6 [file 44318_2026_790_MOESM6_ESM.zip › Figure 6/Figure 6C_pRPA_TelC_U2OS_BLM_rescue/C3-U2OS_SLX4IP_KO_clone_2_siFANCM_siBLM_pS33-RPA.tif]

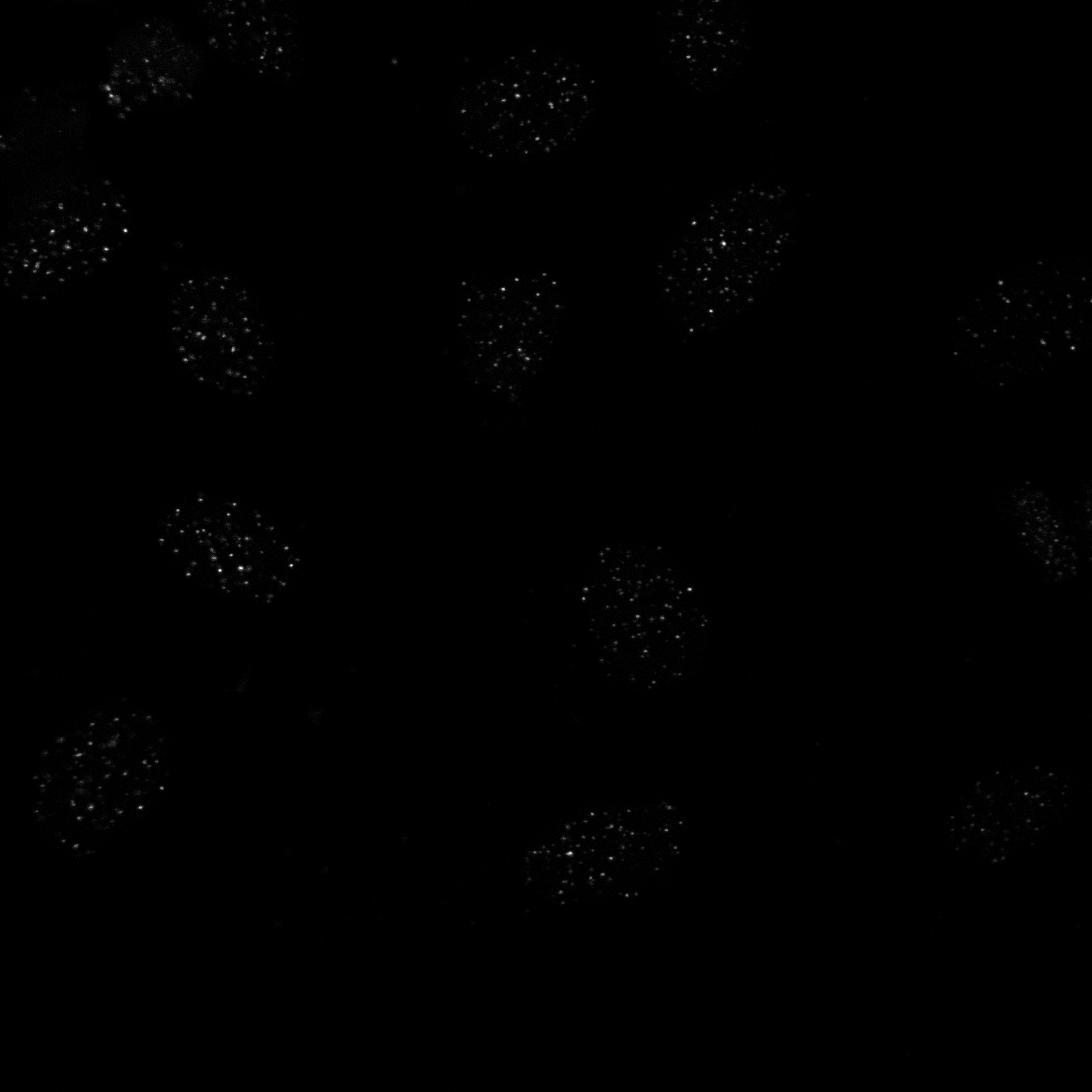

Supplement: Supplementary file 6 — Source data Fig. 6 [file 44318_2026_790_MOESM6_ESM.zip › Figure 6/Figure 6C_pRPA_TelC_U2OS_BLM_rescue/C4-U2OS_SLX4IP_KO_clone_1_siFANCM_siBLM_TelC.tif]

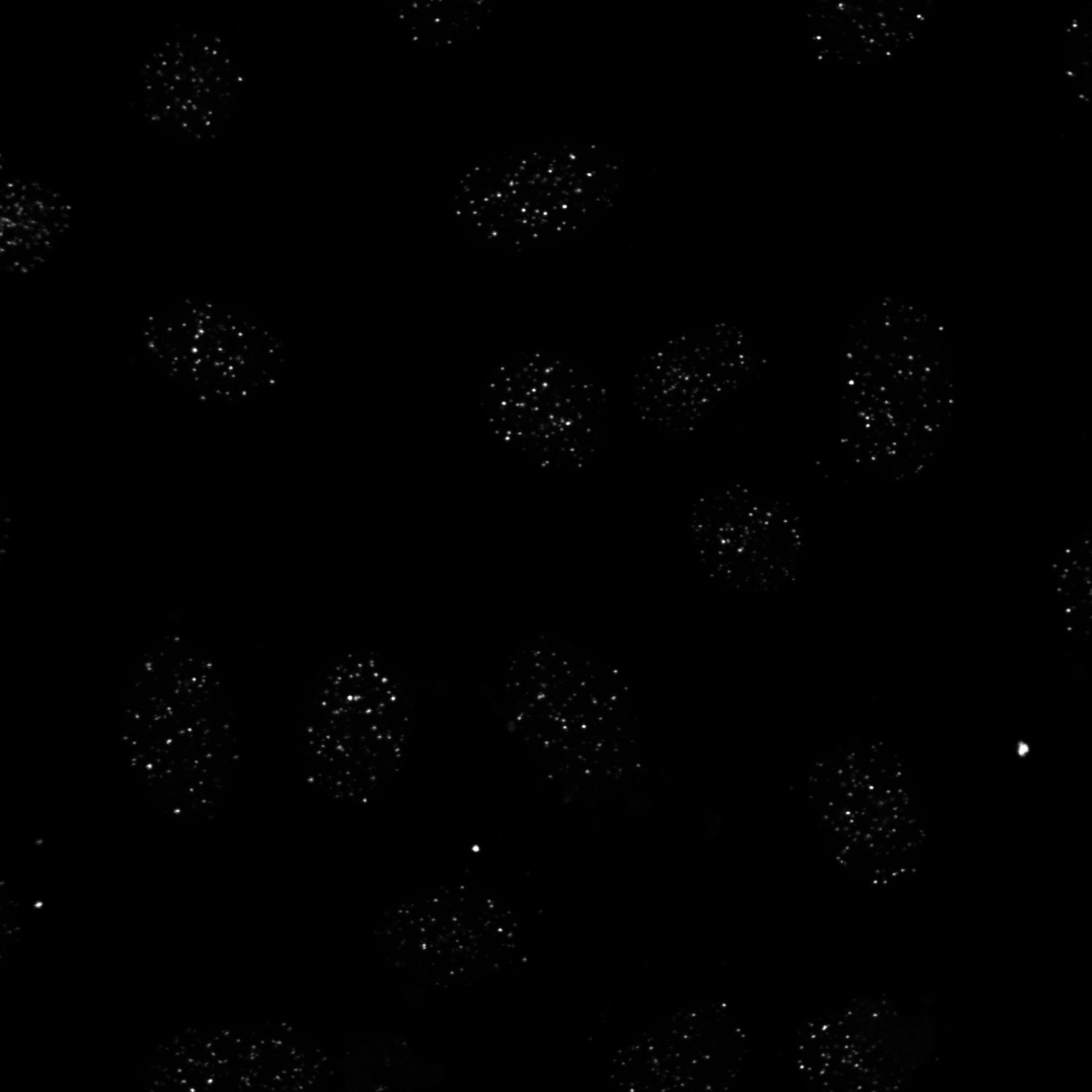

Supplement: Supplementary file 6 — Source data Fig. 6 [file 44318_2026_790_MOESM6_ESM.zip › Figure 6/Figure 6C_pRPA_TelC_U2OS_BLM_rescue/C4-U2OS_SLX4IP_KO_clone_1_siBLM_TelC.tif]

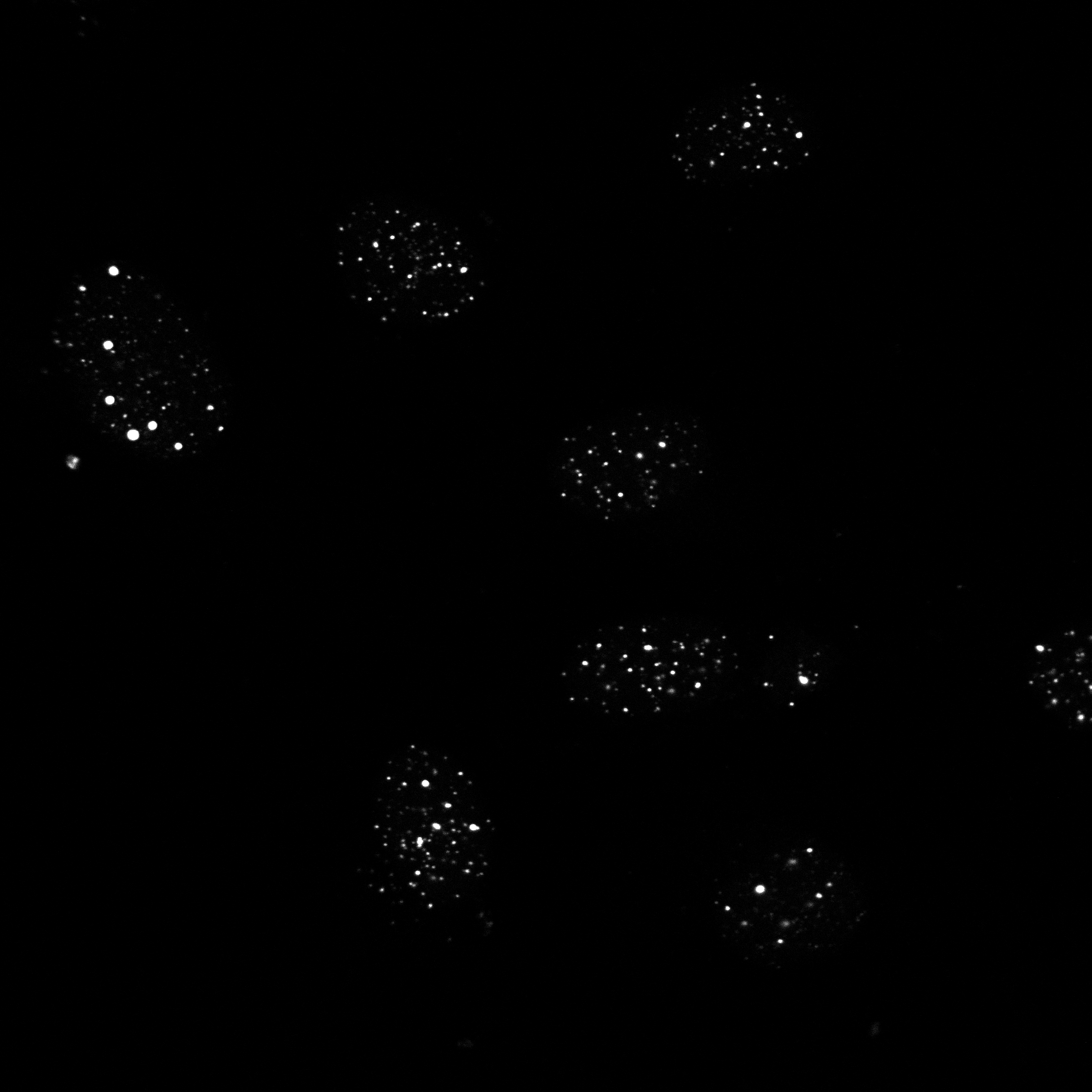

Supplement: Supplementary file 6 — Source data Fig. 6 [file 44318_2026_790_MOESM6_ESM.zip › Figure 6/Figure 6C_pRPA_TelC_U2OS_BLM_rescue/C4-U2OS_WT_siFANCM_TelC.tif]

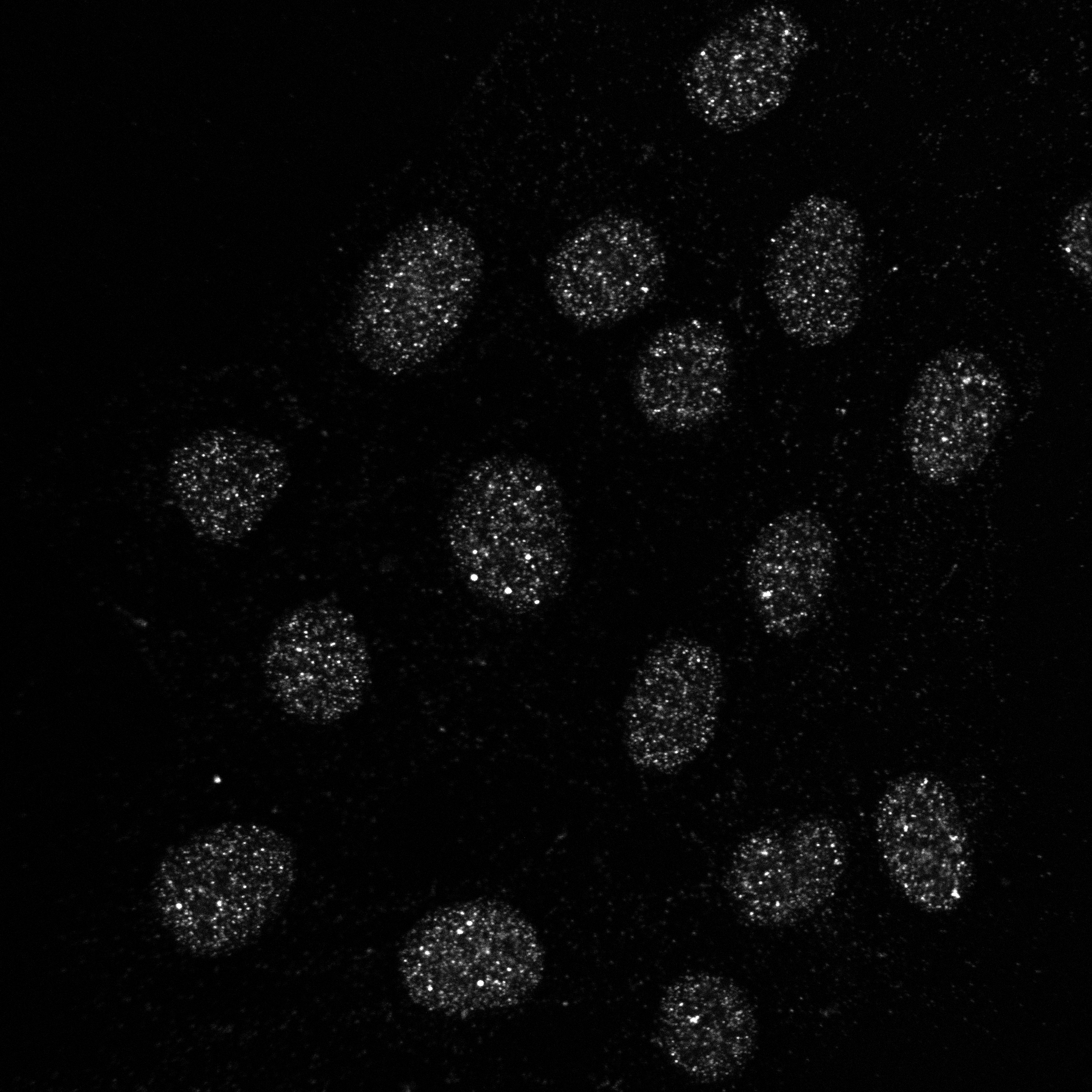

Supplement: Supplementary file 6 — Source data Fig. 6 [file 44318_2026_790_MOESM6_ESM.zip › Figure 6/Figure 6C_pRPA_TelC_U2OS_BLM_rescue/C3-U2OS_SLX4IP_KO_clone_2_siCTRL_pS33-RPA.tif]

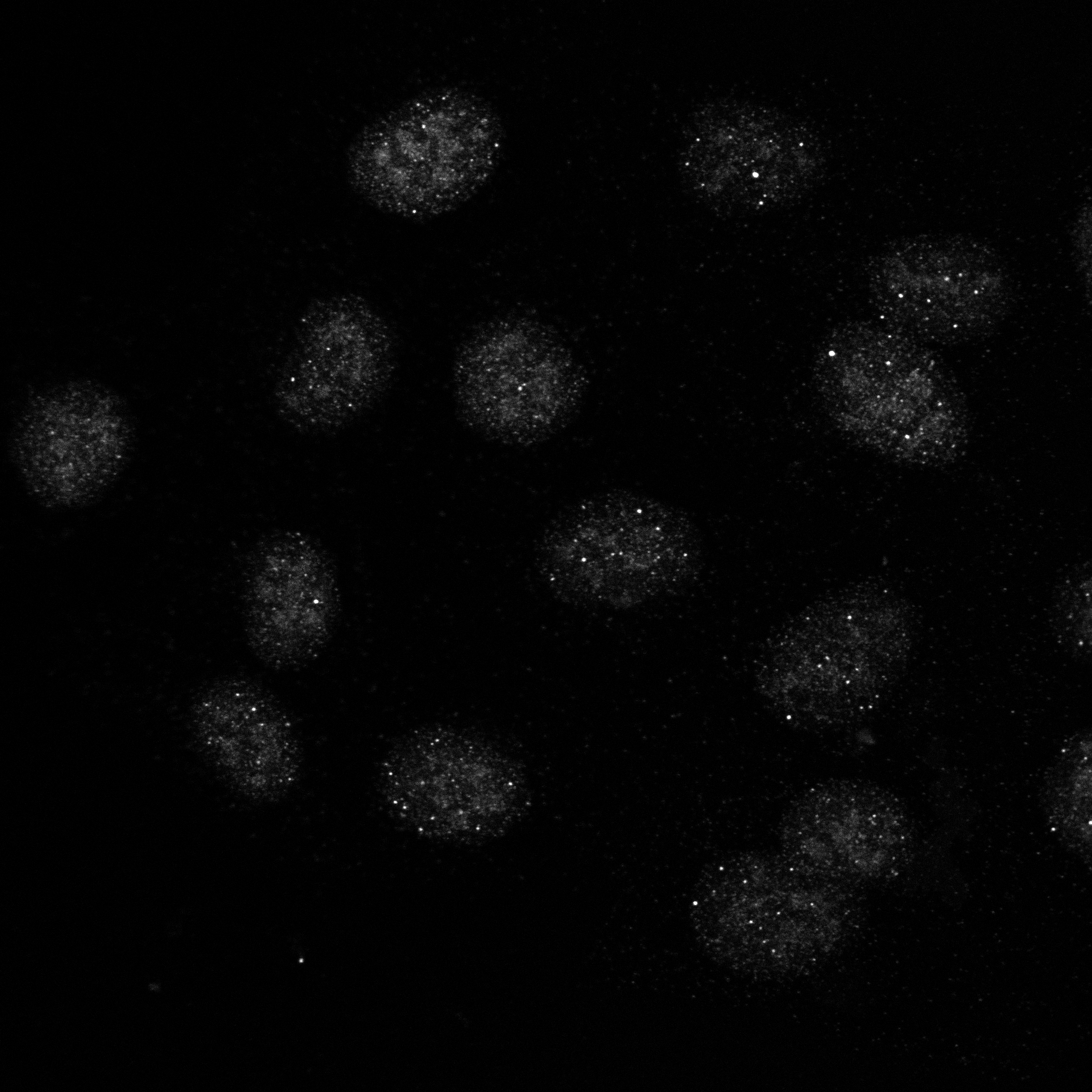

Supplement: Supplementary file 6 — Source data Fig. 6 [file 44318_2026_790_MOESM6_ESM.zip › Figure 6/Figure 6A_BLM_TelC_U2OS_siFANCM/C3-U2OS_SLX4IP_KO_clone_1_siCTRL_BLM.tif]

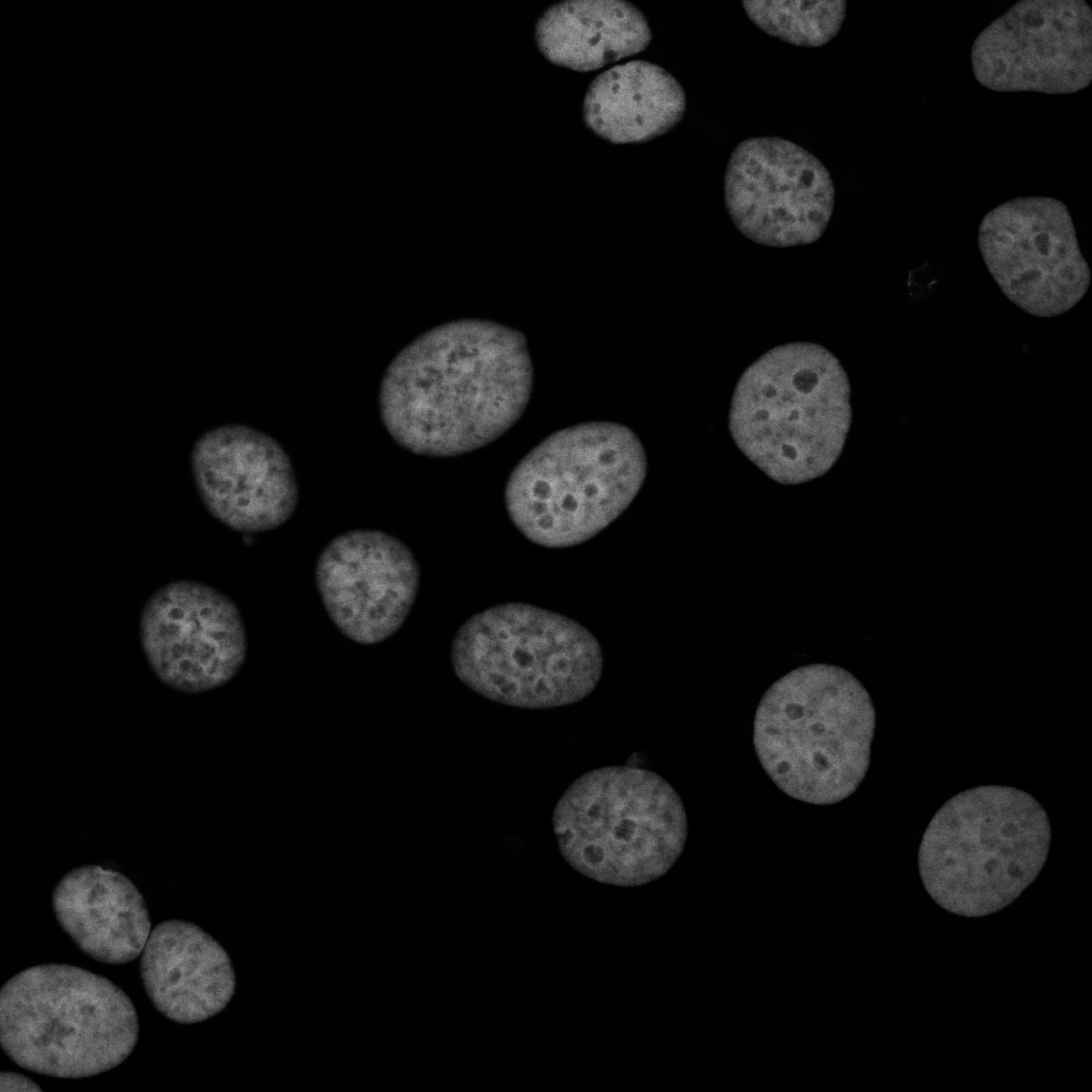

Supplement: Supplementary file 6 — Source data Fig. 6 [file 44318_2026_790_MOESM6_ESM.zip › Figure 6/Figure 6A_BLM_TelC_U2OS_siFANCM/C1-U2OS_SLX4IP_KO_clone_2_siCTRL_DAPI.tif]

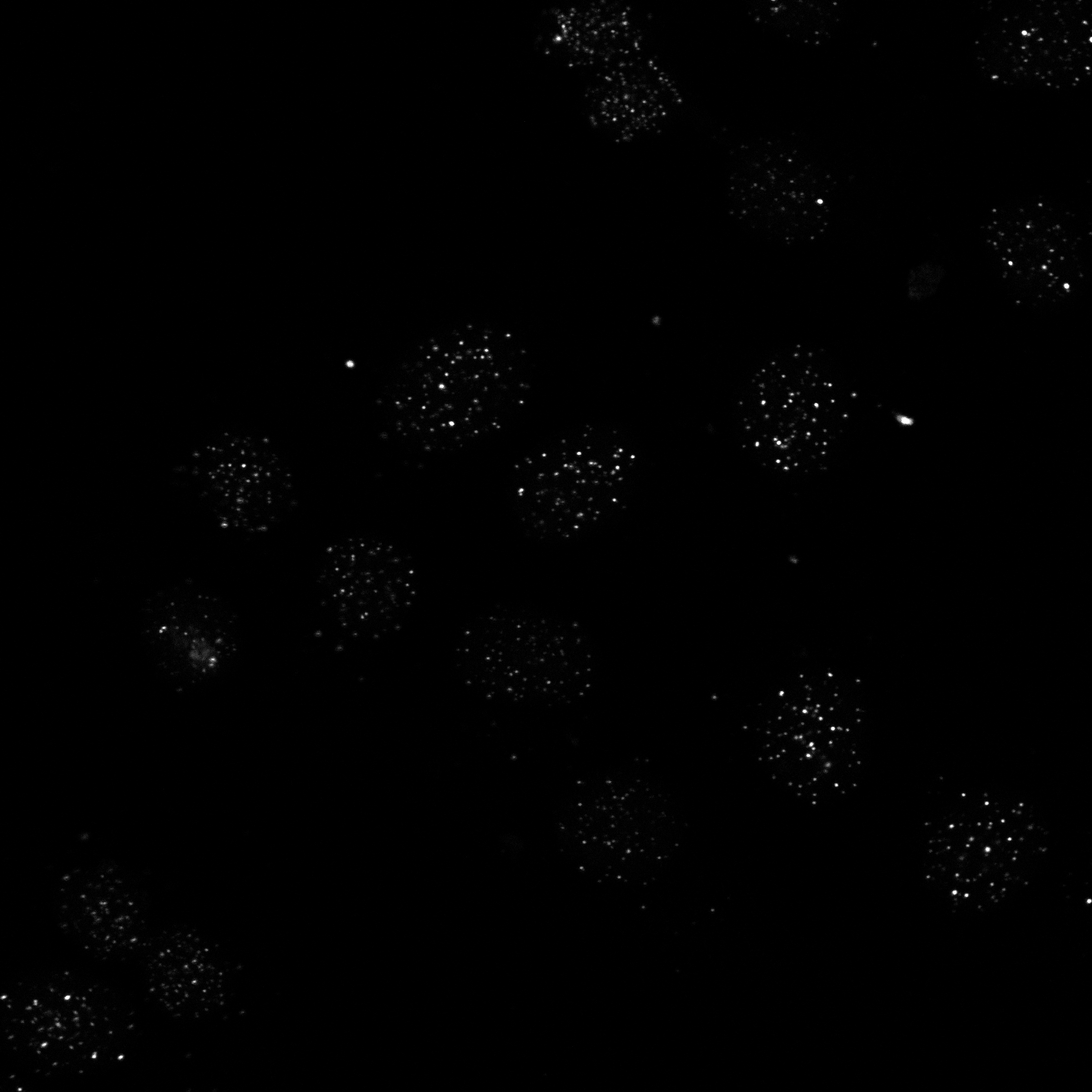

Supplement: Supplementary file 6 — Source data Fig. 6 [file 44318_2026_790_MOESM6_ESM.zip › Figure 6/Figure 6A_BLM_TelC_U2OS_siFANCM/C4-U2OS_SLX4IP_KO_clone_2_siCTRL_TelC.tif]

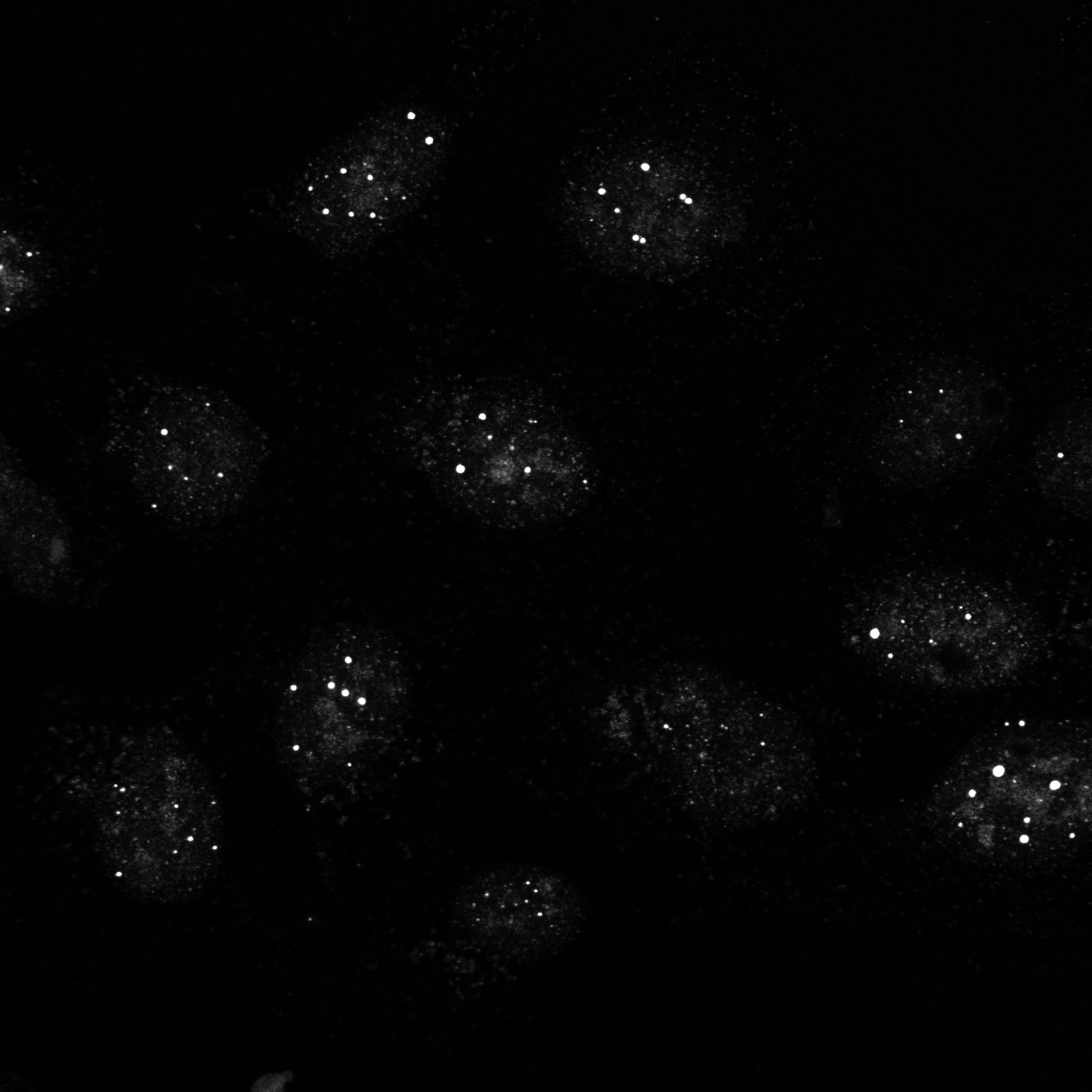

Supplement: Supplementary file 6 — Source data Fig. 6 [file 44318_2026_790_MOESM6_ESM.zip › Figure 6/Figure 6A_BLM_TelC_U2OS_siFANCM/C3-U2OS_WT_siFANCM_BLM.tif]

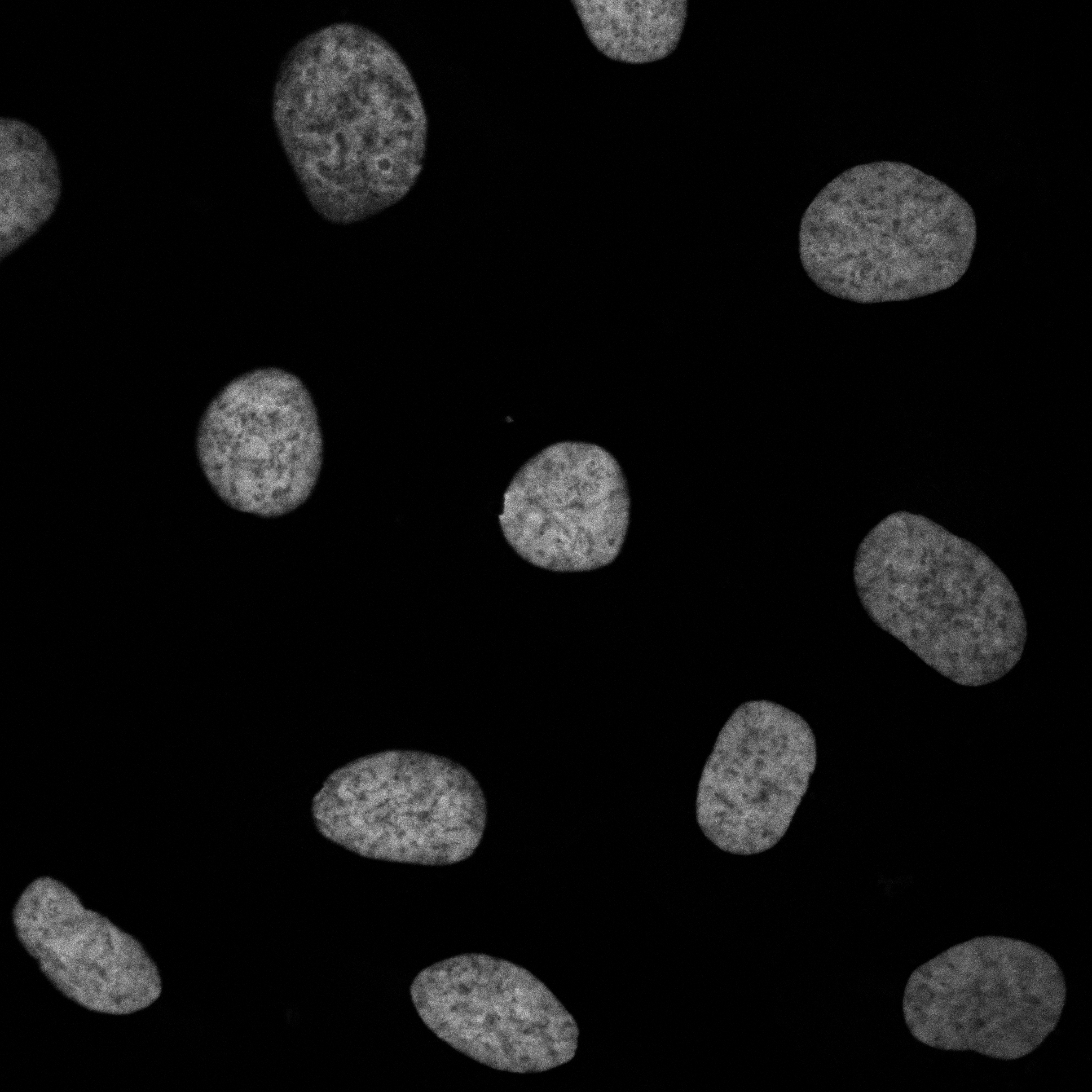

Supplement: Supplementary file 6 — Source data Fig. 6 [file 44318_2026_790_MOESM6_ESM.zip › Figure 6/Figure 6A_BLM_TelC_U2OS_siFANCM/C1-U2OS_SLX4IP_KO_clone_1_siFANCM_DAPI.tif]

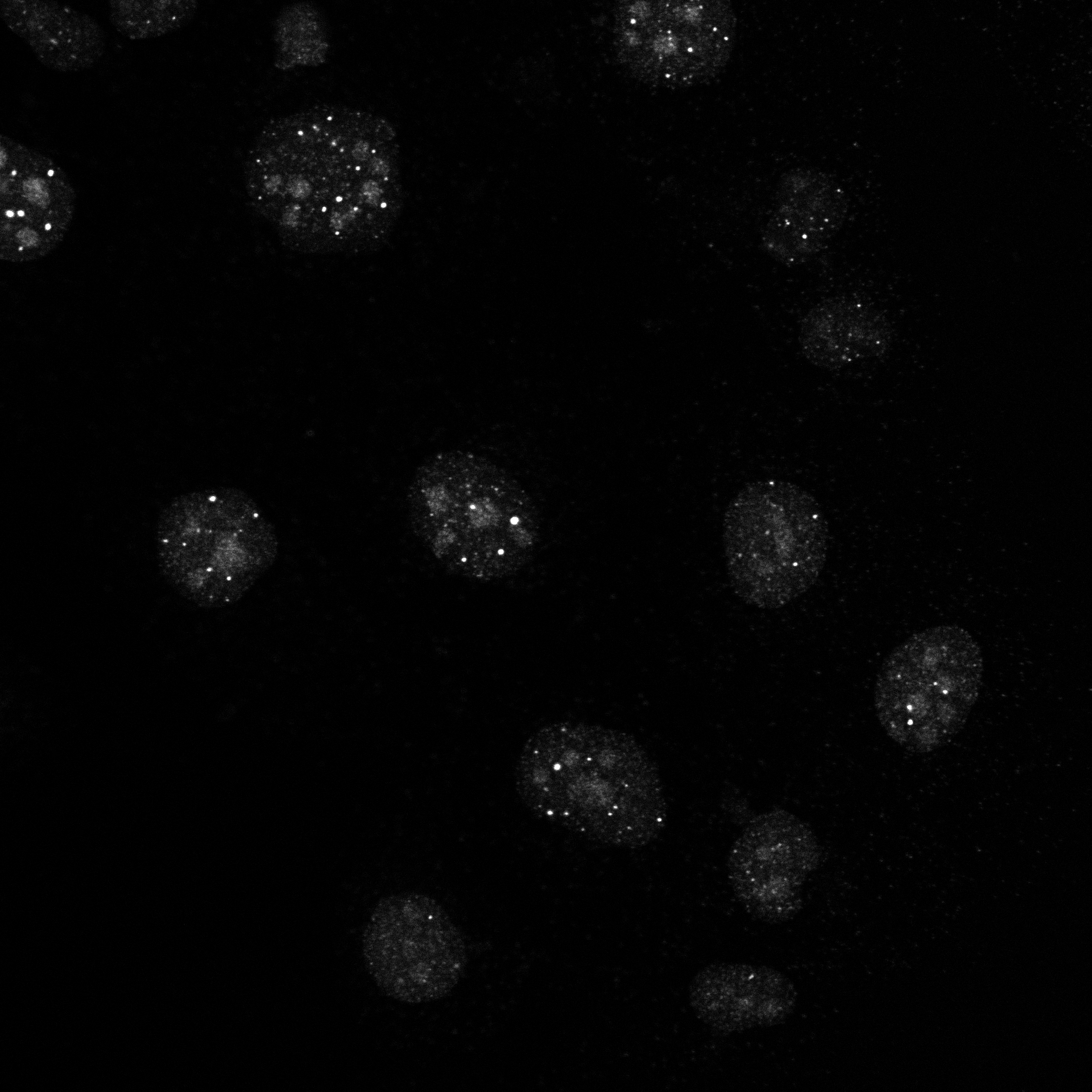

Supplement: Supplementary file 6 — Source data Fig. 6 [file 44318_2026_790_MOESM6_ESM.zip › Figure 6/Figure 6A_BLM_TelC_U2OS_siFANCM/C3-U2OS_SLX4IP_KO_clone_2_siFANCM_BLM.tif]

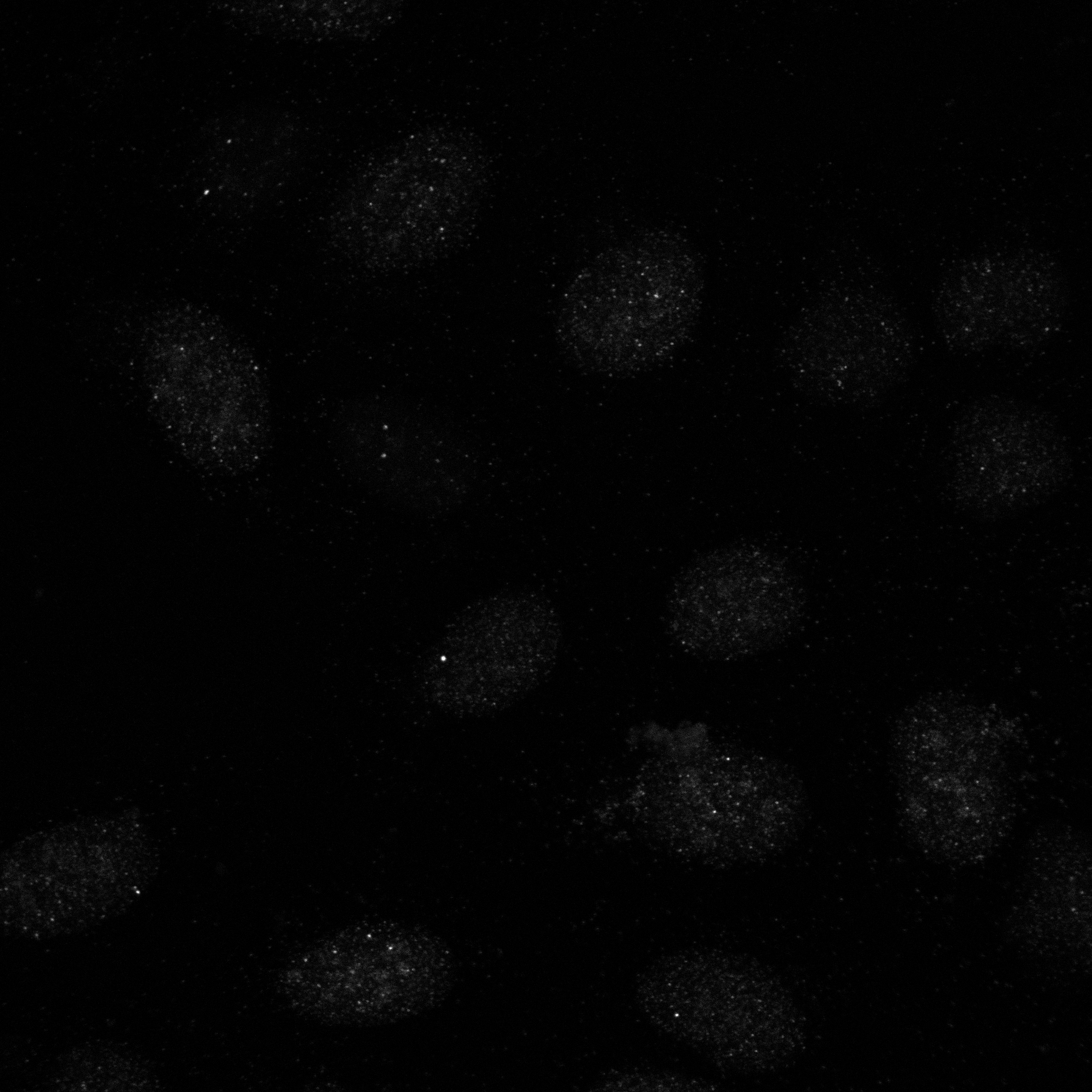

Supplement: Supplementary file 6 — Source data Fig. 6 [file 44318_2026_790_MOESM6_ESM.zip › Figure 6/Figure 6A_BLM_TelC_U2OS_siFANCM/C3-U2OS_WT_siCTRL_BLM.tif]

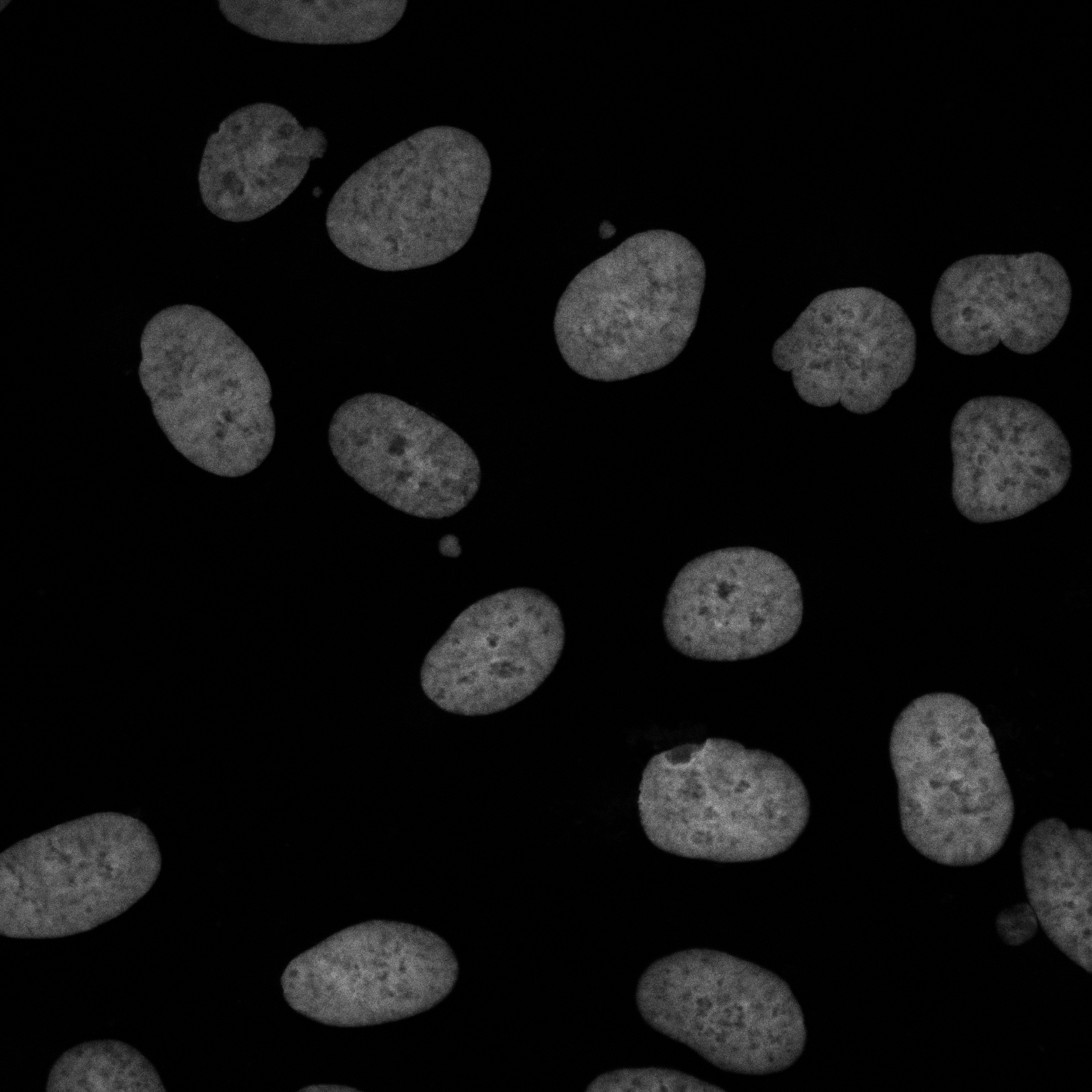

Supplement: Supplementary file 6 — Source data Fig. 6 [file 44318_2026_790_MOESM6_ESM.zip › Figure 6/Figure 6A_BLM_TelC_U2OS_siFANCM/C1-U2OS_WT_siCTRL_DAPI.tif]

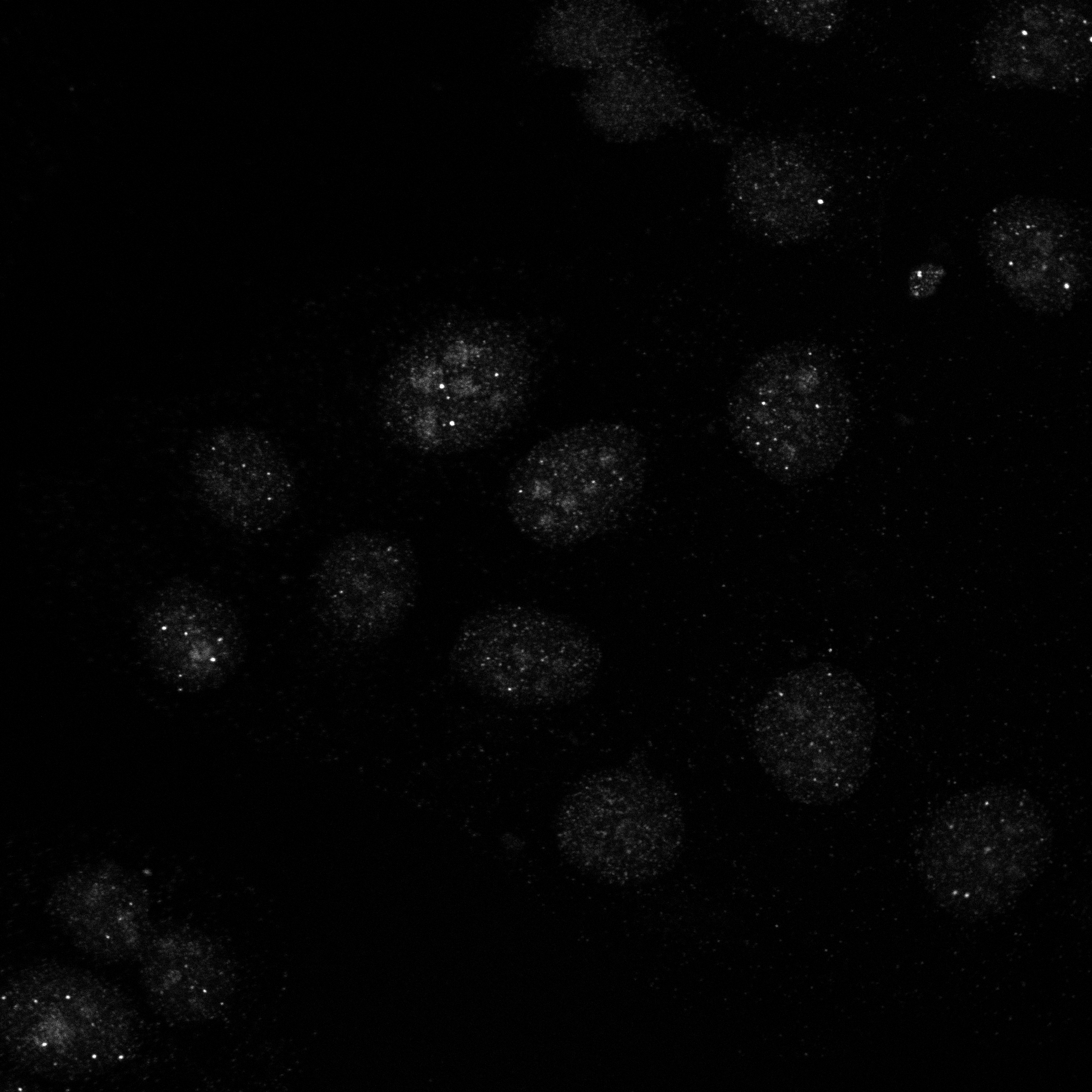

Supplement: Supplementary file 6 — Source data Fig. 6 [file 44318_2026_790_MOESM6_ESM.zip › Figure 6/Figure 6A_BLM_TelC_U2OS_siFANCM/C3-U2OS_SLX4IP_KO_clone_2_siCTRL_BLM.tif]

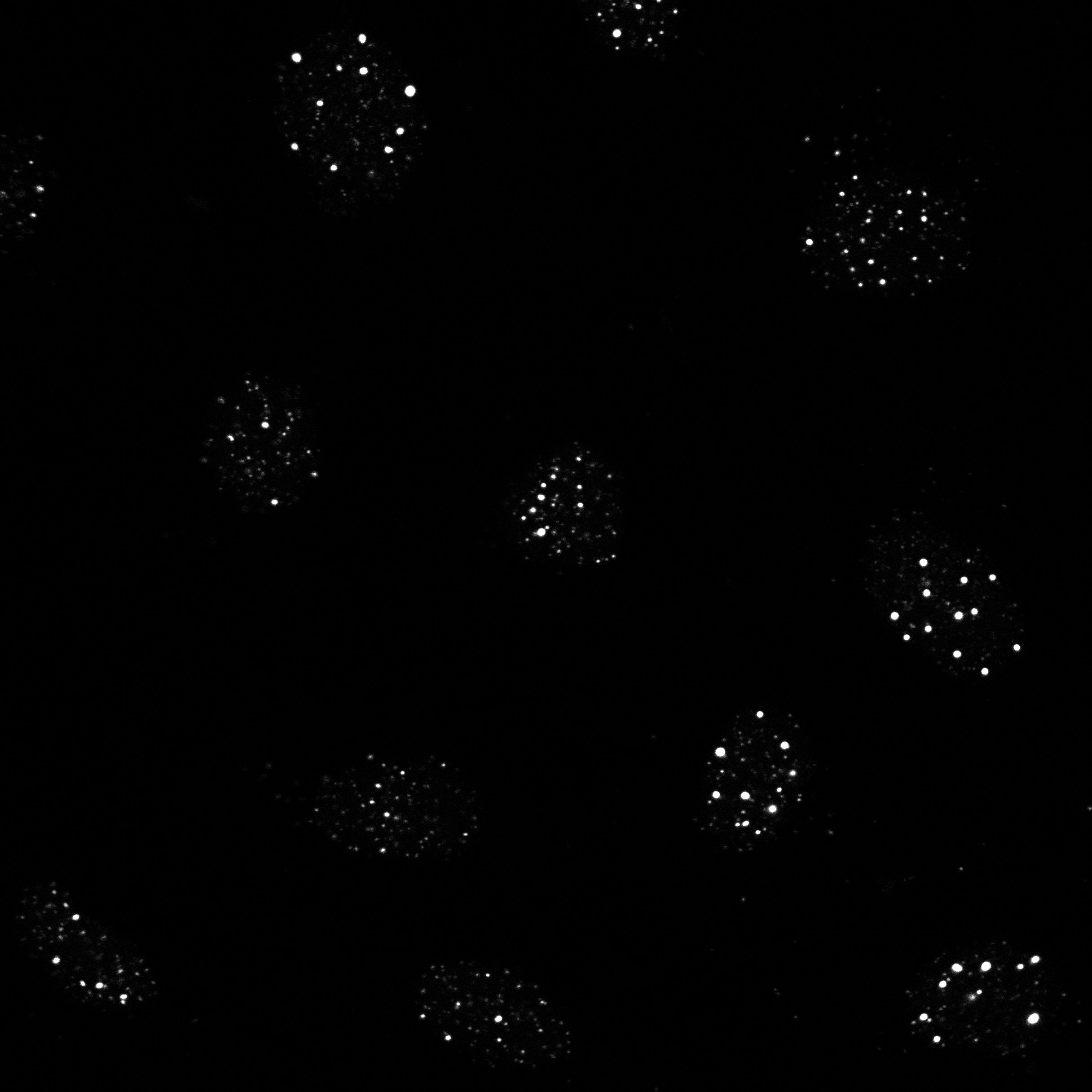

Supplement: Supplementary file 6 — Source data Fig. 6 [file 44318_2026_790_MOESM6_ESM.zip › Figure 6/Figure 6A_BLM_TelC_U2OS_siFANCM/C4-U2OS_SLX4IP_KO_clone_1_siFANCM_TelC.tif]

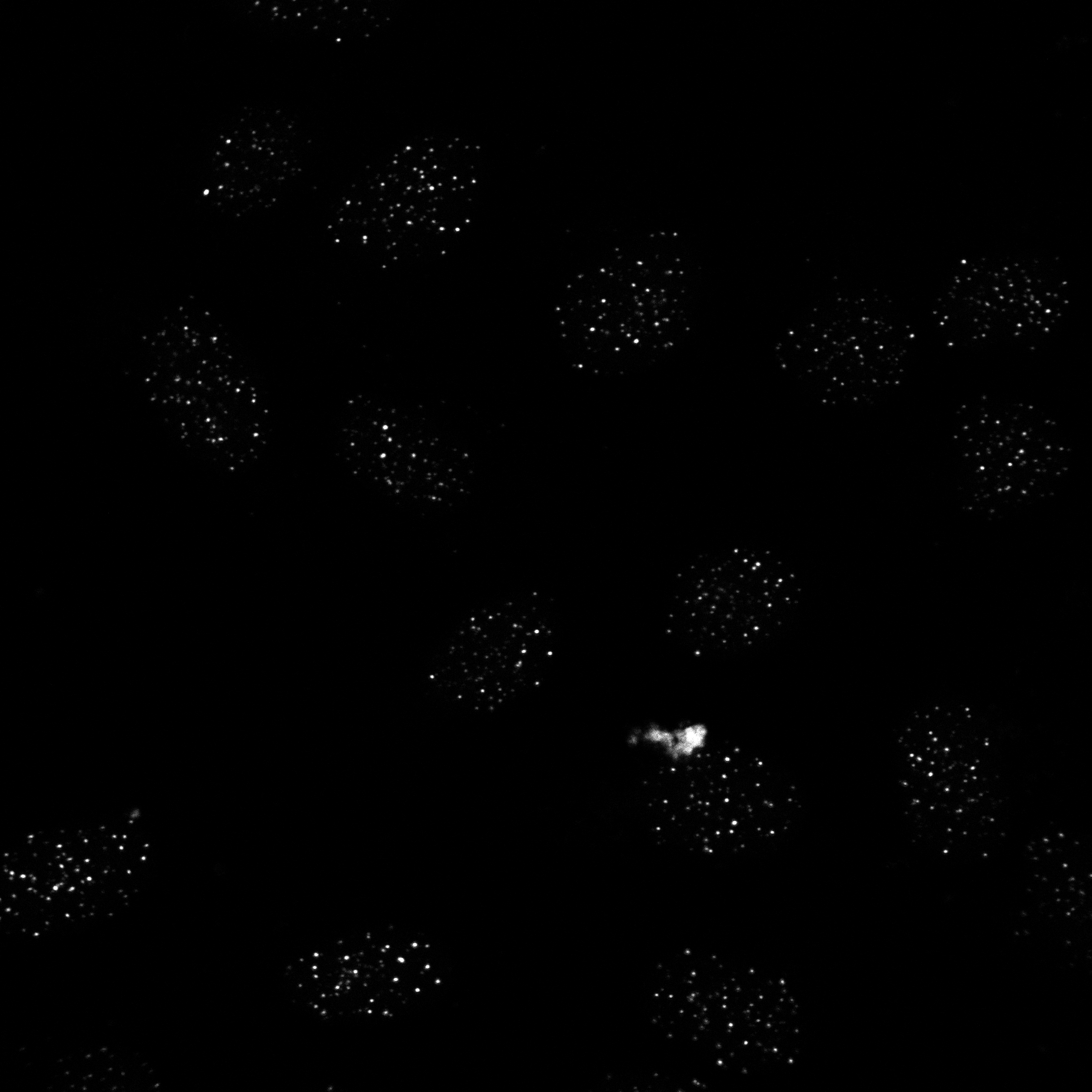

Supplement: Supplementary file 6 — Source data Fig. 6 [file 44318_2026_790_MOESM6_ESM.zip › Figure 6/Figure 6A_BLM_TelC_U2OS_siFANCM/C4-U2OS_WT_siCTRL_TelC.tif]

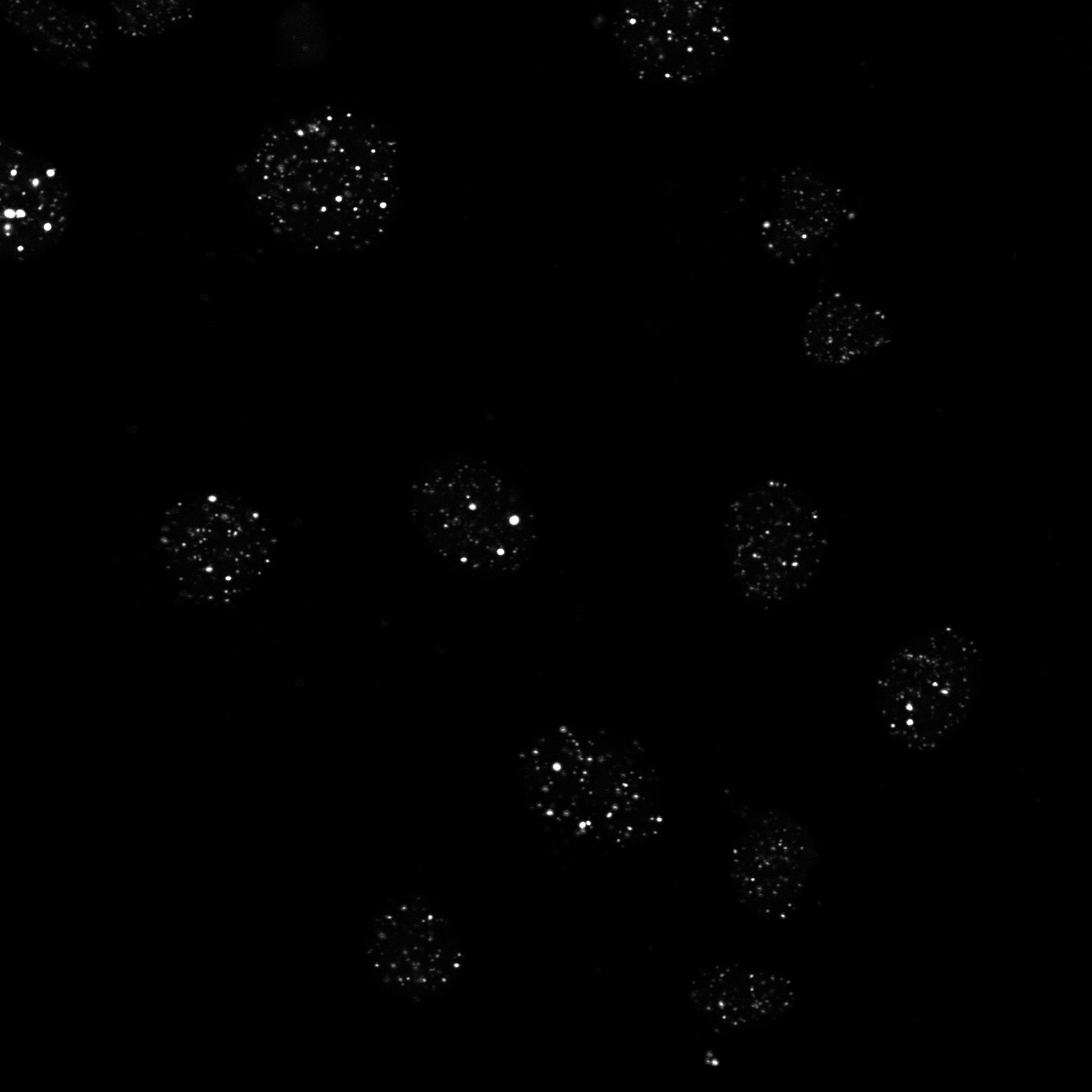

Supplement: Supplementary file 6 — Source data Fig. 6 [file 44318_2026_790_MOESM6_ESM.zip › Figure 6/Figure 6A_BLM_TelC_U2OS_siFANCM/C4-U2OS_SLX4IP_KO_clone_2_siFANCM_TelC.tif]

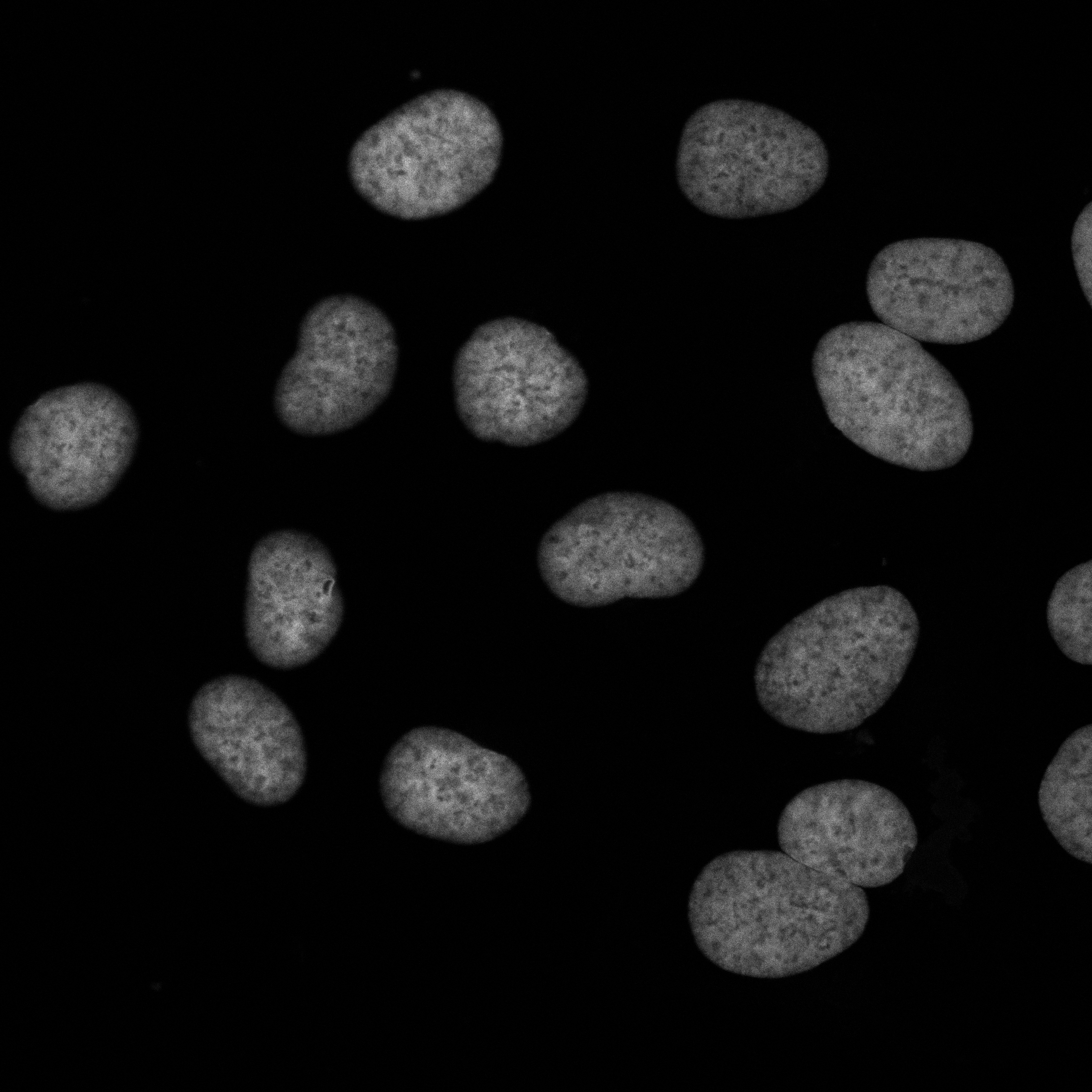

Supplement: Supplementary file 6 — Source data Fig. 6 [file 44318_2026_790_MOESM6_ESM.zip › Figure 6/Figure 6A_BLM_TelC_U2OS_siFANCM/C1-U2OS_SLX4IP_KO_clone_1_siCTRL_DAPI.tif]

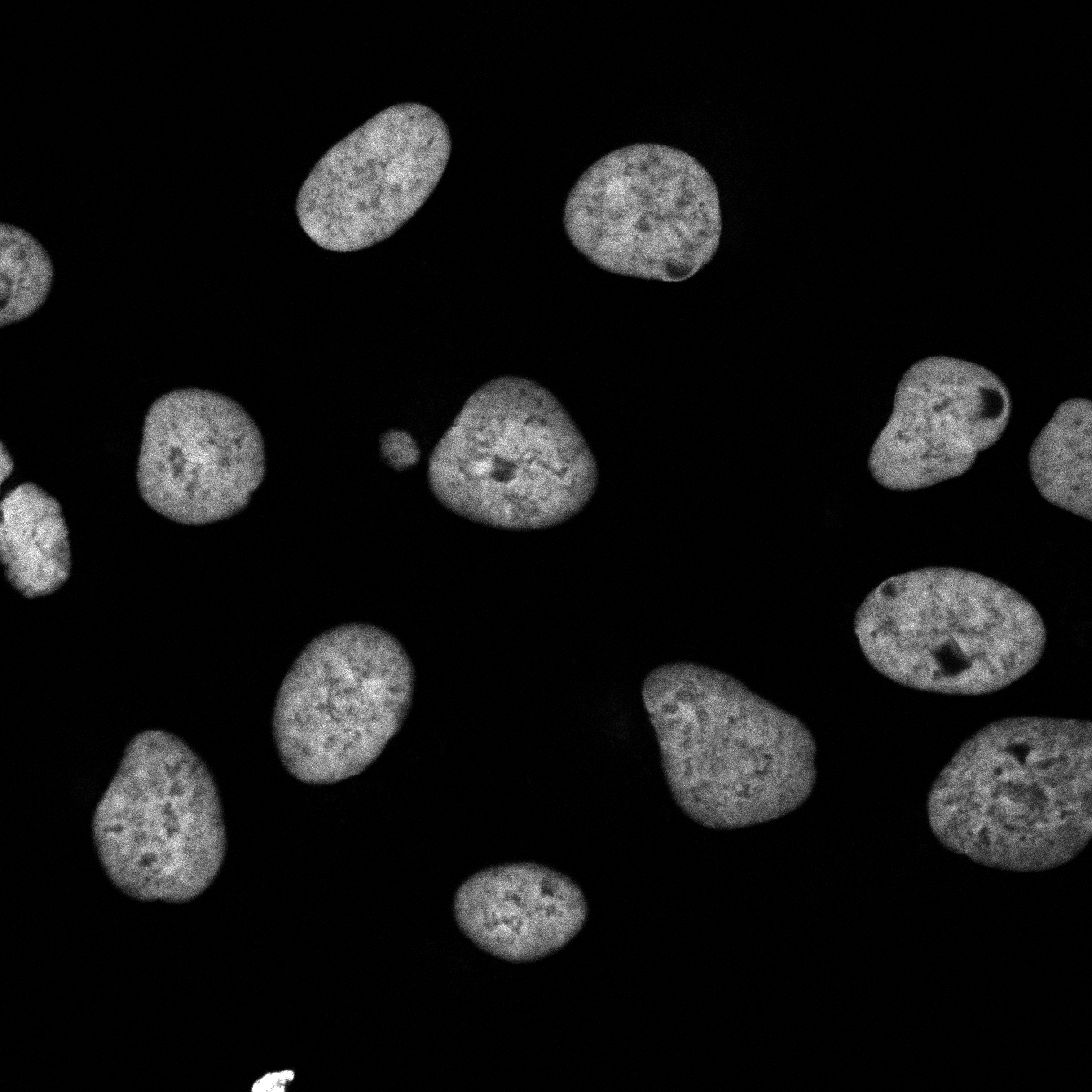

Supplement: Supplementary file 6 — Source data Fig. 6 [file 44318_2026_790_MOESM6_ESM.zip › Figure 6/Figure 6A_BLM_TelC_U2OS_siFANCM/C1-U2OS_WT_siFANCM_DAPI.tif]

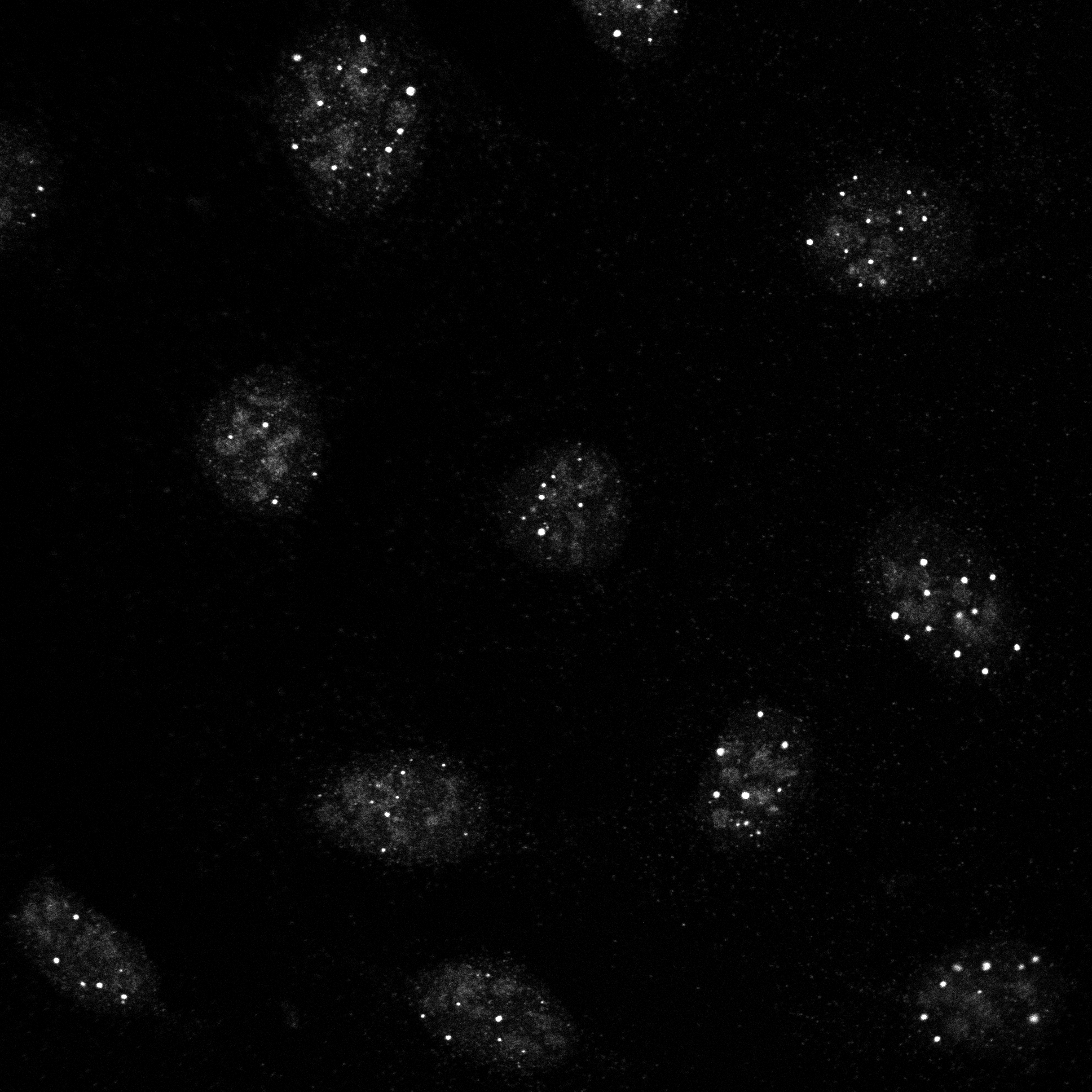

Supplement: Supplementary file 6 — Source data Fig. 6 [file 44318_2026_790_MOESM6_ESM.zip › Figure 6/Figure 6A_BLM_TelC_U2OS_siFANCM/C3-U2OS_SLX4IP_KO_clone_1_siFANCM_BLM.tif]

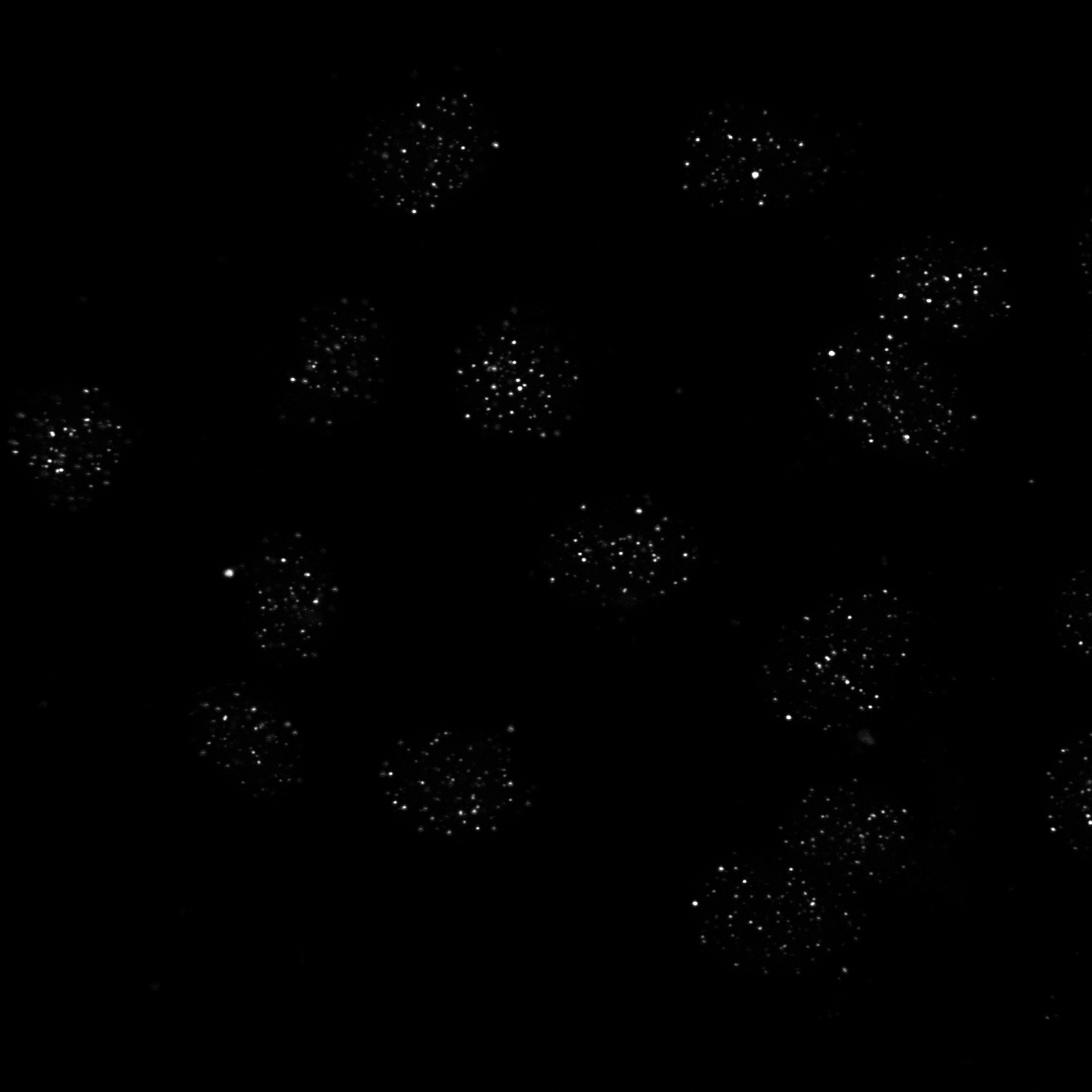

Supplement: Supplementary file 6 — Source data Fig. 6 [file 44318_2026_790_MOESM6_ESM.zip › Figure 6/Figure 6A_BLM_TelC_U2OS_siFANCM/C4-U2OS_SLX4IP_KO_clone_1_siCTRL_TelC.tif]

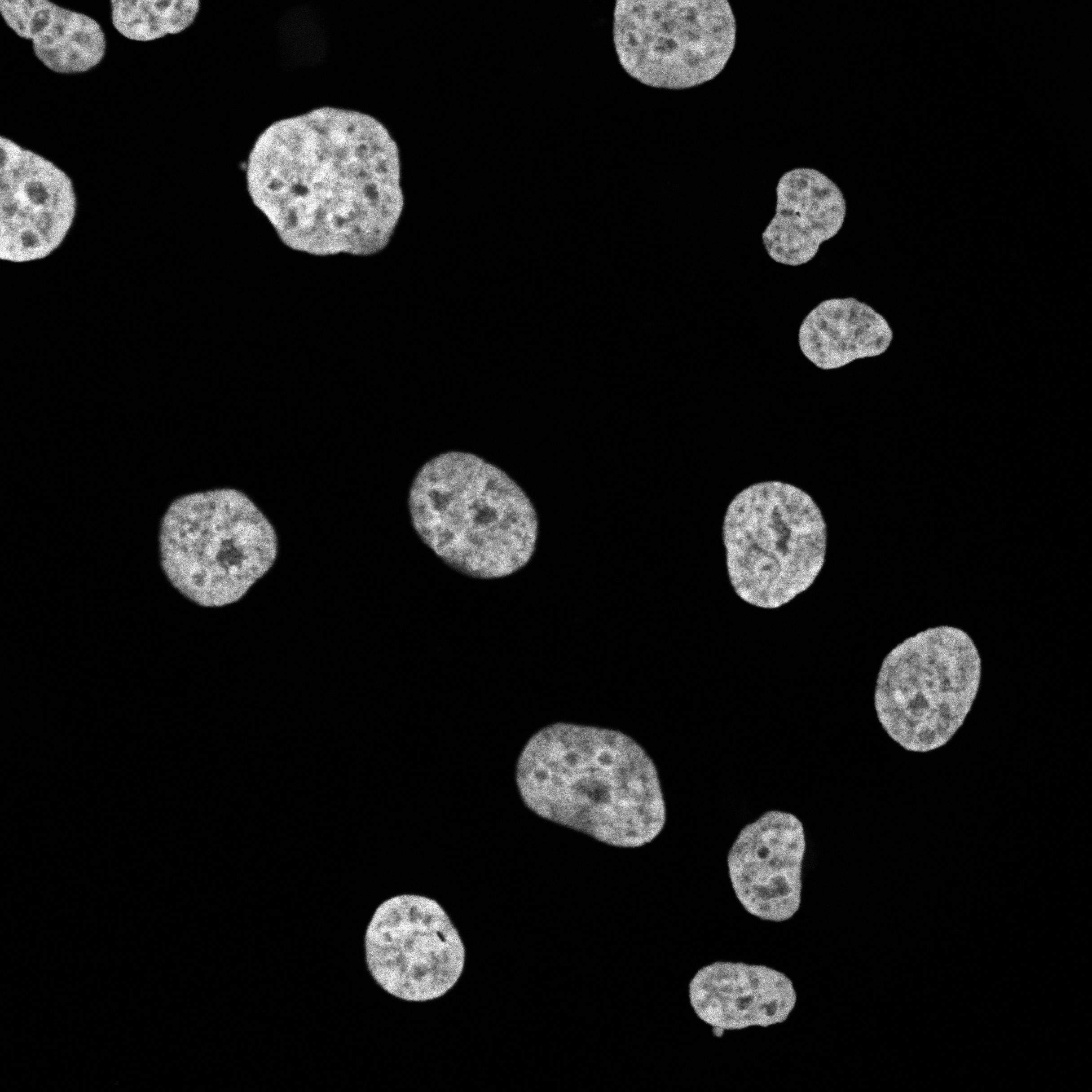

Supplement: Supplementary file 6 — Source data Fig. 6 [file 44318_2026_790_MOESM6_ESM.zip › Figure 6/Figure 6A_BLM_TelC_U2OS_siFANCM/C1-U2OS_SLX4IP_KO_clone_2_siFANCM_DAPI.tif]

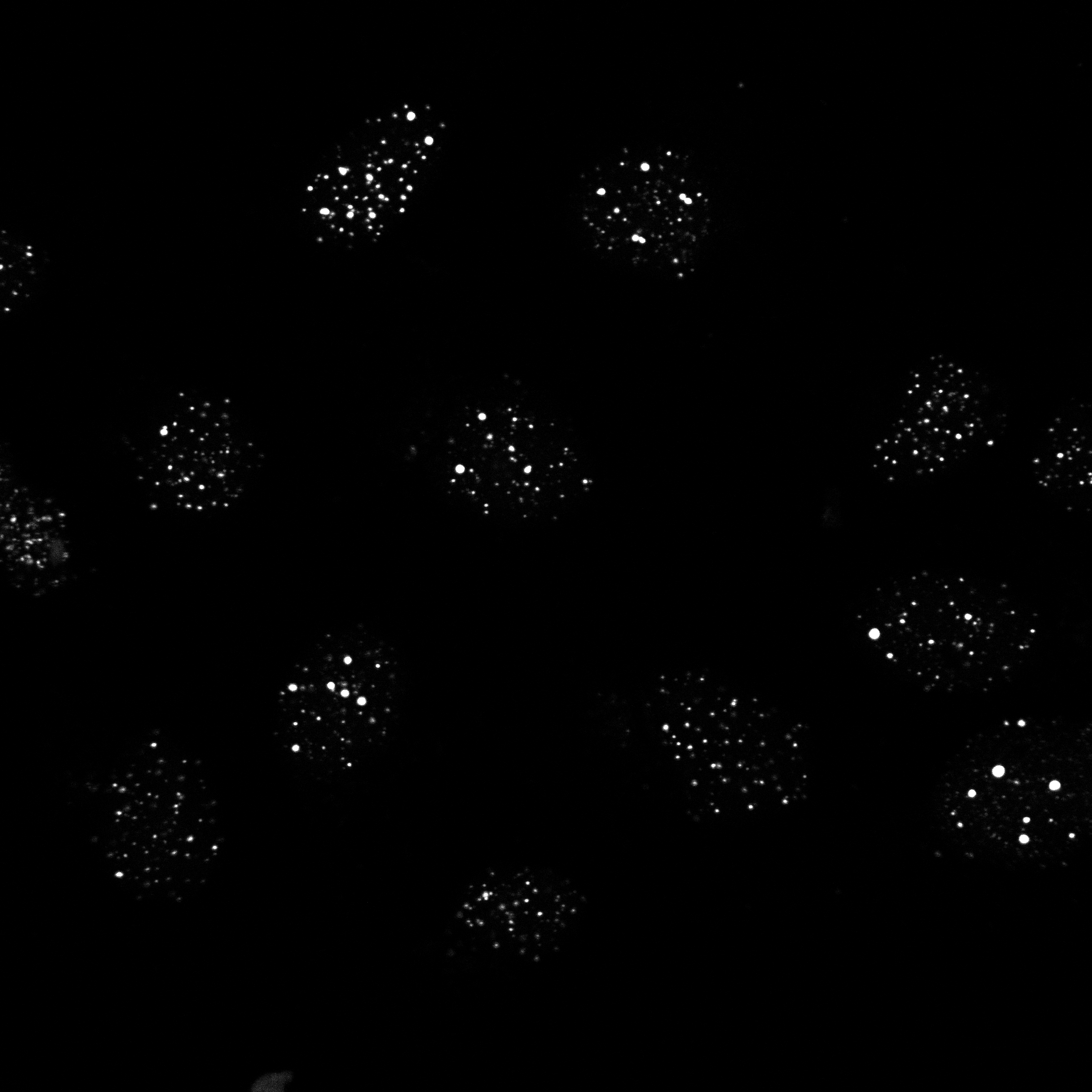

Supplement: Supplementary file 6 — Source data Fig. 6 [file 44318_2026_790_MOESM6_ESM.zip › Figure 6/Figure 6A_BLM_TelC_U2OS_siFANCM/C4-U2OS_WT_siFANCM_TelC.tif]
