# Supplementary material for: SLX4IP limits replication stress globally and at ALT telomeres
Source: EMBO J. 2026 May 7;45(12):4176–219. doi: 10.1038/s44318-026-00790-4 (PMC13269807; doi:10.1038/s44318-026-00790-4)
Supplement: Supplementary file 7 — Expanded View Figures [file 44318_2026_790_MOESM7_ESM.pdf]

Expanded View Figures

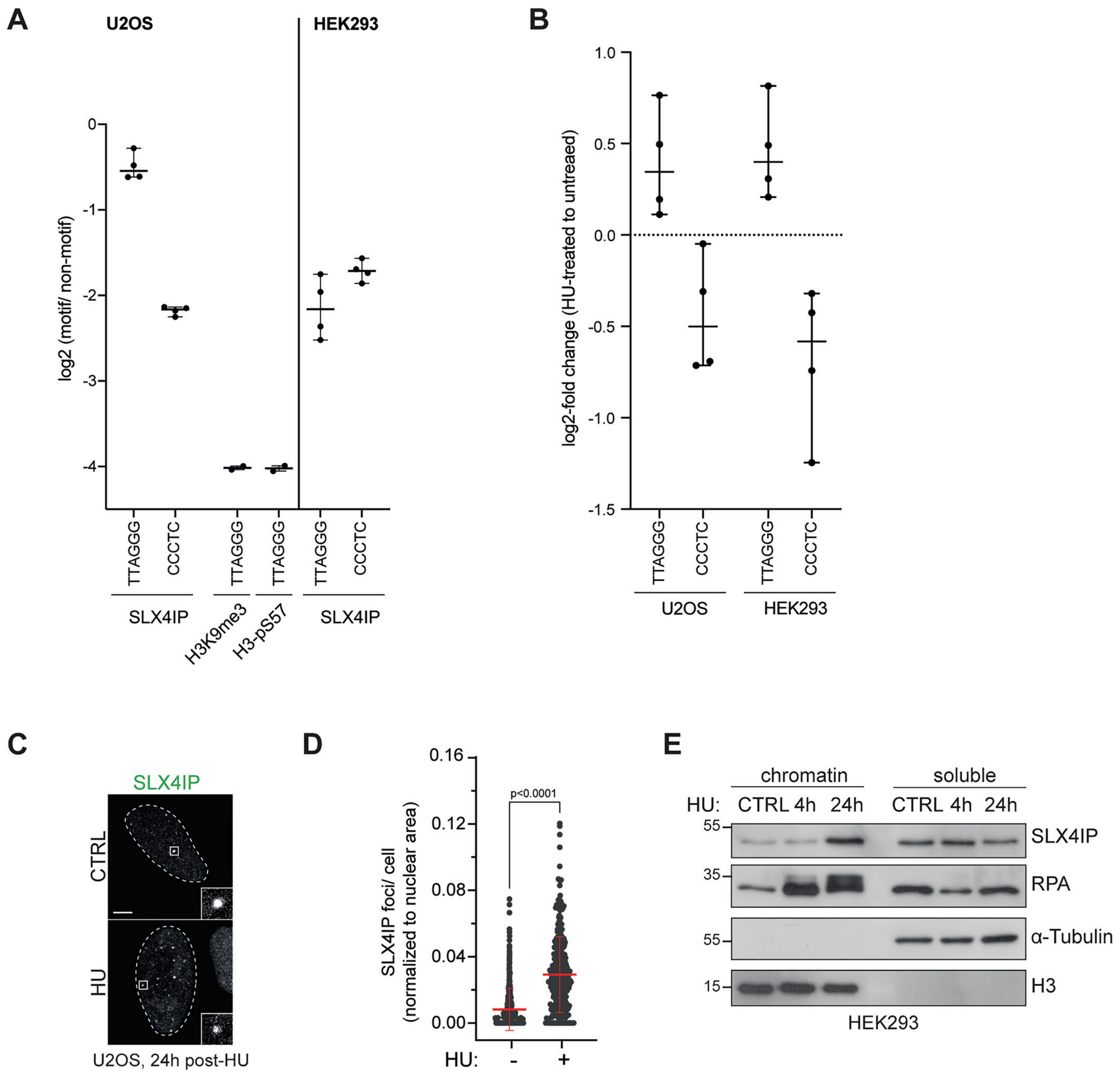

Figure EV1. Replication stress-induced increase in SLX4IP chromatin association.

(**A**) Regex analysis of TTAGGG and CCCTC motifs. TTAGGG and CCCTC repeats were defined as regex search on the sequences for each SLX4IP-enriched peak with fastaRegexFinder. The motif/non-motif ratio was calculated for each replicate. Replicate values were then statistically compared using a *t* test to determine whether the motif is significantly enriched. "non-motif" refers to all reads that do not contain the TTAGGG and CCCTC motif, respectively. Log2 enrichment was calculated from the SLX4IP CUT&Tag sequencing data (untreated) presented in Fig. 1. H3K9me3 and H3-pS57 sequencing data are from (Zhang et al, 2023; Parisis et al, 2023). Data are represented as median ± 95% confidence interval; 4 technical replicates (SLX4IP CUT&Tag). (**B**) Regex analysis of TTAGGG and CCCTC motifs to compare SLX4IP enrichment in untreated and hydroxyurea (HU)-treated cells. Log2 enrichment was calculated from the SLX4IP CUT&Tag sequencing data (untreated or treated with 4 mM hydroxyurea (HU) for 24 h) presented in Fig. 1. Data are represented as median ± 95% confidence interval; four technical replicates. (**C**) U2OS cells were either treated with 4 mM hydroxyurea (HU) or water as a control for 24 h. Cells were then immediately pre-extracted, fixed, and processed for SLX4IP immunofluorescence. Insets are 4X magnifications of the indicated fields. The dotted line represents DAPI (not shown). Scale bar represents 10 μm. (**D**) Quantification of (**C**). The number of SLX4IP foci in each cell was quantified and normalized to nuclear area to account for variations in nuclear size across cells. At least 100 cells per condition and experiment were counted. Data are represented as mean ± SD; $n = 3$, biological replicates; Student's *t* test. The exact *P* value is shown in the figure; $P < 0.05$ was considered statistically significant. (**E**) HEK293 cells were either treated with 4 mM hydroxyurea (HU) or water as a control for 4 h or 24 h. A chromatin fractionation was performed, and the soluble and chromatin fractions were separated by SDS-PAGE and analyzed for SLX4IP and RPA32 levels by immunoblotting. α-Tubulin and H3 were used as loading controls. The numbers on the left denote the molecular weight in kDa.

 © The Author(s)

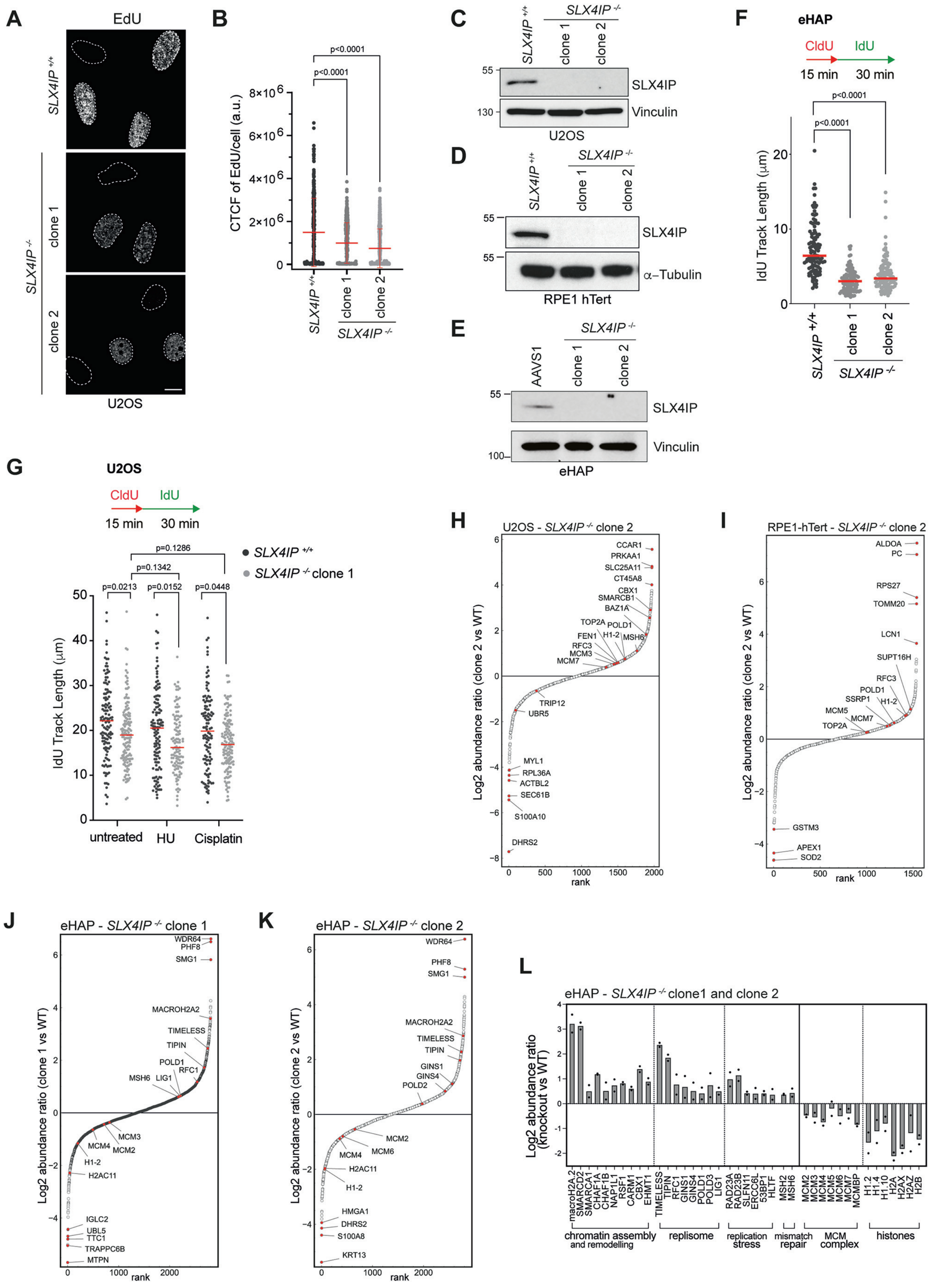
A
EdU
SLX4IP +/+
SLX4IP -/-
clone 1
clone 2
U2OS
B
CTCF of EdU/cell (a.u.)
p<0.0001
p<0.0001
C
SLX4IP
Vinculin
U2OS
D
SLX4IP
α-Tubulin
RPE1 hTert
E
AAVS1
SLX4IP
Vinculin
eHAP
F
eHAP
CldU
IdU
15 min
30 min
IdU Track Length (µm)
G
U2OS
p=0.1286
p=0.1342
p=0.0213
p=0.0152
p=0.0448
SLX4IP +/+
SLX4IP -/- clone 1
untreated
HU
Cisplatin
H
U2OS - SLX4IP -/- clone 2
Log2 abundance ratio (clone 2 vs WT)
rank
I
RPE1-hTert - SLX4IP -/- clone 2
J
eHAP - SLX4IP -/- clone 1
Log2 abundance ratio (clone 1 vs WT)
K
eHAP - SLX4IP -/- clone 2
L
eHAP - SLX4IP -/- clone1 and clone 2
Log2 abundance ratio (Knockout vs WT)
chromatin assembly and remodelling
replisome
replication stress
mismatch repair
MCM complex
histones

Figure EV2. Replication stress analysis of SLX4IP-defi cient cells.

(**A**) U2OS cells were subjected to a 30 min EdU pulse before fixation, followed by staining of EdU via a Click-IT reaction. The dotted line represents DAPI (not shown). Scale bar represents 10 µm. (**B**) Quantification of (**A**). The EdU signal was quantified as corrected total nuclear intensity per cell. At least 100 cells per condition and experiment were counted. Data are represented as mean ± SD; $n = 3$, biological replicates; one-way ANOVA with Dunnett's multiple comparisons post-test. Exact *P* values are shown in the figure; $P < 0.05$ was considered statistically significant. (**C**) Whole-cell lysates of U2OS cells were separated by SDS-PAGE and analyzed for SLX4IP levels by immunoblotting. Vinculin was used as a loading control. The numbers on the left denote the molecular weight in kDa. (**D**) Whole-cell lysates of RPE1-hTert cells were separated by SDS-PAGE and analyzed for SLX4IP levels by immunoblotting. α -Tubulin was used as a loading control. The numbers on the left denote the molecular weight in kDa. (**E**) Whole-cell lysates of eHAP cells were separated by SDS-PAGE and analyzed for SLX4IP levels by immunoblotting. Vinculin was used as a loading control. The numbers on the left denote the molecular weight in kDa. (**F**) eHAP cells were labeled with CldU and IdU before DNA combing. Individual IdU fiber lengths are plotted. Median is indicated; $n = 3$, biological replicates; ANOVA with Dunn's multiple comparisons post-test. Exact *P* values are shown in the figure; $P < 0.05$ was considered statistically significant. (**G**) U2OS cells were labeled with CldU and IdU for the indicated times before DNA combing. Samples were either with 50 µM hydroxyurea (HU; 30 min, during IdU pulse) or 50 µM Cisplatin (30 min, during IdU pulse). Individual IdU fiber lengths are plotted. Median is indicated; $n = 3$, biological replicates; one-way ANOVA with Dunn's multiple comparisons post-test. Exact *P* values are shown in the figure; $P < 0.05$ was considered statistically significant. (**H**) U2OS were subjected to a 20 min EdU pulse to label newly synthesized DNA, followed by iPOND coupled to mass spectrometry. The graph shows the log_2 ratio intensity of proteins accumulated at replication forks in U2OS *SLX4IP*$^{-/-}$ clone 2 compared to *SLX4IP*$^{+/+}$ cells. Related to Fig. 2C,E. (**I**) RPE1-hTert were subjected to a 20 min EdU pulse to label newly synthesized DNA, followed by iPOND coupled to mass spectrometry. The graph shows the log_2 ratio intensity of proteins accumulated at replication forks in RPE1-hTert *SLX4IP*$^{-/-}$ clone 2 compared to *SLX4IP*$^{+/+}$ cells. Related to Fig. 2D,F. (**J**) eHAP were subjected to a 20 min EdU pulse to label newly synthesized DNA, followed by iPOND coupled to mass spectrometry. The graph shows the log_2 ratio intensity of proteins accumulated at replication forks in *SLX4IP*$^{-/-}$ clone 1 compared to *SLX4IP*$^{+/+}$ cells. (**K**) eHAP were subjected to a 20 min EdU pulse to label newly synthesized DNA, followed by iPOND coupled to mass spectrometry. The graph shows the log_2 ratio intensity of proteins accumulated at replication forks in *SLX4IP*$^{-/-}$ clone 2 compared to *SLX4IP*$^{+/+}$ cells. (**L**) Abundance ratios of proteins at nascent DNA for selected proteins across U2OS *SLX4IP*$^{-/-}$ clone 1 and clone 2. Each data point corresponds to the abundance ratio in one clone; the mean is indicated.

© The Author(s)

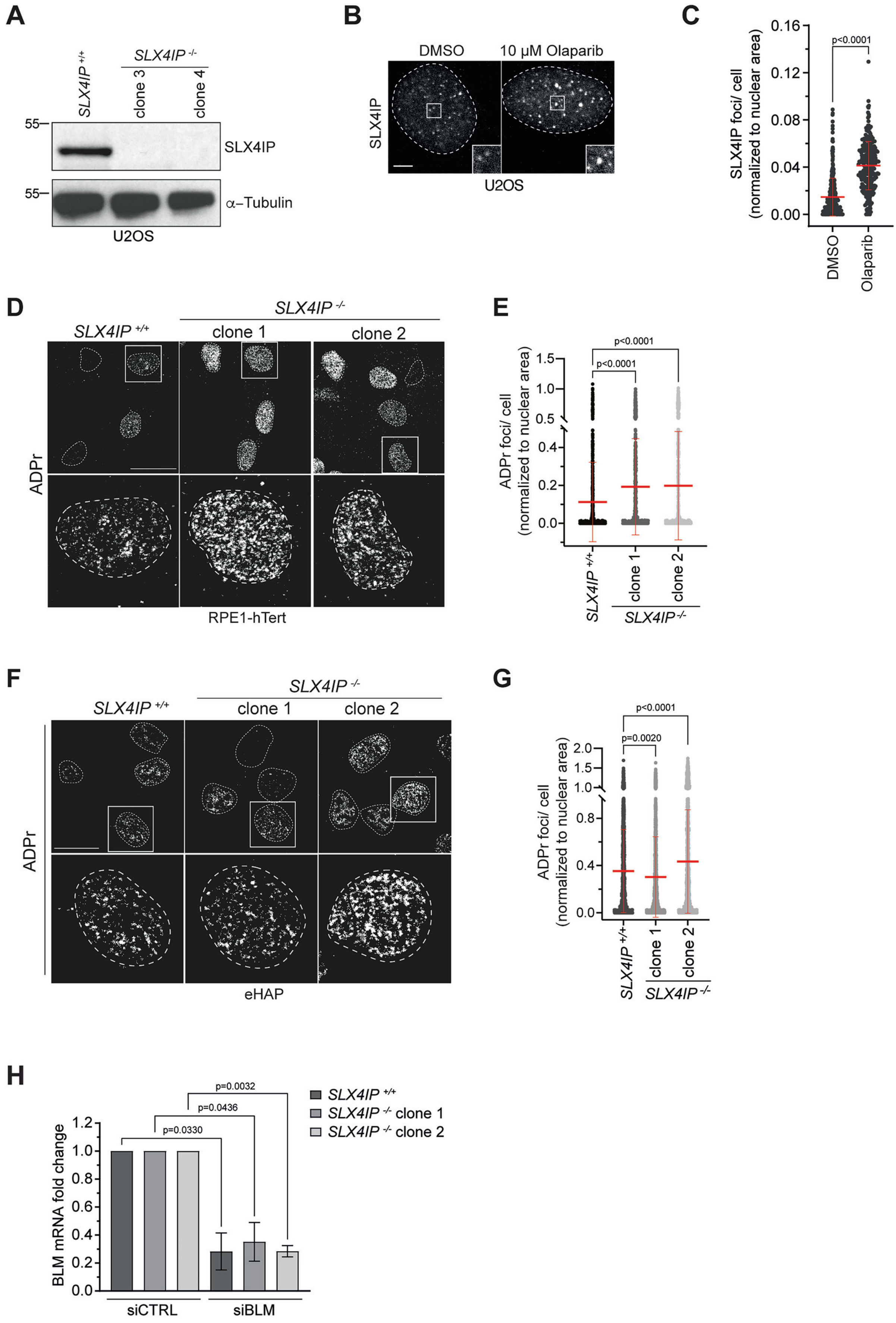
A
SLX4IP +/+
SLX4IP -/-
clone 3
clone 4
55
SLX4IP
55
α–Tubulin
U2OS
B
DMSO
10 μM Olaparib
SLX4IP
U2OS
C
SLX4IP foci/ cell
(normalized to nuclear area)
p<0.0001
0.16
0.12
0.08
0.04
0.00
DMSO
Olaparib
D
SLX4IP +/+
SLX4IP -/-
clone 1
clone 2
ADPr
RPE1-hTert
E
ADPr foci/ cell
(normalized to nuclear area)
p<0.0001
p<0.0001
1.5
1.0
0.5
0.4
0.2
0.0
SLX4IP +/+
clone 1
clone 2
SLX4IP -/-
F
SLX4IP +/+
SLX4IP -/-
clone 1
clone 2
ADPr
eHAP
G
ADPr foci/ cell
(normalized to nuclear area)
p<0.0001
p=0.0020
2.0
1.5
1.0
0.8
0.4
0.0
SLX4IP +/+
clone 1
clone 2
SLX4IP -/-
H
BLM mRNA fold change
p=0.0032
p=0.0436
p=0.0330
1.2
1.0
0.8
0.6
0.4
0.2
0.0
siCTRL
siBLM
SLX4IP +/+
SLX4IP -/- clone 1
SLX4IP -/- clone 2

© The Author(s)

Figure EV3. SLX4IP defi ciency increases ADPr levels on chromatin.

(**A**) Whole-cell lysates of U2OS cells were separated by SDS-PAGE and analyzed for SLX4IP levels by immunoblotting. α-Tubulin was used as a loading control. The numbers on the left denote the molecular weight in kDa. (**B**) U2OS cells were either treated with 10 µM Olaparib or DMSO as a control for 48 h. Cells were then immediately pre-extracted, fixed, and processed for SLX4IP immunofluorescence. Insets are 2X magnifications of the indicated fields. The dotted line represents DAPI (not shown). Scale bar represents 5 µm. (**C**) Quantification of (**B**). The number of SLX4IP foci in each cell was quantified and normalized to nuclear area to account for variations in nuclear size across cells. At least 80 cells per condition and experiment were counted. Data are represented as mean ± SD; $n = 3$, biological replicates; Student's *t* test. The exact *P* value is shown in the figure; $P < 0.05$ was considered statistically significant. (**D**) RPE1-hTert cells were treated with 10 µM PARG inhibitor for 30 min, immediately fixed, and processed for ADPr immunofluorescence. The dotted line represents DAPI (not shown). Lower panels are 3.5X magnifications of the indicated fields. Scale bar represents 25 µm. (**E**) Quantification of (**D**). The number of ADPr foci in each cell was quantified and normalized to nuclear area to account for variations in nuclear size across cells. At least 100 cells per condition and experiment were counted. Data are represented as mean ± SD; $n = 3$, biological replicates; one-way ANOVA with Sidak's multiple comparisons post-test. Exact *P* values are shown in the figure; $P < 0.05$ was considered statistically significant. (**F**) eHAP cells were treated with 10 µM PARG inhibitor for 30 min, immediately fixed, and processed for ADP ribose immunofluorescence. The dotted line represents DAPI (not shown). Lower panels 3X magnifications of the indicated fields. Scale bar represents 15 µm. (**G**) Quantification of (**F**). The number of ADPr foci in each cell was quantified and normalized to nuclear area to account for variations in nuclear size across cells. At least 450 cells per condition and experiment were counted. Data are represented as mean ± SD; $n = 3$, biological replicates; one-way ANOVA with Sidak's multiple comparisons post-test. Exact *P* values are shown in the figure; $P < 0.05$ was considered statistically significant. (**H**) RNA was isolated from cell pellets of each condition, reverse transcribed into cDNA, followed by RT-qPCR. Data were normalized to the siCTRL-treated samples. Data are represented as mean ± SD; $n = 3$, biological replicates; one-way ANOVA with Tukey's multiple comparisons post-test. Exact *P* values are shown in the figure; $P < 0.05$ was considered statistically significant. Relates to Fig. 3D,E.

 © The Author(s)

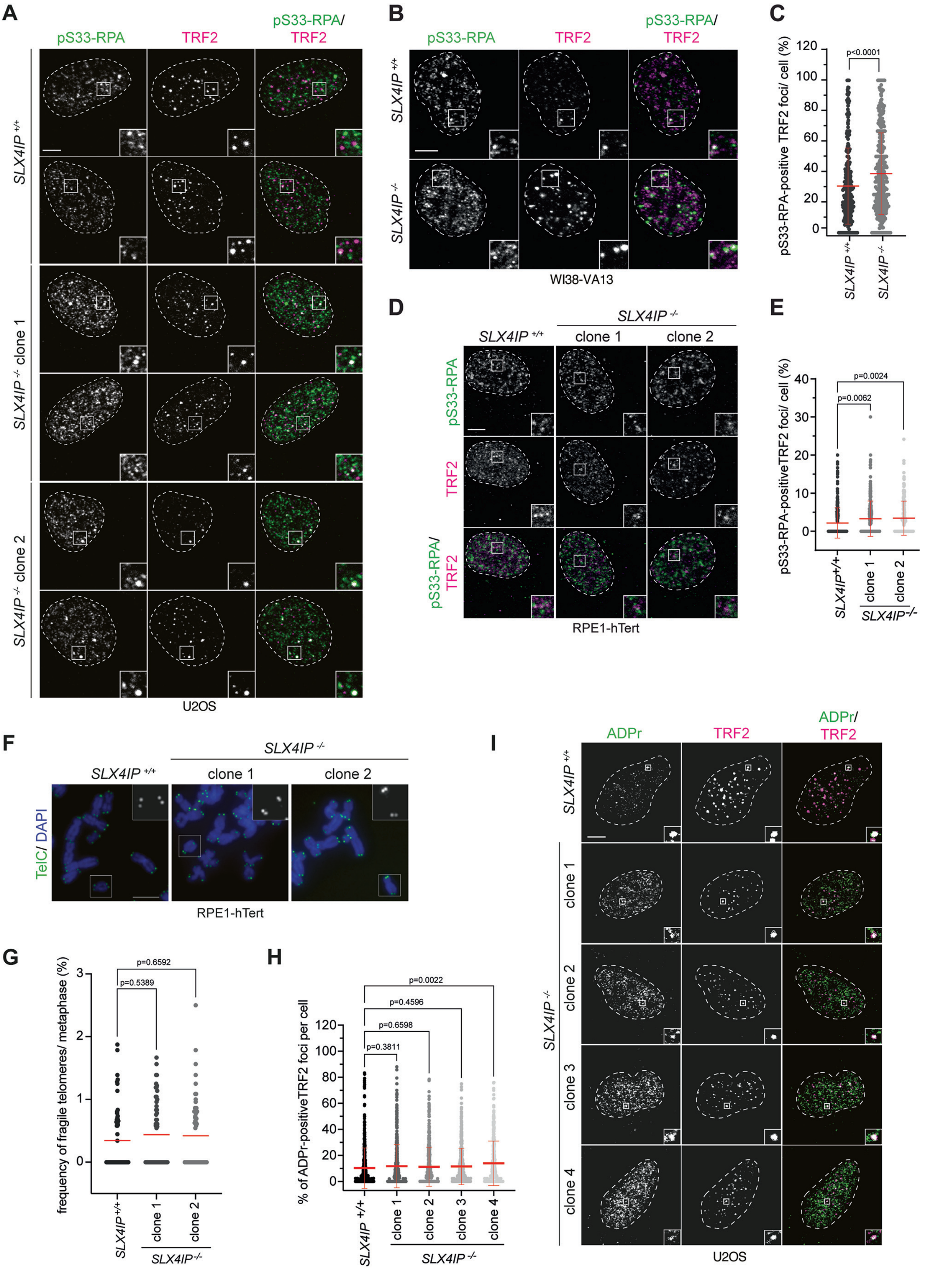
A
pS33-RPA
TRF2
pS33-RPA/ TRF2
SLX4IP +/+
SLX4IP -/- clone 1
SLX4IP -/- clone 2
U2OS
B
pS33-RPA
TRF2
pS33-RPA/ TRF2
SLX4IP +/+
SLX4IP -/-
WI38-VA13
C
pS33-RPA-positive TRF2 foci/ cell (%)
p<0.0001
SLX4IP +/+
SLX4IP -/-
D
SLX4IP +/+
SLX4IP -/-
clone 1
clone 2
pS33-RPA
TRF2
pS33-RPA/ TRF2
RPE1-hTert
E
pS33-RPA-positiveTRF2 foci/ cell (%)
p=0.0024
p=0.0062
SLX4IP+/+
clone 1
clone 2
SLX4IP-/-
F
SLX4IP +/+
SLX4IP -/-
clone 1
clone 2
TelC/ DAPI
RPE1-hTert
G
frequency of fragile telomeres/ metaphase (%)
p=0.6592
p=0.5389
SLX4IP+/+
clone 1
clone 2
SLX4IP-/-
H
% of ADPr-positiveTRF2 foci per cell
p=0.0022
p=0.4596
p=0.6598
p=0.3811
SLX4IP +/+
clone 1
clone 2
clone 3
clone 4
SLX4IP -/-
I
ADPr
TRF2
ADPr/ TRF2
SLX4IP +/+
SLX4IP -/-
clone 1
clone 2
clone 3
clone 4
U2OS

© The Author(s)

Figure EV4. SLX4IP defi ciency causes replication stress at ALT telomeres.

(**A**) U2OS cells were pre-extracted, fixed, and processed for pSer33-RPA and TRF2 immunofluorescence. Insets are 2X magnifications of the indicated fields. The dotted line represents DAPI (not shown). Scale bar represents 5 µm. Additional representative images related to Fig. 4C,D. (**B**) WI38-VA13 cells were pre-extracted, fixed, and processed for pSer33-RPA and TRF2 immunofluorescence. Insets are 2X magnifications of the indicated fields. The dotted line represents DAPI (not shown). Scale bar represents 5 µm. (**C**) Quantification of (**B**). The percentage of TRF2 foci that overlap with pSer33-RPA foci in each cell was quantified. At least 85 cells per condition and experiment were counted. Data are represented as mean ± SD; $n = 3$, biological replicates; Student's *t* test. The exact *P* value is shown in the figure; $P < 0.05$ was considered statistically significant. (**D**) RPE1-hTert cells were pre-extracted, fixed, and processed for pSer33-RPA and TRF2 immunofluorescence. Insets are 2X magnifications of the indicated fields. The dotted line represents DAPI (not shown). Scale bar represents 5 µm. (**E**) Quantification of (**F**). The percentage of TRF2 foci that overlap with pSer33-RPA foci in each cell was quantified. At least 85 cells per condition and experiment were counted. Data are represented as mean ± SD; $n = 3$, biological replicates; one-way ANOVA with Sidak's multiple comparisons post-test. Exact *P* values are shown in the figure; $P < 0.05$ was considered statistically significant. (**F**) RPE1-hTert cells were fixed, and metaphases were processed for telomere PNA (TelC) FISH and DAPI. Insets are ×3 magnifications of the indicated fields. Scale bar represents 100 µm. Arrows indicate fragile telomeres. (**G**) Quantification of (**F**). The number of fragile telomeres counted in each metaphase was normalized to the metaphase size. Data are represented as mean; $n = 2$ with at least 30 metaphases per experiment, biological replicates; one-way ANOVA with Dunnett's multiple comparisons post-test. Exact *P* values are shown in the figure; $P < 0.05$ was considered statistically significant. (**H**) U2OS cells were treated with 10 µM PARG inhibitor for 30 min, fixed, and processed for ADPr and TRF2 immunofluorescence. The number of ADPr foci in each cell was quantified and normalized to nuclear area to account for variations in nuclear size across cells. At least 100 cells per condition and experiment were counted. Data are represented as mean ± SD; $n = 3$, biological replicates; one-way ANOVA with Dunnett's multiple comparisons post-test. Exact *P* values are shown in the figure; $P < 0.05$ was considered statistically significant. (**I**) Representative images of (**H**). The dotted line represents DAPI (not shown). Insets are 3X magnifications of the indicated fields. Scale bar represents 5 µm.

 © The Author(s)

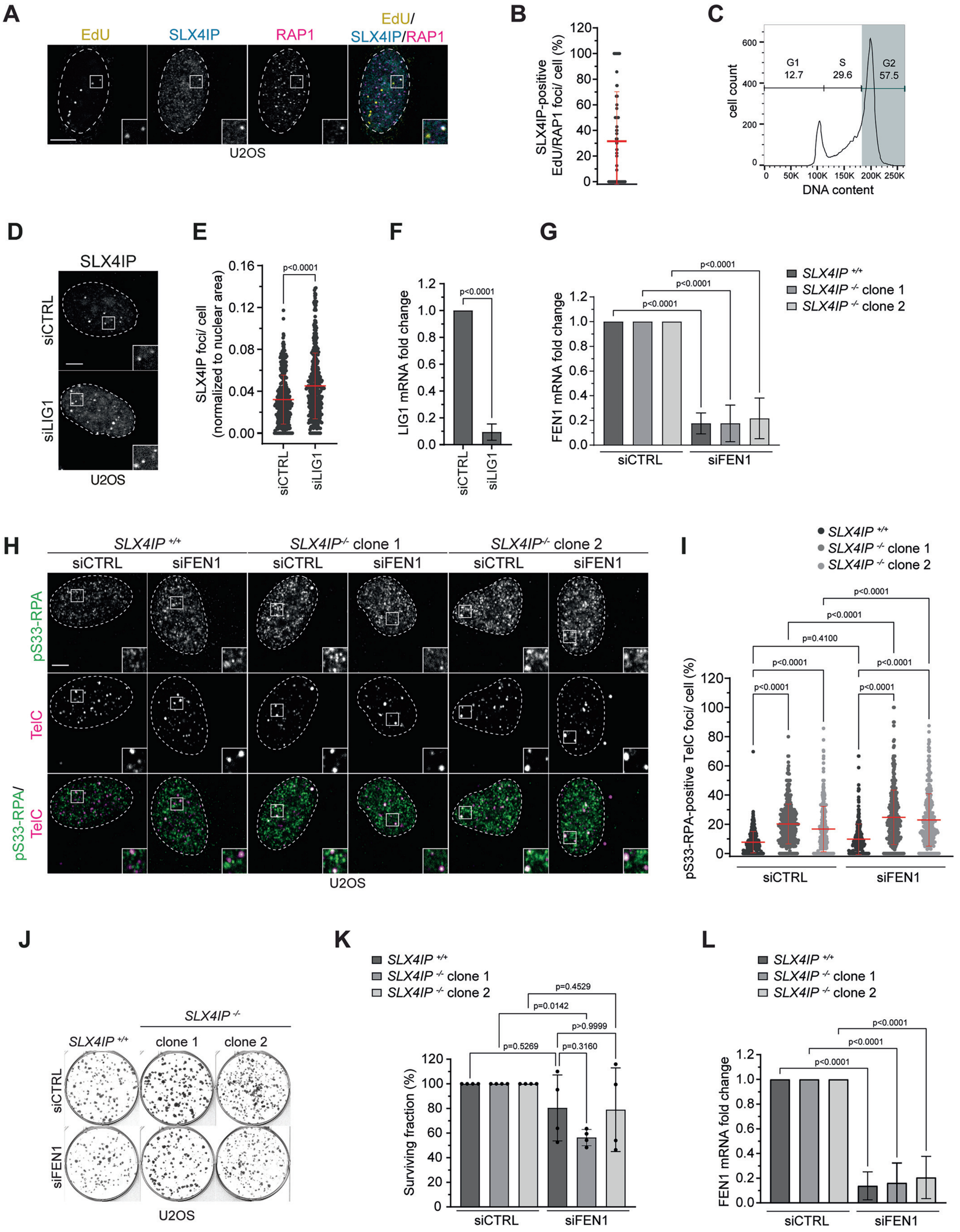
A
EdU
SLX4IP
RAP1
EdU/
SLX4IP/RAP1
U2OS
B
SLX4IP-positive
EdU/RAP1 foci/ cell (%)
C
G1
12.7
S
29.6
G2
57.5
cell count
DNA content
D
SLX4IP
siCTRL
siLIG1
U2OS
E
SLX4IP foci/ cell
(normalized to nuclear area)
p<0.0001
siCTRL
siLIG1
F
LIG1 mRNA fold change
p<0.0001
siCTRL
siLIG1
G
FEN1 mRNA fold change
p<0.0001
siCTRL
siFEN1
SLX4IP +/+
SLX4IP -/- clone 1
SLX4IP -/- clone 2
H
SLX4IP +/+
SLX4IP-/- clone 1
SLX4IP-/- clone 2
siCTRL
siFEN1
pS33-RPA
TelC
pS33-RPA/
TelC
U2OS
I
SLX4IP +/+
SLX4IP -/- clone 1
SLX4IP -/- clone 2
p<0.0001
p=0.4100
pS33-RPA-positive TelC foci/ cell (%)
siCTRL
siFEN1
J
SLX4IP -/-
SLX4IP +/+
clone 1
clone 2
siCTRL
siFEN1
U2OS
K
SLX4IP +/+
SLX4IP -/- clone 1
SLX4IP -/- clone 2
p=0.4529
p=0.0142
p>0.9999
p=0.5269
p=0.3160
Surviving fraction (%)
siCTRL
siFEN1
L
SLX4IP +/+
SLX4IP -/- clone 1
SLX4IP -/- clone 2
p<0.0001
FEN1 mRNA fold change
siCTRL
siFEN1

Figure EV5. SLX4IP potentially regulates lagging-strand replication stress at ALT telomeres.

(**A**) U2OS cells were subjected to a double thymidine block to enrich for G2 cells. An EdU pulse was performed 2 h before fixation, followed by staining of EdU via a Click-IT reaction. SLX4IP and RAP1 were stained by indirect immunofluorescence. Scale bar represents 10 µm. Insets are 2X magnifications of the indicated fields. The dotted line represents DAPI (not shown). Scale bar represents 5 µm. (**B**) Quantification of (**A**). The percentage of SLX4IP foci that overlap with EdU-positive RAP1 foci in G2 cells was quantified and normalized to nuclear area to account for variations in nuclear size across cells. At least 60 cells per experiment were counted. Data are represented as mean ± SD; $n = 3$, biological replicates. (**C**) FACS analysis. U2OS cells subjected to a double thymidine block and released to G2-phase were fixed, stained with DAPI, and analyzed by flow cytometry. The gating was performed based on the cell cycle distribution of asynchronous cells. Related to (**A**). (**D**) U2OS cells were transfected either with non-targeting siRNA (siCTRL) or knocked down for Ligase1 (siLIG1), pre-extracted, fixed, and processed for SLX4IP immunofluorescence. Insets are 2X magnifications of the indicated fields. The dotted line represents DAPI (not shown). Scale bar represents 5 µm. (**E**) Quantification of (**D**). The number of SLX4IP foci in each cell was quantified and normalized to nuclear area to account for variations in nuclear size across cells. At least 100 cells per condition and experiment were counted. Data are represented as mean ± SD; $n = 3$, biological replicates; Student's *t* test. The exact *P* value is shown in the figure; $P < 0.05$ was considered statistically significant. (**F**) RNA was isolated from cell pellets of each condition, reverse transcribed into cDNA, followed by RT-qPCR. Data were normalized to the siCTRL-treated samples; $n = 3$, biological replicates; Student's *t* test. The exact *P* value is shown in the figure; $P < 0.05$ was considered statistically significant. Related to (**D**, **E**). (**G**) RNA was isolated from cell pellets of each condition, reverse transcribed into cDNA, followed by RT-qPCR. Data were normalized to the siCTRL-treated samples. Data are represented as mean ± SD; $n = 3$, biological replicates; one-way ANOVA with Tukey's multiple comparisons post-test. Exact *P* values are shown in the figure; $P < 0.05$ was considered statistically significant. Related to (**H**, **I**). (**H**) U2OS cells were transfected either with non-targeting siRNA (siCTRL) or FEN1 targeting siRNA (siFEN1), pre-extracted, fixed, and processed for pSer33-RPA immunofluorescence followed by telomeric PNA (TelC) FISH. Insets are 2X magnifications of the indicated fields. The dotted line represents DAPI (not shown). Scale bar represents 5 µm. (**I**) Quantification of (**H**). The percentage of TelC foci that overlap with pSer33-RPA foci in each cell was quantified. At least 100 cells per condition and experiment were counted. Data are represented as mean ± SD; $n = 3$, biological replicates; one-way ANOVA with Sidak's multiple comparisons post-test. Exact *P* values are shown in the figure; $P < 0.05$ was considered statistically significant. (**J**) U2OS cells were transfected either with non-targeting siRNA (siCTRL) or FEN1 targeting siRNA (siFEN1) and seeded in a clonogenic survival assay. (**K**) Quantification of (**J**). Data are represented as mean ± SD; $n = 3$, biological replicates; one-way ANOVA with Sidak's multiple comparisons post-test. Exact *P* values are shown in the figure; $P < 0.05$ was considered statistically significant. (**L**) RNA was isolated from cell pellets of each condition, reverse transcribed into cDNA, followed by RT-qPCR. Data were normalized to the siCTRL-treated samples. Data are represented as mean ± SD; $n = 3$, biological replicates; one-way ANOVA with Tukey's multiple comparisons post-test. Exact *P* values are shown in the figure; $P < 0.05$ was considered statistically significant. Related to (**J**, **K**).

 © The Author(s)

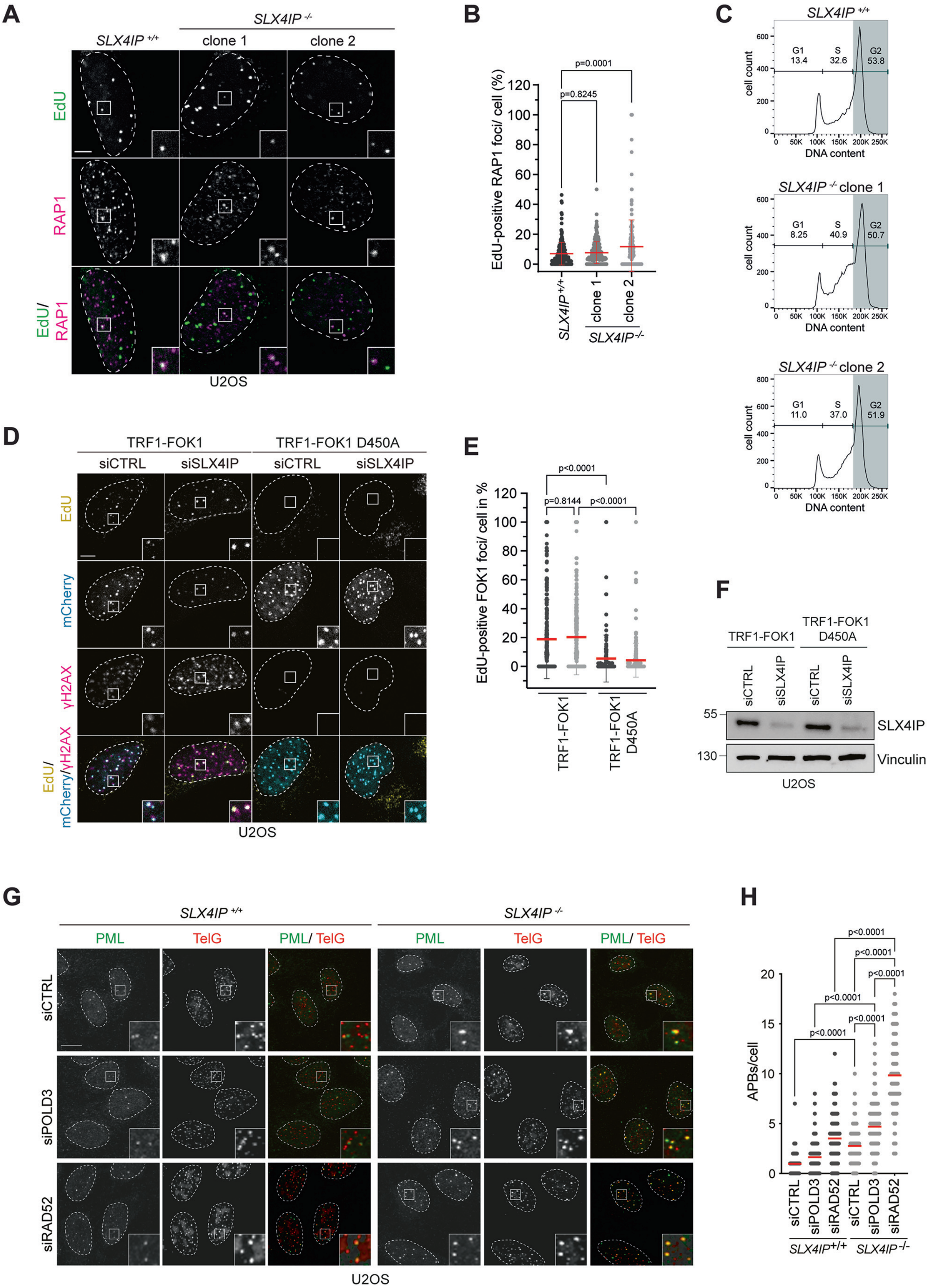
A
SLX4IP +/+
SLX4IP -/-
clone 1
clone 2
EdU
RAP1
EdU/ RAP1
U2OS
B
p=0.0001
p=0.8245
EdU-positive RAP1 foci/ cell (%)
SLX4IP +/+
clone 1
clone 2
SLX4IP-/-
C
SLX4IP +/+
G1 13.4
S 32.6
G2 53.8
cell count
DNA content
SLX4IP -/- clone 1
G1 8.25
S 40.9
G2 50.7
SLX4IP -/- clone 2
G1 11.0
S 37.0
G2 51.9
D
TRF1-FOK1
TRF1-FOK1 D450A
siCTRL
siSLX4IP
EdU
mCherry
γH2AX
EdU/ mCherry/γH2AX
U2OS
E
p<0.0001
p=0.8144
p<0.0001
EdU-positive FOK1 foci/ cell in %
TRF1-FOK1
TRF1-FOK1 D450A
F
TRF1-FOK1
TRF1-FOK1 D450A
siCTRL
siSLX4IP
55
130
SLX4IP
Vinculin
U2OS
G
SLX4IP +/+
SLX4IP -/-
PML
TelG
PML/ TelG
siCTRL
siPOLD3
siRAD52
U2OS
H
p<0.0001
APBs/cell
siCTRL
siPOLD3
siRAD52
SLX4IP+/+
SLX4IP-/-

Figure EV6. SLX4IP is not involved in break-induced replication.

(**A**) U2OS cells were subjected to a double thymidine block to enrich for G2 cells. An EdU pulse was performed 2 h before fixation, followed by staining of EdU via a Click-IT reaction and staining of RAP1 by indirect immunofluorescence. The dotted line represents DAPI (not shown). Insets are 2X magnifications of the indicated fields. Scale bar represents 5 μm. (**B**) Quantification of (**A**). The percentage of RAP1 foci that overlap with EdU foci in each G2 cell was quantified. At least 50 cells per condition and experiment were counted. Data are represented as mean ± SD; $n = 3$, biological replicates; one-way ANOVA with Sidak's multiple comparisons post-test. Exact P values are shown in the figure; $P < 0.05$ was considered statistically significant. (**C**) FACS analysis. U2OS cells subjected to a double thymidine block and released to G2-phase were fixed, stained with DAPI, and analyzed by flow cytometry. The gating was performed based on the cell cycle distribution of asynchronous cells. Related to (**A**, **B**). (**D**) U2OS cells expressing mCherry-labeled TRF1-FOK1 or TRF1-FOK1 D450A were transfected either with non-targeting siRNA (siCTRL) or SLX4IP targeting siRNA (siSLX4IP). They were subjected to a 4 h EdU pulse before fixation, followed by staining of EdU via a Click-IT reaction and staining of γH2AX by indirect immunofluorescence. The dotted line represents DAPI (not shown). Insets are 2X magnifications of the indicated fields. Scale bar represents 5 μm. (**E**) Quantification of (**D**). The percentage of mCherry foci that overlap with EdU foci in each cell was quantified. At least 40 cells per condition and experiment were counted. Data are represented as mean ± SD; $n = 3$, biological replicates; one-way ANOVA with Sidak's multiple comparisons post-test. Exact P values are shown in the figure; $P < 0.05$ was considered statistically significant. (**F**) Whole-cell lysates of U2OS cells were separated by SDS-PAGE and analyzed for SLX4IP levels by immunoblotting. Vinculin was used as a loading control. The numbers on the left denote the molecular weight in kDa. (**G**) U2OS cells were transfected either with non-targeting siRNA (siCTRL), POLD3 targeting siRNA (siPOLD3), or RAD52 targeting siRNA (siRAD52), fixed, and processed for PML immunofluorescence followed by telomeric PNA (TelG) FISH. Insets are 3X magnifications of the indicated fields. The dotted line represents DAPI (not shown). Scale bar represents 10 μm. (**H**) Quantification of (**G**). At least 100 cells per condition and experiment were counted. Mean is indicated; $n = 2$, biological replicates; one-way ANOVA with Tukey's multiple comparisons post-test. Exact P values are shown in the figure; $P < 0.05$ was considered statistically significant.

© The Author(s)

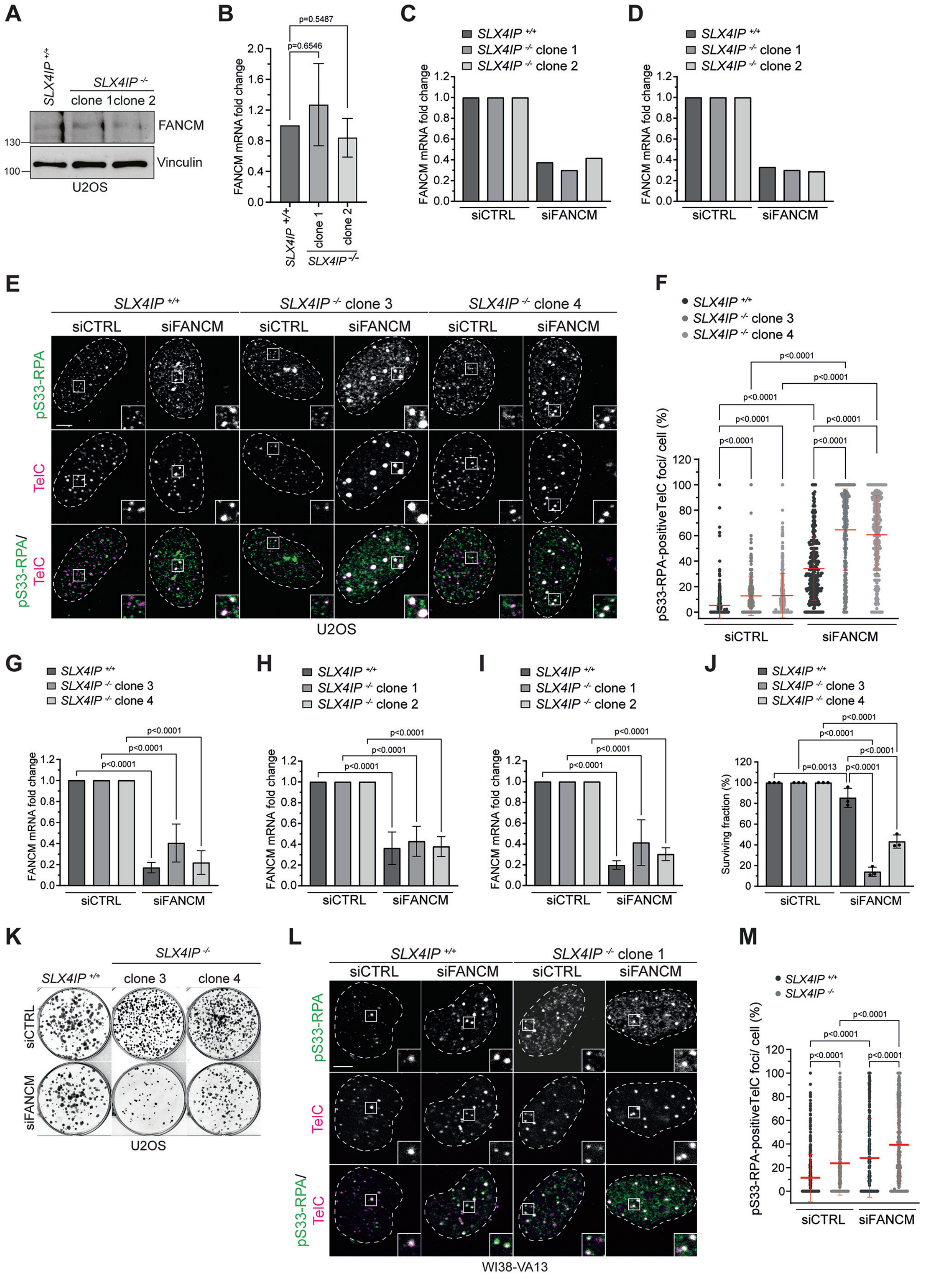
A
SLX4IP +/+
SLX4IP -/-
clone 1
clone 2
FANCM
Vinculin
130
100
U2OS
B
p=0.5487
p=0.6546
FANCM mRNA fold change
2.0
1.6
1.2
0.8
0.4
0.0
SLX4IP +/+
clone 1
clone 2
SLX4IP -/-
C
SLX4IP +/+
SLX4IP -/- clone 1
SLX4IP -/- clone 2
FANCM mRNA fold change
siCTRL
siFANCM
D
SLX4IP +/+
SLX4IP -/- clone 1
SLX4IP -/- clone 2
FANCM mRNA fold change
siCTRL
siFANCM
E
SLX4IP +/+
SLX4IP -/- clone 3
SLX4IP -/- clone 4
siCTRL
siFANCM
pS33-RPA
TelC
pS33-RPA/ TelC
U2OS
F
SLX4IP +/+
SLX4IP -/- clone 3
SLX4IP -/- clone 4
p<0.0001
pS33-RPA-positive TelC foci/ cell (%)
siCTRL
siFANCM
G
SLX4IP +/+
SLX4IP -/- clone 3
SLX4IP -/- clone 4
p<0.0001
FANCM mRNA fold change
siCTRL
siFANCM
H
SLX4IP +/+
SLX4IP -/- clone 1
SLX4IP -/- clone 2
p<0.0001
FANCM mRNA fold change
siCTRL
siFANCM
I
SLX4IP +/+
SLX4IP -/- clone 1
SLX4IP -/- clone 2
p<0.0001
FANCM mRNA fold change
siCTRL
siFANCM
J
SLX4IP +/+
SLX4IP -/- clone 3
SLX4IP -/- clone 4
p<0.0001
p=0.0013
Surviving fraction (%)
siCTRL
siFANCM
K
SLX4IP -/-
SLX4IP +/+
clone 3
clone 4
siCTRL
siFANCM
U2OS
L
SLX4IP +/+
SLX4IP -/- clone 1
siCTRL
siFANCM
pS33-RPA
TelC
pS33-RPA/ TelC
WI38-VA13
M
SLX4IP +/+
SLX4IP -/-
p<0.0001
pS33-RPA-positive TelC foci/ cell (%)
siCTRL
siFANCM

Figure EV7. SLX4IP is synthetic lethal with FANCM in ALT-positive cells.

(**A**) Whole-cell lysates of U2OS cells were separated by SDS-PAGE and analyzed for FANCM levels by immunoblotting. Vinculin was used as a loading control. The numbers on the left denote the molecular weight in kDa. (**B**) RNA was isolated from cell pellets of each condition, reverse transcribed into cDNA, followed by RT-qPCR. Data were normalized to the wild-type samples. Data are represented as mean ± SD; $n = 3$, biological replicates; one-way ANOVA with Dunnett's multiple comparisons post-test. Exact *P* values are shown in the figure; $P < 0.05$ was considered statistically significant. (**C**) RNA was isolated from cell pellets of each condition, reverse transcribed into cDNA followed by RT-qPCR. Data were normalized to the siCTRL-treated samples; $n = 1$. Related to Fig. 5A,B. Panel represents a representative knockdown control experiment. (**D**) RNA was isolated from cell pellets of each condition, reverse transcribed into cDNA, followed by RT-qPCR. Data were normalized to the siCTRL-treated samples; $n = 1$. Related to Fig. 5C,D. Panel represents a representative knockdown control experiment. (**E**) U2OS cells were transfected either with non-targeting siRNA (siCTRL) or FANCM targeting siRNA (siFANCM), pre-extracted, fixed, and processed for pSer33-RPA immunofluorescence followed by telomeric PNA (TelC) FISH. Insets are 2X magnifications of the indicated fields. The dotted line represents DAPI (not shown). Scale bar represents 5 µm. (**F**) Quantification of (**E**). The percentage of TelC foci that overlap with pSer33-RPA foci in each cell was quantified. At least 85 cells per condition and experiment were counted. Data are represented as mean ± SD; $n = 3$, biological replicates; one-way ANOVA with Sidak's multiple comparisons post-test. Exact *P* values are shown in the figure; $P < 0.05$ was considered statistically significant. (**G**) RNA was isolated from cell pellets of each condition, reverse transcribed into cDNA, followed by RT-qPCR. Data were normalized to the siCTRL-treated samples. Data are represented as mean ± SD; $n = 5$, biological replicates; one-way ANOVA with Tukey's multiple comparisons post-test. Exact *P* values are shown in the figure; $P < 0.05$ was considered statistically significant. Related to (**E**, **F**, **J**, **K**). (**H**) RNA was isolated from cell pellets of each condition, reverse transcribed into cDNA followed by RT-qPCR. Data were normalized to the siCTRL-treated samples. Data are represented as mean ± SD; $n = 4$, biological replicates; one-way ANOVA with Tukey's multiple comparisons post-test. Exact *P* values are shown in the figure; $P < 0.05$ was considered statistically significant. Related to Fig. 5E,F. (**I**) RNA was isolated from cell pellets of each condition, reverse transcribed into cDNA, followed by RT-qPCR. Data were normalized to the siCTRL-treated samples. Data are represented as mean ± SD; $n = 4$, biological replicates; one-way ANOVA with Tukey's multiple comparisons post-test. Exact *P* values are shown in the figure; $P < 0.05$ was considered statistically significant. Related to Fig. 5G,H. (**J**) U2OS cells were transfected either with non-targeting siRNA (siCTRL) or FANCM targeting siRNA (siFANCM) and seeded in a clonogenic survival assay. Data are represented as mean ± SD; $n = 3$, biological replicates; one-way ANOVA with Tukey's multiple comparisons post-test. Exact *P* values are shown in the figure; $P < 0.05$ was considered statistically significant. Knockdown validation is shown in (**G**). (**K**) Representative images of (**J**). (**L**) WI38-VA13 cells were transfected either with non-targeting siRNA (siCTRL) or FANCM targeting siRNA (siFANCM), pre-extracted, fixed, and processed for pSer33-RPA immunofluorescence followed by telomeric PNA (TelC) FISH. Insets are 2X magnifications of the indicated fields. The dotted line represents DAPI (not shown). Scale bar represents 5 µm. (**M**) Quantification of (**L**). The percentage of TelC foci that overlap with pSer33-RPA foci in each cell was quantified. At least 100 cells per condition and experiment were counted. Data are represented as mean ± SD; $n = 3$, biological replicates; one-way ANOVA with Sidak's multiple comparisons post-test. Exact *P* values are shown in the figure; $P < 0.05$ was considered statistically significant. Knockdown validation is shown in Fig. EV8C.

 © The Author(s)

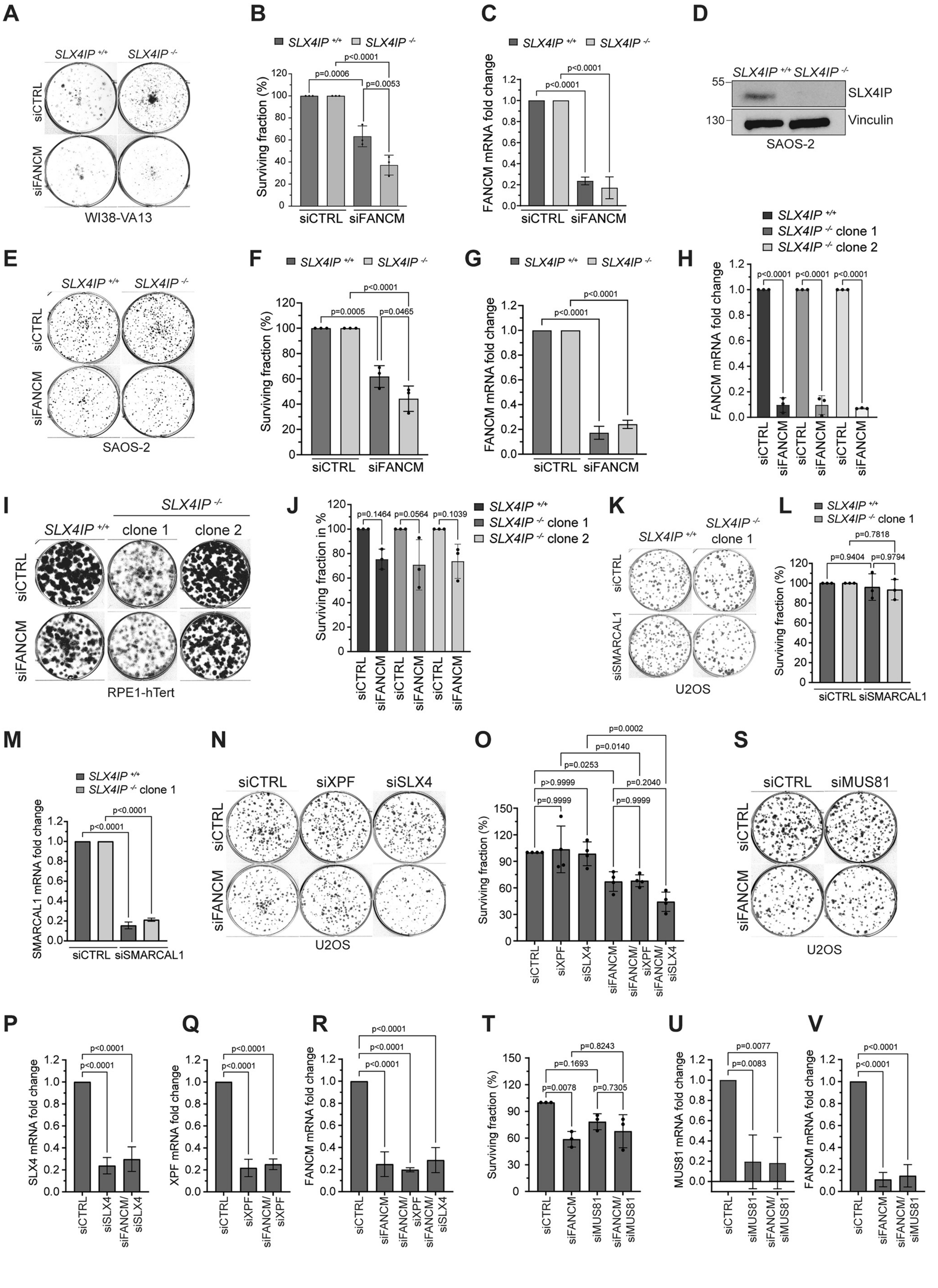
A
SLX4IP +/+
SLX4IP -/-
siCTRL
siFANCM
WI38-VA13
B
SLX4IP +/+
SLX4IP -/-
Surviving fraction (%)
p=0.0006
p<0.0001
p=0.0053
siCTRL
siFANCM
C
FANCM mRNA fold change
p<0.0001
p<0.0001
siCTRL
siFANCM
D
SLX4IP +/+ SLX4IP -/-
55
130
SLX4IP
Vinculin
SAOS-2
E
SAOS-2
F
p=0.0005
p<0.0001
p=0.0465
G
p<0.0001
p<0.0001
H
SLX4IP +/+
SLX4IP -/- clone 1
SLX4IP -/- clone 2
p<0.0001
p<0.0001
p<0.0001
I
SLX4IP -/-
clone 1
clone 2
RPE1-hTert
J
Surviving fraction in %
p=0.1464
p=0.0564
p=0.1039
K
U2OS
siSMARCAL1
L
p=0.7818
p=0.9404
p=0.9794
siCTRL siSMARCAL1
M
SMARCAL1 mRNA fold change
p<0.0001
p<0.0001
N
siCTRL
siXPF
siSLX4
siFANCM
U2OS
O
p=0.0002
p=0.0140
p=0.0253
p>0.9999
p=0.2040
p=0.9999
p=0.9999
siCTRL
siXPF
siSLX4
siFANCM
siFANCM/siXPF
siFANCM/siSLX4
S
siCTRL
siMUS81
siFANCM
U2OS
P
SLX4 mRNA fold change
p<0.0001
p<0.0001
siFANCM/siSLX4
Q
XPF mRNA fold change
p<0.0001
p<0.0001
siFANCM/siXPF
R
FANCM mRNA fold change
p<0.0001
p<0.0001
p<0.0001
T
p=0.8243
p=0.1693
p=0.0078
p=0.7305
siMUS81
siFANCM/siMUS81
U
MUS81 mRNA fold change
p=0.0077
p=0.0083
V
FANCM mRNA fold change
p<0.0001
p<0.0001

Figure EV8. The synthetic lethal interaction between SLX4IP and FANCM is specifi c to ALT-positive cells.

(**A**) WI38-VA13 cells were transfected either with non-targeting siRNA (siCTRL) or FANCM targeting siRNA (siFANCM) and seeded in a clonogenic survival assay. (**B**) Quantification of (**A**). Data are represented as mean ± SD; $n = 3$, biological replicates; one-way ANOVA; with Tukey's multiple comparisons post-test. Exact P values are shown in the figure; $P < 0.05$ was considered statistically significant. (**C**) RNA was isolated from cell pellets of each condition, reverse transcribed into cDNA followed by RT-qPCR. Data were normalized to the siCTRL-treated samples. Data are represented as mean ± SD; $n = 3$, biological replicates; one-way ANOVA with Tukey's multiple comparisons post-test. Exact P values are shown in the figure; $P < 0.05$ was considered statistically significant. Related to Fig. EV7L,M and **A**, **B**). (**D**) Whole-cell lysates of SAOS-2 cells were separated by SDS-PAGE and analyzed for SLX4IP levels by immunoblotting. Vinculin was used as a loading control. The numbers on the left denote the molecular weight in kDa. (**E**) SAOS-2 cells were transfected either with non-targeting siRNA (siCTRL) or FANCM targeting siRNA (siFANCM) and seeded in a clonogenic survival assay. (**F**) Quantification of €. Data are represented as mean ± SD; $n = 3$, biological replicates; one-way ANOVA with Tukey's multiple comparisons post-test. Exact P values are shown in the figure; $P < 0.05$ was considered statistically significant. (**G**) RNA was isolated from cell pellets of each condition, reverse transcribed into cDNA, followed by RT-qPCR. Data were normalized to the siCTRL-treated samples. Data are represented as mean ± SD; $n = 3$, biological replicates; one-way ANOVA with Tukey's multiple comparisons post-test. Exact P values are shown in the figure; $P < 0.05$ was considered statistically significant. Related to (**E**, **F**). (**H**) RNA was isolated from cell pellets of each condition, reverse transcribed into cDNA, followed by RT-qPCR. Data were normalized to the siCTRL-treated samples. Data are represented as mean ± SD; $n = 3$, biological replicates; one-way ANOVA with Tukey's multiple comparisons post-test. Exact P values are shown in the figure; $P < 0.05$ was considered statistically significant. Related to (**I**, **J**). (**I**) RPE1-hTert cells were transfected either with non-targeting siRNA (siCTRL) or FANCM targeting siRNA (siFANCM) and seeded in a clonogenic survival assay. (**J**) Quantification of (**I**). Data are represented as mean ± SD; $n = 3$, biological replicates; one-way ANOVA with Tukey's multiple comparisons post-test. Exact P values are shown in the figure; $P < 0.05$ was considered statistically significant. Knockdown validation is shown in (**H**). (**K**) U2OS cells were transfected either with non-targeting siRNA (siCTRL) or SMARCAL1 targeting siRNA (siSMARCAL1) and seeded in a clonogenic survival assay. (**L**) Quantification of (**K**). Data are represented as mean ± SD; $n = 3$, biological replicates; one-way ANOVA with Tukey's multiple comparisons post-test. Exact P values are shown in the figure; $P < 0.05$ was considered statistically significant. (**M**) RNA was isolated from cell pellets of each condition, reverse transcribed into cDNA, followed by RT-qPCR. Data were normalized to the siCTRL-treated samples. Data are represented as mean ± SD; $n = 3$, biological replicates, one-way ANOVA with Tukey's multiple comparisons post-test. Related to (**K**, **L**). (**N**) U2OS cells were transfected with the denoted combinations of non-targeting siRNA (siCTRL), siFANCM, siSLX4, and siXPF. Cells were then seeded in a clonogenic survival assay. (**O**) Quantification of (**N**). Data are represented as mean ± SD; $n = 4$, biological replicates; one-way ANOVA with Sidak's multiple comparisons post-test. Exact P values are shown in the figure; $P < 0.05$ was considered statistically significant. (**P**) RNA was isolated from cell pellets of each condition, reverse transcribed into cDNA, followed by RT-qPCR. Data were normalized to the siCTRL-treated samples. Data are represented as mean ± SD; $n = 4$, biological replicates; one-way ANOVA with Tukey's multiple comparisons post-test. Exact P values are shown in the figure; $P < 0.05$ was considered statistically significant. Related to (**N**, **O**). (**Q**) RNA was isolated from cell pellets of each condition, reverse transcribed into cDNA followed by RT-qPCR. Data were normalized to the siCTRL-treated samples. Data are represented as mean ± SD; $n = 4$, biological replicates; one-way ANOVA with Tukey's multiple comparisons post-test. Exact P values are shown in the figure; $P < 0.05$ was considered statistically significant. Related to (**N**, **O**). (**R**) RNA was isolated from cell pellets of each condition, reverse transcribed into cDNA, followed by RT-qPCR. Data were normalized to the siCTRL-treated samples. Data are represented as mean ± SD; $n = 4$, biological replicates; one-way ANOVA with Tukey's multiple comparisons post-test. Exact P values are shown in the figure; $P < 0.05$ was considered statistically significant. Related to (**N**, **O**). (**S**) U2OS cells were transfected with the denoted combinations of non-targeting siRNA (siCTRL), siFANCM, and siMUS81. Cells were then seeded in a clonogenic survival assay. (**T**) Quantification of (**S**). Data are represented as mean ± SD; $n = 3$, biological replicates; one-way ANOVA with Sidak's multiple comparisons post-test. Exact P values are shown in the figure; $P < 0.05$ was considered statistically significant. (**U**) RNA was isolated from cell pellets of each condition, reverse transcribed into cDNA, followed by RT-qPCR. Data were normalized to the siCTRL-treated samples. Data are represented as mean ± SD; $n = 3$, biological replicates; one-way ANOVA with Tukey's multiple comparisons post-test. Exact P values are shown in the figure; $P < 0.05$ was considered statistically significant. Related to (**S**, **T**). (**V**) RNA was isolated from cell pellets of each condition, reverse transcribed into cDNA, followed by RT-qPCR. Data were normalized to the siCTRL-treated samples. Data are represented as mean ± SD; $n = 3$, biological replicates; one-way ANOVA with Tukey's multiple comparisons post-test. Exact P values are shown in the figure; $P < 0.05$ was considered statistically significant. Related to (**S**, **T**).

© The Author(s)

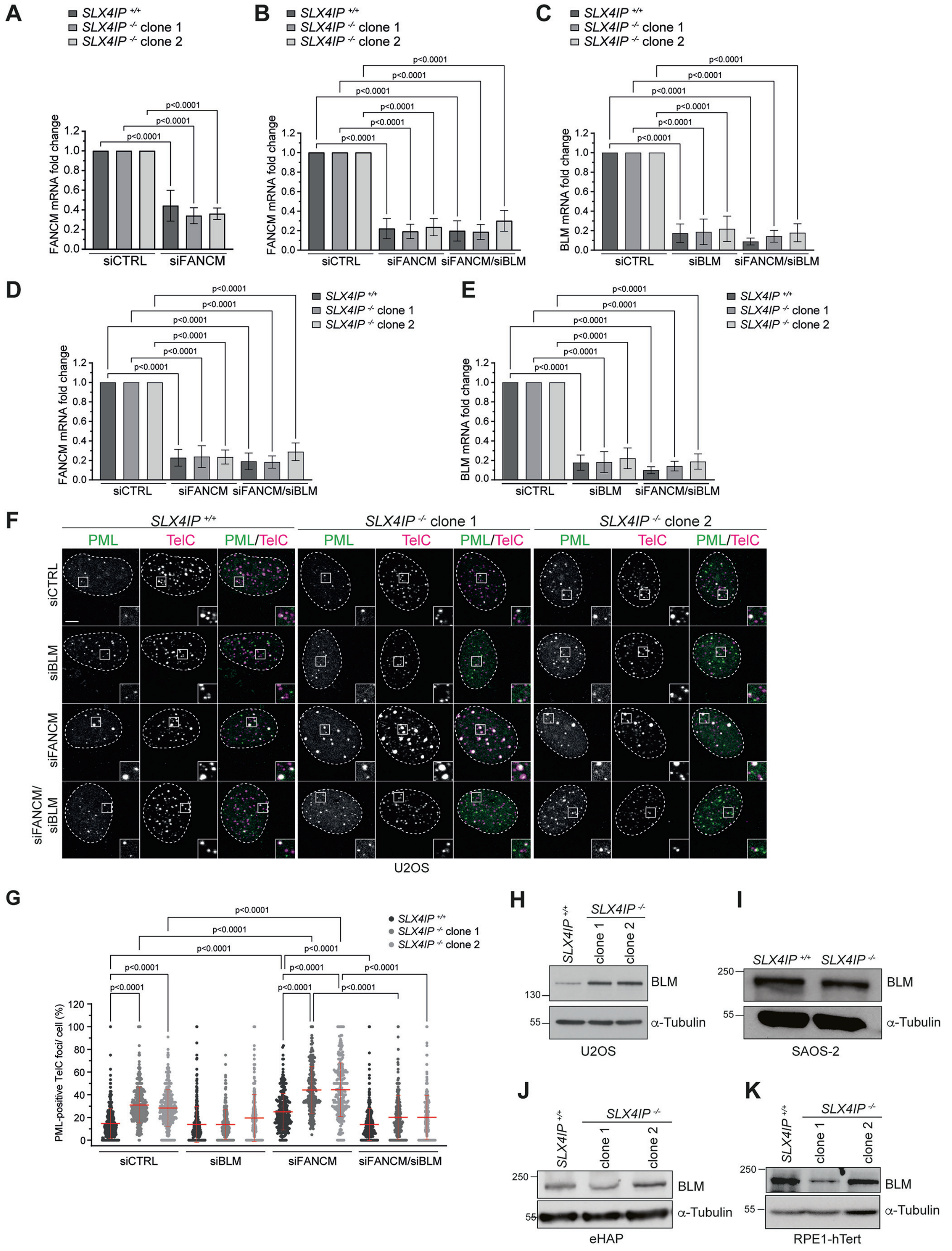
A
B
C
D
E
F
G
H
I
J
K
SLX4IP +/+
SLX4IP -/- clone 1
SLX4IP -/- clone 2
FANCM mRNA fold change
BLM mRNA fold change
p<0.0001
siCTRL
siFANCM
siBLM
siFANCM/siBLM
PML
TelC
PML/TelC
siFANCM/
siBLM
U2OS
PML-positive TelC foci/ cell (%)
SLX4IP -/-
clone 1
clone 2
BLM
α-Tubulin
SAOS-2
eHAP
RPE1-hTert
250
130
55

Figure EV9. Co-depletion of BLM rescues SLX4IP/FANCM-dependent telomere phenotypes.

(**A**) RNA was isolated from cell pellets of each condition, reverse transcribed into cDNA, followed by RT-qPCR. Data were normalized to the siCTRL-treated samples. Data are represented as mean ± SD; $n = 3$, biological replicates; one-way ANOVA with Tukey's multiple comparisons post-test. Exact P values are shown in the figure; $P < 0.05$ was considered statistically significant. Related to Fig. 6A,B. (**B**) RNA was isolated from cell pellets of each condition, reverse transcribed into cDNA, followed by RT-qPCR. Data were normalized to the siCTRL-treated samples. Data are represented as mean ± SD; $n = 3$, biological replicates; one-way ANOVA with Tukey's multiple comparisons post-test. Exact P values are shown in the figure; $P < 0.05$ was considered statistically significant. Related to Fig. 6C,D. (**C**) RNA was isolated from cell pellets of each condition, reverse transcribed into cDNA, followed by RT-qPCR. Data were normalized to the siCTRL-treated samples. Data are represented as mean ± SD; $n = 3$, biological replicates; one-way ANOVA with Tukey's multiple comparisons post-test. Exact P values are shown in the figure; $P < 0.05$ was considered statistically significant. Related to Fig. 6C,D. (**D**) RNA was isolated from cell pellets of each condition, reverse transcribed into cDNA followed by RT-qPCR. Data were normalized to the siCTRL-treated samples. Data are represented as mean ± SD; $n = 4$, biological replicates; one-way ANOVA with Tukey's multiple comparisons post-test. Exact P values are shown in the figure; $P < 0.05$ was considered statistically significant. Related to Fig. 6E,F. (**E**) RNA was isolated from cell pellets of each condition, reverse transcribed into cDNA, followed by RT-qPCR. Data were normalized to the siCTRL-treated samples. Data are represented as mean ± SD; $n = 4$, biological replicates; one-way ANOVA with Tukey's multiple comparisons post-test. Exact P values are shown in the figure; $P < 0.05$ was considered statistically significant. Related to Fig. 6E,F. (**F**) U2OS cells were transfected either with non-targeting siRNA (siCTRL) or knocked down for FANCM (siFANCM), BLM (siBLM), or both (siFANCM/siBLM). Cells were pre-extracted, fixed, and processed for PML immunofluorescence followed by telomeric PNA (TelC) FISH. Insets are 2X magnifications of the indicated fields. The dotted line represents DAPI (not shown). Scale bar represents 5 µm. (**G**) Quantification of (**F**). APBs were quantified as the percentage of TelC foci that overlap with PML foci in each cell. At least 68 cells per condition and experiment were counted. Data are represented as mean ± SD; $n = 3$, biological replicates; one-way ANOVA with Sidak's multiple comparisons post-test. Exact P values are shown in the figure; $P < 0.05$ was considered statistically significant. Knockdown validation is shown in (**B**, **C**). (**H**) Whole-cell lysates of U2OS cells were separated by SDS-PAGE and analyzed for BLM levels by immunoblotting. α-Tubulin was used as a loading control. The numbers on the left denote the molecular weight in kDa. (**I**) Whole-cell lysates of SAOS-2 cells were separated by SDS-PAGE and analyzed for BLM levels by immunoblotting. α-Tubulin was used as a loading control. The numbers on the left denote the molecular weight in kDa. (**J**) Whole-cell lysates of eHap cells were separated by SDS-PAGE and analyzed for BLM levels by immunoblotting. α-Tubulin was used as a loading control. The numbers on the left denote the molecular weight in kDa. (**K**) Whole-cell lysates of eHap cells were separated by SDS-PAGE and analyzed for BLM levels by immunoblotting. α-Tubulin was used as a loading control. The numbers on the left denote the molecular weight in kDa.

 © The Author(s)
