# Supplementary material for: SLX4IP limits replication stress globally and at ALT telomeres
Source: EMBO J. 2026 May 7;45(12):4176–219. doi: 10.1038/s44318-026-00790-4 (PMC13269807; doi:10.1038/s44318-026-00790-4)
Supplement: Supplementary file 1 — Peer Review File [file 44318_2026_790_MOESM1_ESM.pdf]

THE EMBO JOURNAL

SLX4IP limits replication stress globally and at ALT telomeres

Jessica Spindler, Francesca Pandolfo, Anna Koch, Priscilla Piccirillo, Drew Jordahl, Nikhil Venkatesh, Dhruthi Suresh, K Ylvisaker, Anita Jopkiewicz, Johanna Bihler, Sandra Buschbaum, Marcel Morgenstern, Katherine Overmyer, Estelle Vincendeau, Joshua Coon, Pei-Chi Wei, Robert Hänsel-Hertsch, Kavi Mehta, and Stephanie Panier

Corresponding author(s): Stephanie Panier (panier@age.mpg.de) , Kavi Mehta (kmehta@wisc.edu)

Review Timeline:

Submission Date:	19th Jun 25
Editorial Decision:	1st Aug 25
Revision Received:	26th Feb 26
Editorial Decision:	9th Apr 26
Revision Received:	15th Apr 26
Accepted:	17th Apr 26

Editor: Hartmut Vodermaier

Transaction Report:

(Note: With the exception of the correction of typographical or spelling errors that could be a source of ambiguity, letters and reports are not edited. Depending on transfer agreements, referee reports obtained elsewhere may or may not be included in this compilation. Referee reports are anonymous unless the Referee chooses to sign their reports.)

Dear Stephanie,

Thank you for submitting your manuscript on SLX4IP-BLM interplay in ALT and replication stress for our consideration. I sent it to three expert referees, who have now returned the below-copied reports. As you will see, the reports are somewhat equivocal at this stage: although the referees overall appreciate the clarity of the study and the interest of its main findings, they also note that the overall advance conveyed by them is somewhat limited. Moreover, they all remain unsure whether the data are decisive enough to implicate the SLX4IP role specifically in ALT/at telomeres, compared to a more general/genome-wide replication function.

Since it is not clear whether these key concerns could be adequately clarified during revision -which would in my view be required to make the study a sufficient advance to warrant EMBO Journal publication- I would at this point invite you to carefully consider the reviewers' comments, and get back to me with a tentative point-by-point response by early/mid-next week. Based on such a revision plan, I could then get a better understanding of whether a revision for The EMBO Journal would seem realistic, or whether the study would be a better candidate for rapid publication in one of our sister journals (such as EMBO Reports or Life Science Alliance).

Looking forward to hearing from you,

Best regards,

Hartmut

Hartmut Vodermaier, PhD
Senior Editor, The EMBO Journal
h.vodermaier@embojournal.org

Referee #1 (Report for Author)

General Summary
In this manuscript, Panier and colleagues investigate the contribution of SLX4IP to the Alternative Lengthening of Telomeres (ALT) mechanism. Using a combination of fluorescence imaging, proteomics, and molecular analyses of DNA replication, they

describe a connection between SLX4IP and FANCM in regulating BLM action within ALT processes. SLX4IP knockout cells exhibit a hyperactive ALT phenotype with accumulation of PML and replicative stress markers, including phospho-RPA2 and phospho-CHK1 at telomeres. Additionally, DNA fiber analysis reveals delayed replication due to SLX4IP loss. Mechanistically, these adverse effects are linked with deregulated BLM activity, whose levels accumulate globally but also at telomeres due to SLX4IP loss. The authors provide evidence that SLX4IP acts with FANCM against BLM, with depletion of FANCM leading to the death of SLX4IP-deficient cells. Notably, this cell death phenotype is partially reversed by co-depletion of BLM. Thus, a model is proposed envisioning that the role of the SLX4IP-FANCM axis is to limit BLM activity in ALT cancer cells.

This is a nice study, with some relevant insights into the phenotypic impact of SLX4IP loss on the ALT mechanism, with the connection to FANCM and BLM. A series of diverse approaches is employed to study SLX4IP (CUTnTAG, IPOND, DNA fibers). The finding of a synthetic lethal interaction between FANCM and SLX4IP is notable. However, on the negative side, the study suffers from going back and forth between telomeres and general aspects of DNA replication. While the general replication observations are nice, they seem to detract from the overall claim/goal of delineating how SLX4IP ultimately regulates ALT. It raises questions as to SLX4IP's role in ALT, considering the nearly identical distribution of the protein in U2OS and Hek cells as mapped by CUTnTAG. In its present state, it is difficult to interpret the results of the study as otherwise revealing that SLX4IP is a general replication factor with its absence having an as yet unexplained effect on BLM. We recommend that the authors provide additional evidence to support their claims.

Specific major concerns

1. Overall, the manuscript seeks further insights into the role of SLX4IP in the ALT mechanism. Yet, much of the presented data relates to a more general role for SLX4IP in DNA replication. It would be prudent to assess the impact of SLXIP loss on telomere replication and specifically as part of regulating the ALT mechanism. For instance, are the DNA replication defects occurring at telomeres during G2/M phase? Is the break-induced replication mechanism impacted by SLX4IP loss? Is SLX4IP actively recruited or displaced from telomeres actively engaging in telomere extension? The contribution of SLX4IP to telomere regulation by ALT could be assessed more directly. This will strengthen the overall premise and message of the study.

2. Figure 3 shows western blot analysis with a substantial increase in BLM protein levels in SLX4IP-deficient cells. What is the mechanism or molecular basis for this deregulation of BLM? Is this increase in BLM levels specific to SLX4IP-deficient ALT cancer cell lines (i.e. SLX4IP SLX4IP-deficient normal cells or cancer cells expressing telomerase? The authors appropriately cite their previous work showing that SLX4IP does not directly inhibit BLM in vitro, yet do not fully reconcile that point. This should be developed further.

3. Figure 4 summarizes iPOND-MS experiments that show increased histone variants and immature/unligated Okazaki fragments at replication forks in SLX4IP-deficient cells. Overall, this section of the study seems to be particularly preliminary and underdeveloped. Neither aspect is presented in a way directly connecting with ALT.
o First, can the author comment on the rationale for excluding the top hits from consideration for follow-up?
o Second, can the authors give a reason for why there are more histone variants present, considering the presumed accumulation of ssDNA, which is a sub-optimal substrate for nucleosome assembly, at these replication forks?
o Third, the assessment of ADP-ribosylation in SLX4IP-deficient cells is interesting but again preliminary. The S1 sensitivity seems somewhat modest and should be further interrogated to strengthen the authors claims. In addition, as it relates to ALT, can ADP-ribose/PAR foci be detected at telomeres and if so, does this only occur in ALT cancer cell lines and outside of S-phase in G2-phase?
o Fourth, we note that the authors state that they observe increased SLX4IP on chromatin after treatment with Olaparib. Surely this would suggest a PAR-independent basis of SLX4IP recruitment and not that SLX4IP is recruited to manage replication-associated PARylation, as is stated on pg 13.
4. A figure describing the final model or summary of findings should be provided in the final figure.
5. Lastly, the conclusions are based almost exclusively on experiments performed with the U2OS cell line. While this line is widely used as a model for ALT, it is far from representative of all ALT cancers. To strengthen the findings as well as the key claim of potential clinical relevance, the authors should validate their most critical findings in at least one, preferably several, other ALT-positive cell lines.
Minor Concerns
1. The abstract states "Despite its essential role in ALT, BLM must be tightly controlled....". Surely, because of its essential role, it should be tightly controlled.
2. Expanded View Figure 2A-B: missing error bars.
3. At some points, figures are called either Expanded View or Supplementary Figures.
4. In Figure 4, why is ADP-ribose foci per nucleus size per cell used as quantification?

Referee #2 (Report for Author)

In this manuscript, the authors build upon a previous study to further investigate the role of SLX4IP at ALT telomeres. The authors demonstrate that SLX4IP depletion causes an induction of lagging strand specific telomere replication stress in ALT cells, that this

replication stress is heightened by co-depletion of FANCM, that this synthetic lethal interaction involves BLM and can be rescued by BLM depletion, and that SLX4IP functions to restrain the activity of BLM in unwinding unligated Okazaki fragments. Overall, this is a well presented and clear manuscript. The conceptual advance over the previous study is a little limited, but the data are compelling.

Points to address:

- Are the effects of SLX4IP depletion upon replication stress telomere-specific and/or ALT-cell selective? This should be clarified throughout, as some experiments are very clearly focussed on the telomeres, while others (eg Cut & Tag, iPOND etc) are not.
- In Figure 1E - does the addition of HU significantly reduce the fork speed in the absence of SLX4IP, ie. are columns 2 vs 4 statistically significant?
- In Figure 1F - the pS33-RPA staining looks quite excessive, and it is difficult to see an increase in colocalizations. Clone 1 appears to have less colocalizations. Is the antibody specific and are there more representative images?
- Colocalizations are difficult to discern in all IF/FISH images (for example Figure 3D). Arrows might help, but I appreciate this may get crowded in some images.
- I am unclear about the use and interpretation of Cut & Tag. The aim was to determine if SLX4IP was recruited to ALT telomeres during replication stress. The results in the Supp Figure 1E-G do not report peaks at telomeric regions vs non-telomeric regions, but rather show an increase in promoters, fragile sites, etc throughout the genome. How does this (i) address whether the localisation of SLX4IP is telomeric, and (ii) support the conclusion that "these results support the role of SLX4IP in mitigating replication stress at ALT telomeres"? It seems that SLX4IP is responding globally to replication stress and that this is happening independently of ALT activity. The result of this response specifically at telomeres needs further clarity, ie. - is SLX4IP playing a role specifically at ALT telomeres, or is it more genomic and the telomere specific effects and synthetic lethality are attributed to FANCM depletion. Is it possible to evaluate telomeric binding using Cut & Tag?
- Consistent with this concept, it would be useful to examine whether the increase in fragile telomeres and decrease in fork speed after loss of SLX4IP are also seen in ALT-negative cells.
- Do the levels of FANCM at ALT telomeres increase following loss of SLX4IP?
- A significant increase in PAR signal was observed following loss of SLX4IP, indicative of unligated Okazaki fragments. This was reversed with BLM depletion, suggesting that unrestrained BLM activity results in unwound and unligated Okazaki fragments. Is this genomic or telomere-specific? Could this be combined with telomere-FISH to demonstrate a telomeric effect?
- Does FEN1 knockdown reverse the heightened replication stress and unligated Okazaki

fragments caused by SLX4IP/FANCM loss to similar levels as BLM knockdown?

- Lines 123 and 199, FANCM binds to RMI1/2 rather than directly to BLM
- Line 220/221 - "modest"? (word missing).

Referee #3 (Report for Author)

Spindler et al. identify roles of SLX4IP in regulating the BLM helicase during DNA replication. The authors find in U2OS ALT cancer cells that SLX4IP KO clones have higher rates of telomere fragility and reduced replication fork speed throughout the genome. The replication stress marker pSer33RPA was increased at telomeres and pSer345CHK1 foci were also detected. HU-treatment led to SLX4IP accumulation at many loci in the genome as determined by cut&tag profiling indicating genome-wide roles of SLX4IP in response to replication stress. Depletion of the DNA translocase FANCM with SLX4IP-KO showed additive effects on replication stress at telomeres as well as synthetic growth defects. FANCM has been reported before to reduce replication stress at ALT telomeres. Published work also demonstrated that the reduced viability of FANCM-deficient ALT cells depends on BLM. It is reported here that the same is true for SLX4IP. Depletion of BLM in SLX4IP-deficient cells rescued replication stress as well as growth defects. Altogether, SLX4IP appears to prevent the toxic effects of excessive BLM activity. Determination of protein composition near replication forks by iPOND revealed among others enrichment of the flap endonuclease FEN1. Furthermore, by combining DNA combing analysis with S1 nuclease treatment it was found that single stranded gaps accumulated in the analyzed SLX4IP-KO clone. Poly(ADP) ribosylation also increased in the nucleus, which depended on BLM. Altogether the data suggest that loss of SLX4IP leads to defects in lagging strand synthesis throughout the genome by mis-regulation of BLM.

Major concern and comments:

- Clonal variation of telomere phenotypes is a common phenomenon which has been described in several publications. For example, telomere length varies substantially between clones of telomerase-positive cells. Clonal variation for other telomere phenotypes is also highly likely. Consistent with this notion, the two SLX4IP-KO clones show different levels of telomere fragility and marked differences in the other phenotypes described in the manuscript. For replication fork speed and single stranded gaps, only one clone is analyzed. I'm doubtful that the analysis of two or one clone is sufficient to exclude that the effects are not due to clonal variation. In order to achieve convincing results, the sample size of KO-clones must may be significantly increased or it must be demonstrated that expression of wild SLX4IP (e.g. from a cDNA) in the KO-background can rescue the phenotypes. Perhaps the latter experiment is less work.

• The results indicate that SLX4IP restricts BLM activity during replication not only at telomeres but throughout the genome. Although the project may have initially been aimed at investigating specific roles of SLX4IP at ALT telomeres the results indicate much broader roles of SLX4IP. Therefore, I recommend a considerable overhaul of the writing and emphasis of the paper. The title does also not reflect the general roles of SLX4IP in DNA replication which are discovered here. The paper might also reach a broader readership by addressing the general DNA replication community.
• It is found that loss of SLX4IP is synthetic lethal with depletion of FANCM in U2OS ALT cancer cells. However, it is unclear if this effect depends on the unusual telomere structure of ALT cells or if it is due to general defects in DNA replication. Therefore, the analysis should also be carried out in non-ALT cells.

Other comments:
• Figure 1F,G: It would be informative to also include quantification of pSer33RPA foci outside of telomeres.
• Page 9, Expanded Fig. 1D: I doubt that it is justified to specify association with specific regions of the genome with 2 digits after the comma. The measurements are not precise enough. The numbers should be rounded.
• Page 3, line 46 : perhaps write that the combined loss of SLX4IP and FANCM shows synthetic lethality.

Revision Plan

Below is a detailed, point-by-point response to the concerns raised by the reviewers. All response comments are highlighted in red. Importantly, we consider all points addressable and experimentally feasible.

Referee #1 (Report for Author)

General Summary

In this manuscript, Panier and colleagues investigate the contribution of SLX4IP to the Alternative Lengthening of Telomeres (ALT) mechanism. Using a combination of fluorescence imaging, proteomics, and molecular analyses of DNA replication, they describe a connection between SLX4IP and FANCM in regulating BLM action within ALT processes. SLX4IP knockout cells exhibit a hyperactive ALT phenotype with accumulation of PML and replicative stress markers, including phospho-RPA2 and phospho-CHK1 at telomeres. Additionally, DNA fiber analysis reveals delayed replication due to SLX4IP loss. Mechanistically, these adverse effects are linked with deregulated BLM activity, whose levels accumulate globally but also at telomeres due to SLX4IP loss. The authors provide evidence that SLX4IP acts with FANCM against BLM, with depletion of FANCM leading to the death of SLX4IP-deficient cells. Notably, this cell death phenotype is partially reversed by co-depletion of BLM. Thus, a model is proposed envisioning that the role of the SLX4IP-FANCM axis is to limit BLM activity in ALT cancer cells.

This is a nice study, with some relevant insights into the phenotypic impact of SLX4IP loss on the ALT mechanism, with the connection to FANCM and BLM. A series of diverse approaches is employed to study SLX4IP (CUTnTAG, IPOND, DNA fibers). The finding of a synthetic lethal interaction between FANCM and SLX4IP is notable. However, on the negative side, the study suffers from going back and forth between telomeres and general aspects of DNA replication. While the general replication observations are nice, they seem to detract from the overall claim/goal of delineating how SLX4IP ultimately regulates ALT. It raises questions as to SLX4IP's role in ALT, considering the nearly identical distribution of the protein in U2OS and Hek cells as mapped by CUTnTAG. In its present state, it is difficult to interpret the results of the study as otherwise revealing that SLX4IP is a general replication factor with its absence having an as yet unexplained effect on BLM. We recommend that the authors provide additional evidence to support their claims.

Specific major concerns

1. Overall, the manuscript seeks further insights into the role of SLX4IP in the ALT mechanism. Yet, much of the presented data relates to a more general role for SLX4IP in DNA replication. It would be prudent to assess the impact of SLXIP loss on telomere replication and specifically as part of regulating the ALT mechanism. For instance, are the DNA replication defects occurring at telomeres during G2/M phase? Is the break-induced replication mechanism impacted by SLX4IP loss? Is SLX4IP actively recruited or displaced from telomeres actively

engaging in telomere extension? The contribution of SLX4IP to telomere regulation by ALT could be assessed more directly. This will strengthen the overall premise and message of the study.

We will further define the specific role of SLX4IP in regulating replication stress at ALT telomeres, with a particular focus on its function during G2/M phase and break-induced telomere replication (BIR); the required techniques are either already established in our lab or readily implementable. Specifically, we will:

- *Examine SLX4IP foci colocalization with EdU and telomeres to assess active telomere replication*
- *Analyze DNA synthesis at ALT-associated PML bodies in synchronized G2-phase cells (EdU/PML/telomere staining)*
- *Perform MiDAS (mitotic DNA synthesis) assays to test for unresolved telomere replication intermediates*
- *Use the TRF1-FokI system to induce targeted telomeric breaks and BIR activity in the presence and absence of SLX4IP*
- *Explore the genetic interaction between SLX4IP and key BIR factors such as RAD52 and POLD3 by evaluating replication stress phenotypes*
- *Quantify RAD52 foci in SLX4IP knockout cells as an indicator of BIR engagement*

These studies will directly test whether SLX4IP regulates BIR and will strengthen the central conclusion that it protects ALT telomeres during replication.

2. Figure 3 shows western blot analysis with a substantial increase in BLM protein levels in SLX4IP-deficient cells. What is the mechanism or molecular basis for this deregulation of BLM? Is this increase in BLM levels specific to SLX4IP-deficient ALT cancer cell lines (i.e. SLX4IP SLX4IP-deficient normal cells or cancer cells expressing telomerase? The authors appropriately cite their previous work showing that SLX4IP does not directly inhibit BLM in vitro, yet do not fully reconcile that point. This should be developed further.

We will further investigate the molecular basis of increased BLM protein levels in SLX4IP-deficient cells and clarify the nature of the SLX4IP-BLM relationship. Importantly, our preliminary data indicate that BLM upregulation is specific to ALT cells, as BLM levels remain unchanged in SLX4IP-deficient HeLa cells. This supports the idea that SLX4IP does not directly regulate BLM protein levels, but rather that BLM accumulation is a downstream consequence of deregulated ALT activity in the absence of SLX4IP. We hypothesize that hyperactivation of the ALT pathway induces BLM expression as part of a compensatory response.
To gain additional mechanistic insights into this point, we will:

- *Provide western blot data from SLX4IP-deficient non-ALT cells to demonstrate ALT-specificity of the BLM increase*
- *Analyze our existing SLX4IP Cut&Tag data to assess whether SLX4IP binds to the BLM promoter or enhancer regions*
- *Investigate potential changes in the BLM interactome in the presence or absence of SLX4IP using quantitative mass spectrometry with BLM as bait*

Together, these experiments will determine whether the observed increase in BLM reflects a direct, negative effect of SLX4IP on BLM transcripton or whether it involves changes in BLM regulation at the protein-complex level.

3. Figure 4 summarizes iPOND-MS experiments that show increased histone variants and immature/unligated Okazaki fragments at replication forks in SLX4IP-deficient cells.
Overall, this section of the study seems to be particularly preliminary and underdeveloped. Neither aspect is presented in a way directly connecting with ALT.

o First, can the author comment on the rationale for excluding the top hits from consideration for follow-up?
The top iPOND-MS hit was MSH6, which has been implicated in lagging strand processing. Initially, we did not pursue this further to avoid overinterpreting preliminary findings without mechanistic support. However, we recently obtained additional data that indeed confirm a potential connection between SLX4IP and the mismatch repair (MMR) pathway:

- *Immunofluorescence analysis shows increased chromatin-bound MSH6 in SLX4IP knockout U2OS cells*
- *Mass spectrometry and IP-WB identify interactions between SLX4IP and MMR components*
- *Clonogenic assays indicate that SLX4IP-deficient cells are more resistant to alkylating agents (TMZ, MNNG), consistent with altered MMR activity*

These findings suggest that SLX4IP may functionally intersect with MMR, which could contribute to the replication stress phenotypes observed. If we can corroborate these promising insights with additional experiments, we plan to incorporate them into the revised manuscript.

o Second, can the authors give a reason for why there are more histone variants present, considering the presumed accumulation of ssDNA, which is a sub-optimal substrate for nucleosome assembly, at these replication forks?

While the accumulation of ssDNA might seem incompatible with nucleosome assembly, recent studies have shown that chromatin at stressed replication forks is actively and dynamically reorganized to support fork protection and recovery. In particular work from the Taneja and Oberdoerffer labs demonstrates that histone variant deposition is part of an adaptive chromatin response to replication stress. MacroH2A, for example, has been shown to accumulate at fragile sites to help stabilize stalled forks (Kim et al., Mol cell 2018; Gaggioli et al., NCB 2023). As such, the increased presence of histone variants in our iPOND-MS data likely reflects a protective chromatin remodeling response, rather than classical nucleosome assembly on canonical duplex DNA.

o Third, the assessment of ADP-ribosylation in SLX4IP-deficient cells is interesting but again preliminary. The S1 sensitivity seems somewhat modest and should be further interrogated to strengthen the authors claims. In addition, as it relates to ALT, can ADP-ribose/PAR foci be detected at telomeres and if so, does this only occur in ALT cancer cell lines and outside of S-phase in G2-phase?

To address this point, we will:

- *Broaden our S1 nuclease sensitivity and PAR foci analysis to strengthen the link between SLX4IP loss, replication stress, and ADP-ribosylation.*
- *Investigate whether PAR foci localize to telomeres, and assess their cell cycle specificity by focusing on G2-phase cells.*
- *Determine whether such PAR foci are specific to ALT-positive cell lines, further clarifying their relevance to ALT biology.*

o Fourth, we note that the authors state that they observe increased SLX4IP on chromatin after treatment with Olaparib. Surely this would suggest a PAR-independent basis of SLX4IP recruitment and not that SLX4IP is recruited to manage replication-associated PARylation, as is stated on pg 13.

Our model is not that SLX4IP is directly recruited to sites of PARylation. Rather, we propose that SLX4IP acts upstream to limit replication stress that would otherwise trigger PARP activation. When PARP is inhibited by Olaparib and replication stress persists unresolved, SLX4IP accumulates (and persists) on chromatin, likely due to its role in managing stalled forks rather than being recruited by PAR itself. We will revise the relevant statements in the manuscript to reflect this more clearly.

4. A figure describing the final model or summary of findings should be provided in the final figure.

We will include a model in the final figure to clarify our findings and proposed mechanism.

5. Lastly, the conclusions are based almost exclusively on experiments performed with the U2OS cell line. While this line is widely used as a model for ALT, it is far from representative of all ALT cancers. To strengthen the findings as well as the key claim of potential clinical relevance, the authors should validate their most critical findings in at least one, preferably several, other ALT-positive cell lines.

We already have VA13 knockout cells in hand and are actively generating SLX4IP knockouts in SAOS2 and SKNFI backgrounds. This will allow us to validate key findings across multiple ALT models in the revised manuscript.

Minor Concerns

1. The abstract states "Despite its essential role in ALT, BLM must be tightly controlled....".

Surely, because of its essential role, it should be tightly controlled.

We will revise this to "Because of its essential role in ALT, BLM must be tightly controlled…"

2. Expanded View Figure 2A-B: missing error bars.

We will correct this and include error bars in the revised version.

3. At some points, figures are called either Expanded View or Supplementary Figures.

We will correct this in the revised version.

4. In Figure 4, why is ADP-ribose foci per nucleus size per cell used as quantification?

We normalized PAR foci to nuclear size to account for differences in nuclear area between cells. We will clarify this approach in the figure legend and methods section of the revised manuscript.

Referee #2 (Report for Author)

In this manuscript, the authors build upon a previous study to further investigate the role of SLX4IP at ALT telomeres. The authors demonstrate that SLX4IP depletion causes an induction of lagging strand specific telomere replication stress in ALT cells, that this replication stress is heightened by co-depletion of FANCM, that this synthetic lethal interaction involves BLM and can be rescued by BLM depletion, and that SLX4IP functions to restrain the activity of BLM in unwinding unligated Okazaki fragments. Overall, this is a well presented and clear manuscript. The conceptual advance over the previous study is a little limited, but the data are compelling.

Points to address:

• Are the effects of SLX4IP depletion upon replication stress telomere-specific and/or ALT-cell selective? This should be clarified throughout, as some experiments are very clearly focussed on the telomeres, while others (eg Cut & Tag, iPOND etc) are not.

We recognize that assays like Cut&Tag and iPOND inherently capture genome-wide interactions rather than telomere-specific events. This likely caused some of the confusion. To clarify this, we will expand our studies, particularly DNA combing, to non-ALT cell lines to determine whether the replication stress phenotypes are ALT telomere-specific or reflect a broader function of SLX4IP in replication stress.

• In Figure 1E - does the addition of HU significantly reduce the fork speed in the absence of SLX4IP, ie. are columns 2 vs 4 statistically significant?

We will expand the statistical analysis to clarify this. Overall, forks do slow down, but HU does not appear to overtly exacerbate the replication stress phenotype caused by SLX4IP loss. One possible explanation is that loss of SLX4IP already saturates the replication stress response in this context. We will expand on and carefully phrase this point in the revised manuscript.

• In Figure 1F - the pS33-RPA staining looks quite excessive, and it is difficult to see an increase in colocalizations. Clone 1 appears to have less colocalizations. Is the antibody specific and are there more representative images?

Clone 1 visibly has fewer colocalizations likely because telomeres in this clone cluster less tightly than in clone 2, resulting in more individual telomere foci. For this reason, we normalized the pS33-RPA/telomere colocalization by total telomere number. We will provide additional representative images to better illustrate the quantification.

• Colocalizations are difficult to discern in all IF/FISH images (for example Figure 3D). Arrows might help, but I appreciate this may get crowded in some images.

Colocalizations appear as white spots in our green/magenta overlays, which can indeed be challenging to see. We will add arrows in the revised manuscript to help guide readers and reviewers.

• I am unclear about the use and interpretation of Cut & Tag. The aim was to determine if SLX4IP was recruited to ALT telomeres during replication stress. The results in the Supp Figure 1E-G do not report peaks at telomeric regions vs non-telomeric regions, but rather show an increase in promoters, fragile sites, etc throughout the genome. How does this (i) address whether the localisation of SLX4IP is telomeric, and (ii) support the conclusion that "these results support the role of SLX4IP in mitigating replication stress at ALT telomeres"? It seems that SLX4IP is responding globally to replication stress and that this is happening independently of ALT activity. The result of this response specifically at telomeres needs further clarity, ie. - is SLX4IP playing a role specifically at ALT telomeres, or is it more genomic and the telomere specific effects and synthetic lethality are attributed to FANCM depletion. Is it possible to evaluate telomeric binding using Cut & Tag?

We did not include telomere analysis initially due to difficulties in peak calling in repetitive telomere regions. Using TTAGGG repeat counting, we detect SLX4IP enrichment at HEK cell telomeres but not reliably in U2OS cells, likely due to ALT-driven telomere repeat variant complexity. In the revised manuscript, we will include telomere data based on TTAGGG counting in HEK cells and are also currently working on reanalyzing the U2OS data using variant repeat-aware peak calling. Moreover, our Cut&Tag data clearly show SLX4IP enrichment at non-telomeric loci in both ALT and non-ALT cells, consistent with a broader role in replication stress. We will expand the DNA combing experiments to non-ALT cells to further address this.

• Consistent with this concept, it would be useful to examine whether the increase in fragile telomeres and decrease in fork speed after loss of SLX4IP are also seen in ALT-negative cells.

We will perform the telomere fragility assay in non-ALT cells, analogous to the work done in U2OS, to determine if these phenotypes extend beyond ALT cells.

• Do the levels of FANCM at ALT telomeres increase following loss of SLX4IP?

We will assess FANCM protein and mRNA levels in both ALT and non-ALT cell lines by Western blot and qPCR to clarify if FANCM is upregulated after SLX4IP depletion.

• A significant increase in PAR signal was observed following loss of SLX4IP, indicative of unligated Okazaki fragments. This was reversed with BLM depletion, suggesting that unrestrained BLM activity results in unwound and unligated Okazaki fragments. Is this genomic or telomere-specific? Could this be combined with telomere-FISH to demonstrate a telomeric effect?

We will repeat the assay and include telomere co-staining to determine whether the observed increase in PAR is telomere-specific or genome-wide. While PAR staining is not compatible with telomere FISH, we are currently optimizing the protocol to enable co-staining with Shelterin subunits as a proxy for telomere localization.

• Does FEN1 knockdown reverse the heightened replication stress and unligated Okazaki fragments caused by SLX4IP/FANCM loss to similar levels as BLM knockdown?

Our current data predict that SLX4IP acts either epistatically or in a parallel pathway to FEN1, suggesting FEN1 knockdown is unlikely to rescue the phenotype. In addition, we have obtained data showing that combined SLX4IP/FEN1 depletion in U2OS causes a moderate increase in pSer33-RPA signal but no overt synthetic lethality. We will present these data and extend analyses to non-ALT cells in the revised manuscript.

• Lines 123 and 199, FANCM binds to RMI1/2 rather than directly to BLM

We will correct this in the revised manuscript.

• Line 220/221 - "modest"? (word missing).

We will correct this in the revised manuscript.

Referee #3 (Report for Author)

Spindler et al. identify roles of SLX4IP in regulating the BLM helicase during DNA replication. The authors find in U2OS ALT cancer cells that SLX4IP KO clones have higher rates of telomere fragility and reduced replication fork speed throughout the genome. The replication stress marker pSer33RPA was increased at telomeres and pSer345CHK1 foci were also detected. HU-treatment led to SLX4IP accumulation at many loci in the genome as determined by cut&tag profiling indicating genome-wide roles of SLX4IP in response to replication stress. Depletion of the DNA translocase FANCM with SLX4IP-KO showed additive effects on replication stress at telomeres as well as synthetic growth defects. FANCM has been reported before to reduce replication stress at ALT telomeres. Published work also demonstrated that the reduced viability of FANCM-deficient ALT cells depends on BLM. It is reported here that the same is true for SLX4IP. Depletion of BLM in SLX4IP-deficient cells rescued replication stress as well as growth defects. Altogether, SLX4IP appears to prevent the toxic effects of excessive BLM activity. Determination of protein composition near replication forks by iPOND revealed among others enrichment of the flap endonuclease FEN1. Furthermore, by combining DNA combing analysis with S1 nuclease treatment it was found that single stranded gaps accumulated in the analyzed SLX4IP-KO clone. Poly(ADP) ribosylation also increased in the nucleus, which depended on BLM. Altogether the data suggest that loss of SLX4IP leads to defects in lagging strand synthesis throughout the genome by mis-regulation of BLM.

Major concern and comments:

• Clonal variation of telomere phenotypes is a common phenomenon which has been described in several publications. For example, telomere length varies substantially between clones of telomerase-positive cells. Clonal variation for other telomere phenotypes is also highly likely. Consistent with this notion, the two SLX4IP-KO clones show different levels of telomere fragility and marked differences in the other phenotypes described in the manuscript. For replication fork speed and single stranded gaps, only one clone is analyzed. I'm doubtful that the analysis of two or one clone is sufficient to exclude that the effects are not due to clonal variation. In order to achieve convincing results, the sample size of KO-clones must may be significantly increased or it must be demonstrated that expression of wild SLX4IP (e.g. from a cDNA) in the KO-background can rescue the phenotypes. Perhaps the latter experiment is less work.

We have additional SLX4IP knockout clones in U2OS cells, which we will analyze in the revised manuscript to address this concern. Furthermore, we have established SLX4IP knockouts in the ALT-positive cell lines VA13, and are in the process of generating more knockouts in SAOS2 and SKNFI cells. Parallel to these efforts, we are setting up a complementation system to re-express wild-type SLX4IP in knockout cells to confirm rescue of key phenotypes. Overall, these approaches will comprehensively exclude clonal artifacts.

• The results indicate that SLX4IP restricts BLM activity during replication not only at telomeres but throughout the genome. Although the project may have initially been aimed at investigating specific roles of SLX4IP at ALT telomeres the results indicate much broader roles of SLX4IP. Therefore, I recommend a considerable overhaul of the writing and emphasis of the paper. The title does also not reflect the general roles of SLX4IP in DNA replication which are discovered here. The paper might also reach a broader readership by addressing the general DNA replication community.

While the current manuscript focuses largely on data from U2OS cells, which justified emphasizing SLX4IP function at ALT telomeres, we fully agree that addressing its wider genome-wide role in managing replication stress will broaden the manuscript's impact. Therefore, we will substantially restructure the revised manuscript to place a central emphasis on the general replication stress function of SLX4IP.

• It is found that loss of SLX4IP is synthetic lethal with depletion of FANCM in U2OS ALT cancer cells. However, it is unclear if this effect depends on the unusual telomere structure of ALT cells or if it is due to general defects in DNA replication. Therefore, the analysis should also be carried out in non-ALT cells.

We are working on experiments to extend this analysis into non-ALT cell lines and will include these data in the revised manuscript to clarify whether this interaction is ALT-dependent or more general.

Other comments:

• Figure 1F,G: It would be informative to also include quantification of pSer33RPA foci outside of telomeres.

• Page 9, Expanded Fig. 1D: I doubt that it is justified to specify association with specific regions of the genome with 2 digits after the comma. The measurements are not precise enough. The numbers should be rounded.

• Page 3, line 46 : perhaps write that the combined loss of SLX4IP and FANCM shows synthetic lethality.

We will correct these points in the revised manuscript.

Dr. Stephanie Panier
Max Planck Institute for Biology of Ageing
Panier
Joseph-Stelzmann-Str 9b
Cologne 50931
Germany

1st Aug 2025

Re: EMBOJ-2025-121677
SLX4IP acts in parallel to FANCM to limit BLM-dependent replication stress at ALT telomeres

Dear Stephanie,

Thank you for your thoughtful revision plan for your recent EMBO Journal submission. I have now carefully gone through your detailed point-by-point responses, and I really appreciate your well-taken answers and serious consideration of the referees' concerns. I would therefore be happy to invite you to revise the study along the proposed lines, and to resubmit it to The EMBO Journal using the link below. I feel that in particular your plans to alter and re-focus the presentation of the findings, and the further clarification of ALT-specific vs. general replication-stress-linked roles should greatly improve the work; and I also appreciate the planned extension to other ALT (and non-ALT) cell lines and ruling out of possible clonal-variation artifacts, as well as the intended follow-up on the molecular mechanisms.

It is our policy to allow only a single round of (major) revision, so please keep me updated should there be any unexpected problems with the revisions, or should you require an extension beyond the default 3-months deadline. As always, competing manuscript published elsewhere during the course of this revision will not affect our eventual decision on your study. Finally, please note the detailed information and guidelines on how to prepare a revision below (and in our online Guide to Authors) - closely adhering to them shall greatly facilitate the editorial process at the time of resubmission.

Thank you again for the opportunity to consider this work, and I look forward to receiving your revision in due time.

With kind regards,

Hartmut

Hartmut Vodermaier, PhD
Senior Editor, The EMBO Journal
h.vodermaier@embojournal.org

*** PLEASE NOTE: All revised manuscript are subject to initial checks for completeness and adherence to our formatting guidelines. Revisions may be returned to the authors and delayed in their editorial re-evaluation if they fail to comply to the following requirements (see also our Guide to Authors for further information):

1) Every manuscript requires a Data Availability section (even if only stating that no deposited datasets are included). Primary datasets or computer code produced in the current study have to be deposited in appropriate public repositories prior to resubmission, and reviewer access details provided in case that public access is not yet allowed. Further information: embopress.org/page/journal/14602075/authorguide#dataavailability

2) Each figure legend must specify
- size of the scale bars that are mandatory for all micrograph panels
- the statistical test used to generate error bars and P-values
- the type error bars (e.g., S.E.M., S.D.)
- the number (n) and nature (biological or technical replicate) of independent experiments underlying each data point
- Figures may not include error bars for experiments with n<3; scatter plots showing individual data points should be used instead.

3) Revised manuscript text (including main tables, and figure legends for main and EV figures) has to be submitted as editable text file (e.g., .docx format). We encourage highlighting of changes (e.g., via text color) for the referees' reference.

4) Each main and each Expanded View (EV) figure should be uploaded as individual production-quality files (preferably in .eps, .tif, .jpg formats). For suggestions on figure preparation/layout, please refer to our Figure Preparation Guidelines: http://bit.ly/EMBOPressFigurePreparationGuideline

5) Point-by-point response letters should include the original referee comments in full together with your detailed responses to them (and to specific editor requests if applicable), and also be uploaded as editable (e.g., .docx) text files.

6) Please complete our Author Checklist, and make sure that information entered into the checklist is also reflected in the manuscript; the checklist will be available to readers as part of the Review Process File. A download link is found at the top of our Guide to Authors: embopress.org/page/journal/14602075/authorguide

7) All authors listed as (co-)corresponding need to deposit, in their respective author profiles in our submission system, a unique ORCiD identifier linked to their name. Please see our Guide to Authors for detailed instructions.

8) Please note that supplementary information at EMBO Press has been superseded by the 'Expanded View' for inclusion of additional figures, tables, movies or datasets; with up to five EV Figures being typeset and directly accessible in the HTML version of the article. For details and guidance, please refer to: embopress.org/page/journal/14602075/authorguide#expandedview

9) To facilitate reproducibilty and cross-laboratory adoption of methodologies, please structure the Materials & Methods section as outlined in our guide to authors, including a completed Reagents and Tools Table that can be downloaded from our author guidelines as well (https://www.embopress.org/page/journal/14602075/authorguide#structuredmethods).

10) Digital image enhancement is acceptable practice, as long as it accurately represents the original data and conforms to community standards. If a figure has been subjected to significant electronic manipulation, this must be clearly noted in the figure legend and/or the 'Materials and Methods' section. The editors reserve the right to request original versions of figures and the original images that were used to assemble the figure. Finally, we generally encourage uploading of numerical as well as gel/blot image source data; for details see: embopress.org/page/journal/14602075/authorguide#sourcedata

At EMBO Press we ask authors to provide source data for the main manuscript figures. You will receive a separate email with instructions for providing source data with your revised manuscript, including how to upload and organize the files.

Further information is available in our Guide For Authors: https://www.embopress.org/page/journal/14602075/authorguide

In the interest of ensuring the conceptual advance provided by the work, we recommend submitting a revision within 3 months (30th Oct 2025). Please discuss the revision progress ahead of this time with the editor if you require more time to complete the revisions.

REBUTTAL LETTER

Spindler, Pandolfo et al. : SLX4IP limits replication stress globally and at ALT telomeres

Referee #1 (Report for Author)

General Summary

In this manuscript, Panier and colleagues investigate the contribution of SLX4IP to the Alternative Lengthening of Telomeres (ALT) mechanism. Using a combination of fluorescence imaging, proteomics, and molecular analyses of DNA replication, they describe a connection between SLX4IP and FANCM in regulating BLM action within ALT processes. SLX4IP knockout cells exhibit a hyperactive ALT phenotype with accumulation of PML and replicative stress markers, including phospho-RPA2 and phospho-CHK1 at telomeres. Additionally, DNA fiber analysis reveals delayed replication due to SLX4IP loss. Mechanistically, these adverse effects are linked with deregulated BLM activity, whose levels accumulate globally but also at telomeres due to SLX4IP loss. The authors provide evidence that SLX4IP acts with FANCM against BLM, with depletion of FANCM leading to the death of SLX4IP-deficient cells. Notably, this cell death phenotype is partially reversed by co-depletion of BLM. Thus, a model is proposed envisioning that the role of the SLX4IP-FANCM axis is to limit BLM activity in ALT cancer cells.
This is a nice study, with some relevant insights into the phenotypic impact of SLX4IP loss on the ALT mechanism, with the connection to FANCM and BLM. A series of diverse approaches is employed to study SLX4IP (CUTnTAG, IPOND, DNA fibers). The finding of a synthetic lethal interaction between FANCM and SLX4IP is notable. However, on the negative side, the study suffers from going back and forth between telomeres and general aspects of DNA replication. While the general replication observations are nice, they seem to detract from the overall claim/goal of delineating how SLX4IP ultimately regulates ALT. It raises questions as to SLX4IP's role in ALT, considering the nearly identical distribution of the protein in U2OS and Hek cells as mapped by CUTnTAG. In its present state, it is difficult to interpret the results of the study as otherwise revealing that SLX4IP is a general replication factor with its absence having an as yet unexplained effect on BLM. We recommend that the authors provide additional evidence to support their claims.

We thank the reviewer for their careful evaluation of our manuscript and for their thoughtful and constructive comments. We particularly appreciate the recognition of the strengths of our manuscript. We have carefully considered the reviewer's concerns and substantially revised the manuscript to clarify the conceptual framework and improve the overall flow of the manuscript.

To address the reviewer's concern about the conceptual organization, we have restructured the presentation of the results to provide a clearer and more logical progression. The revised manuscript first establishes SLX4IP as a regulator of the replication stress response in a general context (Figures 1–3), defining its role in limiting replication-associated stress and its functional relationship with BLM in this context. Building on this, we then

demonstrate that ALT telomeres constitute a particularly vulnerable genomic environment in which this SLX4IP-dependent replication stress-limiting function becomes critical (Figure 4). Finally, we position SLX4IP within a pathway parallel to FANCM (Figure 5) and reveal that the observed synthetic lethality with FANCM arises from deregulated BLM activity (Figure 6).

*In line with this refocusing, we have revised the title to: "***SLX4IP limits replication stress globally and at ALT telomeres***."*

Importantly, although SLX4IP contributes to replication stress control in multiple cellular contexts, our data indicate that SLX4IP does not function as a core replication factor. Rather, the results support a model in which SLX4IP acts as a modulator of BLM-dependent replication-associated processes. Loss of SLX4IP leads to pathological BLM-dependent replication intermediates, resulting in replication stress, ssDNA gap accumulation, and checkpoint activation. As such, ALT telomeres emerge not as the exclusive site of SLX4IP action, but as a genomic region that is intrinsically sensitive to perturbations in this regulatory axis. The pronounced ALT-associated phenotypes are therefore best explained by the elevated baseline replication stress at these loci, which renders ALT telomeres particularly vulnerable to deregulated BLM activity. This interpretation reconciles the genome-wide replication phenotypes with the telomere-specific defects and provides a unified conceptual model for SLX4IP function.

Below, we provide a point-by-point response to the reviewer's specific concerns.

Specific major concerns

1. Overall, the manuscript seeks further insights into the role of SLX4IP in the ALT mechanism. Yet, much of the presented data relates to a more general role for SLX4IP in DNA replication. It would be prudent to assess the impact of SLXIP loss on telomere replication and specifically as part of regulating the ALT mechanism. For instance, are the DNA replication defects occurring at telomeres during G2/M phase? Is the break-induced replication mechanism impacted by SLX4IP loss? Is SLX4IP actively recruited or displaced from telomeres actively engaging in telomere extension? The contribution of SLX4IP to telomere regulation by ALT could be assessed more directly. This will strengthen the overall premise and message of the study.

We thank the reviewer for this insightful and constructive comment. We fully agree that clarifying how SLX4IP links replication stress to ALT-associated telomere regulation is essential for strengthening the central message of our manuscript. In the revised manuscript, we have thus restructured and expanded key sections to present this relationship more clearly.

To assess whether SLX4IP directly impacts telomere replication or BIR, we examined its localization at actively replicating telomeres and analyzed the consequences of its depletion for telomeric DNA synthesis. We now present data showing that approximately 30% of EdU-positive telomeres colocalize with SLX4IP in G2-enriched ALT cells (Expanded View 4J-K). However, despite elevated replication stress and increased APB formation in the absence of SLX4IP, we observe no corresponding increase in telomeric EdU incorporation, either in G2-enriched cells or

following TRF1-FokI–induced breaks (Expanded View 4R-U). Furthermore, co-depletion of SLX4IP with RAD52 or POLD3 (key factors in BIR) further enhances APB formation (Expanded View 4V).

We interpret these observations as evidence that SLX4IP is not directly required for BIR initiation or telomeric DNA synthesis. Instead, our data support a model in which SLX4IP acts upstream by limiting replication stress and maintaining ongoing replication at telomeric forks. In its absence, replication stress leads to accumulation of APBs and recombination-associated structures (Panier et al., Mol Cell 2019), yet telomeric DNA synthesis remains inefficient or abortive. Importantly, this is consistent with replication forks being stalled but structurally intact, rather than collapsed. This interpretation is further supported by our expanded U2OS iPOND data (Figure 2C and E, Expanded View 2H), which show that SLX4IP-deficient forks in U2OS cells are enriched for MCMs, RFCs, POLD1/3, FEN1, Ligase 1, TOP1/2A, histones, and chromatin remodelers. These findings indicate that the replication machinery is still mostly assembled at stalled forks, consistent with a state that would be refractory to productive BIR.

In sum, the data presented in our revised manuscript support a model in which SLX4IP acts upstream to preserve telomeric replication. Loss of SLX4IP elevates replication stress at stalled forks, leading to APB accumulation and other recombination-associated markers at ALT telomeres. These outcomes likely reflect the consequences of unresolved replication stress rather than a direct role for SLX4IP in BIR-mediated telomere extension.

2. Figure 3 shows western blot analysis with a substantial increase in BLM protein levels in SLX4IP-deficient cells. What is the mechanism or molecular basis for this deregulation of BLM? Is this increase in BLM levels specific to SLX4IP-deficient ALT cancer cell lines (i.e. SLX4IP SLX4IP-deficient normal cells or cancer cells expressing telomerase? The authors appropriately cite their previous work showing that SLX4IP does not directly inhibit BLM in vitro, yet do not fully reconcile that point. This should be developed further.

We thank the reviewer for this important comment. In the revised manuscript, we have further examined the regulation of BLM levels following SLX4IP loss across multiple cellular contexts.

Our new data indicate that BLM upregulation is not a universal consequence of SLX4IP deficiency. While we observe a substantial increase in BLM protein levels in U2OS cells, BLM levels do not consistently change in SLX4IP-deficient SAOS-2, RPE1-hTert, and eHAP cells (Expanded View 6F-I). These findings argue against a model in which SLX4IP directly regulates BLM expression. Instead, they support a context-dependent mechanism of how BLM levels change in the absence of SLX4IP.

Importantly, the differential BLM response across cell lines does provide new mechanistic insight: If SLX4IP directly controlled BLM expression, comparable effects would be expected across cellular backgrounds. The selective BLM upregulation observed in U2OS cells therefore supports an indirect model in which increased BLM levels reflect downstream consequences of SLX4IP loss. In U2OS cells, SLX4IP deficiency results in pronounced replication stress and recombination-associated phenotypes. Under these conditions, elevated BLM levels are consistent with a compensatory response to altered replication dynamics rather than direct transcriptional regulation by SLX4IP.

The variability observed between ALT-positive cell lines (U2OS and SAOS-2) is likewise consistent with emerging views of ALT as a heterogeneous process rather than a uniform pathway (Cesare et al, 2009; Henson et al, Nat Biotech 2009; Loe et al, Genes Dev 2020). Replication stress responses, recombination dynamics, and reliance on specific DNA processing activities differ substantially across ALT models. The distinct effects on BLM levels observed here indeed align with this heterogeneity.

We fully agree with the reviewer that defining the molecular basis of SLX4IP-dependent BLM regulation represents an important mechanistic question. Our current and previous findings argue against either direct transcriptional regulation of BLM expression or direct modulation of BLM enzymatic activity by SLX4IP (Panier et al., Mol Cell 2019). An alternative possibility is that SLX4IP, which lacks enzymatic activity and likely functions as a scaffold protein, influences BLM regulation by modulating BLM's interactions with partner proteins in specific cellular and genomic contexts. Addressing this question will require comprehensive and quantitative analysis of SLX4IP-dependent changes in BLM-associated protein networks. Given the scope and complexity of such studies, particularly across distinct ALT and non-ALT systems, we consider this an important direction for future investigation. We now explicitly discuss this mechanistic possibility in the revised discussion (lines 414ff)).

3. Figure 4 summarizes iPOND-MS experiments that show increased histone variants and immature/unligated Okazaki fragments at replication forks in SLX4IP-deficient cells. Overall, this section of the study seems to be particularly preliminary and underdeveloped. Neither aspect is presented in a way directly connecting with ALT.

o First, can the author comment on the rationale for excluding the top hits from consideration for follow-up?

We agree with the reviewer that the initial presentation of the iPOND-MS experiments required further clarification and development. In the revised manuscript, we have substantially expanded and strengthened this dataset.

Regarding the reviewer's question concerning the exclusion of top hits, we apologize for the lack of clarity in the original presentation. In the initial dataset, MSH6 emerged as the top-ranked hit. At the time, we chose not to emphasize this observation to avoid overinterpreting a single prominent factor without sufficient mechanistic support. To minimize potential confusion, MSH6 was therefore not highlighted in the graphical summary. We agree that this decision warranted clearer explanation. In the revised manuscript, we now transparently label both top- and bottom-ranked hits in the rank plot summaries. We have also included MSH6 alterations in SLX4IP-deficient cells as a point of discussion and future inquiry.

Importantly, the iPOND-MS analysis has now been significantly expanded. The revised dataset includes experiments performed in U2OS, RPE1-hTert, and eHAP cells, with two independent SLX4IP-deficient clones analyzed per cell line. These experiments are further supported by corresponding DNA combing analyses in these clones and cell lines, providing orthogonal validation of the replication phenotypes across cell lines. Collectively, this expanded dataset is considerably more comprehensive and robust than in the original manuscript.

Across both U2OS and RPE1-hTert cells, the iPOND-MS profiles consistently reveal enrichment of chromatin remodelers, histones, replisome-associated components, and multiple replication stress–associated factors (Figure

2C-E, Expanded View 2H-I)). Mismatch repair factors are also reproducibly enriched, particularly in U2OS cells. Rather than reflecting isolated protein changes, these patterns represent coherent replication stress signatures consistent with replication fork slowing. SLX4IP-deficient eHAP cells likewise display strong replication stress signatures. Here, the enrichment patterns differ, suggesting a relative shift toward fork collapse rather than predominant fork slowing or stalling (Expanded View 2J-L). While chromatin remodellers and certain replisome components remain enriched, we observe a broader representation of replication stress and DNA damage response factors, likely exacerbated by SLFN11 expression in eHAPs which drives RPA-dependent MCM unloading and aggressive checkpoint activation. Consistently, histones and MCMs are depleted from replication forks in this cell line. Although the magnitude and composition of specific enriched factors vary between cell lines, the dominant and unifying feature of the dataset is the consistent emergence of replication stress-associated signatures following SLX4IP loss.

To further improve interpretability and address space limitations inherent to rank plot labeling, we now additionally provide a comparative visualization of chromatin-associated factors, histones, replisome components, replication stress markers, and mismatch repair proteins. This analysis highlights common replication stress signatures across independent clones and cell lines to emphasize the reproducibility and coherence of the dataset.

o Second, can the authors give a reason for why there are more histone variants present, considering the presumed accumulation of ssDNA, which is a sub-optimal substrate for nucleosome assembly, at these replication forks?

We thank the reviewer for this insightful comment. While ssDNA accumulation may appear incompatible with nucleosome assembly, increasing evidence indicates that chromatin at stressed replication forks is actively and dynamically reorganized rather than depleted. Indeed, replication stress induces adaptive chromatin remodelling responses that are mechanistically distinct from conventional nucleosome assembly. Histone variant deposition, including macroH2A family members and linker histone H1 variants, has been directly linked to fork protection and genome stability under replication stress conditions (Kim et al., Mol Cell 2018; Gaggioli et al., Nat Cell Biol 2023; Ozgencil et al., Life Sci Alliance 2023, Feng et al., PNAS 2019).

Importantly, the apparent discrepancy between increased histone association in iPOND-MS and ssDNA gaps detected by the S1-modified DNA fiber assay reflects the distinct nature of these measurements. iPOND captures proteins associated with EdU-labeled nascent DNA, which predominantly represents replicated duplex DNA, whereas the S1 assay detects post-replicative ssDNA gaps between completed DNA segments. Thus, histone association detected by iPOND can readily coexist with ssDNA gaps that are invisible to iPOND but detectable by S1 nuclease treatment.

In this context, the increased presence of histones and histone variants in our iPOND-MS dataset is unlikely to reflect canonical nucleosome assembly on ssDNA. Rather, it is consistent with replication stress-induced chromatin remodelling that is associated with perturbed fork progression and fork protection mechanisms. We have clarified this interpretation in the revised manuscript (line 457ff).

o Third, the assessment of ADP-ribosylation in SLX4IP-deficient cells is interesting but again preliminary. The S1

sensitivity seems somewhat modest and should be further interrogated to strengthen the authors claims. In addition, as it relates to ALT, can ADP-ribose/PAR foci be detected at telomeres and if so, does this only occur in ALT cancer cell lines and outside of S-phase in G2-phase?

We thank the reviewer for these important suggestions, which have significant implications for clarifying the mechanistic role of SLX4IP at stressed replication forks in the revised manuscript.

To strengthen the S1 nuclease analysis, we repeated the experiment using a modified labeling protocol designed to improve assay sensitivity. Specifically, we slightly increased the duration of the second pulse to maximize the length of nascent DNA susceptible to S1 nuclease cleavage. Under these conditions, we now observe a clear and reproducible shortening of DNA fibers in two independent U2OS SLX4IP knockout clones (Figure 3A), supporting the conclusion that SLX4IP loss leads to increased post-replicative ssDNA gaps. We also included a more detailed explanation of this data in the discussion that may suggest another importance for SLX4IP in termination (to be followed up in future studies) (lines 408ff).

Consistent with a replication stress-associated phenotype, we also detect increased ADPr foci in SLX4IP-deficient RPE1-hTert cells, whereas this phenotype is less pronounced in eHAP cells (Expanded View 3D-G). This pattern mirrors the distinct replication stress signatures observed in our iPOND-MS datasets and indicates that the global increase in ADPr is not restricted to ALT-positive cells. With respect to the reviewer's question regarding telomeric ADPr, we do not detect a specific enrichment of ADPr foci at telomeres in the absence of SLX4IP in U2OS cells, at least at the resolution level of our antibody (Expanded View 4H-I). However, ALT telomeric regions may still be vulnerable to fork destabilization based on the increase in ATR signalling at telomeres and consistent with SLX4IP's broader role in fork protection.

Based on these new findings, we now conclude in the revised manuscript that SLX4IP primarily functions as a regulator of replication fork stability, most likely by limiting ssDNA gap formation and facilitating proper processing of replication intermediates, rather than by directly controlling DNA synthesis, either at the leading or the lagging strand. Accordingly, the phenotypes observed following SLX4IP loss are best interpreted as consequences of replication stress, rather than defects in DNA synthesis itself.

This distinction is particularly relevant in the context of ALT telomeres. While telomeric DNA is inherently challenging to replicate, ALT telomeres exhibit additional features that further exacerbate replication difficulties such as elevated levels of secondary DNA structures and a highly altered chromatin organization. In this setting, factors that preserve replication fork stability become particularly critical. A genome-wide increase in replication stress following SLX4IP loss is therefore expected to have disproportionately severe consequences at ALT telomeres, where replication challenges are intrinsically heightened.

Consistent with this, we observe robust accumulation of replication stress markers at telomeres despite the absence of detectable telomere-specific enrichment of ADP ribosylation (Figure 4C-F). Importantly, SLX4IP deficiency does not lead to increased EdU-positive telomeres or elevated BIR-associated DNA synthesis (Expanded View 4R-U). Instead, the data indicate that telomeres engage recombination pathways but fail to efficiently move forward with DNA synthesis.

We propose that SLX4IP contributes to maintaining replication forks competent for productive downstream BIR at ALT telomeres. In the absence of SLX4IP, elevated ssDNA gaps and replication stress promote recombination attempts. However, these events appear inefficient or abortive, resulting in the accumulation of recombination intermediates and APBs without a corresponding increase in productive DNA synthesis.

We discuss this interpretation in the revised manuscript (lines 457ff).

o Fourth, we note that the authors state that they observe increased SLX4IP on chromatin after treatment with Olaparib. Surely this would suggest a PAR-independent basis of SLX4IP recruitment and not that SLX4IP is recruited to manage replication-associated PARylation, as is stated on pg 13.

Our model is not that SLX4IP is directly recruited to sites of PARylation. Rather, we propose that SLX4IP acts upstream to limit replication stress that would otherwise trigger PARP activation. When PARP is inhibited by Olaparib and replication stress persists unresolved, SLX4IP accumulates on chromatin, likely due to its role in managing stalled forks rather than being recruited by PAR itself. We have revised the relevant statements in the manuscript to reflect this more clearly (line 256f).

4. A figure describing the final model or summary of findings should be provided in the final figure.

We now present a model in revised Figure 7.

5. Lastly, the conclusions are based almost exclusively on experiments performed with the U2OS cell line. While this line is widely used as a model for ALT, it is far from representative of all ALT cancers. To strengthen the findings as well as the key claim of potential clinical relevance, the authors should validate their most critical findings in at least one, preferably several, other ALT-positive cell lines.

In the revised manuscript, we have expanded our analyses to additional ALT-positive cell lines. Specifically, we repeated critical experiments in WI38-VA13 and SAOS-2 cells. Consistent with our observations in U2OS cells, SLX4IP deficiency in WI38-VA13 cells results in a pronounced increase in pS33-RPA at telomeres, indicating elevated replication stress at ALT telomeres (Expanded View 4B-E)). Furthermore, we observe a clear augmentation of this phenotype upon FANCM depletion, which is accompanied by a synthetic growth defect (Expanded View 5M-N). Importantly, we also observed this growth defect in SAOS-2 cells, albeit at milder levels (Expanded View 5P-Q). In contrast, these effects are not detected in RPE1-hTert cells, supporting the specificity of this genetic interaction within ALT-associated cellular contexts (Expanded View 5R-S)).

While the magnitude of the growth defect varies between ALT cell lines, the phenotypes are reproducible and directionally consistent. Such variability is well aligned with the established heterogeneity of ALT mechanisms across different cellular backgrounds (Cesare et al, 2009; Henson et al, Nat Biotech 2009; Loe et al, Genes Dev 2020). Crucially, the conserved nature of the SLX4IP-FANCM interaction across multiple ALT models reinforces the generality of our conclusions rather than limiting them to U2OS cells.

In the revised manuscript, we have updated the discussion to clarify that although quantitative responses may differ across ALT systems, the mechanistic relationship between SLX4IP loss, replication stress, and FANCM dependency is preserved (lines 491ff).

Minor Concerns

1. The abstract states "Despite its essential role in ALT, BLM must be tightly controlled....". Surely, because of its essential role, it should be tightly controlled.

We have revised this in the introduction (line 122).

2. Expanded View Figure 2A-B: missing error bars.

The Old Expanded View Figure 2A–B (Expanded View 5C-D in the revised manuscript) shows data from a single representative experiment, which is why error bars are not displayed. Knockdown efficiency was experimentally validated in the initial experiment using qPCR. For subsequent biological replicates, knockdown efficiency was not re-assessed by qPCR, as identical siRNA reagents and experimental conditions were used. Importantly, these independent experiments reproducibly yielded the same phenotypic outcomes presented in Figure 5AB, consistent with effective FANCM depletion. To clarify this point, we have revised the figure legend to indicate that the panels represent a representative experiment.

3. At some points, figures are called either Expanded View or Supplementary Figures.

We have corrected this mistake in the revised manuscript.

4. In Figure 4, why is ADP-ribose foci per nucleus size per cell used as quantification?

We normalized ADPr foci to nuclear size to account for differences in nuclear area between cells. To avoid confusion, we have re-labelled the y-axis on the graphs to "number of foci/ cell (normalized to nuclear area)". In addition, have added the following sentence to the methods section of the revised manuscript:" To correct foci numbers to the nuclear size, the number of foci of each nucleus was divided by the respective nucleus area." (line 706f).

Referee #2 (Report for Author)

In this manuscript, the authors build upon a previous study to further investigate the role of SLX4IP at ALT telomeres. The authors demonstrate that SLX4IP depletion causes an induction of lagging strand specific telomere replication stress in ALT cells, that this replication stress is heightened by co-depletion of FANCM, that this synthetic lethal interaction involves BLM and can be rescued by BLM depletion, and that SLX4IP functions to restrain the activity of BLM in unwinding unligated Okazaki fragments. Overall, this is a well presented and clear manuscript. The conceptual advance over the previous study is a little limited, but the data are compelling.

We thank the reviewer for the positive evaluation of our manuscript. We appreciate the comment regarding the conceptual advance and agree that further clarifying this aspect is important.

While SLX4IP has previously been characterized primarily in the context of ALT as telomere factor (Panier et al., Mol Cell 2019, Robinson et al. Sci Signal 2021, Mangosh et al., Life Sci Alliance 2020, Mangosh et al., Molecular Cancer Research 2021), its physiological function has remained largely undefined. A central conceptual advance of this revised manuscript is the identification of SLX4IP as a regulator of the cellular response to replication stress. In the manuscript, we establish this function across a spectrum of ALT-positive and ALT-negative cell lines, demonstrating that the role of SLX4IP in replication stress regulation is not restricted to ALT-positive cells but represents a broader physiological function.

Building on this, we then show that ALT telomeres are particularly sensitive to SLX4IP loss. This heightened vulnerability is consistent with the intrinsic replication challenges of ALT telomeres, which are characterized by elevated replication stress and recombination engagement. Thus, rather than defining SLX4IP solely as an ALT-specific factor, our findings position SLX4IP as a general replication stress regulator whose loss causes highly amplified consequences at ALT telomeres.

Importantly, our data further reveal that the genetic interaction between SLX4IP and FANCM is specific to ALT cells. This finding suggests that ALT telomeres exhibit a unique dependency on the SLX4IP-FANCM axis to manage replication-associated stress. We propose that this synthetic lethal interaction reflects the exceptional replication burden characteristic of ALT telomeres. This observation has potential translational relevance given emerging strategies to target FANCM in ALT-positive cancers.

We have revised the manuscript to more clearly emphasize these conceptual advances.

Below, we provide a point-by-point response to the reviewer's specific concerns.

Points to address:

• Are the effects of SLX4IP depletion upon replication stress telomere-specific and/or ALT-cell selective? This

should be clarified throughout, as some experiments are very clearly focussed on the telomeres, while others (eg Cut & Tag, iPOND etc) are not.

We thank the reviewer for this important comment and agree that distinguishing between telomere-specific and genome-wide effects of SLX4IP depletion is essential for interpreting our data. We recognize that assays such as Cut&Tag and iPOND inherently capture only genome-wide phenotypes, which may have contributed to ambiguity in the original manuscript.

To directly address this issue, we have expanded our DNA combing analyses across ALT-positive (U2OS) and ALT-negative cell lines (RPE1-hTert and eHAP). These experiments reveal pronounced replication fork slowing following SLX4IP loss in multiple cellular backgrounds (Figure 2CD, Expanded View 2HI), indicating that replication stress represents a general consequence of SLX4IP deficiency rather than an ALT-specific phenotype.

Consistent with these functional observations, we substantially expanded the iPOND-MS dataset, which now includes U2OS, RPE1-hTert, and eHAP cells, with two independent SLX4IP-deficient clones analyzed per cell line. Across both U2OS and RPE1-hTert cells, the iPOND-MS profiles consistently reveal enrichment of chromatin remodelers, histones, replisome-associated components, and multiple replication stress–associated factors (Figure 2EF). Notably, mismatch repair factors are reproducibly enriched, particularly in U2OS cells. Rather than reflecting isolated protein changes, these patterns represent coherent replication stress signatures consistent with replication fork stalling. SLX4IP-deficient eHAP cells likewise display strong replication stress signatures. Here, the enrichment patterns differ, suggesting a relative shift toward fork collapse rather than predominant fork stalling (Expanded View 2J-L). While chromatin remodellers and certain replisome components remain enriched, we observe a broader representation of replication stress and DNA damage response factors. Notably, histones and MCM are depleted from replication forks in this cell line. Although the magnitude and composition of specific enriched factors vary between cell lines, the dominant and unifying feature of the dataset is the consistent emergence of replication stress-associated signatures following SLX4IP loss.

Finally, we further expanded the ADP ribosylation analysis to include RPE1-hTert and eHAP cells (Expanded View 2DG). SLX4IP-deficient RPE1-hTert cells exhibit increased ADP ribosylation, consistent with the phenotype observed in U2OS cells. This effect is not observed in eHAP cells, which aligns with the distinct replication fork composition detected in the iPOND analysis.

Collectively, these data, along with the Cut&Tag data, indicate that replication stress induced by SLX4IP depletion is not ALT-specific but reflects a broader physiological function of SLX4IP. ALT telomeres, owing to their intrinsic replication challenges, represent particularly vulnerable genomic regions where the consequences of SLX4IP loss become strongly manifested. We have revised the manuscript to clarify this distinction throughout.

• In Figure 1E - does the addition of HU significantly reduce the fork speed in the absence of SLX4IP, ie. are columns 2 vs 4 statistically significant?

We expanded the statistical analysis of old Figure 1E (revised Figure 2G) and find that HU induces a modest but statistically significant additional reduction in fork speed in SLX4IP-deficient cells, although the effect is not large.

We now also included treatment with cisplatin and observed a similar pattern. We interpret this modest enhancement as likely reflecting that SLX4IP loss already imposes substantial replication stress, which might limit the relative impact of further exogenous stress.

• In Figure 1F - the pS33-RPA staining looks quite excessive, and it is difficult to see an increase in colocalizations. Clone 1 appears to have less colocalizations. Is the antibody specific and are there more representative images?

The apparent difference in pS33-RPA/telomere colocalizations between the clones primarily reflects differences in telomere clustering rather than antibody performance. Specifically, the clone highlighted by the reviewer exhibits more pronounced telomere clustering, which reduces the number of visually separable telomere foci. This difference can create the impression of fewer colocalization events at the image level. To account for this, we normalized pS33-RPA/telomere colocalizations to the total number of telomere foci. This approach corrects for clone-specific differences in telomere clustering and provides a more accurate comparison of replication stress signaling at telomeres. To improve clarity, we have now included additional representative images (Expanded View 4A) and added arrows to guide the reader in the main text figures (Figure 4CE, Figure 5ACE, Figure 6AC).

In addition, we identified and corrected an inconsistency in clone nomenclature in this figure. The clone referenced by the reviewer corresponds to the previously labeled clone 1, which is now correctly designated as clone 2 in the revised manuscript.

• Colocalizations are difficult to discern in all IF/FISH images (for example Figure 3D). Arrows might help, but I appreciate this may get crowded in some images.

We agree that colocalizations in some microscopy images can be difficult to discern, as noted by the reviewer for example in old Figure 3D (revised Figure 6C). We typically use green/magenta overlays to distinguish the two channels and make colocalizations appear as white spots. This color scheme is generally more accessible than standard red/green overlays for readers with red-green color blindness. To further aid interpretation, we have added arrows to revised Figure 4CE, Figure 5ACE and Figure 6AC to help guide readers.

• I am unclear about the use and interpretation of Cut & Tag. The aim was to determine if SLX4IP was recruited to ALT telomeres during replication stress. The results in the Supp Figure 1E-G do not report peaks at telomeric regions vs non-telomeric regions, but rather show an increase in promoters, fragile sites, etc throughout the genome. How does this (i) address whether the localisation of SLX4IP is telomeric, and (ii) support the conclusion that "these results support the role of SLX4IP in mitigating replication stress at ALT telomeres"? It seems that SLX4IP is responding globally to replication stress and that this is happening independently of ALT activity. The result of this response specifically at telomeres needs further clarity, ie. - is SLX4IP playing a role specifically at ALT telomeres, or is it more genomic and the telomere specific effects and synthetic lethality are attributed to FANCM depletion. Is it possible to evaluate telomeric binding using Cut & Tag?

We thank the reviewer for this comment and agree that the interpretation of the CUT&Tag experiments required further clarification.

We first note that conventional peak calling is inherently limited at telomeres due to their highly repetitive nature and the short-read design of CUT&Tag. Standard alignment and peak-calling algorithms cannot reliably resolve TTAGGG repeat regions, which is why we did not include telomeric peaks in the original analysis.

To directly address this limitation, we added a repeat-based analysis strategy in the revised manuscript where we quantified telomeric enrichment by regex-based counting of TTAGGG repeats from the raw sequencing reads (Thorn et al., Nat Comm 2022; https://github.com/dariober/bioinformatics-cafe/tree/master/fastaRegexFinder). This approach allows unbiased detection of telomeric sequences independent of alignment constraints. Using this method, we detect clear enrichment of TTAGGG-containing reads in U2OS cells (Expanded View 1A). Although TTAGGG motif reads were less abundant than reads without the TTAGGG sequence (hence the negative log2), overall, their proportional representation was increased relative to that of CCCTC motifs, which correspond to CTCF-binding sites, indicating preferential recovery of telomeric sequences. Importantly, we validated this analysis using published Cut&Tag / ChIP-seq datasets from U2OS cells (H3K9me3 and pS57-H3) as negative controls, confirming that repeat-based quantification specifically detects telomeric signals only when present in the raw sequencing reads.

Notably, we did not observe enrichment of TTAGGG relative to CCCTC in unchallenged HEK293 cells, indicating that less recruitment to telomeres in this cell line (Expanded View 1A). However, following HU treatment, TTAGGG reads increased in both U2OS and HEK293 datasets (Expanded View 1B). This behaviour is consistent with our imaging data (Panier et al., Mol Cell 2019) and supports a model in which SLX4IP recruitment to telomeres is replication stress-dependent. Because ALT telomeres experience elevated endogenous replication stress even in untreated conditions, SLX4IP telomeric enrichment is readily detectable in U2OS cells but minimal in HEK293 cells.

Importantly, the CUT&Tag datasets also reveal widespread SLX4IP enrichment at non-telomeric loci in both ALT and non-ALT cells. These genome-wide patterns are fully consistent with a broader role of SLX4IP in replication stress responses rather than a strictly telomere-restricted function. To further explore this genome-wide association from a replication-point of view, we re-analyzed the CUT&Tag dataset for replication-associated features and observe significant SLX4IP enrichment near replication origins genome-wide, particularly following HU treatment (Figure 1EF). This enrichment provides independent support for a role of SLX4IP at stressed replication forks. Replication stress induced by HU is known to alter origin-proximal chromatin, fork dynamics and origin firing (for example Gaggioli et al., Nat Cell Biol 2023; Ercilla et al., Cell Mol Life Sci 2019, Feng et al., PNAS 2019), and SLX4IP accumulation at these regions is therefore consistent with its proposed function in replication fork stability and processing rather than sequence-specific targeting.

Collectively, the revised analyses of the CUT&Tag data clarify that SLX4IP exhibits both genome-wide and telomere-associated recruitment, with telomeric localisation emerging preferentially under conditions of replication stress and ALT telomeres. ALT telomeres therefore represent a context in which SLX4IP-dependent replication functions become especially evident rather than a site of exclusive SLX4IP activity.

To improve conceptual clarity and logical flow, we have reorganized the manuscript accordingly. The revised structure now first establishes the genome-wide role of SLX4IP in replication stress (Revised Figure 1-3), followed by focused analyses of ALT telomeric phenotypes (Revised Figure 4-6).

• Consistent with this concept, it would be useful to examine whether the increase in fragile telomeres and decrease in fork speed after loss of SLX4IP are also seen in ALT-negative cells.

We thank the reviewer for this important suggestion. To directly address this point, we expanded our DNA combing analyses to include both ALT-positive (U2OS) and ALT-negative cell lines (RPE1-hTert and eHAP). These experiments reveal pronounced replication fork slowing following SLX4IP loss across multiple cellular backgrounds, demonstrating that replication stress represents a general consequence of SLX4IP deficiency rather than an ALT-specific phenotype (Figure 2AB, Expanded View 2F). In contrast, we do not observe a statistically significant increase in telomere fragility in ALT-negative RPE1-hTert cells following SLX4IP depletion (Expanded View 4FG). This result is consistent with the distinct replication environments of ALT-negative versus ALT telomeres. ALT-negative telomeres are typically more stable and experience lower basal replication stress, which likely allows these cells to buffer or compensate for the increased replication stress induced by SLX4IP loss.

Our data therefore support a model in which SLX4IP functions broadly in replication fork stability, while telomeric consequences become particularly evident in ALT cells, where telomeres are intrinsically exposed to elevated replication stress.

• Do the levels of FANCM at ALT telomeres increase following loss of SLX4IP?

To address whether FANCM levels change following SLX4IP loss, we examined both FANCM protein and mRNA levels in U2OS cells. We do not detect a significant alteration in FANCM abundance at either the protein or transcript level upon SLX4IP depletion (Expanded View 5AB). These observations indicate that the functional interaction between SLX4IP and FANCM is unlikely to arise from compensatory changes in FANCM expression.

• A significant increase in PAR signal was observed following loss of SLX4IP, indicative of unligated Okazaki fragments. This was reversed with BLM depletion, suggesting that unrestrained BLM activity results in unwound and unligated Okazaki fragments. Is this genomic or telomere-specific? Could this be combined with telomere-FISH to demonstrate a telomeric effect?

We thank the reviewer for this insightful comment. To determine whether the increased ADPr signal reflects a genomic or telomere-specific effect, we performed combined ADPr and telomere-FISH analyses. These experiments revealed that ADPr foci are predominantly global and are not specifically enriched at telomeres upon SLX4IP loss (Expanded View 4H–I). This indicates that the elevated ADPr signal primarily reflects a genome-wide replication stress phenotype rather than a telomere-restricted defect. Nevertheless, we note that the absence of detectable telomeric enrichment does not exclude the presence of ssDNA gaps at ALT telomeres, given their small contribution to total genomic DNA. Consistent with this interpretation, iPOND-MS analysis identified enrichment of lagging-strand replication factors (FEN1 and Ligase 1) specifically in SLX4IP-deficient ALT-positive cells

(Figure 2E), suggesting heightened lagging-strand vulnerability. Supporting a functional link, SLX4IP telomeric accumulation increased upon Ligase 1 depletion, whereas FEN1 co-depletion did not exacerbate telomeric RPA phosphorylation or growth defects, indicating that SLX4IP operates in a pathway that overlaps with lagging-strand replication stress (Expanded View 4L-Q). Collectively, these findings support a model in which SLX4IP loss induces global replication stress while ALT telomeres display a particular sensitivity linked to lagging-strand processing.

• Does FEN1 knockdown reverse the heightened replication stress and unligated Okazaki fragments caused by SLX4IP/FANCM loss to similar levels as BLM knockdown?

We thank the reviewer for the insightful comment, which has important mechanistic implications for how we view SLX4IP function at the replication fork. In the revised manuscript, we present new data showing that FEN1 knockdown does not augment replication stress, APB formation, or synthetic lethality in SLX4IP-deficient cells, indicating that SLX4IP and FEN1 are epistatic with respect to replication stress (Expanded View 4NQ).

Based on our ADPr and S1 nuclease analyses (which are not telomere-specific), SLX4IP acts globally to maintain replication fork stability. Consistently, we now present data showing increased ADPr foci not only in ALT-positive U2OS but also in SLX4IP-deficient, ALT-negative RPE1-hTert cells (Expanded View 3DE). We did not observe this phenotype in eHAP cells (Expanded View 3FG), likely reflecting the distinct replication fork collapse signature observed in the eHAP iPOND-MS dataset (Expanded View 2J-L). Importantly, while we do not observe a strong enrichment of ADPr foci specifically at ALT telomeres (Extented View 4HI), at least at the detection level of our assay, ALT telomeric regions may still be vulnerable to fork destabilization based on the increase in ATR signalling at telomeres and consistent with SLX4IP's broader role in fork protection.

Based on our new data on both FEN1 and ADPr levels, we propose the following revised model, intentionally avoiding overinterpretation of a direct role for SLX4IP specifically at the lagging-strand: SLX4IP primarily functions as a regulator of replication fork stability, most likely by limiting ssDNA gap formation and facilitating proper processing of replication intermediates, rather than directly controlling lagging-strand DNA synthesis. Loss of SLX4IP destabilizes forks and generates intermediates that require FEN1-mediated processing, consistent with the observed epistasis between SLX4IP and FEN1. Accordingly, the phenotypes observed upon SLX4IP loss are best interpreted as consequences of replication stress rather than intrinsic defects in lagging-strand DNA synthesis. We discuss this interpretation in the revised manuscript (lines 457ff).

• Lines 123 and 199, FANCM binds to RMI1/2 rather than directly to BLM

We have corrected this in the revised manuscript (line 315f).

• Line 220/221 - "modest"? (word missing).

We have corrected this in the revised manuscript (line 352f).

Referee #3 (Report for Author)

Spindler et al. identify roles of SLX4IP in regulating the BLM helicase during DNA replication. The authors find in U2OS ALT cancer cells that SLX4IP KO clones have higher rates of telomere fragility and reduced replication fork speed throughout the genome. The replication stress marker pSer33RPA was increased at telomeres and pSer345CHK1 foci were also detected. HU-treatment led to SLX4IP accumulation at many loci in the genome as determined by cut&tag profiling indicating genome-wide roles of SLX4IP in response to replication stress. Depletion of the DNA translocase FANCM with SLX4IP-KO showed additive effects on replication stress at telomeres as well as synthetic growth defects. FANCM has been reported before to reduce replication stress at ALT telomeres. Published work also demonstrated that the reduced viability of FANCM-deficient ALT cells depends on BLM. It is reported here that the same is true for SLX4IP. Depletion of BLM in SLX4IP-deficient cells rescued replication stress as well as growth defects. Altogether, SLX4IP appears to prevent the toxic effects of excessive BLM activity. Determination of protein composition near replication forks by iPOND revealed among others enrichment of the flap endonuclease FEN1. Furthermore, by combining DNA combing analysis with S1 nuclease treatment it was found that single stranded gaps accumulated in the analyzed SLX4IP-KO clone. Poly(ADP) ribosylation also increased in the nucleus, which depended on BLM. Altogether the data suggest that loss of SLX4IP leads to defects in lagging strand synthesis throughout the genome by mis-regulation of BLM.

We thank the reviewer for their careful evaluation of our manuscript and for their constructive comments.

Below, we provide a point-by-point response to the reviewer's specific concerns.

Major concern and comments:

• Clonal variation of telomere phenotypes is a common phenomenon which has been described in several publications. For example, telomere length varies substantially between clones of telomerase-positive cells. Clonal variation for other telomere phenotypes is also highly likely. Consistent with this notion, the two SLX4IP-KO clones show different levels of telomere fragility and marked differences in the other phenotypes described in the manuscript. For replication fork speed and single stranded gaps, only one clone is analyzed. I'm doubtful that the analysis of two or one clone is sufficient to exclude that the effects are not due to clonal variation. In order to achieve convincing results, the sample size of KO-clones must may be significantly increased or it must be demonstrated that expression of wild SLX4IP (e.g. from a cDNA) in the KO-background can rescue the phenotypes. Perhaps the latter experiment is less work.

We thank the reviewer for raising this important point regarding potential clonal variation. We fully agree that clonal variability is a well-recognized phenomenon, and we appreciate the reviewer's suggestion to strengthen our analyses.

To directly address this concern, we have substantially expanded our experimental validation beyond the initially presented clones. Specifically, we repeated the iPOND, and DNA combing assays, in three independent cellular backgrounds, U2OS, RPE1-hTERT, and eHAP cellsFor each cell line, two independently derived SLX4IP knockout clones were analyzed (Figure 2C-F, Expanded View 2H-L). In addition, we also repeated the and S1 nuclease assays in two independent knockout clones. Importantly, replication-associated phenotypes were reproducibly observed across clones and cellular contexts, including reduced replication fork speed, accumulation of S1-sensitive single-stranded DNA gaps, and increased replication stress signaling (Figure 2AB, Expanded View 2F, Figure 3A-C, Expanded View 5E-G). These results demonstrate that the observed replication defects are not restricted to a single clone and cannot be explained by clone-specific variation.

We further expanded the telomere-focused analyses by examining additional independently derived SLX4IP knockout clones in U2OS cells as well as SLX4IP-deficient WI38-VA13 cells (Panier et al., Mol Cell 2019), and a pooled SLX4IP knockout SAOS-2 population (Expanded View 4BC, Expanded View 5L-Q). Notably, across these distinct experimental systems, we consistently recapitulated the central phenotypes described in the manuscript, including increased telomeric pSer33-RPA, elevated nuclear ADP-ribosylation, and synthetic growth defects upon FANCM depletion. The pooled SAOS-2 population is particularly informative in this context, as it excludes clonal variability as a confounding factor.

Together, these additional experiments demonstrate that both the replication stress phenotypes and the telomere-associated defects represent robust and reproducible consequences of SLX4IP loss rather than clone-specific effects.

• The results indicate that SLX4IP restricts BLM activity during replication not only at telomeres but throughout the genome. Although the project may have initially been aimed at investigating specific roles of SLX4IP at ALT telomeres the results indicate much broader roles of SLX4IP. Therefore, I recommend a considerable overhaul of the writing and emphasis of the paper. The title does also not reflect the general roles of SLX4IP in DNA replication which are discovered here. The paper might also reach a broader readership by addressing the general DNA replication community.

We thank the reviewer for this insightful comment and fully agree with the underlying assessment. While the initial focus of the manuscript was on SLX4IP function at ALT telomeres, our results indeed revealed a broader role of SLX4IP in the regulation of replication stress across the genome.

Consistent with the reviewer's suggestion, we have extensively restructured the manuscript to improve clarity and logical progression. The revised Results section now first defines SLX4IP as a regulator of replication stress responses in a general genomic context (Revised Figures 1-3), including its role in limiting replication-associated stress and its functional relationship with BLM. We then demonstrate that ALT telomeres represent a genomic environment in which this SLX4IP-dependent function becomes particularly critical (Revised Figure 4). Finally, we position SLX4IP within a pathway operating in parallel to FANCM (Revised Figure 5) and show that the synthetic growth defects observed upon combined SLX4IP and FANCM loss arise from deregulated BLM activity (Revised

Figure 6). This reorganization clarifies the conceptual hierarchy of the findings and aligns the manuscript with the broader biological implications of the manuscript

In line with this refocusing, we have revised the title to: "SLX4IP limits replication stress globally and at ALT telomeres"

We believe that these revisions substantially improve the conceptual clarity of the manuscript and broaden its relevance to the DNA replication community, while preserving the significance of the ALT-related findings.

• It is found that loss of SLX4IP is synthetic lethal with depletion of FANCM in U2OS ALT cancer cells. However, it is unclear if this effect depends on the unusual telomere structure of ALT cells or if it is due to general defects in DNA replication. Therefore, the analysis should also be carried out in non-ALT cells.

To determine whether the synthetic lethality between SLX4IP loss and FANCM depletion is ALT-specific, we repeated the analysis in non-ALT RPE1-hTERT cells (Expanded View 5RS). In contrast to U2OS cells, FANCM depletion did not result in synthetic lethality in SLX4IP-deficient RPE1-hTERT cells. These results indicate that the synthetic growth defect is context-dependent and support the conclusion that ALT cells are particularly vulnerable to combined SLX4IP and FANCM deficiency.

Other comments:

• Page 9, Expanded Fig. 1D: I doubt that it is justified to specify association with specific regions of the genome with 2 digits after the comma. The measurements are not precise enough. The numbers should be rounded.

We have amended this in revised Figure 1A (old Expanded View 1D).

• Page 3, line 46 : perhaps write that the combined loss of SLX4IP and FANCM shows synthetic lethality.

We have corrected this in the revised manuscript (line 52f).

Dr. Stephanie Panier
Max Planck Institute for Biology of Ageing
Panier
Joseph-Stelzmann-Str 9b
Cologne 50931
Germany

9th Apr 2026

Re: EMBOJ-2025-121677R
SLX4IP limits replication stress globally and at ALT telomeres

Dear Stephanie,

Thank you for submitting your revised manuscript to our editorial office. It has now been re-reviewed by the three original referees, and I am happy to say that all of them found the study significantly improved and now suitable for publication. Prior to final acceptance, there are only a few important editorial issues that I need to ask you for:

- The referees noted a few minor corrections to be incorporated, see below.

- Our routine pre-acceptance image checks indicated that (parts of) some microscopy images are re-displayed in separate figures (Fig. 3B and Fig EV4I, Fig 6C and Fig EV6D), without any mention/explanation of this in the respective figure legends. Please, carefully check and clarify this, and either correct or explicitly state and rationalize in each respective legend.

- Please upload each main and EV figure as individual single-page files. Note that EV figures do not have to be linked to particular main figures, so that multi-page EV figures might be better split up into separate, additional EV figures. Additionally, please check that each figure panel is referenced at least once in the text (some callouts are currently missing), and that all callouts appear sequentially - if necessary, you may first call-out the complete figure (e.g., Fig 3A-E) before referencing individual panels. Finally, please make sure to stick to the nomenclature 'Fig. 3A,B' and 'Fig. EV4D' instead of 'Expanded View 4D'.

- Please adjust the order of the manuscript sections: Title page with complete author information, Abstract, Keywords, Introduction, Results, Discussion, Methods, Data Availability, Acknowledgements, Disclosure and Competing Interests Statement, References, Main Figure Legends, Tables, Expanded Figure Legends.

- Please double-check to make sure to all relevant funding information in the manuscript is congruent with the info entered into our submission system. Currently missing in the submission system are: Center for Molecular Medicine Cologne grant/ project number JRG-X; Ministry of Culture and Science of the State of North-Rhine-Westphalia "Netzwerke 2021" grant/ project number CANTAR

- Please rename the Conflict of Interest section into "Disclosure and Competing Interests Statement", in accordance with our updated Guide to Authors (https://www.embopress.org/competing-interests)

- As we are switching from a free-text author contribution statement towards a more formal statement based on Contributor Role Taxonomy (CRediT) terms, please remove the present Author Contribution section and instead specify each author's contribution(s) directly in the Author Information page of our submission system during upload of the final manuscript. See https://casrai.org/credit/ for more information.

- Please move the Reagents and Tools table from the main article file, and upload it as a separate text file. Also, please make sure to adhere to the template table downloadable from our author guidelines:
https://www.embopress.org/page/journal/14693178/authorguide#structuredmethods

- Please carefully go through the reference list, which contains several entries lacking full citation information such as volume or page/eLocator numbers. Please make sure that for references with multiple authors, the first up to 10 authors should be listed, followed by 'et al.' after that (please refer to our Guide to Authors for additional information on EMBO J reference format).

- In the provided Source Data files, please make sure to properly label the corresponding panels of Figure 2 in the respective Source Data file folder.

- Please provide suggestions for a short 'blurb' text prefacing and summing up the study in two sentences (max. 250 characters), followed by 3-5 one-sentence 'bullet points' (maybe using the 'Highlights' currently included in the manuscript text itself); they

will form the basis of an editor-written 'Synopsis' accompanying the online version of the article. Please also upload a synopsis image, which can be used as a "visual title" for the synopsis section of your paper (maybe based on the schematics in Fig 7?). The image should be in JPG format with the modest dimensions of exactly 550 pixels wide and 300 pixels high.

- Finally, our data editors have raised the following queries regarding figures, data, and legends; I would appreciate if you briefly answered to them in the cover letter of your final submission, and made the requested text modifications with changes/additions highlighted via the "Track changes" option, to facilitate our final checking:
1. For figures 2E, F, EV2 L, EV4 G, V, in which data from less than 3 replicates is displayed, please remove any error bars and make sure to plot the individual data points instead of an average
2. Please describe entity for 'n' (e.g., biological or technical replicates) in the legends of figures 2A, B; 6F, EV2 F, G; EV5 M, P, Q, R, S, T, U, V, W, Y, Z; EV6 A-C
3. Please define the annotated p values ****/***/**/* as well as provide the exact p-values for the same in the legend of figure 1B as appropriate.
4. Please define the exact p values in the legends of figures 1F, 2A, B; 3A, C, E; 4B, D, F; 5B, D, F, H; 5B, D, F, H; 6B, D, F; EV1 D, EV2 F, G; EV3 C, E, G, H; EV4 C, E, H L, M, N, O, P, Q, R, T; 5F, G, H, I, J, L; M, N, P, Q, R, U, V, W, Y, Z; EV6 A, B, C, E.
5. Please indicate the statistical test used for data analysis in the legend of figure 1B

I am therefore returning the manuscript to you for a final round of minor revision, to allow you to make these modifications and upload the revised files. Once we will have received them, we should hopefully be ready to swiftly proceed with formal acceptance and production of the manuscript.

With kind regards,

Hartmut

Hartmut Vodermaier, PhD
Senior Editor, The EMBO Journal
h.vodermaier@embojournal.org

*** PLEASE NOTE: All revised manuscript are subject to initial checks for completeness and adherence to our formatting guidelines. Revisions may be returned to the authors and delayed in their editorial re-evaluation if they fail to comply to the following requirements. As a first step please read our guidelines for revised submissions:
https://link.springer.com/journal/44318/submission-guidelines#cms-Revised-submissions

1) Every manuscript requires a Data Availability section (even if only stating that no deposited datasets are included). Primary datasets or computer code produced in the current study have to be deposited in appropriate public repositories prior to resubmission, and reviewer access details provided in case that public access is not yet allowed.

2) Each figure legend must specify
- size of the scale bars that are mandatory for all micrograph panels
- the statistical test used to generate error bars and P-values
- the type error bars (e.g., S.E.M., S.D.)
- the number (n) and nature (biological or technical replicate) of independent experiments underlying each data point
- Figures may not include error bars for experiments with n<3; scatter plots showing individual data points should be used instead.

3) Revised manuscript text (including main tables, and figure legends for main and EV figures) has to be submitted as editable text file (e.g., .docx format). We encourage highlighting of changes (e.g., via text color) for the referees' reference.

4) Each main and each Expanded View (EV) figure should be uploaded as individual production-quality files (preferably in .eps, .tif, .jpg formats). For suggestions on figure preparation/layout, please refer to our Figure Preparation Guidelines:
https://media.springernature.com/original/springer-cms/rest/v1/content/27825798/data/v1

5) Point-by-point response letters should include the original referee comments in full together with your detailed responses to them (and to specific editor requests if applicable), and also be uploaded as editable (e.g., .docx) text files.

6) Please complete our Author Checklist, and make sure that information entered into the checklist is also reflected in the manuscript; the checklist will be available to readers as part of the Review Process File.

7) All authors listed as (co-)corresponding need to deposit, in their respective author profiles in our submission system, a unique ORCiD identifier linked to their name. Please see our Guide to Authors for detailed instructions.

8) Please note that supplementary information at EMBO Press has been superseded by the 'Expanded View' for inclusion of additional figures, tables, movies or datasets; with up to five EV Figures being typeset and directly accessible in the HTML version of the article.

9) To facilitate reproducibilty and cross-laboratory adoption of methodologies, please structure the Materials & Methods section as outlined in our guide to authors, including a completed Reagents and Tools Table.

10) Digital image enhancement is acceptable practice, as long as it accurately represents the original data and conforms to community standards. If a figure has been subjected to significant electronic manipulation, this must be clearly noted in the figure legend and/or the 'Materials and Methods' section. The editors reserve the right to request original versions of figures and the original images that were used to assemble the figure. Finally, we generally encourage uploading of numerical as well as gel/blot image source data.

At EMBO Press we ask authors to provide source data for the main manuscript figures. You will receive a separate email with instructions for providing source data with your revised manuscript, including how to upload and organize the files.

In the interest of ensuring the conceptual advance provided by the work, we recommend submitting a revision within 3 months (8th Jul 2026). Please discuss the revision progress ahead of this time with the editor if you require more time to complete the revisions. Use the link below to submit your revision:

Link Not Available

Referee #1:

The authors have done a very thorough job in addressing the queries and recommendations in the prior review. The re-orienting of the papers emphasis works nicely and reads well. The work is solid and conclusions rational. Even though the net gain in knowledge that this study provides remains a bit limited and there remains much that is unknown about the mechanism by which SLX4IP regulates DNA replication and telomere maintenance, the study is important and sets the stage for further advances.

Referee #2:

The authors have completed extensive additional experiments/analysis to thoroughly and comprehensively address the reviewers' comments. This has substantially improved the manuscript. In particular, delineation of the genomic vs telomeric role of SLX4IP has provided mechanistic clarity and strengthened the conclusions. It is a lovely manuscript and I support publication.
Minor typographical errors that I noticed:
Line 173: "...but not in ALT-negative cells."
Line 188: remove "in"
Line 263 "...mirrors the fork..."
Line 383: SLX4IP

Referee #3:

The authors have revised the paper very carefully. All my comments are very well addressed.

EMBO Press Author Checklist

Corresponding Author Name: Stephanie Panier	
Journal Submitted to: The EMBO Journal	
Manuscript Number: EMBOJ-2025-121677R	

USEFUL LINKS FOR COMPLETING THIS FORM
The EMBO Journal - Author Guidelines
EMBO Reports - Author Guidelines
Molecular Systems Biology - Author Guidelines
EMBO Molecular Medicine - Author Guidelines

Reporting Checklist for Life Science Articles (updated January
This checklist is adapted from Materials Design Analysis Reporting (MDAR) Checklist for Authors. MDAR establishes a minimum set of requirements in transparent reporting in the life sciences (see Statement of Task: 10.31222/osf.io/9sm4x). Please follow the journal's guidelines in preparing your
Please note that a copy of this checklist will be published alongside your article.

Abridged guidelines for figures
1. Data
The data shown in figures should satisfy the following conditions:
- the data were obtained and processed according to the field's best practice and are presented to reflect the results of the experiments in an accurate and unbiased manner.
- ideally, figure panels should include only measurements that are directly comparable to each other and obtained with the same assay.
- plots include clearly labeled error bars for independent experiments and sample sizes. Unless justified, error bars should not be shown for technical
- if n<5, the individual data points from each experiment should be plotted. Any statistical test employed should be justified.
- Source Data should be included to report the data underlying figures according to the guidelines set out in the authorship guidelines on Data

2. Captions
Each figure caption should contain the following information, for each panel where they are relevant:
- a specification of the experimental system investigated (eg cell line, species name).
- the assay(s) and method(s) used to carry out the reported observations and measurements.
- an explicit mention of the biological and chemical entity(ies) that are being measured.
- an explicit mention of the biological and chemical entity(ies) that are altered/varied/perturbed in a controlled manner.
- the exact sample size (n) for each experimental group/condition, given as a number, not a range;
- a description of the sample collection allowing the reader to understand whether the samples represent technical or biological replicates (including how many animals, litters, cultures, etc.).
- a statement of how many times the experiment shown was independently replicated in the laboratory.
- definitions of statistical methods and measures:
  - common tests, such as t-test (please specify whether paired vs. unpaired), simple χ2 tests, Wilcoxon and Mann-Whitney tests, can be unambiguously identified by name only, but more complex techniques should be described in the methods section;
  - are tests one-sided or two-sided?
  - are there adjustments for multiple comparisons?
  - exact statistical test results, e.g., P values = x but not P values < x;
  - definition of 'center values' as median or average;
  - definition of error bars as s.d. or s.e.m.

Please complete ALL of the questions below.
Select "Not Applicable" only when the requested information is not relevant for your study.

Materials

Newly Created Materials	Information included in the manuscript?	In which section is the information available? (Reagents and Tools Table, Materials and Methods, Figures, Data Availability Section)
New materials and reagents need to be available; do any restrictions apply?	Yes	Materials and Methods, Reagents and Tools Table

Antibodies	Information included in the manuscript?	In which section is the information available? (Reagents and Tools Table, Materials and Methods, Figures, Data Availability Section)
For **antibodies** provide the following information:<br>- Commercial antibodies: RRID (if possible) or supplier name, catalogue number and or/clone number<br>- Non-commercial: RRID or citation	Yes	Reagents and Tools Table

DNA and RNA sequences	Information included in the manuscript?	In which section is the information available? (Reagents and Tools Table, Materials and Methods, Figures, Data Availability Section)
**Short novel DNA or RNA including primers, probes**: provide the sequences.	Yes	Reagents and Tools Table

Cell materials	Information included in the manuscript?	In which section is the information available? (Reagents and Tools Table, Materials and Methods, Figures, Data Availability Section)
**Cell lines:** Provide species information, strain. Provide accession number in repository **OR** supplier name, catalog number, clone number, and/**OR** RRID.	Yes	Reagents and Tools Table
**Primary cultures:** Provide species, strain, sex of origin, genetic modification status.	Not Applicable	
Report if the cell lines were recently **authenticated** (e.g., by STR profiling) and tested for mycoplasma contamination.	Yes	Materials and Methods

Experimental animals	Information included in the manuscript?	In which section is the information available? (Reagents and Tools Table, Materials and Methods, Figures, Data Availability Section)
**Laboratory animals or Model organisms:** Provide species, strain, sex, age, genetic modification status. Provide accession number in repository **OR** supplier name, catalog number, clone number, **OR** RRID.	Not Applicable	
**Animal observed in or captured from the field:** Provide species, sex, and age where possible.	Not Applicable	
Please detail **housing and husbandry conditions**.	Not Applicable	

Plants and microbes	Information included in the manuscript?	In which section is the information available? (Reagents and Tools Table, Materials and Methods, Figures, Data Availability Section)
**Plants:** provide species and strain, ecotype and cultivar where relevant, unique accession number if available, and source (including location for collected wild specimens).	Not Applicable	
**Microbes:** provide species and strain, unique accession number if available, and source.	Not Applicable	

Human research participants	Information included in the manuscript?	In which section is the information available? (Reagents and Tools Table, Materials and Methods, Figures, Data Availability Section)
If collected and within the bounds of privacy constraints report on age, sex and gender or ethnicity for all study participants.	Not Applicable	

Core facilities	Information included in the manuscript?	In which section is the information available? (Reagents and Tools Table, Materials and Methods, Figures, Data Availability Section)
If your work benefited from core facilities, was their service mentioned in the acknowledgments section?	Yes	Acknowledgements Section

Design

Study protocol	Information included in the manuscript?	In which section is the information available? (Reagents and Tools Table, Materials and Methods, Figures, Data Availability Section)
If study protocol has been **pre-registered, provide DOI in the manuscript**. For clinical trials, provide the trial registration number **OR** cite DOI.	Not Applicable	
Report the **clinical trial registration number** (at ClinicalTrials.gov or equivalent), where applicable.	Not Applicable	

Laboratory protocol	Information included in the manuscript?	In which section is the information available? (Reagents and Tools Table, Materials and Methods, Figures, Data Availability Section)
Provide DOI OR other citation details if **external detailed step-by-step protocols** are available.	Not Applicable	

Experimental study design and statistics	Information included in the manuscript?	In which section is the information available? (Reagents and Tools Table, Materials and Methods, Figures, Data Availability Section)
Include a statement about **sample size** estimate even if no statistical methods were used.	Yes	Figure Legends
Were any steps taken to minimize the effects of subjective bias when allocating animals/samples to treatment (e.g. **randomization procedure**)? If yes, have they been described?	Yes	Materials and Methods
Include a statement about **blinding** even if no blinding was done.	Yes	Materials and Methods
Describe **inclusion/exclusion criteria** if samples or animals were excluded from the analysis. Were the criteria pre-established?<br><br>If sample or data points were omitted from analysis, report if this was due to attrition or intentional exclusion and provide justification.	Not Applicable	
For every figure, are **statistical tests** justified as appropriate? Do the data meet the assumptions of the tests (e.g., normal distribution)? Describe any methods used to assess it. Is there an estimate of variation within each group of data? Is the variance similar between the groups that are being statistically compared?	Yes	Figure Legends

Sample definition and in-laboratory replication	Information included in the manuscript?	In which section is the information available? (Reagents and Tools Table, Materials and Methods, Figures, Data Availability Section)
In the figure legends: state number of times the experiment was **replicated** in laboratory.	Yes	Figure Legends
In the figure legends: define whether data describe **technical or biological replicates**.	Yes	Figure Legends

Ethics

Ethics	Information included in the manuscript?	In which section is the information available? (Reagents and Tools Table, Materials and Methods, Figures, Data Availability Section)
Studies involving **human participants**: State details of **authority granting ethics approval** (IRB or equivalent committee(s), provide reference number for approval.	Not Applicable	
Studies involving **human participants**: Include a statement confirming that **informed consent** was obtained from all subjects and that the experiments conformed to the principles set out in the WMA Declaration of Helsinki and the Department of Health and Human Services Belmont Report.	Not Applicable	
Studies involving **human participants:** For publication of **patient photos**, include a statement confirming that consent to publish was obtained.	Not Applicable	
Studies involving experimental **animals**: State details of **authority granting ethics approval** (IRB or equivalent committee(s), provide reference number for approval. Include a statement of compliance with ethical regulations.	Not Applicable	
Studies involving **specimen and field samples:** State if relevant **permits** obtained, provide details of authority approving study; if none were required, explain why.	Not Applicable	

Dual Use Research of Concern (DURC)	Information included in the manuscript?	In which section is the information available? (Reagents and Tools Table, Materials and Methods, Figures, Data Availability Section)
Could your study fall under dual use research restrictions? Please check biosecurity documents and list of **select agents and toxins** (CDC): https://www.selectagents.gov/sat/list.htm	Not Applicable	
If you used a select agent, is the security level of the lab appropriate and reported in the manuscript?	Not Applicable	
If a study is subject to dual use research of concern regulations, is the name of the **authority granting approval and reference number** for the regulatory approval provided in the manuscript?	Not Applicable	

Reporting

The MDAR framework recommends adoption of discipline-specific guidelines, established and endorsed through community initiatives. Journals have their own policy about requiring specific guidelines and recommendations to complement MDAR.

Adherence to community standards	Information included in the manuscript?	In which section is the information available? (Reagents and Tools Table, Materials and Methods, Figures, Data Availability Section)
State if relevant guidelines or checklists (e.g., **ICMJE, MIBBI, ARRIVE, PRISMA**) have been followed or provided.	Not Applicable	
For **tumor marker prognostic studies,** we recommend that you follow the **REMARK** reporting guidelines (see link list at top right). See author guidelines, under 'Reporting Guidelines'. Please confirm you have followed these guidelines.	Not Applicable	
For **phase II and III randomized controlled trials**, please refer to the **CONSORT** flow diagram (see link list at top right) and submit the CONSORT checklist (see link list at top right) with your submission. See author guidelines, under 'Reporting Guidelines'. Please confirm you have submitted this list.	Not Applicable	

Data Availability

Data availability	Information included in the manuscript?	In which section is the information available? (Reagents and Tools Table, Materials and Methods, Figures, Data Availability Section)
Have **primary datasets** been deposited according to the journal's guidelines (see 'Data Deposition' section) and the respective accession numbers provided in the Data Availability Section?	Yes	Data Availability Section
Were **human clinical and genomic datasets** deposited in a public access-controlled repository in accordance to ethical obligations to the patients and to the applicable consent agreement?	Not Applicable	
Are **computational models** that are central and integral to a study available without restrictions in a machine-readable form? Were the relevant accession numbers or links provided?	Not Applicable	
If publicly available data were reused, provide the respective **data citations in the reference list**.	Yes	Reference List
